# Revision of the ant genus *Melophorus* (Hymenoptera, Formicidae)

**DOI:** 10.3897/zookeys.700.11784

**Published:** 2017-09-20

**Authors:** Brian E. Heterick, Mark Castalanelli, Steve O. Shattuck

**Affiliations:** 1 Curtin University of Technology, GPO Box U1987, Perth WA, Australia, 6845; 2 Western Australian Museum, Locked Bag 49, Welshpool DC. WA, Australia, 6986; 3 EcoDiagnostics Pty Ltd, 48 Banksia Rd, Welshpool WA 6106; 4 C/o CSIRO Entomology, P. O. Box 1700, Canberra, Australia, ACT 2601

**Keywords:** Hymenoptera, Formicidae, Formicinae, *Melophorus*, taxonomy, new species, Australia.

## Abstract

The fauna of the purely Australian formicine ant genus *Melophorus* (Hymenoptera: Formicidae) is revised. This project involved integrated morphological and molecular taxonomy using one mitochondrial gene (COI) and four nuclear genes (AA, H3, LR and Wg). Seven major clades were identified and are here designated as the *M. aeneovirens*, *M. anderseni*, *M. biroi*, *M. fulvihirtus*, *M. ludius*, *M. majeri* and *M. potteri* species-groups. Within these clades, smaller complexes of similar species were also identified and designated species-complexes. The *M. ludius* species-group was identified purely on molecular grounds, as the morphology of its members is indistinguishable from typical members of the *M. biroi* species-complex within the *M. biroi* species-group. Most species-complexes sampled were also found to be monophyletic. Sequencing generally supported monophyly in taxa sampled but some species of the *M. fieldi* complex and *M. biroi* were not monophyletic and the implications arising from this are discussed in this monograph. Based on morphology, ninety-three species are recognized, 73 described as new. A further new species (here called 'Species K' [TERC Collection]) is noted in the taxonomic list, but is not described in this work. One species is removed from *Melophorus*: *M. scipio* Forel is here placed provisionally in *Prolasius*. Six species and five subspecies pass into synonymy. Of the full species, *M. constans* Santschi, *M. iridescens* (Emery) and *M. insularis* Wheeler are synonymized under *M. aeneovirens* (Lowne), *M. pillipes* Santschi is synonymized under *M. turneri* Forel, *M. marius* Forel is synonymized under *M. biroi* Forel, and *M. omniparens* Forel is synonymized under *M. wheeleri* Forel. Of the subspecies, *M. iridescens fraudatrix* and *M. iridescens froggatti* Forel are synonymized under *M. aeneovirens* (Lowne), *M. turneri aesopus* Forel and *M. turneri candidus* Santschi are synonymized under *M. turneri* Forel and *M. fieldi propinqua* Viehmeyer is synonymized under *M. biroi*. *Camponotus cowlei* Froggatt is reinstated as a junior synonym of *Melophorus bagoti* Lubbock. In addition, the subspecies *M. fieldi major* Forel, *M. ludius sulla* Forel and *M. turneri perthensis* Forel are raised to species. A key to workers of the genus is supplied. A lectotype is designated for *M. curtus* Forel, *M. sulla*, and *M. turneri*.

## Introduction

*Melophorus* is an easily characterized member of the ant subfamily Formicinae. This genus shares with all formicines the possession of an acidipore (a tubular structure that disseminates formic acid against enemies). The genus *Melophorus* is entirely restricted to Australia, and all known members are thermophilic or believed to be predominantly so, mainly being terrestrial foragers. The very many species of *Melophorus* are particularly abundant in hot, arid or semi-arid environments where they are diurnally active, mostly in the summer months. Arguably, *Melophorus* is one of the most poorly understood large ant genera in the world. Just a fraction of the constituent species has been described to the present time, the group has never been monographed, and synonymy is prevalent among the 32 taxa that have received names. Here we provide the first comprehensive monograph of the genus. Because this group is so little known, the likely total number of species has been regarded as problematic. Figures as high as 1000 species (Andersen 2007) have been suggested, but this monograph indicates the real figure is around one tenth of that.

**Taxonomic history.** The genus *Melophorus* was originally erected for the single species *Melophorus bagoti* (Lubbock 1883). However, a species described earlier in the genus *Formica* was later revealed to be a *Melophorus* (*M. aeneovirens* [Lowne 1865]). Lubbock’s diagnosis of the genus was poor and initially *Melophorus* lacked any real systematic framework. As a result, *Melophorus* was initially very broadly defined and accumulated species, including non-Australian species, that are now placed in other genera.

Forel (1911) had already commented on the diverse morphology of *Melophorus* that caused many of the then known species to be confused with unrelated genera or extralimital genera (i.e. those occurring outside of Australia). However, based on the appearance of the proventriculus, he affirmed their basic unity at the generic level. In 1912, he made a more determined effort to formally link the overly broad ‘*Melophorus*’ with taxa that seemed to him to share significant apomorphies (Forel 1912). In this work, ‘*Melophorus*’ was placed in a subfamily titled Camponotinae (originally erected by Forel in 1878 as ‘’ and later [1893] corrected by him). Forel divided the Camponotinae into three assemblages of taxa, based principally on the proventriculus. He also provided all-caste keys to the formicine genera known at the time. ‘*Melophorus*’ found a home in the second assemblage, the so-called Mesocamponotinae, and in a tribe, Melophorini, that consisted of *Melophorus* and another Australian genus, *Notoncus*. These two genera were distinguished from other formicines by their 12-segmented antennae and (with the exception of the ‘Australian *Melophorus*’!) the presence of a discoidal cell in the wing of the sexuals.

Within the first two decades of the twentieth century most of the species wrongly placed in *Melophorus* were correctly reassigned by Forel and another great myrmecologist of his era, Carlo Emery. Similarly, species such as *M. aeneovirens* were incorporated into *Melophorus*. Wheeler (1920) replaced the old subfamily name Camponotinae with Formicinae, based on his work on larval characters, and established the foundations of a subfamily classification that remained substantially unchanged (with just a few additions) until the modern era of molecular systematics.

By 1922, the taxonomic status of *Melophorus* as a well-defined genus in its own right was gradually starting to emerge, although in that year Carlo Emery still subsumed genuine *Melophorus* species (along with several *Prolasius* species and *Notoncus spinisquamis* André) under a subgenus ‘*Melophorus*’ (Emery 1922). ‘*Prolasius*’ and ‘*Lasiophanes*’ constituted the remaining two subgenera under the broader umbrella genus *Melophorus*. Interestingly, *Notoncus* was treated by Emery as a discrete, though closely related genus. In Emery’s treatment, all non-Australian taxa had now been removed from the subgenus ‘*Melophorus*’, although of the diagnostic characters that Emery listed for workers of the subgenus, only polymorphism would now be recognized as a useful character.

William Morton Wheeler (1935) made another major advance in rearranging the structure of the Melophorini, an arrangment largely retained in modern thinking on the group. *Lasiophanes*, *Melophorus* and *Prolasius* (the last including, however, two *Notoncus* species) assumed full generic status. The remaining genera in the tribe Melophorini were *Diodontolepis* (including *Notoncus spinisquamis* only), most of the remaining *Notoncus* under the genus of that name, *Pseudonotoncus* and *Myrmecorhynchus*. Wheeler removed a number of names from the existing genus *Melophorus* and reassigned them to *Prolasius*. Wheeler also tackled variation within *Melophorus* by erecting three subgenera: *Melophorus* ***sensu stricto*** for the gracile forms like *M. aeneovirens* (Lowne) that resemble the American honey-pot ants in the genus *Myrmecocystus*, *Erimelophorus* for the large-headed forms and *Trichomelophorus* for the peculiar *M. hirsutus* Forel.

The last major contribution to the taxonomy of *Melophorus* in its own right was made by Brown (1955). He dismissed Wheeler’s three subgenera on the basis of structure and habits and formally synonymized them. He also drew attention to two important diagnostic characters for the genus, namely, the long ‘ammochaetae’ (psammophore) together with the J-shaped setae on the mentum and also the elongate propodeal spiracle.

Since 1955 there has been no serious taxonomic consideration of the genus. Andersen (2000), in a field guide, provided a key to northern *Melophorus* that included very many groups. These groups appear to have been chosen on phenetic grounds, and the characters used, e.g., sculpture, pilosity, etc. were mainly those that have little validity at the species-group or subgenus level. A few characters used, such as length of the palps, have more significance, and they are discussed later in this work. A later discussion (Andersen 2007), evidently developed from the first (i.e., Andersen 2000), and covering a broader suite of *Melophorus*, is much more substantial, and (to our minds) accurately identifies some apomorphies that set apart several, although not all, significant species-groups that we recognize. In this case, Andersen subsumed such groupings under broader ‘radiations’. His identification of the *M. fulvihirtus*, the *M. potteri* and the *M. aeneovirens* species-groups are supported in this work, as, in general terms, are the characters used to differentiate them, viz., appearance of the palps, the presence or absence of metatibial apical spurs, and the clypeal apron (in the *aeneovirens* species-group). Nonetheless, not all the characters mentioned are correctly applied (e.g., the metatibial spur is not absent in *M. fulvihirtus*, as stated by Andersen). Other important characters, such as the placement of the clypeal psammophore, the shape and dentition of the mandible and the nature of the preapical metatibial spines, are discussed in this work.

Apart from Andersen’s two works, there has been little else on the genus since 1955. Two rather aberrant and interesting *Melophorus*, *M. anderseni* and *M. majeri*, were described by Agosti in 1997, the first new species to be described since *Melophorus bruneus* McAreavey in 1949. No *Melophorus* species have been described since 1997.

At a higher taxonomic level, Agosti (1991) used the unfused helcium (the collar-like pre-tergite and pre-sternite of the third gastral segment) and the close alignment of the hind coxae to place *Melophorus* in the *Formica* species-group. However, Bolton (2003) reverted to the traditional morphological argument based on the anatomy of the proventriculus, along with details of the mandible and metatibial setae, to situate *Melophorus* as the sole genus in the Tribe Melophorini. Recent molecular work (Moreau et al. 2006 using five nuclear genes and one mitochondrial gene) strongly supported an Australoid formicine clade comprising *Myrmecorhynchus*, *Melophorus*, *Prolasius* and *Notoncus*, confirming the suspicions of the early Twentieth century myrmecologists such as Forel, Emery and Wheeler. Finally, Ward et al. (2016), in a molecular phylogenetic study, posited a tribe Melophorini that consists of eight purely Australian genera (*Melophorus*, *Myrmecorhynchus*, *Notoncus*, *Notostigma*, *Prolasius*, *Pseudonotoncus*, *Stigmacros* and *Teratomyrmex*) and one South American genus, *Lasiophanes*. Interestingly, this paper confirms much of Wheeler’s earlier (1935) analysis, especially when one considers that the uncommon and localised genus *Teratomyrmex* had not yet been discovered.

**Biology and ecology**. Although the genus *Melophorus* is generally poorly known, several species, most notably *Melophorus bagoti* Lubbock, have been the focus of concentrated research. Much of the current interest in *M. bagoti* concerns homing strategies in this ant (e.g., Wehner et al. 2006, Narendra 2007a, Narendra 2007b, Legge et al. 2010); while more occasional research has covered nest structure and activity (Conway 1992), thermophilism (Christian and Morton 1992) and general biology and taxonomy (Wheeler 1908). Other *Melophorus* species that have received individual attention have been *Melophorus perthensis* (as ‘*Melophorus turneri perthensis*’) (Majer et al. 2011), an unidentified species in the *Melophorus aeneovirens* group (Hoffman 1998) and those taxa that feature in taxonomic papers with some commentary on the habits of the species described (e.g., *Melophorus fulvihirtus*, in Clark 1941, and *Melophorus anderseni* and *M. majeri*, in Agosti 1997).

**Figure 1.**
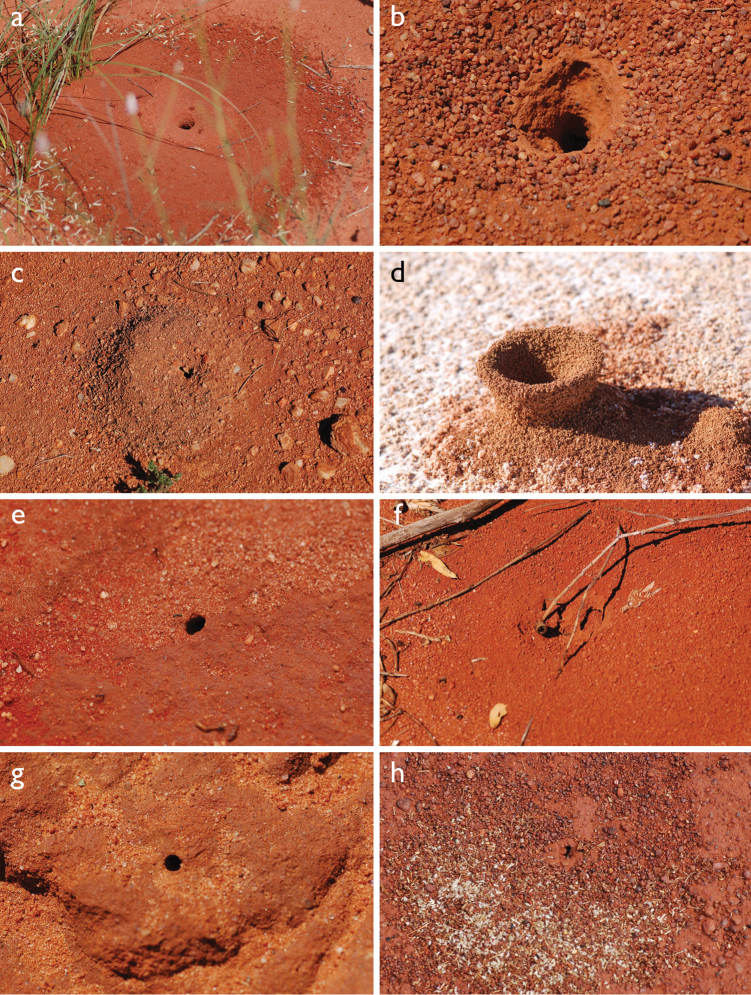
*Melophorus* nest entrances. **a** *M. bagoti* **b** *M. gracilipes* **c** *M. longipes* **d** M. *marmar* **e** *M. mjobergi* **f** *M. perthensis* **g** *M. sulla*, and **h** *M. wheeleri*.

In terms of general role in the environment, *Melophorus* ants are a highly thermophilic group that fills the same ecological niche as *Cataglyphis* in the Old world and *Myrmecocystus* in North America (Andersen 2007). All species are active only during the day, mostly at peak diurnal temperatures and predominantly in the summer months. Briese and Macauley (1980) found that one species (‘*Melophorus* sp. A’) studied at Emmet Vale, NSW, was only active when the temperature was between 37°C and 54°C, and this species was inactive for prolonged periods in the cooler months. Species are richest in arid or semi-arid environments, and relatively few inhabit thick forest or cooler, mesic areas. There are no alpine specialists. Typically, *Melophorus* species nest directly in soil (see Figure 1) and can rarely be found under stones or woody debris. Although they may forage on tree-trunks and vegetation no arboreal nesters are known. Soil nests of very common species such as *Melophorus turneri* and *M. perthensis* Wheeler are easily recognizable by the small, crescentic dunes of excavated sand that surround the nest hole (senior author pers. obs.). *Melophorus* nests are often or always closed with sand or pebbles at night (McAreavey 1947, current authors pers. obs.). Members of this genus are exceptionally timid ants, avoiding conflict with other ants (e.g., meat ants) by occupying a different temporal niche. They will also retreat into their nest upon the slightest human disturbance, and will not reemerge for several minutes (Shattuck 1999).

*Melophorus* ants have a diverse range of diets, depending on the species, and some are quite specialized. *Melophorus potteri* McAreavey is a termite raider (McAreavey 1947, Andersen 2000), and its bluntly serrate mandibles with a single large apical tooth may represent an adaptation to this lifeway. Interestingly, raids are carried out by individual workers, and not columns of workers, as in many formicid termitophages. A second species in the *M. potteri* group (*Melophorus pelecygnathus* Heterick et al. this work) has extraordinary hatchet-shaped, edentate mandibles and may have similar behavior. However, the third species in this group (*Melophorus macroschismus* Heterick et al. this work) has the normal 5-toothed dentition of most *Melophorus*. *Melophorus anderseni* Agosti and *Melophorus fulvihirtus* Clark raid meat ants’ (*Iridomyrmex purpureus* species-group) nests. Clark (1941) mentions the raiding activity of *M. fulvihirtus* in a brief note, but Agosti’s (1997) observations on *M. anderseni* are outlined in detail: this latter species uses subtle behavioural characters and the scent of its host so that it can carry off meat ant pupae. Workers of this ant were seen by Agosti to actually rub themselves against meat ant workers in order to obtain their nest odour.

The *M. wheeleri* species-group, in which the major workers have powerful, truncate, inwardly curved mandibles, are said to be obligate granivores (Andersen 2007). In some cases, exotic plants with suitable seeds may have replaced the use of native species in the diet of members of this species-group: for instance, in southern WA, *Melophorus wheeleri* Forel is known to feed on the seeds of the exotic grass *Avena barbata* (Harris and Standish 2008).

Apart from such specialized forms, most *Melophorus* are omnivores but include a large amount of vegetable material in their diet. Berg (1975) concluded that *Melophorus* species have a strong preference for elaiosomes, the nutrient rich appendages found on the seeds of many Australian plants. Elaiosome-eating species such as *M. perthensis* are likely to play an important role in native seed germination (Majer et al. 2011). *Melophorus perthensis* is one of the major seed harvesters in the West Australian jarrah forest (Majer 1982). Davidson and Morton (1981), who examined seed removal in inland NSW, found that diaspores (seeds modified for dispersal) moved by ‘*Melophorus* sp. H’ tended to be in the smaller range (» 4.09 mg). Animal protein in the case of omnivores may be supplied in the form of insects roasted in desert heat (Narendra 2007a) or dead ants discarded by their nestmates (Briese and Macauley 1981). Such scavenged Formicidae can constitute a surprisingly high percentage of food items: Briese and Macauley’s study includes tabular information that 40% of the food items taken back to their nest by an unnamed *Melophorus* species (‘*Melophorus* sp. A’) were of this nature. Several gracile species forage on the trunks of eucalypts. Greenslade (1979) states that one such species, with a peculiarly flattened head, is adapted to foraging under bark. *Melophorus* may attend lycaenid butterfly larvae that produce sugary glandular secretions, but known associations of lycaenid larvae with this genus of ants appear to be relatively minor and purely facultative (Eastwood and Fraser 1999, Fiedler 2001).

In turn, *Melophorus* species, especially their alates, are an important food source for some agamid lizards (e.g., they are one of the few sources of food for the Lake Eyre dragon lizard *Ctenophorus maculosus* Mitchell [Chan et al. 2009]), other ants (e.g., *Iridomyrmex*) and spiders (a zodariid spider is mentioned as an important predator of *Melophorus bagoti* by Muser et al. 2005). On the other hand, non-agamid lizards prefer termites over ants (Abensperg-Traun and Steven 1997). Information on *Melophorus* as a dietary item for other Australian vertebrates is sketchy at best and usually lacking. *Melophorus* species are rare in the diet of numbats (Calaby 1960), and there is little or no specific information on genera of ants, other than *Iridomyrmex*, included in the food intake of other ant-eating mammals such as echidnas and several smaller carnivorous marsupials that will eat ants. Insectivorous snakes may take *Melophorus*, although there is just the one record of *Melophorus* as a prey item (for *Ramatyphlops australis*) in the list of ant genera eaten by blind snakes (Typhlopidae) supplied by Webb and Shine (1993). *Melophorus* alates, presumably, also run the risk of predation by birds. Worker ants, however, are probably not taken by most birds because of the uncomfortably high temperatures at which they are active and their speed over the ground surface. Replete workers of *Melophorus bagoti* are famous as food for desert dwelling Aboriginal people, but some references to their use are qualified (e.g., Conway 1992) or reject that repletes of *M. bagoti* are acceptable as human fare (thus, Australian Myrmecology Gallery 2002).

**Homing strategies**. *Melophorus bagoti* (and, in all likelihood, other desert-dwelling species of *Melophorus*) uses a type of homing device called path-integration that enables ants to return to their nest along the shortest return route in a featureless environment (Narendra 2007a). Presumably, this type of orienting also compensates for the difficulty in following pheromone trials laid on shifting or unstable, windblown soils. Ants may also be blown off their paths by dust storms (Narendra 2007b). In a carefully manipulated experiment, Legge et al. (2010) pursued this question further. Where path integration information was not available, *M. bagoti* workers did not use visual landmarks (artificially provided in the study). Instead, they oriented in a particular direction (in this case the exit from an artificial arena) regardless of whether or not it was aligned with the ants’ nest. Thus, it appears that vectors used by the ant to return to its nest are based on compass information gleaned from previous trips, i.e., the path integrator does not process information based on the current journey. The nature of the landmarks that enable these ants to learn local vectors in natural circumstances have not been determined as yet, but Legge et al. surmise that possibly the skyline is a factor, as is polarized light.

**Figure 2.**
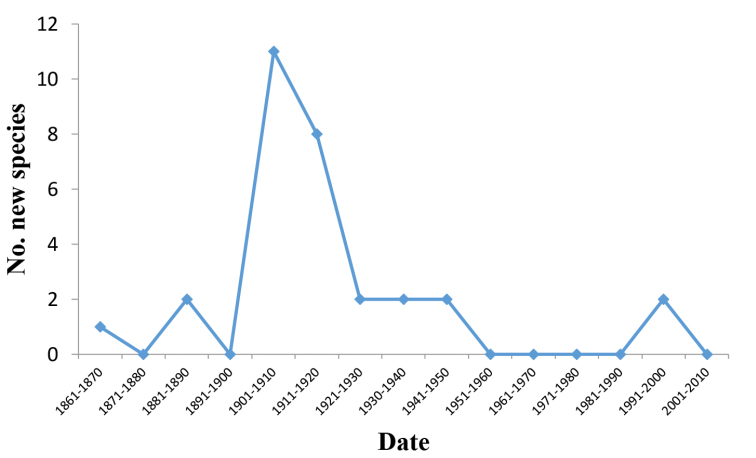
Numbers by decade of new species of *Melophorus* appearing in the taxonomic literature since 1865 (when *Melophorus aeneovirens* was described).

**The current position of *Melophorus*.** This genus is currently very poorly characterized considering its importance in the Australian landscape. At the time of writing there are only 24 full species and eight subspecies in the taxonomic literature. One taxon (*Melophorus cowlei* Froggatt, here reinstated as a junior synonym of *Melophorus bagoti*) was misidentified for quite a large portion of its history as a *Camponotus*. The lack of taxonomic activity, especially in regards to the smaller species, is quite puzzling. Synonymy is extensive, and clusters around a small handful of the larger and more ubiquitous taxa (namely, *M. aeneovirens*, *M. biroi* Forel, M. *turneri* and *M. wheeleri*). Much of the descriptive work took place in the era 1900-1930 (see Figure 2), which corresponds to the most productive period of the great continental taxonomist-systematist Auguste Forel, who described almost fifty per cent of the *Melophorus* fauna prior to this monograph. After 1930 the description of new *Melophorus* species has largely been the province of the amateur researcher Father John J. McAreavey and the economic entomologist John Clark.

**Biomonitoring Potential**. In Australia, *Melophorus* ants are currently an important component in the Ant Functional Group (e.g., Andersen 1990, 1995) mix used by researchers and land managers to investigate the success of minesite, rangeland and burnt area rehabilitation. However, their thermophilism also makes them a potential candidate in investigations into microhabitat alteration as climate and weather patterns change under the influence of global warming. Hypothetically, the natural range of many species may be expected to be extended with increasing aridity and higher average temperatures, particularly in the south west of the Australian continent.

*Melophorus* is deserving of closer scientific scrutiny for a variety of reasons. Not only is it one of the largest Australian ant genera, being more speciose and biologically diverse than genera like *Iridomyrmex* and *Monomorium*, but its importance in the fabric of many Australian ecosystems and its biomonitoring potential may be even greater than the abovementioned groups. Unrelated areas of research involving *Melophorus* ants, such as the analysis of thermophilic activity, orientation using path-integration and the dispersal of elaiosome-producing seeds, have already become well-established, and members of this genus undoubtedly have other adaptations that could be identified and studied with profit.

As a final note, in the field most members of this genus of ants are extremely difficult to catch because of their rapid movement across the ground. The senior author found that major workers and minor workers of large species could be slowed without damage by dabbing gently at them with cotton wool impregnated with ethanol, and then picking the ants off the cotton wool with forceps. Minor workers were collected by suctioning – mainly at the entrance to their nest – with a pooter and transferring them immediately to an ice bath (i.e., a basin filled with ice and water). This had the effect of paralyzing these thermophilic organisms so that they lay quiescent on the water surface and could be transferred with ease to a vial of ethanol.

## Phylogeny: materials and methods

The procedure followed here is that results of the molecular work will be presented first, culminating in a reconstructed evolutionary tree, and then will follow the morphological taxonomic results and discussion.

### Sources of material and images

The following Museums and research institutions provided loans or images or otherwise assisted with making material available for this study:

**AMS** Australian Museum, Sydney, Australia.

**ANIC** Australian National Insect Collection, Canberra, ACT, Australia.

**BMNH** The Natural History Museum, London, UK.

**CASC** California Academy of Sciences, San Francisco, California, USA.

**HNHM** Hungarian Natural History Museum, Budapest, Hungary.

**JDM** Curtin Ant Collection, Curtin University of Technology, Perth, Western Australia, Australia (now amalgamated under WAM–see below).

**MCZ** Museum of Comparative Zoology, Harvard University, Cambridge, Massachusetts, USA.

**MHNG** Muséum d’Histoire Naturelle, Geneva, Switzerland.

**MSNG** Museo Civico di Storia Naturale ’Giacomo Doria’, Genoa, Italy.

**NHMB** Naturhistorisches Museum, Basle, Switzerland.

**QM** Queensland Museum, Brisbane, Queensland, Australia

**SAMA** South Australian Museum, Adelaide, South Australia, Australia.

**SMNH** Naturhistoriska riksmuseet, Stockholm, Sweden

**WAM** Western Australian Museum, Perth, Western Australia, Australia.

**ZHMB** Museum für Naturkunde der Humboldt Universität, Berlin, Germany.

### Abbreviations of depositories

Novel type material (holotypes and paratypes) was deposited in the following institutions: **ANIC, BMNH, MCZ, QM** and **SAMA**. Note: Although it did not provide material or images, reference is also made in this monograph to The Tropical Ecosystems Research Centre (TERC), centred in Darwin, Northern Territory, Australia.

### Measurements and indices (Figure 3)

Major and minor workers of each species have been included in the measurements where possible. However, for some uncommon species only minor or major workers are known. Size and shape characters were quantified and are reported as lengths or indices. Measurements were made with a stereomicroscope using a dual-axis stage micrometer wired to digital readouts. All measurements were recorded in thousandths of a millimetre, but expressed here to the nearest hundredth. The following measurements and indices are reported (readings are presented in mm).

**EL** Eye length: maximum length of the eye measured in profile

**HL** Head length: maximum head length in full-face (dorsal) view, measured from the anterior-most point of the clypeal margin to the midpoint of a line drawn across the posterior margin of the head.

**HW** Head width: maximum head width measured in full-face (dorsal) view, excluding the eyes.

**ML** Mesosomal length: mesosomal length measured from the anterior surface of the pronotum proper (excluding the collar) to the posterior extension of the propodeal lobes.

**MTL** Maximum tibial length: maximum length of the tibia of the middle leg, excluding the proximal part of the articulation which is received into the distal end of the femur.

**PpH** Propodeal height: maximum height of propodeum measured tangentially from a line drawn between the metanotal groove to the posterior extension of the propodeal lobes.

**PpL** Propodeal length: length of propodeum measured from the metanotal groove to the posterior extension of the propodeal lobes.

**SL** Scape length: scape (first antennal segment) length excluding the basal neck and condyle.

**CI** Cephalic index: head width (HW)/head length (HL) × 100.

**EI** Eye index: eye length (EL)/head width (HW) × 100.

**SI** Scape index: scape index: SL/HW × 100.

**Figure 3.**
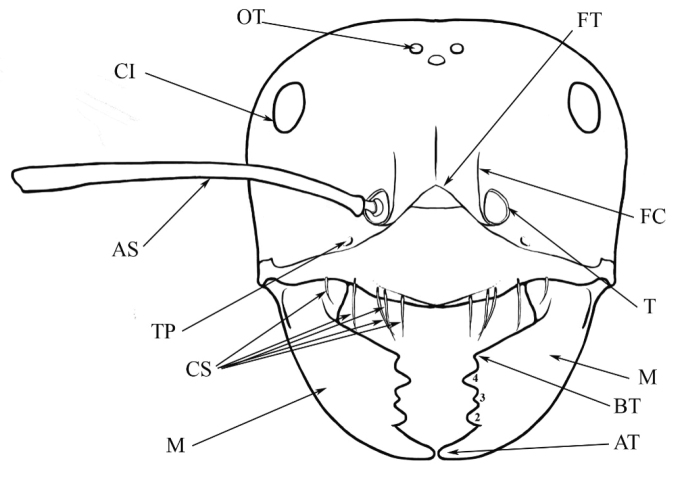
Frons of head of *Melophorus* showing structures mentioned in this monograph: **AS** antennal scape **AT** apical tooth **BT** basal tooth **CI** compound eye **CS** clypeal psammophor**e, c**onsisting of a fringe of setae (nb. only those on RHS of head labelled) **FC** frontal carina **FT** frontal triangle **M** mandible **OT** ocellar triangl**e, c**onsisting of three ocelli (simple eyes) **T** torulus **TP** tentorial pit; 2, 3, 4, tooth sequence commencing from the apical tooth.

### Molecular phylogenetics

**Tissue collection**. Specimens were collected by hand and placed into 100% ethanol.

**DNA extraction.** A non-destructive DNA extraction method, ANDE (Castalanelli et al. 2010) was used to extract DNA from all the specimens used in this study.

**Amplification and Sanger sequencing.** For specimens M001-M154 and M303-M356, amplification of the five genes (COI, LR, Wg, AA, and H3) was performed using the primers outlined in Table 1. PCR amplification volumes were 25 µl, including 2.5 µl of ANDE extracted DNA (1:10 dilution DNA:water) which was the equivalent of 20 to 25 ng/µl. The reaction mix comprised of 1 × polymerase buffer (Bioline; MyTaq), 0.2 µM of each primer. The thermocycler conditions were: 95 °C for 10 min; 40 cycles of: 95 °C for 30 s, annealing for 30 s, and 72 °C for extension, with a single final extension.

Quality and quantity of the amplified PCR products were determined on a 1.5% agarose gel. Sequencing of the amplified genes was carried out by AGRF (Australian Genomic Research Facility) using an Applied Biosystems ABI 3730 48-capillary DNA analyser with Big Dye Terminator Technology according to the manufacturer’s protocols (Applied Biosystems).

**Table 1.** Primers used during this study.

**Gene**	**Primer Name**		
mT COI (COI)	LCO1490	5'-GGT CAA CAA ATC ATA AAG ATA TTG G-3'	Folmer et al. 1994
	HCO2198	5'-TAA ACT TCA GGG TGA CCA AAA AAT CA-3'	Folmer et al. 1994
	Jerry	5'-CAA CAT TTA TTT TGA TTT TTT GG-3'	Simon et al. 1994
	CI-J-1718	5'-GGA GGA TTT GGA AAT TGA TTA GTT CC-3'	Simon et al. 1994
nDNA Wingless (Wg)	Wg578F	5'-TGC CAN GTG AAR ACY TGC TGG ATG CG-3'	Ward and Downie 2005
	Wg1032R	5'-ACY TCG CAG CAC CAR TGG AA -3'	Abouheif and Wray 2002
nDNA Abdominal-A (AA)	ant-M	5'-CGG CAC CGG CGA TAT GAG TAC GAA ATT C-3'	De Menten et al. 2003
	ant-J	5'-GGG TTG TTG GCA GGA TGT CAA AGG ATG-3'	De Menten et al. 2003
nDNA Rhodopsin (LR)	LR143F	5'-GAC AAA GTK CCA CCR GAR ATG CT-3'	Ward and Downie 2005
	LR639ER	5'-YTT ACC GRT TCC ATC CRA ACA-3'	Ward and Downie 2005
nDNA Histone 3 (H3)	H3F	5'-ATG GCT CGT ACCAAG CAG AC(ACG) GC-3'	Colgan et al. 1998
	H3R	5'-ATA TCC TT(AG) GGC AT(AG) AT(AG) GTG AC-3'	Colgan et al. 1998

**Amplification and amplicon sequencing.** For specimens M155-302, amplification of the five genes gene was performed using the primer outlined in Table 1, with the additional M13 sequences 5'-GTAAAACGACGGCCAGT-'3 and 5'-AACAGCTATGACCATG-'3, added to the 5' end of the forward and reverse sequences, respectively. PCR volume was 25 µl, including 2.5 µl of ANDE extracted DNA (1:10 dilution DNA:water) which was the equivalent of 20 to 25 ng/µl. The reaction mix comprised of 1 × polymerase buffer (Bioline; MyTaq) and 0.2 µM of each primer. The thermocycler conditions were: 95 °C for 10 min, 40 cycles of: 95 °C for 30 seconds, annealing for 30 seconds, and 72 °C for extension; with a single final extension of 72 °C for 30 s for 5 minutes.

To prepare the amplicons for next generation sequencing a second round of PCR amplification was performed. The primers consisted of the Roche Lib-A adapters (Roche), a unique multiplex identifier (MID) sequence, and the complementary M13 sequence. In total 96 unique primer sets were constructed so that batches of 96 samples could be pooled and analysed at once. PCR volume was 25 µl, including 2.5 µl of first round amplifcation extracted DNA (1:100 dilution DNA:water). The reaction mix comprised 1 × polymerase buffer (Bioline; MyTaq) and 0.2 µM of each primer. The thermocycler conditions were: 95 °C for 10 min; 10 cycles of: 95 °C for 30 seconds, annealing for 30 seconds, and 72 °C for extension; with a single final extension.

Quality and quantity of the amplified PCR products were determined on a Labchip GXII (Perkin Elmer) using the HT 1K LabChip (Perkin Elmer). For each gene, the successfully amplified PCR products were normalised to 1ng/uL and pooled. Each gene pool was purified using the E-Gel® SizeSelect™ Gels (Thermofisher), then further purified using Agencourt AMPure XP (Beckman Coulter) as per the manufacturers’ instructions. The quantity of each gene was determined on a Labchip GXII (Perkin Elmer) using the HT 1K LabChip (Perkin Elmer) and each gene mixed to an equimolar concentration, term library. The library was diluted 1 in 1,000 and further purified twice using Agencourt AMPure XP as per the manufacturers’ instructions. The library was quantified using quantitative PCR as per Bunce et al. (2012) and diluted to approximately 2 million copies. The GS Junior sequencing run was set up as per the manufacturers’ instructions, and run for 200 flows.

**Data analysis.** The data were exported in fasta format and analysed using a self-written bioinformatics script based in Python (Python Software Foundation). Each sequence was analysed for its MID and primer sequence, which allowed the sequence to be assigned back to its specimen identification number and gene with all associated sequences saved in a single fasta file. For each specimen, the fasta file for each gene was imported in Geneious research software (BioMatters) and assembled and checked for sequencing errors. Low-occurring single nucleotide polymorphisms (SNP) were ignored, but where an SNP was >30% an IUPAC (International Union of Pure and Applied Chemistry) code was used. Each gene was translated using the standard invertebrate mitochondrial and nuclear codes, where applicable, to detect the presence of nuclear mitochondrial DNA (NUMTs).

**Phylogenetic analysis.** Phylogenetic relationships among each of the complexes and species were assessed using *BEAST (Drummond et al. 2012). To test the complexes proposed here, individuals were grouped according to their complex and similar species were grouped together according to their morphological identification. Each of the five genes was systematically tested using the substitution models General Time Reversible (GTR), Hasegawa-Kishino-Yano (HKY), Jukes-Cantor (JC) and clock models (strict and lognormal). Due to the high divergences within the COI gene, this data set was split and the first and second codon bases were examined with and without the third base. Each of these combinations were evaluated with gen=10,000,000, log=1000. Evolutionarily Stable Strategy (ESS) scores were evaluated for each run to determine which model was the best for each gene. The final tree models were HKY for the genes AA, Wg, and H3, and GTR for COI and LR. Strict clock rates were run for all genes for 50,000,000 generations and logged every 5,000 generations.

### Evolutionary history

BEAST (Drummond and Rambaut, 2007) was used to estimate temporal distance between target taxa and their most recent common ancestor (Time to Most Recent Common Ancestor, or TMRCA). This program is suitable for evaluating rooted, time-based phylogenies using strict or relaxed molecular clocks; it is also useful as a framework for testing evolutionary hypotheses by focusing on multiple tree topologies, rather than merely a single tree. BEAST uses the Markov Chain Monte Carlo (MCMC) algorithm sampling method to average across tree space (i.e., the dimension that includes all possible trees), so that each tree is weighted proportionally to its posterior probability. In our case, only four out of the five genes were selected due to the availability of other Formicinae sequences deposited on genbank (NCBI). The evolutionary clock model Generalised Time Reversible (GTR) was applied to the LR and COI genes. In contrast, the Hasegawa, Kishino and Kano (HKY) model was applied to the AA and Wg genes. These models were selected as they each provided optimal ESS scores. No *Melophorus* fossils are known (http://antwiki.org/wiki/Checklist_of_Melophorus_species), so were not available for this exercise. The first node, including the root height parameters, was offset to 145 million years ago (Ma ago) with an exponential prior distribution and a mean of 10 Ma, which returned quantiles of 145.5 and 175.0 Ma ago, i.e., 5% and 95% respectively. The lower boundary represented the date calculated by Brady et al. (2006), while the upper boundary corresponded to the MRCA boundary for ants calculated by Moreau et al. (2006). The second node calibrated the MRCA for the Melophorini group, which was set with an exponential prior distribution offset to 52.6 Ma ago (the lowest date calculated by Blaimer et al. (2015) and mean 7 Ma, which returned an upper quantile of 78.42 Ma ago. This upper bound coincides with the vicariance event of Australia splitting from New Zealand and New Caledonia (80–60 Ma ago) (Stevens 1989, Yan and Kroenke 1993). These bounds are also supported by the placement of the South America genus *Lasiophanes* within Melophorini (Ward et al. 2016).

## Results

### Mitochondrial and nuclear sequence data

Forty-six taxa from the *M. aeneovirens*, *M. biroi*, *M. ludius* and *M. potteri* species-groups produced useable sequences (Table 2). No representatives of the *M. anderseni* or *M. majeri* species-groups were available for analysis, and a single representative of the *M. fulvihirtus* species-group failed to produce a useable sequence The number of samples amplified for each gene ranged from 116 (AA) to 141 (Wg). In total, 83 individuals from 33 species were successfully sequenced for all five genes, and these constitute most of the species discussed in the section dealing with molecular analysis.

Within the genus *Melophorus* the COI gene fragment was the most divergent with a maximum interspecific divergence of 17.3% (Table 3.). Similarly, the intraspecific divergence was four time higher than that for the nuclear genes. Several species had COI intraspecific divergences >5%; these included *M. bagoti*, *M. biroi*, *M. perthensis*, *M. rufoniger*, *M. sulla*, *M. turneri*, and *M. wheeleri*. As expected, the nuclear genes were considerably more conserved than the mitochondrial gene and ranged from an average intraspecific distance of 0.87% to 1.89% for AA and H3, respectively. The minimal divergences within the nuclear genes are reflected in the lack of clarity they bring when included in the species trees.

**Table 2.** Taxonomic groups and the number of gene sequences generated.

Group	Complex	Species	AA	COI	H3	LR	Wg
***aeneovirens***	*aeneovirens*	*canus*	1	1		1	1
		*griseus*	1	1		1	1
		*platyceps*		1		1	1
		*praesens*	1	1	1	1	1
		*rufoniger*	3	3	1	3	3
		*sulconotus*	1	1		1	1
		*teretinotus*					1
	*bagoti*	*bagoti*	7	7	8	9	9
		*gracilipes*	3	4		4	4
	*nemophilus*	*nemophilus*	1	1	1	1	1
***biroi***	*biroi*	*biroi*	6	8	7	8	8
		*graciliceps*	2	3		3	2
		*gracilis*	2	3	4	3	4
		*lissotriches*	1	1	1		1
		*mjobergi*	7	7	7	7	7
		*postlei*	1	2	2	2	2
	*brevignathus*	*marmar*	2	2	2	2	2
	*fieldi*	*ankylochaetes*	2	2	2	2	2
		*bruneus*		1	1	1	1
		*eumorphus*	1	1	1	1	1
		*fieldi*		1	1	1	1
		*fulvidus*	1	1	1	1	1
		*hirsutipes*	3	5	5	5	5
		*incisus*	2	2	2	2	2
		*inconspicuus*	1	1	1	1	1
		*lanuginosus*	6	4	6	6	6
		*longipes*	4	5	5	5	5
		*microtriches*	5	4	5	5	5
		*perthensis*	5	6	5	6	6
		*sulla*	6	5	5	6	6
		*turneri*	15	14	18	18	18
		*vitreus*	1	1	1	1	1
	*wheeleri*	*chauliodon*	1	4	4	4	4
		*caeruleoviolaceus*	1	1	1	1	1
		*laticeps*	1	1	1	1	1
		*parvimolaris*	1	1	1	1	1
		*pelorocephalus*	2	2	2	2	1
		*purpureus*	1	1	1	1	1
		*wheeleri*	11	10	12	11	14
		*xouthos*	1	1	1	1	1
	*oblongiceps*	*oblongiceps*	1		1	1	1
***ludius***	*hirsutus*	*hirsutus*	1	1		1	1
	*ludius*	*ludius*		1	1		1
		*pusillus*	1	1	1	1	1
		*translucens*	1	2	2	2	1
***potteri***	*potteri*	*potteri*	2	1	2	2	2
**Total**			116	126	123	137	141

**Table 3.** Maximum pairwise sequence divergence within species, for the five genes in this study. Blank cells indicate that only one specimen was sequenced for that gene-species combination.

Species	AA	COI	H3	LR	Wg
*ankylochaetes*	0	0.9	0	0.2	1
*bagoti*	0.9	8.3	11	2.5	1.1
*biroi*	1.7	10.8	9.2	1.4	2.5
*chauliodon*		2.5	1	2.7	1.3
*gracilis*	1.1	0.9	0	1.4	1.5
*hirsutipes*	0.3	7	1.9	0.5	2.2
*incisus*	0.9	0	0.7	0	0
*lanuginosus*	1.3	4.7	1.3	2.2	0.8
*marmar*	0	0	0.3	0.9	0.8
*microtriches*	0.3	1.2	0.5	0.4	0.5
*mjobergi*	0.8	1.4	3	0.8	2.4
*pelorocephalus*	0	0	0.7	0	
*perthensis*	0.3	5.5	0.7	1.3	1.6
*postlei*		1.2	0.7	0.3	0.4
*potteri*		0	0	0	0
*rufoniger*	1.3	10		1.1	2.4
*sulla*	1	8.9	0.7	2.3	1.4
*translucens*		0	0	0.2	
*turneri*	3.3	11.8	2.3	2.5	2.8
*wheeleri*	0.8	7.8	2.3	2.2	2.8
**Average Intraspecific**	**0.87**	**4.15**	**1.9**	**1.2**	**1.4**
**Maximum Interspecific**	**4.1**	**17.3**	**12**	**7.1**	**6.5**

* Blank cells indicate that only a single gene was sequenced and no value was calculated

### Phylogenetic analysis and discussion

**Figure 4.**
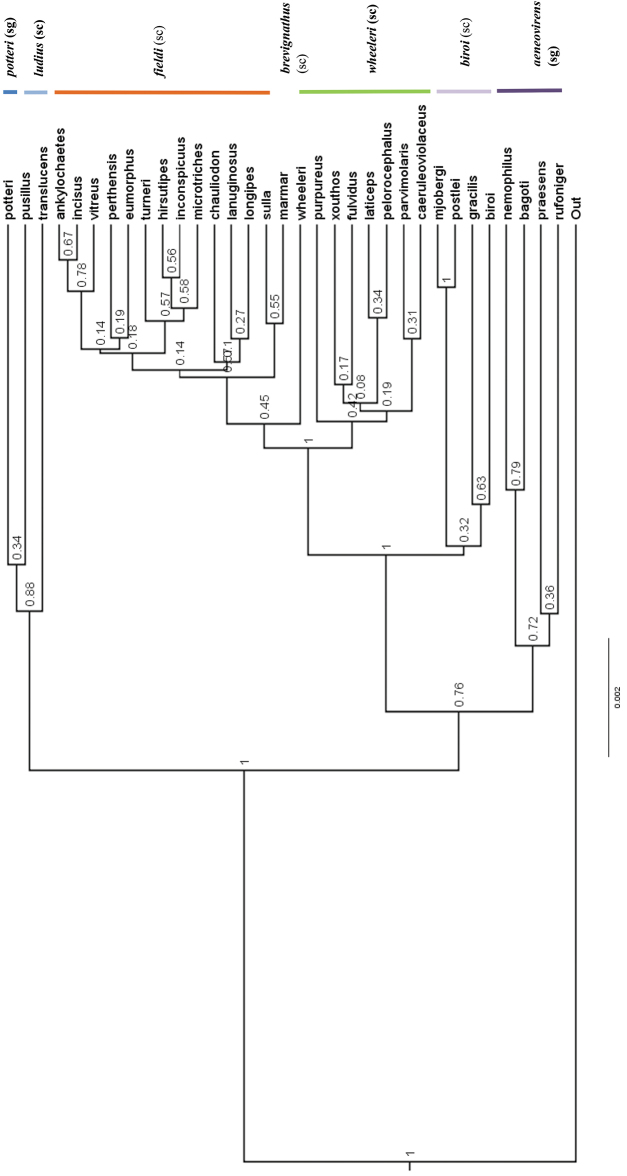
Phylogenetic species tree for five genes. Species-groups and complexes are colour-coded. Outgroups include *Notoncus gilberti*, *Nylanderia glabrior*, *Leptanilla* sp., *Strumigenys pulchella*, and *Aenictus* sp. Each vertical color-bar links taxa of the same species-group or species-complex. Numbers above lines represent branch support; the scale bar refers to genetic change of 0.002 nucleotide substitutions (‘sc’ = species-complex; ‘sg’ = species-group).

Overall, the results of the species-level tree (Figure 4) are concordant with the morphology. The species tree reveals well supported clades at the complex level with the most prominent exception being *M. chauliodon* (which aligns with the *M. fieldi* complex based on the genetics, although the mandibular morphology of the major worker would seem to place it within the *M. wheeleri* complex). However, a three-gene species tree (Suppl. material 1) constructed using the LR, Wg and COI genes strongly places *M. chauliodon* within the *M. wheeleri* complex. In fact, our conclusions based on the morphological appearance of the taxa is that the three-gene tree appears to corroborate some of the relationships suggested by the morphology more than does the five-gene tree. This may be due to confusion arising from uninformative signals from the histone and Abdominal-A genes in the latter. Thus, in the three-gene tree, *M. fulvidus* and *M. chauliodon* (as mentioned above) are placed near to taxa with which they share anatomically significant structures. In the five-gene tree they appear out of place. Indeed, most species anatomically associated with the *M. wheeleri* complex and the *M. fieldi* complex also show corresponding associations within the three-gene tree. The placement of *M. xouthos* and *M. parvimolaris* in the *M. wheeleri* complex, however, is supported by the five-gene tree but not the three-gene tree.

A significant exception to the general concordance of morphology and genetics is the position of the taxa *Melophorus hirsutus*, *M. pusillus* and *M. translucens* on both three-gene and five-gene species trees. The clade including these three ants exhibits an unexpectedly basal relationship to indisputable members of the *M. biroi* species-group. This is counterintuitive, as their morphology would support a close relationship between these taxa and those within the *M. mjobergi*-*biroi* clade, let alone the broader species-group. However tempting it might be to suggest that sequencing issues were responsible for this unexpected result, the algorithm used produced the same disconcerting result for individual gene trees as well as multiple gene trees, so that it cannot readily be thought of as artificial (but see further discussion under Evolutionary history discussion).

The sister-species relationships are in contrast to the generally strongly supported broader evolutionary patterns; for example, the relationships within the *M. fieldi* and *M. wheeleri* complexes are only weakly supported. The lack of support reflects the choice of genes: the mitochondrial gene was strongly divergent with high levels of homoplasy at the third codon base. When analysis was conducted with the third base incorporated, several species became polyphyletic and the ESS scores dropped below 200. However, the removal of the third base dramatically increased the ESS scores and rendered almost all species monophyletic, apart from *M. biroi*, *M. turneri*, *M. sulla*, and *M. hirsutipes*.

Given that the removal of the third codon base still left consistent paraphyly or even polyphyly for *M. turneri*, *M. sulla* and *M. hirsutipes*, other explanations for this phenomenon are required. The most obvious one is incorrect identification. (We confess here that the inaccurate identification of these sometimes maddeningly similar little ants accounted for many early sequencing discrepancies before good diagnostic characters could be discovered!) However, even after careful checking and rechecking, the three taxa mentioned continued to pose problems requiring a different explanation. Firstly, the splitting could be indicative of sibling or cryptic species not identified by morphological analysis. The high pairwise divergence among the three species (> 5%) might suggest this explanation. This is less likely to be the case with *M. hirsutipes* in which the degree of genetic divergence is less pronounced (7.8% compared with 11.8% in *M. turneri* and 8.9% in *M. sulla*), although there is a greater degree of physical variability among *M. hirsutipes* workers than there is in the other two taxa. In the case of *M. hirsutipes* also, the three-gene tree showed results (e.g., the placement on the tree of samples M153 and M323) that were disparate compared with the results of other trees. In the three-gene tree (strongly influenced by the COI configuration), M153 and M323 are on different branches, but they are sisters in the Wg tree. Similar results occurred with *M. turneri*. This suggests possible hybridisation. The senior author found likely hybrids of *M. turneri* and *M. bruneus* north of Wiluna, and incomplete speciation or, at least, relatively high gene flow between more-or-less discrete species, may also account for the polyphyly seen. Thirdly, since genotype does not automatically match phenotype, the trees could be illustrating incomplete lineage assortment, with more than one allele found in the genes used for a given species represented in the sequencing. Since these three species are all very common ants that occur across a wide range of ecosystems throughout Australia, incomplete lineage assortment is a possibility that should also be investigated.

Possibly one or a combination of these three interpretations (and there are other possibilities) could account for the differences seen on the trees for these three similar but morphologically discrete species. Perhaps the greatest likelihood, given their strongly derived position in the three-gene tree, is that the incidence of multiple alleles through frequent mutations in the mitochondrial gene COI due to rapid evolution is very high in these taxa. The field evidence of possible hybridization in this *Melophorus* complex may also indicate the fixation of the speciation process is not yet complete for *M. hirsutipes*, *M. sulla* and *M. turneri* (this could also be the explanation for separation of samples for the same taxon in the slower evolving Wg tree).

**Figure 5.**
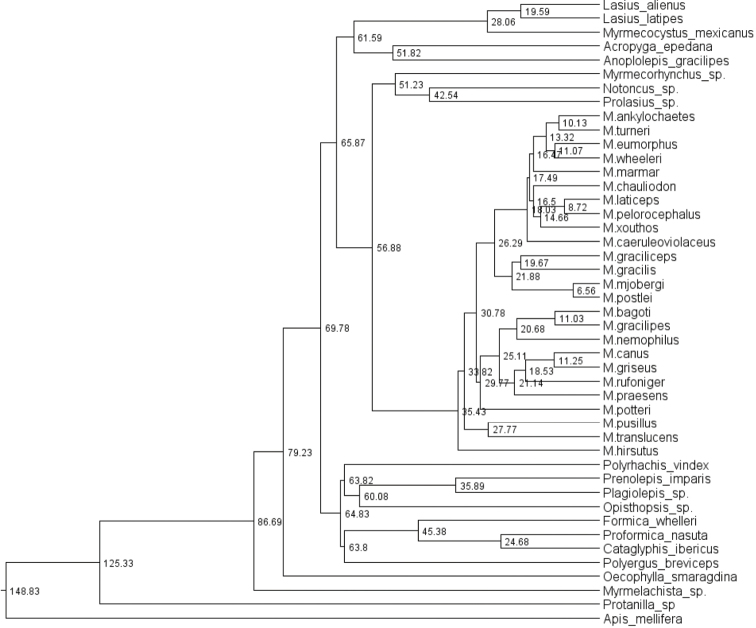
Calculated dates of divergence for TMRCA for *Melophorus* and other sample formicine taxa, including several of the postulated nearest relatives of *Melophorus* (i.e., other Melophorini). Outgroups are *Apis mellifera* and *Protanilla* sp.

The issues surrounding *M. biroi* (which occupies a much more basal position on the five-gene tree) are more substantial, and this taxon is likely a complex of two or more species (displayed in Figure 5). This matter is discussed further under the individual species accounts.

### Evolutionary history discussion

The phylogenetic history of Formicidae and, to a lesser extent, Formicinae, has been discussed extensively by Moreau et al. (2006) and Brady et al. (2006). These two groups of authors suggest that TMRCA of formicoids/poneroids and leptanillines lived between 116 (Brady et al. 2006) and 156 (Moreau et al. 2006) Ma ago, with the advent of the Formicinae ranging from 77 (Brady et al. [2006]) to 92 (Moreau et al. [2006]) Ma ago. More recently, Blaimer et al. (2015) has provided further insight into the Formicinae and its constituent tribes. Our chief aim in this work was to estimate the divergence dates for the species-groups within *Melophorus* (Table 4).

**Table 4.** Summary of divergence dates (Ma ago) here proposed for select crown taxa including *Melophorus* species-groups and complexes.

Subfamily	Ma ago
Leptanillinae	149,56
Formicinae	103,65
**Tribe**	
Melophorini	57,03
**Genus**	
*Melophorus*	35,43
**Species-Group**	
*aeneovirens*	21,14
**Species complex**	
*bagoti*	11,03
*biroi*	21,88
*ludius*	27,77
*fieldi*	13,32
*wheeleri*	16,42

Based on the estimated ages of this study, we propose that the Formicinae arose significantly earlier than the 77 Ma ago estimated by Brady et al. (2006), and closer to the dates estimated by Moreau et al. (2006) and Blaimer et al. (2015). Furthermore, formicine fossil records suggest a MRCA exceeding 92 Ma ago (Grimaldi and Agosti 2000), which is within the parameters of our data. The MRCA of 57.03 Ma ago for Melophorini (congruent with Blaimer et al. [2015]) coincides with the vicariance events that led to the separation of Australia from New Zealand and New Caledonia (80-60 Ma ago [Stevens 1989, Yan and Kroenke 1993]). The inclusion of the genus *Lasiophanes* by Ward et al. (2016), which is endemic to South America, also supports this date. For organisms other than ants, the link between South America and Australia flora and fauna and New Zealand has been well documented for this period (e.g., mostly extinct lineages of southern beech (*Nothofagus*) (Cook and Crisp 2005), marsupials (Mitchell et. al 2014, Nilsson et al. 2004, Nilsson et al. 2010), and Diptera (Yeates and Wiegmann 2005). The other Melophorini are almost entirely restricted to Australia. The sole exceptions are *Prolasius advena*, which is endemic to New Zealand but has Australian affinities (http://www.landcareresearch.co.nz/publications/factsheets/Factsheets/prolasius-advenus), and *Notoncus gilberti*, which is widespread on the mesic east and south-west coasts of Australia. This species has also been recorded from New Guinea (http://www.antwiki.org/wiki/Notoncus_gilberti) but could conceivably have been introduced by human agency. Within the Melophorini the origins of *Melophorus* were estimated to be ~35Ma ago, again congruent with Blaimer et al. (2015).

Within the *Melophorus* genus, *M. hirsutus* shares a MRCA with the remainder of the species sampled that is estimated at ~35 Ma ago. Not only does this ant have several unique attributes (i.e., the virtual absence of a metanotal groove, a translucent lamina surrounding the pronotum, and distinctive conical or sub-conical eyes and protrusive antennal sclerite [this latter seen only in one population of another, unrelated *Melophorus* species]), it is phylogenetically basal and remains one of the few *Melophorus* to favour a mesic environment. Within Australia, this species is confined to the wet east coast and is not found much west of the Great Dividing Range. During the period in which *Melophorus hirsutus* appears to have arisen, Australia was experiencing moist climatic conditions but started to dry out during the middle of the Miocene (23-5 Ma ago). Temperatures became more elevated in the period 21-14 Ma ago. After 14 Ma ago, temperatures dropped sharply. Acidification of waterways in central Australia continued to increase and river systems reduced their output and became salt lakes (White 1994).

**Figure 6.**
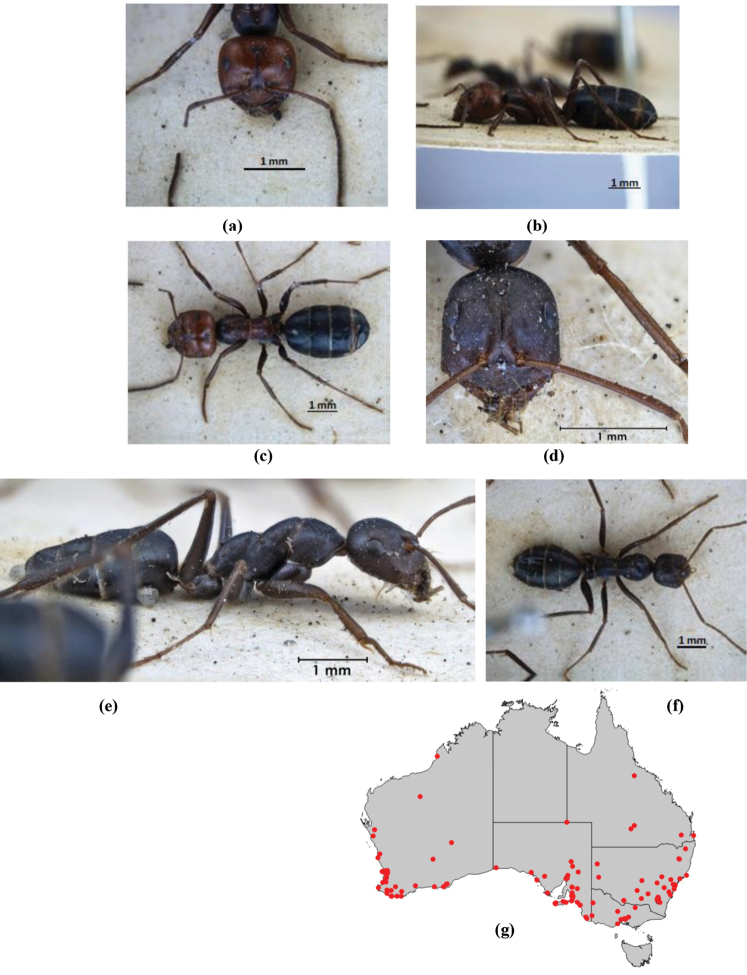
*Melophorus aeneovirens* (Lowne): BMNH major worker syntype frons (**a**), profile (**b**) and dorsum (**c**); BMNH minor worker syntype frons (**d**), profile (**e**) and dorsum (**f**); distribution map for the species (**g**).

Based on our molecular findings, this coincides with the period in which rapid diversification within the *M. fieldi* complex occurred (Figure 6). The *M. fieldi* complex is the most speciose group within *Melophorus*. These findings corroborate other studies that support the hypothesis that the aridification of Australia has led to rapid diversification of plants, marsupials, and various reptile groups (see table 1 in Byrne et al. 2008).

## Classification and taxonomy: results

### Genus Melophorus

Taxon classificationAnimaliaHymenopteraFormicidae

Lubbock, 1883

Melophorus Lubbock 1883: 51. Type-species: Melophorus bagoti, by monotypy.
Erimelophorus Wheeler 1935: 71. (described as a subgenus of Melophorus; junior synonym of Melophorus by Brown 1955: 474). Type-species: Melophorus wheeleri, by original designation.
Trichomelophorus Wheeler 1935: 71. (described as a subgenus of Melophorus; junior synonym of Melophorus by Brown 1955: 474). Type-species: Melophorus hirsutus, by original designation.

#### Worker diagnosis.

Polymorphic ants characterized by a combination of long, J-shaped setae on the mentum, an elongate (often slit- or comma-shaped) propodeal spiracle, presence of a metapleural gland, antennal insertions abutting the posterior clypeal margin and three ocelli in an ocular triangle in all workers. The genus is taxonomically compact, and with the above characters taken into consideration, its members cannot be mistaken for any other group of ants. The descriptions below relate to the small minor worker and the large major worker. Media workers are of intermediate appearance but more closely resemble the minor worker.

#### Queen diagnosis.

Larger to smaller than the conspecific major worker. Palp formula, ocelli, appearance of antenna and number of antennal segments, and number of mandibular teeth are as for the conspecific major worker. Mesoscutum about as wide as long in species seen. Parapsidal lines parallel and usually distinct. Axillae small and widely separated, the separation much greater than their width. The wing is of the ‘*Camponotus*’ form, with a radial cell, a first cubital cell and one submarginal cell. The cross-vein cu-a is present but the discoidal cell and cross vein m-cu are absent. Ergatoid or brachypterous queens have not been identified.

#### Male diagnosis.

(Caution: this diagnosis is based on only a small number of specimens that we have been able to identify as *Melophorus* males). Males with typical characteristics of males of this subfamily. The head bears an ocellar triangle that may be weakly turreted in some species, the antennal scape is usually longer to much longer than the head and the 13-segmented antenna is elbowed. The eyes are generally larger than those in the corresponding queen or major worker but not globose or bulbous in males seen. The mandible is 1- to 4-toothed in males seen; if 1-toothed, then apical tooth is prominent. The wing is as for the queen, but in one species a second small submarginal cell may be present, possibly limited to certain individuals. The parapsidal line is always present, weakly to strongly impressed; the notaulus is absent or present only as a median appendix arising from the base of the mesoscutum. The axillae are small and very widely separated from one another, the separation much greater than their width. Workerlike, wingless males are known for at least one desert-dwelling species.

#### Worker description.

**Minor worker. Head.** Head oval, square, cordate or rectangular, its posterior margin strongly convex to strongly concave. Compound eyes always present, usually medium to large (eye length generally 0.2× length of side of head capsule ≥), rarely small. Eye shape elliptical across most species, often slightly reniform due to an invagination of cuticle on the outer margin of the eye, much more rarely ovoid, roughly circular or oval tending to elongate; in some populations of *M. hirsutus* eyes flattened and embedded in cuticular prominences when seen in full-face view, or projecting roundly or even bizarrely conical. In full-face view, eyes mostly situated in the upper half of the head capsule, rarely (mostly in very small minor workers) at about the midpoint of the capsule, but never anteriad of the midpoint. In profile, eyes usually placed ahead of the midline of the head capsule, less frequently at about the midline or fractionally in front of it, but never behind the midline. Three small ocelli always present. In full-face view, frontal carinae convex, concave, straight, convergent posteriad or divergent posteriad, sometimes raised (particularly near antennal insertion), vestigial beyond antennal insertions in a few taxa. Torulus (antennal sclerite) pedunculated in a couple of species but otherwise inconspicuous. Antenna always 12-segmented. Scape very variable in length, but usually attaining the posterior margin of the head capsule, and often extending well beyond it. Clypeus never longitudinally carinate, or with protruding clypeal teeth but otherwise variable in appearance. Posterior margin of clypeus mostly arched and falling away from its mid-sector (i.e., between the frontal carinae), but lateral sectors of clypeus on same plane as mid-sector in a few species. Anterior clypeal margin often convex, with or without an anteromedial dimple, but concave, acuminate, spatulate and variously protrusive clypeal morphologies also occur. The midpoint of the clypeus may be notched, especially in the *aeneovirens* species-group. A clypeal psammophore of long setae (or ammochaetae) stretched across that sclerite is found in all *Melophorus* species, and may be located at any elevation, from at or just above the anterior clypeal margin to just under the posterior clypeal margin. Mandibular and gular psammophores of long, curved setae also usually present. **Mouthparts.** Palp formula mostly 6,4, but 3,4 and 3,3 also occur, maxillary palps variable in length from not reaching the hypostomal bridge to attaining the mesopleuron when they are fully extended. Apical segment of maxillary palp usually oblongiform, but may be narrow and acuminate or even vestigial and barely visible under a stereomicroscope. Mandible most commonly with five distinct teeth, but number of teeth and denticles ranges from four to over 15, mandible edentate except for apical tooth in one species. In five-toothed species apical tooth is nearly always much longer than remaining teeth and teeth 2 and 4 are typically longer than tooth 3 (teeth count taken from apical to basal tooth), basal tooth often offset and directed vertically. Masticatory margin usually vertical or weakly oblique, but strongly oblique in a few species. **Mesosoma.** Spines or denticles always absent from pronotum and mesonotum, present on propodeum only in *M. majeri*. Posterior pronotum planar, rounded or angled. Mesothoracic spiracles often opening on small dorsal prominences, these prominences stalk-like in *M. hirsutus*. Mesopleural process absent. Mesopleuron folds over onto flattened mesosternum without being demarcated by strong carina. Dorsal face of propodeum may be uniformly convex to flat, abruptly conical, or convex anteriad and flattened posteriad. Propodeal angle commonly absent, but may be sharply defined and is produced as denticles in *M. majeri*. Declivitous face of propodeum convex to flat, longer to shorter than dorsal face. Metanotal groove usually present, forming a weak to strong angle between the mesonotum and propodeum, but is absent or vestigial in a few species. Propodeal spiracle of variable appearance, but always prominent and longer than wide, usually situated at or near propodeal declivity, but nearer to midline of propodeum in some species-groups and in individual species. **Petiole.** Node always present. In profile, node often squamiform, but may be thicker, e.g., subcuboidal or triangular, and is typically directed posteriad. In full-face view, dorsum of node generally rounded, more rarely angulate, weakly acuminate or indented anteromedially. Petiole with a ventral lobe or keel. **Gaster.** First tergite vertical and somewhat overarching the petiole in dorsal view, with a small depression for receiving the rear of the node: **Legs.** Mesotibia and metatibia with one simple spur in most species, spurs reduced or absent in a few groups. **General characters.** Integument variable, from thick and sculptured to thin, flexible and glossy in very small minor workers of some species, mesonotum may be translucent. Sculpture, where present, predominantly consisting of microreticulation or fine striation, frank rugosity or larger striae found in very few species. Mesopleuron usually with some sculpture, even in otherwise smooth and glossy species. Appressed setae often small, and well-spaced, thick in some species producing pubescence and a silvery sheen in some lights. Erect setae tend to be short and bristly, more rarely fine, long; setae modified and spatulate or even clavate distally in a few species. Many species without erect setae on mesosoma and gaster, or with erect setae confined to margins of gastral tergites. **Proventriculus.** Proventriculus asepalous.

#### Major worker.

As for minor worker, but differing in the following particulars: **Head.** Usually planar or weakly concave, but may be strongly concave, never strongly convex. Head often strongly horizontally rectangular, distinctly broader than wide, especially in *M. wheeleri* species complex. Antennal scapes shorter than in corresponding minor worker, often barely exceeding or not even attaining the posterior margin of the head. Anterior margin of clypeus very variable and may be different to that of corresponding minor worker, often folded or turned back in large-headed species, one major worker with V-shaped anteromedial protuberance projecting anteriad. **Mouthparts.** Palps, if reduced, as for corresponding minor worker, but may be 3,2 (less than for minor worker) in one species. Otherwise, mouthparts as for corresponding minor worker in many species, but differing significantly in *M. wheeleri* and *M. laticeps* species complexes; here, major worker with powerful, truncate, inwardly curved mandibles with four to five teeth in taxa for which major worker is known (likely to be more in unconfirmed major worker of *Melophorus* species K (TERC)), basal margin folded over horizontally towards buccal cavity to form a planar surface that is delimited partially or fully along its length by a carina. Masticatory margin of mandible vertical, oblique or medially indented. (Characters of mesosoma and metasoma generally as for corresponding minor worker, but dorsal profile of mesosoma may vary, with pronotum and mesonotum generally more raised and convex. Erect setae nearly always present in major worker, even where the corresponding minor worker is glabrous).

### Synopsis of *Melophorus* species

*aeneovirens* species-group

*aeneovirens* complex

***aeneovirens*** (Lowne 1865)

=*constans* Santschi 1928 **syn n.**

=*insularis* Wheeler 1934 **syn n.**

=*iridescens* (Emery 1887) **syn n.**

=*iridescensfraudatrix* Forel 1915 **syn n.**

=*iridescensfroggatti* Forel 1902 **syn n.**

***attenuipes* sp. n.**

***canus* sp. n.**

***castaneus* sp. n.**

***clypeatus* sp. n.**

***curtus*** Forel 1902

***fulgidus* sp. n.**

***gibbosus* sp. n.**

***griseus* sp. n.**

***kuklos* sp. n.**

***mullewaensis* sp. n.**

***platyceps* sp. n.**

***praesens* sp. n.**

***rufoniger* sp. n.**

***sulconotus* sp. n.**

***tenuis* sp. n.**

***teretinotus* sp. n.**

*bagoti* complex

***bagoti*** Lubbock 1883

=*Camponotus cowlei* Froggatt 1896 **comb. rev.**

***gracilipes* sp. n.**

*nemophilus* complex

***nemophilus* sp. n.**

*anderseni* species-group

***anderseni*** Agosti 1998

***andersenioides* sp. n.**

***chrysus* sp. n.**

***subulipalpus* sp. n.**

*biroi* species-group

*biroi* complex

***argus* sp. n.**

***biroi*** Forel 1907

=*fieldipropinqua* Viehmeyer 1925 **syn. n.**

=*marius* Forel 1910 **syn. n.**

***castanopus* sp. n.**

***compactus* sp. n.**

***cuneatus* sp. n.**

***dicyrtos* sp. n.**

***graciliceps* sp. n.**

***gracilis* sp. n.**

***latinotus* sp. n.**

***lissotriches* sp. n.**

***longiceps* sp. n.**

***macrops* sp. n.**

***microreticulatus* sp. n.**

***minimus* sp. n.**

***mjobergi*** Forel 1915

***postlei* sp. n.**

***propebiroi* sp. n.**

***turbineus* sp. n.**

*brevignathus* complex

***brevignathus* sp. n.**

***marmar* sp. n.**

***quadratus* sp. n.**

*fieldi* complex

***ankylochaetes* sp. n.**

***bruneus*** McAreavey 1949

***eumorphus* sp. n.**

***fieldi*** Forel 1910

***fulvidus* sp. n.**

***gilliatensis* sp. n.**

***hirsutipes* sp. n.**

***incisus* sp. n.**

***inconspicuus* sp. n.**

***isaiah* sp. n.**

***lanuginosus* sp. n.**

***longipes* sp. n.**

***major*** Forel 1915 **stat. n.**

***microtriches* sp. n.**

***orthonotus* sp. n.**

***paramorphomenus* sp. n.**

***perthensis*** Wheeler 1934 **stat. n.**

***sericothrix* sp. n.**

***setosus* sp. n.**

***solitudinis* sp. n.**

***sulla*** Forel 1910 **stat. n.**

***turneri*** Forel 1910

=*pillipes* Santschi 1919 **syn n.**

=*turneriaesopus* Forel 1910 **syn. n.**

=*turnericandidus* Santschi 1919 **syn. n.**

***vitreus* sp. n.**

*oblongiceps* complex

***oblongiceps* sp. n.**

*wheeleri* complex

***brevipalpus* sp. n.**

***caeruleoviolaceus* sp. n.**

***cerasinoniger* sp. n.**

***chauliodon* sp. n.**

***diversus* sp. n.**

***hexidens* sp. n.**

***laticeps*** Wheeler 1915

***parvimolaris* sp. n.**

***pelorocephalus* sp. n.**

***prominens* sp. n.**

***purpureus* sp. n.**

(‘species K’ [TERC])

***wheeleri*** Forel 1910

=*omniparens* Forel 1915 **syn. n.**

***xouthos* sp. n.**

*fulvihirtus* species-group

***barbellulatus* sp. n.**

***fulvihirtus*** Clark 1941

*ludius* species-group

*hirsutus* complex

***hirsutus*** Forel 1902

*ludius* complex

***ludius*** Forel 1902

***pusillus* sp. n.**

***translucens* sp. n.**

*majeri* species-group

***majeri*** Agosti 1998

*potteri* species-group

***macroschismus* sp. n.**

***pelecygnathus* sp. n.**

***potteri*** McAreavey 1947

**Total**: 93 species

**Removed from *Melophorus***

***scipio*** Forel 1915 (to *Prolasius*) **comb. rev.**

### Key to *Melophorus* species based on workers

The key to species is based on workers, as species-level separation of the other adult castes is not only extremely difficult, but of very limited utility. Moreover, the reproductive castes of the less common species have either not been collected or are often unable to be associated with workers (since most material examined has come from trapping or opportunistic hand collection, rather than from nests). Major and minor workers of a specific *Melophorus* taxon have been included in the measurements where possible. However, for some uncommon species only minor or major workers are known. (Note. ‘species K’ (TERC) and other *Melophorus* specimens held in the TERC Collection were not available as loan material except under restrictive conditions that were not acceptable to the authors of this paper.

**Table:** 

1	Pronotum surrounded by translucent lamina; metanotal groove obsolete in minor worker, a weak furrow in major worker; torulus pedunculate around antennal insertions; cuticle of mesosoma with dense striolate-microreticulate sculpture (eastern Australian seaboard) (*M. ludius* species-group) (*M. hirsutus* species-complex) (Fig. 91(a–h)	***hirsutus***
–	Pronotum without translucent lamina; if cuticle of mesosoma with dense striolate-microreticulate sculpture, then metanotal groove always present, although it may be reduced to a slight transverse indentation; torulus never pedunculate as above (except in one rare population of *M. lanuginosus*)	**2**
2	Large, oblique propodeal spiracle situated well before declivitous face of propodeum, the spiracle bisecting much of the propodeum; mandible modified in two of the three species (either securiform, its masticatory margin with small to minute, blunt, evenly-sized teeth (except for the long, sharp, apical tooth), or edentate); PF reduced (3,4) in two of the three constituent species (*M. potteri* species-group)	**3**
–	Spiracle not as above, generally situated on declivitous face of propodeum; mandible and mandibular teeth not modified as above, teeth usually pointed	**5**
3	Mandible edentate or with minute crenulations along its masticatory margin; head capsule elongate-rectangular; anterior margin of clypeus straight; PF 3,4 (**JDM 1032**) (inland areas of least QLD, SA and WA) (Fig. 97a–g)	***pelecygnathus***
–	Mandible not edentate, mandibular teeth large enough to be easily distinguished; head capsule square, rectangular or oval, if rectangular then anteromedial clypeal margin protrusive; PF 3,4 or 6,4	**4**
4	PF 3,4; general appearance glossy; head capsule square or rectangular; anterior clypeal margin with square protrusion; basal margin of mandible denticulate; basal margin of mandible expanded distally (widespread) (Fig. 98a–g)	***potteri***
–	PF 6,4; general appearance matt; head capsule oval; anterior clypeal margin broadly convex but not protrusive; basal margin of mandible not denticulate; basal margin of mandible evenly curved throughout its length (**JDM 1082**) (inland SW WA) (Fig. 96a–d)	***macroschismus***
5	Propodeum armed with short, acute denticles; pronotum, mesonotum and propodeum flattened and delimited by blunt carinae in minor worker; antennal lobes raised, the sector between them recessed and oval-longitudinal in shape; metatibial apical spur absent (Fig. 95a–g) (*M. majeri* species-group)	***majeri***
–	Propodeum always unarmed; mesosoma never carinate as above, usually with rounded surfaces; if antennal lobes raised then sector contained within them triangular or hemispherical; metatibial apical spur well-developed in most groups	**6**
6	Head and mesosoma extensively covered with short, stout, peg-like bristles; in outline, pronotum and mesonotum flattened; metatibial apical spur stout but very short (*M. fulvihirtus* species-group)	**7**
–	Head and mesosoma not extensively covered with stout, peg-like bristles; pronotum and mesonotum generally sinuous in outline	**8**
7	Head, body and appendages covered in short, peg-like setae; cuticle strongly matt with coriaceous sculpture; posterior margin of head of all workers broadly concave; mandible of minor worker very finely striate, individual striae invisible under low power magnification (NSW, SA, Vic) (Fig. 90a–g)	***fulvihirtus***
–	Short peg-like setae much reduced on antennae and absent from legs; cuticle finely shagreenate and weakly shining; posterior margin of head of minor worker planar; mandible of minor worker coarsely striate (very rare, southern WA; SA) (**JDM 613**) (Fig. 89a–g)	***barbellulatus***
8	Maxillary palp segments short (not reaching neck sclerite), narrow and terminating in a subulate (awl-shaped) segment; PF 6,4; metatibial apical spur absent; in full-face view, masticatory margin of mandible strongly oblique with four teeth in known major workers (except *chrysus*), and four to six teeth in minor worker (*M. anderseni* species-group)	**9**
–	Maxillary palp of variable length, but often extending beyond neck sclerite and always with lobiform terminal segment; PF < 6,4 where palp segments extremely reduced and difficult to see; metatibial apical spur nearly always present and elongate; in full-face view, masticatory margin of mandible in major worker usually not strongly oblique, but if so, then almost always with at least five teeth; minor workers with ≥ five teeth	**12**
9	In profile, petiolar node thick and quadrate or broadly rectangular in the minor worker; major worker hairy and rather matt in appearance	**10**
–	In profile, petiolar node thickly squamiform in the minor worker; known major worker (*M. chrysus*) smooth and glossy in appearance	**11**
10	Anterior clypeal margin broadly convex and protrusive; clypeal psammophore located below midline of clypeus (major worker) or near its anterior margin (minor worker); antennal scape of minor worker devoid of erect setae (northern Australia) (Fig. 26a–h)	***anderseni***
–	Anterior margin of clypeus weakly convex, clypeus folded back and not protrusive; clypeal psammophore located at midline of clypeus; antennal scape of minor worker with many short, erect, bristly setae (**JDM 1105)** (mid-west WA; NSW) (Fig. 27a–d)	***andersenioides***
11	In profile, pronotum smoothly rounded and inclined at angle > 30°; head and body strongly shining to glossy, with superficial microreticulation only; in profile, clypeus evenly convex or more strongly convex posteriorly, but not bulbous; yellow; “*pillipes*” condition (whorls of fine, erect setae on appendages) in some populations (widespread but very rare) (**JDM 898**) (Fig. 28a–g)	***chrysus***
–	In profile, pronotum more-or-less flattened, only very weakly inclined anteriad; head and body weakly to moderately shining, the sculpture ranging from superficial microreticulation to evident shagreenation or minutely striate sculpture; in profile, clypeus strongly convex, tending to bulbous (major worker unknown); ochraceous; “*pillipes*” condition not seen (**JDM 1182**) (known from three collections only in WA, SA) (Fig. 29a–d)	***subulipalpus***
12	Setae of clypeal psammophore fine and placed at around the midpoint of clypeus; anterior of margin of clypeus a moderately flattened curve in all workers and not covering the base of the mandibles; five to seven mandibular teeth; major worker with same mandibular structure as minor worker; in profile, mesosoma long and gracile, with obliquely descending propodeum (*M. aeneovirens* species-group (part), [*M. nemophilus* species-complex]) (Bassian distribution) (one species: *M. nemophilus*) (**JDM 1036**) (Fig. 25a–h)	***nemophilus***
–	If with fine clypeal psammophore set at midpoint of clypeus and number of mandibular teeth more than five, then largest major worker with short, massive, elbowed mandible directed posteriad, mesosoma usually not long and gracile, and declivitous face of propodeum not as above	**13**
13	In full-face view, anterior clypeal margin convex, apron-like and covering whole or part of the retracted mandible, the medial clypeal sector often produced so that it is protrusive when seen in profile; psammophore frequently with coarse and well-separated ammochaetae, these always placed on or just above anterior margin; in profile, propodeum elongate and oblique or broadly rounded (*M. aeneovirens* species-group [part])	**14**
–	In full-face view, anterior clypeal margin variable, but not covering whole or part of the retracted mandible, the medial clypeal sector not narrowly protrusive, although it may be broadly protuberant; psammophore often placed along the midpoint of the clypeus or even above it; in profile, propodeum typically truncate or narrowly rounded (*M. biroi* and *ludius* species-groups)	**32**
14	In full-face view, psammophore in a row slightly above anterior clypeal margin; anterior margin of clypeus acuminate at its midpoint; metatibia may have more than two rows of preapical spines (*M. bagoti* complex)	**15**
–	In full-face view, psammophore ranged along or just above anterior margin of clypeus and following the curve of the margin; anterior margin of clypeus broadly medially produced, and often with central notch that may be deeply impressed, but is never acuminate at its midpoint; metatibia with maximum of two rows of preapical spines (*M. aeneovirens* complex)	**16**
15	Metatibia armed with two rows of preapical spines (**JDM 199**) (arid and semi-arid WA) (Fig. 24a–g)	***gracilipes***
–	Metatibia armed with five rows of preapical spines (arid areas; widespread) (Fig. 23a–k)	***bagoti***
16	Main teeth of mandible supplemented with small or indistinct denticles, total number of teeth and denticles ≥ six; in full-face view, head of minor worker indented below the eye, giving a bell-shaped appearance to the head capsule; workers matt, shagreenate, with very many long, flexuous setae over silvery pubescence (widespread but occasional) (**JDM 788**) (Fig. 8a–g)	***canus***
–	Teeth of mandible not as above, total number of teeth usually five; head of minor worker (weakly) laterally indented only in *Melophorus griseus*, which has five distinct mandibular teeth; in full-face view head shape square with rounded posterior angles, rectangular or oval, often with domed posterior margin of head; workers with or without erect setae, where present these usually short and bristly and never long and flexuous	**17**
17	In profile, head capsule very strongly compressed dorsoventrally, especially in minor worker; maxillary palps short, not reaching neck; in profile, dorsum of mesosoma more-or-less linear after weak anterior pronotal incline, the metanotal groove vestigial in minor worker and weakly impressed in media and major workers; anterior clypeal margin weakly convex; bicoloured; (Bassian distribution) (**JDM 787**) (Fig. 17a–g)	***platyceps***
–	In profile, if head capsule very strongly compressed dorsoventrally (*M. tenuis*), then mesosoma not more-or-less linear, although it may be generally elongate, and maxillary palps longer, reaching to neck sclerite	**18**
18	In full-face view, eyes of minor worker placed high on head, breaking outline of head capsule where the broadly convex posterior margin of head meets the sides; major and minor worker glabrous, smooth and shining, appressed setae spaced much greater than their own length apart; in profile, major worker with flat mesonotum that overarches the pronotum and; in full-face view, with a protruding, weakly bifurcate anteromedial clypeal margin (two pins, Coward Springs, SA) (Fig. 12a–g)	***fulgidus***
–	In full-face view, eyes of minor worker generally not breaking outline of head; if eyes of minor worker do break the outline of the head capsule, then eyes placed much lower on head; posterior margin of head generally strongly convex (domed) or more-or-less planar, but if tending to broadly convex, then worker not glabrous, smooth and shining (major worker often similar to the above, but can be distinguished if mesonotum is convex, *or*, the ant has dense appressed pubescence and/or short, erect setae on the mesosoma, *or*, anteromedial clypeal margin is not protrusive and bifurcate)	**19**
19	Minor worker very small (HW ≤ 0.50 mm); metafemur of minor worker of peculiar appearance, being attenuated to the midpoint and thereafter of uniform width to its junction with the tibia (media worker with similar condition, but attenuation more gradual and less conspicuous); head of minor worker rather elongate and very strongly domed; anteromedial sector of clypeus of major worker flattened towards its anterior margin and not extending beyond flanks of clypeus (midwest coast of WA only) (**JDM 1102**) (Fig. 7a–g)	***attenuipes***
–	Minor worker larger (HW > 0.50 mm); metafemur of normal appearance, i.e., gradually attenuating towards its junction with the tibia; head of minor worker generally roundly rectangular or square with a domed or planar posterior margin of head; anteromedial section of clypeus of known major workers protrusive to varying degrees and often also projecting when seen in profile	**20**
20	Tibiae with fine, appressed pubescence in addition to stout, socketed, appressed to subdecumbent setae	**21**
–	Tibiae with stout, socketed, appressed to subdecumbent setae only, fine, appressed pubescence lacking	**24**
21	In profile, minor worker (major worker unknown) with pronotum flattened posteriad and mesonotum also flattened before descending steeply to metanotal groove; minor worker larger (HW ≈ 1.50 mm); bright, light orange with brown gaster (Kimberley region of WA; NT) (Fig. 20a–d)	***sulconotus***
–	In profile, posterior pronotum and mesonotum of minor worker weakly to strongly convex; minor worker smaller (HW ≤ 0.90 mm); colour variable but not as above	**22**
22	Antennal scapes and tibiae without bristly erect and semi-erect setae, and short, erect setae normally sparse on dorsum of mesosoma; head of minor worker oval or squared and frontal triangle triangular; in profile, mesosoma of minor worker usually gracile, pronotum and mesonotum forming a gentle curve (non-gracile populations [e.g., Armidale, NSW] also lack bristly erect or semi-erect setae on antennal scapes or tibiae); upper frons of major worker partially shagreenate (very variable morphology, possibly two or more species represented here) (Eastern Australia [mainly]; NT, WA; no SA records) (Fig. 11a–g)	***curtus***
–	Antennal scapes and tibiae with bristly erect and semi-erect setae normally present on all surfaces, and short, erect setae moderately abundant on dorsum of mesosoma; if bristly erect setae abraded or absent from antennal scapes and tibiae, then head of minor worker bell-shaped in full-face view and frontal triangle narrowly semi-oval (some workers of *M. griseus*); in profile, pronotum and mesonotum may form a strong convexity; frons of major worker (only known for *Melophorus gibbosus*) uniformly distinctly microreticulate	**23**
23	Frontal carinae of minor worker (major worker unknown) raised and laminate at edges; frontal triangle narrowly semi-oval; sides of head concave below eyes, giving it a bell-shaped appearance; in profile, pronotum and mesonotum form a gentle curve (as in Fig.) (Kimberley region, WA) (**JDM 948**) (Fig. 14a–d)	***griseus***
–	Frontal carinae of workers not raised or laminate at edges; frontal triangle triangular; sides of head of minor worker not divergent below eyes; in profile, pronotum and mesonotum form a strong convexity (as in Fig. 38) (widespread but uncommon) (**JDM 1112**) (Fig. 13a–g)	***gibbosus***
24	In profile, mesosoma of minor worker with a compact appearance, its dorsal outline describing a pronounced arc due to shape of the mesonotum and mesopleuron (mesosternal outline and dorsum of mesonotum strongly convergent anteriorly)	**25**
–	In profile, mesosoma of minor worker tending to linear in orientation, its dorsal outline straight or describing a weak arc (mesosternal outline and dorsum of mesonotum weakly convergent to subparallel anteriorly)	**27**
25	Mesonotum and propodeum of minor worker confluent, metanotal groove completely lacking (major worker unknown) (northern WA; NT) (**JDM 897**) (Fig. 22a–d)	***teretinotus***
–	Metanotal groove present in minor worker, albeit may be shallow, mesonotum and propodeum clearly separated	**26**
26	In profile, clypeus distinctly recurved at about midpoint, produced over mandible as small ledge; in full-face view, anterior margin of major and minor worker clypeus a broadly convex, sometimes crenulate curve that does not protrude over apical curve of mandible; in profile, dorsum of minor worker mesosoma moderately arcuate (widespread; common) (Fig. 6a–g)	***aeneovirens***
–	In profile, clypeus straight or weakly and broadly convex, produced over mandible as a very pronounced ledge; in full-face view, anteromedial margin of major and minor worker clypeus produced as a narrow flange that is distinctly notched or even forked at its midpoint; dorsum of minor worker mesosoma strongly arcuate, almost elliptical (NT, Kimberley region WA) (Fig. 15a–g)	***kuklos***
27	In full-face view, anteromedial margin of clypeus produced as a narrow, rectangular flange with a straight or weakly indented edge (otherwise, anatomically identical with *M. praesens*) (Pilbara region, WA) (**JDM 1242**) (Fig. 10a–g)	***clypeatus***
–	In full-face view, anteromedial margin of clypeus not produced as a narrow, rectangular flange	**28**
28	Head of minor worker conspicuously dorso-ventrally compressed (seen in profile, head width < 0.5 × head length); anterior clypeal margin evenly convex (northern Kimberley, northern NT) (major worker unknown)	***tenuis***
–	Head of minor worker not conspicuously dorso-ventrally compressed (seen in profile, head width > 0.5 × head length); anterior clypeal margin with a medial notch or crenulation	**29**
29	In profile, pronotum of minor worker flattened; in dorsal view, pronotum of minor worker moderately attenuated anteriad; black, very gracile species (one pin only of two damaged minor workers from Mullewa, WA) (Fig. 16a–d)	***mullewaensis***
–	In profile, pronotum of minor worker convex; in dorsal view, pronotum of minor worker with globose outline, that sclerite not or only very weakly attenuated anteriad	**30**
30	Major and minor workers with medial sector of clypeus produced broadly as a flange that projects over the basal half of the mandibles; larger species (HW of large major workers ≥ 3 mm); foreparts light tan or orange to dark crimson (never brown), gaster brown (widespread, mainly arid and semi-arid) (**JDM 545**) (Fig. 19a–j)	***rufoniger***
–	Major and minor workers with clypeus broadly and gently convex, projecting over base of mandibles only; smaller species (HW of large major workers ≤ 2.3 mm); foreparts mostly brown, but can be black to light reddish-brown	**31**
31	Minor and media worker with slight medioccipital protuberance (best seen in full-face view with head tilted down slightly); pronotum and gaster of all workers with appressed setae overlapping or at least much closer to neighbouring setae than their own length; in profile, propodeum of minor worker with flattened, elongate dorsum, a barely discernible propodeal angle and often a sharp anterior peak (arid and semi-arid) (**JDM 304**) (Fig. 18a–g)	***praesens***
–	Minor and media worker without medioccipital protuberance, posterior margin of head broadly and weakly convex tending to planar when seen In full-face view, pronotum (and, often, gaster) of all worker castes with very short, often inconspicuous appressed setae, these separated from one another by much more than their own length; in profile, propodeum of minor worker without a sharp anterior peak, and commonly with a gently rounded dorsum and weak but discernible propodeal angle (arid and semi-arid) (**JDM 1034**) (Fig. 9a–g)	***castaneus***
32	Major and minor workers with combination of long mandibles (i.e., apical tooth of retracted mandible reaching to at least the tentorial pit on opposite side of head capsule), similar appearance of head capsule in major and minor workers and short maxillary palps, segments four to six combined barely longer than segment three, in profile, the entire palp reaching only to middle of venter of head capsule in minor worker when head is moderately inclined; head of major worker without short, massive, elbowed mandible directed posteriad; all workers with five mandibular teeth; median sector of clypeus uniformly convex and moderately protuberant, anterior clypeal midpoint a small, blunt angle (*oblongiceps* complex) (Lake Eyre Basin, SA) (Fig. 75a–g)	***oblongiceps***
–	Major and minor workers without combination of long mandibles, similar appearance of head in major and minor workers and short palps; if head of major worker similar to that of minor worker, then mandible short (i.e., apical tooth of retracted mandible reaching at most to antennal insertion on opposite side of head) (remaining *M. biroi* and *M. ludius* species-group members)	**33**
33	In full-face view, area around frontal carinae and medial sector of clypeus deeply recessed in major worker, and also in media workers of *Melophorus compactus*; in major, media and minor workers of one species, torulus (antennal sclerite) produced to form a pronounced and sometimes pedunculate flange that encircles the base of the antennal scape; psammophore generally placed on or just above anterior margin of clypeus; minor workers hairy with thick, longish, unmodified erect setae (pedunculate torulus present; clypeal psammophore placed at midpoint) or with bristly, short erect setae (pedunculate torulus absent; clypeal psammophore placed at or just above anterior clypeal margin)	**34**
–	In full-face view in major and media worker, area around frontal carinae and medial sector not noticeably recessed and torulus not expanded and pedunculated, torulus inconspicuous in all subcastes; most minor workers without the combination of anteriorly placed psammophore and hairiness with bristly, unmodified erect setae (minor workers of *M. sericothrix* may key out here as they are hairy with bristly setae, but these setae are longer, i.e., diameter of eye ≥, whereas, in *M. mjobergi* and *M. postlei* the bristly, erect setae are much shorter, i.e., diameter of eye <<)	**37**
34	Midline of head capsule between frontal carinae with a short, vertical flange in major and media workers; clypeal psammophore placed at around midpoint of clypeus; workers with thatch-like distribution of long white setae on pronotum, erect setae on mesosoma tend to be coarse and relatively long (one rare population from Pilbara region, WA) (**JDM 472** (pt))	***lanuginosus***
–	Midline of head capsule between frontal carinae without a short flange in major and media workers; clypeal psammophore placed on or just above anterior border of clypeus; workers with short, bristly, erect, unmodified setae on head, body and antennal scape (*biroi* complex (pt))	**35**
35	Head of major worker without distinct sculpture, strongly shining, setae-bearing sockets on head (if visible) appearing as tiny pits only, sculpture also reduced on head of media worker, which is moderately shining; in full-face view, antennal carina of major worker distinctly extended posteriad past antennal insertion, posterior extension of carina straight; eye of minor worker placed medially on head capsule and large in size (in profile, eye length ≈ 0.40× length of side of head capsule) (Eastern Australia) (Fig. 33a–g)	***compactus***
–	Head of all workers microreticulate and moderately shining to matt, setae-bearing sockets on head of major worker impressed so as to appear as small, oval depressions; in full-face view, antennal carina of major worker limited to flange around antennal insertion, weak ridge representing posterior extension of carina strongly concave posteriad; eye of minor worker placed anteriorly on head capsule and moderate in size (in profile, eye length ≈ 0.25× length of side of head capsule)	**36**
36	Appressed setae in all workers fine and forming pubescence that largely obscures underlying cuticle, which is matt in appearance (northern Australia) (**JDM 485**) (Fig. 44a–g)	***postlei***
–	Appressed setae in all workers loose and relatively thick and not obscuring underlying cuticle, which is moderately shining in appearance (northern Australia, apparently sympatric with above species) (**JDM 899**) (Fig. 43a–g)	***mjobergi***
37	Gaster with curved erect setae, semi-erect setae and a few decumbent setae only, genuine appressed setae lacking; body generally strongly sculptured and hirsute, antennal scapes and legs with whorls of many fine, straight setae (several hairy members of the *M. fieldi* complex)	**38**
–	Gaster with at least well-spaced appressed setae between the longer, erect or semi-erect pilosity if ant has hairy legs and scape	**40**
38	Mesonotum and propodeum globose, mesonotum, mesopleuron and propodeum separated from each other by a deep sulcus (WA, NT) (**JDM 945**) (Fig. 58a–g)	***incisus***
–	Mesonotum and propodeum not globose; mesonotum separated from mesopleuron and propodeum by weak groove or indentation	**39**
39	In profile, petiolar node of major and media workers narrow, squamiform, scale-like in appearance; (petiolar node of minor worker not so distinctive, but ant can be distinguished by more shining appearance of head and anterior mesosoma and the rounded propodeum); head of major worker shining in appearance (widespread arid and semi-arid) (**JDM 1070**) (Fig. 52a–g)	***ankylochaetes***
–	In profile, petiolar node of media and minor worker a low, rounded tubercle, that of major worker erect and not narrowly squamiform or scale-like in appearance; minor worker matt or with dull sheen only and with broadly truncate propodeum; head of major worker distinctly matt and may be rugulose (widespread, abundant) (**JDM 532**) (Fig. 58(g)-(k),(l))	***hirsutipes*** (part)
40	Small (HW of small minor workers 0.50 mm ≤, HW of known major workers ≤ 0.80 mm) species; weakly sculptured overall, with cuticle of mesosoma visibly thin, the mesonotum translucent to varying degrees; mesopleuron either smooth or with vestigial sculpture only (nb. taxa in couplet 40 are members of the *M. ludius* complex)	**41**
–	Usually larger species but if small, then cuticle of mesosoma thick and opaque (including the mesonotum), and mesopleuron generally with pronounced microreticulate or other impressed sculpture. (*Melophorus ludius* minor workers may be confused with minor workers of the following two taxa but can be distinguished from them by virtue of the combination of a moderate-sized eye and truncate [but not elongate] propodeum. In this case, however, the mesosoma is opaque though glossy.)	**42**
41	In profile, propodeum of all worker castes narrow and obliquely elongate, with a declivitous face that descends at an angle near 45°, minor worker propodeal spiracle elongate (≥ 0.67× length of propodeum, measured along the spiracle); eye moderate-sized (EL 0.12-0.15; EI 20-36); (southwest WA) (**JDM 500**) (Fig. 94a–g)	***translucens***
–	In profile, propodeum of all worker castes not narrow and obliquely elongate as above; minor worker propodeal spiracle shorter (≤ 0.50 length of propodeum, measured dorsoventrally); eye generally larger (EL 0.15–0.21; EI 31-40) (mainland Australia; arid) (**JDM 1235**) (Fig. 93a–g)	***pusillus***
42	In profile, head of minor worker weakly to moderately dorsoventrally compressed; in profile, eye set above midpoint of gena; clypeal psammophore set at around midpoint of clypeus to halfway between midpoint and anterior margin (major worker) and below midpoint to just above anterior margin (media and minor worker); head, body and legs of minor worker strongly pubescent, all workers with many short, unmodified, prickly, erect setae on head and body with from a couple to a moderate number distributed along antennal scapes, but such setae mainly or wholly absent from tibiae; propodeum with obliquely declivitous face; major worker with several preapical metatibial spines (Bassian distribution; SA and WA) (**JDM 617**) (Fig. 69a–g)	***sericothrix***
–	If minor and major worker similar in profile to the above and generally pubescent, then at least some short, erect setae modified (spatulate or thickened distally)	**43**
43	Minor and major workers worker clothed with fine, appressed silvery setae that form pubescence in minor worker, at least, in conjunction with multiple scattered, modified erect setae (modified setae varying from distally slightly flattened to clavate) on head, mesosoma and gaster (ants in couplets 43–57 belong to the *M. biroi* complex, (pt), except for *M. ludius*)	**44**
–	Minor and major workers not clothed with both fine, appressed silvery setae and multiple scattered, modified erect setae (either of these conditions may be individually present)	**45**
44	In full-face view, eye of minor worker moderately convex, bulging well beyond outline of head capsule; in profile, mesosoma of minor worker sinuous, the mesonotum dipping towards its junction with the propodeum, forming a weak v-shaped notch; frontal carinae of major worker straight or weakly convex, cuticle of major worker head matt or weakly shining and minutely pitted (widespread; arid) (**JDM 1121**) (Fig. 39a–g)	***lissotriches***
–	In full-face view, eye of minor worker only weakly convex, barely interrupting outline of head capsule; in profile, pronotum of minor worker rising gently, and mesosoma thereafter more-or-less straight, the mesonotum not dipping towards the propodeum, the metanotal groove not demarcated by a v-shaped notch; frontal carinae of major worker concave, cuticle of major worker head smooth and shining (mainly southern Australia; arid and semi-arid) (**JDM 784**) (Fig. 36a–g)	***graciliceps***
45	Metatibia of major worker with only one preapical spur; clypeal psammophore placed anteriorly, at or just above anterior margin of clypeus in minor worker and often in major worker; legs compact; head in profile dorsoventrally compressed in minor worker of many taxa, with eyes placed high on sides; mainly smaller species (HW of smallest minor 0.36 mm, average HW of smallest minors 0.46 mm; HW of largest known major 1.29 mm, average HW of largest majors (where known) 1.05 mm)	**46**
–	Metatibia of major worker and often minor worker with two or more preapical spurs; clypeal psammophore usually placed just below to above centrepoint of clypeus (caution: clypeus may be folded back giving appearance of anterior margin); legs often spindly with elongate metatibia in minor worker; head in profile usually ovate, rarely visibly dorsoventrally compressed; mainly larger species (HW of smallest minor 0.43 mm, average HW of smallest minors 0.77 mm; HW of largest known major 3.57 mm, average HW of largest majors (where known) 1.85 mm)	**59**
46	In profile, propodeal dorsum of minor and media workers extremely narrow and almost acuminate; metanotal groove in all workers narrowly and deeply impressed; mesonotum of minor worker hypertrophied so that metathoracic spiracle is situated on its underside in a distinctly lateral position; all workers with weak pubescence and a moderate number of erect setae on mesosoma (southwest WA) (**JDM 230**) (Fig. 34a–g)	***cuneatus***
–	Propodeal dorsum of minor and media workers mostly moderately truncate, but if narrow then metanotal groove v-shaped; mesonotum of minor worker not hypertrophied as above, the metathoracic spiracle situated on dorsum of ant or nearly so; workers without weak pubescence (except in minor worker of *Melophorus propebiroi*)	**47**
47	Minor worker with relatively long, partially overlapping appressed setae that form a weak pubescence on mesosoma and gaster; short, bristly, erect mesosomal setae present and plentiful (major worker unknown; southwest WA) (**JDM 1221**) (Fig. 46a–d)	***propebiroi***
–	Minor worker with short, non-overlapping appressed setae on mesosoma and gaster that do not form pubescence; short, bristly, erect mesosomal setae sparse or absent in minor worker	**48**
48	Profile of minor worker (major worker unknown) bimodal, with smooth, glabrous, rounded pronotum and propodeum, and scalloped mesopleuron; petiolar node thickly squamiform, almost tubercular (Torresian, in the far north) (Fig. 30a–d)	***argus***
–	Profile of minor worker not as above, specifically never bimodal and glabrous with a smooth rounded propodeum; propodeum most commonly truncate to varying degrees and propodeal angle may be raised; petiolar node more narrowly squamiform, and may strongly flattened, especially in the major and media workers	**49**
49	In profile, head of minor worker distinctly dorsoventrally flattened; in full-face view, head of minor worker extremely narrow, CI ≤ 75 (Lake Mere, NSW only, major worker unknown) (Fig. 40a–d)	***longiceps***
–	In full-face view, if head of minor worker dorsoventrally flattened, then distinctly broader, CI ≥ 85	**50**
50	Minor worker (major worker unknown) with propodeum produced vertically and conical in profile; propodeal spiracle very large, approximately 0.75× height of propodeum; eye larger (eye length approximately 0.40× length of side of head capsule) (two pins, Gawler Ra., SA) (Fig. 47a–d)	***turbineus***
–	Propodeum of minor worker not produced vertically except in *M. dicyrtos*, not conical in profile; propodeal spiracle shorter (≤ 0.60× height of propodeum)	**51**
51	Head and mesosoma of minor worker strongly matt, dull, with uniform, minute, net-like microreticulation; a transverse row of short, stout unmodified setae placed across centre of pronotum; in profile, propodeum weakly rounded or forming a slight angle between dorsal and declivitous faces (major worker unknown: known only from Eneabba region and Yanchep NP, WA) (**JDM 1180**) (Fig. 38a–d)	***latinotus***
–	Head and mesosoma of minor worker with moderate to strong sheen and without transverse row of short, erect pronotal setae as described above; microreticulate sculpture, where present, mainly confined to mesopleuron, except in *M. microreticulatus*	**52**
52	In full-face view, major worker with posterior clypeal margin not arched or falling away between antennal insertion and tentorial pit; anterior clypeal margin straight and not protuberant; in profile, major worker mesosoma elongate, smoothly bimodal; first gastral tergite of both major and minor worker with line of erect marginal setae and with other erect setae present, and workers very small (major worker HW ≤ 0.65 mm, minor worker HW ≤ 0.40 mm); minor worker with a pair or several long, erect setae at midpoint of pronotum; major and minor worker mesosoma glossy, with superficial sculpture only (northern WA only) (Fig. 37a–g)	***gracilis***
–	In full-face view, major worker often with posterior clypeal margin arched and falling away between antennal insertion and tentorial pit; anterior clypeal margin usually weakly convex, protuberant or with anteromedial dimple indicated; if major worker with posterior clypeal margin not arched or falling away between antennal insertion and tentorial pit and with glossy, unsculptured appearance (*M. ludius*), then profile compact with raised mesonotum and narrow, rounded propodeum; if minor worker small and with glossy, unsculptured mesosoma, then usually glabrous and always without erect marginal setae on first gastral tergite	**53**
53	In profile, propodeum of minor and major worker protuberant and strongly truncate, with elevated dorsal surface which is much shorter than declivitous surface; larger ants (HW of minor worker 0.65 mm ≥, HW of major worker 1.25 mm ≥); metanotal groove characteristically a deep V-shaped notch; most workers with long, flexuous setae but these may be short and bristly (northern NT, QLD and WA) (**JDM 470**) (Fig. 35a–g)	***dicyrtos***
–	In profile, propodeum of minor and major worker not of above appearance, propodeum either rounded or truncate, but if truncate then not narrowly produced with elevated dorsal surface; minor worker much smaller (HW 0.50 mm ≤) and known major workers usually smaller, except for *M. castanopus*; metanotal groove of minor worker usually weakly impressed (see figure below); minor workers often glabrous or nearly so	**54**
54	Eye large (EI 40-41); mesosoma with at least several erect setae, including propodeal setae; In profile, metanotal groove of minor worker deeply impressed and propodeum rounded into its declivitous face; first gastral tergite with erect setae, including a line of marginal setae; (major worker unknown) (v. rare; NSW and QLD) (Fig. 41a–d)	***macrops***
–	Without combination of large eye and erect marginal setae on first gastral tergite; in profile, metanotal groove of minor worker usually weakly impressed and propodeum usually more-or-less truncate, descending into its declivitous face through an angle; minor worker usually glabrous, though may have one or two erect setae on pronotum	**55**
55	Minor worker with combination of large eye (EI 40 ≥), small size (HW 0.40 mm ≥), lack of erect setae on mesosoma and first gastral tergite and distinct microreticulate sculpture over mesosoma (may be weaker on pronotum in some specimens); major worker with posterior clypeal margin not arched or falling away between antennal insertion and tentorial pit; anterior clypeal margin straight and not protuberant; major worker pronotum tending to bulbous and in profile breaking the mesosoma outline by protruding above the mesonotum; mesosomal sculpture as for minor worker (southern mainland Australia; arid and semi-arid) (**JDM 792**) (Fig. 42a–g)	***microreticulatus***
–	Minor worker without combination of large eye, small size and distinct microreticulate sculpture over mesonotum; erect marginal setae may be present; if major worker with clypeal conformation as above, then major worker mesosoma glossy and unsculptured or with vestigial microreticulation on mesopleuron and propodeum only, and pronotum does not protrude above the mesosomal outline when seen in profile	**56**
56	Smaller species (minor worker HW 0.40 mm ≤, largest major worker HW 1.05 mm ≤); in profile, minor worker head ovate without distinct dorsoventral flattening; major worker with posterior clypeal margin not arched or falling away between antennal insertion and tentorial pit; anterior clypeal margin straight and not protuberant	**57**
–	Larger species (minor worker HW 0.47 mm ≥, largest major worker HW 1.10 mm ≥); in profile, minor worker head weakly to strongly dorso-ventrally flattened, with eye placed high on side; major worker with posterior clypeal margin arched, falling away between antennal insertion and tentorial pit; anterior clypeal margin convex, protuberant and may be dimpled	**58**
57	In profile, minor worker propodeum weakly truncate or rounded, descending into its declivitous face at an angle of about forty-five degrees; minor worker eye moderate (EI 19-30); minor worker antennal scape longer (SI to 120); mesosoma and first gastral tergite occasionally with one or a couple of erect setae in eastern populations; major worker morphologically similar to the following, but larger (HW of measured major worker 1.05 mm) (widespread) (Fig. 92a–g)	***ludius***
–	In profile, minor worker propodeum strongly truncate and cuboidal, descending into its declivitous face at an angle approaching ninety degrees; minor worker eye large (EI 40-41); minor worker antennal scape shorter (SI as little as 94); mesosoma and first gastral tergite always glabrous; major worker similar to the above but minute (HW of measured major worker 0.47 mm) (arid QLD, SA and WA) (**JDM 1220**) (Fig. 43a–g)	***minimus***
58	Minor worker with erect setae on first gastral tergite, including a line of marginal setae; mesosoma also with a couple to a few, usually stout erect setae (several specimens may need to be seen because of abrasion), these setae rarely abundant and modified (one series, Pymble, NSW); in profile, pronotum, mesonotum and propodeum in all worker castes flattened and on same plane (NSW, SA, TAS and VIC) (Fig. 32a–g)	***castanopus***
–	Minor worker lacking erect setae on first gastral tergite; mesosoma also glabrous in minor worker; in profile, pronotum and mesonotum of minor worker tending to be rounded; mesosoma of major worker compact and rounded (similar to *M. ludius*); propodeum truncate (in which case it is below level of mesonotum) or rounded, the dorsal face much shorter than the declivitous face (widespread, abundant, possible species complex) (Fig. 31a–m)	***biroi***
59	In full-face view, head capsules of major, media and minor workers square with small, flattened eyes, except in media and minor workers of *M. marmar*, which have a large, convex eye (flattened in the major worker); in profile, eyes placed anteriad of midline of head capsule; anterior margin of clypeus distinctly sinuate, projecting anteromedially as a bluntly triangular extension or flattened dimple in major and media workers; five-toothed mandible of all workers very narrow, parallel and coarsely striate throughout its length; maxillary palps in all workers short, barely attaining neck sclerite at their greatest extremity and often only reaching midpoint of venter of head capsule when head is moderately inclined (*M. brevignathus* complex)	**60**
–	In full-face view, head capsule of major, media and minor workers differing in various respects from the above, which may include width and size of eyes, shape of head and conformation of clypeus and mandibles; in profile, eyes generally placed around midline of head capsule; if mandibles narrow, then elbowed, massive and directed posteriad in large major worker and often finely striate in minor and media worker; maxillary palps variable, may be long, reaching mesopleuron in small minor worker when head is moderately inclined	**62**
60	In full-face view, eye placed very high on capsule, and slightly above imaginary horizontal line separating head capsule (excluding mandibles) into equal upper and lower sectors; workers glabrous (NSW; NT, inland SA) (Fig. 50a–g)	***quadratus***
–	In full-face view, eye placed lower on head capsule, its bottom third intersected by an imaginary horizontal line separating head capsule (excluding mandibles) into equal upper and lower sectors; major and media worker, at least, with some erect pilosity on mesosoma, mesosoma of major and media worker may be relatively hirsute	**61**
61	In full-face view, sides of head capsule straight or even weakly concave anteriad, slightly divergent towards posterior margins of head; frontal carinae shorter and parallel, approximately 0.25× as long again as the vertical height of the frontal triangle; appressed setae on first gastral sclerite longer, separated from preceding and succeeding rows by ≤ 1× their own length; eyes of minor and media workers large and convex (inland SA; WA) (Fig. 49a–h)	***marmar***
–	In full-face view, sides of head capsule distinctly convex; frontal carinae longer, divergent posteriad, approximately 0.50× as long again as the vertical height of the frontal triangle; appressed setae on first gastral tergite shorter, separated from preceding and succeeding rows by at least 2× their own length; eyes of minor worker similar to those of major worker (NT, southern QLD) (Fig. 48a–g)	***brevignathus***
62	Minor and major worker with five mandibular teeth; major worker with similar mandible to minor worker, not massive, elbowed and directed posteriad; in profile, maxillary palps long, at least in minor worker, attaining mesopleuron when head of ant moderately inclined; in full-face view, anterior margin of clypeus in all workers usually weakly and evenly convex, commonly with anteromedial dimple or weak protuberance (*M. fieldi* complex)	**63**
–	Minor worker often with more than five teeth (up to 15), largest major worker with short, massive, elbowed mandible directed posteriad; in profile, maxillary palps shorter in major and generally in minor workers, in minor worker, when head of ant moderately inclined, only attaining neck sclerite at their maximum extent in most species; in full-face view, anterior margin of clypeus in large major worker usually planar or weakly concave, other subcastes variable but planar or narrowly protuberant anterior clypeal margins predominate (*M. wheeleri* complex)	**87**
63	Worker thorax with metanotum apparently developed and confluent with mesonotum, often extending over the propodeum; metanotal suture obsolete, its position indicated only by a superficial, transverse furrow (more pronounced in major worker); propodeum reduced in size and wedge-shaped, with narrow end of wedge often under fold of metanotum; metathoracic spiracle lateral and situated within metanotal sector (NT; WA) (**JDM 1063**) (Fig. 67a–g)	***paramorphomenus***
–	Worker thorax of normal appearance, with metathoracic spiracle situated on or near dorsum of mesosoma	**64**
64	Major, media and minor worker uniformly microreticulate, in profile, petiolar node of minor and media thick and tuberculate in shape, that of major worker squamiform; tibial apical spur absent; mesosoma of minor and media workers matt, glabrous, that of major worker with ≈ 12 short, bristly, erect setae (minor and media workers orange, major worker orange with brown gaster) (NT, QLD, northern WA; arid; rare) (**JDM 970**) (Fig. 56a–g)	***fulvidus***
–	Appearance not as above: specifically, if orange with thick petiolar node, then appearance not uniformly microreticulate, and erect setae, most commonly flexuous, present on mesosoma of all workers; tibial apical spur present	**65**
65	At least lower metafemur (shown here) and all of metatibia with whorls of erect setae directed at 45–90° to surface	**66**
–	Metafemur and metatibia without whorls of erect setae directed at 45-90° to surface of extremity, pilosity usually restricted to appressed or subdecumbent setae (where erect setae present, these confined to two or three lines of setae, more commonly several individual setae)	**70**
66	Tibiae and antennal scape matt, strongly microreticulate; erect setae on metatibia shorter, stout, length of longest setae < greatest width of tibia; appressed metatibial setae thickly distributed and often forming a distinct pubescence, metatibia matt; gaster of minor worker strongly pubescent (common and widespread) (**JDM 520**) (Fig. 65a–g)	***microtriches***
–	Tibiae and antennal scape moderately to strongly shining and smooth or with superficial microreticulation; erect setae on metatibia longer, fine, length of longest setae ≥ greatest width of tibia; appressed metatibial setae not conspicuous, metatibia shining; gaster of minor worker usually without long, overlapping appressed setae that form pubescence	**67**
67	Mesopleuron smooth and shining in minor and media worker at least, ant very glossy; HW of small minor 0.65 mm ≤	**68**
–	Mesopleuron with at least superficial sculpturing in all workers, ant often shining, but not glossy overall; HW of small minor usually 0.65 mm >	**69**
68	Ant gracile, with outline of mesosternum parallel to outline of mesonotum; in profile, outline of clypeus distinctly protuberant; in profile, petiolar node lower (» 1.2× as high as wide) (major worker unknown) (NT, QLD, WA; arid) (**JDM 699**) (Fig. 74a–d)	***vitreus* (‘ *pillipes* ’)**
–	Ant compact, with outline of mesosternum strongly anteriorly convergent to outline of mesonotum; in profile, outline of clypeus weakly convex; in profile, petiolar node higher (» 1.4× as high as wide) (this form mainly Eastern Australian) (Fig. 73m–r, y)	***turneri* (‘ *pillipes* ’)**
69	Anteromedial dimple prominent and protruding as a V-shaped lip; gaster and usually pronotum (at least) with overlapping long, appressed setae that form a silvery thatch in between long, curved, erect setae (arid Australia) (**JDM 472**) (Fig. 62a–g)	***lanuginosus* (‘ *pillipes* ’)**
–	Anteromedial dimple weakly developed or vestigial and not protruding as a V-shaped lip; appressed setae between long, curved, erect setae shorter and not forming a silvery thatch (widespread) (Fig. 58a–f, l)	***hirsutipes*** (part) **(“*pillipes*”)**
70	With combination of: (1) eye relatively large (eye length 0.50× length of side of head capsule in minor worker, approximately 0.33× length of side of head capsule in major worker ≥), (2) in full-face view, periphery of upper frons surrounded to about the level of the eyes with short, bristly, erect setae that are often flattened distally; (3) minor worker small (HW ≈ 0.56–0.59 mm); (4) non-iridescent head of major worker relatively smooth and gleaming (e.g., Fig. 54a) and, (5) clypeal psammophore of fine setae placed at or about midpoint of clypeus	**71**
–	Without this combination of characters: specifically, if with relatively large eye and modified setae on the periphery of the frons, then minor worker larger (HW > 0.73mm) and major worker head with fine microreticulation and pitting or iridescent (e.g., Fig. 60a); in other cases setae on frons long and unmodified, and may be lacking entirely	**72**
71	Minor worker concolorous blackish-brown, finely shagreenate with a dull silky sheen; in profile, pronotum rises gradually to its junction with mesonotum without any obvious convexity, while mesonotum is slightly humped; mesosoma with a few to many short, straight, bristly setae, these often expanded distally, length of longest setae ≤ greatest length of antennal scape; appressed setae short, inconspicuous; major worker and media worker concolorous brown (orange-yellow with brown gaster in holotype), with scattered short, sometimes modified setae; appressed setae on gaster well-separated and do not overlap (mainly semi-arid) (Fig. 55a–g)	***fieldi***
–	Minor worker variable shades of brown or dark reddish-brown but not blackish-brown with dull, silky sheen; in profile, pronotum smoothly convex, mesonotum completes the curve without a median hump; erect setae on mesosoma often numerous and flexuous, length of longer setae ≥ greatest width of antennal scape (in relatively glabrous minor workers, appressed setae on mesosoma long and conspicuously pale); major worker similar to the above, but short, sometimes modified setae are far more numerous and give a shaggy appearance to most major workers; appressed setae on gaster longer and overlap (mainly semi-arid) (Fig. 53a–g)	***bruneus*** (part)
72	Body of major and minor worker clothed with modified, erect setae (distally thickened, clavate or spatulate) to various degrees in addition to unmodified setae; frontal carinae straight or weakly convex	**73**
–	True modified, erect setae absent from body of major and minor worker (although some erect setae may be thick and short); frontal carinae divergent posteriad	**74**
73	In full-face view, sculpture of major and minor worker head matt with microreticulate sculpture; frontal carinae weakly divergent posteriad; appressed setae on appendages sparse, thick and mostly separated from each other by more than their own length (inland WA) (**JDM 1247**) (Fig. 71a–g)	***solitudinis***
–	In full-face view, sculpture of major and minor worker head pitted and weakly gleaming with superficial microreticulation; appressed setae on appendages abundant and fine, individual setae strongly overlapping (northern inland areas; rare) (**JDM 618**) (Fig. 70a–g)	***setosus***
74	Pale yellow, depigmented ants; in full-face view often with clypeus, genae and mandibles paler than frons; mesosoma very often glabrous (widespread, arid and semi-arid) (Fig. 72a–g)	***sulla*** (*sensu stricto*)
–	Ants darker or bicoloured, often with some erect pilosity on mesosoma	**75**
75	In profile, pronotum smoothly rounded and mesonotum elongate and broadly convex and strongly developed in all workers, so that pronotum and mesonotum combine to form a near semi-circle; minor worker with truncate propodeum with a protuberant dorsum that usually rises above metanotal groove; colour of minor worker uniformly brown or brown with slightly darker gaster, major worker brown or orange-brown with dark brown gaster (widespread and abundant) (Fig. 68a–g)	***perthensis* Wheeler**
–	In profile, mesonotal outline not as above, if mesonotum strongly convex then pronotum with flattened dorsum and/or mesonotum not elongate; propodeal dorsum of minor worker usually planar or weakly convex though may be high; colour variable, but often with gaster much darker than foreparts in minor worker	**76**
76	In profile, major and media worker with smooth, elongate propodeum, propodeal angle indicated by only a faint curve; metanotal groove a weak impression so that mesonotal and propodeal outline is barely interrupted; mesonotum and mesopleuron not separated by impression or suture; mesosternal outline strongly convergent anteriad with outline of mesonotum (media and major workers only known; bicoloured orange-and-dark-brown ants) (a few pins, Pilbara region, WA) (**JDM 1161**) (Fig. 66a–d)	***orthonotus***
–	In profile, propodeum of major and minor worker not elongate as above, and usually with at least a rounded propodeal angle; metanotal groove distinct and mesonotum and propodeum not nearly confluent as above; mesonotum and mesopleuron indicated by distinct impression if not a suture; mesosternal outline usually more-or-less parallel with outline of mesonotum	**77**
77	In full-face view, eye of all workers placed very high on capsule, and slightly above imaginary horizontal line separating head capsule (excluding mandibles) into equal upper and lower sectors; workers reddish-brown with pale tibiae and distal femur (WA, SA and NT) (**JDM 1246**) (Fig. 61a–g)	***isaiah***
–	In full-face view, eye placed lower on head capsule, at least its bottom eighth intersected by an imaginary horizontal line separating head capsule (excluding mandibles) into equal upper and lower sectors	**78**
78	In profile, clypeal psammophore in minor worker placed just behind anterior clypeal margin and well below midpoint of clypeus in major worker; eye in most specimens large (eye length 0.20 to 0.25 × length of side of head capsule in major worker, 0.33 to 0.50 × length of side of head capsule in minor worker); propodeum in major worker narrow with an oblique dorsal face; shining brown with gaster slightly darker (NSW, WA) (Fig. 64a–g)	***major***
–	In profile, clypeal psammophore placed just below midpoint to above midpoint of clypeus; eye variable but often smaller	**79**
79	Clypeus folded back abruptly slightly below its midpoint, so that anterior portion projects as a protuberance at about 90° to mandible; clypeal psammophore placed along line of demarcation (rare, localised; QLD) (Fig. 57a–g)	***gilliatensis***
–	Clypeus generally rounded or flattened towards its anterior margin, not abruptly folded back as above	**80**
80	Appressed setae on the gaster in all workers very small and inconspicuous when gaster is moderately distended, separated from one another by at least their own length, these appressed setae also inconspicuous on mesosoma and never long and silvery; mesosoma glabrous in minor worker or with one or a few flexuous, erect setae; node of minor worker often squamiform; most commonly cuticle shining or even glossy with vestigial or weak shagreenation	**81**
–	Appressed setae on gaster of all workers longer and overlapping when gaster is moderately distended, and may form a silvery thatch, appressed setae on mesosoma also overlapping and may be long and cross-linked forming a silvery thatch; mesosoma most frequently with numerous setae, if these sparse then often shorter and stout; node of minor worker characteristically not squamiform, and may be bent distally; most commonly cuticle distinctly shagreenate with weak to moderate sheen	**84**
81	Minor worker very small (HW < 0.55 mm); frons of head capsule smooth and shining; eye large (eye length approximately 0.40 × length of head capsule); mesopleuron with distinct, wrinkled or scalloped sculpture that may extend to the mesonotum; propodeum a rounded cube; major worker also very small (HW ≤ 0.73 mm) with large eye (eye length approximately 0.38 × length of head capsule); mesopleural sculpture as for minor worker (**JDM 786**) (Bassian, inland) (Fig. 54a–g)	***eumorphus***
–	Ant larger (minor worker HW 0.57 mm ≥; known major workers HW > 1.50 mm); if minor worker smooth and shining and generally similar (smallest minor workers of *M. vitreus*) then mesopleuron smooth and unsculptured or with very superficial microreticulate pattern	**82**
82	In profile, minor worker gracile, with short, thick petiolar node terminating in a smoothly rounded vertex; clypeus rounded and somewhat protuberant (see Fig. 74); eye very large (eye length ≥ 0.50 × length of side of head capsule); cuticle extremely glossy, with mesopleuron completely smooth or with very superficial microreticulate pattern (sp. **JDM 699**)	***vitreus*** (*sensu stricto*)
–	In profile, minor worker less gracile with generally squamiform node that is often inclined at its vertex; clypeus weakly protuberant and usually with distinct dimple at its midpoint (see fig); eye moderate to large (eye length 0.20–0.49× length of side of head capsule); cuticle less glossy tending to matt in some populations, with mesopleuron weakly shagreenate or microreticulate	**83**
83	Metafemur of minor worker shorter and stouter (0.75 × length of mesosoma ≤); in profile, propodeum generally with a weak to strong angle between dorsal and declivitous surfaces; major worker difficult to distinguish from that of *M. longipes*, but mesonotum generally moderately convex in profile and metafemur uniformly pale brown ochre to yellowish; tibia same colour as femur (variation across populations and molecular data suggest the possible presence of one or more cryptic species, but these cannot be defined morphologically) (widespread, abundant) (Fig. 73a–l, s–y)	***turneri* Forel** (*sensu stricto*)
–	Metafemur of minor worker longer and attenuated towards its junction with tibia (0.90 × length of mesosoma); in profile, dorsum of propodeum smoothly curved on to its declivitous face; mesonotum of major worker flat to weakly convex and metafemur increasingly depigmented towards its articulation with the tibia, and the tibia depigmented yellowish-white (NT, SA, WA) (**JDM 474**) (Fig. 63a–g)	***longipes***
84	Mesosoma glabrous; in full-face view, head strongly microreticulate, matt; eye relatively large (EI ≈ 41), appressed setae on gaster elongate and pale	***bruneus*** (minor workers in some populations)
–	Mesosoma nearly always with some erect setae (usually abundant), where lacking (a few minor workers of *M. inconspicuus*) eye smaller (EI ≤ 37) and, in full-face view, head always with some sheen	**85**
85	Mesosoma with erect setae long, flexuous and curved (pronotal setae and those on anterior mesosoma curved posteriad, setae on posterior mesonotum curved anteriad, those on propodeum also curved in both directions); appressed setae on mesonotum and gaster rather long but not combining to form a silvery pubescence; in profile, anterior two-thirds of clypeus often straight or slightly concave, forming an oblique surface, the anteromedial clypeal dimple weak or vestigial; in profile, petiolar node in minor worker thick, to about 0.7× as wide as high, and its dorsum may be noticeably directed posteriad, especially in minor worker (**JDM 532**) (similar to Fig. 58a–f, l)	***hirsutipes*** (part)
–	Erect mesosomal setae (where present) not long and flexuous, though they may be curved, however, these setae are frequently stout and bristly; appressed setae on mesonotum and gaster may be long and silvery in appearance forming a thick, thatch-like pubescence; in profile, clypeus evenly convex or weakly concave in its anterior third, but not as above; petiolar node thinner, much less than 0.7× as wide as high and either straight or weakly bent posteriad	**86**
86	Appressed setae on mesosoma and gaster long, forming a coarse, silvery pubescence; erect setae on mesosoma longer (longer setae > diameter of eye); cuticle with strong shagreenate sculpture, matt or with weak sheen; petiolar node in minor worker thicker, to about 0.6× as wide as high, also not a true scale in major worker; ‘*pillipes*’ condition present in some populations (see above) (**JDM 472**) (Fig. 62a–g)	***lanuginosus*** (part)
–	Appressed setae on mesosoma and gaster shorter, silvery pubescence limited to small areas of the pronotum or entirely absent; erect setae on mesosoma generally fewer and more bristly in appearance (often < diameter of eye) and entire mesosoma may be glabrous; cuticle generally with weak shagreenate sculpture, except for mesopleuron, and foreparts commonly moderately shining; petiolar node in minor worker essentially squamiform, that of major worker a true scale; ‘*pillipes*’ condition always absent (widespread, abundant) (**JDM 28**) (Fig. 60a–g)	***inconspicuus***
87	Minor worker mandible with 15 or more sharp teeth (major worker not inspected) (Victoria River district, NT (Andersen 2007))	‘**species K**’ (TERC)
–	Minor worker with nine teeth ≤	**88**
88	Anterior margin of clypeus in major and media workers a prominent rim that is produced anteromedially as a broadly angulate projection, this projection directed anteriad at angle of about 90° to head capsule when seen in profile; mandible of major worker usually with three distinct teeth (fourth tooth reduced to an angle in most cases); mandible of minor workers minutely striate with six or more teeth on masticatory margin (mid- and north-west WA) (**JDM 1244**) (Fig. 85a–g)	***prominens***
–	If mandible of minor worker minutely striate with six or more teeth on masticatory margin, then major and media workers without above clypeal configuration (in similar species, anteromedial clypeal projection is directed ventrally); major worker with four or five teeth.	**89**
89	Palps extremely short and variable in appearance and number of segments, palps when directed posteriad not reaching the hypostomal border, PF 4,3 or 3,3 or even apparently 2,3 (in undissected major worker) (interior SA) (Fig. 76a–h)	***brevipalpus***
–	Palps longer, extending beyond hypostomal border and PF always 6,4	**90**
90	Colour pale, depigmented yellow; colour of frons in full-face view often two-tone, colour lighter on lower genae (minor and media workers indistinguishable from *M. sulla* [*sensu stricto*]) (a few records from inland WA)	‘ ***sulla*** ’
–	Colour not pale, depigmented yellow	**91**
91	Strongly bicoloured, relatively smooth species with black, brown or variegated brown head, orange mesosoma and black gaster, gaster and often head (in darker morphs) with distinct bluish to violet iridescence; shorter setae on frons and pronotum may be expanded, mainly at their tip (arid inland Australia) (**JDM 1245**) (Fig. 77a–g)	***caeruleoviolaceus***
–	Species without characteristic bluish or violet iridescence on gaster; erect setae on head and pronotum usually fine, erect setae often lacking entirely on mesosoma	**92**
92	Anterior sector of the clypeus of minor worker strongly folded back towards the mandible, and clypeal psammophore placed on a distinct ledge that may be carinate, minor worker mandible with 5-9 teeth and denticles; head, mesosoma and gaster of all workers with short, inconspicuous appressed setae that are usually separated by more than their own length on the gaster (if more elongate, as in some small minor workers, then ant is glossy and weakly sculptured); media and major workers larger (HW of large major worker ≥ 2.60 mm)	***wheeleri* cluster** (see couplet 93 below)
–	If clypeus distinctly folded back towards the mandible and clypeal psammophore placed on a ledge in minor worker, then head, mesosoma and gaster with relatively long, whitish, appressed setae that overlap and form a weak pubescence on the gaster and ant has distinct microreticulate or shagreenate sculpture and is matt or has a weak sheen (in such cases, minor worker with five mandibular teeth and HW of known major worker smaller than above [HW ≈ 1.45 mm])	**94**
93	Head of minor worker smooth, with vestigial sculpture and often glossy; erect non-marginal setae always present on first gastral tergite, with one or two small erect setae also present on the pronotum of some individuals; eye of minor worker large (0.30× length of side of head capsule); major worker usually with many fine, erect setae on mesosoma (though these may be more sparse and bristly in a few individuals) and dark crimson or reddish-black with a glossy head capsule that is slightly lighter in colour (inland QLD, NSW, one NT record) (Fig. 80a–g)	***diversus***
–	Head of minor worker always with some sculpture and matt to moderately shining in appearance; erect setae almost always lacking on pronotum and first gastral tergite, but if present (a few SA populations) then ant of intermediate appearance; eye of minor worker usually less than 0.30× length of side of head capsule; major worker similar to above, but either with glabrous mesosoma or with short, bristly setae on that part, commonly matt in appearance (WA, NT) but may be glossy (eastern states) with bright orange (rarely, dark crimson) head capsule (other Australia) (Fig. 87a–j)	***wheeleri***
94	Clypeus distinctly folded back towards mandible and clypeal psammophore placed on a ledge in minor worker (carina may be present or absent in a given individual–if absent, check for long, appressed setae); head, mesosoma and gaster with relatively long, whitish, appressed setae that overlap and form a weak pubescence on the gaster in minor and known major workers; cuticle conspicuously shagreenate or microreticulate and matt or with a weak sheen	**95**
–	Clypeus variable in appearance (flattened or weakly protuberant or depressed anteriorly) but not distinctly folded back as above; head, mesosoma and gaster with short, inconspicuous appressed setae that are usually separated by more than their own length on the gaster (if more elongate, as in some small minor workers, then ant is glossy and weakly sculptured)	**96**
95	Ant uniformly brown; minor worker smaller (HW ≈ 0.70 mm) (NT, northern WA) (**JDM 951**) (Fig. 83a–g)	***parvimolaris***
–	Ant bicoloured, with tawny orange head, antennae and mesosoma, and black gaster and legs (legs have a bluish iridescent sheen); minor worker larger (HW ≈ 0.90 mm) (major worker unknown) (inland SA, WA; a few records only) (**JDM 1033)** (Fig. 88a–d)	***xouthos***
96	All workers with narrowly convex mesonotum; pronotum steeply (i.e., 60° >) inclined and flattened dorsally; minor workers with maximum of five mandibular teeth; major worker with all teeth in the same plane; bright red or reddish-orange ants with dark brown gaster (SE Australia) (Fig. 78a–g)	***cerasinoniger***
–	Minor and media workers, at least, with straight or only weakly sinuous mesonotum; pronotum in minor and media workers moderately (i.e., ≈ 45°) inclined and curved throughout its length; minor workers may have more than five mandibular teeth; if major worker similar in colour and profile, then basal tooth is weakly to strongly offset	**97**
97	Minor worker (major worker unknown) very similar to *M. fieldi* complex minor workers in appearance, but with six mandibular teeth and short maxillary palps (as described in couplet 33); basal tooth the same size as the other non-apical teeth and not offset, masticatory margin of mandible almost vertical; in full-face view, anterior clypeal margin evenly convex; in profile, clypeus protuberant (as in many *fieldi* complex minor workers); clypeal psammophore set at midpoint of clypeal sclerite (NSW) (Fig. 81a–d)	***hexidens***
–	If minor worker with combination of six teeth and clypeal psammophore set at midpoint of clypeal sclerite, then masticatory margin of mandible oblique and basal tooth distinctly offset and may be enlarged; anterior clypeal margin generally planar or nearly so in full-face view	**98**
98	In full-face view, broad head capsule of minor worker expanded towards mandibular insertions giving it a slight to strongly accentuated trapezoidal shape; basal margin of known major worker mandible a carinate ledge set at 90° to the rest of the mandible throughout its length, the (slightly) offset basal tooth horizontal and the sub-basal tooth with a horizontal and a vertical plane; mandibular carina present to varying degrees in media and minor workers; workers with glabrous mesosoma	**99**
–	In full-face view, sides of head capsule of minor worker convex, straight or even slightly concave, head square; apical margin of major worker mandible not completely carinate throughout its length; carina absent or reduced to a blunt edge in mandible of minor worker; erect setae present on mesosoma of nearly all *M. purpureus* workers (a few minor workers glabrous) and some *M. chauliodon* major workers	**100**
99	In full-face view, genae of minor and media worker strongly divergent towards anterior angles of head capsule, the latter noticeably trapezoidal, an imaginary perpendicular line drawn from the base of the eye excluding a section of the anterior clypeal margin whose width is greater than the width of the eye seen from in front; masticatory margin of mandible strongly oblique; head brown above lighter below, upper frons distinctly darker than mesosoma (large major worker not seen) (inland WA, possibly SA) (**JDM 1079**) (Fig. 84a–g)	***pelorocephalus***
–	In full-face view, genae of minor worker weakly divergent towards anterior angles of head capsule, the latter basically square, an imaginary line drawn from the base of the eye excluding a section of the anterior clypeal margin whose width is about that of the eye seen from in front; masticatory margin of mandible weakly oblique; head and mesosoma of the same shade (i.e., tan to orange-tan) (arid inland Australia) (**JDM 971**) (Fig. 82a–g)	***laticeps***
100	Eye of minor worker an elongated ellipse, length of eye ≥ 0.3 × length of side of head capsule; at least minor and media workers glabrous; major and media workers with basal mandibular tooth strongly offset to varying degrees, often tusk-like (widespread) (**JDM 783**) (Fig. 79a–g)	***chauliodon***
–	Eye of minor worker spheroidal, length of eye ≤ 0.25 × length of side of head capsule; minor and media workers often with two or more bristly erect setae, especially on pronotum; offset basal tooth of major and media workers not prominent and never tusk-like (southwest WA) **(JDM 1077**) (Fig. 86a–g)	***purpureus***

**Key figure 1:**
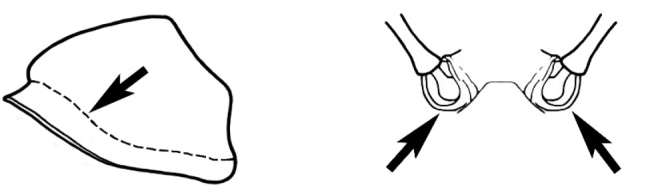


**Key figure 2:**
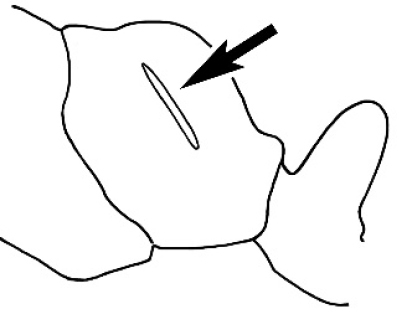


**Key figure '2:**
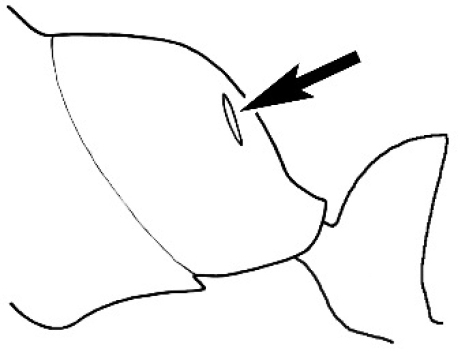


**Key figure 3:**
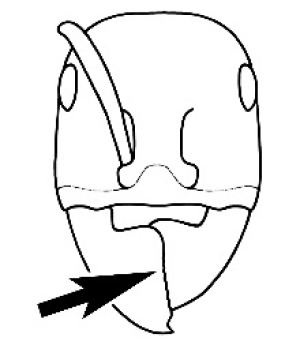


**Key figure '3:**
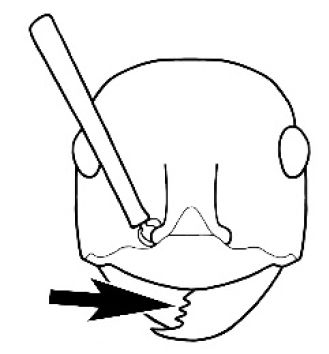


**Key figure 4:**
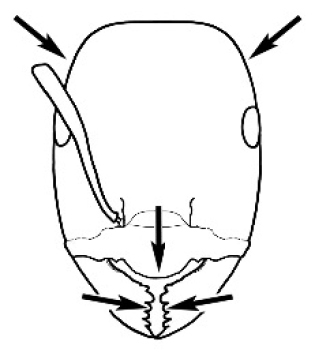


**Key figure '4:**
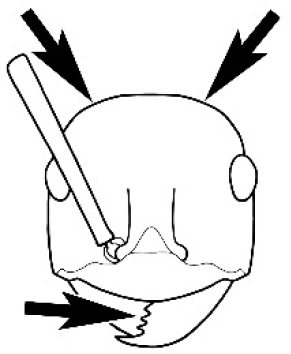


**Key figure 5:**
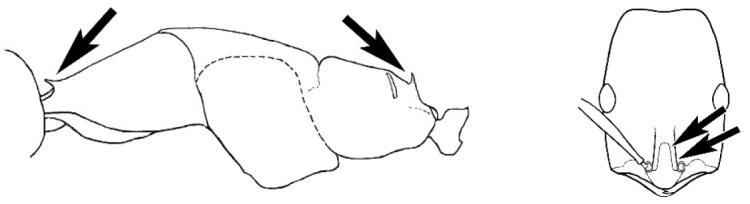


**Key figure 6:**
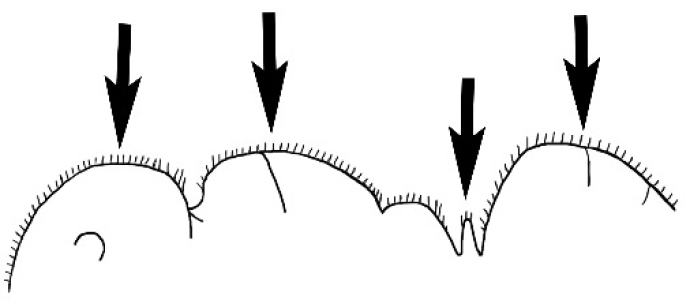


**Key figure 7:**
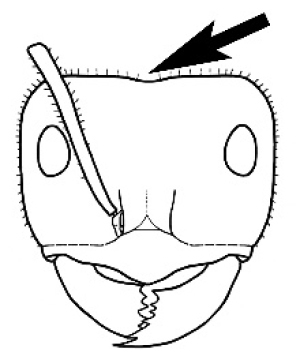


**Key figure '7:**
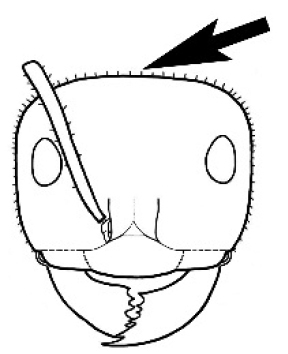


**Key figure 8:**
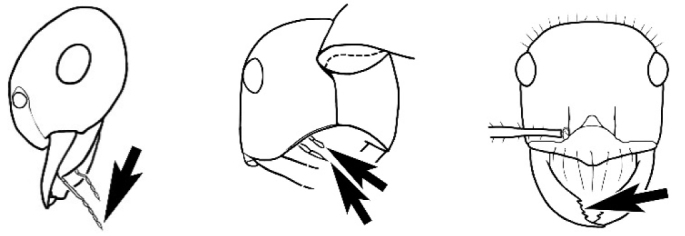


**Key figure '8:**
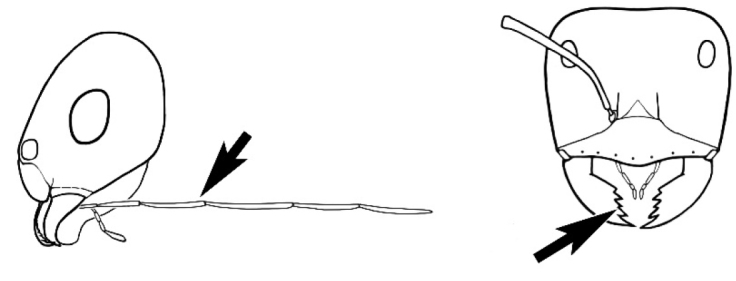


**Key figure 10:**
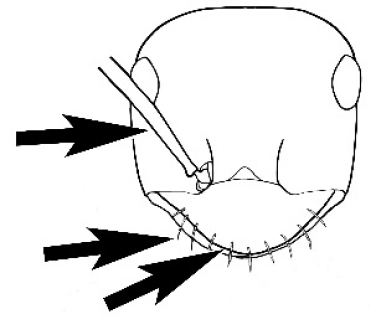


**Key figure '10:**
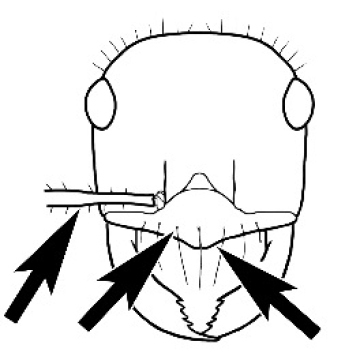


**Key figure 11:**
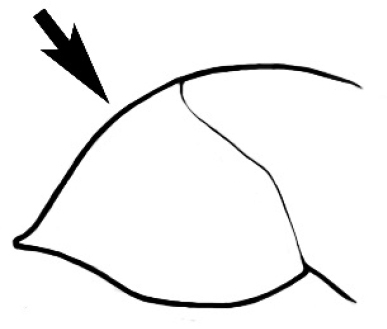


**Key figure '11:**
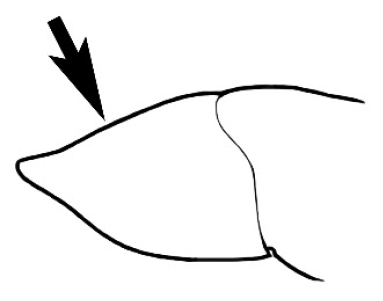


**Key figure 12:**
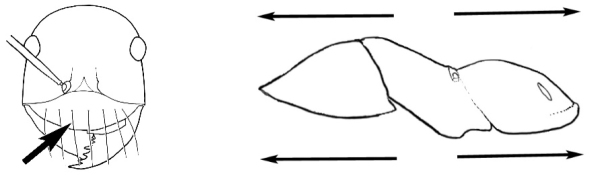


**Key figure 13:**
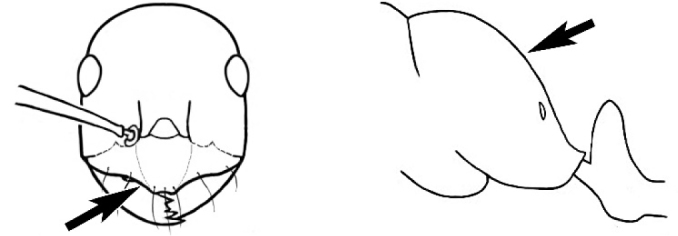


**Key figure '13:**
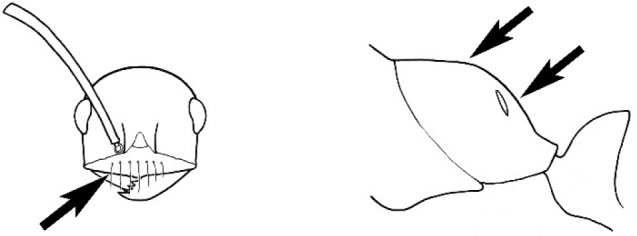


**Key figure 14:**
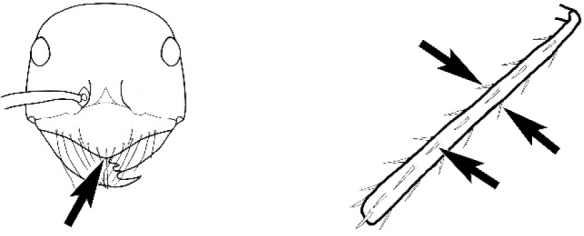


**Key figure '14:**
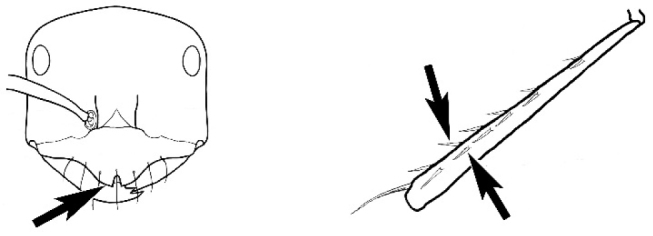


**Key figure 16:**
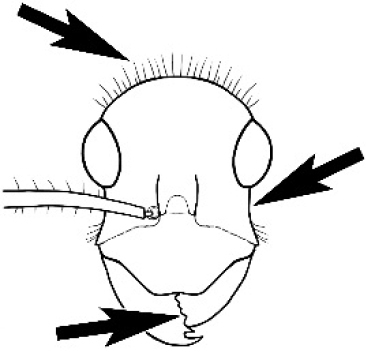


**Key figure 17:**
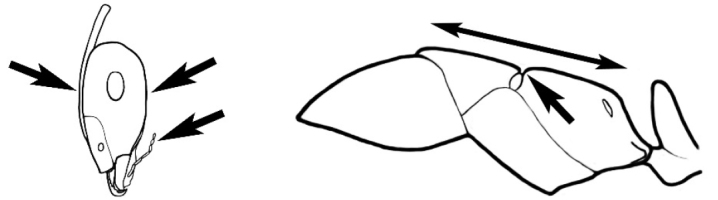


**Key figure 18:**
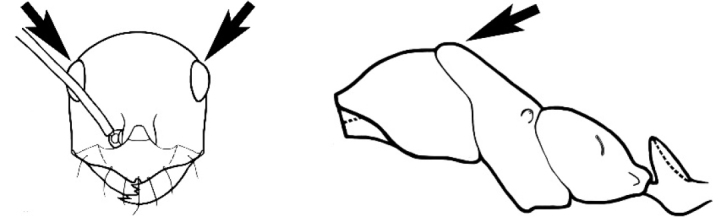


**Key figure '18:**
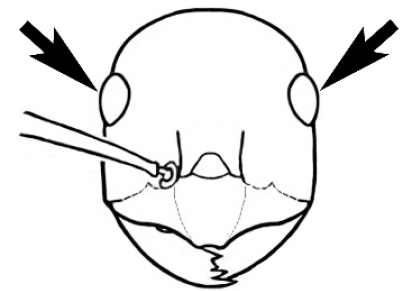


**Key figure 19:**
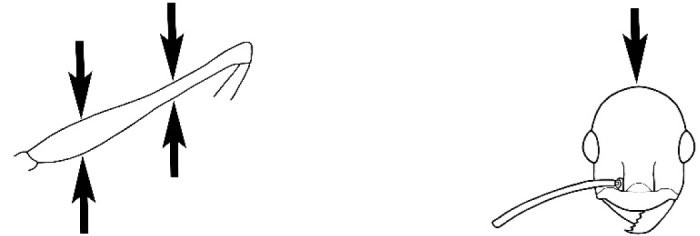


**Key figure '19:**
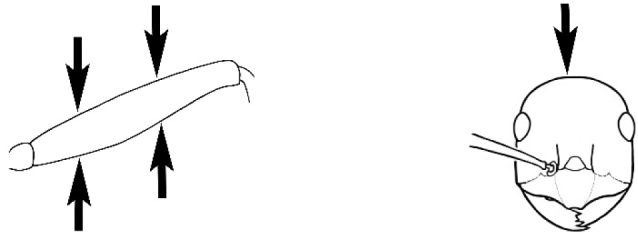


**Key figure 20:**
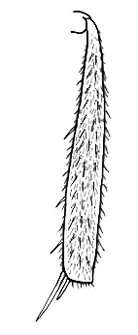


**Key figure '20:**
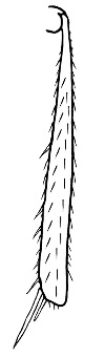


**Key figure 21:**
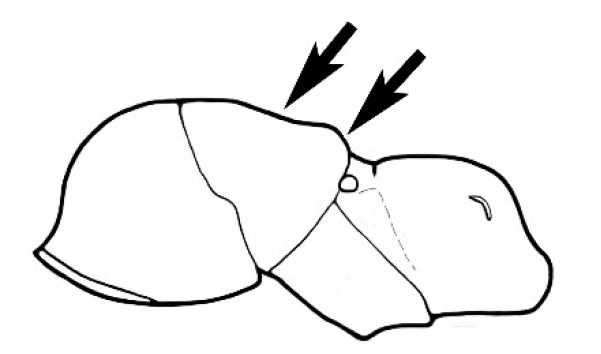


**Key figure '21:**
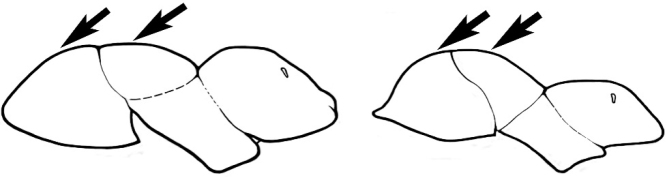


**Key figure 22:**
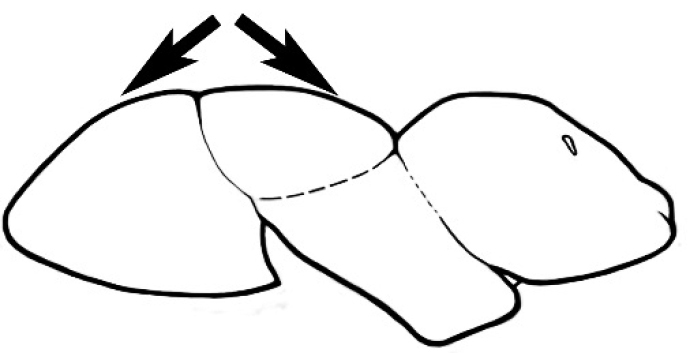


**Key figure '22:**
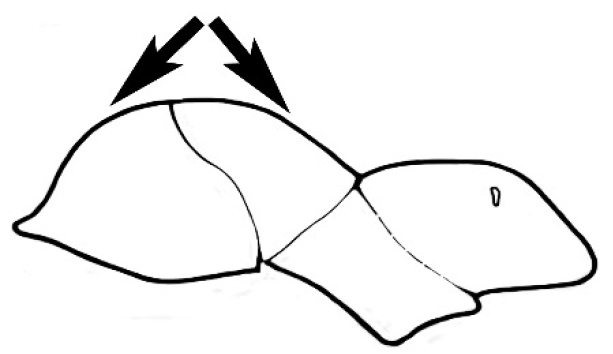


**Key figure 23:**
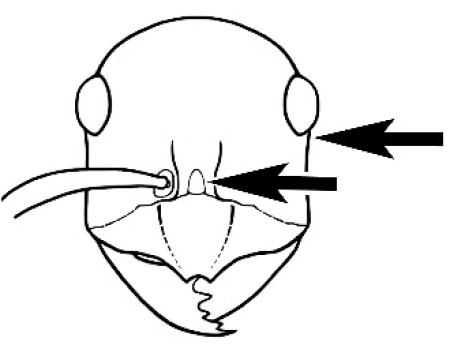


**Key figure '23:**
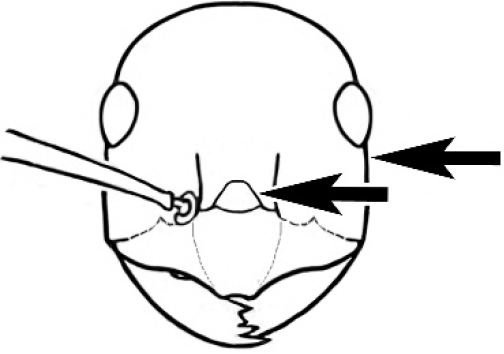


**Key figure 24:**
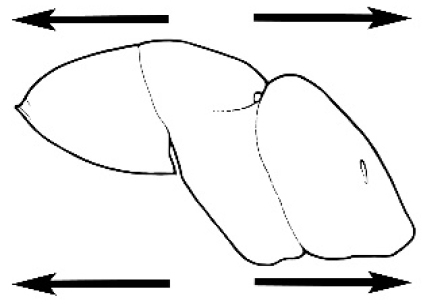


**Key figure '24:**
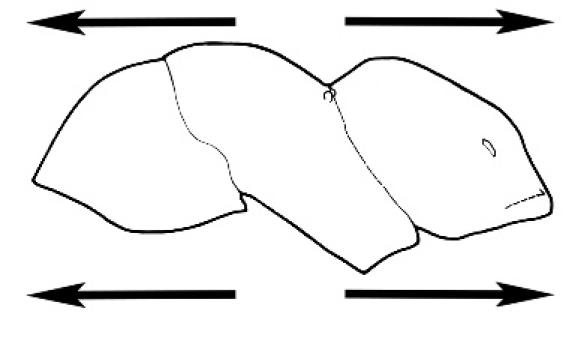


**Key figure 25:**
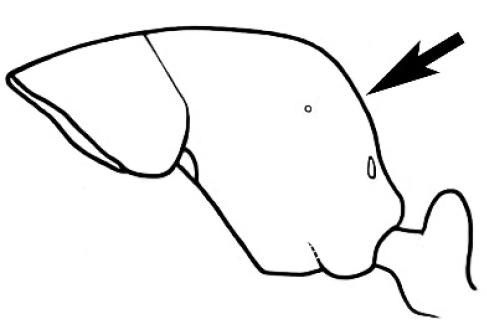


**Key figure '25:**
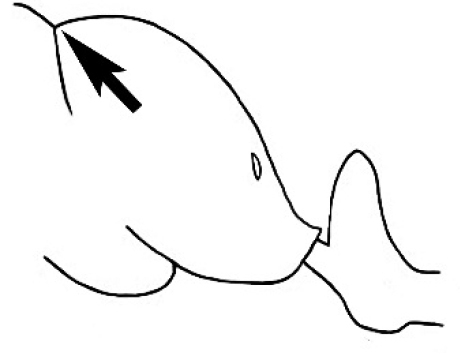


**Key figure 26:**
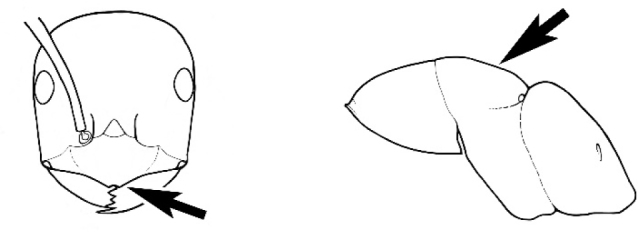


**Key figure '26:**
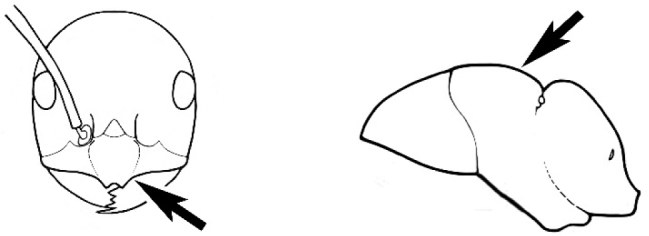


**Key figure 27:**
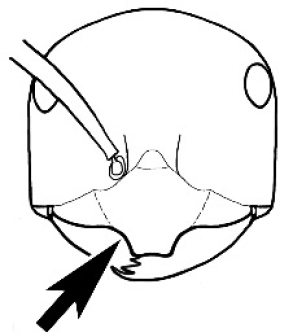


**Key figure '27:**
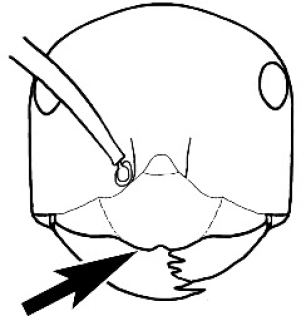


**Key figure 29:**
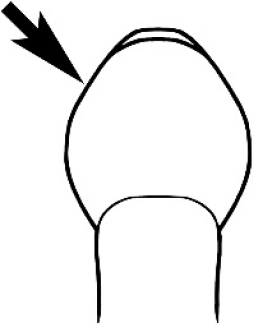


**Key figure '29:**
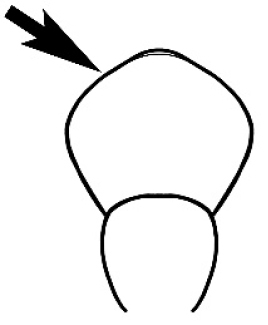


**Key figure 30:**
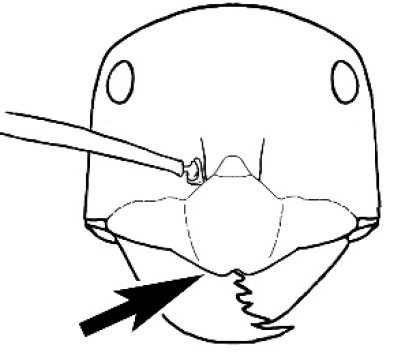


**Key figure '30:**
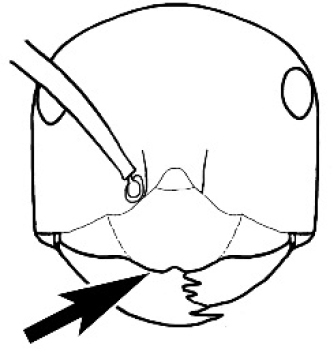


**Key figure 31:**
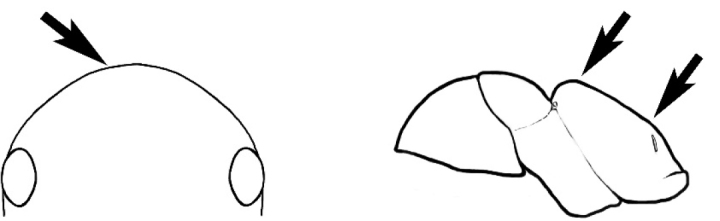


**Key figure '31:**
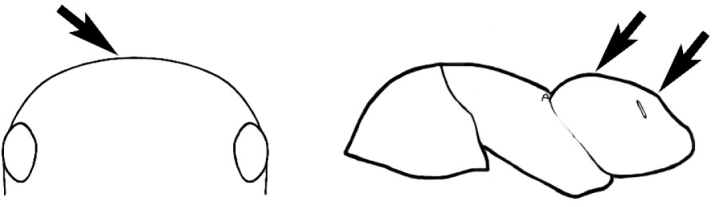


**Key figure 32:**
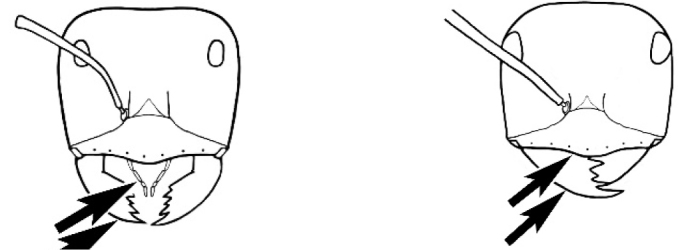


**Key figure 33:**
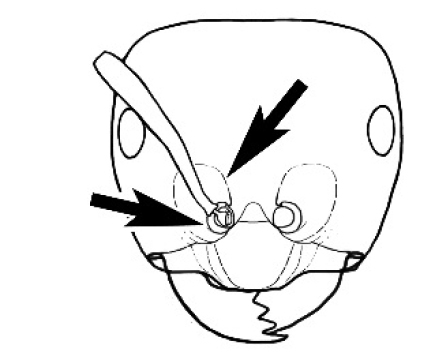


**Key figure '33:**
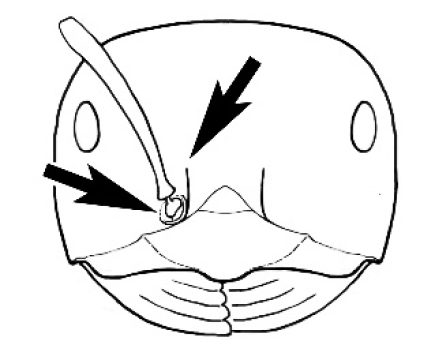


**Key figure 35:**
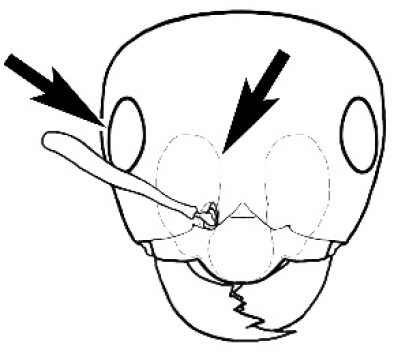


**Key figure '35:**
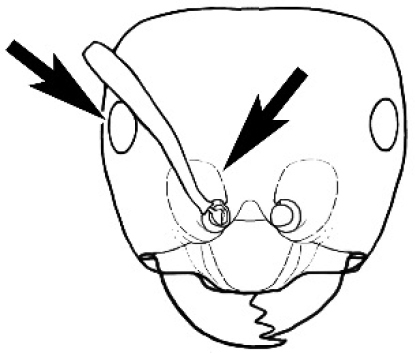


**Key figure 37:**
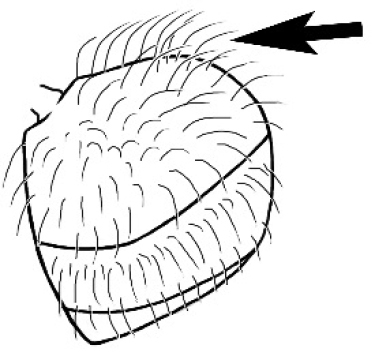


**Key figure '37:**
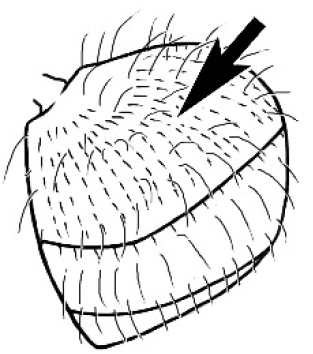


**Key figure 38:**
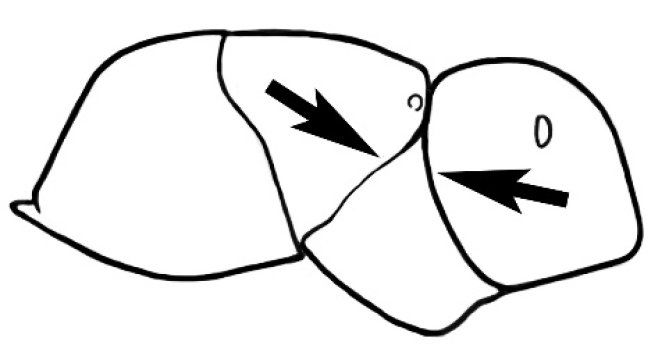


**Key figure 38:**
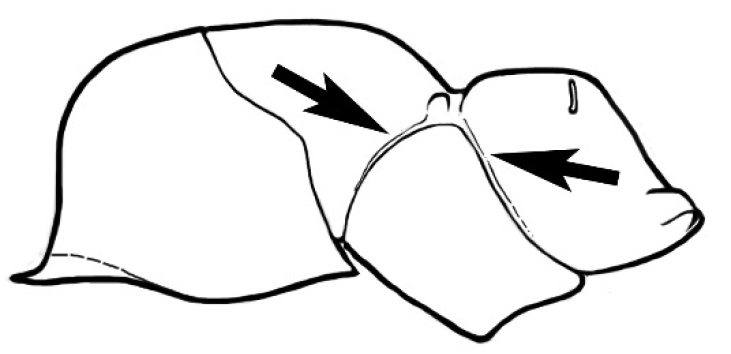


**Key figure 41:**
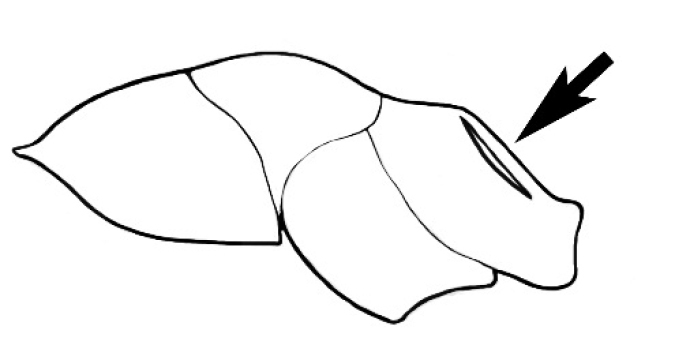


**Key figure 42:**
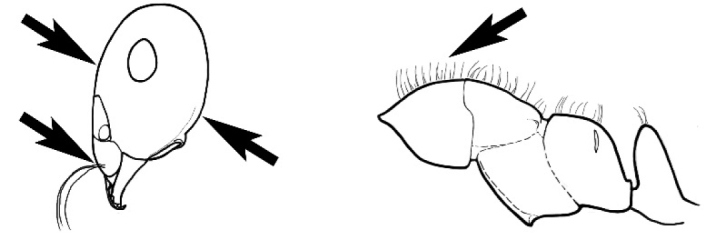


**Key figure 43:**
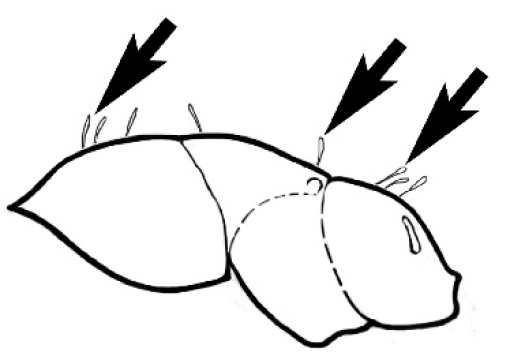


**Key figure 44:**
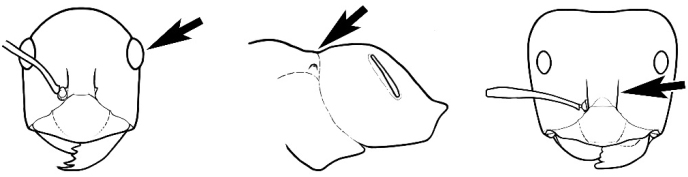


**Key figure '44:**
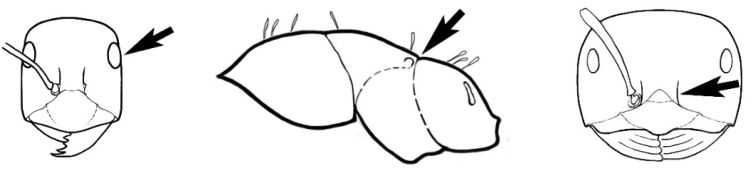


**Key figure 45:**
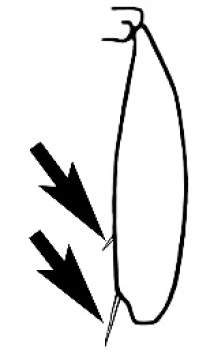


**Key figure '45:**
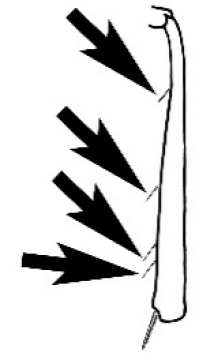


**Key figure 46:**
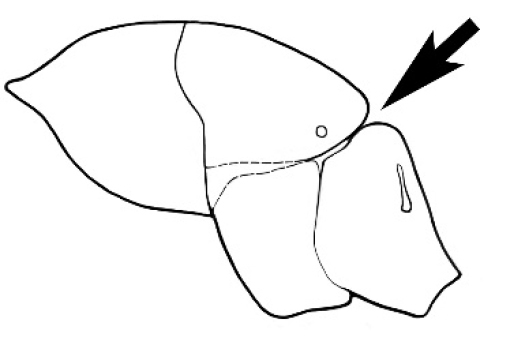


**Key figure '46:**
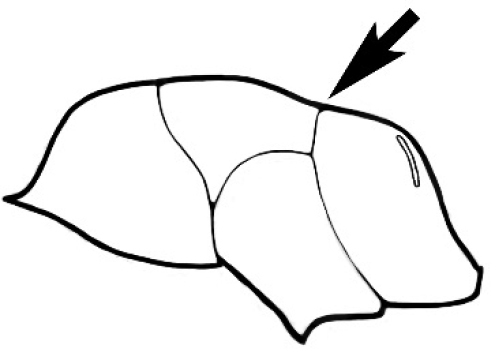


**Key figure 47:**
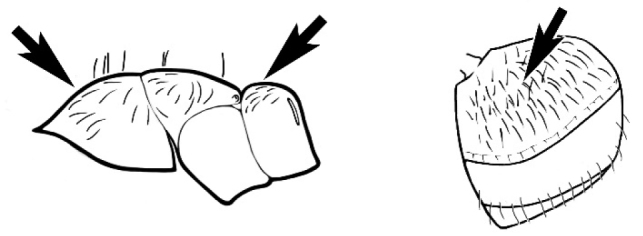


**Key figure 48:**
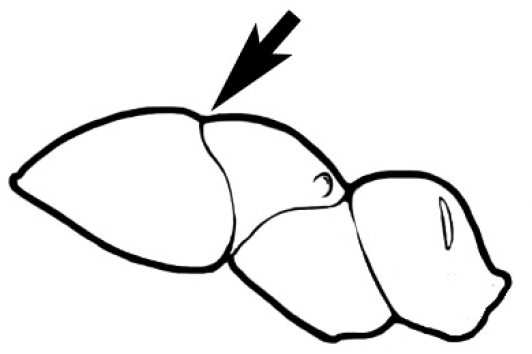


**Key figure 49:**
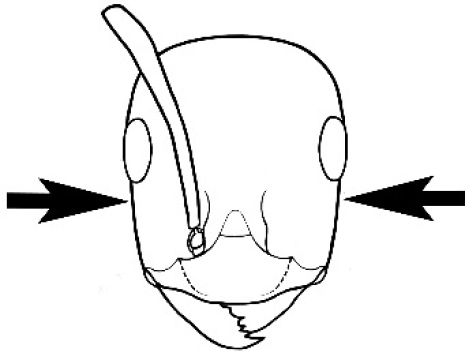


**Key figure 50:**
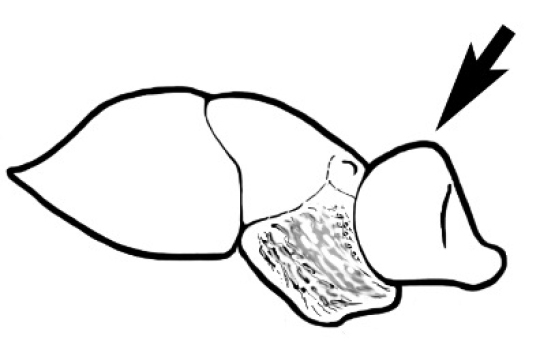


**Key figure 52:**
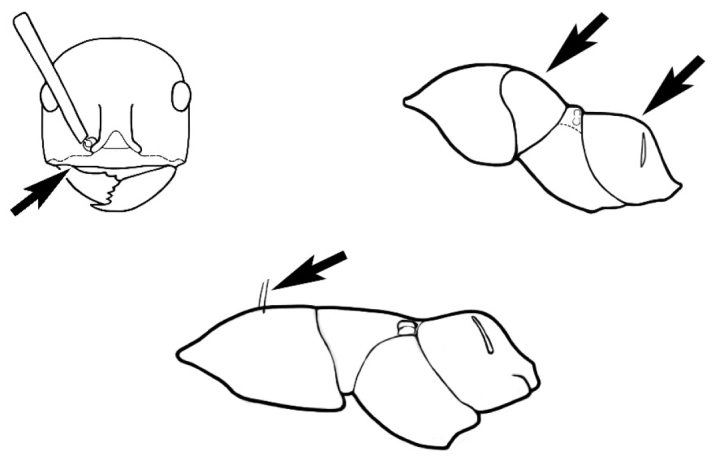


**Key figure '52:**
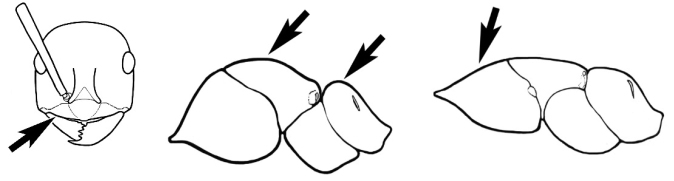


**Key figure 53:**
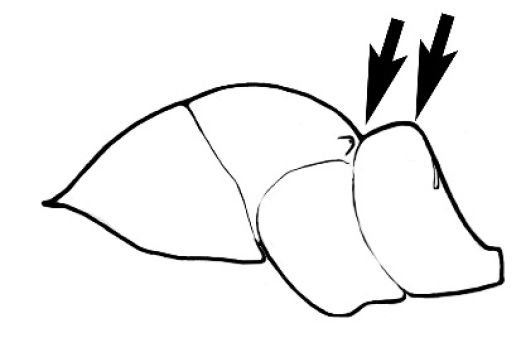


**Key figure '53:**
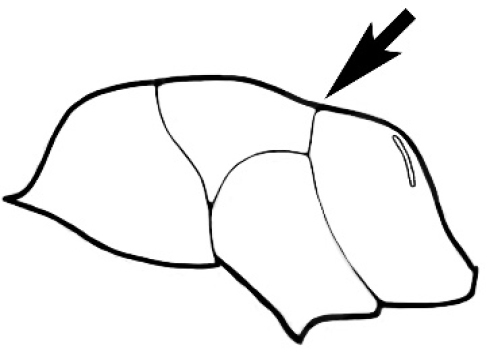


**Key figure 54:**
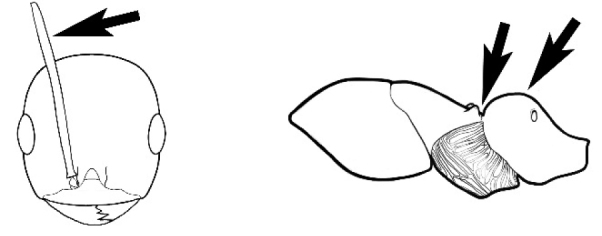


**Key figure 56:**
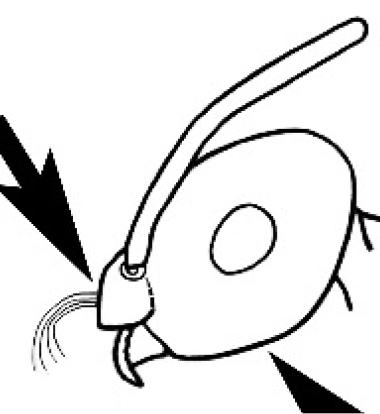


**Key figure '56:**
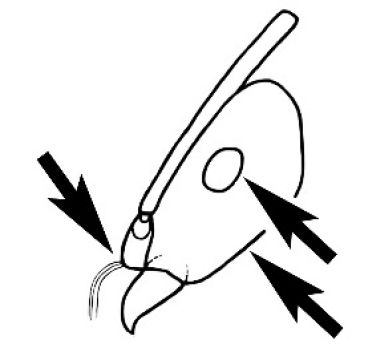


**Key figure 57:**
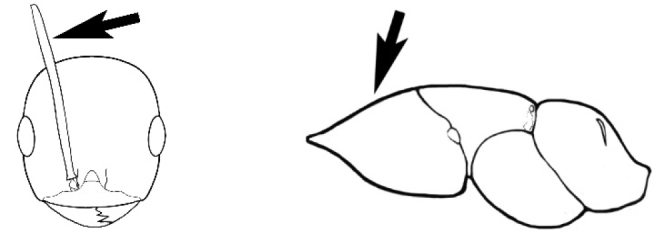


**Key figure '57:**
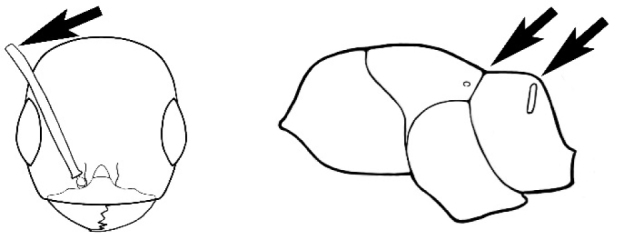


**Key figure 58:**
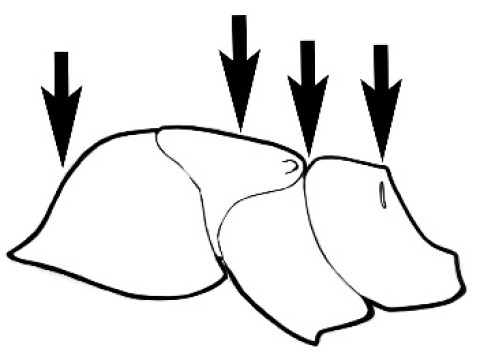


**Key figure '58:**
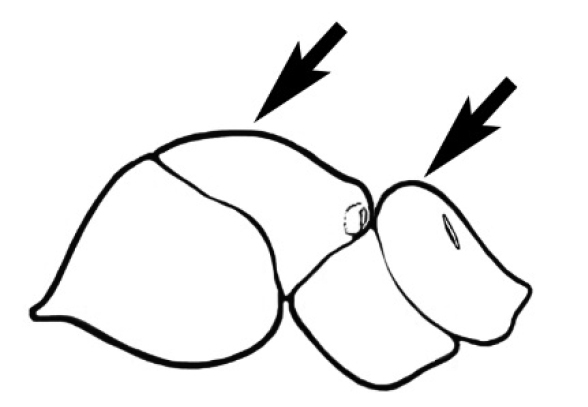


**Key figure 59:**
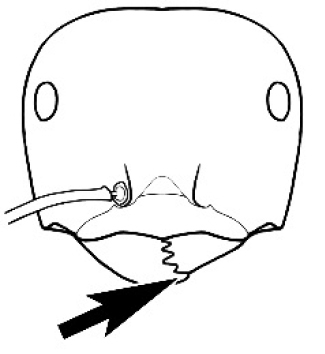


**Key figure '59:**
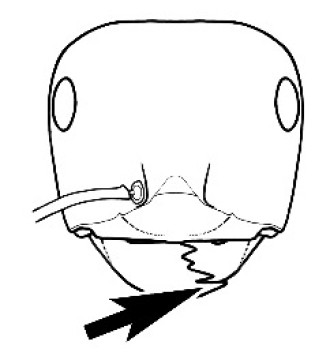


**Key figure 60:**
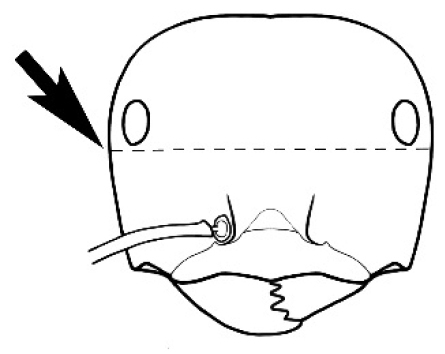


**Key figure '60:**
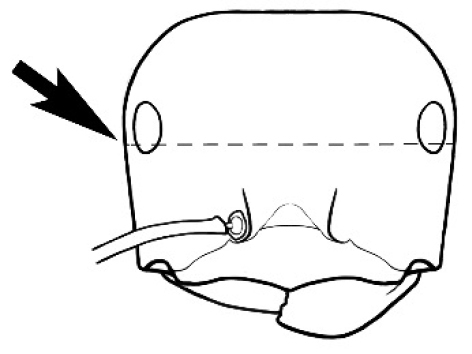


**Key figure 61:**
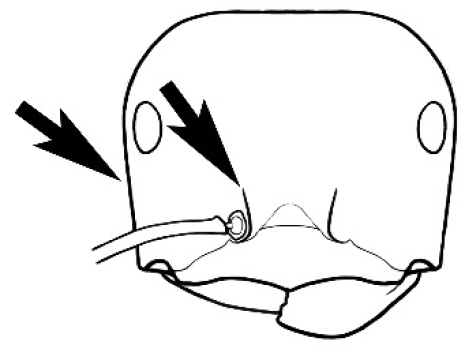


**Key figure '61:**
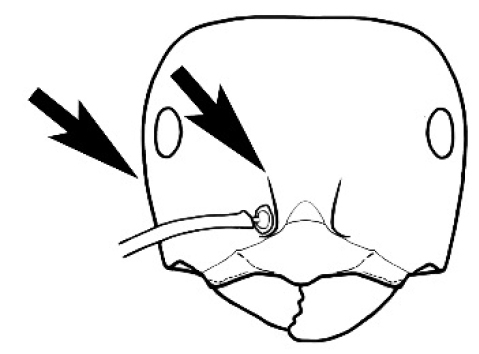


**Key figure 62:**
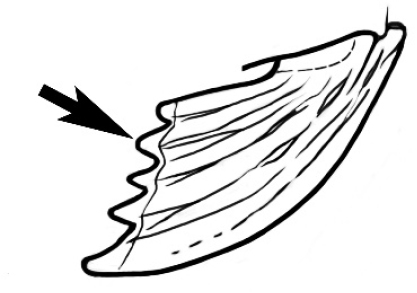


**Key figure '62:**
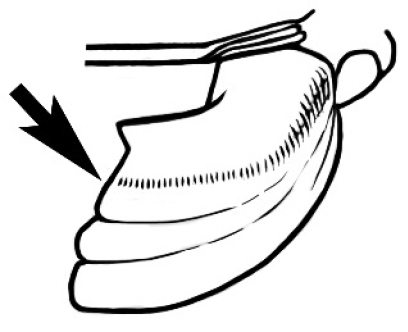


**Key figure 63:**
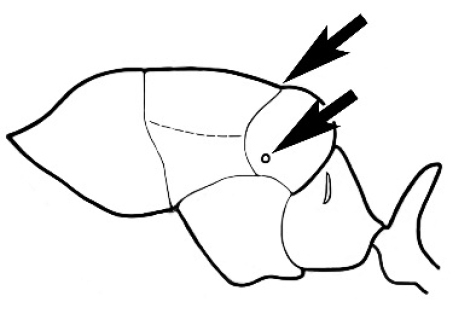


**Key figure '63:**
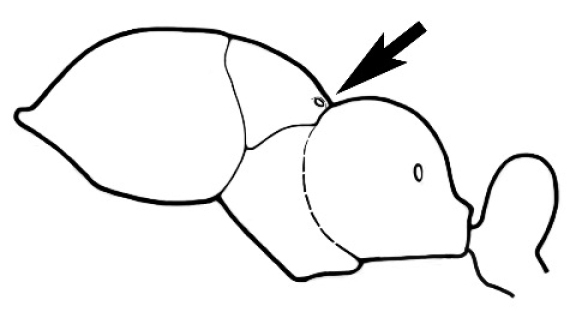


**Key figure 64:**
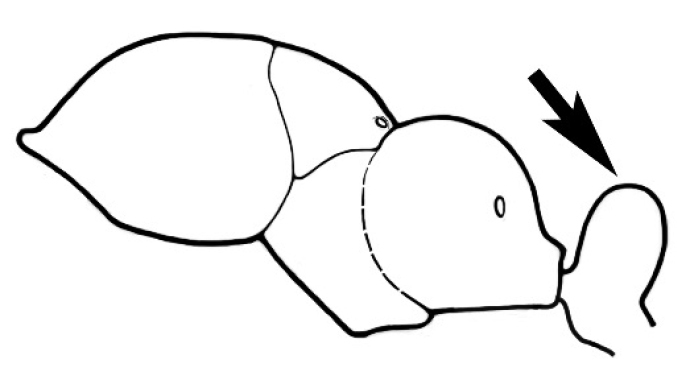


**Key figure 65:**
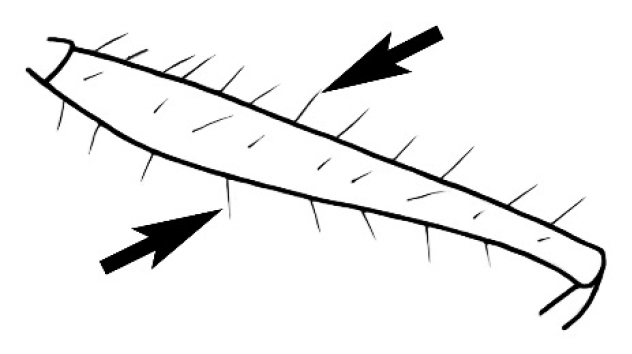


**Key figure '65:**
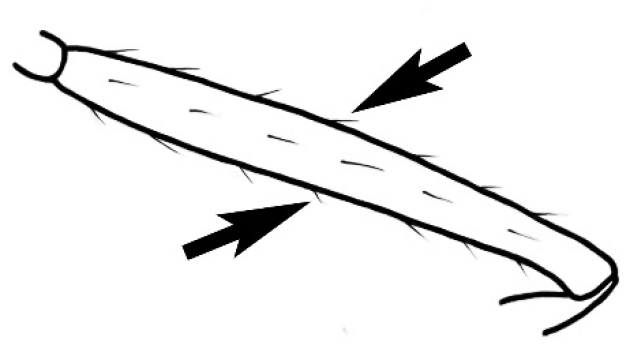


**Key figure 60:**
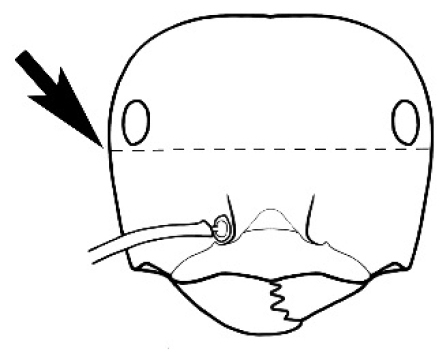


**Key figure 68:**
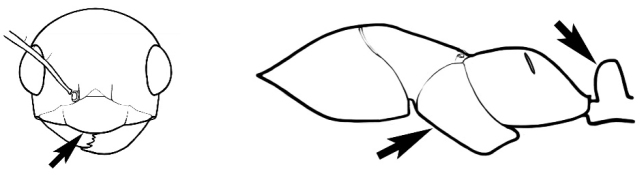


**Key figure '68:**
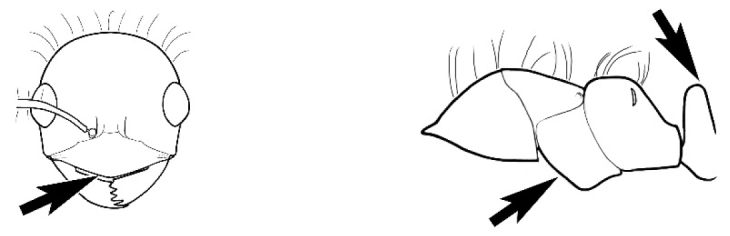


**Key figure 70:**
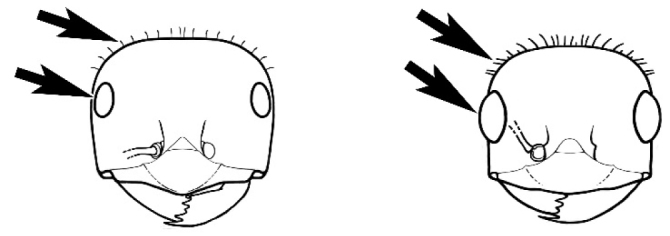


**Key figure '70:**
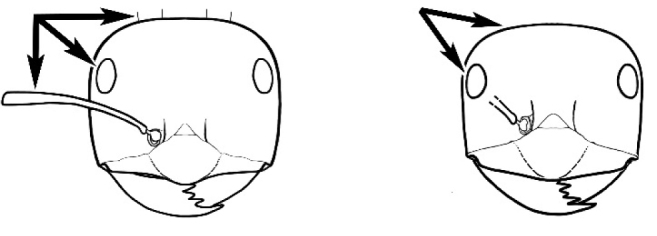


**Key figure 71:**
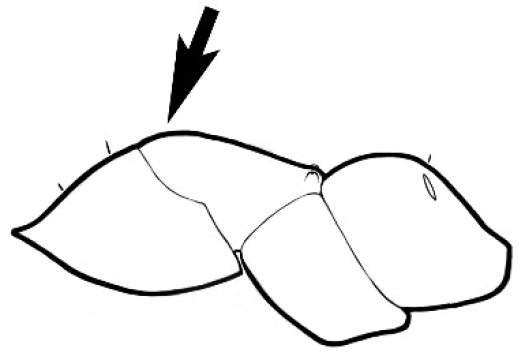


**Key figure '71:**
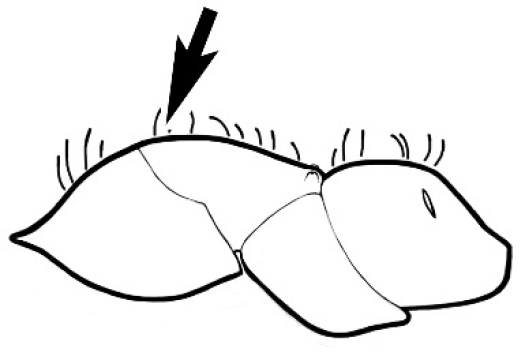


**Key figure 72:**
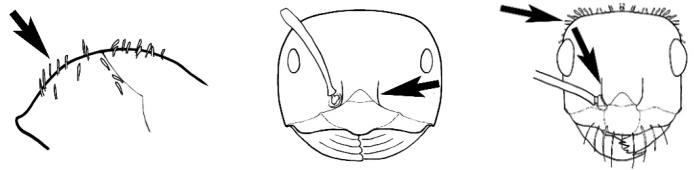


**Key figure '72:**
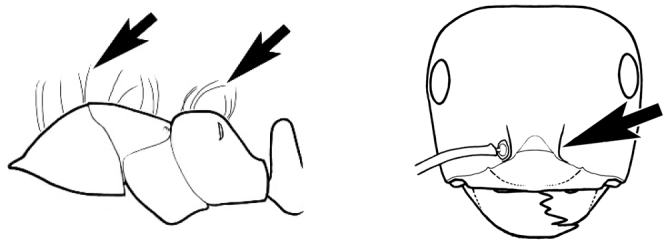


**Key figure 73:**
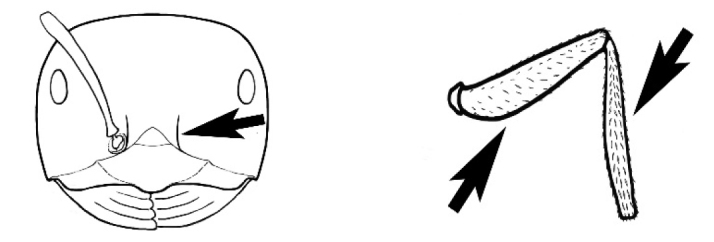


**Key figure '73:**
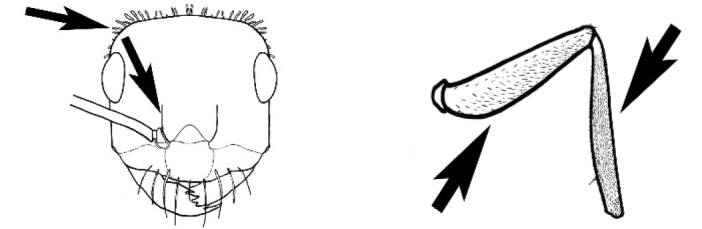


**Key figure 75:**
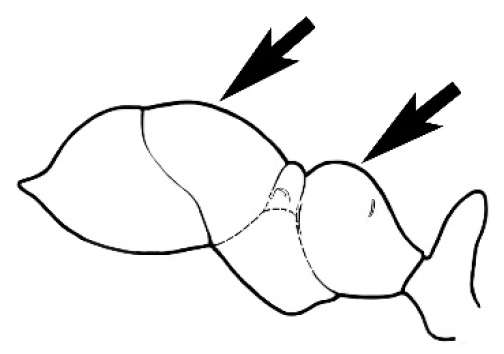


**Key figure '75:**
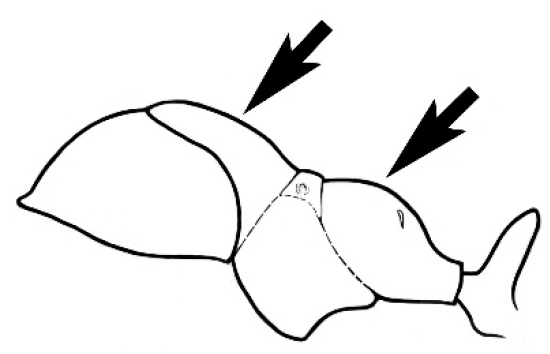


**Key figure 76:**
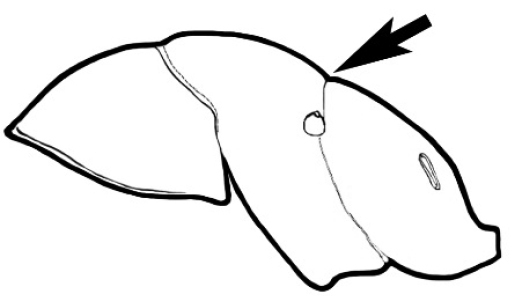


**Key figure '76:**
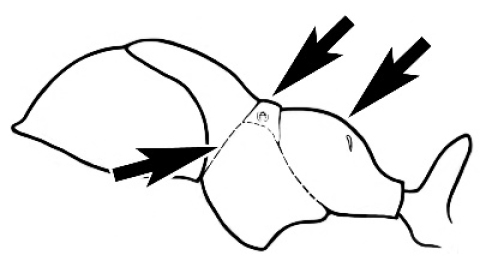


**Key figure 77:**
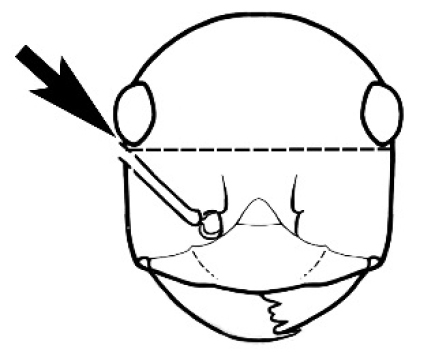


**Key figure '77:**
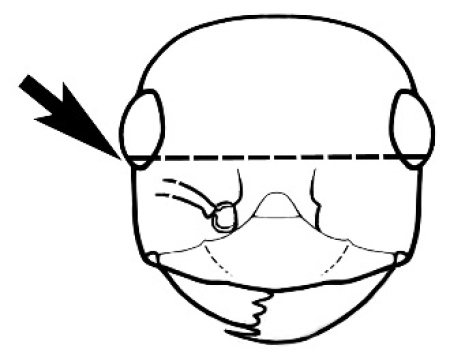


**Key figure 78:**
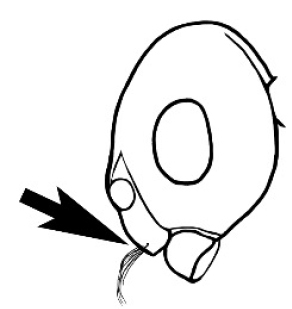


**Key figure '78:**
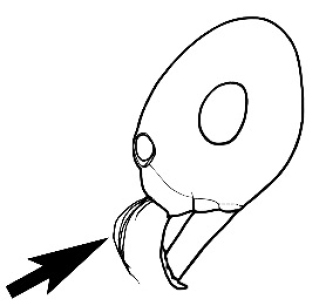


**Key figure 79:**
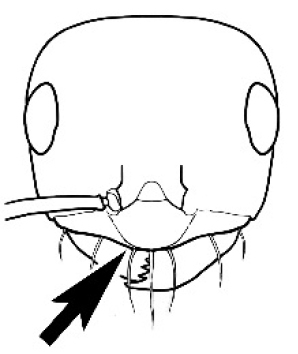


**Key figure 80:**
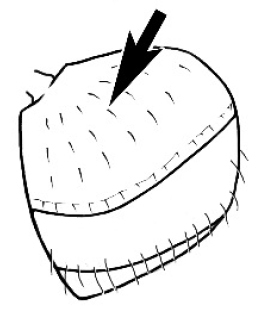


**Key figure '80:**
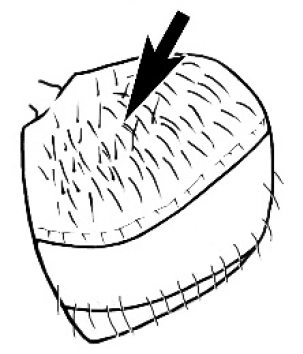


**Key figure 81:**
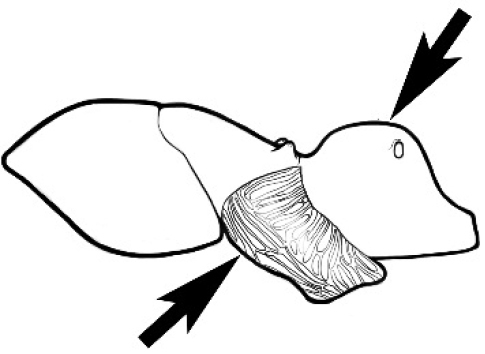


**Key figure 83:**
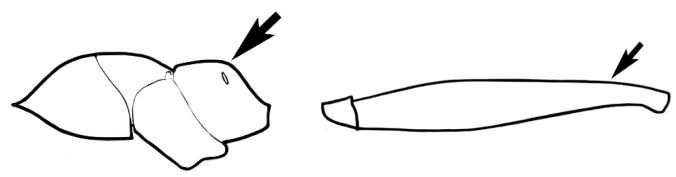


**Key figure '83:**
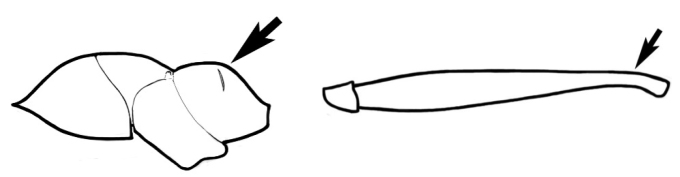


**Key figure 85:**
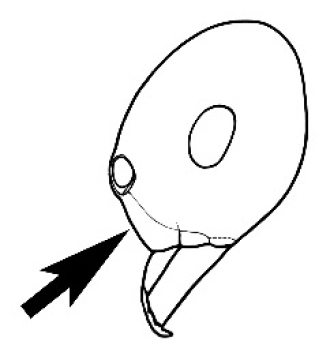


**Key figure '85:**
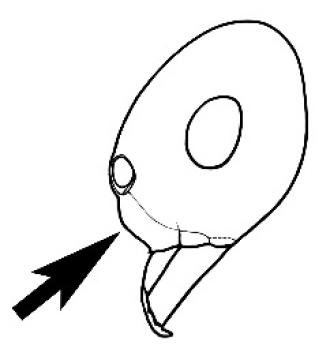


**Key figure 88:**
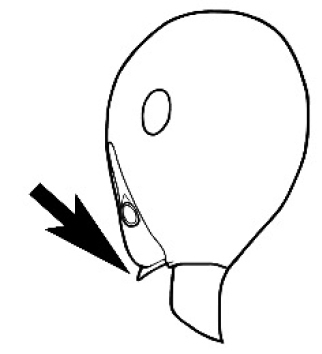


**Key figure '88:**
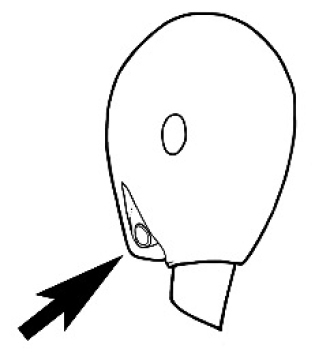


**Key figure 89:**
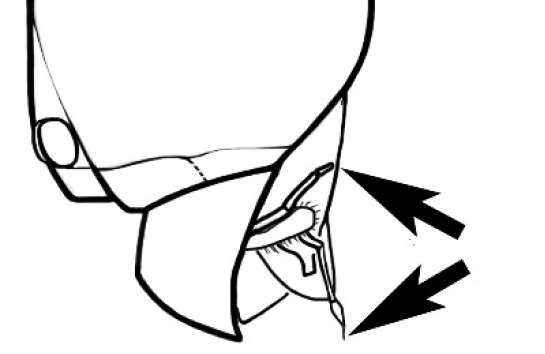


**Key figure 92:**
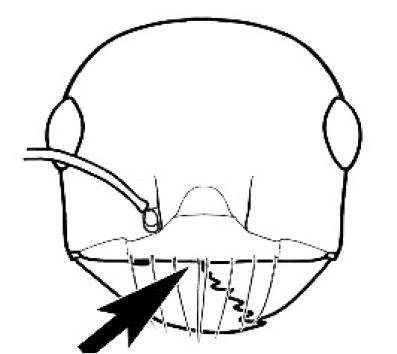


**Key figure 94:**
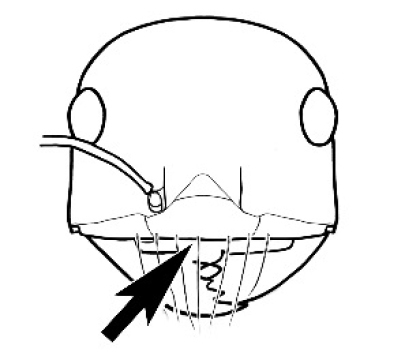


**Key figure 95:**
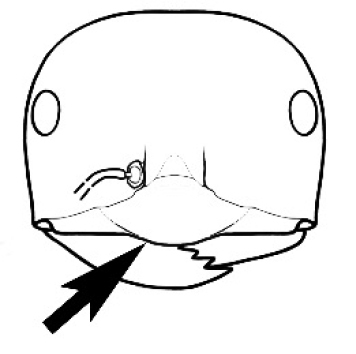


**Key figure 96:**
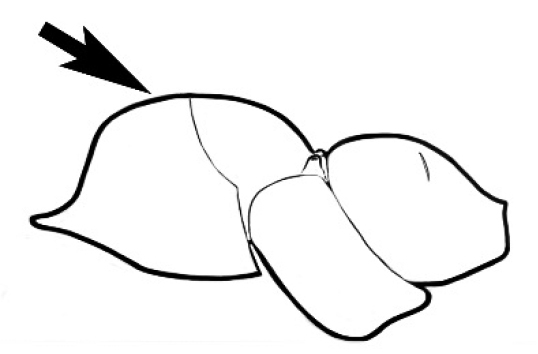


**Key figure '96:**
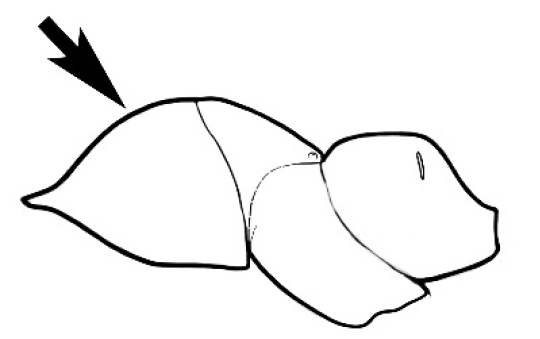


**Key figure 97:**
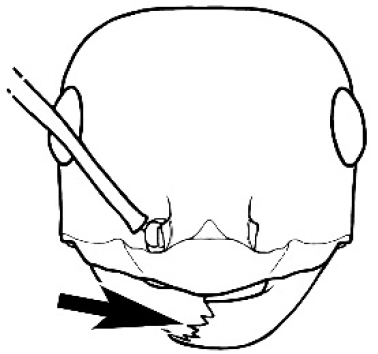


**Key figure 98:**
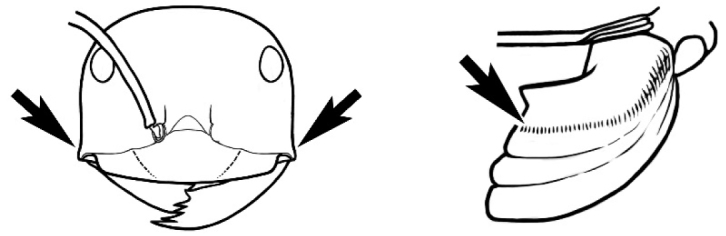


**Key figure '98:**
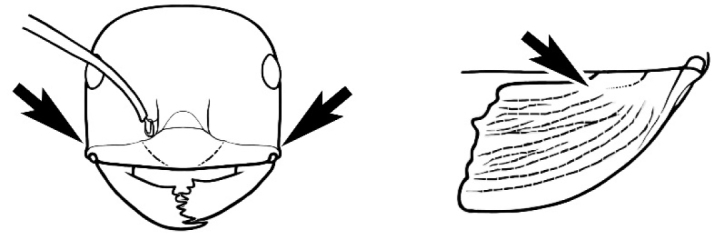


**Key figure 99:**
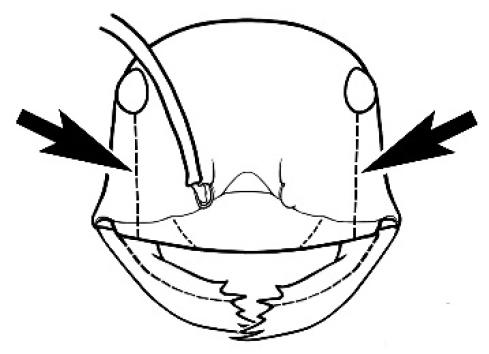


**Key figure '99:**
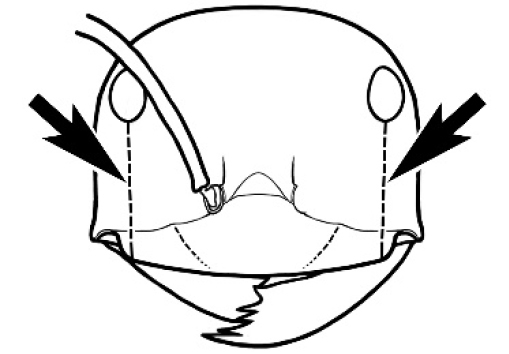


**Key figure 100:**
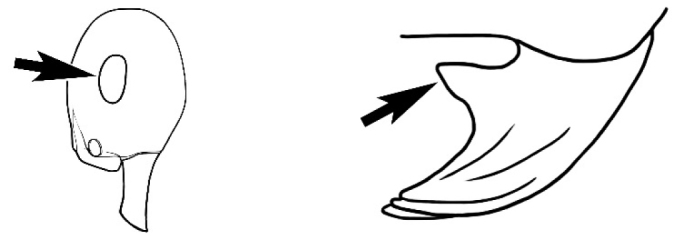


**Key figure '100:**
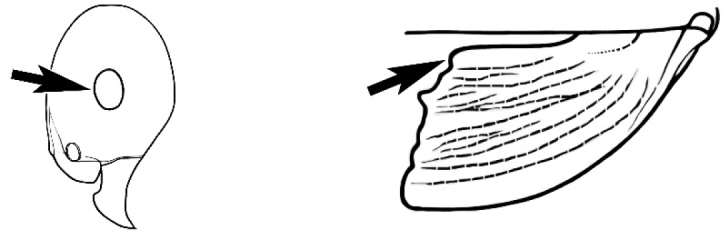


**Descriptions.** (Unless otherwise stated, specimens mentioned under ‘other material examined’ are housed in the Australian National Insect Collection [ANIC]. Curtin Ant Collection material [designated ‘JDM’] is now the property of the Western Australian Museum [WAM]).

### *Melophorus aeneovirens* species-group

This includes what are probably the most morphologically generalised *Melophorus*, with ancient traits. The *M. aeneovirens* species-group is comprised of three complexes (*aenovirens*, *bagoti*, and *nemophilus*). Most members of the group are elongate and gracile and include the predominant *Melophorus* species in cooler, wetter and more heavily forested environments, especially on the east coast, as well as parts of the Torresian zone. While some taxa have broad geographic ranges, others are known from a few collections only and appear to be highly localised. With the exception of a cluster of species around *M. curtus* (i.e., *M. praesens*, *M. rufoniger* and *M. tenuis*) the species are readily recognisable and do not pose great taxonomic difficulties.

Phylogenetically, the genetics of the *M. aeneovirens* species-group are highly congruent with the morphology. Despite only successfully sequencing several species, we obtained a representative species for each of the species complexes and here show that each complex is monophyletic. The *M. aeneovirens* complex is the most basal complex (well supported by the three- and five-gene trees), and the *M. nemophilus* and *M. bagoti* clades are sister complexes, which is reflected in the similarity in their morphological features.

### *Melophorus aeneovirens* complex

Among all the *M. aeneovirens* complexes, this is the most speciose. The members of the complex are grouped together, based on several morphological features (particularly, the appearance of the anterior margin of the clypeus, the mandible, placement of the ammochaetae and the sloping appearance of the propodeum). The three-gene tree (Suppl. material 1) is the most telling when it comes to explaining the phylogeny of this complex. Based on the information derived from the successfully sequenced species, *M. canus* and *M. griseus* are sister species and the most highly derived. Interestingly, *M. praesens* is sister to *M. platyceps* in this gene tree, despite significant morphological differences (flattened body, short labial palps, etc.) that appear to place *M. platyceps* in its own complex.

### Melophorus aeneovirens

Taxon classificationAnimaliaHymenopteraFormicidae

(Lowne)

Formica aeneovirens Lowne 1865: 276 (combination in Cataglyphis by Mayr 1876: 78; in Melophorus by Forel 1902: 488). Types. Syntype major and minor workers [BMNH] without collection data. (The original description by Lowne indicates an unspecified number of major and minor workers were collected near Port Jackson, New South Wales by the author in September-November, 1862.) (examined: photographs of BMNH specimens). Photograph. Melophorus constansSantschi 1928: 475. Type. Syntype major worker, Wattle Glen (as ‘Idattle’ Glen), Victoria [NHMB] (examined: AntWeb image of NHMB specimen CASENT0912336). **Syn. n.**
Melophorus insularis Wheeler 1934: 151. Types. Syntype media and minor workers: Rottnest Island, Western Australia [WAM, BMNH] (examined: WAM specimens Reg. Nos. E-88963/88964, AntWeb images of BMNH specimen BMNH(E)1016286, CASENT0903263). **Syn. n.**
Melophorus iridescens (Emery) Emery 1887: 247. Type. Syntype major worker: Mt Victoria, New South Wales [MSNG] (examined: AntWeb images of MSNG specimen [CASENT0905125]). **Syn. n.**
Melophorus iridescens var. froggatti Forel 1902: 487. Types. Syntype major and minor workers: Sydney, New South Wales [ANIC, MHNG] (Examined: ANIC specimens Database No. ANIC32-053436, AntWeb images of MHNGmajor and minor worker specimens CASENT0909815. CASENT0909816). **Syn. n.**
Melophorus iridescens var. fraudatrix Forel 1915: 87 (footnote).

#### Types.

Syntype media and minor workers: Healesville, Victoria [ANIC, BMNH] (Examined: ANIC specimens, AntWeb images of BMNH specimen BMNH(E)1016284, CASENT0903261). **Syn. n.**

#### Other material examined.

**Australian Capital Territory**: Black Mountain (Barnett, N.J.), Black Mt., Site 1 (Barnett, N.J. [ANIC32-029785]), Canberra (Lowery, B.B.), Capital Hill, Canberra (Lowery, B.B.), Lees Spring (Greaves, T.), Mt. Ainslie (Greaves, T.), Mt. Ainslie (Lowery, B.B.), Mt. Stromlo (Greaves, T.). **New South Wales**: 14 km SW Cootamundra (Lowery, B.B.), 2 mi S Mt Cambewarra (Lowery, B.B.), 8 km S Shooters Hill, 28 km S Oberon (Shattuck, S.O.), Barellan (Lowery, B.B.), Canobolas State Forest (Gush, T.), Condobolin (Lowery, B.B.), Cowan, Sydney (Lowery, B.B.), Fowlers Gap Research Station (Naumann, I.D. & Cardale, J.C.), Lane Cove, Burns Bay, Sydney (Lowery, B.B.), Maroota State Forest (Gush, T.), Memorial Drive, Armidale (Lowery, B.B.), Mudgee (Lowery, B.B.), Myall Lakes National Park (York, A.), near Deniliquin (Zakharov), near Deniliquin (Zakharov [ANIC32-013875]), Newholme Road, near Armidale (Sakurai, Y.), Putty, 50 mi N Windsor (Lowery, B.B.), Pymble (Lowery, B.B.), Royal National Park (Gush, T.), Spring Range Park, 7.5 km NE Hall (Shattuck, S.O. [ANIC32-053450]), The Gib, Bowral (Lowery, B.B.), The Rock Nature Reserve, about 20 mi SE Wagga Wagga (Lowery, B.B.), Tomago (Jackson, G.P. [ANIC32-015239]), West of foothills, Tinderry Mountains (Lowery, B.B.), Whiporie, 55 km S Casino (York, A.). **Queensland**: 2 km N Charleville (Edwards, E.D. & Fisk, J.H.), Gregory Dev. Rd, Sardine Ck (Monteath & Cook), N. Stradbroke Is. Enterprise (QM Party), Toowoomba (Greaves, T.). **South Australia**: 10 km SW Meningie (Greenslade, P.J.M.), 13 km S Quorn, Flinders Ranges (Greenslade, P.J.M.), 15 km ENE Beltana (Greenslade, P.J.M.), 19 mi NW Port Lincoln (Greaves, T.), 22 mi E Eucla (Greaves, T.), 2 km S Ravine des Casoars, Kangaroo Island (Greenslade, P.J.M.), 30 km E Poeppel Corner, Simpson Desert (Greenslade, P.J.M.), 48 km E Minnipa (DEH Surv. 587-KDO 014 [M83]), 4 km S Salt Creek, Coorong (Greenslade, P.J.M.), 7 km W Penneshaw, Kangaroo Island (Greenslade, P.J.M.), Adelaide (Lea), Alligator Creek, South Flinders Range (Greenslade, P.J.M.), Banff, Coorong (Greenslade, P.J.M.), Belair (Greenslade, P.J.M.), Belair (Greenslade, P.J.M.), Bridgewater (Greenslade, P.J.M.), Bridgewater (Baker, G.F.), Calca (Lowery, B.B.), Cambrai (Greenslade, P.J.M.), Ceduna (Lowery, B.B.), Ceduna (Casperson, K.), Cleland, Mt. Lofty Ranges (Yeatman, E.), Cockatoo, Coorong (Greenslade, P.J.M.), Cockatoo, Coorong (Greenslade, P.J.M.), Glen Osmond (Greenslade, P.J.M.), Koonamore (Greenslade, P.J.M.), Kuitpo (Greenslade, P.J.M.), Mambray Creek (Greenslade, P.J.M.), Messert Res., Coorong (Greenslade, P.J.M.), Napperby, Flinders Ranges (Greenslade, P.J.M.), North of Breakneck River, Kangaroo Island (Greenslade, P.J.M.), North of Breakneck River, Kangaroo Island (Greenslade, P.J.M.), Oraparinna, Flinders Ranges (Greenslade, P.J.M.), Para Wirra (Greenslade, P.J.M.), Scrubby Gully, Sevenhill (Lowery, B.B.), Sevenhill (Lowery, B.B.), Sevenhill (Lowery, B.B.), Sevenhill (Lowery, B.B.), Sevenhill (Lowery, B.B.), Sevenhill (Lowery, B.B.), Victor Harbour (Greenslade, P.J.M.), West Bay, Kangaroo Island (Greenslade, P.J.M.). **Victoria**: Anglesea (Andersen, A.N. [JDM32-001829]), 15 km WNW Yaapeet (Andersen, A.N.), Bendigo (McAreavey, J.), Big Desert (Andersen, A.N.), Bull Creek, Glenaladale National Park (Andersen, A.N.), Heathcote, near Bendigo, Mt. Ida (Lowery, B.B.), Kew (McAreavey, J.), Wangarratta (Bruce, W.A.), Watsonia (Lowery, B.B.), Wattle Park, Melbourne, Werribee (Lowery, B.B.). **Western Australia**: 14 km S of Billabong R/D House, Nth/West Coastal Hwy (Heterick, B.E. [JDM32-001495]), 24 mi ESE Broome (McInnes & Dowse [ANIC32-900075]), 49 mi WSW Ravensthorpe (Greaves, T.), 4 km NW of Gleneagle (Heterick, B.E. & Jacobs, M. [JDM32-001494]), 8 mi E Northcliffe (Taylor, R.W.), Albany (Greaves, T.), Alcoa (Wallace, J.), Bartons Mill (Majer, J.D.), Bartons Mill (Majer, J.D. [JDM32-001521]), Black Swan Mine (Langlands, P. & Osbourne, J. [JDM32-001496]), Bunbury (Lowery, B.B.), Canning Vale (Knowles, D.G. [JDM32-001498]), Coalmine Beach, Nornalup National Park, Walpole (Lawrence, J. &N.), Coalmine Rd. (collector unknown [JDM32-001830]), Denmark (Lowery, B.B.), Dwellingup (Majer, J.D.), Dwellingup (Majer, J.D. [JDM32-001500]), Eneabba (Fox, J. [JDM32-001533]), Goora Hill [Gora Hill]] (Greaves, T. [ANIC32-052115]), Gora Hill (Greaves, T.), Harvey (Mercovich, C.), Jandakot (collector unknown [JDM32-001530]), Jandakot (collector unknown [JDM32-001529]), Jurien (collector unknown [JDM32-001532]), Kalamunda (Greaves, T.), Kalbarri National Park (Ward, P.S.), Kings Park, Perth (Lowery, B.B.), Kojonup (Majer, J.D. [JDM32-001512]), Manjimup (Majer, J.D. [JDM32-001509]), Melaleuca Grove, Thomas River (Greaves, T.), Mordalup (Majer, J.D. [JDM32-001531]), Mt. Clare, 4 mi W Walpole (Taylor, R.W.), Mt. Ragged, west foot (Taylor, R.W.), Mulga, NE Goldfields (Pringle, H.J.R. [ANIC32-029627]), Mundaring (Clark, J.), nr. Wungong Dam (Heterick, B.E. [JDM32-001493]), Perth (Clark, J.), Reabold Hill (Majer, J.D. [JDM32-001534]), Red Hill (Heterick, B.E. [JDM32-001492]), Rottnest Is. (collector unknown [JDM32-001536]), Rottnest Island (Lea), Talyuberlup Picnic Area, Stirling Range National Park (Lawrence, J. &N.), Telegraph Hill, Esperance (Lowery, B.B.), Without Locality (Majer, J.D. [JDM32-001535]).

#### Diagnosis.

*Melophorus aeneovirens* is a member of the *M. aeneovirens* species-group (in full-face view, the anterior clypeal margin convex, apron-like and covering whole or part of the retracted mandible, except in *M. nemophilus*, the medial clypeal sector often produced so that it is protrusive when seen in profile; the psammophore frequently with coarse and well-separated ammochaetae, these always placed on or just above anterior margin; in profile, the propodeum elongate and oblique or broadly rounded), and the *M. aeneovirens* species-complex (in full-face view, psammophore ranged along or just above anterior margin of clypeus and following the curve of the margin; anterior margin of clypeus broadly medially produced, and often with central notch that may be deeply impressed, but is never acuminate at its midpoint; metatibia with maximum of two rows of preapical spines). In *M. aeneovirens* the tibiae possess stout, socketed, appressed to subdecumbent setae only, with fine, appressed pubescence lacking. In profile, the minor worker mesosoma is compact and is arcuate in outline. *Melophorus aeneovirens* can be distinguished from similar species by having, in profile, the clypeus distinctly recurved at about midpoint and produced over the mandible as a small ledge; in full-face view, the anterior margin of the major and minor worker clypeus forms a broadly convex, sometimes crenulate curve that does not protrude over apical the curve of mandible.

#### Minor worker description.

**Head.** Head square; posterior margin of head strongly convex; frons shining with superficial shagreenation or microreticulation only; pilosity of frons a mixture of a few well-spaced, erect setae interspersed with appressed setae only. Eye moderate (eye length 0.20–0.49 length of side of head capsule); in full-face view, eyes set above midpoint of head capsule; in profile, eye set anteriad of midline of head capsule; eyes elliptical or slightly reniform. In full-face view, frontal carinae concave; frontal lobes straight in front of antennal insertion. Anteromedial clypeal margin narrowly convex and protruding, clypeal margin entire or very weakly indented, or narrowly convex and protruding anteromedially, clypeal midpoint distinctly notched; clypeal psammophore set at or above midpoint of clypeus; palp formula 6,4. Five to six mandibular teeth in minor worker; mandibles triangular, weakly incurved; third mandibular tooth distinctly shorter than apical tooth and teeth numbers two and four; masticatory margin of mandibles approximately vertical or weakly oblique. **Mesosoma.** Integument of pronotum, mesonotum and mesopleuron moderately shining and shagreenate throughout; anterior mesosoma in profile broadly convex; erect pronotal setae absent; in profile, metanotal groove deep, ‘V’-shaped; propodeum shining and shagreenate; propodeum smoothly rounded or with indistinct angle; propodeal dorsum and declivity confluent; erect propodeal setae always absent; appressed propodeal setulae long and closely aligned, creating pubescence; propodeal spiracle situated at least twice its width from the declivitous face of propodeum, and shorter (length < 0.50 × height of propodeum). **Petiole.** In profile, petiolar node subcuboidal, vertex bluntly rounded; in full-face view, shape of petiolar node generally rounded with median indentation or hollow; node shining and distinctly shagreenate-microreticulate. **Gaster.** Gaster weakly shining with indistinct shagreenation; pilosity of first gastral tergite consisting of long, closely aligned, whitish, appressed setae, with erect setae (present in at least some workers) confined to margin of sclerite. **General characters.** Colour of foreparts reddish-brown through red to dark crimson, legs dark brown, gaster blackish-brown to black.

#### Major worker description.

**Head.** Head quadrate (i.e., heart-shaped); posterior margin of head strongly concave; cuticle of frons shining with superficial shagreenation or microreticulation only; frons consisting exclusively or almost exclusively of well-spaced, appressed setae only (small, erect setae, if present, usually confined to ocular triangle or posterior margin of head). Eye moderate (eye length 0.20–0.49 length of head capsule); in full-face view, eyes set above midpoint of head capsule; in profile, eye set anteriad of midline of head capsule; eyes elliptical. In full-face view, frontal carinae concave; frontal lobes straight in front of antennal insertion. Anterior clypeal margin narrowly convex and protruding anteromedially, clypeal margin entire or weakly indented; clypeal psammophore set at or just above anterior clypeal margin; palp formula 6,4. Five to six mandibular teeth in major worker; mandibles triangular, weakly incurved; third mandibular tooth distinctly shorter than apical tooth and teeth numbers two and four; masticatory margin of mandibles approximately aligned vertically or weakly oblique. **Mesosoma.** Integument of pronotum, mesonotum and mesopleuron moderately shining and shagreenate throughout; anterior mesosoma in profile broadly convex; erect pronotal setae short (i.e., shorter than length of eye) and unmodified; in profile, metanotal groove shallow, broadly V- or U-shaped; propodeum shining and shagreenate; propodeum smoothly rounded or with indistinct angle; propodeal dorsum and declivity confluent; erect propodeal setae present and abundant (at least a dozen); appressed propodeal setae long and closely aligned, creating pubescence; propodeal spiracle situated at least twice its width from the declivitous face of propodeum, and shorter (length less than 0.50 × height of propodeum). **Petiole.** In profile, petiolar node squamiform; in full-face view, shape of petiolar node uniformly rounded; node shining and faintly shagreenate-microreticulate. **Gaster.** Gaster weakly shining with indistinct shagreenation; pilosity of first gastral tergite consisting of well-spaced short, inconspicuous, appressed setae only, erect setae always absent. **General characters.** Colour of foreparts reddish-brown through red to dark crimson, legs dark brown, gaster blackish-brown to black.

#### Measurements.

Worker (n = 6): CI 91–115; EI 14–28; EL 0.19–0.34; HL 0.73–2.12; HW 0.67–2.44; ML 1.15–2.47; MTL 0.60–1.39; PpH 0.13–0.25; PpL 0.61–1.15; SI 67–142; SL 0.95–1.62.

#### Comments.

*Melophorus aeneovirens* (Lowne) was the first *Melophorus* species to be described, and was originally placed in the genus *Formica*. This is among the most common of all the *Melophorus* species, and is found in all mainland states. This ant is particularly common on the eastern seaboard, but does not appear to occur in Tasmania. Regrettably, despite its abundance in many areas, no specimens were available for sequencing. In general, there is some morphological variation, particularly among minor workers that could be examined using genetic markers (some of this variation is mentioned by Wheeler (1934) in his description of *M. insularis*). Colour varies from orange to blackish-brown among minor workers and the degree of compression of the dorsum of the propodeum also varies: in some cases this sclerite is rather convex in outline but it can be straight or even fractionally concave. The metanotal groove varies from deeply to weakly incised. Minor workers among different populations can also vary markedly in size and sculpture. However, all minor workers share the recurved appearance of the clypeus when this is seen in profile, an asymmetrical vertex (like *M. praesens* and *M. rufoniger*) and an anterior clypeal margin that is never strongly produced so as to form a ledge (this feature serves to separate *M. aeneovirens* from nearly all other members of the *M. aeneovirens* species-group). Because of its ubiquity, *M. aeneovirens* has attracted a lot of taxonomic attention, and *M. constans*, *M. insularis*, *iridescens*, *M. iridescens froggatti*, and *M. iridescens fraudatrix* all reveal consistency as regards the diagnostic characters determined for the species in this revision and become junior synonyms in this work. Furthermore, *M. aeneovirens* from Port Jackson and *M. iridescens froggatti* from Sydney come from the same population. The major worker in each case has an orange head and dark brown body and blackish gaster.

The distribution of this taxon is predominantly southern and most collections have been taken in mesic, coastal localities, with far fewer records from arid and semi-arid areas. This is the common *Melophorus* species taken in well-watered forests. Lowne (1865) mentions this ant formed small nests with an inconspicuous opening, and nests in Port Jackson NSW (the type locality) were usually concealed under leaves or stones. The habits of *M. aeneovirens* may well be mentioned in the literature without the species being identified, but little has been recorded under that name or under the synonymic names. Since this species occurs in a very wide range of habitats, including grassy lawns of suburban residences, the most logical inference is that it is a generalized scavenger.

### Melophorus attenuipes

Taxon classificationAnimaliaHymenopteraFormicidae

Heterick, Castalanelli & Shattuck sp. n.

http://zoobank.org/FAA8F3D9-1A6F-4F8D-98B2-6C6853D8C7F8

#### Types.

Holotype minor worker (bottom ant) from 1 km N Mulga Downs OC 22°19'33"S, 118°58'47"E, Western Australia, 24 May 2004-4 May 2005, CALM Pilbara Survey, Site RHNW05, ethylene glycerol pitfalls [JDM32-002000] (WAM). Paratypes: major worker, on same pin and with same details as holotype (WAM); minor worker, Zuytdorp 27°15'S, 114°04'E, Western Australia, 26 September-19 October 1994, A. Sampi/L. Cresswell, ZU4: Wet pits WAM/CALM survey Carnarvon Basin [JDM32-001998] (ANIC); minor worker, Zuytdorp 27°15'S, 114°04'E, Western Australia, 26 September-19 October 1994, A. Sampi/L. Cresswell, ZU4: Dry pits WAM/CALM survey Carnarvon Basin [JDM32-001999] (MCZ).

#### Diagnosis.

*Melophorus attenuipes* is a member of the *M. aeneovirens* species-group (in full-face view, the anterior clypeal margin convex, apron-like and covering whole or part of the retracted mandible, except in *M. nemophilus*, the medial clypeal sector often produced so that it is protrusive when seen in profile; the psammophore frequently with coarse and well-separated ammochaetae, these always placed on or just above anterior margin; in profile, the propodeum elongate and oblique or broadly rounded), and the *M. aeneovirens* species-complex (in full-face view, psammophore ranged along or just above anterior margin of clypeus and following the curve of the margin; anterior margin of clypeus broadly medially produced, and often with central notch that may be deeply impressed, but is never acuminate at its midpoint; metatibia with maximum of two rows of preapical spines). The Minor worker of this species is characterised as being very small (HW ≤ 0.50 mm), but it is the metafemur of the minor worker that is the best diagnostic charcter. This body part is attenuated to the midpoint and thereafter is of uniform width until its junction with the tibia (the media worker has a similar metafemur, but the attenuation is more gradual and less conspicuous). The head of the minor worker rather elongate and very strongly domed. The major worker can best be identified by the flattened anteromedial sector of its clypeus, this sector not extending beyond the lateral flanks of the clypeus.

#### Minor worker description.

**Head.** Head approximately oval with straight sides; posterior margin of head strongly convex; frons matt or with weak sheen, microreticulate or microreticulate-shagreenate; frons consisting exclusively or almost exclusively of well-spaced, appressed setae only (small, erect setae, if present, usually confined to ocular triangle or posterior margin of head). Eye moderate (eye length 0.20–0.49 length of side of head capsule); in full-face view, eyes set at about midpoint of head capsule; in profile, eye set around midline of head capsule; eyes elliptical or slightly reniform. In full-face view, frontal carinae distinctly concave; frontal lobes curved toward antennal insertion. Anteromedial clypeal margin broadly and evenly convex; clypeal psammophore set at or just above anterior clypeal margin; palp formula 6,4. Five mandibular teeth in minor worker; mandibles triangular, weakly incurved; third mandibular tooth distinctly shorter than apical tooth and teeth numbers two and four; masticatory margin of mandibles approximately vertical or weakly oblique. **Mesosoma.** Integument of pronotum, mesonotum and mesopleuron with weak to moderate sheen and superficial microreticulation (more pronounced on mesopleuron); anterior mesosoma in profile smoothly rounded anteriad, thereafter pronotum and whole of mesonotum flattened and on same plane as propodeum; erect pronotal setae absent; in profile, metanotal groove shallow, indicated mainly by an angle; propodeum shining and shagreenate; propodeum angulate, propodeal angle blunt; length ratio of propodeal dorsum to its declivity between 1:1 and 1:2; erect propodeal setae always absent; appressed propodeal setulae sparse or absent, if present then not regularly spaced; propodeal spiracle situated on or beside declivitous face of propodeum, and longer (length ≥ 0.50 × height of propodeum). **Petiole.** In profile, petiolar node squamiform; in full-face view, shape of petiolar node uniformly rounded; node shining and smooth with vestigial sculpture. **Gaster.** Gaster shining, shagreenate (‘LP record’ appearance); pilosity of first gastral tergite consisting of well-spaced short, inconspicuous, appressed setae only, erect setae always absent. **General characters.** Colour of head and foreparts tan, gaster chocolate, coxae and femora brown, tibia and tarsi mainly depigmented yellowish.

#### Major worker description.

**Head.** Head quadrate (i.e., heart-shaped); posterior margin of head weakly convex; cuticle of frons matt or with weak sheen, microreticulate; frons consisting exclusively or almost exclusively of well-spaced, appressed setae only (small, erect setae, if present, usually confined to ocular triangle or posterior margin of head). Eye moderate (eye length 0.20–0.49 length of head capsule); in full-face view, eyes set above midpoint of head capsule; in profile, eye set anteriad of midline of head capsule; eyes elliptical. In full-face view, frontal carinae straight, convergent posteriad; frontal lobes curved inward in front of antennal insertion. Anterior clypeal margin narrowly convex and protruding anteromedially, clypeal margin entire or weakly indented; clypeal psammophore set at or just above anterior clypeal margin; palp formula 6,4. Five mandibular teeth in major worker; mandibles triangular, weakly incurved; third mandibular tooth distinctly shorter than apical tooth and teeth numbers two and four; masticatory margin of mandibles approximately aligned vertically or weakly oblique. **Mesosoma.** Integument of pronotum, mesonotum and mesopleuron matt or with weak sheen and microreticulate throughout; anterior mesosoma in profile smoothly rounded anteriad, thereafter pronotum and whole of mesonotum flattened and on same plane as propodeum; erect pronotal setae absent; in profile, metanotal groove deep, V-shaped; propodeum matt or with a weak sheen and microreticulate; propodeum smoothly rounded or with indistinct angle; propodeal dorsum and declivity confluent; erect propodeal setae absent; appressed propodeal setae short, separated by more than own length and inconspicuous; propodeal spiracle situated on or beside declivitous face of propodeum, and longer (length ≥ 0.50 × height of propodeum). **Petiole.** In profile, petiolar node squamiform; in full-face view, shape of petiolar node tapered with squared-off vertex; node matt, shagreenate. **Gaster.** Gaster shining, shagreenate (‘LP record’ appearance); pilosity of first gastral tergite consisting of well-spaced short, inconspicuous, appressed setae, erect setae (present in at least some workers) confined to margin of sclerite. **General characters.** Colour of foreparts and legs orange tan (head slightly more reddish), gaster chocolate.

#### Measurements.

Worker (n = 3): CI 80–114; EI 20–32; EL 0.15–0.31; HL 0.56–1.37; HW 0.45–1.57; ML 0.74–1.69; MTL 0.53–1.10; PpH 0.09–0.18; PpL 0.42–0.72; SI 79–156; SL 0.70–1.24.

#### Comments.

The minor worker of this small species is recognizable by virtue of its small size (HW ≤ 0.50mm), much attenuated and long hind femur and strongly domed vertex. The media and major workers are less easily characterized, but the major worker has a flattened clypeus. The species appears to be restricted to the mid-west and Pilbara regions of Western Australia, and is known from a couple of collections only (WAM, one TERC record). Nothing is known of its biology or its genetics.

#### Etymology.

Compound of Latin *ad* (‘towards’) plus *tenuis* (‘thin’) and Greek *pes* (pl. of *pous* ‘foot’); adjective in nominative singular.

**Figure 7.**
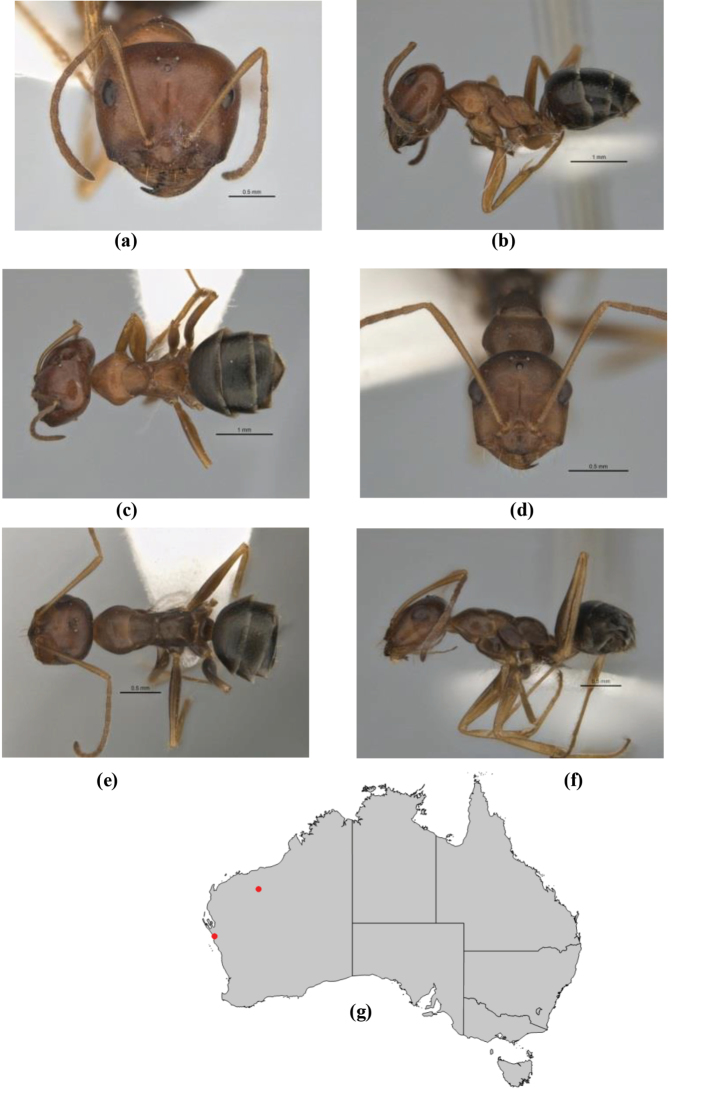
*Melophorus attenuipes* sp. n.: major worker paratype (JDM32-002000–top ant) frons (**a**), profile (**b**) and dorsum (**c**); minor worker holotype (JDM32-002000–bottom ant) frons (**d**), profile (**e**) and dorsum (**f**); distribution map for the species (**g**). Low resolution scale bars: 1 mm (**b, c**); 0.5 mm (**a, d–f**).

### Melophorus canus

Taxon classificationAnimaliaHymenopteraFormicidae

Heterick, Castalanelli & Shattuck sp. n.

http://zoobank.org/09B13F9B-95F3-45C8-9F2B-99EA1E2741BD

#### Types.

Holotype minor worker (top ant) from Goora Rock area, 70 miles from Esperance, Western Australia, 23 December 1969, B.B. Lowery, limestone mallee under rock [ANIC32-900003] (ANIC). Paratypes: 2 media workers, major worker and minor worker on same pin and with same details as the holotype (ANIC); 3 minor workers and major worker from Denmark, Western Australia, 19 December 1969, B.B. Lowery [ANIC32-900001] (BMNH); 2 minor workers, media worker, 3 major workers and male from Trundle, New South Wales, 9 January 1964, B.B. Lowery, in red soil, box pine scrub, Y35 [ANIC32-900005] (MCZ); minor worker from 107.2 km SSE of Newman 23°53'54"S, 120°36'14"E, Western Australia, October 1996, S. van Leeuwen & R. N. Bromilow, Invert., pitfall trap S3, Sand plain [JDM32-001987] (WAM).

#### Other material examined.

**Queensland**: ‘Merigol’ (Beutel, T.), Billabong W Salty Bore, Cravens Peak Station (Lemann, C. [ANIC32-035406]). **South Australia**: Black Oak Creek, Koonamore (Greenslade, P.J.M. [ANIC32-900004]). **Western Australia**: 0.9 km along Tanami Rd (Heterick, B.E. [M202]), Geraldton (Clark, J. [ANIC32-900002]), Mumbemarra Hill (10 km E of Geraldton) (Heterick, B.E. [JDM32-001986]).

#### Diagnosis.

*Melophorus canus* is a member of the *M. aeneovirens* species-group (in full-face view, the anterior clypeal margin convex, apron-like and covering whole or part of the retracted mandible, except in *M. nemophilus*, the medial clypeal sector often produced so that it is protrusive when seen in profile; the psammophore frequently with coarse and well-separated ammochaetae, these always placed on or just above anterior margin; in profile, the propodeum elongate and oblique or broadly rounded), and the *M. aeneovirens* species-complex (in full-face view, psammophore ranged along or just above anterior margin of clypeus and following the curve of the margin; anterior margin of clypeus broadly medially produced, and often with central notch that may be deeply impressed, but is never acuminate at its midpoint; metatibia with maximum of two rows of preapical spines). This particular species is distinctive because the main teeth of the mandible are supplemented with small or indistinct denticles, the total number of teeth and denticles ≥ six. Also, in full-face view, the head of the minor worker is indented below the eye, giving a bell-shaped appearance to the head capsule. This latter character is shared only with *M. griseus*, which, however has the typical unmodified *Melophorus* mandible. The species has a very dull, shaggy appearance, the workers being matt, shagreenate, and with very many long, flexuous setae over silvery pubescence.

#### Minor worker description.

**Head.** Head approximately oval, sides of head divergent towards mandibular articulations; posterior margin of head strongly convex; frons matt or with weak sheen, microreticulate or microreticulate-shagreenate; pilosity of frons a mixture of many long, curved, semi-erect setae and decumbent and appressed setae of varying lengths that form a coarse pubescence. Eye moderate (eye length 0.20–0.49 length of side of head capsule); in full-face view, eyes set above midpoint of head capsule; in profile, eye set anteriad of midline of head capsule; eyes elliptical or slightly reniform. In full-face view, frontal carinae straight or weakly convex, or straight, divergent posteriad; frontal lobes straight in front of antennal insertion, or curved inward in front of antennal insertion. Anteromedial clypeal margin narrowly protrusive anteromedially, the protrusion with a square border; clypeal psammophore set at or just above anterior clypeal margin; palp formula 6,4. Mandibular teeth in minor worker consisting of four distinct apical teeth with the basal denticle separated by one to several minute denticles or crenulations; mandibles triangular, weakly incurved; third mandibular tooth distinctly shorter than apical tooth and teeth numbers two and four; masticatory margin of mandibles approximately vertical or weakly oblique. **Mesosoma.** Integument of pronotum, mesonotum and mesopleuron with weak to moderate sheen, shagreenate on pronotum and dorsum of mesonotum, otherwise microreticulate; anterior mesosoma in profile broadly convex; appearance of erect pronotal setae long (i.e., longest erect setae longer than length of eye) and unmodified; in profile, metanotal groove shallow, broadly V or U-shaped; propodeum shining and shagreenate; propodeum angulate, propodeal angle blunt; length ratio of propodeal dorsum to its declivity between 3:2 and 4:3; erect propodeal setae present and abundant (greater than 12); appressed propodeal setulae long and closely aligned, creating pubescence; propodeal spiracle situated at least twice its width from the declivitous face of propodeum, and shorter (length < 0.50 × height of propodeum). **Petiole.** In profile, petiolar node squamiform; in full-face view, shape of petiolar node uniformly rounded; node shining and distinctly shagreenate-microreticulate. **Gaster.** Gaster weakly shining with indistinct shagreenation; pilosity of first gastral tergite consisting of a mixture of curved, erect and semi-erect setae and decumbent and appressed setae that form a variable pubescence. **General characters.** Colour brown or black (concolorous or bicoloured).

#### Major worker description.

**Head.** Head quadrate (i.e., heart-shaped); posterior margin of head planar or weakly concave; cuticle of frons matt or with weak sheen, microreticulate; pilosity of frons a mixture of many long, curved, semi-erect setae over well-spaced short semi-erect, decumbent and appressed setae. Eye moderate (eye length 0.20–0.49 length of head capsule); in full-face view, eyes set above midpoint of head capsule; in profile, eye set anteriad of midline of head capsule; eyes elliptical. In full-face view, frontal carinae straight, divergent posteriad; frontal lobes straight in front of antennal insertion. Anterior clypeal margin narrowly convex and protruding anteromedially, clypeal margin entire or weakly indented; clypeal psammophore set at or just above anterior clypeal margin; palp formula 6,4. Mandibular teeth in major worker consisting of four distinct apical teeth with the basal denticles separated by one to several minute denticles or crenulations; mandibles triangular, weakly incurved; third mandibular tooth distinctly shorter than apical tooth and teeth numbers two and four; masticatory margin of mandibles approximately aligned vertically or weakly oblique. **Mesosoma.** Integument of pronotum, mesonotum and mesopleuron matt or with weak sheen and microreticulate throughout; anterior mesosoma in profile pronotum smoothly rounded anteriad and flattened posteriad, mesonotum narrowly convex; erect pronotal setae long (i.e., longer than length of eye) and unmodified; in profile, metanotal groove shallow, broadly V- or U-shaped; propodeum matt or with a weak sheen and microreticulate; propodeum angulate, propodeal angle blunt; length ratio of propodeal dorsum to its declivity between 3:2 and 1:1; erect propodeal setae present and abundant (at least a dozen); appressed propodeal setae long and closely aligned, creating pubescence; propodeal spiracle situated nearer to midpoint of propodeum than to its declivitous face, and shorter (length less than 0.50 × height of propodeum), or situated at least twice its width from the declivitous face of propodeum, and shorter (length less than 0.50 × height of propodeum). **Petiole.** In profile, petiolar node squamiform; in full-face view, shape of petiolar node generally rounded with median indentation; node shining and faintly shagreenate-microreticulate. **Gaster.** Gaster weakly shining with indistinct shagreenation; pilosity of first gastral tergite consisting of a mixture of curved, erect and semi-erect setae and decumbent setae that form a variable pubescence. **General characters.** Colour dark reddish-brown.

#### Measurements.

Worker (n = 6): CI 87–111; EI 21–43; EL 0.31–0.41; HL 0.83–1.72; HW 0.72–1.91; ML 1.29–1.93; MTL 0.77–1.00; PpH 0.16–0.22; PpL 0.51–0.80; SI 57–137; SL 0.98–1.08.

#### Comments.

*Melophorus canus* is widely distributed throughout mainland Australia but is nowhere particularly common. The species is rendered distinctive by the long flexuous setae over thick, shining grey, appressed pilosity (hence the name), the somewhat bell-shaped appearance of the head of the minor worker when seen in full-face view and the appearance of the mandible. Genetic sequencing places it as the sister species to *M. griseus*. Collections have been made in a variety of habitats and geographic zones, and vegetation associations include limestone mallee and box-pine scrub but the habits of this species are unknown. A picture of the minor worker is found in fig. 15d in Greenslade 1979.

#### Etymology.

Latin *canus* (‘grey’ or ‘hoary’); adjective in nominative singular.

**Figure 8.**
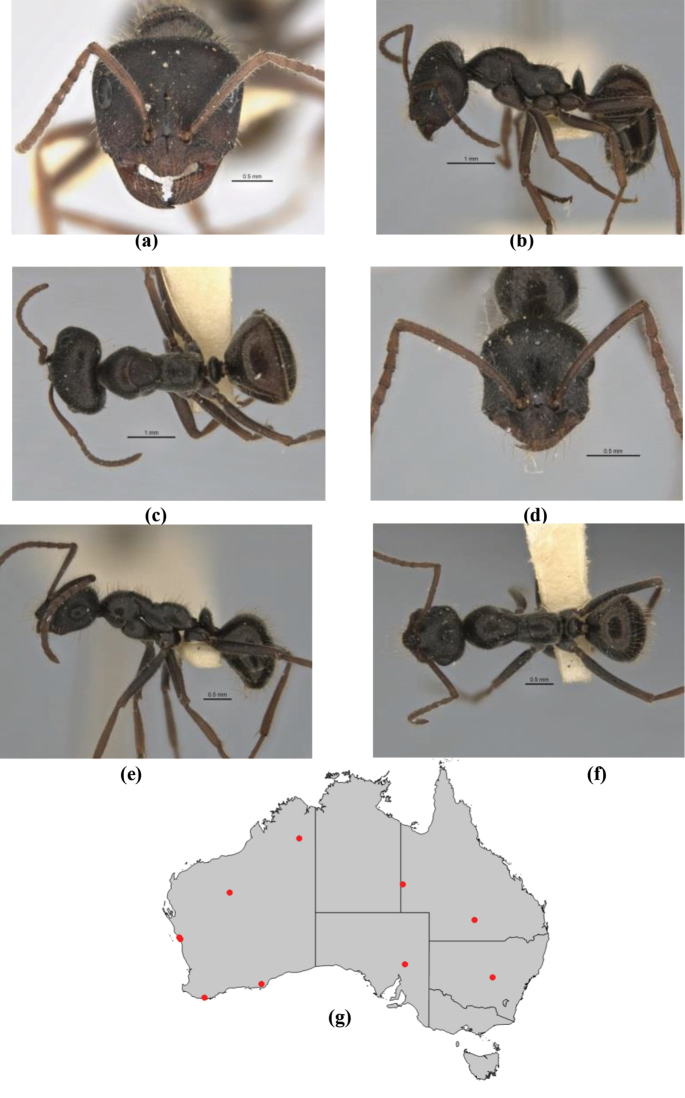
*Melophorus canus* sp. n.: major worker paratype (ANIC32-900003–third ant from top) frons (**a**), profile (**b**) and dorsum (**c**); minor worker (ANIC32-900003–top ant) holotype frons (**d**), profile (**e**) and dorsum (**f**); distribution map for the species (**g**). Low resolution scale bars: 1 mm (**b, c**); 0.5 mm (**a, d–f**).

### Melophorus castaneus

Taxon classificationAnimaliaHymenopteraFormicidae

Heterick, Castalanelli & Shattuck sp. n.

http://zoobank.org/226CE451-5272-4E58-8FFA-71427E4AD8BF

#### Types.

Holotype minor worker (bottom ant) from Gawler Ranges, South Australia, 5 October 1972, P.J.M. Greenslade, (8) [ANIC32-900180] (ANIC). Paratypes: major worker and media worker on same pin and with same details as holotype (ANIC); minor worker from 2 km E of Cowangie 35°14'S, 141°24'E, Victoria, 5 November 1991, S. Shattuck #2617.7 (ANIC); 3 minor workers from Kunoth Paddock, near Alice Springs, Northern Territory, 24-26 September 1980, P.J.M. Greenslade, Part A., (MCZ); minor worker from Sturt National Park, New South Wales, November 1979, P.J.M. Greenslade, Traps 2, 40) (BMNH); 2 minor workers from Kunoth Paddock, near Alice Springs, Northern Territory, 24 [September]- 8 October 1974, P.J.M. Greenslade, G. (QM); minor worker from 105 km N of Yuendumu, Northern Territory, 23 May 1986, P.J.M. Greenslade, (12), (Mu15) (SAM); media worker from Ethel Creek, Western Australia, 1993-4, P.A. Varris, ID: *Melophorus froggatti* Forel [*sic*], Det. by: Heterick, B.E., Date: 14 November 2006, checked against syntype specimen [*M. froggatti* is here regarded as a junior synonym of the closely related and similar *M. aeneovirens*-BEH] [ANIC32-004580) (WAM).

#### Other material examined.

**New South Wales**: 40 km NNW Louth, Lake Mere (Greenslade, P.J.M.), Bogan River (Armstrong, J.), CSIRO Lake Mere Field Station, near Louth (Bryannah, M.). **Northern Territory**: Kunoth Paddock, near Alice Springs (Greenslade, P.J.M.), Melville Island (Hoffmann, B.). **Queensland**: 42 mi NW Julia Creek (Dowse, J.E.), 4 mi ESE Toobeah (Greaves, T.). **South Australia**: 2.8 km NNW Four Hills Trig. Peake Stn (Stony Desert Survey UB02 [M90]), 7 km NW Morgan (Greenslade, P.J.M.). **Western Australia**: Ethel Creek (Varris, P.A. [JDM32-001499]), 10 mi NNW Gordon Downs Homestead (McInnes & Dowse [ANIC32-900085]), 11 km W Terhan Water Hole (Heatwole, H.).

#### Diagnosis.

*Melophorus castaneus* is a member of the *M. aeneovirens* species-group (in full-face view, the anterior clypeal margin convex, apron-like and covering whole or part of the retracted mandible, except in *M. nemophilus*, the medial clypeal sector often produced so that it is protrusive when seen in profile; the psammophore frequently with coarse and well-separated ammochaetae, these always placed on or just above anterior margin; in profile, the propodeum elongate and oblique or broadly rounded), and the *M. aeneovirens* species-complex (in full-face view, psammophore ranged along or just above anterior margin of clypeus and following the curve of the margin; anterior margin of clypeus broadly medially produced, and often with central notch that may be deeply impressed, but is never acuminate at its midpoint; metatibia with maximum of two rows of preapical spines). In *M. castaneus* the tibiae possess stout, socketed, appressed to subdecumbent setae only, with fine, appressed pubescence lacking. In profile, the mesosoma of the minor worker tends to linear in orientation, its dorsal outline straight or describing a weak arc (the mesosternal outline and the dorsum of the mesonotum being weakly convergent to subparallel anteriorly). The minor worker of *Melophorus castaneus* can readily be distinguished from its nearest relatives (*M. clypeatus*, *M. praesens* and *M. rufoniger*) by the uniformly rounded appearance of the vertex of the head when seen in full-face view, and by the uniformly convex propodeum. The major worker is less distinctive, but generally has the rounded propodeum seen in the minor worker, wheareas the *M. praesens* major worker propodeum is more sloping. The major worker clypeus also lacks the well-developed medial flange found in the major worker of *M. rufoniger* and is smaller (HW of the former has a HW of ≤ 2.10mm versus > 3 mm in *M. rufoniger*). The major worker of *M. clypeatus* also has a more protrusive clypeal flange than does the major worker of *M. castaneus*.

#### Minor worker description.

**Head.** Head approximately oval with straight sides; posterior margin of head strongly convex; frons shining with superficial shagreenation or microreticulation only, or matt or with weak sheen, shagreenate; pilosity of frons a mixture of a few well-spaced, erect setae interspersed with appressed setae only, or consisting exclusively or almost exclusively of well-spaced, appressed setae only (small, erect setae, if present, usually confined to ocular triangle or posterior margin of head). Eye moderate (eye length 0.20–0.49 length of side of head capsule); in full-face view, eyes set above midpoint of head capsule; in profile, eye set anteriad of midline of head capsule; eyes elliptical or slightly reniform. In full-face view, frontal carinae concave; frontal lobes straight in front of antennal insertion. Anteromedial clypeal margin narrowly convex and protruding, clypeal midpoint distinctly notched; clypeal psammophore set at or just above anterior clypeal margin; palp formula 6,4. Five mandibular teeth in minor worker; mandibles triangular, weakly incurved; third mandibular tooth distinctly shorter than apical tooth and teeth numbers two and four; masticatory margin of mandibles approximately vertical or weakly oblique. **Mesosoma**. Integument of pronotum, mesonotum and mesopleuron moderately shining and shagreenate throughout; anterior mesosoma in profile broadly convex; erect pronotal setae absent; in profile, metanotal groove shallow, broadly V or U-shaped; propodeum shining and finely striolate and microreticulate; propodeum smoothly rounded or with indistinct angle; propodeal dorsum and declivity confluent; erect propodeal setae always absent; appressed propodeal setulae long, each reaching setae behind and in front, but not forming pubescence, or short, separated by more than own length and inconspicuous; propodeal spiracle situated at least twice its width from the declivitous face of propodeum, and shorter (length < 0.50 × height of propodeum). **Petiole.** In profile, petiolar node rectangular, its vertex blunt, directed posteriad; in full-face view, shape of petiolar node uniformly rounded; node shining and smooth with vestigial sculpture. **Gaster.** Gaster shining, shagreenate (‘LP record’ appearance); pilosity of first gastral tergite consisting of well-spaced short, inconspicuous, appressed setae only, erect setae always absent. **General characters.** Colour of foreparts and appendages brown, gaster dark brown.

#### Major worker description.

**Head.** Head quadrate (i.e., heart-shaped); posterior margin of head weakly concave; cuticle of frons matt or with weak sheen, microreticulate; pilosity of frons a mixture of a few well-spaced, erect setae interspersed with appressed setae only. Eye moderate (eye length 0.20–0.49 length of head capsule); in full-face view, eyes set above midpoint of head capsule; in profile, eye set anteriad of midline of head capsule; roughly ovoid, eye narrowed posteriad. In full-face view, frontal carinae straight, divergent posteriad; frontal lobes straight in front of antennal insertion. Anterior clypeal margin narrowly convex and protruding anteromedially, clypeal midpoint notched; clypeal psammophore set at or just above anterior clypeal margin; palp formula 6,4. Five to six mandibular teeth in major worker; mandibles triangular, weakly incurved; third mandibular tooth distinctly shorter than apical tooth and teeth numbers two and four; masticatory margin of mandibles approximately aligned vertically or weakly oblique. **Mesosoma.** Integument of pronotum, mesonotum and mesopleuron with weak to moderate sheen, shagreenate on pronotum and dorsum of mesonotum, otherwise microreticulate; anterior mesosoma in profile broadly convex; erect pronotal setae short, (i.e., shorter than length of eye) and unmodified; in profile, metanotal groove deep, V-shaped; propodeum matt or with a weak sheen and microreticulate; propodeum always smoothly rounded; propodeal dorsum and declivity confluent; erect propodeal setae absent; propodeal spiracle situated on or beside declivitous face of propodeum, and shorter (length less than 0.50 × height of propodeum). **Petiole.** In profile, petiolar node squamiform; in full-face view, shape of petiolar node uniformly rounded; node shining and smooth with vestigial microreticulation anteriad. **Gaster.** Gaster shining, shagreenate (‘LP record’ appearance); pilosity of first gastral tergite consisting of well-spaced, short, thick, erect setae interspersed with minute, appressed setae. **General characters.** Colour of head and legs reddish-tan, mesosoma brownish-orange, gaster black.

#### Measurements.

Worker (n = 8): CI 84-111; EI 18-26; EL 0.21-0.37; HL 0.95-1.88; HW 0.79-2.08; ML 1.41-2.25; MTL 0.83-1.41; PpH 0.13-0.27; PpL 0.63-0.93; SI 77-147; SL 1.16-1.60

#### Comments.

This very typical member of the *M. aeneovirens* species-group is distinguished from similar species by the combination of its rounded propodeum (rather angulate in the similar *M. praesens*), the shape of its clypeus (less projecting than in *M. rufoniger*) and the lack of asymmetry in the vertex of the head capsule in the minor worker. The tibiae have socketed setae only, enabling it to be distinguished from morphologically similar populations of *M. curtus*. This species is most common in drier habitats, where it is likely to be a generalized forager, and it has a wide distribution in mainland Australia. However, this ant seems to be more common in eastern and central parts of the country. Some workers have been collected in modified habitats such as paddocks and rehabilitated grasslands. No specimens were available for sequencing during this project, but the appearance strongly suggests that the genetics of *M. castaneus* will eventually prove to be similar to those of *M. rufoniger* and *M. praesens*.

#### Etymology.

Latin *castaneus* (‘chestnut’ [i.e., coloured]); adjective in nominative singular.

**Figure 9.**
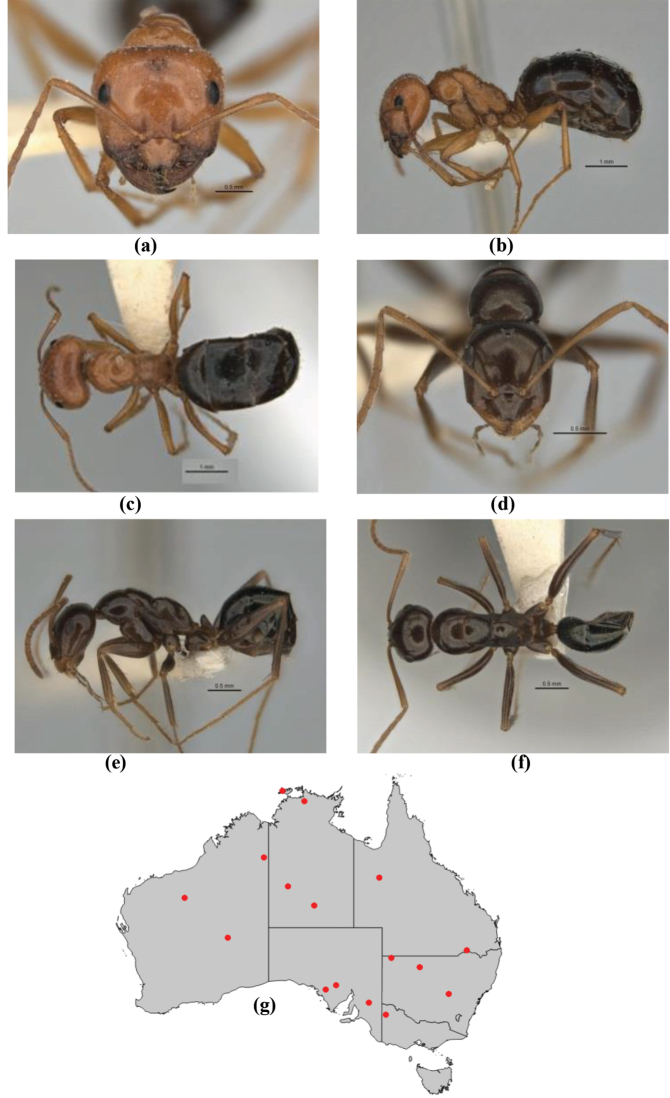
*Melophorus castaneus* sp. n.: major worker paratype (ANIC32-900180–top ant) frons (**a**), profile (**b**) and dorsum (**c**); minor worker holotype (ANIC32-900180–bottom ant) frons (**d**), profile (**e**) and dorsum (**f**); distribution map for the species (**g**). Low resolution scale bars: 1 mm (**b, c**); 0.5 mm (**a, d–f**).

### Melophorus clypeatus

Taxon classificationAnimaliaHymenopteraFormicidae

Heterick, Castalanelli & Shattuck sp. n.

http://zoobank.org/1C580977-0D20-4D64-A5EB-AA3572475269

#### Types.

Holotype minor worker (bottom ant) from 19.8 km WNW of Mt Berry 22°25'47"S, 116°16'47"E, Western Australia, 9 September 2003-10 October 2004, CALM Pilbara Survey, Site WYE10, Ethylene glycol pitfalls [JDM32-001497] (WAM). Paratypes: major and minor worker on same pin and with same details as the holotype (WAM); major and 2 minor workers on separate pin with exactly same details as the above (ANIC).

#### Diagnosis.

*Melophorus clypeatus* is a member of the *M. aeneovirens* species-group (in full-face view, the anterior clypeal margin convex, apron-like and covering whole or part of the retracted mandible, except in *M. nemophilus*, the medial clypeal sector often produced so that it is protrusive when seen in profile; the psammophore frequently with coarse and well-separated ammochaetae, these always placed on or just above anterior margin; in profile, the propodeum elongate and oblique or broadly rounded), and the *M. aeneovirens* species-complex (in full-face view, psammophore ranged along or just above anterior margin of clypeus and following the curve of the margin; anterior margin of clypeus broadly medially produced, and often with central notch that may be deeply impressed, but is never acuminate at its midpoint; metatibia with maximum of two rows of preapical spines). In *M. clypeatus* the tibiae possess stout, socketed, appressed to subdecumbent setae only, with fine, appressed pubescence lacking. In profile, the minor worker mesosoma In profile, the mesosoma of the minor worker tends to linear in orientation, its dorsal outline straight or describing a weak arc (the mesosternal outline and the dorsum of the mesonotum being weakly convergent to subparallel anteriorly. *Melophorus clypeatus* can be distinguished from from the (likely closely related) *M. praesens* by the narrow, rectangular, flanged appearance of the anteromedial margin of the clypeus in both major and minor workers, the flange having a straight or weakly indented edge.

#### Minor worker description.

**Head.** Head approximately oval with straight sides; posterior margin of head strongly convex; frons matt or with weak sheen, microreticulate or microreticulate-shagreenate; frons consisting exclusively or almost exclusively of well-spaced, appressed setae only (small, erect setae, if present, usually confined to ocular triangle or posterior margin of head). Eye moderate (eye length 0.20–0.49 length of side of head capsule); in full-face view, eyes set above midpoint of head capsule; in profile, eye set anteriad of midline of head capsule; eyes elliptical or slightly reniform. In full-face view, frontal carinae straight, divergent posteriad; frontal lobes curved toward antennal insertion. Anteromedial clypeal margin narrowly protrusive anteromedially, the protrusion with a square border; clypeal psammophore set at or just above anterior clypeal margin; palp formula 6,4. Five mandibular teeth in minor worker; mandibles triangular, weakly incurved; third mandibular tooth distinctly shorter than apical tooth and teeth numbers two and four; masticatory margin of mandibles approximately vertical or weakly oblique. **Mesosoma.** Integument of pronotum, mesonotum and mesopleuron with weak to moderate sheen, shagreenate on pronotum and dorsum of mesonotum, otherwise microreticulate; anterior mesosoma in profile broadly convex; erect pronotal setae absent; in profile, metanotal groove a weak or vestigial furrow; propodeum shining and shagreenate; propodeum always smoothly rounded; propodeal dorsum and declivity confluent; erect propodeal setae always absent; appressed propodeal setulae sparse or absent, if present then not regularly spaced; propodeal spiracle situated on or beside declivitous face of propodeum, and shorter (length < 0.50 × height of propodeum). **Petiole.** In profile, petiolar node squamiform; in full-face view, shape of petiolar node square with rounded angles; node shining and distinctly shagreenate-microreticulate. **Gaster.** Gaster shining, shagreenate (‘LP record’ appearance); pilosity of first gastral tergite consisting of well-spaced short, inconspicuous, appressed setae only, erect setae always absent. **General characters.** Colour tan, gaster chocolate.

#### Major worker description.

**Head.** Head square; posterior margin of head weakly concave; cuticle of frons matt or with weak sheen, microreticulate; frons consisting exclusively or almost exclusively of well-spaced, appressed setae only (small, erect setae, if present, usually confined to ocular triangle or posterior margin of head). Eye moderate (eye length 0.20–0.49 length of head capsule); in full-face view, eyes set above midpoint of head capsule; in profile, eye set anteriad of midline of head capsule; eyes elliptical. In full-face view, frontal carinae straight, divergent posteriad; frontal lobes straight in front of antennal insertion. Anterior clypeal margin narrowly convex and protruding anteromedially, clypeal midpoint notched; clypeal psammophore set at or just above anterior clypeal margin; palp formula 6,4. Five mandibular teeth in major worker; mandibles triangular, weakly incurved; third mandibular tooth distinctly shorter than apical tooth and teeth numbers two and four; masticatory margin of mandibles approximately aligned vertically or weakly oblique. **Mesosoma.** Integument of pronotum, mesonotum and mesopleuron with weak to moderate sheen, shagreenate on pronotum and dorsum of mesonotum, otherwise microreticulate; anterior mesosoma in profile broadly convex; erect pronotal setae short, (i.e., shorter than length of eye) and unmodified; in profile, metanotal groove shallow, broadly V- or U-shaped; propodeum shining and finely striolate and microreticulate; propodeum always smoothly rounded; propodeal dorsum and declivity confluent; erect propodeal setae absent; propodeal spiracle situated on or beside declivitous face of propodeum, and shorter (length less than 0.50 × height of propodeum). **Petiole.** In profile, petiolar node squamiform; in full-face view, shape of petiolar node tapered with blunt vertex, or tapered with squared-off vertex; node matt, shagreenate. **Gaster.** Gaster shining, shagreenate (‘LP record’ appearance); pilosity of first gastral tergite consisting of well-spaced, short, thick, erect setae interspersed with minute, appressed setae. **General characters.** Colour tan, gaster chocolate.

#### Measurements.

Worker (n = 2): CI 93–108; EI 16–25; EL 0.23–0.37; HL 0.96–2.09; HW 0.89–2.26; ML 1.52–2.27; MTL 0.89–1.31; PpH 0.13–0.20; PpL 0.71–1.10; SI 73–149; SL 1.33–1.65.

#### Comments.

Our knowledge of *Melophorus clypeatus* is restricted to two collections (ten workers) taken from the Pilbara region of Western Australia (WAM) and several records from TERC (one from Mt Isa, QLD). Nothing is known of its habits or biology, but the close resemblance to *M. praesens* (from which it differs only in colour and in the appearance of the clypeus) suggests similar habits to the latter.

#### Etymology.

Latin *clypeus* (‘armed with a shield’ [referring to the prominent clypeus]); noun in the nominative singular standing in apposition to the generic name

**Figure 10.**
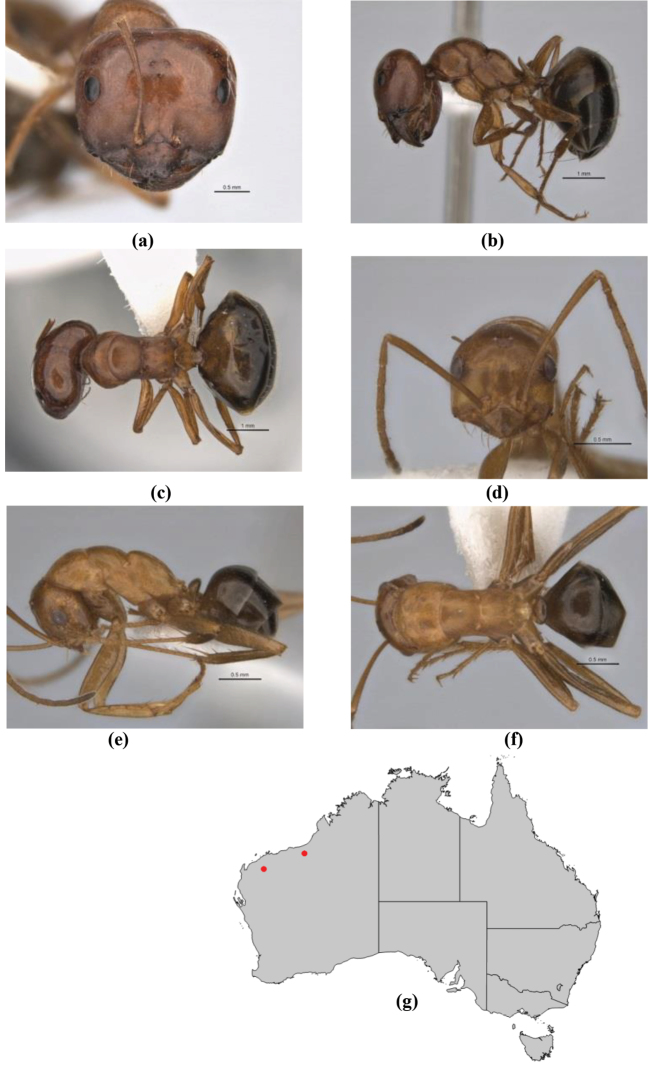
*Melophorus clypeatus* sp. n.: major worker paratype (JDM-001497–top ant) frons (**a**), profile (**b**) and dorsum (**c**); minor worker holotype (JDM32-001497–bottom ant on separate pin) frons (**d**), profile (**e**) and dorsum (**f**); distribution map for the species (**g**). Low resolution scale bars: 1 mm (**b, c**); 0.5 mm (**a, d–f**).

### Melophorus curtus

Taxon classificationAnimaliaHymenopteraFormicidae

Forel

Melophorus curtus Forel 1902: 485.

#### Types.

Syntype small major worker, MacKey, Queensland [ANIC], here designated lectotype: (examined: ANIC specimen ANIC32-053434). Also examined: syntype minor worker with same details as lectotype [ANIC], here designated a paralectotype; AntWeb images of syntype major and minor worker, MacKey, Queensland [BMNH] (CASENT0903264, CASENT0903265), here designated paralectotypes; AntWeb images of syntype major and minor worker, MacKey, Queensland [MHNG] (CASENT0909810, CASENT0909811), here designated paralectotypes. Also designated a paralectotype is a syntype queen [MHNG] (Taylor and Brown 1985), not seen.

#### Other material examined.

**New South Wales**: 40 km W Hillston (Greenslade, P.J.M. [ANIC32-900023]), Armidale (Lowery, B.B. [ANIC32-900016]), Armidale, west of golf course (Lowery, B.B. [ANIC32-900015]), Bogan River (Armstrong, J. [ANIC32-900017]), Bourke (Barrett, C.L. [ANIC32-900018]), Emmett Vale (Valentine [ANIC32-900020]), Hay (Lowery, B.B. [ANIC32-900021]), Hillston, The Common (Lowery, B.B. [ANIC32-900022]), Lachlan River, Condobolin (Lowery, B.B. [ANIC32-900019]), Mungindi (Lowery, B.B. [ANIC32-900024]), Round Mountain (NE NSW) (Lowery, B.B. [ANIC32-900025]), Whiporie, 55 km S Casino (York, A. [ANIC32-900026]). **Northern Territory**: CSIRO TERC, Darwin (Shattuck, S.O. [ANIC32-047317]), Hills, 27 km SW Katherine (Greenslade, P.J.M. [ANIC32-900009]), Kapalga, Alligator Rivers area (Greenslade, P.J.M. [ANIC32-900007]), Kapalga, Kakadu National Park (Andersen, A.N. [ANIC32-900008]), Manbullo I., SW Katherine (Greenslade, P.J.M. [ANIC32-900010]). **Queensland**: ‘Merigol’ (Beutel, T.), ‘Paingo’ turnoff (Monteath & Cook), ‘Wallaroo’ Hwy; 6 km WNW (Burwell/Monteath), 33 mi SSW Tara (Dowse, J.E. [ANIC32-900014]), Blackwood NP (Monteath & Cook), Blair Athol Mine (Houston, W. [ANIC32-040330]), Corry HS, 2 km SW, Retro-Corry Rd (Burwell), Fanning R Hmstd, 3.3 km SE (Monteith & Cook), Illaweena St. Drewvale (QM Party), Koondooloo, Hannaford Rd North via Tara (House, A./Brown, S.), Lizard Island (Reichel, H. [ANIC32-900011]), Lolworth NP (Wright, S.), Lords Table (Burwell), Moolayember Ck NP (Wright/Burwell), Moolayember Ck NP (Wright/Burwell), Moolayember Ck NP (Wright/Burwell), Mt Castor, 0.5 km W (Burwell/Monteath), Mt Pollux, SW base (Burwell, C.), Mt Pollux, SW base (Burwell, C.), N. Stradbroke Is. Enterprise (QM Party), Proserpine, Thompson Creek (Raven & Burwell), Ransome Reserve (QM Party), Redlands Hilliards Ck, nr South St (RHC1) (Stanisic, J.), Redlands Hilliards Ck, nr South St (RHC1) (BAAM/QM Party), St George (Lowery, B.B. [ANIC32-900013]), St George (Lowery, B.B. [ANIC32-900012]), Springsure, 16 km N (Monteath, G.), Tindaree, Hannaford Rd South via Tara (House, A./Brown, S.), Tindaree, Hannaford Rd South via Tara (House, A./Brown, S.), Wolfgang Peak (Burwell, C.J./ Monteath, G.). **Victoria**: Patho (Potter, H.A. [ANIC32-900027]), Patho (Potter, H.A. [ANIC32-039539]). **Western Australia**: Argyle Diamond Mine via Kununurra (Postle, A. [JDM32-004540]), Mulga, NE Goldfields (Pringle, H.J.R. [ANIC32-029573]), Packsaddle (van Leeuwen, S. [JDM32-004542]), Pilbara locality unknown (Unknown [JDM32-004539]), Tropicana minesite (Summerhayes, J. [JDM32-004541]).

#### Diagnosis.

*Melophorus curtus* is a member of the *M. aeneovirens* species-group (in full-face view, the anterior clypeal margin convex, apron-like and covering whole or part of the retracted mandible, except in *M. nemophilus*, the medial clypeal sector often produced so that it is protrusive when seen in profile; the psammophore frequently with coarse and well-separated ammochaetae, these always placed on or just above anterior margin; in profile, the propodeum elongate and oblique or broadly rounded), and the *M. aeneovirens* species-complex (in full-face view, psammophore ranged along or just above anterior margin of clypeus and following the curve of the margin; anterior margin of clypeus broadly medially produced, and often with central notch that may be deeply impressed, but is never acuminate at its midpoint; metatibia with maximum of two rows of preapical spines). In *M. curtus* the tibiae possess fine, appressed pubescence in addition to stout, socketed, appressed to subdecumbent setae This species differs from *M. sulconotus* in the appearance of the mesosoma, which, in profile, has a pronotum and mesonotum that are weakly to strongly convex in the minor worker, The antennal scapes and tibiae lack bristly erect and semi-erect setae, and short, erect setae are normally sparse on dorsum of mesosoma. The head of the minor worker is oval or squared and the frontal triangle is triangular in appearance. This combination of characters separates *M. curtus* from *M. gibbosus* and *M. griseus*, which share the pilosity pattern seen on the metatibae in *M. curtus*. Both *M. griseus* and *M. gibbosus* have erect setae on the antennal scape and the tibiae. The major worker of *M. curtus* can also be distinguished from that of *M. gibbosus* (the major worker of *M. griseus* is unknown) by its partially shagreenate frons, the frons being microreticulate in the *M. gibbosus* major worker.

#### Minor worker description.

**Head.** Head approximately oval with straight sides; posterior margin of head strongly convex; frons matt or with weak sheen, shagreenate; frons consisting almost completely of appressed setae that may form pubescence (tiny, erect setae, if present, usually confined to ocular triangle). Eye moderate (eye length 0.20–0.49 length of side of head capsule); in full-face view, eyes set above midpoint of head capsule; in profile, eye set anteriad of midline of head capsule; eyes elliptical or slightly reniform. In full-face view, frontal carinae concave; frontal lobes straight in front of antennal insertion. Anteromedial clypeal margin narrowly convex and protruding, clypeal margin entire or very weakly indented; clypeal psammophore set at or just above anterior clypeal margin; palp formula 6,4. Five to six mandibular teeth in minor worker; mandibles triangular, weakly incurved; third mandibular tooth distinctly shorter than apical tooth and teeth numbers two and four; masticatory margin of mandibles approximately vertical or weakly oblique. **Mesosoma**. Integument of pronotum, mesonotum and mesopleuron moderately shining and shagreenate throughout; anterior mesosoma in profile broadly convex; appearance of erect pronotal setae short, (i.e., longest erect setae shorter than length of eye) and unmodified, or erect pronotal setae absent; in profile, metanotal groove shallow, broadly V or U-shaped; propodeum shining and shagreenate; propodeum smoothly rounded or with indistinct angle; propodeal dorsum and declivity confluent; erect propodeal setae variable in number, may be absent; appressed propodeal setulae long and closely aligned, creating pubescence; propodeal spiracle situated on or beside declivitous face of propodeum, and shorter (length < 0.50 × height of propodeum). **Petiole.** In profile, petiolar node squamiform; in full-face view, shape of petiolar node square with rounded angles; node shining and distinctly shagreenate-microreticulate. **Gaster.** Gaster weakly shining with indistinct shagreenation; pilosity of first gastral tergite consisting of thick, appressed setae that form pubescence, interspersed with numerous short, bristly, erect setae, or consisting of long, closely aligned, whitish, appressed setae, with erect setae (present in at least some workers) confined to margin of the sclerite. **General characters.** Colour brown, gaster slightly darker in some workers.

#### Major worker description.

**Head.** Head quadrate (i.e., heart-shaped); posterior margin of head weakly concave; cuticle of frons shining with superficial shagreenation or microreticulation only; pilosity of frons a mixture of a few well-spaced, erect setae interspersed with appressed setae only. Eye small (eye length less than 0.2 × length of head capsule); in full-face view, eyes set above midpoint of head capsule; in profile, eye set anteriad of midline of head capsule; eyes elliptical. In full-face view, frontal carinae concave; frontal lobes curved inward in front of antennal insertion. Anterior clypeal margin narrowly convex and protruding anteromedially, clypeal midpoint notched; clypeal psammophore set at or above midpoint of clypeus; palp formula 6,4. Five mandibular teeth in major worker; mandibles triangular, weakly incurved; third mandibular tooth distinctly shorter than apical tooth and teeth numbers two and four; masticatory margin of mandibles approximately aligned vertically or weakly oblique. **Mesosoma.** Integument of pronotum, mesonotum and mesopleuron matt or with weak sheen and microreticulate throughout; anterior mesosoma in profile broadly convex; erect pronotal setae short, (i.e., shorter than length of eye) and unmodified; in profile, metanotal groove shallow, broadly V- or U-shaped; propodeum matt or with a weak sheen and indistinctly shagreenate; propodeum smoothly rounded or with indistinct angle; propodeal dorsum and declivity confluent; erect propodeal setae present and sparse to moderate (1-12); appressed propodeal setae long and closely aligned, creating pubescence; propodeal spiracle situated on or beside declivitous face of propodeum, and shorter (length less than 0.50 × height of propodeum). **Petiole.** In profile, petiolar node squamiform; in full-face view, shape of petiolar node uniformly rounded, or generally rounded with median indentation; node shining and faintly shagreenate-microreticulate. **Gaster.** Gaster shining, shagreenate (‘LP record’ appearance); pilosity of first gastral tergite consisting of thick, appressed setae that form pubescence, interspersed with numerous short, bristly, erect setae. **General characters.** Colour of foreparts orange-red, gaster brown to black.

#### Measurements.

Worker (n = 8): CI 87–109; EI 15–31; EL 0.18–0.40; HL 0.68–2.40; HW 0.59–2.62; ML 0.94–2.81; MTL 0.57–1.81; PpH 0.11–0.35; PpL 0.49–1.23; SI 72–149; SL 0.88–1.90.

#### Comments.

Most collections of *Melophorus curtus* have been made in Eastern Australia, where it is quite common, but no specimens were available for sequencing. There is some doubt that the taxon, as defined here, is monophyletic: although it is characterized by the presence of fine, appressed pubescence of the tibiae in addition to stout, socketed, appressed to subdecumbent setae, and also the absence of erect setae on the antenna and hind tibiae, other features are very variable, especially in the minor worker. Quite compressed, short-legged, relatively hairy workers are common in the Armidale area of NSW, while larger, more elongate and glabrous or near glabrous workers are common further north. In northern Australia a very elongate form with long, silvery, appressed setae on the head and body occurs. (However, this ant may represent the media worker of *M. tenuis*, which is here described on the basis of the minor only.) The moderately gracile morphotype, which is the most common, can also easily be mistaken for *M. praesens* or *M. castaneus*, and the tibiae of specimens must be examined carefully to distinguish the former species from the latter two. The possibility that there could be at least two or three cryptic species cannot be discounted, and further investigation involving genetics work is desirable in order to unravel the phylogenetics of such variable populations. A small syntype major worker from MacKey is here designated lectotype. This specimen not only represents the most common morph of *M. curtus* but has clearly recognizable defining features that belong to this taxon. The other syntypes here become paralectotypes.

Based on label data, this ant has a predilection for black soil, and has been taken in both dry sclerophyll woodland and savannah. Pitfall traps are a common means of capture. In all likelihood this species is a generalized scavenger.

**Figure 11.**
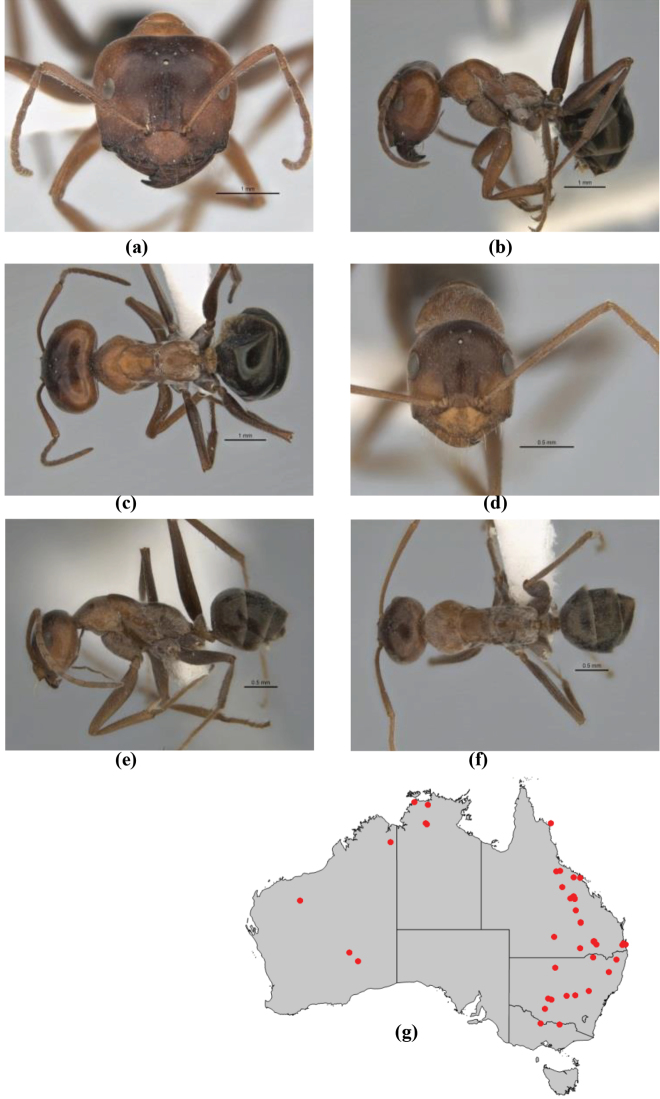
*Melophorus curtus* Forel: non–type major worker (ANCI32-900019–top ant) frons (**a**), profile (**b**) and dorsum (**c**); non–type minor worker frons (ANCI32-900019–bottom ant) (**d**), profile (**e**) and dorsum (**f**); distribution map for the species (**g**). Low resolution scale bars: 1 mm (**a–c**); 0.5 mm (**d–f**).

### Melophorus fulgidus

Taxon classificationAnimaliaHymenopteraFormicidae

Heterick, Castalanelli & Shattuck sp. n.

http://zoobank.org/971D5E94-7299-4063-9783-C19F0840BB25

#### Types.

Holotype minor worker (bottom ant) from Coward Springs 29.24S, 136.49E, South Australia, 22 September 1972, J. E. Feehan, ANIC Ant Vial 16.70 [ANIC32-066609] (ANIC). Paratypes: major and minor worker on same pin with same details as holotype (ANIC); queen and 2 minor workers from Coward Springs 29.24S, 136.49E, South Australia, 22 September 1972, J. E. Feehan, ANIC Ant Vial 16.70 [ANIC32 900006] (MCZ).

#### Diagnosis.

*Melophorus fulgidus* is a member of the *M. aeneovirens* species-group (in full-face view, the anterior clypeal margin convex, apron-like and covering whole or part of the retracted mandible, except in *M. nemophilus*, the medial clypeal sector often produced so that it is protrusive when seen in profile; the psammophore frequently with coarse and well-separated ammochaetae, these always placed on or just above anterior margin; in profile, the propodeum elongate and oblique or broadly rounded), and the *M. aeneovirens* species-complex (in full-face view, psammophore ranged along or just above anterior margin of clypeus and following the curve of the margin; anterior margin of clypeus broadly medially produced, and often with central notch that may be deeply impressed, but is never acuminate at its midpoint; metatibia with maximum of two rows of preapical spines). *Melophorus fulgidus* is not particularly distinctive, but can be identified in having, in full-face view, the eyes of the minor worker placed high on the head and breaking the outline of head capsule where the broadly convex posterior margin of the head meets the sides. Unlike most membes of the species-complex, both major and minor worker are glabrous, smooth and shining, with the appressed setae spaced much greater than their own length apart. In profile, the major worker has a flat mesonotum that overarches the pronotum, and, in full-face view, a protruding, weakly bifurcate anteromedial clypeal margin. This combination of characters separate *M. fulgidus* from other *Melophorus* in its species-complex, but careful checking is required. The species appears to be very localised in the Flinders Ranges in mid-north South Australia.

#### Minor worker description.

**Head.** Head approximately oval with straight sides; posterior margin of head strongly convex; frons shining with superficial shagreenation or microreticulation only; frons consisting exclusively or almost exclusively of well-spaced, appressed setae only (small, erect setae, if present, usually confined to ocular triangle or posterior margin of head). Eye moderate (eye length 0.20–0.49 length of side of head capsule); in full-face view, eyes set above midpoint of head capsule; in profile, eye set anteriad of midline of head capsule; eyes elliptical or slightly reniform. In full-face view, frontal carinae straight, convergent posteriad; frontal lobes straight in front of antennal insertion. Anteromedial clypeal margin narrowly protrusive anteromedially, the protrusion with a square border; clypeal psammophore set at or just above anterior clypeal margin; palp formula 6,4. Five mandibular teeth in minor worker; mandibles triangular, weakly incurved; third mandibular tooth distinctly shorter than apical tooth and teeth numbers two and four; masticatory margin of mandibles approximately vertical or weakly oblique. **Mesosoma.** Integument of pronotum, mesonotum and mesopleuron shining and mainly smooth, vestigial shagreenation most noticeable on humeri and mesopleuron; anterior mesosoma in profile convex anteriad, mesonotum often slightly overlapping pronotum, mesosoma planar or slightly sinuate posteriad; erect pronotal setae absent; in profile, metanotal groove shallow, broadly V or U-shaped; propodeum shining and uniformly striolate; propodeum smoothly rounded or with indistinct angle; propodeal dorsum and declivity confluent; erect propodeal setae always absent; appressed propodeal setulae short, separated by more than own length and inconspicuous; propodeal spiracle situated on or beside declivitous face of propodeum, and shorter (length < 0.50 × height of propodeum). **Petiole.** In profile, petiolar node squamiform; in full-face view, shape of petiolar node uniformly rounded; node shining and smooth throughout. **Gaster.** Gaster shining, shagreenate (‘LP record’ appearance); pilosity of first gastral tergite consisting of well-spaced short, inconspicuous, appressed setae only, erect setae always absent. **General characters.** Colour brown.

#### Major worker description.

**Head **. Head square; posterior margin of head weakly concave; cuticle of frons matt or with weak sheen, indistinctly shagreenate; frons consisting exclusively or almost exclusively of well-spaced, appressed setae only (small, erect setae, if present, usually confined to ocular triangle or posterior margin of head). Eye moderate (eye length 0.20–0.49 length of head capsule); in full-face view, eyes set above midpoint of head capsule; in profile, eye set anteriad of midline of head capsule; eyes elliptical. In full-face view, frontal carinae straight, convergent posteriad; frontal lobes straight in front of antennal insertion. Anterior clypeal margin narrowly convex and protruding anteromedially, clypeal midpoint notched; clypeal psammophore set at or just above anterior clypeal margin; palp formula 6,4. Five mandibular teeth in major worker; mandibles triangular, weakly incurved; third mandibular tooth distinctly shorter than apical tooth and teeth numbers two and four; masticatory margin of mandibles approximately aligned vertically or weakly oblique. **Mesosoma.** Integument of pronotum, mesonotum and mesopleuron moderately shining and shagreenate throughout; anterior mesosoma in profile convex anteriad, mesonotum overlapping pronotum, planar or slightly sinuate posteriad; erect pronotal setae absent; in profile, metanotal groove shallow, broadly V- or U-shaped; propodeum shining and shagreenate; propodeum always smoothly rounded; propodeal dorsum and declivity confluent; erect propodeal setae absent; propodeal spiracle situated on or beside declivitous face of propodeum, and shorter (length less than 0.50 × height of propodeum). **Petiole.** In profile, petiolar node squamiform; in full-face view, shape of petiolar node generally rounded with median indentation; node shining and faintly shagreenate-microreticulate. **Gaster.** Gaster shining, shagreenate (‘LP record’ appearance); pilosity of first gastral tergite consisting of well-spaced short, inconspicuous, appressed setae only, erect setae always absent. **General characters.** Colour of head orange, remainder of body brown.

#### Measurements.

Worker (n = 2): CI 94–109; EI 19–29; EL 0.26–0.36; HL 0.94–1.75; HW 0.88–1.90; ML 1.54–2.18; MTL 1/03–1.37; PpH 0.15–0.20; PpL 0.69–0.90; SI 85–152; SL 1.34–1.61.

#### Comments.

This species is known from two pins, both from Coward Springs, South Australia. The highly polished appearance, the overlapping mesonotum in the major worker and the high placement of the eyes on the head capsule in the minor worker help to distinguish this species from similar species in the *M. aeneovirens* group. Nothing is known of its biology.

#### Etymology.

Latin *fulgidus* (‘gleaming’); participle in nominative singular.

**Figure 12.**
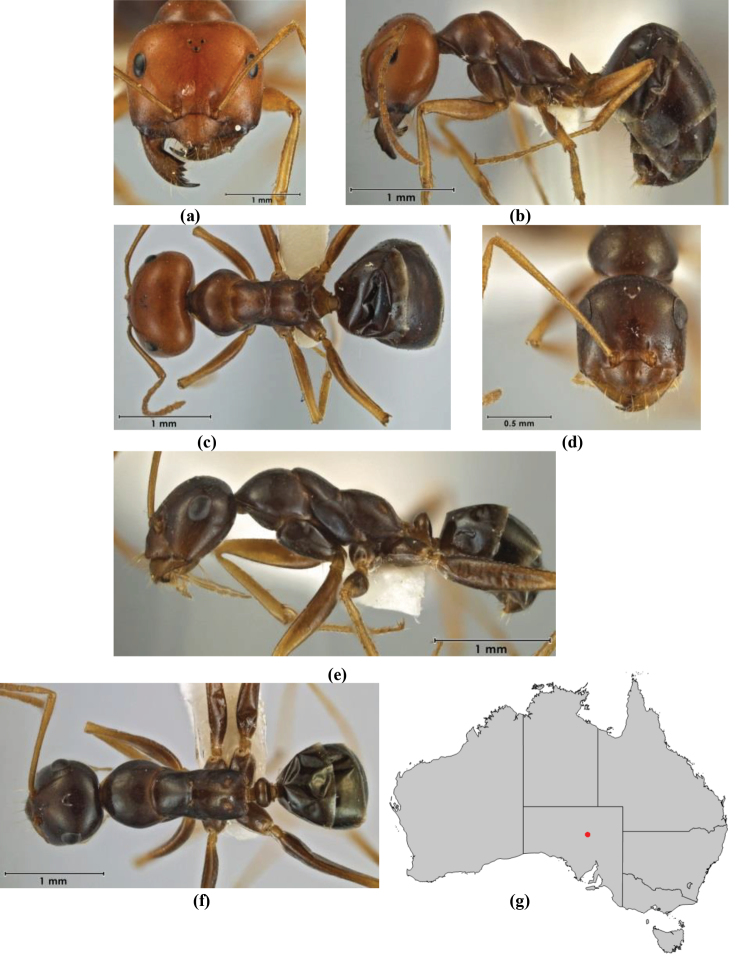
*Melophorus fulgidus* sp. n.: major worker (ANIC32-066609–top ant) paratype frons (**a**), profile (**b**) and dorsum (**c**); minor worker holotype (ANIC32-066609–bottom ant) frons (**d**), profile (**e**) and dorsum (**f**); distribution map for the species (**g**).

### Melophorus gibbosus

Taxon classificationAnimaliaHymenopteraFormicidae

Heterick, Castalanelli & Shattuck sp. n.

http://zoobank.org/7A7E365F-BF96-4BD0-A272-E74CDB7BF3D9

#### Types.

Holotype minor worker from Alligator Creek, S. Flinders Ra., South Australia, 23 April 1973, P.J.M. Greenslade [ANIC32-900191] (ANIC). Paratypes: 2 major workers, 2 minor workers, 1 alate queen, 1 male from Watsonia, Victoria, 24 May 1958, B. B. Lowery, ‘*Melophorus biroi*’ [*sic*] [ANIC32-900073] (BMNH); 3 minor workers from CSIRO Lake Mere field stn near Louth, New South Wales, paddock B Grove, January 1995, M. Bryannah, ‘Meloph. sp. × 6’ (MCZ); minor worker from Bauple, Queensland, August 1996, C. Vanderwoude, S.F. 958 *E. maculata* open forest plot 11 cpt 19, *Melophorus* sp. B (QM), 1 media worker from Fowlers Gap, New South Wales, November 1979, P.J.M. Greenslade, Traps 5 (27) (WAM).

#### Other material examined.

**Queensland**: ‘Merigol’ (Beutel, T.), Proserpine, Deadman Creek (Raven, R.), Tindaree, Hannaford Rd South via Tara (House, A./Brown, S.). **South Australia**: Mt. Remarkable, Flinders Ranges (Greenslade, P.J.M. [ANIC32-900072]), Oraparinna, Flinders Ranges (Greenslade, P.J.M.). **Western Australia**: Queen Victoria Spring (CALM [JDM32-001570]), Westonia (Harris, R. *et. al.* [JDM32-001569]).

#### Diagnosis.

*Melophorus gibbosus* is a member of the *M. aeneovirens* species-group (in full-face view, the anterior clypeal margin convex, apron-like and covering whole or part of the retracted mandible, except in *M. nemophilus*, the medial clypeal sector often produced so that it is protrusive when seen in profile; the psammophore frequently with coarse and well-separated ammochaetae, these always placed on or just above anterior margin; in profile, the propodeum elongate and oblique or broadly rounded), and the *M. aeneovirens* species-complex (in full-face view, psammophore ranged along or just above anterior margin of clypeus and following the curve of the margin; anterior margin of clypeus broadly medially produced, and often with central notch that may be deeply impressed, but is never acuminate at its midpoint; metatibia with maximum of two rows of preapical spines). In *M. gibbosus* the tibiae possess fine, appressed pubescence in addition to stout, socketed, appressed to subdecumbent setae. Unlike its near relative, *M. griseus*, the frontal carinae of the worker are not raised or laminate at the edges; the frontal triangle is triangular in appearance and the sides of the head of minor worker are not divergent below eyes. In profile, the pronotum and mesonotum form a strong convexity, and this is very distinctive of the ant. The major worker also has a uniformly microreticulate head.

#### Minor worker description.

**Head.** Head approximately oval with straight sides; posterior margin of head weakly convex; frons matt or with weak sheen, microreticulate or microreticulate-shagreenate; frons consisting of appressed pubescence, with many short, unmodified, erect setae. Eye moderate (eye length 0.20–0.49 length of side of head capsule); in full-face view, eyes set above midpoint of head capsule; in profile, eye set anteriad of midline of head capsule; eye more-or-less circular. In full-face view, frontal carinae concave; frontal lobes straight in front of antennal insertion. Anteromedial clypeal margin narrowly convex and protruding, clypeal midpoint distinctly notched; clypeal psammophore set at or just above anterior clypeal margin; palp formula 6,4. Five to six mandibular teeth in minor worker; mandibles triangular, weakly incurved; third mandibular tooth distinctly shorter than apical tooth and teeth numbers two and four; masticatory margin of mandibles approximately vertical or weakly oblique. **Mesosoma.** Integument of pronotum, mesonotum and mesopleuron moderately shining and shagreenate throughout; anterior mesosoma in profile pronotum smoothly rounded anteriad and flattened posteriad, mesonotum narrowly convex; appearance of erect pronotal setae short, (i.e., longest erect setae shorter than length of eye) and unmodified; in profile, metanotal groove shallow, broadly V or U-shaped; propodeum shining and shagreenate; propodeum always smoothly rounded; propodeal dorsum and declivity confluent; erect propodeal setae present and abundant (greater than 12); appressed propodeal setulae long and closely aligned, creating pubescence; propodeal spiracle situated at least twice its width from the declivitous face of propodeum, and shorter (length < 0.50 × height of propodeum). **Petiole.** In profile, petiolar node squamiform; in full-face view, shape of petiolar node uniformly rounded; node shining and distinctly shagreenate-microreticulate. **Gaster.** Gaster weakly shining with indistinct shagreenation; pilosity of first gastral tergite consisting of thick, appressed setae that form pubescence, interspersed with numerous short, bristly, erect setae. **General characters.** Colour concolorous brown.

#### Major worker description.

**Head.** Head oval; posterior margin of head planar or weakly convex; cuticle of frons matt or with weak sheen, microreticulate; frons consisting of appressed pubescence, with many short, unmodified, erect setae. Eye moderate (eye length 0.20–0.49 length of head capsule); in full-face view, eyes set above midpoint of head capsule; in profile, eye set anteriad of midline of head capsule; eyes elliptical. In full-face view, frontal carinae straight, divergent posteriad; frontal lobes straight in front of antennal insertion. Anterior clypeal margin narrowly convex and protruding anteromedially, clypeal midpoint notched; clypeal psammophore set at or just above anterior clypeal margin; palp formula 6,4. Five mandibular teeth in major worker; mandibles triangular, weakly incurved; third mandibular tooth distinctly shorter than apical tooth and teeth numbers two and four; masticatory margin of mandibles approximately aligned vertically or weakly oblique. **Mesosoma.** Integument of pronotum, mesonotum and mesopleuron matt with indistinct shagreenate sculpture throughout; anterior mesosoma in profile pronotum smoothly rounded anteriad and flattened posteriad, mesonotum narrowly convex; erect pronotal setae short, (i.e., shorter than length of eye) and unmodified; in profile, metanotal groove shallow, broadly V- or U-shaped; propodeum matt or with a weak sheen and indistinctly shagreenate; propodeum smoothly rounded or with indistinct angle; propodeal dorsum and declivity confluent; erect propodeal setae present and abundant (at least a dozen); appressed propodeal setae long and closely aligned, creating pubescence; propodeal spiracle situated nearer to midpoint of propodeum than to its declivitous face, and shorter (length less than 0.50 × height of propodeum). **Petiole.** In profile, petiolar node squamiform; in full-face view, shape of petiolar node generally rounded with median indentation; node shining and faintly shagreenate-microreticulate. **Gaster.** Gaster weakly shining with indistinct shagreenation; pilosity of first gastral tergite consisting of thick, appressed setae that form pubescence, interspersed with numerous short, bristly, erect setae. **General characters.** Colour brown, head and gaster darker than mesosoma.

#### Measurements.

Worker (n = 6): CI 95–107; E 18–27I; EL 0.21–0.27; HL 0.81–1.42; HW 0.77–1.52; ML 1.14–1.62; MTL 0.65–0.93; PpH 0.13–0.23; PpL 0.49–0.73; SI 77–121; SL 0.93–1.18.

#### Comments.

As with several other members of its species-group, *M. gibbosus* has a wide distribution in mainland Australia, but is relatively uncommon. Based on its appearance, this species seems to be a member of a small clade that also includes M. *griseus* and *M. canus*. However, this can not be tested as no specimens were available for sequencing. Specimen data indicate it has been collected from *Eucalyptus maculata* open forest and from *Callitris* woodland as well as from a paddock. Queensland Museum material also confirms its presence in mulga and brigalow regrowth. Erect bristly setae all over the head, body, antennal scapes and legs and a thoracic hump in the minor worker are sufficient to identify this species. The major worker has a distinctive, uniformly microreticulate frons.

#### Etymology.

Late Latin *gibbosus* (‘humpbacked’); adjective in the nominative singular.

**Figure 13.**
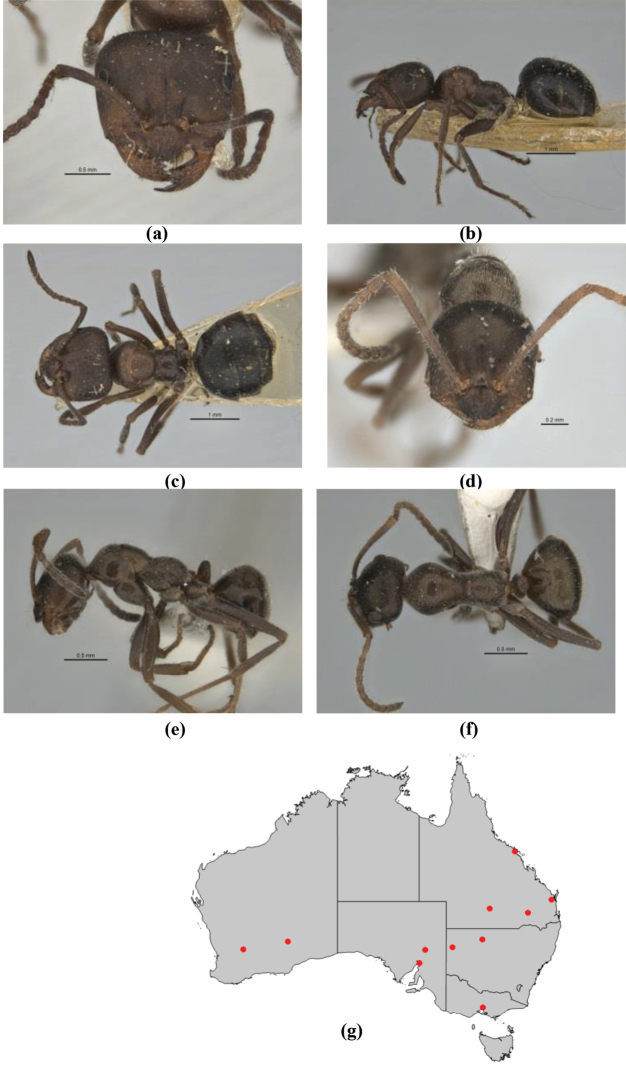
*Melophorus gibbosus* sp. n.: major worker paratype (ANIC32-900073–second point from top; end ant) frons (**a**), profile (**b**) and dorsum (**c**); minor worker holotype (ANIC32-900031) frons (**d**), profile (**e**) and dorsum (**f**); distribution map for the species (**g**). Low resolution scale bars: 1 mm (**b, c**); 0.5 mm (**a, e–f**); 0.2 (**d**).

### Melophorus griseus

Taxon classificationAnimaliaHymenopteraFormicidae

Heterick, Castalanelli & Shattuck sp. n.

http://zoobank.org/5B0258CA-A311-4AC4-BBD7-B1DA09D0CBCB

#### Types.

Holotype minor worker from Argyle Diamonds via Kununurra, Western Australia, September 1989, A. T. Postle, Site 15 Melophorus sp. [JDM32-004555] (WAM). Paratypes: 2 minor workers from Gibb River Road turnoff 15°50.096'S, 128°18.664'E, Western Australia, 9 February 2013 (3.40pm), Heterick, B.E., Sample 35: parking area with tall grass nearby, ABRS M21 (WAM).

#### Other material examined.

**Western Australia**: Gibb River Rd turnoff (Heterick, B.E., B.E. [M217/M218]).

#### Diagnosis.

*Melophorus griseus* is a member of the *M. aeneovirens* species-group (in full-face view, the anterior clypeal margin convex, apron-like and covering whole or part of the retracted mandible, except in *M. nemophilus*, the medial clypeal sector often produced so that it is protrusive when seen in profile; the psammophore frequently with coarse and well-separated ammochaetae, these always placed on or just above anterior margin; in profile, the propodeum elongate and oblique or broadly rounded), and the *M. aeneovirens* species-complex (in full-face view, psammophore ranged along or just above anterior margin of clypeus and following the curve of the margin; anterior margin of clypeus broadly medially produced, and often with central notch that may be deeply impressed, but is never acuminate at its midpoint; metatibia with maximum of two rows of preapical spines). In *M. griseus* the tibiae possess fine, appressed pubescence in addition to stout, socketed, appressed to subdecumbent setae *Melophorus griseus* differs from *M. curtus* by the features mentioned above. The Frontal carinae of the minor worker is raised and laminate at the edges, the frontal triangle is narrowly semi-oval, the sides of head are concave below eyes, giving it a bell-shaped appearance and, in profile, the in profile, pronotum and mesonotum form a gentle curve. These features separate it from its likely nearest relative, *M. gibbosus*.

#### Minor worker description.

**Head.** Head approximately oval, sides of head divergent towards mandibular articulations; posterior margin of head planar or weakly convex; frons matt or with weak sheen, microreticulate or microreticulate-shagreenate; frons consisting of appressed pubescence, with many short, unmodified, erect setae. Eye moderate (eye length 0.20–0.49 length of side of head capsule); in full-face view, eyes set above midpoint of head capsule; in profile, eye set anteriad of midline of head capsule; eyes elliptical or slightly reniform. In full-face view, frontal carinae straight, divergent posteriad; frontal lobes straight, elevated. Anteromedial clypeal margin narrowly convex and protruding, clypeal midpoint distinctly notched; clypeal psammophore set at or just above anterior clypeal margin; palp formula 6,4. Five mandibular teeth in minor worker; mandibles triangular, weakly incurved; third mandibular tooth distinctly shorter than apical tooth and teeth numbers two and four; masticatory margin of mandibles approximately vertical or weakly oblique. **Mesosoma.** Integument of pronotum, mesonotum and mesopleuron matt with indistinct shagreenate sculpture throughout; anterior mesosoma in profile broadly convex; appearance of erect pronotal setae short, (i.e., longest erect setae shorter than length of eye) and unmodified; in profile, metanotal groove shallow, broadly V or U-shaped; propodeum matt or with a weak sheen and indistinctly shagreenate; propodeum smoothly rounded or with indistinct angle; propodeal dorsum and declivity confluent; erect propodeal setae present and abundant (greater than 12); appressed propodeal setulae long and closely aligned, creating pubescence; propodeal spiracle situated at least twice its width from the declivitous face of propodeum, and shorter (length < 0.50 × height of propodeum). **Petiole.** In profile, petiolar node squamiform; in full-face view, shape of petiolar node generally rounded with median indentation or hollow; node matt with indistinct microsculpture. Gaster weakly shining with indistinct shagreenation; pilosity of first gastral tergite consisting of thick, appressed setae that form pubescence, interspersed with numerous short, bristly, erect setae. **General characters.** Colour brown, gaster slightly darker than foreparts.

#### Measurements.

Worker (n =1): CI 91; EI 23; EL 0.23; HL 1.10; HW 1.01; ML 1.58; MTL 1; PpH 0.166; PpL 0.62; SI 136; SL 1.40.

#### Comments.

This ant is differentiated from *M. canus* in lacking the long, silky setae and the dentiform mandible, the mandible in this case being five-toothed. The lack of a thoracic hump also helps to separate the minor worker from the minor worker of *M. gibbosus*. Most workers have bristly, erect setae on the antennae and tibiae. Workers collected in long grass near Dunham River, Western Australia, were extremely timid solitary foragers among (much more numerous) workers of *Iridomyrmex minor*, which they resemble in the field. The species is both genetically and morphologically closely related to *M. canus* but is more restricted in its distribution, being apparently confined to the top end, with collections made in NT, QLD and WA (TERC = ‘Group L’) and the Kimberley region in Western Australia (WAM).

#### Etymology.

Latin *griseus* (‘gray’); adjective in the nominative singular.

**Figure 14.**
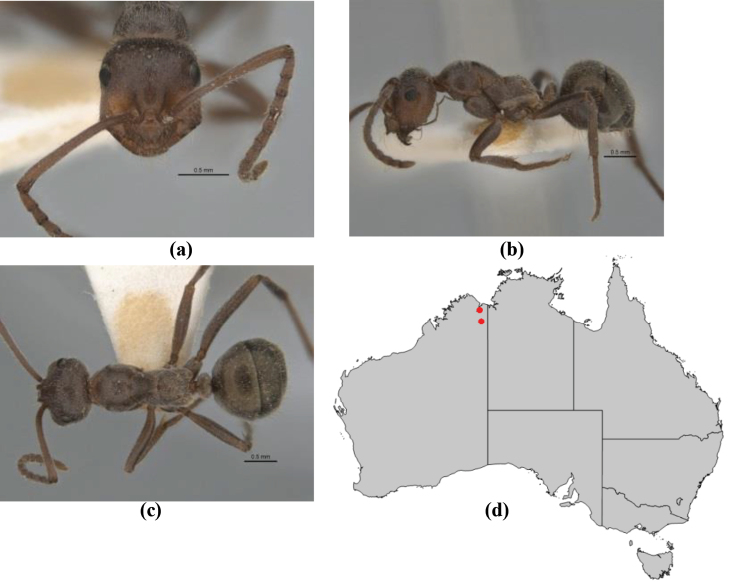
*Melophorus griseus* sp. n.: minor worker holotype (JDM32-004555) frons (**a**), profile (**b**) and dorsum (**c**); distribution map for the species (**d**). Low resolution scale bars: 0.5 mm (**a–c**).

### Melophorus kuklos

Taxon classificationAnimaliaHymenopteraFormicidae

Heterick, Castalanelli & Shattuck sp. n.

http://zoobank.org/460E9D5B-1ADF-4023-9B6C-A51702092A2A

#### Types.

Holotype minor worker from Simpson Gap 23.43S, 133.43E, Northern Territory, 6 October 1972, J. E. Feehan, [ANIC32-900095] (ANIC). Paratypes: major worker from slopes above Baroalba Springs 12.47S, 132.51E, Northern Territory, 13 June 1973, R.W. Taylor, Accession 73.608, [ANIC32-900094] (ANIC); dealate queen and 3 minor workers from c. 5 km S of Tor Rock 11.59'S, 133.05'E, 5 June 1973, Northern Territory, R.W. Taylor, outcrop area, Acc. 73.451 (BMNH); major and minor worker from slopes above Baroalba Springs 12.47S, 132.51E, Northern Territory, 17 November 1972, R.W. Taylor & J.E. Feehan, *Euc.* savanna, Acc. 72.1006, ANIC ANTS Vial 38.92 (MCZ).

#### Other material examined.

**Northern Territory**: Baroalba Spring (Taylor, R.W.), **Western Australia**: Augustus Island.

#### Diagnosis.

*Melophorus kuklos* is a member of the *M. aeneovirens* species-group (in full-face view, the anterior clypeal margin convex, apron-like and covering whole or part of the retracted mandible, except in *M. nemophilus*, the medial clypeal sector often produced so that it is protrusive when seen in profile; the psammophore frequently with coarse and well-separated ammochaetae, these always placed on or just above anterior margin; in profile, the propodeum elongate and oblique or broadly rounded), and the *M. aeneovirens* species-complex (in full-face view, psammophore ranged along or just above anterior margin of clypeus and following the curve of the margin; anterior margin of clypeus broadly medially produced, and often with central notch that may be deeply impressed, but is never acuminate at its midpoint; metatibia with maximum of two rows of preapical spines). In *M. kuklos* the tibiae possess stout, socketed, appressed to subdecumbent setae only, with fine, appressed pubescence lacking. In profile, the minor worker mesosoma is compact and has an arcuate outline. *Melophorus kuklos* most closely resembles *M. aeneovirens*, but in the former, in profile, the clypeus is straight or weakly and broadly convex, and produced over the mandible as a very pronounced ledge. In full-face view, the anteromedial margin of the major and minor worker clypeus is produced as a narrow flange that is distinctly notched or even forked at its midpoint. Also, in contrast to *M. aeneovirens*, the dorsum of the minor worker mesosoma is strongly arcuate and almost elliptical.

#### Minor worker description.

**Head.** Head approximately oval with straight sides; posterior margin of head extended posteriad as a convex, sloping surface with a slight medioccipital protuberance; frons shining with superficial shagreenation or microreticulation only; frons consisting exclusively or almost exclusively of well-spaced, appressed setae only (small, erect setae, if present, usually confined to ocular triangle or posterior margin of head). Eye moderate (eye length 0.20–0.49 length of side of head capsule); in full-face view, eyes set above midpoint of head capsule; in profile, eye set anteriad of midline of head capsule; eyes elliptical or slightly reniform. In full-face view, frontal carinae straight, divergent posteriad; frontal lobes curved inward in front of antennal insertion. Anteromedial clypeal margin narrowly convex and protruding, clypeal midpoint distinctly notched; clypeal psammophore set at or just above anterior clypeal margin; palp formula 6,4. Five mandibular teeth in minor worker; mandibles triangular, weakly incurved; third mandibular tooth distinctly shorter than apical tooth and teeth numbers two and four; masticatory margin of mandibles approximately vertical or weakly oblique. **Mesosoma.** Integument of pronotum, mesonotum and mesopleuron shining and mainly smooth, vestigial shagreenation most noticeable on humeri and mesopleuron; anterior mesosoma in profile broadly convex; erect pronotal setae absent; in profile, metanotal groove generally shallow (NT) but may be more deeply impressed (WA), broadly V or U-shaped; propodeum shining and shagreenate; propodeum uniformly flattened along an oblique trajectory; propodeal dorsum and declivity confluent; erect propodeal setae always absent; appressed propodeal setulae short, separated by more than own length and inconspicuous; propodeal spiracle situated on or beside declivitous face of propodeum, and shorter (length < 0.50 × height of propodeum). **Petiole.** In profile, petiolar node squamiform; in full-face view, shape of petiolar node square with rounded angles; node shining and smooth with vestigial sculpture. **Gaster.** Gaster shining with superficial microreticulation; pilosity of first gastral tergite consisting of well-spaced short, inconspicuous, appressed setae only, erect setae always absent. **General characters.** Colour dark brown.

#### Major worker description.

**Head.** Head square; posterior margin of head planar or weakly concave; cuticle of frons matt or with weak sheen, microreticulate; frons consisting exclusively or almost exclusively of well-spaced, appressed setae only (small, erect setae, if present, usually confined to ocular triangle or posterior margin of head). Eye small (eye length less than 0.2 × length of head capsule); in full-face view, eyes set above midpoint of head capsule; in profile, eye set anteriad of midline of head capsule; eyes elliptical. In full-face view, frontal carinae straight, divergent posteriad; frontal lobes curved inward in front of antennal insertion. Anterior clypeal margin narrowly convex and protruding anteromedially, clypeal margin entire or weakly indented, or narrowly convex and protruding anteromedially, clypeal midpoint notched; clypeal psammophore set at or just above anterior clypeal margin; palp formula 6,4. Five mandibular teeth in major worker; mandibles triangular, weakly incurved; third mandibular tooth distinctly shorter than apical tooth, but equivalent in length to remaining teeth; masticatory margin of mandibles approximately aligned vertically or weakly oblique. **Mesosoma.** Integument of pronotum, mesonotum and mesopleuron with weak to moderate sheen, shagreenate on pronotum and dorsum of mesonotum, otherwise microreticulate; anterior mesosoma in profile broadly convex; erect pronotal setae short, (i.e., shorter than length of eye) and unmodified; in profile, metanotal groove shallow, broadly V- or U-shaped; propodeum shining and shagreenate; propodeum smoothly rounded or with indistinct angle; propodeal dorsum and declivity confluent; erect propodeal setae absent; propodeal spiracle situated on or beside declivitous face of propodeum, and shorter (length less than 0.50 × height of propodeum). **Petiole.** In profile, petiolar node squamiform; in full-face view, shape of petiolar node uniformly rounded; node shining and smooth throughout. **Gaster.** Gaster shining, shagreenate (‘LP record’ appearance); pilosity of first gastral tergite consisting of well-spaced, erect and semi-erect setae interspersed with regularly spaced appressed setae. **General characters.** Colour russet.

#### Measurements.

Worker (n = 4): CI 89–106; EI 16–29; EL 0.18–0.25; HL 0.69–1.48; HW 0.61–1.57; ML 1.02–1.74; MTL 0.54–0.87; PpH 0.12–0.17; PpL 0.52–0.87; SI 72–141; SL 0.86–1.14.

#### Comments.

All but two of the known collections of this compact little member of the *M. aeneovirens* group have been taken in the NT, although the collection localities range from the far north (Baroalba Spring and Tor Rock in Arnhem Land) to Simpson Gap in the West MacDonnell Ranges, near Alice Springs. The two exceptions are an ant collected on Augustus Island, WA, at a malaise trap (WAM) and a collection from Yampi Island Station, WA (TERC; tentatively this species). *Melophorus kuklos* is most similar to *M. aeneovirens*, from which it is distinguished by its more arcuate mesosoma and its strongly produced clypeus, which is bifurcated anteromedially. No specimens were available for sequencing, but its morphology suggests a close relationship with *M. aeneovirens*. Although ecological data are limited it appears to be catholic in its requirements, samples having been taken from a rocky outcrop, eucalypt savannah and rainforest.

#### Etymology.

Greek *kuklos* (‘circle’, referring to the species’ outline); noun in the nominative singular standing in apposition to the generic name.

**Figure 15.**
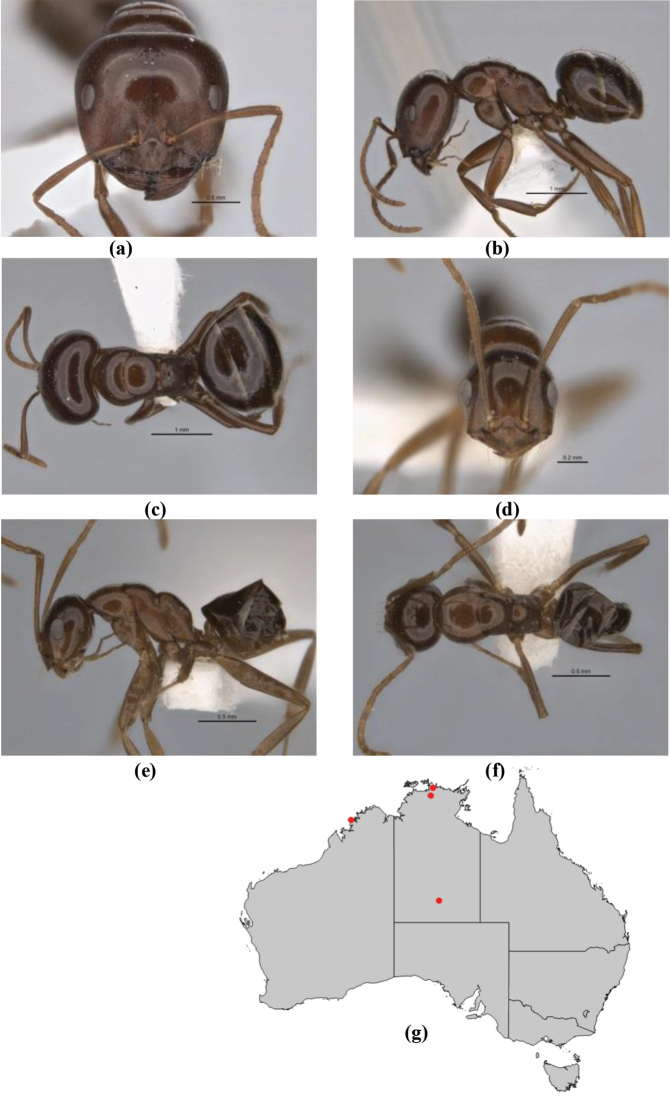
*Melophorus kuklos* sp. n.: major worker paratype (ANIC32-900094) frons (**a**), profile (**b**) and dorsum (**c**); minor worker holotype (ANIC32-900095) frons (**d**), profile (**e**) and dorsum (**f**); distribution map for the species (**g**). Low resolution scale bars: 1 mm (**b, c**); 0.5 mm (**a, e–f**); 0.2 (**d**).

### Melophorus mullewaensis

Taxon classificationAnimaliaHymenopteraFormicidae

Heterick, Castalanelli & Shattuck sp. n.

http://zoobank.org/44C81B1A-1FA6-4A1E-9220-601D51183CEA

#### Types.

Holotype minor worker (top ant) from Mullewa, Western Australia, 15 September 1931 [collector unknown] [ANIC32-06621] (ANIC). Paratype: Minor worker on same pin and with same details as holotype (ANIC).

#### Diagnosis.

*Melophorus mullewaensis* is a member of the *M. aeneovirens* species-group (in full-face view, the anterior clypeal margin convex, apron-like and covering whole or part of the retracted mandible, except in *M. nemophilus*, the medial clypeal sector often produced so that it is protrusive when seen in profile; the psammophore frequently with coarse and well-separated ammochaetae, these always placed on or just above anterior margin; in profile, the propodeum elongate and oblique or broadly rounded), and the *M. aeneovirens* species-complex (in full-face view, psammophore ranged along or just above anterior margin of clypeus and following the curve of the margin; anterior margin of clypeus broadly medially produced, and often with central notch that may be deeply impressed, but is never acuminate at its midpoint; metatibia with maximum of two rows of preapical spines). In *M. mullewaensis* the tibiae possess stout, socketed, appressed to subdecumbent setae only, with fine, appressed pubescence lacking. In profile, the mesosoma of the minor worker tends to linear in orientation, its dorsal outline straight or describing a weak arc (the mesosternal outline and the dorsum of the mesonotum being weakly convergent to subparallel anteriorly). Only the (damaged) minor worker of *Melophorus mullewaensis* is known. This species can be distinguished from all others in its species-complex by its flattened, attenuated pronotum.

#### Minor worker description.

**Head.** Head approximately oval with straight sides; posterior margin of head strongly convex; frons matt or with weak sheen, microreticulate or microreticulate-shagreenate; frons consisting exclusively or almost exclusively of well-spaced, appressed setae only (small, erect setae, if present, usually confined to ocular triangle or posterior margin of head). Eye moderate (eye length 0.20–0.49 length of side of head capsule); in full-face view, eyes set above midpoint of head capsule; in profile, eye set anteriad of midline of head capsule; eyes elliptical or slightly reniform. In full-face view, frontal carinae straight or weakly convex, frontal lobes straight in front of antennal insertion. Anteromedial clypeal margin broadly and evenly convex and protrusive; clypeal psammophore set at or just above anterior clypeal margin; palp formula 6,4. Five mandibular teeth in minor worker; mandibles triangular, weakly incurved; third mandibular tooth distinctly shorter than apical tooth and teeth numbers two and four; masticatory margin of mandibles approximately vertical or weakly oblique. **Mesosoma.** Integument of pronotum, mesonotum and mesopleuron with weak to moderate sheen, shagreenate on pronotum and dorsum of mesonotum, otherwise microreticulate; anterior mesosoma in profile convex anteriad, mesonotum often slightly overlapping pronotum, mesosoma planar or slightly sinuate posteriad; erect pronotal setae absent; in profile, metanotal groove shallow, indicated mainly by an angle; propodeum matt or with a weak sheen and indistinctly shagreenate; propodeum angulate, propodeal angle blunt; length ratio of propodeal dorsum to its declivity greater than 2:1; erect propodeal setae always absent; appressed propodeal setulae long, each reaching setae behind and in front, but not forming pubescence; propodeal spiracle situated on or beside declivitous face of propodeum, and shorter (length < 0.50 × height of propodeum). **Petiole.** In profile, petiolar node rectangular, vertex blunt, directed posteriad; in full-face view, shape of petiolar node uniformly rounded; node shining and distinctly shagreenate-microreticulate. **Gaster.** Gaster missing in two known specimens. **General characters.** Colour chocolate.

#### Measurements.

Worker (n = 2): CI 88; EI 25–28; EL 0.25–0.26; HL 1.05–1.18; HW 0.92–1.04; ML 1.91–2.01; MTL 1.13–1.21; PpH 0.17–0.14; PpL 0.80–0.87; SI 144 (one specimen); SL 1.49 (one specimen).

#### Comments.

This ant is known from a single pin of two damaged minor workers collected in the vicinity of Mullewa, Western Australia, very many years ago. These ants resemble the much more common *Melophorus aeneovirens* but are more gracile and the pronotum is more-or-less planar. Unfortunately the gaster of both specimens is missing. Nothing more is known about the species.

#### Etymology.

Named after the type locality; third declension masculine suffix added to toponym to form an adjective.

**Figure 16.**
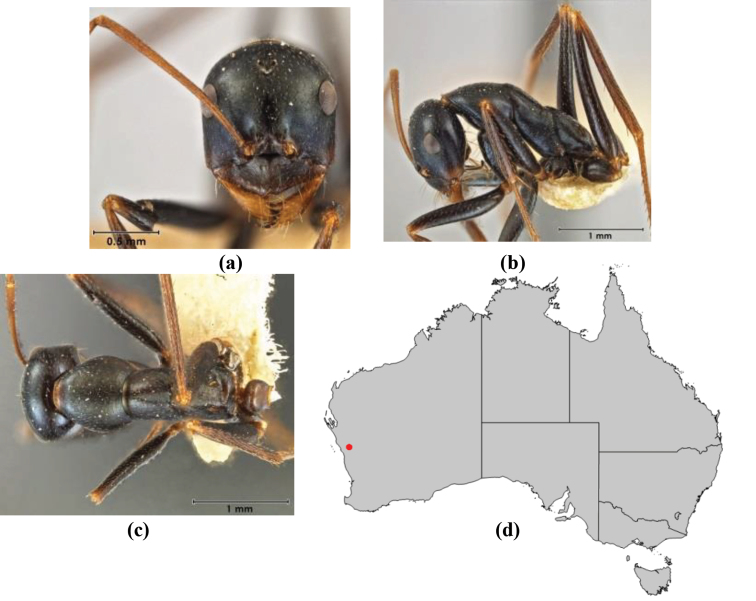
*Melophorus mullewaensis* sp. n.: minor worker holotype (ANIC32-06621–top ant) frons (**a**), profile (**b**) and dorsum (**c**); distribution map for the species (**d**).

### Melophorus platyceps

Taxon classificationAnimaliaHymenopteraFormicidae

Heterick, Castalanelli & Shattuck sp. n.

http://zoobank.org/4DA669F0-15AF-4EA0-BD7D-2E90D1CDC2A8

#### Types.

Holotype minor worker (top ant) from Koonamore, South Australia, 24 February 1973, P.J.M. Greenslade, (2) [ANIC32-900117]. Paratypes: 2 major workers on same pin and with same details as holotype (ANIC); 3 minor workers from 31.3 km S of Shearer’s Quarters 141°01'40"S, 34°50'30"E, Millewa South Bore Track, Murray Sunset National Park, Victoria, 14-23 November 2002, C. Lambkin, D. Yeates, N. Starwick & J. Recsei, 2m Sharkey malaise opening in closed mallee [ANIC32-043294] (ANIC); 2 major workers and minor worker from Blyth, N of Adelaide, South Australia, 11 June 1957, B.B. Lowery, 50 ft, mallee scrub, ANIC Ants Vial 22.69 (BMNH); major and 2 minor workers from Blyth, N of Adelaide, South Australia, 28 November 1957, B.B. Lowery, mallee scrub, ANIC Ants Vial 22.68 (MCZ); 3 minor workers from 5 km NW Ketchowla HS, South Australia, 23 January 1975, P.J.M. Greenslade, (5) (SAM); minor worker 2 km N of Wongan Hills, Western Australia, 24 February 1989, B. Heterick, soil, native vegetation, rural environment, 505, 8 *Mel*BH35 (WAM).

#### Other material examined.

**South Australia**: 10 km E Mt Ive Homestead, Gawler Ranges (Greenslade, P.J.M.), 15 km NE Mt Bryan (Greenslade, P.J.M.), 49 km N Minnipa (DEH Surv. 507-KDO-013 [M80/M106]), Brookfield Conservation Park (Shattuck, S.O.), Cambrai (Greenslade, P.J.M.), Cambrai (Greenslade, P.J.M.), Oraparinna (Greenslade, P.J.M.), Wudinna townsite (Heterick, B.E. [M329]). **Victoria**: 15 km WNW Yaapeet (Andersen, A.N.), ca. 15 km WNW Yaapeet, Wyperfeld National Park (Yen, A.H.), Murray Sunset Natl. Park, Millewa South Bore Track, 31.1 km S Shearers Quarters (Lambkin, C., Yeates, D., Starwick, N. & Recsei, J. [ANIC32-043294]). **Western Australia**: Woolundra Rd. (NW of Doodlakine) (Abensperg-Traun, M. [JDM32-001984]).

#### Diagnosis.

*Melophorus platyceps* is a member of the *M. aeneovirens* species-group (in full-face view, the anterior clypeal margin convex, apron-like and covering whole or part of the retracted mandible, except in *M. nemophilus*, the medial clypeal sector often produced so that it is protrusive when seen in profile; the psammophore frequently with coarse and well-separated ammochaetae, these always placed on or just above anterior margin; in profile, the propodeum elongate and oblique or broadly rounded), and the *M. aeneovirens* species-complex (in full-face view, psammophore ranged along or just above anterior margin of clypeus and following the curve of the margin; anterior margin of clypeus broadly medially produced, and often with central notch that may be deeply impressed, but is never acuminate at its midpoint; metatibia with maximum of two rows of preapical spines). *Melophorus platyceps* has a very flattened head, but can be separated from several other flat-headed Melophorus by its short maxillary palps which don’t reach the neck, and, in particular, in its more-or-less linear mesosomal dorsum after a weak anterior pronotal incline (when seen in profile). These two features, in particular, distinguish it from the otherwise similar *M. tenuis*, which belongs to the same species-complex. The metanotal groove is vestigial in the minor worker and weakly impressed in media and major workers, and the anterior clypeal margin weakly convex.

#### Minor worker description.

**Head.** Head rectangular; posterior margin of head weakly convex; frons coriaceous; frons consisting exclusively or almost exclusively of well-spaced, appressed setae only (small, erect setae, if present, usually confined to ocular triangle or posterior margin of head). Eye moderate (eye length 0.20–0.49 length of side of head capsule); in full-face view, eyes set above midpoint of head capsule; in profile, eye set anteriad of midline of head capsule; eyes elliptical or slightly reniform. In full-face view, frontal carinae straight, divergent posteriad; frontal lobes straight in front of antennal insertion. Anteromedial clypeal margin broadly and evenly convex; clypeal psammophore set at or just above anterior clypeal margin; palp formula 6,4. Five mandibular teeth in minor worker; mandibles triangular, weakly incurved; third mandibular tooth distinctly shorter than apical tooth and teeth numbers two and four; masticatory margin of mandibles approximately vertical or weakly oblique. **Mesosoma.** Integument of pronotum, mesonotum and mesopleuron matt with indistinct shagreenate sculpture throughout; anterior mesosoma in profile weakly elevated anteriad, thereafter uniformly more-or-less flat with metanotal suture absent or vestigial; erect pronotal setae absent; in profile, metanotal groove a weak or vestigial furrow; propodeum matt or with weak sheen and finely striolate; propodeum angulate, propodeal angle blunt; length ratio of propodeal dorsum to its declivity about 2:1; erect propodeal setae always absent; appressed propodeal setulae short, separated by more than own length and inconspicuous; propodeal spiracle situated on or beside declivitous face of propodeum, and shorter (length < 0.50 × height of propodeum). **Petiole.** In profile, petiolar node squamiform; in full-face view, shape of petiolar node square with rounded angles; node weakly shining and faintly striolate. **Gaster.** Gaster shining, shagreenate (‘LP record’ appearance); pilosity of first gastral tergite consisting of well-spaced short, inconspicuous, appressed setae only, erect setae always absent. **General characters.** Colour of foreparts orange or orange tan, gaster dark brown.

#### Major worker description.

**Head.** Head square; posterior margin of head planar or weakly concave; cuticle of frons matt or with weak sheen, indistinctly shagreenate; frons consisting exclusively or almost exclusively of well-spaced, appressed setae only (small, erect setae, if present, usually confined to ocular triangle or posterior margin of head). Eye moderate (eye length 0.20–0.49 length of head capsule); in full-face view, eyes set above midpoint of head capsule; in profile, eye set anteriad of midline of head capsule; eyes elliptical. In full-face view, frontal carinae straight, divergent posteriad; frontal lobes straight in front of antennal insertion. Anterior clypeal margin broadly and evenly convex; clypeal psammophore set below midpoint of clypeus; palp formula 6,4. Five mandibular teeth in major worker; mandibles triangular, weakly incurved; third mandibular tooth distinctly shorter than apical tooth and teeth numbers two and four; masticatory margin of mandibles approximately aligned vertically or weakly oblique. **Mesosoma.** Integument of pronotum, mesonotum and mesopleuron with weak to moderate sheen, shagreenate on pronotum and dorsum of mesonotum, otherwise microreticulate; anterior mesosoma in profile convex anteriad, mesonotum overlapping pronotum, planar or slightly sinuate posteriad; erect pronotal setae short, (i.e., shorter than length of eye) and unmodified, or erect pronotal setae absent; in profile, metanotal groove shallow, indicated mainly by an angle and metathoracic spiracles; propodeum matt or with weak sheen and microreticulate-striolate; propodeum angulate, propodeal angle blunt; length ratio of propodeal dorsum to its declivity about 4:3; erect propodeal setae absent; appressed propodeal setae short, separated by more than own length and inconspicuous; propodeal spiracle situated on or beside declivitous face of propodeum, and shorter (length less than 0.50 × height of propodeum). **Petiole.** In profile, petiolar node squamiform; in full-face view, shape of petiolar node square with rounded angles; node shining and faintly shagreenate-microreticulate. **Gaster.** Gaster shining, shagreenate (‘LP record’ appearance); pilosity of first gastral tergite consisting of well-spaced short, inconspicuous, appressed setae, erect setae (present in at least some workers) confined to margin of the sclerite. **General characters.** Colour as for minor worker.

#### Measurements.

Worker (n = 4): CI 84-115; EI 19-33; EL 0.20-0.29; HL 0.73-1.30; HW 0.62-1.50; ML 1.21-1.83; MTL 0.68-0.98; PpH 0.11-0.21; PpL 0.51-0.72; SI 77-156; SL 0.96-1.16

#### Comments.

On the three-gene tree (Figure 5) this species is sister to *M. praesens*. This species is unlikely to be mistaken for any other *Melophorus* because of its combination of an elongate, flattened head, flattened trunk and short palps. Also, like *M. nemophilus*, this is quintessentially an ant of the southern mallee woodlands, and its range exactly coincides with that of the former species. Label data are minimal, as is so often the case with this genus. The species is depicted in Greenslade 1979, Fig. 15a. Greenslade suggested the ant may be adapted to foraging under bark, and this species was among the most common *Melophorus* species taken in a recent collection by University of Western Australia researchers using bee-intercept traps on the base of eucalypts in the Avon Wheatbelt district.

#### Etymology.

Compound of Greek *platys* (‘broad’, ‘flat’) plus Latin -*ceps* (‘-headed’ [from caput]); adjective in the nominative singular.

**Figure 17.**
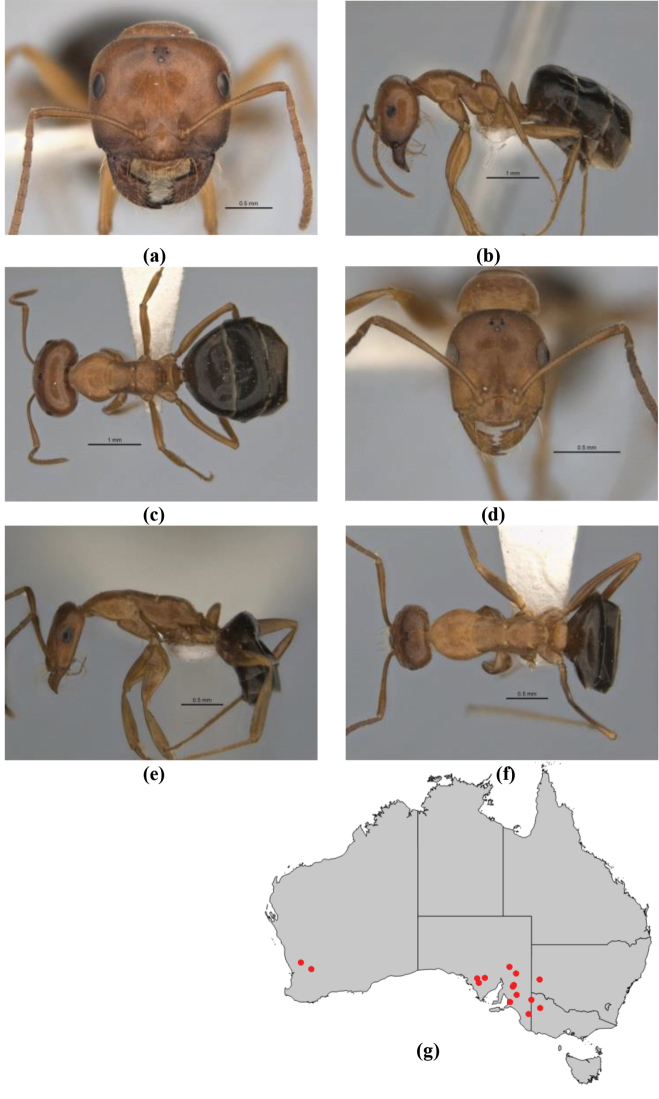
*Melophorus platyceps* sp. n.: major worker paratype (ANIC32-900117–top ant) frons (**a**), profile (**b**) and dorsum (**c**); minor worker holotype (ANIC32-900117–middle ant) frons (**d**), profile (**e**) and dorsum (**f**); distribution map for the species (**g**). Low resolution scale bars: 1 mm (**b, c**); 0.5 mm (**a, d–f**).

### Melophorus praesens

Taxon classificationAnimaliaHymenopteraFormicidae

Heterick, Castalanelli & Shattuck sp. n.

http://zoobank.org/13E54655-9150-4BBA-8361-8F907F6F6EBD

#### Types.

Holotype minor worker (top ant) from 85 km W of Mabel Creek, South Australia, 8–10 October 1980, P.J.M. Greenslade pt B, 24) [ANIC32-900179] (ANIC); Paratypes: 2 minor workers on same pin and with same details as holotype (ANIC); minor worker from 109.5 km SE Newman, 23°52'59"S, 120°38'31"E, Western Australia, June 1986, S. van Leeuwen & R.N. Bromilow, [JDM32-001483], Curtin University JDM Collection, donated 12 Jan. 2015 (BMNH); minor worker from 85 km W Mabel Creek, South Australia, 8-10 October 1980, P.J.M. Greenslade, Pt A, 23), *Melophorus* sp. 6 loan ANIC 1991 (MCZ); 3 minor workers from Sandringham, [SW] Queensland, February 1980, P.J.M. Greenslade leg. Morton, (10) (QM); 2 minor workers from 30 km E Poeppel Corner, Simpson Desert, South Australia, 25 August 1977, P.J.M. Greenslade (ANIC32-900083) (SAM); minor and 2 major workers from 24 miles ESE of Broome, 17 April 1963, McInnes & Dowse, 28706, Central & NW Aust. 1963, Series A223 (WAM).

#### Other material examined.

**Queensland**: 40 km E Cameron Corner (Greenslade, P.J.M.), Sandringham (Morton, S.). **South Australia**:, 3 minor workers (one broken in two) from Brookfield, South Australia, 29 October 1991, (Shattuck, s. [#2506.6]); 10 km E Mt Ive HS, Gawler Ranges, South Australia, (Greenslade, P.J.M.), Mt Serle Stn, 2.3 km SSE Moosha Bore (Flinders Ra surv REH0501). **Victoria**: 6.3 km N Hattah (Yen, A.L. [M109]). **Western Australia**: 8 km N Bullfinch (Heterick, B.E. [M130/M131]), Theda/Doongan, North Kimberley (Cross, A.) [not on map], Derby (Dodd, W.D.), Kojonup (Majer, J.D. [JDM32-001474]), Pardoo Stn turnoff (Heterick, B.E. [M266]), Tutanning, (Perth, A. [JDM32-001480] [major worker – imaged, this work]).

#### Diagnosis.

*Melophorus praesens* is a member of the *M. aeneovirens* species-group (in full-face view, the anterior clypeal margin convex, apron-like and covering whole or part of the retracted mandible, except in *M. nemophilus*, the medial clypeal sector often produced so that it is protrusive when seen in profile; the psammophore frequently with coarse and well-separated ammochaetae, these always placed on or just above anterior margin; in profile, the propodeum elongate and oblique or broadly rounded), and the *M. aeneovirens* species-complex (in full-face view, psammophore ranged along or just above anterior margin of clypeus and following the curve of the margin; anterior margin of clypeus broadly medially produced, and often with central notch that may be deeply impressed, but is never acuminate at its midpoint; metatibia with maximum of two rows of preapical spines). In *M. praesens* the tibiae possess stout, socketed, appressed to subdecumbent setae only, with fine, appressed pubescence lacking. In profile, the mesosoma of the minor worker tends to linear in orientation, its dorsal outline straight or describing a weak arc (the mesosternal outline and the dorsum of the mesonotum being weakly convergent to subparallel anteriorly). *Melophorus praesens* is almost identical to *M. clypeatus* but can be distinguished from that species by the less protrusive clypeus in both major and minor workers. Minor workers of *Melophorus praesens* can be distinguished from those of the similar *M. castaneus* by the asymmetrical appearance of the vertex and by the overlapping appressed setae on the gaster, and from minor workers of *M. rufoniger* by their smaller size (*M. rufoniger* HW of major worker >3 mm, HW of minor worker > 1 mm; *M. praesens* HW of major worker ≤ 2.3 mm, HW of minor worker < 1 mm) and the less protrusive medial sector of the clypeus.

#### Minor worker description.

**Head.** Head approximately oval with straight sides; posterior margin of head extended posteriad as a convex, sloping surface with a slight medioccipital protuberance; frons matt or with weak sheen, microreticulate or microreticulate-shagreenate; frons consisting exclusively or almost exclusively of well-spaced, appressed setae only (small, erect setae, if present, usually confined to ocular triangle or posterior margin of head). Eye moderate (eye length 0.20–0.49 length of side of head capsule); in full-face view, eyes set above midpoint of head capsule; in profile, eye set anteriad of midline of head capsule; eyes elliptical or slightly reniform. In full-face view, frontal carinae straight or weakly convex; frontal lobes straight in front of antennal insertion. Anteromedial clypeal margin narrowly convex and protruding, clypeal midpoint distinctly notched; clypeal psammophore set at or just above anterior clypeal margin; palp formula 6,4. Five mandibular teeth in minor worker; mandibles triangular, weakly incurved; third mandibular tooth distinctly shorter than apical tooth and teeth numbers two and four; masticatory margin of mandibles approximately vertical or weakly oblique. **Mesosoma**. Integument of pronotum, mesonotum and mesopleuron moderately shining and shagreenate throughout; anterior mesosoma in profile broadly convex; erect pronotal setae absent; in profile, metanotal groove deep, ‘V’-shaped, or shallow, broadly V or U-shaped; propodeum shining and shagreenate; propodeum smoothly rounded or with indistinct angle; propodeal dorsum and declivity confluent; erect propodeal setae always absent; appressed propodeal setulae long, each reaching setae behind and in front, but not forming pubescence; propodeal spiracle situated on or beside declivitous face of propodeum, and shorter (length < 0.50 × height of propodeum). **Petiole.** In profile, petiolar node subcuboidal, vertex bluntly rounded; in full-face view, shape of petiolar node uniformly rounded; node shining and smooth with vestigial sculpture. **Gaster.** Gaster shining, shagreenate (‘LP record’ appearance); pilosity of first gastral tergite consisting of well-spaced, long, whitish, appressed setae only, erect setae always absent. **General characters.** Two main colour forms: concolorous chocolate, and orange-tan with chocolate gaster.

#### Major worker description.

**Head.** Head quadrate (i.e., heart-shaped); posterior margin of head planar or weakly concave; cuticle of frons shining with superficial shagreenation or microreticulation only, or matt or with weak sheen, indistinctly shagreenate; frons consisting exclusively or almost exclusively of well-spaced, appressed setae only (small, erect setae, if present, usually confined to ocular triangle or posterior margin of head). Eye small (eye length less than 0.2 × length of head capsule); in full-face view, eyes set above midpoint of head capsule; in profile, eye set anteriad of midline of head capsule; eyes elliptical. In full-face view, frontal carinae straight, divergent posteriad; frontal lobes straight in front of antennal insertion. Anterior clypeal margin narrowly convex and protruding anteromedially, clypeal margin entire or weakly indented; clypeal psammophore set at or just above anterior clypeal margin; palp formula 6,4. Five mandibular teeth in major worker; mandibles triangular, weakly incurved; third mandibular tooth distinctly shorter than apical tooth and teeth numbers two and four; masticatory margin of mandibles approximately aligned vertically or weakly oblique. **Mesosoma.** Integument of pronotum, mesonotum and mesopleuron with weak to moderate sheen, shagreenate on pronotum and dorsum of mesonotum, otherwise microreticulate; anterior mesosoma in profile convex anteriad, mesonotum overlapping pronotum, planar or slightly sinuate posteriad; erect pronotal setae short, (i.e., shorter than length of eye) and unmodified; in profile, metanotal groove shallow, broadly V- or U-shaped; propodeum shining and microreticulate; propodeum smoothly rounded or with indistinct angle; propodeal dorsum and declivity confluent; erect propodeal setae present and sparse to moderate (1-12); appressed propodeal setae long and closely aligned, creating pubescence, or long, each reaching setae behind and in front, but not forming pubescence; propodeal spiracle situated on or beside declivitous face of propodeum, and shorter (length less than 0.50 × height of propodeum). **Petiole.** In profile, petiolar node squamiform; in full-face view, shape of petiolar node uniformly rounded; node shining and smooth with vestigial microreticulation anteriad. **Gaster.** Gaster shining, shagreenate (‘LP record’ appearance); pilosity of first gastral tergite consisting of longish, closely aligned, appressed setae interspersed with short, bristly, erect setae (some distally flattened). **General characters.** Colour of foreparts tan or reddish chocolate, gaster brown to chocolate.

#### Measurements.

Worker (n = 6): CI 86–108; EI 17–28; EL 0.24–0.38; HL 0.99–2.13; HW 0.85–2.31; ML 1.63–2.65; MTL 0.97–1.60; PpH 0.18–0.25; PpL 0.78–1.31; SI 76–157; SL 1.34–1.76.

#### Comments.

*Melophorus praesens* has a very broad distribution throughout Australia: records have come from all mainland states except NSW, but it probably occurs there as well. This species *sensu lato* can be distinguished from others in the *M. aeneovirens* complex by a combination of its asymmetrical head capsule, the shape of the propodeum and the conformation of the clypeus (not as protrusive as that of the very similar *M. rufoniger*, and resembling more that found in *M. aeneovirens* in most specimens). The tibiae completely lack fine, appressed pubescence in the northern topics and desert form, but there may be a few tiny setae, especially at the base of the tibia, that can be seen in some lights, in the southern form. A five-gene tree places this species as sister to *M. rufoniger*, which it very closely resembles. This ant has mainly been collected in arid and semi-arid areas. In more mesic localities it is replaced by *M. aeneovirens*. Labels reveal little in the way of additional data, but the species has a generalized morphology typical of the group and probably has habits to match. Collections have been made from a variety of habitats. One specimen hand collected near Pardoo, WA, was active in thundery conditions on a recently burnt plain, while another specimen was hand collected in Bullfinch, WA on red loam soil. Yet another sample was collected at Mt Serle Stn, SA, from a creek line in low rocky hills.

Note: what is here called ‘*praesens*’ reveals perhaps two forms that can only be distinguished by very subtle differences in the length and appearance of the appressed setae on the tibiae and gaster. Paratypes have been chosen that unequivocally match the holotype (northern/desert form), which has sparse, stout appressed setae on the tibiae and short appressed setae on the gaster that are mostly separated by more than their own length and do not form pubescence. All paratypes have orange-brown foreparts and brown gasters. There is the possibility that the hairier southern form (which has shining, elongate setae on the gaster that may form a weak pubescence, and a few minute appressed setae on the tibiae among rather elongate, fine appressed setae) is genetically distinct, and may constitute a western population of *M. curtus*, which it closely resembles in general morphology. In some series with dark brown minor workers the metanotal groove is deeply impressed and the propodeum has a marked anterior peak. Specimens have mainly been taken from south-west WA in drier areas and the south-east corner of SA. Further adding to the complication, the specimens from Brookfield (SA) and Junana Rock (WA), are particularly hirsute and have fine pubescence on the mesosoma in addition to the long, fine appressed setae on the gaster. Insufficient specimens have been sequenced (none of indubitable *M. curtus*) to do more than flag these apparent subtle differences here for future investigation.

#### Etymology.

Latin *praesens* (‘at hand’); participle in the nominative singular.

**Figure 18.**
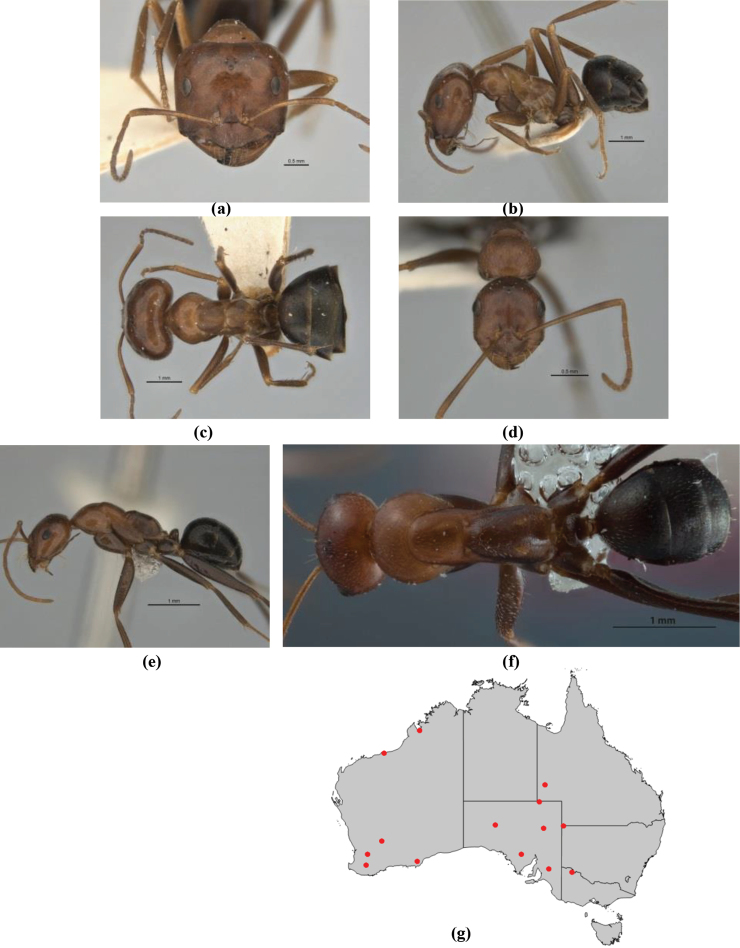
*Melophorus praesens* sp. n.: major worker (JDM32-001480) frons (**a**), profile (**b**) and dorsum (**c**); minor worker holotype (ANIC32-900117–top ant) frons (**d**), profile (**e**) and dorsum (**f**); distribution map for the species (**g**). Low resolution scale bars: 1 mm (**b, c**); 0.5 mm (**a, d–f**). Low resolution scale bars: 1 mm (**b, c, e**); 0.5 mm (**a, d**).

### Melophorus rufoniger

Taxon classificationAnimaliaHymenopteraFormicidae

Heterick, Castalanelli & Shattuck sp. n.

http://zoobank.org/CCA679CB-872D-4EAB-A6DB-B72E3E291CE2

#### Types.

Minor worker (bottom ant) from Koonamore, South Australia, 24-27 February 1973, P.J.M. Greenslade, pitfall traps [ANIC32-066663] (ANIC). Paratypes: media and minor worker on same pin and with same data as holotype (ANIC); media and 2 minor workers from Katarapko island, South Australia, 29 April 1999, Loxton High, River Red Gum Pitfall [ANIC32-046417] (ANIC); 2 minor workers from Whiporie, 55 km s of Casino 29°15'S, 152°27'E, New South Wales, February 1997, SGO. 7-9, ‘*Melophorus* sp. 1 RTU 78 (ANIC); major and minor worker from Koonamore, South Australia, 24 February 1973, P.J.M. Greenslade, 1), [ANIC32-066641] (BMNH); major and minor worker from WNW Morgan, South Australia, 2 March 1975, P.J.M. Greenslade (leg. R. B. Halliday), (Nest 284) (MCZ); minor and 1 media worker from Cooloola, Noosa River, Queensland, 20 February 1977, P.J.M. Greenslade, (7) (QM); 2 minor workers from Reny Island 34°03'59"S, 140°42'41"E, South Australia, 6 November 1998, Renmark High, Black Box flight intercept [ANIC32-046592] (SAM). 2 minor workers from 15 km ESE Gympie 26°16'S, 152°48'E, Queensland, 2 January 1989, S. O. Shattuck,100m, #1244, Wet sclerophyll, random ground foragers, ANIC ANTS VIAL 77.109 [ANIC32-044509] (WAM).

#### Other material examined.

**Australian Capital Territory**: Black Mountain (Greaves, T.), Black Mountain (Greaves, T.), Black Mountain (Mercovich, C.), Mt. Pleasant (Lowery, B.B.). **New South Wales**: 12 mi WSW Boomi (Greaves, T.), 40 km NNW Louth, Lake Mere (Greenslade, P.J.M.), Bogan River (Armstrong, J.), Broken Hill (Lowery, B.B.), Callubri Station, 2 mi from homestead (Greaves, T.), Callubri Station, 2 mi from homestead (Greaves, T.), CSIRO Lake Mere Field Station, near Louth (Bryannah, M.), Elura Mine, Cobar (Greenslade, P.J.M.), Fowlers Gap (Greenslade, P.J.M.), Fowlers Gap (Greenslade, P.J.M.), Fowlers Gap Research Station (Naumann, I.D. & Cardale, J.C.), Gwydir Hwy, 5 km E Warialda (Reichel, H.L.M. [ANIC32-007246]), Lightning Ridge (Lowery, B.B.), Newholme Road, near Armidale (Sakurai, Y.), S. Ita (Greenslade, P.J.M.), Tomago (Jackson, G.P. [ANIC32-015277]). **Northern Territory**: 200 km NE Alice Springs (Andersen, A.N.), 25 km N Alice Springs (Shattuck, S.O.), 25 km N Alice Springs (Shattuck, S.O.), 27 km SW Katherine (Greenslade, P.J.M.), 5 km S Jabiru, Alligator Rivers area (Greenslade, P.J.M.), Atartinga, NW Alice Springs (Greenslade, P.J.M. [ANIC32-900096]), Bing Bong homestead (Feehan, J.E.), Doyles Ridge (Greaves, T.), Gove, Nhulunbuy (Taylor, R.W. [ANIC32-002773]), Jabiru (Greenslade, P.J.M.), Jabiru (Greenslade, P.J.M.), Johnstons Lagoon (Greaves, T.), Kapalga, Alligator Rivers area (Greenslade, P.J.M.), Koolpinyah (Barrett, C.), Koolpinyah (Hill, G.F.), Kunoth Paddock, near Alice Springs (Greenslade, P.J.M.), Kunoth Paddock, near Alice Springs (Greenslade, P.J.M.), Manbulloo Research Station (Gross, G.), Manbulloo, SW Katherine (Greenslade, P.J.M.), Manbulloo, SW Katherine (Greenslade, P.J.M.), Manbulloo, SW Katherine (Greenslade, P.J.M.), nr Limbunya turnoff (Heterick, B.E. [M222/M223]), slopes above Baroalba Spring (Taylor, R.W. & Feehan, J.E.), slopes above Baroalba Spring (Taylor, R.W. & Feehan, J.E.). **Queensland**: 15.5 km S Emerald (Greenslade, P.J.M.), 2 km N Charleville (Edwards, E.D. & Fisk, J.H. [ANIC32-007282]), 2 km N Rokeby (Zborowski, P. & Horak, M. [ANIC32-043556]), 30 km SSE Heathlands (Shattuck, S.O.), 35 km SSW of Charleville (Edwards, E.D. & Fisk, J.H. [ANIC32-015215]), 3 km W Batavia Downs (Zborowski, P. & Dressler, W. [ANIC32-043028]), 3 km W Batavia Downs (Zborowski, P. & Calder, A. [ANIC32-042992]), 7 km N Finch Hatton (Shattuck, S.O. [ANIC32-045391]), Blair Athol Mine (Houston, W. [ANIC32-040334]), Burwilla, Cooloola (Greenslade, P.J.M.), Byfield State Forest (Shattuck, S.O. [ANIC32-044921]), Carnarvon Gorge National Park (Shattuck, S.O. [ANIC32-044799]), Clermont (Cudmore, R.A.), Como Scarp, Cooloola (Greenslade, P.J.M.), Cooloola (Plowman), Eulo (Greenslade, P.J.M.), Gold Creek Reserve, Brisbane (Lowery, B.B.), Hann River (Zborowski, P. & Horak, M. [ANIC32-031061]), Irene St., Cairns (collector unknown [ANIC32-015156]), Kabali East, Cooloola (Greenslade, P.J.M.), Kabali West, Cooloola (Greenslade, P.J.M.), Kabali West, Cooloola (Greenslade, P.J.M.), Kirrama (Greenslade, P.J.M.), Musgrave, 17 km W Coleman R Crossing (Eastwood, R. [M115]), near Dimbulah (Taylor, R.W. & Feehan, J.), Noosa River, Cooloola (Greenslade, P.J.M.), Noosa River, Cooloola (Greenslade, P.J.M.), Rainbow Beach, National Park Ranger Station (Shattuck, S.O. [ANIC32-044523]), Teewah Creek, Cooloola (Greenslade, P.J.M.), Teewah Creek, Cooloola (Greenslade, P.J.M.), Townsville (Conlith, S.), Wacol, Brisbane (Lowery, B.B.). **South Australia**: 15 km NW Blinman, Flinders Ranges (Greenslade, P.J.M. [ANIC32-900097]), 5 km W Geranium (Greenslade, P.J.M.), 8 km W Lameroo (Greenslade, P.J.M.), Blyth (Lowery, B.B.), Blyth (Lowery, B.B.), Buckleboo Homestead, N Eyre Peninsula (Greenslade, P.J.M.), Gawler Ranges (Greenslade, P.J.M); Katarapko Island (General) (Loxton High [ANIC32-046270]), Katarapko Island (General) (Loxton High [ANIC32-046408]), Koonamore (Greenslade, P.J.M.), Koonamore (Greenslade, P.J.M.), Loxton (Kaires, R. [ANIC32-015153]), Nth Moolooloo Stn, 6.7 km SSE (NWFR Survey PUTO2), Reny Island (Renmark High [ANIC32-046604]), Reny Island (Renmark High [ANIC32-046587]), Sturt Vale Homestead (Greenslade, P.J.M.), WNW Morgan (Halliday, R.B.). **Victoria**: 15 km WNW Yaapeet (Andersen, A.N.), 2 km E Cowangie (Shattuck, S.O.), Chinaman Well, Big Desert (Andersen, A.N.), Heathcote (Lowery, B.B.). **Western Australia**: 17 km E Invermay Stn (Heterick, B.E. [M227]), 26 mi SSE Karonie (Taylor, R.W.), 7 mi SSE Widgiemooltha (Taylor, R.W.), County Downs Hsd (south boundary) (Heterick, B.E. [M247]), Creek Crossing camp, 54 km W Durack River homestead (Shattuck, S.O.), Dunham River (rest area) (Heterick, B.E. [M213]).

#### Diagnosis.

*Melophorus rufoniger* is a member of the *M. aeneovirens* species-group (in full-face view, the anterior clypeal margin convex, apron-like and covering whole or part of the retracted mandible, except in *M. nemophilus*, the medial clypeal sector often produced so that it is protrusive when seen in profile; the psammophore frequently with coarse and well-separated ammochaetae, these always placed on or just above anterior margin; in profile, the propodeum elongate and oblique or broadly rounded), and the *M. aeneovirens* species-complex (in full-face view, psammophore ranged along or just above anterior margin of clypeus and following the curve of the margin; anterior margin of clypeus broadly medially produced, and often with central notch that may be deeply impressed, but is never acuminate at its midpoint; metatibia with maximum of two rows of preapical spines). In *M. rufoniger* the tibiae possess stout, socketed, appressed to subdecumbent setae only, with fine, appressed pubescence lacking. In profile, the mesosoma of the minor worker tends to linear in orientation, its dorsal outline straight or describing a weak arc (the mesosternal outline and the dorsum of the mesonotum being weakly convergent to subparallel anteriorly). *Melophorus rufoniger* major and minor workers can be distinguished by their large size (HW > 1 mm in minor workers and > 3 mm in major workers), the asymmetrical vertex in the minor worker and the well-developed and protrusive clypeal flange in both major and minor workers. This species is also predominantly red-and-black compared with the shades of brown or yellow seen in similar species.

#### Minor worker description.

**Head.** Head approximately oval, sides of head divergent towards mandibular articulations; posterior margin of head extended posteriad as a convex, sloping surface with a slight medioccipital protuberance; frons shining with superficial shagreenation or microreticulation only; frons consisting almost completely of appressed setae that may form pubescence (tiny, erect setae, if present, usually confined to ocular triangle). Eye moderate (eye length 0.20–0.49 length of side of head capsule); in full-face view, eyes set above midpoint of head capsule; in profile, eye set anteriad of midline of head capsule; eyes elliptical or slightly reniform. In full-face view, frontal carinae straight, divergent posteriad, or distinctly concave; frontal lobes straight in front of antennal insertion. Anteromedial clypeal margin narrowly convex and protruding, clypeal midpoint distinctly notched; clypeal psammophore set at or just above anterior clypeal margin; palp formula 6,4. Five to six mandibular teeth in minor worker; mandibles triangular, weakly incurved; third mandibular tooth distinctly shorter than apical tooth and teeth numbers two and four; masticatory margin of mandibles approximately vertical or weakly oblique. **Mesosoma.** Integument of pronotum, mesonotum and mesopleuron moderately shining and shagreenate throughout; anterior mesosoma in profile broadly convex; erect pronotal setae absent; in profile, metanotal groove deep, ‘V’-shaped, or shallow, broadly V or U-shaped; propodeum shining and shagreenate; propodeum angulate, propodeal angle blunt; length ratio of propodeal dorsum to its declivity greater than 2:1; erect propodeal setae always absent; appressed propodeal setulae minute and closely aligned, creating a silvery sheen; propodeal spiracle situated on or beside declivitous face of propodeum, and shorter (length < 0.50 × height of propodeum). **Petiole.** In profile, petiolar node rectangular, vertex blunt, directed posteriad; in full-face view, shape of petiolar node uniformly rounded, or square with rounded angles; node shining and smooth with vestigial sculpture. **Gaster.** Gaster shining, shagreenate (‘LP record’ appearance); pilosity of first gastral tergite consisting of well-spaced, long, whitish, appressed setae only, erect setae always absent. **General characters.** Colour of foreparts orange to reddish-tan, legs light tan, gaster chocolate.

#### Major worker description.

**Head.** Head quadrate (i.e., heart-shaped); posterior margin of head strongly concave; cuticle of frons shining and smooth except for piliferous pits, or shining with superficial shagreenation or microreticulation only; frons consisting exclusively or almost exclusively of well-spaced, appressed setae only (small, erect setae, if present, usually confined to ocular triangle or posterior margin of head). Eye small (eye length less than 0.2 × length of head capsule); in full-face view, eyes set above midpoint of head capsule; in profile, eye set anteriad of midline of head capsule; eyes elliptical. In full-face view, frontal carinae straight, divergent posteriad; frontal lobes straight in front of antennal insertion. Anterior clypeal margin narrowly convex and protruding anteromedially, clypeal midpoint notched; clypeal psammophore set at or just above anterior clypeal margin; palp formula 6,4. Five mandibular teeth in major worker; mandibles triangular, weakly incurved; third mandibular tooth distinctly shorter than apical tooth, but equivalent in length to remaining teeth; masticatory margin of mandibles approximately aligned vertically or weakly oblique. **Mesosoma.** Integument of pronotum, mesonotum and mesopleuron matt with indistinct shagreenate sculpture throughout; anterior mesosoma in profile broadly convex; erect pronotal setae absent; in profile, metanotal groove shallow, broadly V- or U-shaped; propodeum matt or with a weak sheen and indistinctly shagreenate; propodeum smoothly rounded or with indistinct angle; propodeal dorsum and declivity confluent; erect propodeal setae absent; appressed propodeal setae minute and closely aligned, creating a silvery sheen; propodeal spiracle situated on or beside declivitous face of propodeum, and shorter (length less than 0.50 × height of propodeum). **Petiole.** In profile, petiolar node squamiform; in full-face view, shape of petiolar node uniformly rounded, or generally rounded with median indentation, or square with rounded angles; node shining and faintly shagreenate-microreticulate. **Gaster.** Gaster shining, shagreenate (‘LP record’ appearance); pilosity of first gastral tergite consisting of well-spaced short, inconspicuous, appressed setae only, erect setae always absent. **General characters.** Colour of foreparts reddish- or orange-brown, gaster brown to chocolate.

#### Measurements.

Worker (n = 8): CI 87–109; EI 14–25; EL 0.28–0.47; HL 1.26–3.19; HW 1.10–3.46; ML 2.12–3.59; MTL 1.43–2.30; PpH 0.21–0.47; PpL 0.96–1.65; SI 69–158; SL 1.74–2.39.

#### Comments.

This species, like the preceding one, is widely distributed throughout Australia, although it avoids the wetter south-west corner of WA and similarly cool, moist habitats in SA and Vic. *Melophorus rufoniger* is on average larger than *M. praesens* and lighter in colour, predominantly red or orange with a darker gaster. The clypeus is produced well beyond the base of the mandibles, and this feature and the asymmetrical head enable it to be distinguished from other members of its complex except for some morphologically very similar populations of reddish *M. curtus*, in which case the pilosity of the hind femur must be closely examined. Despite its close physical resemblance to *M. praesens*, a three-gene tree places this species as sister to *M. sulconotus*, a quite different-looking species with a distinctively plateau-like pronotum and mesonotum that descends abruptly to the propodeum. (*Melophorus sulconotus* was not successfully sequenced for the full five genes, so *M. rufoniger* and *M. praesens* appear as sisters on the five-gene tree, as might be expected from the morphology.) *Melophorus rufoniger* has been collected frequently in both red clay soil and yellow soil and also in a variety of vegetation zones. The latter include eucalyptus savanna, sclerophyll woodland, riparian woodland, mallee, *Callitris* woodland and *Melaleuca* woodland. One WA worker from Karonie was collected by sweeping, suggesting this species may forage on low vegetation. Given its typical appearance and its environmental adaptability, this species is likely a generalized scavenger.

#### Etymology.

Latin *rufus* plus *niger* (‘red-and-black’); adjective in the nominative singular.

**Figure 19.**
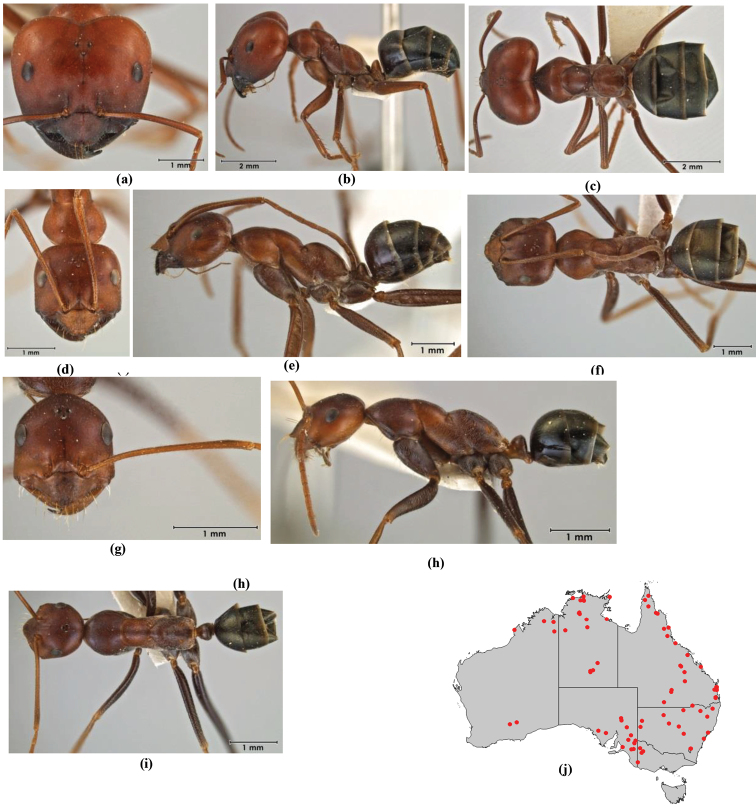
*Melophorus rufoniger* sp. n.: major worker paratype (ANIC32-066641–top ant) frons (**a**), profile (**b**) and dorsum (**c**); paratype media worker (ANIC32-046417) frons (**d**), profile (**e**) and dorsum (**f**); minor worker holotype (ANIC32-066663–bottom ant) frons (**g**), profile (**h**) and dorsum (**i**); distribution map for the species (**j**).

### Melophorus sulconotus

Taxon classificationAnimaliaHymenopteraFormicidae

Heterick, Castalanelli & Shattuck sp. n.

http://zoobank.org/42DEA22D-236E-417D-A5E3-007070B61E6B

#### Types.

Holotype minor worker (middle ant), Kapalga, Alligator Rivers area, Northern Territory, 7-9 September 1993, P.J.M. Greenslade, 8Aii traps, [ANIC32-900071] (ANIC). Paratypes: 2 minor workers on same pin and with same data as holotype (ANIC).

#### Other material examined.

**Western Australia**: 4 km E Lake Argyle (Heterick, B.E. [M219]).

#### Diagnosis.

*Melophorus sulconotus* is a member of the *M. aeneovirens* species-group (in full-face view, the anterior clypeal margin convex, apron-like and covering whole or part of the retracted mandible, except in *M. nemophilus*, the medial clypeal sector often produced so that it is protrusive when seen in profile; the psammophore frequently with coarse and well-separated ammochaetae, these always placed on or just above anterior margin; in profile, the propodeum elongate and oblique or broadly rounded), and the *M. aeneovirens* species-complex (in full-face view, psammophore ranged along or just above anterior margin of clypeus and following the curve of the margin; anterior margin of clypeus broadly medially produced, and often with central notch that may be deeply impressed, but is never acuminate at its midpoint; metatibia with maximum of two rows of preapical spines). In *M. sulconotus* the tibiae possess fine, appressed pubescence in addition to stout, socketed, appressed to subdecumbent setae No major worker was available for examination. The chief diagnostic feature in the minor worker is the flattened posterior pronotum and mesonotum which descends steeply to the metanotal groove. This species is restricted to the wetter Northern Kimberley distinct and the far north Northern Territory.

#### Minor worker description.

**Head.** Head square; posterior margin of head weakly convex; frons matt or with weak sheen, microreticulate or microreticulate-shagreenate; frons consisting of appressed pubescence, with many short, unmodified, erect setae. Eye small (eye length less than 0.2 × length of side of head capsule); in full-face view, eyes set above midpoint of head capsule; in profile, eye set anteriad of midline of head capsule; eyes elliptical or slightly reniform. In full-face view, frontal carinae straight or weakly convex; frontal lobes straight, elevated. Anteromedial clypeal margin broadly and evenly convex and protrusive; clypeal psammophore set at or just above anterior clypeal margin; palp formula 6,4. Five mandibular teeth in minor worker; mandibles triangular, weakly incurved; third mandibular tooth distinctly shorter than apical tooth and teeth numbers two and four; masticatory margin of mandibles approximately vertical or weakly oblique. **Mesosoma.** Integument of pronotum, mesonotum and mesopleuron matt or with weak sheen and microreticulate throughout; anterior mesosoma in profile smoothly rounded anteriad, thereafter pronotum and whole of mesonotum flattened and on same plane as propodeum; appearance of erect pronotal setae short, (i.e., longest erect setae shorter than length of eye) and unmodified; in profile, metanotal groove deep, ‘V’-shaped; propodeum matt or with a weak sheen and microreticulate; propodeum always smoothly rounded; propodeal dorsum and declivity confluent; erect propodeal setae present and abundant (greater than 12); appressed propodeal setulae minute and closely aligned, creating a silvery sheen; propodeal spiracle situated at least twice its width from the declivitous face of propodeum, and shorter (length < 0.50 × height of propodeum). **Petiole.** In profile, petiolar node squamiform; in full-face view, shape of petiolar node uniformly rounded; node shining and smooth with vestigial sculpture. **Gaster.** Gaster shining, shagreenate (‘LP record’ appearance); pilosity of first gastral tergite consisting of thick, appressed setae that form pubescence, interspersed with numerous short, bristly, erect setae. **General characters.** Colour of foreparts orange, legs light tan, gaster dark brown.

#### Measurements.

Worker (n = 2): CI 97–99; EI 19–20; EL 0.27–0.29; HL 1.38–1.55; HW 1.34–1.53; ML 2.24–2.46; MTL 1.47–1.58; PpH 0.22–0.26; PpL 0.86–0.93; SI 120–127; SL 1.71–1.84.

#### Comments.

This rare and rather attractive Torresian species in the *M. aeneovirens* species-group is known only from the minor and media workers. The profile is distinctive (noted under ‘Comments’ for *Melophorus rufoniger*). Genetic sequencing indicates the ant may be sister to *M. rufoniger*. Specimens collected from the Victoria Hwy roadside near Kununurra, Western Australia, decorated their nest with flattened pebbles much larger than the ants.

#### Etymology.

Latin *sulcus* (‘groove’) plus Neo-Latin *notus* (‘back’); adjective in the nominative singular.

**Figure 20.**
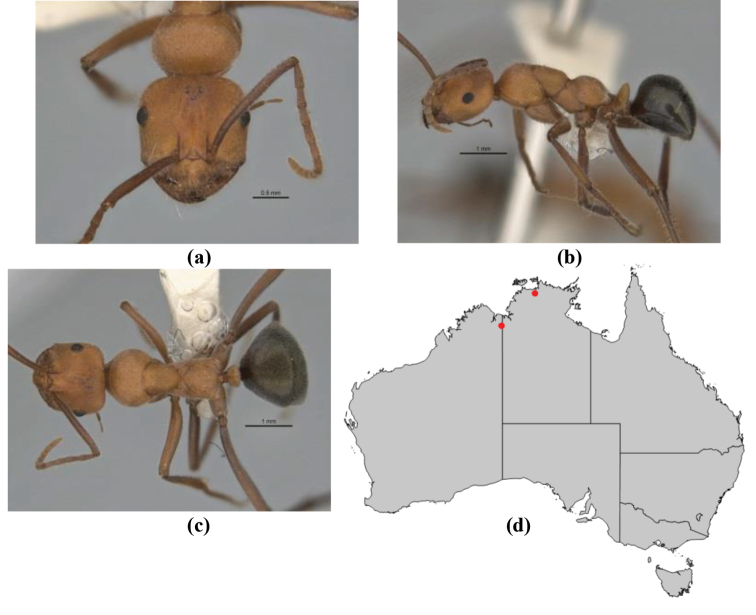
*Melophorus sulconotus* sp. n.: minor worker holotype (ANIC32-900071–middle ant) frons (**a**), profile (**b**) and dorsum (**c**); distribution map for the species (**d**). Low resolution scale bars: 1 mm (**b, c**); 0.5 mm (**a**).

### Melophorus tenuis

Taxon classificationAnimaliaHymenopteraFormicidae

Heterick, Castalanelli & Shattuck sp. n.

http://zoobank.org/293E535F-ABF4-4A50-8F28-C5C60F21EDF9

#### Type.

Holotype minor worker from 1.3 km NE of Morgan River, 15°08'10"S, 126°09'09"E, Western Australia, 20 August 2012, A. Cross; 345m elevation, ethylene glycol pitfall, open savannah [*sic*] woodland over sandy soil; sp. C [ANIC32-900210] (WAM).

#### Diagnosis.

*Melophorus tenuis* is a member of the *M. aeneovirens* species-group (in full-face view, the anterior clypeal margin convex, apron-like and covering whole or part of the retracted mandible, except in *M. nemophilus*, the medial clypeal sector often produced so that it is protrusive when seen in profile; the psammophore frequently with coarse and well-separated ammochaetae, these always placed on or just above anterior margin; in profile, the propodeum elongate and oblique or broadly rounded), and the *M. aeneovirens* species-complex (in full-face view, psammophore ranged along or just above anterior margin of clypeus and following the curve of the margin; anterior margin of clypeus broadly medially produced, and often with central notch that may be deeply impressed, but is never acuminate at its midpoint; metatibia with maximum of two rows of preapical spines). In *M. tenuis* the tibiae possess stout, socketed, appressed to subdecumbent setae only, with fine, appressed pubescence lacking. In profile, the mesosoma of the minor worker tends to linear in orientation, its dorsal outline straight or describing a weak arc (the mesosternal outline and the dorsum of the mesonotum being weakly convergent to subparallel anteriorly). Only the minor worker of *Melophorus tenuis* is known. This species can be distinguished from all others in its species-complex its dorsoventrally flattened head, its long maxillary palps (short in the superficially similar *M. platyceps*) and its weakly sinuous mesosomal outline when seen in profile.

#### Minor worker description.

**Head.** Head approximately oval, sides of head divergent towards mandibular insertions; posterior margin of head extended posteriad as a convex, sloping surface with a slight medioccipital protuberance; cuticle of frons with weak sheen, finely shagreenate; in single specimen pilosity of frons consisting exclusively of well-spaced, appressed setae only. Eye moderate (eye length 0.20–0.49 length of side of head capsule); in full-face view, eyes set above midpoint of head capsule; in profile, eyes set around midline of head capsule; eye shape elliptical. Antennal carinae straight in full-face view; antennal lobes curved toward antennal insertion. Anterior clypeal margin broadly and evenly convex; clypeal psammophore set at or just above anterior clypeal margin; palp formula 6,4. Five mandibular teeth in minor worker; mandibles triangular, weakly incurved; third mandibular tooth distinctly shorter than apical tooth and teeth numbers two and four; masticatory margin of mandibles approximately vertical or weakly oblique. **Mesosoma.** Integument of pronotum, mesonotum and mesopleuron moderately shining and shagreenate throughout; anterior mesosoma in profile flattened, promesonotum on same plane as propodeum; erect pronotal setae absent; in profile, metanotal groove shallow, indicated mainly by an angle; propodeum shining and shagreenate; propodeum elongate, smoothly rounded; propodeal dorsum and declivity confluent; erect propodeal setae always absent; appressed propodeal setulae short, separated by more than own length and completely inconspicuous; propodeal spiracle situated at least twice its width from the declivitous face of propodeum, and shorter (length < 0.50 × height of propodeum). **Petiole.** In profile, petiolar node subcuboidal, its vertex bluntly rounded; in full-face view, shape of petiolar node uniformly rounded; node shining and faintly shagreenate. **Gaster.** Gaster shining, shagreenate (‘LP record’ appearance); pilosity of first gastral tergite consisting of well-spaced short, inconspicuous, appressed setae only, erect setae always absent. **General characters.** Colour uniformly dark olive-brown.

#### Measurements.

Worker (n = 1): CI 82; EI 30; EL 0.25; HL 0.99; HW 0.82; ML 1.72; MTL 1.18; PpH 0.16; PpL 0.67; SI 168

#### Comments.

*Melophorus tenuis* has an appearance somewhat reminiscent of *Melophorus attenuipes* and *Melophorus mullewaensis*, but, although it is likely closely related to these taxa, it can be distinguished from them by the much flattened head and the appearance of the femur and clypeus. This ant has been collected from a number of sites in the far north of Australia, but only one minor worker was available for this project. TERC material from WA has been collected from King Edward River, Bachesten Creek, Purnululu NP, Parry Lagoon and the Mitchell Plateau. TERC also holds specimens from Bradshaw Stn, Barunga, Nitmuluk NP, Bullo Gorge and Kakadu in the NT.

Pitfall-trapped specimens collected by Adam Cross in Doongan and Theda Stations in the Kimberley were found in four study plots on shallow, sandstone-derived soils overlying Pentecost sandstone. The vegetation was open savannah woodland dominated by *Eucalyptus tetradonta* and *E. miniata* over shrubs and low annual grasses. Three of the four plots were near the Morgan River, and the fourth was on Noongallah Creek (A. Cross, pers. comm.). The holotype is one of the Morgan River specimens from that project.

#### Etymology.

Latin (‘thin’, ‘slender’); adjective in the nominative singular.

**Figure 21.**
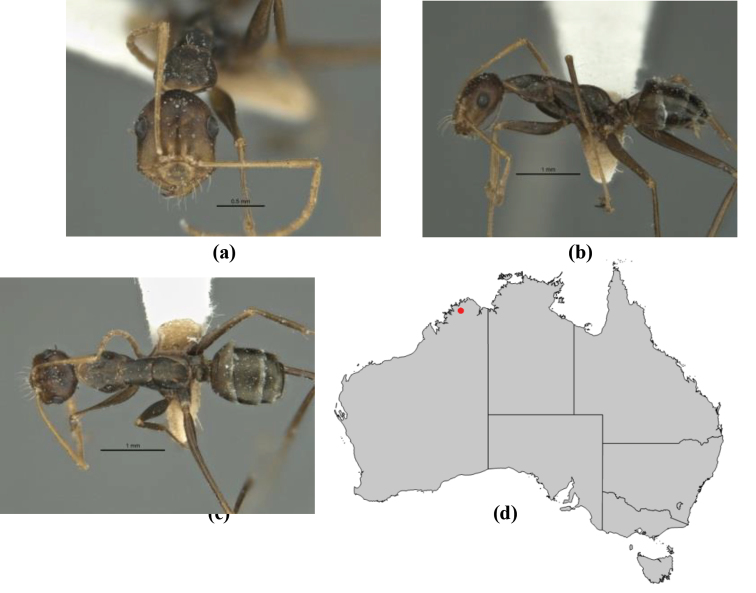
*Melophorus tenuis* sp. n.: minor worker holotype (ANIC32-900210) frons (**a**), profile (**b**) and dorsum (**c**); distribution map for the species (**d**). Low resolution scale bars: 1 mm (**b, c**); 0.5 mm (**a**).

### Melophorus teretinotus

Taxon classificationAnimaliaHymenopteraFormicidae

Heterick, Castalanelli & Shattuck sp. n.

http://zoobank.org/1EEBF09F-EFA6-47BF-91E9-EDCEF82871FE

#### Types.

Holotype minor worker (top ant) from Ethel Creek, Western Australia, February 1996, F. K. Sengarayar [JDM32-004552] (WAM). Paratypes: minor worker on same pin and with same data as holotype (WAM); minor worker from Barrow Island, 20°47'38'S, 115°36'24"E, Western Australia, 24 April, 2005, S. Callan, Suction RI pit AL [JDM32-004553] (ANIC).

#### Other material examined.

**Western Australia**: Argyle Diamond Mine via Kununurra (Postle, A. [JDM32-004554]), Barrow Island (N. Gunawardene/C. Taylor [M314]).

#### Diagnosis.

*Melophorus teretinotus* is a member of the *M. aeneovirens* species-group (in full-face view, the anterior clypeal margin convex, apron-like and covering whole or part of the retracted mandible, except in *M. nemophilus*, the medial clypeal sector often produced so that it is protrusive when seen in profile; the psammophore frequently with coarse and well-separated ammochaetae, these always placed on or just above anterior margin; in profile, the propodeum elongate and oblique or broadly rounded), and the *M. aeneovirens* species-complex (in full-face view, psammophore ranged along or just above anterior margin of clypeus and following the curve of the margin; anterior margin of clypeus broadly medially produced, and often with central notch that may be deeply impressed, but is never acuminate at its midpoint; metatibia with maximum of two rows of preapical spines). In *M. teretinotus* the tibiae possess stout, socketed, appressed to subdecumbent setae only, with fine, appressed pubescence lacking. The minor worker only is known. *Melophorus teretinotus* cannot be mistaken for any other *Melophorus*: the mesosoma is strongly arched, smooth and glabrous, the mesonotum and propodeum are confluent and the metanotal groove is completely lacking.

#### Minor worker description.

**Head.** Head square; posterior margin of head extended posteriad as a convex, sloping surface with a slight medioccipital protuberance; frons shining with superficial shagreenation or microreticulation only; frons consisting exclusively or almost exclusively of well-spaced, appressed setae only (small, erect setae, if present, usually confined to ocular triangle or posterior margin of head). Eye moderate (eye length 0.20–0.49 length of side of head capsule); in full-face view, eyes set above midpoint of head capsule; in profile, eye set anteriad of midline of head capsule; eyes elliptical or slightly reniform. In full-face view, frontal carinae concave; frontal lobes curved inward in front of antennal insertion. Anteromedial clypeal margin narrowly convex and protruding, clypeal midpoint distinctly notched; clypeal psammophore set at or just above anterior clypeal margin; palp formula 6,4. Five mandibular teeth in minor worker; mandibles triangular, weakly incurved; third mandibular tooth distinctly shorter than apical tooth and teeth numbers two and four; masticatory margin of mandibles approximately vertical or weakly oblique. **Mesosoma.** Integument of pronotum, mesonotum and mesopleuron moderately shining and shagreenate throughout; anterior mesosoma in profile broadly convex; erect pronotal setae absent; in profile, metanotal groove absent; propodeum shining and uniformly striolate; propodeum always smoothly rounded; propodeal dorsum and declivity confluent; erect propodeal setae always absent; appressed propodeal setulae short, separated by more than own length and inconspicuous; propodeal spiracle situated on or beside declivitous face of propodeum, and shorter (length < 0.50 × height of propodeum). **Petiole.** In profile, petiolar node squamiform; in full-face view, shape of petiolar node uniformly rounded; node shining and smooth throughout. **Gaster.** Gaster shining, shagreenate (‘LP record’ appearance); pilosity of first gastral tergite consisting of well-spaced short, inconspicuous, appressed setae only, erect setae always absent. **General characters.** Colour brown, gaster slightly darker than foreparts.

#### Measurements.

Worker (n = 4): CI 82–85; EI 26–30; EL 0.21–0.25; HL 0.83–1.16; HW 0.68–0.98; ML 1.38–1.64; MTL 0.62–0.79; PpH 0.10–0.17; PpL 0.68–1.04; SI 127–149; SL 1.02–1.25.

#### Comments.

The odd outline of the minor worker of this species makes it impossible to confuse it with any other *Melophorus*. The absence of the metanotal groove is distinctive, and the shining, broadly expansive pronotum and mesonotum smoothed over into a deeply declivitous propodeum brings to one’s mind the curve of a snail shell. Only the minor worker has so far been recognized. All records have come from WA, where it appears occasionally in collections from the Kimberley and Pilbara region on the mainland and from Barrow Island, but the species is also likely to occur in the NT. The ecology of this ant is not known. Attempts to sequence its DNA have achieved only limited success with only the wingless gene successfully amplifying. Its closest relation is *M. praesens*, based on pairwise divergence of the Wg gene (1.8% pairwise divergence).

#### Etymology.

Latin *terete* (‘cylindrical and tapering’) plus Neo-Latin *notus* (‘back’); adjective in the nominative singular.

**Figure 22.**
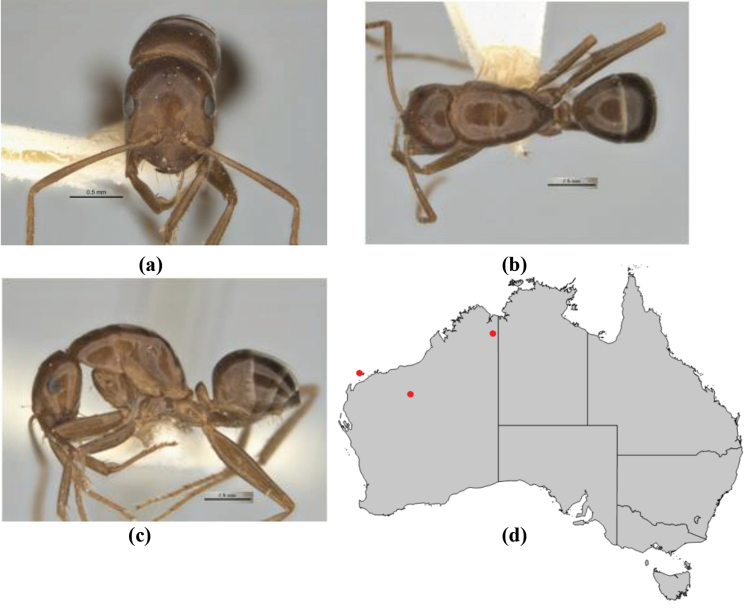
*Melophorus teretinotus* sp. n.: minor worker holotype (JDM32-004552) frons (**a**), profile (**b**) and dorsum (**c**); distribution map for the species (**d**). Low resolution scale bars: 0.5 mm (**a–c**).

### *Melophorus bagoti* complex

This complex consists of two widespread and spectacular, large *Melophorus* that are often encountered in drier habitats throughout Australia. *Melophorus bagoti* has attracted a lot of research interest as its habits and its appearance make it a relatively easy research subject compared with smaller, less easily identifiable species. The complex is identifiable by the conformation of the clypeus and the appearance of the clypeal psammophore.

The five-gene phylogenetic trees reveal good branch support for the *M. bagoti* complex as a monophyletic group and as sister to the *M. nemophilus* complex (see figures 3 and 4, respectively). In respect of *M. bagoti*, intraspecific pairwise divergences for the genes COI and H3 were considerably higher than for all other *Melophorus* species, the difference being attributed to sample M143. This specimen has an iridescent black gaster (orange or greyish-orange and non-iridescent for the other samples). Despite the high pairwise divergences for the COI and H3 genes, M143 not was not split from the other *bagoti*, being monophyletic within the remaining samples, and the Wg gene shows it fitting comfortably into the clade (next to the more typical M220) that includes the sequenced samples for this species. More specimens are needed before there is any thought of assigning this spectacular form a new species status.

### Melophorus bagoti

Taxon classificationAnimaliaHymenopteraFormicidae

Lubbock

Melophorus bagoti Lubbock 1883: 52, pl. 2, figs 1–10. Type. Syntype major worker without an original label [BMNH] (examined: AntWeb image of BMNH specimen BMNH(E)1016283, CASENT0903260).
Camponotus cowlei Froggatt. Froggatt, W. W. (1896). Honey ants. pp. 385–392 In: Spencer B (Ed.) Report on the work of the Horn Scientific Expedition to Central Australia. Melbourne: Melville, Mullen & Slade Part 2 Zoology. [p. 387 pl. 27] (combination as Melophorus cowlei by Wheeler 1908:388; combination as Camponotus (Myrmophyma) cowlei by Emery 1925:110; junior synonym of Melophorus bagoti by Clark 1930:22; combination and valid species as Camponotus cowlei by Taylor and Brown 1985:112). Types. Syntype major workers (assumed) and queen on separate pins, without collection data [AMS]. [Not seen] Syntype worker(s), queen(s), male(s), Spencer Gorge, McDonnell Ranges, Northern Territory [museum unknown]. **Rev. comb.** (Note. The material includes the discoloured head and pronotum of a major worker and a worker gaster that have been glued to a newish-looking card rectangle, but the gaster does not appear to belong to that pin as it is shining and new in appearance, and may not even be that species. All three ants are labeled with their caste, surname and initials of Froggatt and date (1895) (label 1), and the scientific name (‘Camponotus cowlei Froggatt’) (label 2). There is no mention of the collection locality. A gelatin capsule holds broken fragments of legs.)

#### Other material examined.

**New South Wales**: 40 km NNW Louth, Lake Mere (Greenslade, P.J.M.), 40 km NNW Louth, Lake Mere (Greenslade, P.J.M.), CSIRO Lake Mere Field Station, north Louth (Bryannah, M.), Sturt National Park (Greenslade, P.J.M.). **Northern Territory**: 18 km E The Granites (Morton, S. & Greenslade, P.J.M.), 25 km N Alice Springs (Shattuck, S.O.), 25 km SSW The Granites, Tanami Desert (Morton, S. & Greenslade, P.J.M.), 35 km SW The Granites, Tanami Desert (Morton, S. & Greenslade, P.J.M.), 28 km WbyS Docker River (Feehan, J.E.), Glen Helen (Shattuck, S.O.), 61.5 km E Kalkarindji (Heterick, B.E. [M220]), Avon Downs (Colles, D.H.), Barry Caves (Feehan, J.E.), Batchelor (Hill, G.F. [ANIC32-039541]), Ellery Ck., Big Hole, West Macdonnells NP (Shattuck, S.O. [ANIC32-029215]), Kings Creek Station (Donellan, S.), nr Limbunya turnoff (Heterick, B.E. [M224]), Mataranka (Greaves, T), Rabbit Flat near Tanami (Wearne, K), Ti Tree, 170km N Alice Springs (Hiddins, L.). **Queensland**: ‘Gumbardo’ (Beutel, T.), ‘Gumbardo’ (Beutel, T.), ‘Gumbardo’ (Beutel, T.), ‘Gumbardo’ (Beutel, T.), Holts Creek, 8 km N Musselbrook Camp (Naumann, I. D.), ‘Merigol’ (Beutel, T.), ‘Merigol’ (Beutel, T.), Mica Creek, Mount Isa (Burwell, C. J.), 13 km from S-bend on Plum Pudding Track (Lemann, C. [ANIC32-035469]), 15 mi S Kamilaroi Homestead (Dowse, J.E.), Mt. Isa (Weatherill, L.). **South Australia**: 14.5 km W Wallatinna HS (Pitjantjatjara Lands Survey [M89/M105]), 18 mi W Mt. Morris (McInnes & Dowse), 25 mi W Mt. Morris (McInnes & Dowse). **Western Australia**: 10 km NE Woree Hill (Heterick, B.E. [M180/M181/M182]), 11 km E Willare Bridge (Heterick, B.E. [M246]), 12 mi N Wittenoom (McInnes & Dowse), 12 mi S Cardawan Homestead, SSW Mundiwindi (McInnes & Dowse [ANIC32-900069]), 14 km E Roebuck Plains RH (Heterick, B.E. [M187]), 150 km SW Giles Meteorological Station (Heatwole, H.), 16 km SbyW Onslow (Feehan, J.E.), 26 mi NE Paynes Find on Sandstone Road (Douglas, A.M. & M.J.), 27 km SE Newman (Britton, E.B.), 34 mi ENE Broome (McInnes & Dowse), 47 km E Fitzroy Crossing (Heterick, B.E. [M235]), 50 km N Carnarvon (Morton, S.R. [ANIC32-900070]), 56 mi WSW Mt. Gordon, Browne Range (McInnes, R. & Dowse, J.), 68 km S Newman (Britton, E.B.), 70 km N Halls Creek (Heterick, B.E. [M211]), 74 km EbyN Cosmo Newberry (Feehan, J.E.), 85 km E Meentheena OC (CALM Pilbara Survey [JDM32-004588]), Alpha Is., Monte Bello Islands (Campbell, T.G. [ANIC32-039538]), Argyle Diamonds via Kununurra (Postle, A.T. [JDM32-004532]), Broome (Weatherill, L.), Derby (Campbell, J.G.), Derby (Marchant [JDM32-004536]), Garden Well, 9 km SWbyS Mt. Phoenix (Feehan, J.E.), Jigalong (Hickmer, J.), Little Sandy Desert (Guthrie N. A. [M143]), Marillana Station (Dunlop, J.N. [JDM32-004535]), Nita Downs turnoff (Heterick, B.E. [M262]), Tropicana Minesite (Summerhayes, J. [JDM32-004587]), Well 31, Canning Stock Route between Meekatharra and Billiluna Pool [Collector unknown], Willare Bridge (Heterick, B.E. [M190]), Willie Creek turnoff (Heterick, B.E. [M276]), Windjana Gorge National Park (Ward, P.S.).

#### Diagnosis.

*Melophorus bagoti* is a member of the *M. aeneovirens* species-group (in full-face view, the anterior clypeal margin convex, apron-like and covering whole or part of the retracted mandible, except in *M. nemophilus*, the medial clypeal sector often produced so that it is protrusive when seen in profile; the psammophore frequently with coarse and well-separated ammochaetae, these always placed on or just above anterior margin; in profile, the propodeum elongate and oblique or broadly rounded). The ant is also placed as a member of the *M. bagoti* complex because of the acuminate appearance of the midpoint of the anterior clypeal margin. In full-face view, the psammophore occurs as a row of long, thick setae set slightly above the anterior clypeal margin. *Melophorus bagoti* has five rows of preapical tibial spines on the metatibia, and this distinguishes it from its sister, *M. gracilipes*, and all other *Melophorus*.

#### Minor worker description.

**Head.** Head square or rectangular, tending to trapezoid; posterior margin of head extended posteriad as a convex, sloping surface with a slight medioccipital protuberance; frons shining with superficial shagreenation or microreticulation only; frons consisting exclusively or almost exclusively of well-spaced, appressed setae only (small, erect setae, if present, usually confined to ocular triangle or posterior margin of head). Eye moderate (eye length 0.20–0.49 length of side of head capsule); in full-face view, eyes set above midpoint of head capsule; in profile, eye set anteriad of midline of head capsule; eyes elliptical or slightly reniform. In full-face view, frontal carinae straight or weakly convex; frontal lobes straight in front of antennal insertion. Anteromedial clypeal margin convex, weakly acuminate anteromedially; clypeal psammophore set at or just above anterior clypeal margin; palp formula 6,4. Five to six mandibular teeth in minor worker; mandibles triangular, weakly incurved; third mandibular tooth distinctly shorter than apical tooth and teeth numbers two and four; masticatory margin of mandibles approximately vertical or weakly oblique. **Mesosoma.** Integument of pronotum, mesonotum and mesopleuron uniformly shagreenate to moderately shining and shagreenate throughout; anterior mesosoma in profile broadly convex; erect pronotal setae absent; in profile, metanotal groove shallow, broadly V or U-shaped; propodeum shining and shagreenate; propodeum smoothly rounded or with indistinct angle or bluntly angulate; length ratio of propodeal dorsum to its declivity about 2:1; propodeal dorsum and declivity confluent; erect propodeal setae always absent; appressed propodeal setulae short, separated by more than own length and inconspicuous; propodeal spiracle situated at least twice its width from the declivitous face of propodeum, and shorter (length < 0.50 × height of propodeum). **Petiole.** In profile, petiolar node a broadly right-angled triangle, node with steeply declivitous posterior face; in full-face view, shape of petiolar node uniformly rounded; node shining and smooth throughout. **Gaster.** Gaster smooth and glossy or shining, shagreenate (‘LP record’ appearance); pilosity of first gastral tergite consisting of well-spaced short, inconspicuous, appressed setae, erect setae (present in at least some workers) confined to margin of sclerite. **General characters.** Colour mostly uniformly deep orange, but some workers with foreparts and appendages orange, and gaster black with blue-green iridescence.

#### Major worker description.

**Head.** Head horizontally rectangular, broader than wide; posterior margin of head planar or weakly convex; cuticle of frons ranging from matt or with weak sheen, indistinctly shagreenate through to shining with superficial shagreenation or microreticulation; pilosity of frons a mixture of a few well-spaced, erect setae interspersed with appressed setae only. Eye moderate (eye length 0.20–0.49 length of head capsule); in full-face view, eyes set above midpoint of head capsule; in profile, eye set anteriad of midline of head capsule; eyes elliptical. In full-face view, frontal carinae straight or weakly convex; frontal lobes straight in front of antennal insertion. Anterior clypeal margin convex, acuminate anteromedially, margin entire; clypeal psammophore set at or just above anterior clypeal margin; palp formula 6,4. Five to six mandibular teeth in major worker; mandibles triangular, weakly incurved; third mandibular tooth distinctly shorter than apical tooth and teeth numbers two and four; masticatory margin of mandibles approximately aligned vertically or weakly oblique. **Mesosoma.** Integument of pronotum, mesonotum and mesopleuron moderately shining and shagreenate throughout; anterior mesosoma in profile broadly convex; erect pronotal setae long (i.e., longer than length of eye) and unmodified; in profile, metanotal groove shallow, broadly V- or U-shaped; propodeum shining and shagreenate, or matt or with a weak sheen and microreticulate; propodeum angulate, propodeal angle blunt; length ratio of propodeal dorsum to its declivity between 3:2 and 4:3; erect propodeal setae absent; appressed propodeal setae short, separated by more than own length and inconspicuous; propodeal spiracle situated at least twice its width from the declivitous face of propodeum, and shorter (length less than 0.50 × height of propodeum). **Petiole.** In profile, petiolar node a broadly right angled triangle, node with steeply declivitous posterior face; in full-face view, shape of petiolar node generally rounded with median indentation; node shining and faintly shagreenate-microreticulate. **Gaster.** Gaster shining, shagreenate (‘LP record’ appearance); pilosity of first gastral tergite consisting of well-spaced short, inconspicuous, appressed setae, erect setae (present in at least some workers) confined to margin of the sclerite. **General characters.** Colour as for minor worker.

#### Measurements.

Worker (n = 8): CI 99–118; EI 16–22; EL 0.30–0.52; HL 1.41–2.81; HW 1.39–3.33; ML 2.66–4.59; MTL 1.91–3.01; PpH 0.32–0.47; PpL 1.23–1.73; SI 81–147; SL 2.04–2.71.

#### Comments.

*Melophorus bagoti* is the *Melophorus* with which the average layperson from the drier rural areas is probably best acquainted because of its bright colour and its size. The size alone makes this species unmistakable except for *M. gracilipes*, from which it can be distinguished by the presence of five rows of tibial spurs (compared with the normal two rows in the latter species). *Melophorus bagoti* has been recorded from all mainland Australian states except Victoria, but appears to be most common in the NT and WA. A number of samples of the taxon have been sequenced, and these reveal a monophyletic group (see above discussion of the complex).

Syntype specimens (at least two) for ‘*Camponotus cowlei*’ have been seen. Although very badly damaged, sufficient of the ants remain, including the all-important head capsule and spinous tibiae, for the identity of the species not to be in doubt. ‘*Camponotus cowlei*’ therefore reverts to its previous synonym under the genus *Melophorus*.

As discussed in the Introduction, this ant has been the focus of a number of studies that look at orientation in desert ants, but its thermophilic aspects and nest structure have also gained attention (see references in the Introduction): along with *Melophorus perthensis*, this is the best known and most thoroughly researched *Melophorus*. In drier regions *M. bagoti* workers are frequently seen scurrying rapidly over the ground surface foraging for seeds and carrion. In the Kimberley, the principal author also saw many workers climbing over a small shrub when it was in flower, seeking nectar.

**Figure 23.**
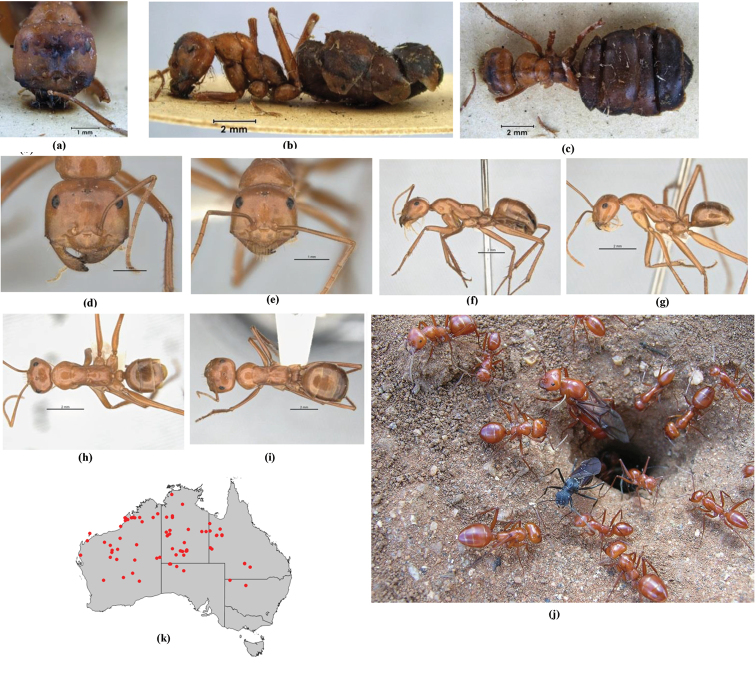
*Melophorus bagoti* Lubbock: BMNH major worker syntype (CASENT0903260) frons (**a**), profile (**b**) and dorsum (**c**); non–type major worker (ANIC3-900190–top ant) frons (**d**), profile (**f**) and dorsum (**i**); non–type minor worker (ANIC3-900190–bottom ant) frons (**e**), profile (**g**) and dorsum (**h**); *Melophorus bagoti* workers and reproductives at nest entrance (photo credit: A. J. Narendra) (**j**); distribution map for the species (**k**). Low resolution scale bars: 2 mm (**f, g, h, i**); 0.5 mm (**d, g**).

### Melophorus gracilipes

Taxon classificationAnimaliaHymenopteraFormicidae

Heterick, Castalanelli & Shattuck sp. n.

http://zoobank.org/F944B84B-6574-443C-8C68-8BE3DCFDAAD3

#### Types.

Holotype minor worker (bottom ant-slightly damaged) from Yamarna Homestead 28°10'S, 123°40'E, Western Australia, 16 December 1996-30 January 1997, S. Richardson, Acacia woodland with spinifex, sandy loam [ANIC32- 032240] (ANIC). Paratypes: major worker and media worker on same pin and with same details as holotype (ANIC); 2 media workers from 55 miles E of Wiluna, Western Australia, 10 October 1969, McInnes & Dowse (ANIC); major worker and 2 minor workers from Murchison River, Western Australia, ‘*Melophorus bagoti* Lubbock, Det. J.J. McAreavey, 7 January 1960 [*sic*] [these specimens can easily be distinguished from *M. bagoti* by the the two rows of spines on the metatibia-BEH] (BMNH); 2 media workers from 10 km SE Karonie, Western Australia, 9 November 1969, Key’s field notes. Trip 163, Stop 19405.6, ground strays, evening, sclerophyll wood., R.W. Taylor (MCZ); Major worker and minor worker from 30 km S of ‘The Overlander’, Western Australia, 20 March 1987, B. Heterick, soil, native vegetation, rural environment, 183, 8*Mel*BH1 (WAM); 2 minor workers from Rabbit Proof Fence Road 30°26'57"S, 118°31'13"E, Western Australia, 21 November 2009, Heterick, B.E., Laterite sandplain, proteaceous heathland with small trees , by hand, early pm [JDM32-004618] (WAM).

#### Other material examined.

**Western Australia**: 100 km E Wiluna (Forrest, J.), 10 mi SE Karonie (Taylor, R.W.), 11 mi E Meekatharra (McInnes & Dowse), 13 mi SE Giles (McInnes & Dowse), 16 mi S Leonora (McInnes & Dowse), 16 mi W Coonana (Key, K.), 18 mi SE Agnew (McInnes & Dowse), 30 km S of ‘The Overlander’ (Heterick, B.E. [JDM32-001951]), 30 mi E Paynes Find on Sandstone Road (Douglas, A.M. & M.J.), 31 mi SW Mundiwindi (McInnes & Dowse), 4 mi W Mt Charles (McInnes & Dowse), 55 mi E Wiluna (McInnes & Dowse), 60 km N Ajana (Upton, M.S.), 7 mi WSW Manunda Road House (McInnes & Dowse), Ethel Creek (Varris, P.A.), G. J. Rd, 108 km E Carnarvon (Heterick, B.E. [M310]), Geeraning Rock (Heterick, B.E. [JDM32-001950]), Kanka water hole, near Warburton (Heatwole, H. & Greenslade, P.J.M.), Lyndon River (Mercer, R.), Mardathuna Rd turnoff (Heterick, B.E. [M08]), Meekatharra (Mercovich, C.), Mulga, NE Goldfields (Pringle, H.J.R. [ANIC32-029629]), Mullewa, Murchison River (Greaves, T.), nr Pamellia Hill (Heterick, B.E. [M178]), Pannawonica Hill (Bokhari, F. [M150]), Rabbit Proof Fence Rd. (Heterick, B.E. [JDM32-004618]), Sandstone Rd turnoff (Heterick, B.E.[M168/M169/M170]), Tardun (Mercovich, C.), Wiluna, Yamarna Homestead (Richardson, S.).

#### Diagnosis.

*Melophorus gracilipes* is a member of the *M. aeneovirens* species-group (in full-face view, the anterior clypeal margin convex, apron-like and covering whole or part of the retracted mandible, except in *M. nemophilus*, the medial clypeal sector often produced so that it is protrusive when seen in profile; the psammophore frequently with coarse and well-separated ammochaetae, these always placed on or just above anterior margin; in profile, the propodeum elongate and oblique or broadly rounded). The ant is also placed as a member of the *M. bagoti* complex because of the acuminate appearance of the midpoint of the anterior clypeal margin. In full-face view, the psammophore occurs as a row of long, thick setae set slightly above the anterior clypeal margin. Unlike its sister species, *M. bagoti*, *M. gracilipes* has just two rows of preapical tibial spines on the metatibia.

#### Minor worker description.

**Head.** Head approximately oval, sides of head divergent towards mandibular articulations; posterior margin of head extended posteriad as a convex, sloping surface with a slight medioccipital protuberance; frons matt or with weak sheen, microreticulate or microreticulate-shagreenate; frons consisting exclusively or almost exclusively of well-spaced, appressed setae only (small, erect setae, if present, usually confined to ocular triangle or posterior margin of head). Eye moderate (eye length 0.20–0.49 length of side of head capsule); in full-face view, eyes set above midpoint of head capsule; in profile, eye set anteriad of midline of head capsule; eyes elliptical or slightly reniform. In full-face view, frontal carinae straight or weakly convex, or straight, convergent posteriad; frontal lobes curved toward antennal insertion. Anteromedial clypeal margin convex, weakly acuminate anteromedially; clypeal psammophore set below midpoint of clypeus, or set at or just above anterior clypeal margin; palp formula 6,4. Five to six mandibular teeth in minor worker; mandibles triangular, weakly incurved; third mandibular tooth distinctly shorter than apical tooth and teeth numbers two and four; masticatory margin of mandibles approximately vertical or weakly oblique. **Mesosoma.** Integument of pronotum, mesonotum and mesopleuron moderately shining and shagreenate throughout; anterior mesosoma in profile convex anteriad, mesonotum often slightly overlapping pronotum, mesosoma planar or slightly sinuate posteriad; erect pronotal setae absent; in profile, metanotal groove shallow, broadly V or U-shaped; propodeum shining and uniformly striolate; propodeum always smoothly rounded; propodeal dorsum and declivity confluent; erect propodeal setae always absent; appressed propodeal setulae short, separated by more than own length and inconspicuous; propodeal spiracle situated at least twice its width from the declivitous face of propodeum, and shorter (length < 0.50 × height of propodeum). **Petiole.** In profile, petiolar node a broadly right angled triangle with steeply declivitous posterior face; in full-face view, shape of petiolar node uniformly rounded, or square with rounded angles; node shining and faintly striolate and microreticulate. ***Gaster*. **Gaster shining, shagreenate (‘LP record’ appearance); pilosity of first gastral tergite consisting of well-spaced short, inconspicuous, appressed setae only, erect setae always absent. **General characters.** Colour variable, pale yellow to brown.

#### Major worker description.

**Head.** Head square; posterior margin of head planar or weakly convex; cuticle of frons matt or with weak sheen, microreticulate; frons consisting exclusively or almost exclusively of well-spaced, appressed setae only (small, erect setae, if present, usually confined to ocular triangle or posterior margin of head). Eye small (eye length less than 0.2 × length of head capsule); in full-face view, eyes set above midpoint of head capsule; in profile, eye set anteriad of midline of head capsule; roughly ovoid, eye narrowed posteriad. In full-face view, frontal carinae straight or weakly convex, or straight, convergent posteriad; frontal lobes straight in front of antennal insertion, or curved inward in front of antennal insertion. Anterior clypeal margin convex, acuminate anteromedially, margin entire; clypeal psammophore set below midpoint of clypeus, or set at or just above anterior clypeal margin; palp formula 6,4. Five mandibular teeth in major worker; mandibles triangular, weakly incurved; third mandibular tooth distinctly shorter than apical tooth and teeth numbers two and four; masticatory margin of mandibles approximately aligned vertically or weakly oblique. **Mesosoma.** Integument of pronotum, mesonotum and mesopleuron with weak to moderate sheen, shagreenate on pronotum and dorsum of mesonotum, otherwise microreticulate; anterior mesosoma in profile broadly convex, or convex anteriad, mesonotum overlapping pronotum, planar or slightly sinuate posteriad; erect pronotal setae absent; in profile, metanotal groove shallow, broadly V- or U-shaped; propodeum shining and finely striolate and microreticulate; propodeum angulate, propodeal angle blunt; length ratio of propodeal dorsum to its declivity between 1:1 and 1:2; erect propodeal setae absent; appressed propodeal setae short, separated by more than own length and inconspicuous; propodeal spiracle situated at least twice its width from the declivitous face of propodeum, and shorter (length less than 0.50 × height of propodeum). **Petiole.** In profile, petiolar node narrowly conical, vertex sharply defined, or a broadly right angled triangle; in full-face view, shape of petiolar node generally rounded with median indentation; node shining and faintly striolate and microreticulate. **Gaster.** Gaster shining with superficial microreticulation; pilosity of first gastral tergite consisting of well-spaced short, inconspicuous, appressed setae only, erect setae always absent. **General characters.** Colour concolorous light orange or russet with brown gaster.

#### Measurements.

Worker (n = 8): CI 101–120; EI 15–21; EL 0.29–0.44; HL 1.36–2.54; HW 1.38–3.04; ML 2.44–4.11; MTL 2.47–2.84; PpH 0.21–0.47; PpL 1.04–1.70; SI 89–153; SL 2.11–2.69.

#### Comments.

Superficially, this large species resembles large members of the *M. aeneovirens* complex, but the appearance of the clypeus is sufficient to place it in the *M. bagoti* complex, and genetic sequencing data confirms this placement. The ant can easily be distinguished from *M. bagoti* by its more gracile appearance and the presence of two rows of preapical spines on the metatibia (five rows in *M. bagoti*). As with *M. bagoti*, the colour of the gaster in this taxon can vary from yellowish orange and concolorous with the rest of the ant to an iridescent black, with various shades in between. (In the case of *M. bagoti*, nest series of the most strikingly bicoloured ants seem to be of the one colouration, but workers of other nests can nonetheless show variation in colour between light orange gaster and blackish-orange gaster morphs). A specimen of *M. gracilipes* collected in the Pilbara had the iridescent black gaster, but unfortunately the genetic similarity of this form compared with other, lighter-coloured morphs could not be tested due to poor DNA quality.

*Melophorus gracilipes* is definitely an ant of the arid and semi-arid areas, and is absent from the wetter parts of the southern coastline. On the mainland all records have come from WA, although it likely also occurs in western parts of the NT and SA. As with other common species in the *M. aeneovirens* species-group, *M. gracilipes* has been found in a wide range of phytogeographic zones including hummock grasslands, mulga woodlands, acacia woodlands with spinifex, and sclerophyll woodlands. Red clay soil seems to be a favoured substrate for its nests. This species is a scavenger: a couple of workers hand collected near Pamellia Hill, WA, were carrying a dead grasshopper.

#### Etymology.

Latin *gracilis* (‘slender’) plus *pes* (‘foot’); adjective in the nominative singular.

**Figure 24.**
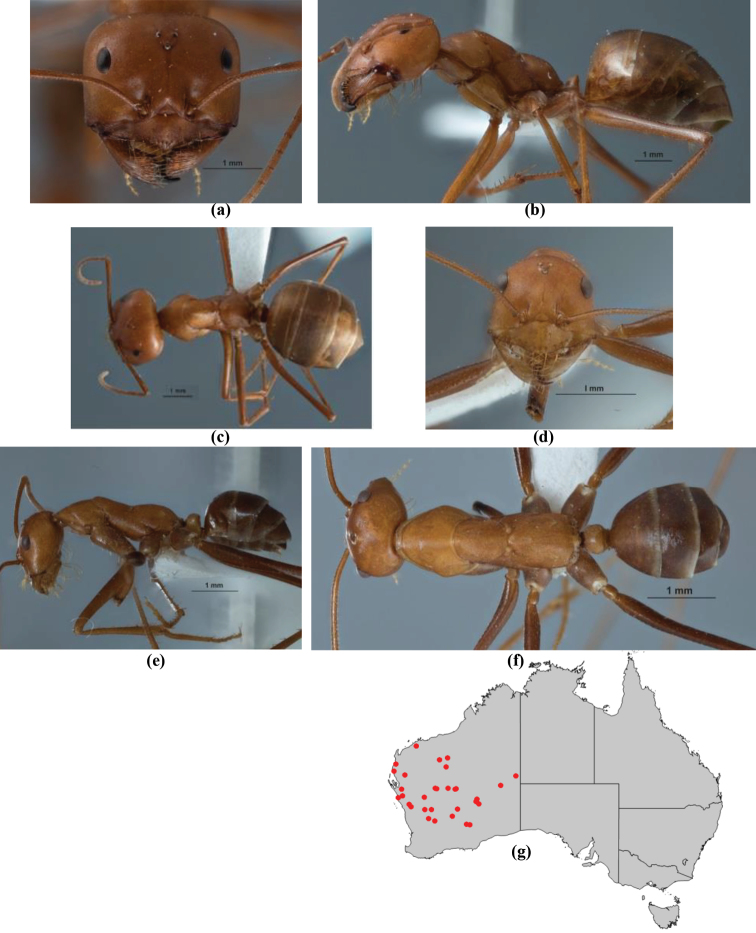
*Melophorus gracilipes* sp. n.: major worker paratype (ANIC32-032240–top ant) frons (**a**), profile (**b**) and dorsum (**c**); minor worker (ANIC32-032240–bottom ant) holotype frons (**d**), profile (**e**) and dorsum (**f**); distribution map for the species (**g**).

### *Melophorus nemophilus* complex

The *Melophorus nemophilus* complex consists of one temperate area species that forages on trees as well as the ground. The high placement of the clypeal psammophore makes it unique among its nearest relatives. Phylogenetically the *M. nemophilus* complex is monophyletic and sister to the *M. bagoti* complex (see Figure 3).

### Melophorus nemophilus

Taxon classificationAnimaliaHymenopteraFormicidae

Heterick, Castalanelli & Shattuck sp. n.

http://zoobank.org/FC2E38ED-F908-46AF-BCE9-2C9261D00198

#### Types.

Holotype media worker (top ant) from 17 miles N of Colona HS, South Australia, 23 October 1960, McInnes & Dowse, Series A 336 [ANIC32-900077] (ANIC). Paratypes: 2 major workers on same pin and with same details as holotype (ANIC); 3 minor workers from 31.1 km S of Shearer’s Quarters 141°01'40"S, 34°50'30"E, Millewa South Bore Track, Murray Sunset National Park, Victoria, 14-23 November 2002, C. Lambkin, D. Yeates, N. Starwick & J. Recsei, 2m Sharkey malaise opening in closed mallee [ANIC32-043297] (ANIC); major worker, 2 media workers and a minor worker from 15 km WNW Yaapeet 32°42'S, 141°52'E, Victoria, 13 January 1980, 1500 hrs, A. N. Andersen, *Melophorus* (BMNH); 2 minor workers from Cambrai, South Australia, 4 February 1972, P.J.M. Greenslade, dune IIb (MCZ); minor worker from 10 km E Mt Ive HS, Gawler Ranges, South Australia, 21 October 1980, P.J.M. Greenslade, (1) (MCZ); minor worker from 10 km E Mt Ive HS, Gawler Ranges, South Australia, 22 October 1980, P.J.M. Greenslade, A2 Sa (SAM); media worker from Cambrai, South Australia, 20 January 1972, P.J.M. Greenslade, dune III (SAM); minor worker from Junana Rock 33.23S, 123.24E, 9 km NW of Mt Ragged, Western Australia, 26 October 1977, R.W. Taylor, 77.667 day strays (WAM).

#### Other material examined.

**South Australia**: Callanna Bore, Callanna Stn (Stony Desert survey), Cambrai (Greenslade, P.J.M.), Cambrai (Greenslade, P.J.M.), Cambrai (Greenslade, P.J.M.), Ferries McDonald NP (Mathews, E. G [M116]), Keith, 5 km W Mt. Rough, Coorong (Greenslade, P.J.M. [ANIC32-900078]), near Mt. Westall (Taylor, R.W., Bartell, R.J. & Lowery, B.B.), Poochera (Taylor, R.W. & Bartell, R.J.), Scorpion Springs CP, 6.3 km S Pine Hut Soak (Forrest, J. [M91]), Wudinna townsite (Heterick, B.E. [M334]). **Victoria**: Little Desert Nat. Pk., Eastern Block, Dahlenburgs Mill Track, 17.8 km S Nhill (Lambkin, C., Yeates, D., Starwick, N. & Recsei, J. [ANIC32-030801]), Wyperfeld National Park, Murrayville Track, 45.2 km SSE Murrayville (Lambkin, C., Yeates, D., Starwick, N. & Recsei, J. [ANIC32-030935]). **Western Australia**: 28 mi SbyW Balladonia Motel (Taylor, R.W.); media worker from Junana Rock (Taylor, R.W.).

#### Diagnosis.

*Melophorus nemophilus* is a member of the *M. aeneovirens* species-group (in full-face view, the anterior clypeal margin convex, apron-like and covering whole or part of the retracted mandible, except in *M. nemophilus*, the medial clypeal sector often produced so that it is protrusive when seen in profile; the psammophore frequently with coarse and well-separated ammochaetae, these always placed on or just above anterior margin; in profile, the propodeum elongate and oblique or broadly rounded) but is placed in its own, monotypic species-complex. Uniquely for this group, the setae of the clypeal psammophore are fine and placed at around the midpoint of clypeus, and the anterior margin of the clypeus is a moderately flattened curve in all workers and does not cover the base of the mandibles. The ant has five to seven mandibular teeth, the major worker having the same mandibular structure as minor worker, and, in profile, the mesosoma is long and gracile, with an obliquely descending propodeum. Molecular data support the placement of *M. nemophilus* as belonging to a unique complex within the broader *M. aeneovirens* species-group.

#### Minor worker description.

**Head.** Head rectangular; posterior margin of head strongly convex; frons matt or with weak sheen, shagreenate; frons consisting exclusively or almost exclusively of well-spaced, appressed setae only (small, erect setae, if present, usually confined to ocular triangle or posterior margin of head). Eye moderate (eye length 0.20–0.49 length of side of head capsule); in full-face view, eyes set above midpoint of head capsule; in profile, eye set around midline of head capsule; eyes elliptical or slightly reniform. In full-face view, frontal carinae straight or weakly convex; frontal lobes straight in front of antennal insertion. Anteromedial clypeal margin broadly and evenly convex; clypeal psammophore set at or above midpoint of clypeus; palp formula 6,4. Five to seven mandibular teeth in minor worker; mandibles triangular, weakly incurved; third mandibular tooth distinctly shorter than apical tooth and teeth numbers two and four; masticatory margin of mandibles approximately vertical or weakly oblique. **Mesosoma.** Integument of pronotum, mesonotum and mesopleuron moderately shining and shagreenate throughout; anterior mesosoma in profile convex anteriad, mesonotum often slightly overlapping pronotum, mesosoma planar or slightly sinuate posteriad; erect pronotal setae absent; in profile, metanotal groove shallow, broadly V or U-shaped; propodeum shining and shagreenate; propodeum smoothly rounded or with indistinct angle; propodeal dorsum and declivity confluent; erect propodeal setae always absent; appressed propodeal setulae long and separated by at least own length; propodeal spiracle situated at least twice its width from the declivitous face of propodeum, and shorter (length < 0.50 × height of propodeum). **Petiole.** In profile, petiolar node subcuboidal, vertex bluntly rounded; in full-face view, shape of petiolar node uniformly rounded; node shining and smooth with vestigial sculpture. **Gaster.** Gaster weakly shining with indistinct shagreenation; pilosity of first gastral tergite consisting of well-spaced short, inconspicuous, appressed setae only, erect setae always absent. **General characters.** Colour light brown to blackish, usually concolorous, but gaster may be darker.

#### Major worker description.

**Head.** Head square; posterior margin of head planar or weakly concave; cuticle of frons shining with superficial shagreenation or microreticulation only; frons consisting exclusively or almost exclusively of well-spaced, appressed setae only (small, erect setae, if present, usually confined to ocular triangle or posterior margin of head). Eye moderate (eye length 0.20–0.49 length of head capsule); in full-face view, eyes set at midpoint of head capsule; in profile, eye set anteriad of midline of head capsule; eyes elliptical. In full-face view, frontal carinae concave; frontal lobes straight in front of antennal insertion. Anterior clypeal margin broadly and evenly convex; clypeal psammophore set at or just above anterior clypeal margin; palp formula 6,4. Five to six mandibular teeth in major worker; mandibles triangular, weakly incurved; third mandibular tooth distinctly shorter than apical tooth and teeth numbers two and four; masticatory margin of mandibles approximately aligned vertically or weakly oblique. **Mesosoma.** Integument of pronotum, mesonotum and mesopleuron moderately shining and shagreenate throughout; anterior mesosoma in profile convex anteriad, mesonotum overlapping pronotum, planar or slightly sinuate posteriad; erect pronotal setae absent; in profile, metanotal groove shallow, indicated mainly by an angle and metathoracic spiracles; propodeum shining and shagreenate; propodeum smoothly rounded or with indistinct angle; propodeal dorsum and declivity confluent; erect propodeal setae absent; appressed propodeal setae short, separated by more than own length and inconspicuous; propodeal spiracle situated on or beside declivitous face of propodeum, and shorter (length less than 0.50 × height of propodeum). **Petiole.** In profile, petiolar node squamiform; in full-face view, shape of petiolar node generally rounded with median indentation; node shining and faintly shagreenate-microreticulate. **Gaster.** Gaster weakly shining with indistinct shagreenation; pilosity of first gastral tergite consisting of well-spaced short, inconspicuous, appressed setae only, erect setae always absent. **General characters.** Colour of foreparts orange or brownish-orange with dark brown gaster.

#### Measurements.

Worker (n = 8): CI 96–123; EI 17–30; EL 0.25–0.40; HL 0.86–1.94; HW 0.83–2.38; ML 1.65–2.77; MTL 1.25–1.83; PpH 0.16–0.33; PpL 0.72–1.18; SI 77–94; SL 0.78–1.83.

#### Comments.

This common, gracile species is intermediate in appearance between the *M. bagoti* complex and the *M. aeneovirens* complex. Limited sequencing data suggests the ant forms a monotypic complex basal to *M. bagoti* and *M. gracilipes*. Morphologically, this species is readily distinguished from others in the *M. aeneovirens* group by the high placement of the clypeal ammochaetae on the clypeus. *Melophorus nemophilus* is an arid or semi-arid resident of the southern states, and has been recorded from SA, Victoria and WA. The species may also occur in southwestern NSW, but is most probably absent from QLD and Tasmania. Most samples have been collected from remote areas in dunes, mallee woodland and spinifex and mallee woodland. The species most probably derives much of its nutriment from carrion, honeydew and nectar. A photograph by Ajay Narendra shows two minor workers carrying the corpse of a membracid bug while a major worker looks on (Fig. 6). Interestingly, this very gracile species can climb and forages on trees: the senior author of this paper has seen workers of *M. nemophilus* scurrying along smooth-trunked eucalypts near Norseman, WA, and workers have been taken in malaise traps in Little Desert N P, Murray Sunset N P and Wyperfeld N P in mid-western and northern Victoria.

#### Etymology.

Latin *nemus* (‘forest’) plus Neo-Latin *philus* (‘attracted to’); noun in the nominative singular standing in apposition to the generic name.

**Figure 25.**
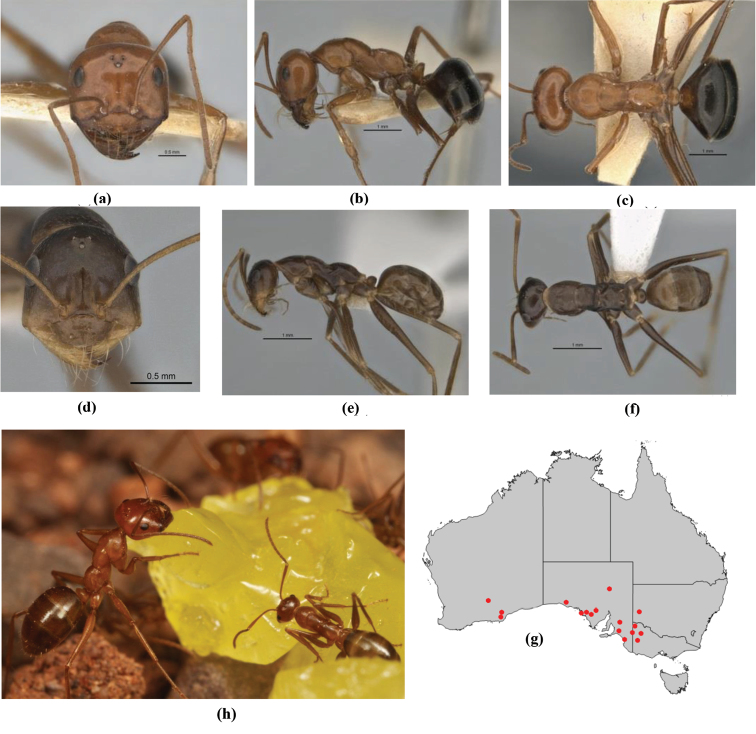
*Melophorus nemophilus* sp. n.: media worker holotype (ANIC32-900077–top ant) frons (**a**), profile (**b**) and dorsum (**c**); non–type minor worker (ANIC32-030935) frons (**d**), profile (**e**) and dorsum (**f**); *Melophorus nemophilus* minor and major worker at bait (photo credit: A. J. Narendra) (**g**); distribution map for the species (**h**). Low resolution scale bars: 1 mm (**b, c**); 0.5 mm (**e, f**).

### *Melophorus anderseni* species-group

This small group of species is characterized by at least two distinctive apomorphies; namely, short, six-segmented maxillary palps with a strongly acuminate final segment and absent metatibial spur. At least one member is a stealth raider of northern meat ant (*Iridomyrmex sanguineus*) colonies. No specimens of this species-group were available for sequencing.

### Melophorus anderseni

Taxon classificationAnimaliaHymenopteraFormicidae

Agosti

Melophorus anderseni Agosti 1997: 163, figs 4, 5 (w.q.)

#### Types.

Holotype minor worker, paratype minor workers male and queen, TERC site, Darwin, Northern Territory [ANIC] [CASC] (examined: ANIC holotype (middle ant) CASENT0172008, paratype minor worker CASENT0172007 on the same pin, one worker of *Iridomyrmex sanguineus* on the same pin as holotype and paratype with a label indicating association of the two species, ANIC paratype queen and two minor workers CASENT0172009, AntWeb image of paratype male [CASC] CASENT0173921.

#### Other material examined.

**Northern Territory**: Darwin (Shattuck, S.O. [ANIC32-066666]), Mudginberri (Andersen, A.N. [ANIC32-066653]).

#### Diagnosis.

*Melophorus anderseni* is a member of the *M. anderseni* species-group (maxillary palp segments short [not reaching neck sclerite], narrow and terminating in a subulate [awl-shaped] segment; PF 6,4; metatibial apical spur absent; in full-face view, masticatory margin of mandible strongly oblique with four teeth in known major workers [except *chrysus*], and four to six teeth in minor worker). This species is distinguished from three other members of the group by having a thick, rectangular or quadrate petiolar node in the minor worker, the anterior clypeal margin broadly convex and protrusive; the clypeal psammophore located below the midline of the clypeus (major worker) or near its anterior margin (minor worker) and in having the antennal scape of minor worker devoid of erect setae. The species is confined to northern Australia.

#### Minor worker description.

**Head.** Head approximately oval with straight sides; posterior margin of head planar or weakly convex; frons matt or with weak sheen, shagreenate; frons consisting almost completely of appressed setae that may form pubescence (tiny, erect setae, if present, usually confined to ocular triangle). Eye moderate (eye length 0.20–0.49 length of side of head capsule); in full-face view, eyes set above midpoint of head capsule; in profile, eye set anteriad of midline of head capsule; eyes elliptical or slightly reniform. In full-face view, frontal carinae straight or weakly convex; frontal lobes straight in front of antennal insertion. Anteromedial clypeal margin broadly and evenly convex and protrusive; clypeal psammophore set at or just above anterior clypeal margin; palp formula 6,4. Four to five mandibular teeth in minor worker; mandibles narrow, strap-like, internal and external margins parallel or nearly so; third mandibular tooth distinctly shorter than apical tooth and teeth numbers two and four; masticatory margin of mandibles strongly oblique. **Mesosoma.** Integument of pronotum, mesonotum and mesopleuron moderately shining and shagreenate throughout; anterior mesosoma in profile broadly convex; erect pronotal setae absent; in profile, metanotal groove a weak or vestigial furrow; propodeum shining and shagreenate; propodeum angulate, propodeal angle blunt; length ratio of propodeal dorsum to its declivity about 1:1; erect propodeal setae present and abundant (greater than 12); appressed propodeal setulae long and closely aligned, creating pubescence; propodeal spiracle situated nearer to midpoint of propodeum than to its declivitous face, and shorter (length < than 0.50 × height of propodeum). **Petiole.** In profile, petiolar node cuboidal, rounded above; in full-face view, shape of petiolar node uniformly rounded; node shining and distinctly shagreenate-microreticulate. **Gaster.** Gaster shining with superficial microreticulation; pilosity of first gastral tergite consisting of thick, appressed setae that form pubescence, interspersed with numerous short, bristly, erect setae. **General characters.** Colour reddish-brown, gaster slightly darker.

#### Major worker description.

**Head.** Head square; posterior margin of head planar or weakly convex; cuticle of frons shining and smooth except for piliferous pits; pilosity of frons a mixture of well-spaced, distinctly longer erect and semi-erect setae interspersed with shorter decumbent setae. Eye moderate (eye length 0.20–0.49 length of head capsule); in full-face view, midpoint of head capsule; in profile, eye set anteriad of midline of head capsule; eyes elliptical. In full-face view, frontal carinae distinctly concave, frontal lobes curved toward antennal insertion. Anterior clypeal margin broadly and evenly convex; clypeal psammophore set at or above midpoint of clypeus; palp formula 6,4. Mandibular teeth in major worker always four; mandibles narrow, strap-like, internal and external borders parallel or nearly so; third mandibular tooth distinctly shorter than apical tooth and teeth numbers two and four; masticatory margin of mandibles strongly oblique. **Mesosoma.** Integument of pronotum, mesonotum and mesopleuron moderately shining and shagreenate throughout; anterior mesosoma in profile broadly convex; erect pronotal setae short, (i.e., shorter than length of eye) and unmodified; in profile, metanotal groove shallow, indicated mainly by an angle and metathoracic spiracles; propodeum shining and shagreenate; propodeum angulate, propodeal angle blunt; length ratio of propodeal dorsum to its declivity between 1:1 and1:2; erect propodeal setae present and abundant (at least a dozen); appressed propodeal setae long and closely aligned, creating pubescence; propodeal spiracle situated nearer to midpoint of propodeum than to its declivitous face, and shorter (length less than 0.50 × height of propodeum). **Petiole.** In profile, petiolar node narrowly conical, vertex sharply defined; in full-face view, shape of petiolar node generally rounded with median indentation; node shining and faintly shagreenate-microreticulate. **Gaster.** Gaster weakly shining with indistinct shagreenation; pilosity of first gastral tergite consisting of thick, appressed setae that form pubescence, interspersed with numerous short, bristly, erect setae. **General characters.** Colour dull orange, gaster brownish-orange.

#### Measurements.

Worker (n = 2): CI 98–114; EI 19–24; EL 0.25–0.32; HL 1.06–1.45; HW 1.04–1.65; ML 1.76–1.94; MTL 1.28–1.40; PpH 0.16–0.19; PpL 0.69–0.78; SI 73–135; SL 1.20–1.39.

#### Comments.

Only a few collections of this social parasite of *Iridomyrmex sanguineus* have been taken, and the description of the worker by Agosti (1997) is in informal, telegraphic style, and is characterized more formally here for the first time. The behavior of *M. anderseni* in raiding nests of *I. sanguineus* has been well documented by the same author (Agosti 1997). TERC material has been taken in Kakadu, Kidman Springs, the Ranger Uranium Mine lease (NT), Mt Isa and Tin Camp Creek (QLD). All ANIC collections have been taken in the NT, but the ant may also occur in the Kimberley region of WA (at least) where populations of its host also occur. The apparent rarity of the species may be due to under collection because of its very specialized habits. *Melophorus anderseni* has several apomorphic characters shared only by the following species (notably very short palps and an absent metatibial apical spur), but its position within the genus as a whole is uncertain in the absence of genetic sequencing. However, the morphology suggests it is not close to the *M. aeneovirens* group. A possible close relationship with the *M. fieldi* group and, in particular, the *M. fieldi* complex is indicated by the presence of an anteromedian dorsal lip or protuberance in the queen (though not the workers).

**Figure 26.**
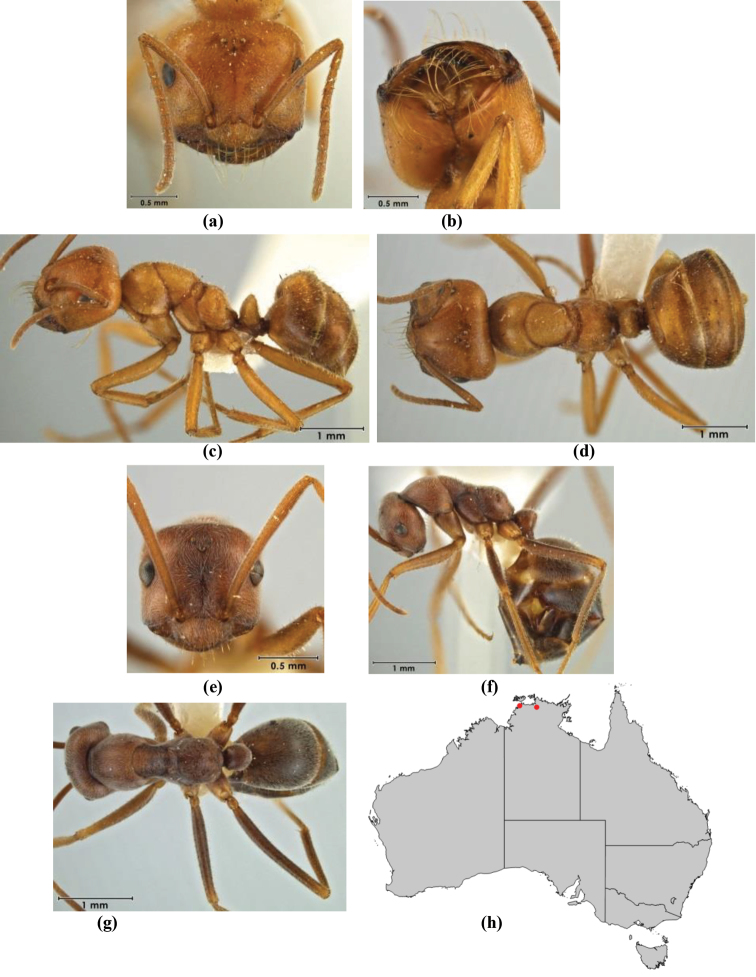
*Melophorus anderseni* Agosti: non–type major worker (ANIC32-066653) frons (**a**), underside of head showing palps (**b**), profile (**c**) and dorsum (**d**); non–type minor worker (ANIC32-066666) frons (**e**), profile (**f**) and dorsum (**g**); distribution map for the species (**h**).

### Melophorus andersenioides

Taxon classificationAnimaliaHymenopteraFormicidae

Heterick, Castalanelli & Shattuck sp. n.

http://zoobank.org/DB37A088-0EB6-482E-ADB1-1C3EA6CB5E61

#### Types.

Holotype minor worker (bottom ant) from 40 miles N of Warren, New South Wales, 23 December 1963, B.B. Lowery, red soil, ANIC ANTS VIAL 68.156 [ANIC32-900082] (ANIC). Paratypes: Major worker and minor worker on same pin as holotype (ANIC); 2 media and a minor worker from Grantham, Queensland, 24 January 1957, B.B. Lowery, dry sclerophyll, ANIC ANTS VIAL 68.209 (BMNH); 3 major workers, media worker and minor worker from Warwick, Queensland, 4 January 1966, B.B. Lowery, Red soil, hilly, sav. woodland, edge of *I. detectus* nest, morning, R28 (MCZ); minor worker and major worker from Grantham, Queensland, 24 January 1957, B.B. Lowery [ANIC32-900081] (QM); major worker from ROC lease, Eneabba, Western Australia, 10-16 April 1997, L. Bisevac/B.E.Heterick, Plot 77AS, pitfall traps [JDM32-002001] (WAM); 2 minor workers and major worker from Nerren Nerren Stn 27°00’s, 114°32’e, Western Australia, 26 September-19 October 1994, M.S. Harvey/J.M. Waldock, NE4 wet pits, WAQM/CALM survey, Carnarvon Basin [JDM32-002002] (WAM).

#### Diagnosis.

*Melophorus andersenioides* is a member of the *M. anderseni* species-group (maxillary palp segments short [not reaching neck sclerite], narrow and terminating in a subulate [awl-shaped] segment; PF 6,4; metatibial apical spur absent; in full-face view, masticatory margin of mandible strongly oblique with four teeth in known major workers [except *chrysus*], and four to six teeth in minor worker). This species is distinguished from three other members of the group by having a thick, rectangular or quadrate petiolar node in the minor worker, the anterior margin of clypeus weakly convex, the clypeus folded back and not protrusive, the clypeal psammophore located at the midline of clypeus and in having the antennal scape of minor worker with many short, erect, bristly setae.

#### Minor worker description.

**Head.** Head square; posterior margin of head planar or weakly convex; frons shining with indistinct microsculpture that is most pronounced on lower surfaces; frons consisting of appressed pubescence, with many short, unmodified, erect setae. Eye moderate (eye length 0.20–0.49 length of side of head capsule); in full-face view, eyes set above midpoint of head capsule; in profile, eye set anteriad of midline of head capsule; eyes elliptical or slightly reniform. In full-face view, frontal carinae concave; frontal lobes straight in front of antennal insertion. Anteromedial clypeal margin broadly convex with anteromedial dimple; clypeal psammophore set at or above midpoint of clypeus; palp formula 6,4. Mandibular teeth in minor worker four to five; mandibles narrow, strap-like, internal and external margins parallel or nearly so; third mandibular tooth distinctly shorter than apical tooth and teeth numbers two and four, or distinctly shorter than apical tooth and tooth no. two, tooth no. four vestigial, or distinctly shorter than apical tooth, but equivalent in length to remaining teeth, or absent; masticatory margin of mandibles strongly oblique. **Mesosoma.** Integument of pronotum, mesonotum and mesopleuron moderately shining and shagreenate throughout; anterior mesosoma in profile broadly convex; erect pronotal setae absent; in profile, metanotal groove a weak or vestigial furrow; propodeum shining and shagreenate; propodeum angulate, propodeal angle blunt; length ratio of propodeal dorsum to its declivity about 1:1; erect propodeal setae present and abundant (greater than 12); appressed propodeal setulae long and closely aligned, creating pubescence; propodeal spiracle situated nearer to midpoint of propodeum than to its declivitous face, and shorter (length < than 0.50 × height of propodeum). **Petiole.** In profile, petiolar node cuboidal, rounded above; in full-face view, shape of petiolar node uniformly rounded; node shining and distinctly shagreenate-microreticulate. **Gaster.** Gaster shining with superficial microreticulation; pilosity of first gastral tergite consisting of thick, appressed setae that form pubescence, interspersed with numerous short, bristly, erect setae. **General characters.** Colour brownish-orange to dark russet.

#### Major worker description.

**Head.** Head horizontally rectangular, broader than wide; posterior margin of head planar or weakly concave; cuticle of frons shining and smooth except for piliferous pits; frons consisting of appressed pubescence, with many short, unmodified, erect setae. Eye moderate (eye length 0.20–0.49 length of head capsule); in full-face view, midpoint of head capsule; in profile, eye set anteriad of midline of head capsule; eyes elliptical. In full-face view, frontal carinae distinctly concave; frontal lobes curved toward antennal insertion. Anterior clypeal margin broadly convex with anteromedial dimple; clypeal psammophore set at or above midpoint of clypeus; palp formula 6,4. Four to five mandibular teeth in major worker; mandibles narrow, strap-like, internal and external borders parallel or nearly so; third mandibular tooth distinctly shorter than apical tooth and teeth numbers two and four; masticatory margin of mandibles strongly oblique. **Mesosoma.** Integument of pronotum, mesonotum and mesopleuron shining with indistinct microsculpture that is most pronounced on lower surfaces; anterior mesosoma in profile steeply rounded anteriad, thereafter pronotum and whole of mesonotum flattened and on a higher plane than propodeum; erect pronotal setae short, (i.e., shorter than length of eye) and unmodified; in profile, metanotal groove shallow, indicated mainly by an angle and metathoracic spiracles; propodeum shining and generally smooth, with only weak indistinct shagreenation; propodeum angulate, propodeal angle blunt; length ratio of propodeal dorsum to its declivity between 1:1 and 1:2; erect propodeal setae present and abundant (at least a dozen); appressed propodeal setae long and closely aligned, creating pubescence; propodeal spiracle situated on or beside declivitous face of propodeum, and shorter (length less than 0.50 × height of propodeum). **Petiole.** In profile, petiolar node squamiform; in full-face view, shape of petiolar node tapered with blunt vertex; node shining and smooth with vestigial microreticulation anteriad. **Gaster.** Gaster weakly shining with indistinct shagreenation; pilosity of first gastral tergite consisting of thick, appressed setae that form pubescence, interspersed with numerous short, bristly, erect setae. **General characters.** Colour brownish-orange.

#### Measurements.

Worker (n = 5): CI 104–127; EI 18–25; EL 0.25–0.31; HL 0.97–1.36; HW 1.00–1.74; ML 1.60–2.04; MTL 1.56–1.40; PpH 0.19–0.23; PpL 0.64–0.84; SI 72–136; SL 1.25–1.37.

#### Comments.

This ant has the species-group level characters of the previous taxon, but differs from it in the form of the clypeus (not protrusive in this species) and its greater hairiness. *Melophorus andersenioides* has a wider distribution than the foregoing (being found in NSW, QLD and WA), and has been collected in red soil. Label notes on two pins associate it with nests of *Iridomyrmex purpureus* (as ‘*I. detectus*’, its junior synonym), and it is altogether possible that *M. andersenioides* is a social parasite on that species analogously with its relation.

#### Etymology.

*anderseni* (see above) plus Greek -*oides* (‘resembling’).

**Figure 27.**
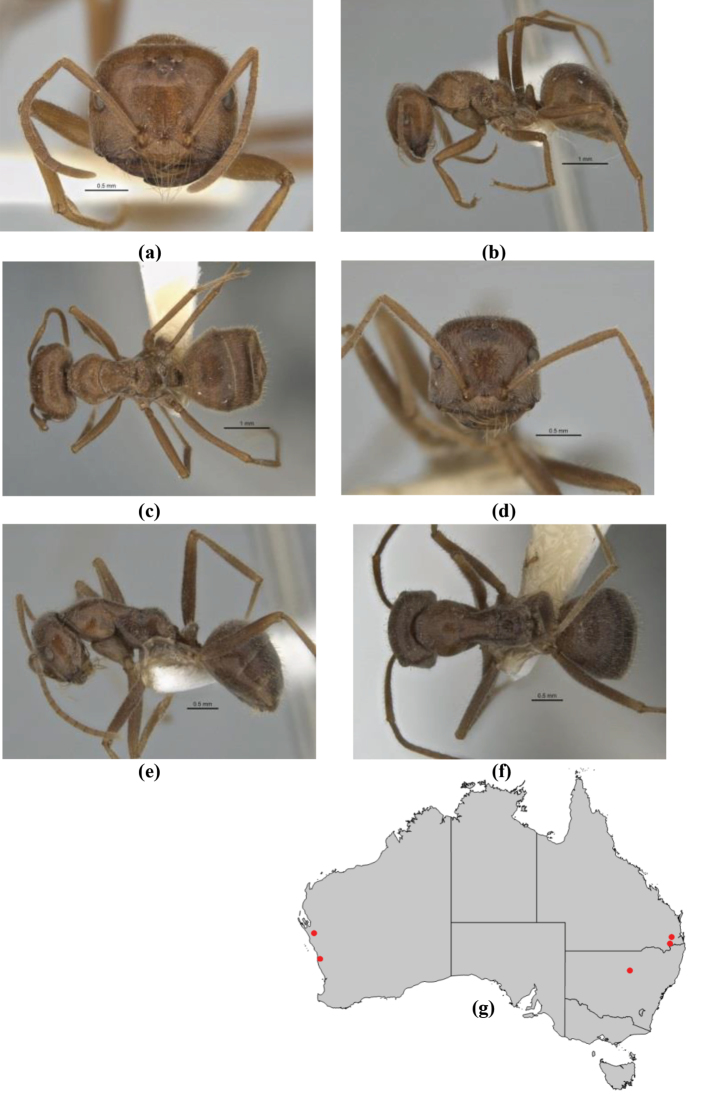
*Melophorus andersenioides* sp. n.: major worker paratype (ANIC32-900082) frons (**a**), profile (**b**) and dorsum (**c**); minor worker holotype (ANIC32-900082–bottom ant) frons (**d**), profile (**e**) and dorsum (**f**); distribution map for the species (**g**). Low resolution scale bars: 1 mm (**b, c**); 0.5 mm (**a, d–f**).

### Melophorus chrysus

Taxon classificationAnimaliaHymenopteraFormicidae

Heterick, Castalanelli & Shattuck sp. n.

http://zoobank.org/8173EC7D-79F1-4112-BD01-A2001006FFE5

#### Types.

Holotype minor worker from Wungong Catchment 32°16'40"S, 116°08'12"E, Western Australia, 6-13 February 2009, D. Kabay, Water Corporation thinning: thinning plot dieback tree upland jarrah forest, 2A, 3Y [JDM32-004655] (WAM). Paratypes: Major worker and 2 minor workers from Streaky Bay, South Australia, 3 October 1957, B.B Lowery, mallee scrub, [folded paper label] ‘*Melophorus*, Streaky Bay: SA 450 m[iles] W of Adelaide, mallee scrub, sandy nest in [*sic*] among galleries of *Melophorus laticeps* Wh. 3.10.57 B.B. Lowery’ [ANIC32-900127] (ANIC); minor worker from Cambrai, South Australia, 1-4 February 1972, P.J.M. Greenslade, dune Ib (SAM); 2 minor workers from Mount Bruce 22°37'32"S, 118°17'05"E, September 1991, S van Leeuwen, Fire/Mulga Research 1-2a [JDM32-004804] (WAM).

#### Other material examined.

**South Australia**: 16 mi N Gawler (Greaves, T.), Calca, near Streaky Bay (Lowery, B.B. [ANIC32-900146]). **Victoria**: 15 km WNW Yaapeet (Andersen, A.N.).

#### Diagnosis.

*Melophorus chrysis* is a member of the *M. anderseni* species-group (maxillary palp segments short [not reaching neck sclerite], narrow and terminating in a subulate [awl-shaped segment]; PF 6,4; metatibial apical spur absent; in full-face view, masticatory margin of mandible strongly oblique with four teeth in known major workers [except *chrysus*], and four to six teeth in minor worker). This species is distinguished from others in the group by having, in profile, the petiolar node thickly squamiform in the minor worker, the major worker smooth and glossy in appearance, and also by having, in profile, the pronotum smoothly rounded and inclined at angle > 30°, and, in profile, the clypeus evenly convex or more strongly convex posteriorly, but not bulbous. The “*pillipes*” condition (whorls of fine, erect setae on appendages) occurs in some populations.

#### Minor worker description.

**Head.** Head square; posterior margin of head weakly convex; frons shining with superficial shagreenation or microreticulation only; pilosity of frons a mixture of short, erect and semi-erect setae interspersed with shorter decumbent setae and well-spaced, short, appressed setae. Eye moderate (eye length 0.20–0.49 length of side of head capsule); in full-face view, eyes set at about midpoint of head capsule; in profile, eye set around midline of head capsule; eyes elliptical or slightly reniform. In full-face view, frontal carinae straight, divergent posteriad; frontal lobes curved toward antennal insertion. Anteromedial clypeal margin broadly and evenly convex; clypeal psammophore set at or above midpoint of clypeus; palp formula 6,4. Five mandibular teeth in minor worker; mandibles narrow, strap-like, internal and external margins parallel or nearly so; third mandibular tooth distinctly shorter than apical tooth and teeth numbers two and four; masticatory margin of mandibles strongly oblique. **Mesosoma.** Integument of pronotum, mesonotum and mesopleuron shining and mainly smooth, vestigial shagreenation most noticeable on humeri and mesopleuron; anterior mesosoma in profile broadly convex; appearance of erect pronotal setae short, (i.e., longest erect setae shorter than length of eye) and unmodified; in profile, metanotal groove shallow, indicated mainly by an angle; propodeum shining and smooth or with superficial and almost invisible microsculpture; propodeum smoothly rounded or with indistinct angle; propodeal dorsum and declivity confluent; erect propodeal setae variable in number, may be absent; appressed propodeal setulae sparse or absent, if present then not regularly spaced; propodeal spiracle situated on or beside declivitous face of propodeum, and longer (length ≥ 0.50 × height of propodeum). **Petiole.** In profile, petiolar node squamiform; in full-face view, shape of petiolar node uniformly rounded; node shining and smooth throughout. **Gaster.** Gaster smooth and glossy; pilosity of first gastral tergite consisting of well-spaced, erect and semi-erect setae interspersed with regularly placed appressed setae, or consisting wholly or mainly of long, curved setae, appressed setae apparently absent. **General characters.** Colour light yellow to gamboge yellow.

#### Major worker description.

**Head.** Head horizontally rectangular, broader than wide; posterior margin of head planar or weakly concave; cuticle of frons shining and smooth except for piliferous pits; pilosity of frons a mixture of a few well-spaced, erect setae interspersed with appressed setae only. Eye moderate (eye length 0.20–0.49 length of head capsule); in full-face view, midpoint of head capsule; in profile, eye set anteriad of midline of head capsule; eyes elliptical. In full-face view, frontal carinae distinctly concave; frontal lobes curved toward antennal insertion. Anterior clypeal margin broadly convex with anteromedial dimple; clypeal psammophore set at or above midpoint of clypeus; palp formula 6,4. Five mandibular teeth in major worker; mandibles narrow, strap-like, internal and external borders parallel or nearly so; third mandibular tooth distinctly shorter than apical tooth and teeth numbers two and four; masticatory margin of mandibles strongly oblique. **Mesosoma.** Integument of pronotum, mesonotum and mesopleuron shining and mainly smooth, vestigial shagreenation most noticeable on humeri and mesopleuron; anterior mesosoma in profile broadly convex; erect pronotal setae short, (i.e., shorter than length of eye) and unmodified; in profile, metanotal groove shallow, broadly V- or U-shaped; propodeum shining and generally smooth, with only weak indistinct shagreenation; propodeum always smoothly rounded; propodeal dorsum and declivity confluent; erect propodeal setae present and abundant (at least a dozen); appressed propodeal setae sparse or absent, if present then not regularly spaced; propodeal spiracle situated on or beside declivitous face of propodeum, and shorter (length less than 0.50 × height of propodeum). **Petiole.** In profile, petiolar node squamiform; in full-face view, shape of petiolar node tapered with blunt vertex; node shining and smooth with vestigial microreticulation anteriad. **Gaster.** Gaster weakly shining with indistinct shagreenation; pilosity of first gastral tergite consisting mainly of well-spaced short, semi-erect and decumbent setae. **General characters.** Colour gamboge yellow.

#### Measurements.

Worker (n = 6): CI 101–117; EI 22–32; EL 0.18–0.25; HL 0.56–0.96; HW 0.57–1.12; ML 0.81–1.32; MTL 0.56–0.80; PpH 0.09–0.11; PpL 0.36–0.58; SI 76–116; SL 0.66–0.85.

#### Comments.

This and the following species are rarely seen. This species is treated as a member of the *anderseni* group by Andersen (coming under ‘Group I’ in Andersen, 2007), and this is likely a correct placement. All four taxa possess short palps with an acuminate final segment, and the metatibial apical spur is absent. The close relationship of *Melophorus chrysus* to members of the *M. fieldi* complex is rendered more likely because it exhibits the ‘*pillipes*’ condition, otherwise seen only in ants belonging to that complex. The morphology is also similar. *Melophorus chrysus* is more common than the following taxon and has been collected in all Australian mainland states except NSW. Several ANIC specimens have been collected in mallee and this may be the usual habitat for the ant, but it has also been collected in marri forest just south of Perth, WA, and from a dune in SA. Most populations are glabrous, but several exhibit the very hairy ‘*pillipes*’ condition. No specimens have been sequenced and nothing is known of the ant’s habits apart from the information contained on the label mentioned for the ANIC paratypes.

#### Etymology.

*Chrysus* (Greek) was the spirit of gold; noun in the nominative singular standing in apposition to the generic name.

**Figure 28.**
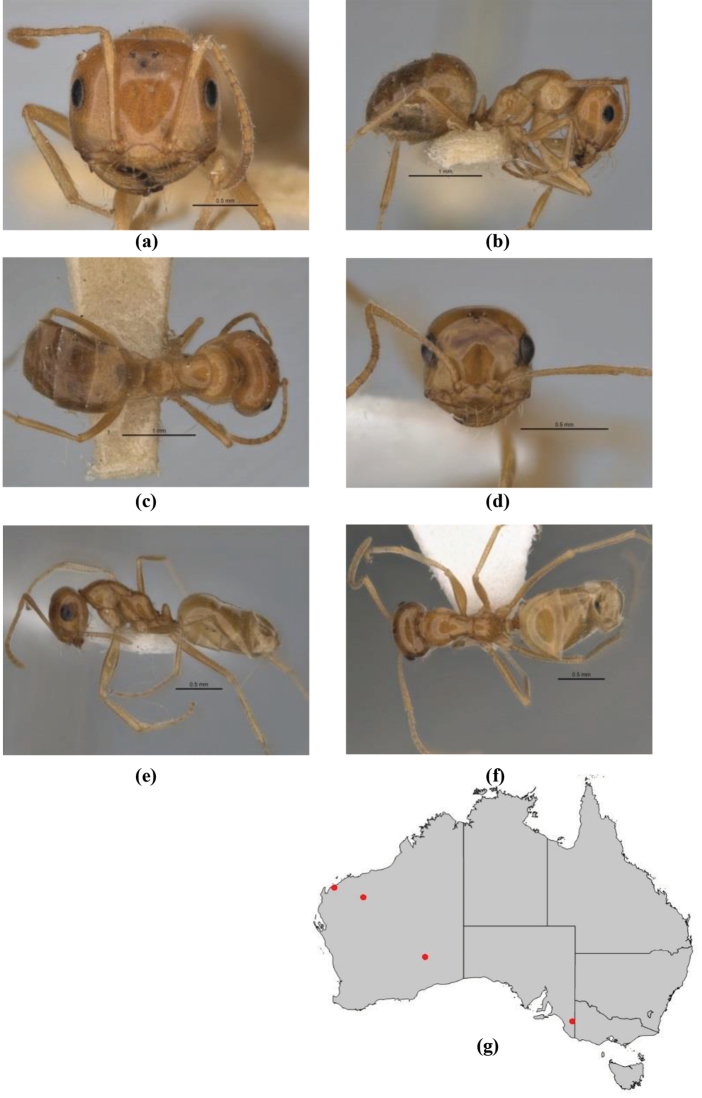
*Melophorus chrysus* sp. n.: non–type major worker (ANIC32-900146–middle ant)frons (**a**), profile (**b**) and dorsum (**c**); minor worker holotype (JDM32-004655) frons (**d**), profile (**e**) and dorsum (**f**); distribution map for the species (**g**). Low resolution scale bars: 1 mm (**b, c**); 0.5 mm (**a, d–f**).

### Melophorus subulipalpus

Taxon classificationAnimaliaHymenopteraFormicidae

Heterick, Castalanelli & Shattuck sp. n.

http://zoobank.org/8E4FCC70-F32A-4954-87FA-22A635B10DA9

#### Types.

Holotype minor worker from Tropicana Minesite 29°15'40"S, 124°35'50"E, Western Australia, January 2009, J. Summerhayes, pitfall tap, *Casuarina*, CA1:5 [JDM32-004701] (WAM). Paratypes: minor worker from Cambrai, South Australia, 24-28 February 1972, P.J.M. Greenslade, dune [ANIC32-900128] (ANIC); minor worker from 30 km ESE of Onslow 21°46'44"S, 115°22'01"E, Western Australia, 15 May 2006-29 August 2006, CALM Pilbara Survey, Site OYW12: ethylene glycol pitfalls [JDM32-004850] (WAM).

#### Other material examined.

**Western Australia**: Packsaddle (van Leeuwen, S.)

#### Diagnosis.

*Melophorus subulipalpus* is a member of the *M. anderseni* species-group (maxillary palp segments short [not reaching neck sclerite], narrow and terminating in a subulate [awl-shaped] segment; PF 6,4; metatibial apical spur absent; in full-face view, masticatory margin of mandible strongly oblique with four teeth in known major workers [except *chrysus*], and four to six teeth in minor worker). The major worker of this species is unknown. The minor worker of *M. subulipalpus* can be distinguished from its sister species (*M. chrysus*) by having, in profile, the pronotum more-or-less flattened and only very weakly inclined anteriad, the head and body weakly to moderately shining, the sculpture ranging from superficial microreticulation to evident shagreenation or minutely striate sculpture, and in having, in profile, the clypeus strongly convex, tending to bulbous. The “*pillipes*” condition has not been not seen in the few workers collected.

#### Minor worker description.

**Head.** Head square; posterior margin of head strongly convex; frons shining and uniformly striolate, or matt or with weak sheen, microreticulate or microreticulate-shagreenate; frons consisting exclusively or almost exclusively of well-spaced, appressed setae only (small, erect setae, if present, usually confined to ocular triangle or posterior margin of head). Eye moderate (eye length 0.20–0.49 length of side of head capsule); in full-face view, eyes set above midpoint of head capsule; in profile, eye set anteriad of midline of head capsule; eyes elliptical or slightly reniform. In full-face view, frontal carinae straight, divergent posteriad; frontal lobes straight in front of antennal insertion. Anteromedial clypeal margin straight, or broadly and evenly convex; clypeal psammophore set below midpoint of clypeus; palp formula 6,4. Five or six mandibular teeth in minor worker; mandibles narrow, strap-like, internal and external margins parallel or nearly so; in five-toothed workers third mandibular tooth distinctly shorter than apical tooth and tooth no. two, tooth no. four vestigial; masticatory margin of mandibles strongly oblique. **Mesosoma.** Integument of pronotum, mesonotum and mesopleuron shining, uniformly striolate or superficially microreticulate; anterior mesosoma in profile weakly elevated anteriad, thereafter gently sinuate, pronotum and mesonotum on same plane; erect pronotal setae absent; in profile, metanotal groove shallow, indicated mainly by an angle; propodeum shining and uniformly striolate, or matt or with a weak sheen and microreticulate; propodeum angulate, propodeal angle blunt; length ratio of propodeal dorsum to its declivity between1:1 and 1:2; erect propodeal setae always absent; appressed propodeal setulae long, each reaching setae behind and in front, but not forming pubescence; propodeal spiracle situated on or beside declivitous face of propodeum, and shorter (length < 0.50 × height of propodeum). **Petiolar.** In profile, petiolar node subcuboidal, vertex bluntly rounded; in full-face view, shape of petiolar node tapered with blunt vertex; node shining and distinctly shagreenate-microreticulate or superficially microreticulate. **Gaster.** Gaster weakly shining with indistinct shagreenation, or shining, shagreenate (‘LP record’ appearance); pilosity of first gastral tergite consisting of well-spaced short, inconspicuous, appressed setae only, erect setae always absent. **General characters.** Colour pale brownish-yellow to tan.

#### Measurements.

Worker (n = 4): CI 102–108; EI 26–30; EL 0.22–0.25; HL 0.70–0.93; HW 0.72–0.93; ML 1.15–1.53; MTL 0.97–1.29; PpH 0.12–0.15; PpL 0.49–0.63; SI 135–140; SL 1.01–1.26.

#### Comments.

Only four collections are known for this species, which can be distinguished from *M. chrysus* by its more matt appearance, less rounded pronotum and ochre colouration. The species occurs in WA and SA (at least). The sole South Australian specimen was collected from a dune, but otherwise nothing is known of the taxon.

#### Etymology.

Latin *subulus* (‘awl’) plus *palpus* (‘stroking’/‘caress’; applied to the palps of an arthropod); noun in the nominative singular standing in apposition to the generic name.

**Figure 29.**
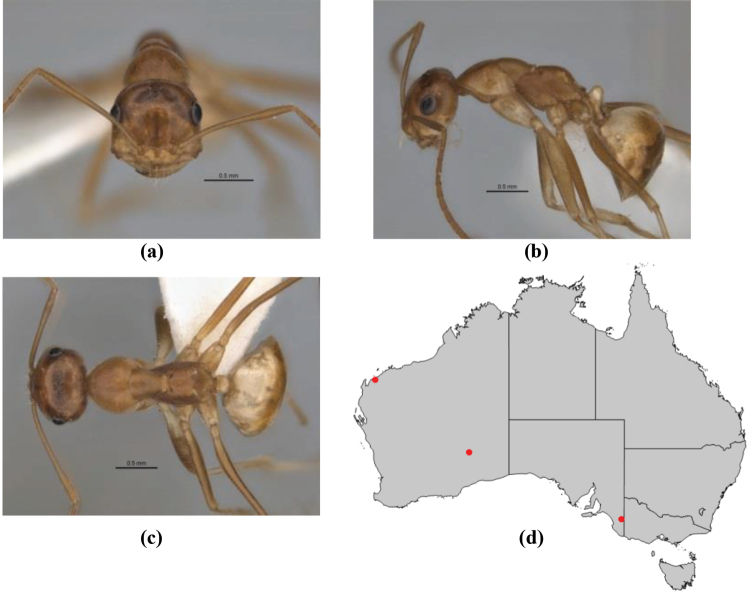
*Melophorus subulipalpus* sp. n.: minor worker holotype (JDM32-004701) frons (**a**), profile (**b**) and dorsum (**c**); distribution map for the species (**d**). Low resolution scale bars: 0.5 mm (**a–c**).

### *Melophorus biroi* species-group

This group of *Melophorus* is far and away the most diverse and species-rich in the genus. Many taxa are common but difficult to identify, even with expert analysis, because of the very similar appearance of anatomical structures and integument. The *M. biroi* species-group consists of five complexes (*biroi*, *brevignathus*, *fieldi*, *oblongiceps*, and *wheeleri*) representatives of all of which were successfully sequenced. The phylogenetic analysis of the complexes showed that each of the complexes were strongly supported, apart from *M. oblongiceps* which was missing the COI gene. As a consequence, the correct phylogenetic placement of *M. oblongiceps* is currently undetermined. The *M. fieldi* complex is the most derived within the group and this complex appears to be continuing to evolve rapidly, making the interpretation of molecular sequences just as difficult as interpretation of the morphology. Some continuing hybridization and lineage assortment is likely for *M. turneri* and several morphospecies that appear to be part of the *M. turneri* cluster. Most of the members of the broader species-group have a typical *Melophorus* ***habitus***, with large-headed, short-limbed major workers and media and minor workers with normally proportioned heads and long limbs. In all probability most species are generalist scavengers of plant (mainly) and animal material, but the *M. wheeleri* complex appears to be granivorous. Belying the reputation of the genus, some species appear not to be thermophiles as they have been collected while active in cooler times of the year.

### *Melophorus biroi* complex

Only seven out of eighteen members of this nominal complex of mostly small *Melophorus* have been genetically sequenced. A major surprise has been that several taxa initially placed in this complex (i.e., *ludius*, *pusillus* and *translucens*) due to their close morphological similarity were discovered to be only distantly related phylogenetically, and cannot be fitted easily even in the broader species-group. These have now been placed in a species-group of their own. Based on similarities of the propodeum, the flattened, plate-like node (in the major worker), and the depressed and slightly elongate head capsule, the following *Melophorus* species may prove to be in the clade that embraces *M. biroi*: *biroi*, *castanopus*, *cuneatus*, *dicyrtos*, *latinotus*, *longiceps*, *macrops*, *microreticulatus*, *propebiroi* and *gracilis*. This group, or, at least, the three members of the group that have been sequenced, appear to have a sister relationship to the *M. mjobergi* clade (which is decidedly hairier and has its own distinctive suite of apomorphies). *Melophorus minimus* appears to also belong to the *M. biroi* clade, but has not been sequenced. A second clade of *Melophorus* that shares a thick, porrect node in the minor caste, a rounded head and propodeum and a strongly microreticulate or distinctly scalloped mesopleuron includes the apparently rare *Melophorus argus* and *M. turbineus*.

Members of the *mjobergi* clade are small, compact *Melophorus*, which resemble *M. biroi* and are related to that taxon. Major workers of *Melophorus graciliceps* and *M. lissotriches* are not conspicuously different from *biroi* and its relatives apart from their hairiness, and are best separated from other members of the *biroi* complex on the basis of molecular data. Major and media workers of *M. compactus*, *M. mjobergi* and *M. postlei*, however, differ from those of *M. biroi* and its fellows by virtue of the deeply recessed area adjoining the lower frontal carinae and antennal insertions, the development of the torulus and, in *M. mjobergi* and *M. postlei*, by the appearance of the frontal carinae, which are vestigial. Major workers of these two species also have small pits around the head capsule. Minor workers of all members of the clade are not separated from those of the *biroi* clade by any one set of characters but are generally hairier and more obviously sculptured. This clade appears to have northern origins, and most collections have been taken north of the Tropic of Capricorn.

### Melophorus argus

Taxon classificationAnimaliaHymenopteraFormicidae

Heterick, Castalanelli & Shattuck sp. n.

http://zoobank.org/54E90C1F-B5F2-40A0-8316-F3547E69B75C

#### Types.

Holotype minor worker (top ant) from Kapalga, Alligator Rivers area, Northern Territory, 7-9 September 1983, P.J.M. Greenslade, 8 i traps, [ANIC32-066597] (ANIC). Paratypes: minor worker on same pin and with same details as holotype (ANIC); two minor workers from Kapalga, Alligator Rivers area, Northern Territory, 7-9 September 1983, P.J.M. Greenslade, 8 ii traps, (13), (MCZ).

#### Diagnosis.

*Melophorus argus* can be placed in the *M. biroi* species-group on the basis of characters of the clypeus, propodeum, mandible and palps. The species is also placed in the *M. biroi* species-complex on the basis of a further suite of characters (viz, metatibia of major worker with only one preapical spur [except rarely in the *mjobergi* clade]; clypeal psammophore placed anteriorly at or just above anterior margin of clypeus in the minor worker and often in the major worker; head dorsoventrally compressed to varying degrees in the minor worker of most species with the eyes placed high on the sides; compact legs, and small body size [[(excluding *mjobergi* clade) HW of smallest minor 0.36 mm, average HW of smallest minors 0.46 mm; HW of largest known major 1.29 mm, average HW of largest majors (where known) 1.05 mm]). The minor worker of *Melophorus argus* (the major worker is unknown) can be recognised by a combination of a promently scalloped mesopleuron, a thickish, dorsall rounded petiolar node and a glabrous mesosoma that is bimodal when viewed in profile.

#### Minor worker description.

**Head.** Head approximately oval with straight sides; posterior margin of head strongly convex; frons shining and smooth except for piliferous pits; frons consisting exclusively or almost exclusively of well-spaced, appressed setae only (small, erect setae, if present, usually confined to ocular triangle or posterior margin of head). Eye moderate (eye length 0.20–0.49 length of side of head capsule); in full-face view, eyes set at about midpoint of head capsule; in profile, eye set anteriad of midline of head capsule; eyes elliptical or slightly reniform. In full-face view, frontal carinae straight, divergent posteriad; frontal lobes curved toward antennal insertion. Anteromedial clypeal margin broadly and evenly convex, or narrowly protrusive anteromedially, the protrusion with a square border; clypeal psammophore set at or just above anterior clypeal margin; palp formula 6,4. Five mandibular teeth in minor worker; mandibles triangular, weakly incurved; third mandibular tooth distinctly shorter than apical tooth and teeth numbers two and four; masticatory margin of mandibles approximately vertical or weakly oblique. **Mesosoma.** Integument of pronotum, mesonotum and mesopleuron shining and smooth on dorsum, entire lower mesopleuron distinctly striolate-microreticulate; anterior mesosoma in profile pronotum smoothly rounded anteriad and flattened posteriad, mesonotum narrowly convex; erect pronotal setae absent; in profile, metanotal groove deep, ‘V’-shaped; propodeum shining, with multiple hair like striolae; propodeum angulate, propodeal angle blunt; length ratio of propodeal dorsum to its declivity about 1:1; erect propodeal setae always absent; appressed propodeal setulae short, separated by more than own length and inconspicuous; propodeal spiracle situated on or beside declivitous face of propodeum, and shorter (length < 0.50 × height of propodeum). **Petiole.** In profile, petiolar node broadly squamiform, almost a tubercle; in full-face view, shape of petiolar node uniformly rounded; node shining and smooth throughout. **Gaster.** Gaster smooth and glossy; pilosity of first gastral tergite consisting of well-spaced short, inconspicuous, appressed setae only, erect setae always absent. **General characters.** Colour concolorous brown.

#### Measurements.

Worker (n = 2): CI 87–90; EI 29–32; EL 0.12–0.11; HL 0.42–0.43; HW 0.36–0.39; ML 0.54–0.58; MTL 0.26–0.27; PpH 0.07–0.08; PpL 0.21–0.21; SI 113–116; SL0.37–0.44.

#### Comments.

This small, northern species is definitely known from the minor worker only, but the principal author of this paper has noted that a possible TERC major worker is similar to that of *M. biroi*. Only two pins, one of two workers from Kapalga, NT and one without any label data, were available for analysis. However, additional material for this species from the NT (Kakadu NP; Howard Springs; Wildman Riv.; Berry Springs; Brydle Hill) and also the Kimberley, WA (4 km W King Cascade) was briefly noted in the TERC Collection. *Melophorus argus* is recognizable by its completely glabrous appearance and its bimodal mesosomal profile. The Kakadu specimens were pitfall trapped by Greenslade, but nothing more is known of this ant.

#### Etymology.

Latinized Greek *argus* (Greek *argos* ‘shining’); participle in the nominative singular.

**Figure 30.**
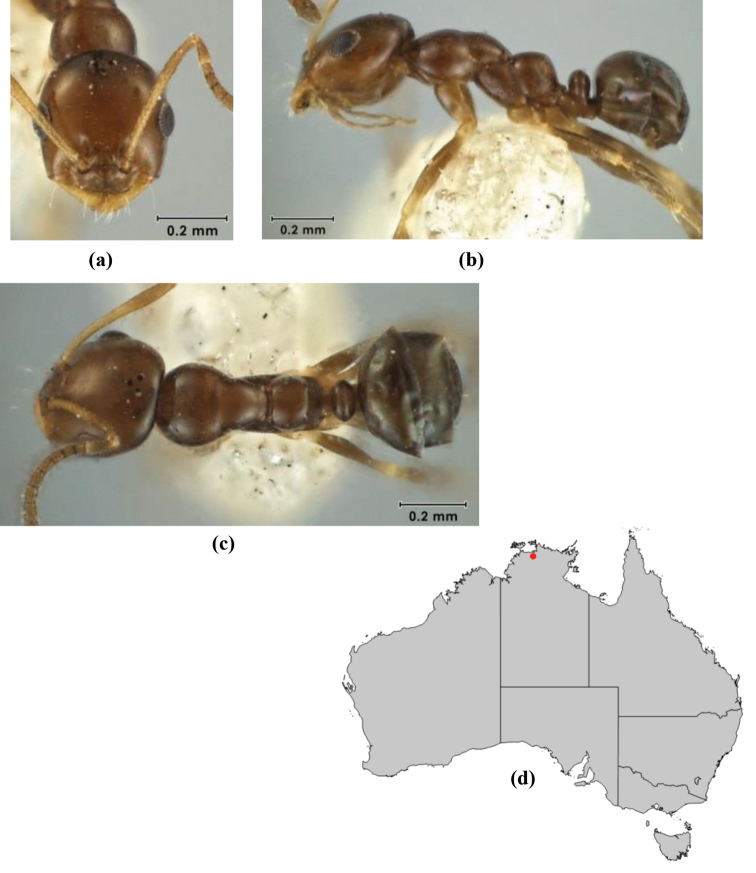
*Melophorus argus* sp. n.: minor worker holotype (ANIC32-066597–top ant) frons (**a**), profile (**b**) and dorsum (**c**); distribution map for the species (**d**).

### Melophorus biroi

Taxon classificationAnimaliaHymenopteraFormicidae

Forel

Melophorus biroi Forel 1907: 29 (combination in M. (Erimelophorus) by Wheeler 1935: 71). Type. Melophorus biroi Forel. Holotype (probable) major worker, Mt Victoria, Blue Mountains, New South Wales [HNHM] (examined: images of HNHM specimen provided by Zoltán Vas).
Melophorus marius Forel 1910: 66 (combination in M. (Erimelophorus) by Wheeler 1935: 71). Type. Holotype major worker Tennants [*sic*] Creek, Northern Territory [MHNG] (examined: MHNG specimen No. 235). **Syn. n.**
Melophorus fieldi propinqua Viehmeyer 1925: 36. Type. Syntype major worker, Liverpool, New South Wales [ZHMB] (examined: Automontage images © B.Schurian/ MfN-Berlin.de, of ZHMB specimen 5C8576). **Syn. n.**

#### Other material examined.

**Australian Capital Territory**: Black Mt., Site 5 (Barnett, N.J. [ANIC32-029813]), Black Mt., Site 5 (Barnett, N.J. [ANIC32-029821]), Mt Ainslie (Lowery, B.B.), Yarralumla (Lowery, B.B.). **New South Wales**: 40 km NNW Louth, Lake Mere (Greenslade, P.J.M.), Armidale (Lowery, B.B.), Belanglo State Forest (Gush, T.), Bomaderry, Nowra (Lowery, B.B.), Cowan Creek (Lowery, B.B.), Fowlers Gap (Greenslade, P.J.M.), Kapunda, N Nyngan (Greenslade, P.J.M.), Lane Cove River, Burns Bay, Sydney (Lowery, B.B.), Mudgee (Lowery, B.B.), Myall Lakes (York, A.), Myall Lakes (Greenslade, P.J.M.), Myall Lakes (York, A.), Myall Lakes National Park (York, 0A.), Newholme Road, near Armidale (Sakurai, Y.), Pymble (Lowery, B.B.), Richmond (Greenslade, P.J.M.), St. Ives, Sydney (Lowery, B.B.). **Northern Territory**: 105 km N Yuendumu (Greenslade, P.J.M.), 25 km N Alice Springs (Shattuck, S.O.), about 3 km W Alice Springs (Feehan, J.E.), Tanami (Greenslade, P.J.M.), Tanami (Greenslade, P.J.M.), Tanami Desert (Greenslade, P.J.M.), vicinity of Ayers Rock (Allwood, A.). **Queensland**: ‘Gumbardo’ (Beutel, T.), ‘Merigol’ (Beutel, T.), 3.6 km NW homestead on Plum Pudding Track, Cravens Peak Station (Lemann, C. [ANIC32-036837]), 40 km E Cameron Corner (Greenslade, P.J.M.), 75 km E Cunnamulla (Greenslade, P.J.M.), Blair Athol Mine (Houston, W. [ANIC32-040331]), Proserpine, Thompson Creek (Raven & Burwell), Sandringham (Greenslade, P.J.M.), Sandringham (Greenslade, P.J.M.), St. George (Lowery, B.B.), Tindaree, Hannaford Rd South via Tara (House, A./Brown, S.), Wacol, Brisbane (Lowery, B.B.). **South Australia**: 10 km E Mt Ive Homestead (Greenslade, P.J.M.), 15 km NE Mt Bryan (Greenslade, P.J.M.), 50 km E Vokes Hill, Victoria Desert (Greenslade, P.J.M.), 53 km E Vokes Hill, Victoria Desert (Greenslade, P.J.M.), 5 km NW Ketchowla Homestead (Greenslade, P.J.M.), 5 km W Mt. Rough, Coorong-Keith (Greenslade, P.J.M.), 5 km WSW Pitlochry Homestead (Greenslade, P.J.M.), 6 km NW Mt Pleasant (Greenslade, P.J.M.), Belair (Greenslade, P.J.M.), Belair (Greenslade, P.J.M. [ANIC32-900109]), Belair (Greenslade, P.J.M.), Belair (Greenslade, P.J.M.), Belair (Greenslade, P.J.M.), Blyth (Lowery, B.B.), Brookfield Conservation Park (Shattuck, S.O.), Cambrai (Greenslade, P.J.M.), Cambrai (Greenslade, P.J.M.), Cambrai (Greenslade, P.J.M.), Cambrai (Greenslade, P.J.M.), Cambrai (Greenslade, P.J.M.), Chowilla (Greenslade, P.J.M.), Eyre Hwy, 9.7 km NE Cootra (Heterick, B.E. [M328]), Fairview Conservation Park, N Lucindale (Greenslade, P.J.M.), Gawler Ranges (Greenslade, P.J.M.), Glen Osmond (Greenslade, P.J.M.), Hale (Greenslade, P.J.M.), Katarapko Island (Loxton High [ANIC32-046389]), Koonamore (Greenslade, P.J.M.), Koonamore (Greenslade, P.J.M.), Koonamore, Milang Conservation Park (Greenslade, P.J.M.), Moorowie Plain (Greenslade, P.J.M.), Mt. Gunson (Case, T.J.), Mt. Remarkable, Flinders Ranges (Greenslade, P.J.M.), Napperby, Flinders Ranges (Greenslade, P.J.M.), North of Breakneck River, Kangaroo Island (Greenslade, P.J.M.), North Sandy Creek, Kangaroo Island (Greenslade, P.J.M.), Ocean Beach, Streaky Bay (Lowery, B.B.), Oraparinna, Flinders Ranges (Greenslade, P.J.M.), Para Wirra (Greenslade, P.J.M.), Rocky River, Kangaroo Island (Greenslade, P.J.M.), Salt Creek, Coorong (Greenslade, P.J.M.), Streaky Bay (Lowery, B.B.), vicinity of Nanam Well, Scorpion Springs Conservation Park (Museum Party), Victor Harbour (Greenslade, P.J.M.), Wudinna townsite (Heterick, B.E. [M331/M333]). **Tasmania**: Barnes Bay, N. Bruny Island (Lowery, B.B.), George Town (Littler, F.M.), Lefroy (Lowery, B.B.). **Victoria**: 15 km W Nyarrin (Greenslade, P.J.M.), 15 km WNW Yaapeet (Andersen, A.N.), 15 km WNW Yaapeet (Andersen, A.N.), Cape Schank (Lowery, B.B.), Cape Schank (Lowery, B.B.), Glenaladale National Park (Andersen, A.N.), Heathcote, near Bendigo (Lowery, B.B.), Hopetoun (Andersen, A.N.), Hurstbridge (Lowery, B.B.), Murray Sunset Natl. Park, Millewa South Bore Track, 5.2 km WSW Shearers Quarters (Lambkin, C., Yeates, D., Starwick, N. & Recsei, J. [ANIC32-043283]), Rotamah Island, Gippsland Lakes (Andersen, A.N.), Studley Park, Kew (Lowery, B.B.), Watsonia (Lowery, B.B.), Watsonia (Lowery, B.B.), Watsonia (Lowery, B.B.). **Western Australia**: 1 km W Canna (Heterick, B.E. [M317/M319]), 1.5 km S Koolyanobbing (Heterick, B.E. [M15]), 11 km W Terhan Water Hole (Heatwole, H.), 4 km W Ellendale Rd (Heterick, B.E. [M239/M240/M241/M242]), 4 km W Ellendale Rd (Heterick, B.E. [M243/M244/M245]), 46 mi WNW Norseman (Taylor, R.W.), 8 km NW Bluff Knoll, Stirling Ranges National Park (Ward, P.S.), Argyle Diamonds via Kununurra (Postle, A.T. [JDM32-001931]), Christmas Tree Well (Heterick, B.E. [M20]), Coomallo Downs (Heterick, B.E. [M12/M49]), County Downs Hsd (south boundary) (Heterick, B.E. [M248]), Eneabba-Leeman Rd. (Dunn, R. [JDM32-001943]), Ethel Creek (Varris, P.A. [JDM32-001940]), Eyre Hwy, 20 km N Norseman (Heterick, B.E. [M338]), Harrismith (Heterick, B.E. [JDM32-001927]), Hines Hill (Heterick, B.E. [JDM32-001929]), Hyden Cemetery (Heterick, B.E. [JDM32-001930]), Int. Holland Tr./Norseman Rd. (Heterick, B.E. [JDM32-001926]), Jurien (collector unknown [JDM32-001972]), Kojonup (Majer, J.D. [JDM32-001935]), Little Sandy Desert (Guthrie, N. A. [M144]), Mt Gibson rest area (Heterick, B.E. [M299/M300/M301]), Mulga, NE Goldfields (Pringle, H.J.R. [ANIC32-029570]), Ongerup-Jerramungup Rd. (Heterick, B.E. [JDM32-001925]), Sandstone Rd turnoff (B. .E Heterick [M296/M297/M298]), Sandstone Rd turnoff (Heterick, B.E. [M294]), Tammin (Clark, J.), The Granites’ Mt Magnet (Heterick, B.E. [M171]), Wannamal West (Heterick, B.E. [JDM32-001939]), Woolgangie (Heterick, B.E. [JDM32-001924]).

#### Diagnosis.

*Melophorus biroi* can be placed in the *M. biroi* species-group on the basis of characters of the clypeus, propodeum, mandible and palps. The species is placed in the *M. biroi* species-complex on the basis of a further suite of characters (viz, metatibia of major worker with only one preapical spur [except rarely in the *mjobergi* clade]; clypeal psammophore placed anteriorly at or just above anterior margin of clypeus in the minor worker and often in the major worker; head dorsoventrally compressed to varying degrees in the minor worker of most species with the eyes placed high on the sides; compact legs, and small body size [[(excluding *mjobergi* clade) HW of smallest minor 0.36 mm, average HW of smallest minors 0.46 mm; HW of largest known major 1.29 mm, average HW of largest majors (where known) 1.05 mm]). *Melophorus biroi* is very similar to several other small or very small *Melophorus*. The minor worker can be distinguished from similar forms by its almost invariably glabrous mesosoma, the lack of erect setae, especially marginal setae, on the gaster, the weakly impressed metanotal groove, the truncate propodeum, the thin, widened petiolar node and the lack of heavy sculpture on the mesopleuron. Since the major worker of many *M. biroi* complex species is not known, the *M. biroi* major worker cannot be diagnosed with high definition, and is best identified from nest series in which minor workers are also included. However, this subcaste has a weakly convex pronotum and mesonotum and a weakly ot moderately impressed metanotal groove enabling it to be separated from the very similar *M. castanopus* (flattened pronotum and mesonotum) and *M. dicyrtos* (strongly convex pronotum and mesonotum and deeply impressed metanotal groove), and the posterior clypeal margin is arched. The anterior clypeal margin often has a small dimple (as in many members of the *M. fieldi* complex). *Melophorus biroi* shows a lot of morphological variation, and there is substantial molecular evidence that this is a species complex of at least several species. Further investigation into *Melophorus biroi* is needed.

#### Minor worker description.

**Head.** Head square, or approximately oval with straight sides; posterior margin of head planar to strongly convex; frons shining with superficial shagreenation or microreticulation only; frons consisting exclusively or almost exclusively of well-spaced, appressed setae only (small, erect setae, if present, usually confined to ocular triangle or posterior margin of head). Eye moderate (eye length 0.20–0.49 length of side of head capsule); in profile, eye set at about midpoint of head capsule; in profile, eye set around midline of head capsule; eyes elliptical or slightly reniform. In full-face view, frontal carinae straight or weakly convex; frontal lobes straight in front of antennal insertion. Anteromedial clypeal margin broadly and evenly convex; clypeal psammophore set at or just above anterior clypeal margin; palp formula 6,4. Five mandibular teeth in minor worker; mandibles triangular, weakly incurved; third mandibular tooth distinctly shorter than apical tooth and teeth numbers two and four; masticatory margin of mandibles approximately vertical or weakly oblique. **Mesosoma.** Integument of pronotum, mesonotum and mesopleuron with weak to moderate sheen and superficial microreticulation (more pronounced on mesopleuron); anterior mesosoma in profile broadly convex, or smoothly rounded anteriad, thereafter pronotum and whole of mesonotum flattened and on same plane as propodeum, or weakly elevated anteriad, thereafter gently sinuate, pronotum and mesonotum on same plane; erect pronotal setae absent; in profile, metanotal groove shallow, broadly V or U-shaped, or a weak or vestigial furrow; propodeum shining and microreticulate; propodeum angulate, propodeal angle blunt; length ratio of propodeal dorsum to its declivity between 1:1 and 1:2; erect propodeal setae always absent; appressed propodeal setulae short, separated by more than own length and inconspicuous; propodeal spiracle situated on or beside declivitous face of propodeum, and longer (length ≥ 0.50 × height of propodeum). **Petiole.** In profile, petiolar node squamiform; in full-face view, shape of petiolar node uniformly rounded; node shining and smooth with vestigial sculpture. **Gaster.** Gaster shining with superficial microreticulation; pilosity of first gastral tergite consisting of well-spaced short, inconspicuous, appressed setae only, erect setae always absent. **General characters.** Colour variable, often orange-and-brown or concolorous brown; if foreparts light-coloured, gaster always brown to black.

#### Major worker description.

**Head.** Head square; posterior margin of head weakly concave; cuticle of frons shining and smooth except for piliferous pits and a few striolae around antennal insertions and frontal carinae; pilosity of frons a mixture of a few well-spaced, erect setae interspersed with appressed setae only. Eye small (eye length less than 0.2 × length of head capsule); in full-face view, eyes set above midpoint of head capsule; in profile, eye set anteriad of midline of head capsule; eyes elliptical. In full-face view, frontal carinae concave; frontal lobes straight in front of antennal insertion. Anterior clypeal margin broadly and evenly convex; clypeal psammophore set at or just above anterior clypeal margin; palp formula 6,4. Five mandibular teeth in major worker; mandibles triangular, weakly incurved; third mandibular tooth distinctly shorter than apical tooth and teeth numbers two and 4; masticatory margin of mandibles approximately aligned vertically or weakly oblique. **Mesosoma.** Integument of pronotum, mesonotum and mesopleuron with weak to moderate sheen, shagreenate on pronotum and dorsum of mesonotum, otherwise microreticulate; anterior mesosoma in profile broadly convex, or pronotum smoothly rounded anteriad and flattened posteriad, mesonotum narrowly convex; erect pronotal setae short and unmodified, or weakly expanded distally; in profile, metanotal groove shallow, broadly V- or U-shaped; propodeum shining and finely striolate and microreticulate; propodeum smoothly rounded or with indistinct angle, or angulate, propodeal angle blunt; length ratio of propodeal dorsum to its declivity greater than 1:2; erect propodeal setae absent; appressed propodeal setae sparse or absent, if present then not regularly spaced; propodeal spiracle situated on or beside declivitous face of propodeum, and shorter (length less than 0.50 × height of propodeum). **Petiole.** In profile, petiolar node squamiform; in full-face view, shape of petiolar node uniformly rounded, or square with rounded angles; node shining and smooth with vestigial microreticulation anteriad. **Gaster.** Gaster shining, shagreenate (‘LP record’ appearance); pilosity of first gastral tergite consisting of short, bristly, erect setae over well-spaced, short, appressed setae. **General characters.** Colour mostly orange with brown gaster, head may be brown with yellow pronotum and mesonotum and brown propodeum and metasoma, or head and gaster may be brown with tan mesosoma.

#### Measurements.

Worker (n = 8): CI 92–113; EI 22–37; EL 0.18–0.24; HL 0.53–0.97; HW 0.49–1.10; ML 0.69–1.24; MTL 0.36–0.66; PpH 0.09–0.12; PpL 0.29–0.51; SI 69–105; SL 0.51–0.76.

#### Comments.

*Melophorus biroi* is the most abundant and widespread of all the small *Melophorus* and is found in all mainland Australian states and in Tasmania. The glabrous mesosoma in the minor worker, lack of erect non-marginal setae on the first gastral tergite and the straight appearance of the mesosoma in profile differentiate this ant from others in its clade. The taxon exhibits a variety of colours and morphologies: most southwestern populations have tawny orange foreparts and a dark gaster, while populations in the northern part of the Swan Coastal plain are uniformly large and dark. *Melophorus biroi* specimens in northern Australian and in arid parts may be rather rugose and brownish in colour, often with darker heads and gasters, while *M. biroi* from the eastern NSW tend to be dark coloured, quite gracile, and with longer heads (although not as long or compressed as seen in *M. longiceps*).

Material for sequencing came from only a portion of the phenotypes seen for this taxon, and this requires caution in interpreting the genetic output. Thus, there may be hidden species among the morphological variations that have not been sequenced. Regarding the sequenced material, there was found to be a rather odd dichotomy between three geographically closely associated collections made in the Kimberley and the remainder of the material for the genes COI, H3 and AA, but all successfully sequenced samples clustered together for genes LR and Wg. Given the widespread nature of the ant and its varied colouration and morphology, the dispersion evident in several of the genes used is unsurprising. However, samples M240 and M241 (which cluster with the quite unrelated *M. xouthos* for gene AA and COI, and, along with sample M248, at the base of all *Melophorus* for gene H3) produced an anomalous result, making the taxon polyphyletic when five- and three-gene trees were generated (since the *M. biroi* samples in the three-gene tree are not monophyletic, they have been assigned alpha suffixes to denote separate morphospecies status). The pinned voucher for sample M240 reveals a planar mesosoma with only a hint of a metanotal groove when seen in profile, and this may be suggestive of a distinct genotype. However, the profile of the sequenced sample M248 and its accompanying pinned voucher are unremarkable and typical of *M. biroi*. Unfortunately, M241 is a singleton and the M240 sequenced sample is represented only by the gaster. All of these workers were collected within a metre or so of each other and at the same time, and were assumed to be nestmates.

*Melophorus marius* Forel represents the lighter coloured version of this taxon (*M. biroi* is dark), but the habitus is the same and the later name *marius* becomes provisionally a junior synonym in this monograph. Also relegated to the position of a junior synonym here is *M. fieldi propinqua*. The syntypes seen for this taxon, both major workers, are superficially similar to *M. fieldi*, but the low placement of the clypeal psammophore, the appearance of the tibiae and tibial spurs and the narrow propodeum reveal its correct identity. Unfortunately, Viehmeyer did not describe minor workers, which look very different to the minor workers of *M. fieldi*. Viehmeyer’s ants constitute a sample of the large-eyed form of *M. biroi*, seen in pockets throughout Australia.

At present, the species diagnosis is the best that can be managed, but there are undeniably questions regarding the monophyly of *M. biroi*, and the possibility that, on further investigation, *M. biroi*, *M. fieldi propinqua* and *M. marius* may prove to constitute more than one species. In fact, the wide genetic divergence makes this putative taxon polyphyletic on a three-gene tree (see Figure 3), and more material – especially from the Kimberley region and from the eastern states – is needed to untangle the currently confusing picture, in which phenetics and genetics are in conflict.

Although the collection data follows the same pattern as for other *Melophorus* in being scant in nature, the sheer volume of specimens collected has resulted in what is likely to be a comprehensive list of habitats: these include *Eucalyptus* woodland, inland dunes, ‘dry sclerophyll’, ‘coastal scrub’, sandstone scrub, mallee, mallee heath, burned mallee, grazed eucalyptus dieback area, box-pine scrub, twig litter, scattered shrubs and spinifex over red sand, relict Kwongan bushland, mulga bushland, remnant brigalow, closed forest, semi-arid red clay soil, white sand over leaves, and paddock. This species also occurs in suburban areas, particularly those with some remnant vegetation. The ant is a ground forager and almost certainly a generalist in its habits.

**Figure 31.**
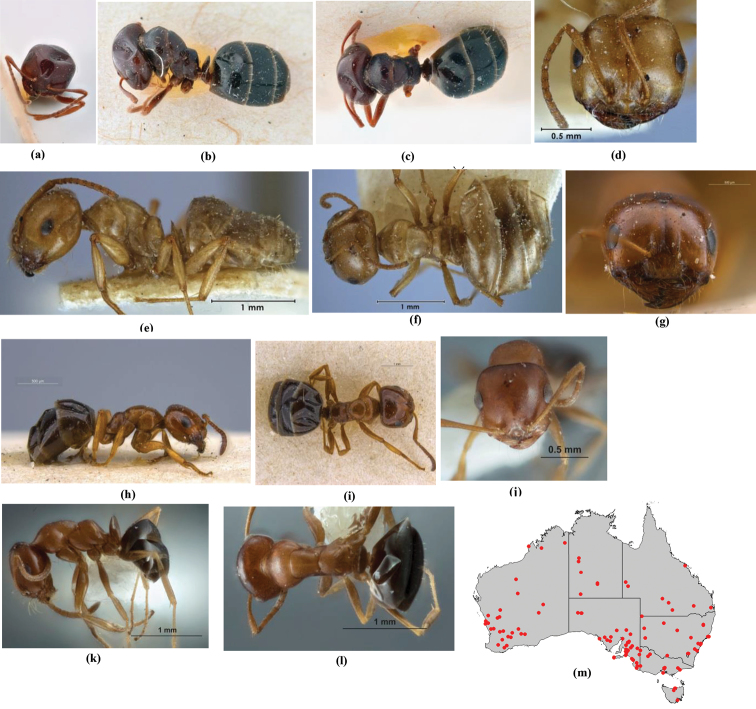
*Melophorus biroi* Forel: HNMH ‘*biroi*’ major worker holotype (probable) frons (**a**), profile (**b**) and dorsum (**c**); MHNG ‘*marius*’ major worker holotype frons (**d**), profile (**e**) and dorsum (**f**); ZHMB ‘*fieldipropinqua*’ major worker syntype (GBIF-D/FoCoL 2211) frons (**g**), profile (**h**) and dorsum (**i**); non–type minor worker (ANIC ANTS Vial 1.210) frons (**j**), profile (**k**) and dorsum (**l**); distribution map for the species for the species (**m**). Low resolution scale bars: 1 mm (**i**); 500µm (**g, h**); (scale bars not supplied on Automontage photographs (**a–c**).

### Melophorus castanopus

Taxon classificationAnimaliaHymenopteraFormicidae

Heterick, Castalanelli & Shattuck sp. n.

http://zoobank.org/73F42AF3-A2C1-46C8-81C6-B0604C08373F

#### Types.

Minor worker (middle ant) from 4 km S of Auburn, South Australia, 14 February 1974, P.J.M. Greenslade [ANIC32-900113] (ANIC). Paratypes: 2 minor workers on same pine and with same details as holotype (ANIC); major, media and minor worker from Bridgewater, South Australia, 23 September 1972, P.J.M. Greenslade (ANIC); major worker from Belair, South Australia, 20-23 December 1971, P.J.M. Greenslade, traps-7 (ANIC); 2 major and a minor worker from Calca, South Australia, 26 September 1957, B.B. Lowery, mallee scrub, ANIC Ants Vial 22.188 (MCZ); 3 minor workers from Harveys Return, Kangaroo Island, South Australia, 15 January 1973, P.J.M. Greenslade, (5) (MCZ); 3 major workers from Morialta Reserve, Lofty Ranges, South Australia, 8 September 1957, B.B. Lowery, 1000ft, dry sclerophyll, ANIC Ants Vial 22.197 [ANIC32-900114] (BMNH); major worker and dealate queen from Belair, South Australia, March 1971, P.J.M. Greenslade, Traps 1217 [ANIC32-900176] (SAM).

#### Other material examined.

**New South Wales**: 3 km N Kosciusko National Park (Lowery, B.B.), 40 km NNW Louth, Lake Mere (Greenslade, P.J.M.), 4 mi E Berry (Lowery, B.B.), 7 Mile Beach, Gerroa (Lowery, B.B.), Cousins Gully Road, Kioloa State Forest (Shattuck, S.O. [ANIC32-035780]), Fitzroy Falls (McAreavey, J.J.), Lane Cove River, Burns Bay, Sydney (Lowery, B.B.), Newholme Road, near Armidale (Sakurai, Y.), Pymble (Lowery, B.B.), Pymble (McAreavey, J.), South Black Ra., Tallaganda Natl. Pk., 14.5 km fr. Hoskinstown (Lambkin, C. & Starwick, N. [ANIC32-043214]), Whiporie, 55 km S Casino (York, A.). **South Australia**: 4 km N Bunker Hill, Kangaroo Island (Greenslade, P.J.M.), 4 km SW Auburn (Greenslade, P.J.M. [ANIC32-900113]), Belair (Greenslade, P.J.M.), Belair (Greenslade, P.J.M.), Belair (Greenslade, P.J.M. [ANIC32-900176]), Belair (Greenslade, P.J.M.), Belair (Greenslade, P.J.M.), Bridgewater (Baker, G.F.), Bridgewater (Baker, G.F.), Cleland, Mt. Lofty Ranges (Yeatman, E.), Jupiter Creek, 4 km W Echunga (Shattuck, S.O.), Mt. Lofty (Lowery, B.B.), Mt. Lofty Ranges, Adelaide (Lowery, B.B.), Mt. Lofty Ranges, Adelaide (Lowery, B.B.), N Sandy Creek, Kangaroo Island (Greenslade, P.J.M.), Para Wirra (Greenslade, P.J.M.), Rocky River, Kangaroo Island (Greenslade, P.J.M.), Rocky River, Kangaroo Island (Greenslade, P.J.M.), Salt Creek, Coorong (Greenslade, P.J.M.), Sandy Creek, Kangaroo Island (Greenslade, P.J.M.), Shackle Track, Kangaroo Island (Greenslade, P.J.M.). **Tasmania**: 3 km E Darlington, Maria Island (Lowery, B.B.). **Victoria**: 10 mi N Glenelg (Lowery, B.B.), Rotamah Island, Gippsland Lakes (Andersen, A.N.), Wangaratta (Bruce, W.A.).

#### Diagnosis.

*Melophorus castanopus* can be placed in the *M. biroi* species-group on the basis of characters of the clypeus, propodeum, mandible and palps. The species is placed in the *M. biroi* species-complex on the basis of a further suite of characters (viz, metatibia of major worker with only one preapical spur [except rarely in the *mjobergi* clade]; clypeal psammophore placed anteriorly at or just above anterior margin of clypeus in the minor worker and often in the major worker; head dorsoventrally compressed to varying degrees in the minor worker of most species with the eyes placed high on the sides; compact legs, and small body size [[(excluding *mjobergi* clade) HW of smallest minor 0.36 mm, average HW of smallest minors 0.46 mm; HW of largest known major 1.29 mm, average HW of largest majors (where known) 1.05 mm]). *Melophorus castanopus* can be most easily confused with *M. biroi* but the appearance of the major and minor workers, seen in profile, is slightly more flattened than the corresponding major and minor workers of the latter species, and the minor worker has erect setae on first gastral tergite, including a line of marginal setae. The minor worker mesosoma also possesses a couple to a few, usually stout erect setae (several specimens may need to be seen because of abrasion). Such setae are abundant and modified in a few isolated populations.

#### Minor worker description.

**Head.** Head square; posterior margin of head planar or weakly convex; frons shining with superficial shagreenation or microreticulation only; pilosity of frons a mixture of a few well-spaced, erect setae interspersed with appressed setae only. Eye moderate (eye length 0.20–0.49 length of side of head capsule); in full-face view, eye set at about midpoint of head capsule; in profile, eye set anteriad of head capsule; eyes elliptical or slightly reniform. In full-face view, frontal carinae straight or weakly convex; frontal lobes straight in front of antennal insertion. Anteromedial clypeal margin broadly and evenly convex; clypeal psammophore set at or just above anterior clypeal margin; palp formula 6,4. Five mandibular teeth in minor worker; mandibles triangular, weakly incurved; third mandibular tooth distinctly shorter than apical tooth and teeth numbers two and four; masticatory margin of mandibles approximately vertical or weakly oblique. **Mesosoma.** Integument of pronotum, mesonotum and mesopleuron with weak to moderate sheen, shagreenate on pronotum and dorsum of mesonotum, otherwise microreticulate; anterior mesosoma in profile smoothly rounded anteriad, thereafter pronotum and whole of mesonotum flattened and on same plane as propodeum; appearance of erect pronotal setae short and unmodified, or weakly expanded distally; in profile, metanotal groove shallow, broadly V or U-shaped; propodeum shining and shagreenate; propodeum angulate, propodeal angle blunt; length ratio of propodeal dorsum to its declivity about 1:1; erect propodeal setae always absent; appressed propodeal setulae short, separated by more than own length and inconspicuous; propodeal spiracle situated on or beside declivitous face of propodeum, and longer (length ≥ 0.50 × height of propodeum). **Petiole.** In profile, petiolar node squamiform; in full-face view, shape of petiolar node uniformly rounded; node shining and distinctly shagreenate-microreticulate. **Gaster.** Gaster shining, shagreenate (‘LP record’ appearance); pilosity of first gastral tergite consisting mainly of short, appressed setae, together with a few erect and semi-erect setae. **General characters.** Colour of most populations concolorous light to dark brown, a few populations with orange foreparts and dark brown gasters.

#### Major worker description.

**Head.** Head square; posterior margin of head planar or weakly concave; cuticle of frons shining and smooth except for piliferous pits and a few striolae around antennal insertions and Frontal carinae; pilosity of frons a mixture of a few well-spaced, erect setae interspersed with appressed setae only. Eye moderate, (eye length 0.20–0.49 length of head capsule); in full-face view, eyes set above midpoint of head capsule; in profile, eye set anteriad of midline of head capsule; eyes elliptical. In full-face view, frontal carinae straight, divergent posteriad; frontal lobes straight in front of antennal insertion. Anterior clypeal margin broadly and evenly convex; clypeal psammophore set at or just above anterior clypeal margin; palp formula 6,4. Five mandibular teeth in major worker; mandibles triangular, weakly incurved; third mandibular tooth distinctly shorter than apical tooth and teeth numbers two and four; masticatory margin of mandibles approximately aligned vertically or weakly oblique. **Mesosoma.** Integument of pronotum, mesonotum and mesopleuron with weak to moderate sheen, shagreenate on pronotum and dorsum of mesonotum, otherwise microreticulate; anterior mesosoma in profile smoothly rounded anteriad, thereafter pronotum and whole of mesonotum flattened and on same plane as propodeum; erect pronotal setae short and unmodified, or weakly expanded distally; in profile, metanotal groove shallow, indicated mainly by an angle and metathoracic spiracles; propodeum matt or with a weak sheen and microreticulate; propodeum angulate, propodeal angle blunt; length ratio of propodeal dorsum to its declivity between 1:1 and 1:2; erect propodeal setae present and sparse to moderate (1-12); appressed propodeal setae short, separated by more than own length and inconspicuous; propodeal spiracle situated on or beside declivitous face of propodeum, and shorter (length less than 0.50 × height of propodeum). **Petiole.** In profile, petiolar node squamiform; in full-face view, shape of petiolar node uniformly rounded; node shining and distinctly microreticulate. **Gaster.** Gaster shining, shagreenate (‘LP record’ appearance); pilosity of first gastral tergite consisting of well-spaced, erect and semi-erect setae interspersed with regularly spaced appressed setae. **General characters.** Colour brownish-orange with brown gaster to concolorous chocolate.

#### Measurements.

Worker (n = 8): CI 93–116; EI 20–29; EL 0.14–0.25; HL 0.51–0.11; HW 0.47–1.29; ML 0.69–1.50; MTL 0.36–0.77; PpH 0.07–0.16; PpL 0.28–0.67; SI 75–124; SL 0.58–0.96.

#### Comments.

*Melophorus castanopus* is a very common species in southeastern Australia, and occurs in NSW, SA, Vic and Tasmania (where it is one of a small handful of species of this heat-loving genus to have been recorded). The ant is very similar to *M. biroi*, but minor workers rarely have a glabrous mesosoma, unlike *M. biroi*, and they always have erect setae on the margin of the first gastral tergite (lacking in *M. biroi* minor workers). Major workers are more difficult to distinguish, but majors for *M. castanopus* have a gently convex pronotum (mostly flattened in *M. biroi* major workers). The genetics of the species are unknown as old wet material from SAMA did not produce a signal.

Habitat notes for the species indicate it has been taken in dry sclerophyll, mallee scrub, sandstone scrub and a grazed *Eucalyptus* dieback area. One sample was taken from ‘rock’(!) in dry sclerophyll woodland. The habits of this ant have not been recorded, but are likely to be similar to those of *M. biroi*.

#### Etymology.

Greek *kastanos* (‘chestnut-tree’; hence the colour) plus *pous* (‘foot’); adjective in the nominative singular.

**Figure 32.**
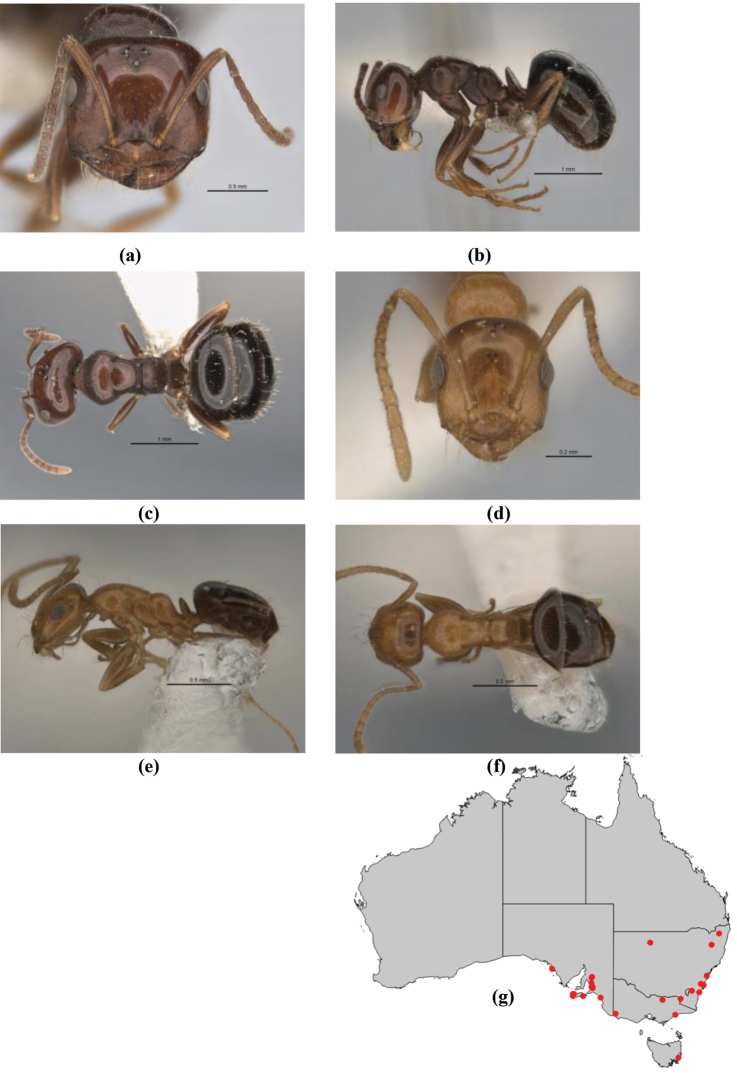
*Melophorus castanopus* sp. n.: major worker paratype (ANIC32-900176–top ant) frons (**a**), profile (**b**) and dorsum (**c**); minor worker holotype (ANIC32-900113–middle ant) frons (**d**), profile (**e**) and dorsum (**f**); distribution map for the species (**g**). Low resolution scale bars: 1 mm (**b, c**); 0.5 mm (**a, e, f**); 0.2 mm (**d**).

### Melophorus compactus

Taxon classificationAnimaliaHymenopteraFormicidae

Heterick, Castalanelli & Shattuck sp. n.

http://zoobank.org/DD477479-CBA5-40D1-9A1F-B70A90D66121

#### Types.

Holotype minor worker (top ant) from St George (‘Saint George’-*sic*), Queensland, 18 January 1966, B.B. Lowery, on black soil and savannah woodland, R 63 [ANIC32-900088] (ANIC). Paratypes: 2 minor workers on same pin and with same details as holotype (ANIC); 3 media workers and minor worker from St George (‘Saint George’-*sic*), Queensland, 18 January 1966, B.B. Lowery, [ANIC32-900169] (MCZ); 2 minor workers, 4 media workers, a major worker, a dealate queen and a male from St George (‘Saint George’-*sic*), Queensland, 18 January 1966, B.B. Lowery, [ANIC32-900089] (QM).

#### Other material examined.

**New South Wales**: Nyngan (Armstrong, J.).

#### Diagnosis.

*Melophorus compactus* can be placed in the *M. biroi* species-group on the basis of characters of the clypeus, propodeum, mandible and palps. The species is also placed in the *M. mjobergi* clade because of characters best seen in the major worker. These include (seen in full-face view) the deeply recessed area around the frontal carinae and medial sector of clypeus visible in the major and media workers, the psammophore generally placed on or just above anterior margin of clypeus, and the minor workers hairy with bristly, short erect setae. *Melophorus compactus* can then be distinguished from *M. mjobergi* and *M. postlei* by the following characters: head of major worker without distinct sculpture and strongly shining, the setae-bearing sockets on head (if visible) appearing as tiny pits only, the sculpture also reduced on the head of media worker, which is moderately shining, in full-face view, the antennal carina of the major worker distinctly extended posteriad past the antennal insertion, the posterior extension of the carina straight, and the eye of the minor worker placed medially on the head capsule and large in size (in profile, eye length ≈ 0.40 × length of side of head capsule).

#### Minor worker description.

**Head.** Head approximately oval with straight sides; posterior margin of head extended posteriad as a convex, sloping surface with a slight medioccipital protuberance; frons matt or with weak sheen, microreticulate or microreticulate-shagreenate; frons consisting exclusively or almost exclusively of well-spaced, appressed setae only (small, erect setae, if present, usually confined to ocular triangle or posterior margin of head). Eye moderate (eye length 0.20–0.49 length of side of head capsule); in full-face view, eyes set above midpoint of head capsule; in profile, eye set anteriad of midline of head capsule; eyes elliptical or slightly reniform. In full-face view, frontal carinae straight or weakly convex; frontal lobes straight in front of antennal insertion. Anteromedial clypeal margin narrowly convex and protruding, clypeal margin entire or very weakly indented, or narrowly convex and protruding anteromedially, clypeal midpoint distinctly notched; clypeal psammophore set at or just above anterior clypeal margin; palp formula 6,4. Four or five mandibular teeth in minor worker; mandibles triangular, weakly incurved; third mandibular tooth distinctly shorter than apical tooth and teeth numbers two and four; masticatory margin of mandibles approximately vertical or weakly oblique. **Mesosoma.** Integument of pronotum, mesonotum and mesopleuron with weak to moderate sheen, shagreenate on pronotum and dorsum of mesonotum, otherwise microreticulate; anterior mesosoma in profile broadly convex; erect pronotal setae absent; in profile, metanotal groove deep, ‘V’-shaped; propodeum shining and shagreenate; propodeum smoothly rounded or with indistinct angle, or uniformly flattened along an oblique trajectory; propodeal dorsum and declivity confluent; erect propodeal setae always absent; appressed propodeal setulae long and closely aligned, creating pubescence, or long, each reaching setae behind and in front, but not forming pubescence; propodeal spiracle situated on or beside declivitous face of propodeum, and shorter (length < 0.50 × height of propodeum). **Petiole.** In profile, petiolar node rectangular, vertex blunt, directed posteriad; in full-face view, shape of petiolar node uniformly rounded; node shining and smooth with vestigial sculpture. **Gaster.** Gaster shining, shagreenate (‘LP record’ appearance); pilosity of first gastral tergite consisting of well-spaced, long, whitish, appressed setae only, erect setae always absent. **General characters.** Colour reddish-brown with brown gaster through to concolorous chocolate

#### Major worker description.

**Head.** Head quadrate (i.e., heart-shaped); posterior margin of head weakly concave; cuticle of frons matt or with weak sheen, microreticulate; pilosity of frons a mixture of a few well-spaced, erect setae interspersed with appressed setae only, or consisting exclusively or almost exclusively of well-spaced, appressed setae only (small, erect setae, if present, usually confined to ocular triangle or posterior margin of head). Eye small (eye length less than 0.2 × length of head capsule); in full-face view, eyes set above midpoint of head capsule; in profile, eye set anteriad of midline of head capsule; eyes elliptical. In full-face view, frontal carinae straight or weakly convex; frontal lobes straight in front of antennal insertion. Anterior clypeal margin narrowly convex and protruding anteromedially, clypeal margin entire or weakly indented; clypeal psammophore set at or just above anterior clypeal margin; palp formula 6,4. Four mandibular teeth in major worker; mandibles triangular, weakly incurved; third mandibular tooth distinctly shorter than apical tooth and teeth numbers two and four; masticatory margin of mandibles approximately aligned vertically or weakly oblique. **Mesosoma.** Integument of pronotum, mesonotum and mesopleuron with weak to moderate sheen, shagreenate on pronotum and dorsum of mesonotum, otherwise microreticulate; anterior mesosoma in profile broadly convex; erect pronotal setae short and unmodified, or weakly expanded distally; in profile, metanotal groove shallow, broadly V- or U-shaped; propodeum matt or with a weak sheen and microreticulate; propodeum smoothly rounded or with indistinct angle, or uniformly flattened along an oblique trajectory; propodeal dorsum and declivity confluent; erect propodeal setae absent; appressed propodeal setae long and closely aligned, creating pubescence, or long and separated by at least own length; propodeal spiracle situated on or beside declivitous face of propodeum, and shorter (length less than 0.50 × height of propodeum). **Petiole.** In profile, petiolar node squamiform; in full-face view, shape of petiolar node uniformly rounded; node shining and faintly shagreenate-microreticulate. **Gaster.** Gaster shining, shagreenate (‘LP record’ appearance); pilosity of first gastral tergite consisting of well-spaced, erect and semi-erect setae interspersed with regularly spaced appressed setae. **General characters.** Colour from medium brown with darker gaster to blackish-crimson.

#### Measurements.

Worker (n = 4): CI 98–107; EI 24–27; EL 0.14–0.22; HL 0.53–0.86; HW 0.52–0.92; ML 0.77–1.12; MTL 0.45–0.52; PpH 0.05–0.09; PpL 0.30–0.46; SI 65–129; SL 0.60–0.67.

#### Comments.

This rather uncommon species is distinguished from other members of the complex by its large eyes, reduced sculpture, the shining appearance of the head capsule without distinct pitting in the major worker, and the well-developed frontal carinae. Minor workers, however, closely resemble those of *M. postlei*, albeit the compound eye is much larger. *Melophorus compactus* is known from a handful of collections in NSW and QLD. This is the most southerly occurring member of the complex among those species with a recessed gena in the major and media worker. A worker collected by Rev. Lowery in St George, QLD came from savannah woodland over black soil plain. No specimens were available for sequencing and nothing more is known of the species.

#### Etymology.

Latin *compactus* (‘thick’); adjective in the nominative singular.

**Figure 33.**
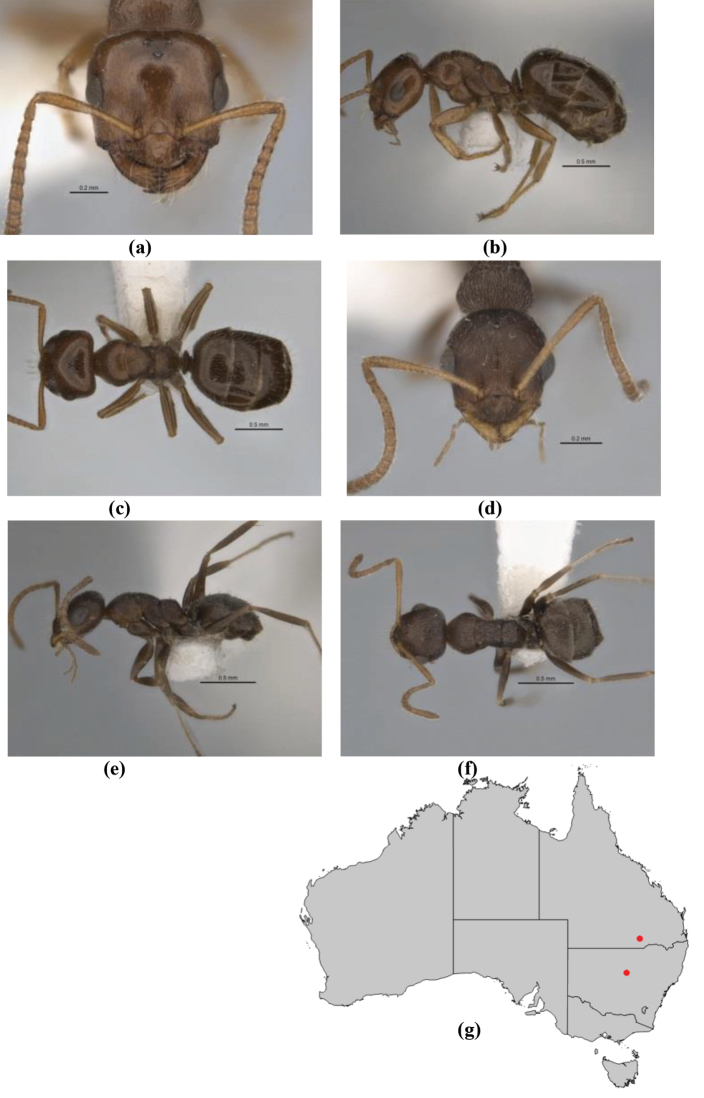
*Melophorus compactus* sp. n.: major worker paratype (ANIC32-900169–third ant from top) frons (**a**), profile (**b**) and dorsum (**c**); minor worker holotype (ANIC32-900088) frons (**d**), profile (**e**) and dorsum (**f**); distribution map for the species (**g**). Low resolution scale bars: 0.5 mm (**b, c**, **e, f**); 0.2 mm (**a, d**).

### Melophorus cuneatus

Taxon classificationAnimaliaHymenopteraFormicidae

Heterick, Castalanelli & Shattuck sp. n.

http://zoobank.org/2C0FD278-DD7E-449C-B038-7D359FEBC350

#### Types.

Minor worker holotype (bottom ant) from Binningup 33°08'46"S, 143°43'15"E, Western Australia, 1 January 2007, Heterick, B.E., coastal scrubland, nest in small tussock [JDM32-001958] (WAM). Paratypes: 2 major workers on same pin and with same details as holotype (WAM); minor worker from Bold Park 31°56'S, 115°46'E, Western Australia, 19–22 March 2004, S. Callan [JDM32-001962] (ANIC); 2 major workers from Jandakot Regional Park 32°06'S, 115°53'E, Western Australia, 28 April 2002, N. Gunawardene, Pitfall trap T2, S7 [JDM32-001960] (BMNH).

#### Other material examined.

**Western Australia**: Jandakot Reg. Pk. (Gunawardene, N. [JDM32-001960]), Reabold Hill (Majer, J.D. [JDM32-001959]).

#### Diagnosis.

*Melophorus cuneatus* can be placed in the *M. biroi* species-group on the basis of characters of the clypeus, propodeum, mandible and palps. The species is placed in the *M. biroi* species-complex on the basis of a further suite of characters (viz, metatibia of major worker with only one preapical spur [except rarely in the *mjobergi* clade]; clypeal psammophore placed anteriorly at or just above anterior margin of clypeus in the minor worker and often in the major worker; head dorsoventrally compressed to varying degrees in the minor worker of most species with the eyes placed high on the sides; compact legs, and small body size [excluding *mjobergi* clade] HW of smallest minor 0.36 mm, average HW of smallest minors 0.46 mm; HW of largest known major 1.29 mm, average HW of largest majors [where known] 1.05 mm). *Melophorus cuneatus* cannot be confused with any other other *Melophorus* except, perhaps, for *M. paramorphomenus*, which, however, has the diagnostic characters of the *M. fieldi* complex. In profile, the propodeal dorsum of minor and media workers of *M. cuneatus* is extremely narrow and almost acuminate, the metanotal groove in all workers is narrowly and deeply impressed, the mesonotum of the minor worker is hypertrophied so that the metathoracic spiracle is situated on its underside in a distinctly lateral position and all workers possess weak pubescence and a moderate number of erect setae on the mesosoma.

#### Minor worker description.

**Head.** Head square; posterior margin of head planar or weakly convex, or planar or weakly concave; frons matt or with weak sheen, microreticulate or microreticulate-shagreenate; pilosity of frons a mixture of a few well-spaced, erect setae interspersed with appressed setae only. Eye moderate (eye length 0.20–0.49 length of side of head capsule); in full-face view, eyes set above midpoint of head capsule; in profile, eye set anteriad of midline of head capsule; eyes elliptical or slightly reniform. In full-face view, frontal carinae concave; frontal lobes straight in front of antennal insertion. Anteromedial clypeal margin straight, or broadly and evenly convex; clypeal psammophore set at or just above anterior clypeal margin; palp formula 6,4. Five mandibular teeth in minor worker; mandibles triangular, weakly incurved; third mandibular tooth distinctly shorter than apical tooth and teeth numbers two and four; masticatory margin of mandibles approximately vertical or weakly oblique. **Mesosoma.** Integument of pronotum, mesonotum and mesopleuron matt or with weak sheen and microreticulate throughout; anterior mesosoma in profile smoothly rounded anteriad, thereafter pronotum and whole of mesonotum flattened and on same plane as propodeum; appearance of erect pronotal setae short, (i.e., longest erect setae shorter than length of eye) and unmodified; in profile, metanotal groove a narrow but deep slit; propodeum matt or with a weak sheen and microreticulate-striolate; propodeum wedge-shaped, tapering dorsad; length ratio of propodeal dorsum to its declivity not applicable, propodeal dorsum reduced to a narrow sliver; erect propodeal setae variable in number, may be absent; appressed propodeal setulae long and closely aligned, creating pubescence; propodeal spiracle situated on or beside declivitous face of propodeum, and longer (length ≥ 0.50 × height of propodeum). **Petiole.** In profile, petiolar node squamiform; in full-face view, shape of petiolar node uniformly rounded; node shining and smooth with vestigial sculpture. **Gaster.** Gaster weakly shining with indistinct shagreenation; pilosity of first gastral tergite consisting of thick, appressed setae that form pubescence, interspersed with numerous short, bristly, erect setae. **General characters.** Colour of foreparts chocolate to black, gaster dark brown to black.

#### Major worker description.

**Head.** Head square; posterior margin of head weakly concave; cuticle of frons shining with superficial shagreenation or microreticulation only; pilosity of frons a mixture of a few well-spaced, erect setae interspersed with appressed setae only. Eye moderate (eye length 0.20–0.49 length of head capsule); in full-face view, eyes set above midpoint of head capsule; in profile, eye set anteriad of midline of head capsule; eyes elliptical. In full-face view, frontal carinae straight, divergent posteriad; frontal lobes straight in front of antennal insertion. Anterior clypeal margin broadly emarginate; clypeal psammophore set at or just above anterior clypeal margin; palp formula 6,4. Five mandibular teeth in major worker; mandibles triangular, weakly incurved; third mandibular tooth distinctly shorter than apical tooth and teeth numbers two and four; masticatory margin of mandibles approximately aligned vertically or weakly oblique. **Mesosoma.** Integument of pronotum, mesonotum and mesopleuron shining and uniformly microreticulate, with shallow dimpling evident on the dorsum of the mesonotum; anterior mesosoma in profile steeply rounded anteriad, thereafter pronotum and whole of mesonotum flattened and on a higher plane than propodeum; erect pronotal setae short, (i.e., shorter than length of eye) and unmodified; in profile, metanotal groove a narrow but deep slit; propodeum shining and microreticulate; propodeum always smoothly rounded; propodeal dorsum and declivity confluent; erect propodeal setae present and sparse to moderate (1-12); appressed propodeal setae long, each reaching setae behind and in front, but not forming pubescence; propodeal spiracle situated on or beside declivitous face of propodeum, and shorter (length less than 0.50 × height of propodeum). **Petiole.** In profile, petiolar node squamiform; in full-face view, shape of petiolar node uniformly rounded, or generally rounded with median indentation; node shining and smooth with vestigial microreticulation anteriad. **Gaster.** Gaster shining, shagreenate (‘LP record’ appearance); pilosity of first gastral tergite consisting of longish, closely aligned, appressed setae interspersed with short, bristly, erect setae (some distally flattened). **General characters.** Colour of foreparts russet to dark reddish-brown, gaster dark brown to brownish-black.

#### Measurements.

Worker (n = 4): CI 98–115; EI 19–27; EL 0.14–0.23; HL 0.52–1.06; HW 0.52–1.23; ML 0.77–1.35; MTL 0.45–0.73; PpH 0.05–0.12; PpL 0.30–0.59; SI 73–129; SL 0.67–0.89.

#### Comments.

*Melophorus cuneatus* is confined to the southwest of Western Australia. All known records have been from the Swan Coastal Plain. This ant is nowhere common, but appears occasionally in collections from relictual bushland in the Perth metropolitan area as well as localities to the south as far as Binningup. The appearance of the mesosoma, in particular the wedge-shaped propodeum, distinguishes minor workers of this ant from all others. The major worker is less spectacular but has weak pubescence on the gaster and a deeply impressed metanotal groove, both of which serve to differentiate it from other members of the complex that occur in the same area. No material has been available for sequencing, but the morphology suggests a close relationship to *M. propebiroi*. There are no ecological notes on labels for this species, but the localities where it has been found support dry sclerophyll woodland.

#### Etymology.

Latin *cuneatus* (‘wedge-shaped’); adjective in the nominative singular.

**Figure 34.**
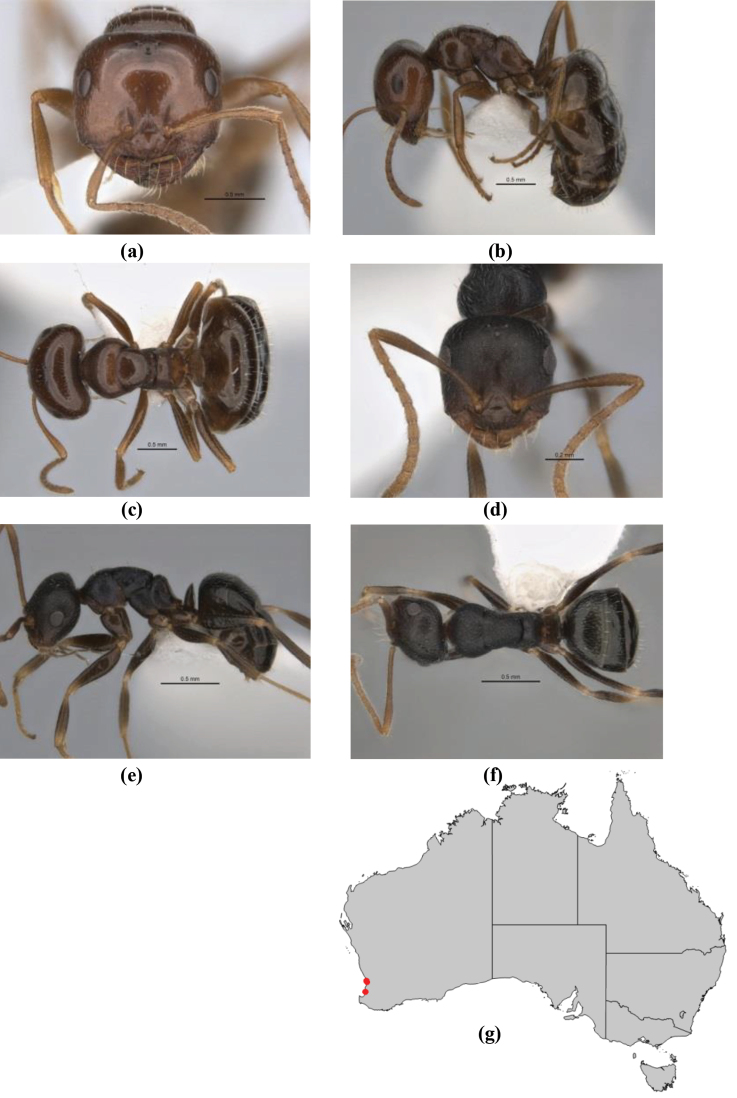
*Melophorus cuneatus* sp. n.: major worker paratype (JDM32-001958–top ant) frons (**a**), profile (**b**) and dorsum (**c**); minor worker holotype (JDM32-001958–bottom ant) frons (**d**), profile (**e**) and dorsum (**f**); distribution map for the species (**g**). Low resolution scale bars: 0.5 mm (**a–c, e, f**); 0.2 mm (**d**).

### Melophorus dicyrtos

Taxon classificationAnimaliaHymenopteraFormicidae

Heterick, Castalanelli & Shattuck sp. n.

http://zoobank.org/2B2EAA06-1822-4A8E-BDF2-12F98634563C

#### Types.

Holotype minor worker (bottom ant) from slopes above Baroalba Spring 12.475S, 132.51E, Northern Territory, 17 November 1972, R.W.Taylor & J.E. Feehan, *Euc.* Savanna, Acc. 72.1006, ANIC ANTS VIAL 38.92 [ANIC32-900177] (ANIC). Paratypes: 2 major workers on same pin and with same details as holotype (ANIC); major and 2 minor workers from Gove Nhulumbuy, Northern Territory, 1 November 1972, R.W. Taylor Accession 72.824, ANIC Ants Vial 15.198 [ANIC32-002772] (ANIC); 2 minor and major worker from 22 km SW of Katherine, Northern Territory, 26 October 1977, P.J.M. Greenslade (2), 10 (BMNH); 3 media workers from slopes above Baroalba Spring 12.475S, 132.51E, Northern Territory, 16 November 1972, R.W.Taylor & J.E. Feehan, *Euc.* Savanna, Acc. 72.1015, ANIC ANTS VIAL 38.104 [ANIC32-900115] (MCZ); major worker and 2 minor workers from Horn Island 10.37S, 142.17E, Torres Strait, 10-27 June 1974, H. Heatwole & E. Cameron, No. HOR.64 [ANIC32-900116] (QM); minor, media and major worker from Yampi 2 Stn, Kimberley Area, Western Australia, C. Palmer, May 2002 [JDM32-004529] (WAM).

#### Other material examined.

**Northern Territory**: 1 km NNE Bullita, Gregory Nat. Park (Weir, T. & Bouchard, P. [ANIC32-051712]), 22 km WSW Borroloola (Feehan, J.E.), Caranbirini Water Hole, 33 km SW Borroloola (Feehan, J.E.), Jabiru (Greenslade, P.J.M.), Jabiru, Alligator Rivers area (Greenslade, P.J.M.), Jabiru, Alligator Rivers area (Greenslade, P.J.M.), Kapalga, Alligator Rivers area (Greenslade, P.J.M.). **Queensland**: 13 km E by S Weipa (Zborowski, P. & Shattuck, S.O. [ANIC32-043330]), 3.5 km SWbyS Mt. Baird (Naumann, I.D.), Clermont, 7.5 km E (Burwell, C.), Hann River (Zborowski, P. & Horak, M. [ANIC32-031071]), Lolworth NP (Wright, S.), Proserpine, Airport Drive (Raven, R.), Red Falls (Monteath, G.), Somerset, Cape York (Cameron, E.), Weipa (Majer, J.D.). **Western Australia**: Argyle Diamond Mine via Kununurra (Postle, A.T. [JDM32-004621]), Argyle Diamond Mine via Kununurra (Postle, A. [JDM32-004988]), Marillana Ck (Dunlop, N. [JDM32-005034]), Mt. Whaleback (Walker, K.J. [JDM32-004942]), Zuytdorp (WAM/CALM [JDM32-001975]).

#### Diagnosis.

*Melophorus dicyrtos* can be placed in the *M. biroi* species-group on the basis of characters of the clypeus, propodeum, mandible and palps. The species is placed in the *M. biroi* species-complex on the basis of a further suite of characters (viz, metatibia of major worker with only one preapical spur [except rarely in the *mjobergi* clade]; clypeal psammophore placed anteriorly at or just above anterior margin of clypeus in the minor worker and often in the major worker; head dorsoventrally compressed to varying degrees in the minor worker of most species with the eyes placed high on the sides; compact legs, and small body size [[(excluding *mjobergi* clade) HW of smallest minor 0.36 mm, average HW of smallest minors 0.46 mm; HW of largest known major 1.29 mm, average HW of largest majors (where known) 1.05 mm]). *Melophorus dicytos* can be recognised and distinguished from species of similar appearance (e.g., *M. biroi* and *M. castanopus*) by virtue of the propodeum of the minor and major worker being protuberant, strongly truncate, and with an elevated dorsal surface which is much shorter than its declivitous surface. This is also a relatively large member of the *M. biroi* complex (HW of minor worker 0.65 mm ≥, HW of major worker 1.25 mm ≥) and is largely tropical (*M. castanopus* in contrast is largely southeastern in distribution and apparently does not occur in NT, QLD and WA, so is probably not sympatric with *M. dicyrtos*). The metanotal groove characteristically is a deep V-shaped notch and most workers have long, flexuous setae on the mesosomal surface but these may be short and bristly.

#### Minor worker description.

**Head.** Head square; posterior margin of head planar or weakly convex, or planar or weakly concave; frons shining with superficial shagreenation or microreticulation only; pilosity of frons a mixture of a few well-spaced, erect setae interspersed with appressed setae only. Eye moderate (eye length 0.20–0.49 length of side of head capsule); in full-face view, eyes set above midpoint of head capsule; in profile, eye set anteriad of midline of head capsule; eyes elliptical or slightly reniform. In full-face view, frontal carinae straight, divergent posteriad; frontal lobes straight in front of antennal insertion. Anteromedial clypeal margin broadly and evenly convex; clypeal psammophore set at or just above anterior clypeal margin; palp formula 6,4. Five mandibular teeth in minor worker; mandibles triangular, weakly incurved; third mandibular tooth distinctly shorter than apical tooth and teeth numbers two and four; masticatory margin of mandibles approximately vertical or weakly oblique. **Mesosoma.** Integument of pronotum, mesonotum and mesopleuron shining and smooth on dorsum, entire lower mesopleuron distinctly striolate-microreticulate, or with weak to moderate sheen and superficial microreticulation (more pronounced on mesopleuron); anterior mesosoma in profile pronotum smoothly rounded anteriad and flattened posteriad, mesonotum narrowly convex; appearance of erect pronotal setae long (i.e., longest erect setae longer than length of eye) and unmodified, or short, (i.e., longest erect setae shorter than length of eye) and unmodified; in profile, metanotal groove deep, ‘V’-shaped; propodeum shining and finely striolate and microreticulate; propodeum angulate, propodeal angle blunt, or distinctly angulate, propodeal angle sharp; length ratio of propodeal dorsum to its declivity greater than 1:2; erect propodeal setae variable in number, may be absent; appressed propodeal setulae long and separated by at least own length, or short, separated by more than own length and inconspicuous; propodeal spiracle situated on or beside declivitous face of propodeum, and longer (length ≥ 0.50 × height of propodeum), or situated on or beside declivitous face of propodeum, and shorter (length < 0.50 × height of propodeum). **Petiole.** In profile, petiolar node squamiform; in full-face view, shape of petiolar node uniformly rounded; node shining and smooth with vestigial sculpture. **Gaster.** Gaster shining, shagreenate (‘LP record’ appearance); pilosity of first gastral tergite consisting of well-spaced, erect and semi-erect setae interspersed with regularly placed appressed setae. **General characters.** Colour concolorous orange tan to dark reddish-brown.

#### Major worker description.

**Head.** Head square; posterior margin of head weakly concave; cuticle of frons striolate anteriad, smooth and shining posteriad; pilosity of frons a mixture of a few well-spaced, erect setae interspersed with appressed setae only. Eye moderate (eye length 0.20–0.49 length of head capsule); in full-face view, eyes set above midpoint of head capsule; in profile, eye set anteriad of midline of head capsule; eyes elliptical. In full-face view, frontal carinae straight or weakly convex in full-face view, or straight, divergent posteriad; frontal lobes curved inward in front of antennal insertion. Anterior clypeal margin broadly convex with anteromedial dimple; clypeal psammophore set at or just above anterior clypeal margin; palp formula 6,4. Five mandibular teeth in major worker; mandibles triangular, weakly incurved; third mandibular tooth distinctly shorter than apical tooth and teeth numbers two and four; masticatory margin of mandibles approximately aligned vertically or weakly oblique. **Mesosoma.** Integument of pronotum, mesonotum and mesopleuron with weak to moderate sheen, shagreenate on pronotum and dorsum of mesonotum, otherwise microreticulate; anterior mesosoma in profile broadly convex; erect pronotal setae long (i.e., longer than length of eye) and unmodified; in profile, metanotal groove deep, V-shaped; propodeum matt or with weak sheen and microreticulate-striolate; propodeum angulate, propodeal angle blunt; length ratio of propodeal dorsum to its declivity greater than 1:2; erect propodeal setae present and sparse to moderate (1-12); appressed propodeal setae short, separated by more than own length and inconspicuous; propodeal spiracle situated on or beside declivitous face of propodeum, and shorter (length less than 0.50 × height of propodeum). **Petiole.** In profile, petiolar node squamiform; in full-face view, shape of petiolar node uniformly rounded; node shining and faintly striolate and microreticulate. **Gaster.** Gaster shining, shagreenate (‘LP record’ appearance); pilosity of first gastral tergite consisting of well-spaced, erect and semi-erect setae interspersed with regularly spaced appressed setae. **General characters.** Colour of foreparts orange tan to dark reddish-black, gaster dark chocolate to blackish.

#### Measurements.

Worker (n = 6): CI 100–117; EI 19–26; EL 0.17–0.24; H 0.66–1.09L; HW 0.66–1.28; ML 0.91–1.42; MTL 0.53–0.84; PpH 0.12–0.13; PpL 0.38–0.58; SI 81–120; SL 0.79–1.04.

#### Comments.

*Melophorus dicyrtos* is preeminently a species of the northern half of Australia, and most samples collected come from the Torresian phytogeographic zone. A dry husk of a worker that is doubtfully placed in this species and that comes from the Zuytdorp area near Kalbarri, WA, represents the most southerly record for the ant, which has been collected in the NT, QLD and WA. No specimens were available for sequencing. Although small, this is a reasonably spectacular species from the *biroi* clade; with its shining cuticle, rounded mesonotum, sharply incised and v-shaped metanotal groove, and its truncate propodeum with an acutely angled division between the dorsal and declivitous faces helping to distinguish the species from similar ants. Several worker samples have been taken in flight intercept traps, suggesting the ant may climb on vegetation. Two NT samples were taken in eucalyptus savannah, and material from the QM has been collected in yellow pan traps in vinescrub growth over yellow basalt, from a faeces-baited pitfall trap in eucalypt woodland, and from closed woodland including one sample from a dung trap. The association of a *Melophorus* species with dung is likely to be merely fortuitous (Burwell, C., pers. comm.).

#### Etymology.

Greek *dikyrtos* (‘two-humped’); adjective in the nominative singular.

**Figure 35.**
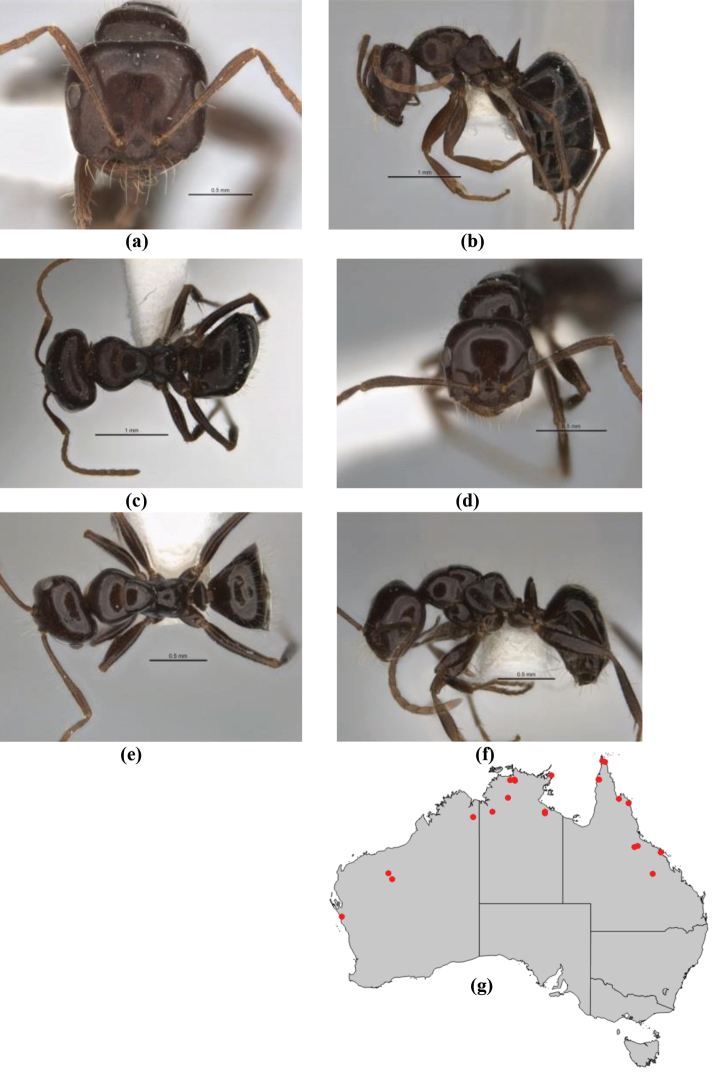
*Melophorus dicyrtos* sp. n.: major worker paratype (ANIC ANTS VIAL 38.92 [ANIC32-900177]–top ant) frons (**a**), profile (**b**) and dorsum (**c**); minor worker holotype (ANIC ANTS VIAL 38.92 [ANIC32-900177]–bottom ant) frons (**d**), profile (**e**) and dorsum (**f**); distribution map for the species (**g**). Low resolution scale bars: 1 mm (**b, c**); 0.5 mm (**a, d–f**).

### Melophorus graciliceps

Taxon classificationAnimaliaHymenopteraFormicidae

Heterick, Castalanelli & Shattuck sp. n.

http://zoobank.org/76F8E129-AA19-498A-8E2B-FDD57235C147

#### Types.

Holotype minor worker (bottom ant) from Heathcote, near Bendigo, Victoria, 26 May 1961, B.B. Lowery, 1000 ft, dry sclerophyll, ANIC ants vial 20.238 [ANIC32-900175] (ANIC). Paratypes: 2 major workers on same pin and with same details as Holotype (ANIC); 1 major and 2 minor workers from Heathcote near Bendigo, Victoria, 29 May 1961, B.B. Lowery, 1000ft, dry sclerophyll, ANIC Ants Vial 20.239 (BMNH); 3 minor workers from Yathong Nature Reserve 145°40'33"S, 32°37'39"S, New South Wales, 3-25 October 2003, C. Lambkin & N. Starwick, 205m, ANIC Bulk Sample 2154, open woodland, field of flowers, Malaise [ANIC32-030855] (MCZ); 6 major and 2 minor workers from St George, Queensland, 7 January 1966, B.B. Lowery, ‘*Melophorus*’, Reddish soil, savannah woodland, R10 (back of label–‘strange nest–deep narrow cone, smooth, a perfect insect trap’) (QM); 2 major and one minor worker from Calca, South Australia, 26 September 1957, B.B. Lowery, mallee scrub, ANIC Ants Vial 22.188 (SAM); 2 major workers from Westonia, Western Australia, coll. approx. 2000; Environmental Biology Department, Curtin University of Technology, [JDM32-001979] (WAM).

#### Other material examined.

**New South Wales**: 24 km E Rankins Spring (Lowery, B.B.), 4 km N Coombah Road House, Route 79 (Crozier, R.H., Imai, H.T. & Ward, P.S.), CSIRO Lake Mere Field Station, near Louth (Bryannah, M.), **Queensland**: ‘Merigol’ (Beutel, T.). **South Australia**: Brookfield Conservation park (Shattuck, S.O. [ANIC32-900110]), Cambrai (Greenslade, P.J.M.), Cambrai (Greenslade, P.J.M.), Cambrai (Greenslade, P.J.M.). **Victoria**: Sea Lake (Goudie [ANIC32-900111]). **Western Australia**: 30 km N Billabong RH (Heterick, B.E. [M25/M35/M36]), 7 km E of Burakin (Heterick, B.E. [JDM32-001978]), Dryandra SF (Norwood, C. [JDM32-001980]).

#### Diagnosis.

*Melophorus graciliceps* can be placed in the *M. biroi* species-group on the basis of characters of the clypeus, propodeum, mandible and palps. The species is also placed in the *M. mjobergi* clade but differs from the most derived species, and the major worker does not have the deeply recessed area around the frontal carinae and medial sector of clypeus or the impressed setae-bearing sockets visible in the major and media workers that is seen in *M. mjobergi*, *M. postlei* and *M. compactus*. Minor and major workers workers of *M. graciliceps* are clothed with fine, appressed silvery setae that form pubescence in the minor worker, at least, in conjunction with multiple scattered, modified erect setae (the modified setae varying from distally slightly flattened to clavate) on the head, mesosoma and gaster. This species can only be confused with its near relation, *M. lissotriches*, but differs in having an only weakly convex eye in the minor worker (strongly convex in *M. lissotriches*), the eye barely interrupting the outline of head capsule. In profile, the pronotum of the minor worker rises gently, and the mesosoma is thereafter more-or-less straight, the mesonotum does not dip towards the propodeum and the metanotal groove is not demarcated by a v-shaped notch. The frontal carinae of the major worker is concave (straight or weakly convex in *M. graciliceps*) and the cuticle of its head is smooth and shining (matt or weakly shining in *M. graciliceps*).

#### Minor worker description.

**Head.** Head square; posterior margin of head planar to strongly convex; frons shining with superficial shagreenation or microreticulation only; frons consisting almost completely of appressed setae that may form pubescence (tiny, erect setae, if present, usually confined to ocular triangle). Eye moderate (eye length 0.20–0.49 length of side of head capsule); in full-face view, eyes set above midpoint of head capsule; in profile, eye set anteriad of midline of head capsule; eyes elliptical or slightly reniform. In full-face view, frontal carinae straight or weakly convex; frontal lobes straight in front of antennal insertion. Anteromedial clypeal margin broadly and evenly convex; clypeal psammophore set at or just above anterior clypeal margin; palp formula 6,4. Five mandibular teeth in minor worker; mandibles triangular, weakly incurved; third mandibular tooth distinctly shorter than apical tooth and teeth numbers two and four; masticatory margin of mandibles approximately vertical or weakly oblique. **Mesosoma.** Integument of pronotum, mesonotum and mesopleuron with weak to moderate sheen and superficial microreticulation (more pronounced on mesopleuron); anterior mesosoma in profile weakly elevated anteriad, thereafter gently sinuate, pronotum and mesonotum on same plane; appearance of erect pronotal setae short and often expanded distally, at times clavate; in profile, metanotal groove a weak or vestigial furrow; propodeum shining and shagreenate; propodeum smoothly rounded or with indistinct angle; propodeal dorsum and declivity confluent; erect propodeal setae present and abundant (greater than 12), or present and sparse to moderate (1-12); appressed propodeal setulae long and closely aligned, creating pubescence; propodeal spiracle situated on or beside declivitous face of propodeum, and longer (length ≥ 0.50 × height of propodeum). **Petiole.** In profile, petiolar node squamiform; in full-face view, shape of petiolar node square with sharp angles; node shining and distinctly shagreenate-microreticulate. **Gaster.** Gaster weakly shining with indistinct shagreenation; pilosity of first gastral tergite consisting of thick, appressed setae that form pubescence, interspersed with numerous short, modified (sometimes clavate), erect setae. **General characters.** Colour brown to blackish-brown.

#### Major worker description.

**Head.** Head square; posterior margin of head planar or weakly concave; cuticle of frons shining and smooth except for piliferous pits; frons consisting mainly of well-spaced appressed and a few mainly modified, clavate (feather-like) erect setae. Eye moderate (eye length 0.20–0.49 length of head capsule); in full-face view, eyes set above midpoint of head capsule; in profile, eye set anteriad of midline of head capsule; eyes elliptical. In full-face view, frontal carinae straight, divergent posteriad; frontal lobes straight in front of antennal insertion. Anterior clypeal margin broadly and evenly convex; clypeal psammophore set below midpoint of clypeus; palp formula 6,4. Five mandibular teeth in major worker; mandibles triangular, weakly incurved; third mandibular tooth distinctly shorter than apical tooth and teeth numbers two and four; masticatory margin of mandibles approximately aligned vertically or weakly oblique. **Mesosoma.** Integument of pronotum, mesonotum and mesopleuron shining and mainly smooth, vestigial shagreenation most noticeable on humeri and mesopleuron; anterior mesosoma in profile broadly convex; erect pronotal setae short and often expanded distally, at times clavate; in profile, metanotal groove shallow, broadly V- or U-shaped; propodeum shining and generally smooth, with only weak, indistinct shagreenation; propodeum always smoothly rounded; propodeal dorsum and declivity confluent; erect propodeal setae present and abundant (at least a dozen); appressed propodeal setae long and closely aligned, creating pubescence; propodeal spiracle situated on or beside declivitous face of propodeum, and longer (length ≥ 0.50 × height of propodeum). **Petiole.** In profile, petiolar node squamiform; in full-face view, shape of petiolar node square with rounded angles; node shining and faintly shagreenate-microreticulate. **Gaster.** Gaster shining with superficial microreticulation; pilosity of first gastral tergite consisting of thick, often distally flattened, erect setae over well-spaced, short, appressed setae. **General characters.** Colour reddish-orange, gaster brown.

#### Measurements.

Worker (n = 8): CI 90-117; EI 20-32; EL 0.19-0.31; HL 0.67-1.32; HW 0.60-1.55; ML 0.98-1.87; MTL 0.71-0.95; PpH 0.09-0.15; PpL 0.43-0.69; SI 70-158; SL 0.95-1.08

#### Comments.

*Melophorus graciliceps* has a very wide distribution and has been recorded from all mainland Australian states except the ACT. This ant is distinguishable from all others except *M. lissotriches* by virtue of the combination of silvery appressed pubescence and modified, erect setae. The species differs from *M. lissotriches* in terms of the appearance of the frontal carinae (major worker) which are concave in this ant and weakly convex in *M. lissotriches*, and the appearance of the mesonotal groove and the position of the compound eyes (minor worker). Molecular sequencing has been only partially successful, but a three-gene tree indicates a close relationship between *M. graciliceps* and *M. biroi*. On the basis of morphology, however, we predict that more inclusive sampling will reveal the former will still fall within the *M. mjobergi* clade.

In general, this species prefers drier areas that support mallee or savanna vegetation, and appears to favour dunes. However, there is one record from a paddock (Coombah, NSW). One interesting note refers to the nest of this ant as being (*verbatim*) ‘strange nest, deep, narrow cone, smooth, a perfect insect trap.’ However, the principal author of this paper found a nest that was unremarkable, and it seems more likely the author of the label (B. B. Lowery) may have somehow confused the entrance burrow of an *Aphaenogaster* species (known to make that sort of nest) with the domicile of this *Melophorus*. Six workers collected at Yathong, NSW, were also taken in a malaise trap, indicating this ant may climb on vegetation if the need arises.

#### Etymology.

Latin *gracilis* (‘slender’) plus -*ceps* (‘-headed’ [from *caput*]); adjective in the nominative singular.

**Figure 36.**
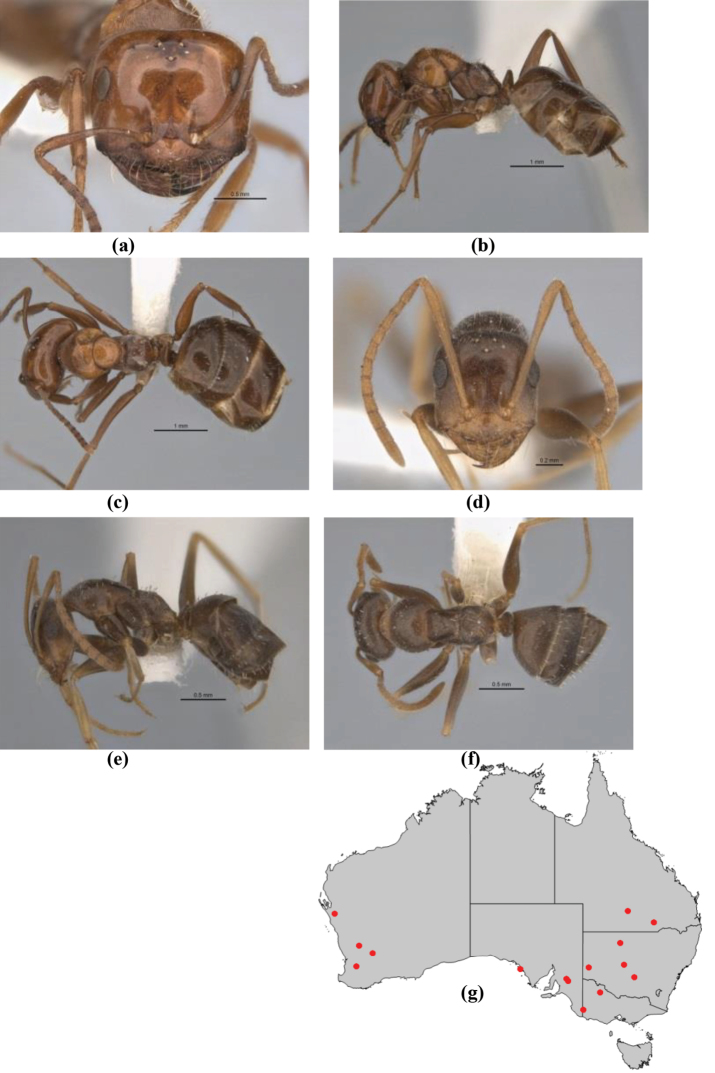
*Melophorus graciliceps* sp. n.: major worker paratype (ANIC32-900175–top ant) frons (**a**), profile (**b**) and dorsum (**c**); minor worker holotype (ANIC32-900175–bottom ant) frons (**d**), profile (**e**) and dorsum (**f**); distribution map for the species (**g**). Low resolution scale bars: 1 mm (**b, c**); 0.5 mm (**a, e**, **f**); 0.2 mm (**d**).

### Melophorus gracilis

Taxon classificationAnimaliaHymenopteraFormicidae

Hetrick, Castalanelli & Shattuck sp. n.

http://zoobank.org/22E6E2F7-6599-4314-91BC-0C0DC694A4BC

#### Types.

Holotype minor worker (middle ant) from Nita Downs turnoff, 19°04.712"S, 121°39.178"E, Western Australia, 14 February 2013 (9.45 am), B.E. Heterick, sample 74: beside road leading to station gates, ABRS M256, [ANIC 32-900209] (WAM). Paratypes: major and minor worker on same pin and with same data as holotype (WAM); minor worker with carrion (second point) from 5 km N Pardoo Hill, 19°52.408"S, 120°35.168"E, Western Australia, 14 February, 2013 (12.25pm), B.E. Heterick, sample 80: beside road: carrying carrion: aspirate, ABRS M265, [ANIC32-900212] (ANIC); 3 minor workers from 42 mi N of Mundiwindi, Western Australia, 26 April 1963, McInnes & Dowse, 29782, A395, [ANIC32-900213] (MCZ).

#### Other material examined.

**Western Australia**: 4 km N Cape Leveque turnoff (Heterick, B.E. [M251]), 42 mi N Mundiwindi (McInnes & Dowse [ANIC32-900213]), 5 km N Pardoo Hill (Heterick, B.E. [ANIC32-900212]), Nita Downs turnoff (Heterick, B.E. [ANIC32-900209]).

#### Diagnosis.

*Melophorus gracilis* can be placed in the *M. biroi* species-group on the basis of characters of the clypeus, propodeum, mandible and palps. The species is placed in the *M. biroi* species-complex on the basis of a further suite of characters (viz, metatibia of major worker with only one preapical spur [except rarely in the *mjobergi* clade]; clypeal psammophore placed anteriorly at or just above anterior margin of clypeus in the minor worker and often in the major worker; head dorsoventrally compressed to varying degrees in the minor worker of most species with the eyes placed high on the sides; compact legs, and small body size [excluding *mjobergi* clade] HW of smallest minor 0.36 mm, average HW of smallest minors 0.46 mm; HW of largest known major 1.29 mm, average HW of largest majors [where known] 1.05 mm). *Melophorus gracilis* resembles a very small, glossy *M. biroi* (major worker HW ≤ 0.65 mm, minor worker HW ≤ 0.40 mm). The minor worker of the ant can be distinguished from *M. biroi* by the possession of a pair or several long, erect setae at the midpoint of the pronotum (the *M. biroi* minor worker nearly always has a glabrous mesosoma with only a tiny pronotal seta or two in very rare individuals) and erect setae on the first gastral tergite including a line of erect marginal setae. The lack of mesosomal setae also distinguishes tiny minor workers of *M. ludius* from minor workers of this ant. The major worker can be distinguished from the major worker of *M. biroi* by the non-protuberant or arched anterior clypeal margin, and from the *M. ludius* major worker by the restriction of erect mesosomal setae to a pair on the pronotum (more setae and these widely dispersed in the *M. ludius* major worker).

#### Minor worker description.

***Head.*** Head approximately oval, sides of head convergent towards mandibular insertions; posterior margin of head strongly convex; frons shining and smooth except for piliferous pits; pilosity of frons consisting almost exclusively of well-spaced, appressed setae only (small, erect setae confined to ocular triangle). Eye moderate (eye length 0.20–0.49 length of side of head capsule); in full-face view, eyes set above midpoint of head capsule; in profile, eye set around midline of head capsule; eye elliptical. In full-face view, frontal carinae straight; frontal lobes straight in front of antennal insertion. Anterior clypeal margin broadly and evenly convex; clypeal psammophore set at or just above anterior clypeal margin; palp formula 6,4.Five mandibular teeth; mandible triangular, weakly incurved; third mandibular tooth distinctly shorter than apical tooth and teeth nos 2 and 4; masticatory margin of mandibles approximately vertical or weakly oblique. **Mesosoma.** Integument of pronotum, mesonotum and mesopleuron shining and mainly smooth throughout; anterior mesosoma smoothly rounded anteriad and flattened posteriad; mesonotum gently convex; a pair to several pronotal setae about as long as eye and unmodified; in profile, metanotal groove shallow, indicated mainly by an angle; integument of propodeum smooth and shining throughout; propodeum rounded or with indistinct angle; propodeal dorsum and declivity confluent; erect propodeal setae sparse (a couple or several); appressed propodeal setae short, separated by more than own length and inconspicuous; propodeal spiracle situated on or beside declivitous face of propodeum, and longer (length ³ ½ × height of propodeum). **Petiole.** In profile, petiolar node squamiform; in full-face view, petiolar node rounded; node shining and smooth throughout. **Gaster.** Gaster smooth and glossy; pilosity of first gastral segment consisting of a few well-spaced erect and semi-erect setae and longish, appressed setae that are separated by about their own length. **General characters.** Honey-coloured, gaster light brown to brown.

#### Major worker description.

**Head.** Head square; posterior margin of head planar or weakly convex; frons shining and smooth except for piliferous pits; pilosity of frons consisting almost exclusively of well-spaced, appressed setae only (small, erect setae confined to ocular triangle). Eye moderate (eye length 0.20–0.49 length of side of head capsule); in full-face view, eyes set at about midpoint of head capsule; in profile, eye set around midline of head capsule; eye elliptical, slightly ovoid posteriad. In full-face view, frontal carinae straight, divergent posteriad; frontal lobes curved towards antennal insertion. Anterior clypeal margin straight; clypeal psammophore set at or just above anterior clypeal margin; palp formula 6,4.Five mandibular teeth; mandible triangular, weakly incurved; third mandibular tooth distinctly shorter than apical tooth and teeth nos 2 and 4; masticatory margin of mandibles strongly oblique. **Mesosoma.** Integument of pronotum, mesonotum and mesopleuron shining and mainly smooth, superficial microreticulation most noticeable on humeri and mesopleuron; anterior mesosoma smoothly rounded anteriad and more flattened posteriad; mesonotum gently convex; pronotum with a pair of setae shorter than eye and unmodified; in profile, metanotal groove shallow, indicated mainly by an angle; integument of propodeum shining with multiple hair-like striolae on metapleuron; propodeum angulate, propodeal angle blunt; length ratio of propodeal dorsum to its declivity about 1:1; erect propodeal setae few in number and minute; appressed propodeal setae short, separated by more than own length and inconspicuous; propodeal spiracle situated on or beside declivitous face of propodeum, and longer (length ³ ½ × height of propodeum). **Petiole.** In profile, petiolar node squamiform; in full-face view, petiolar node rounded; node shining and smooth throughout. **Gaster.** Gaster smooth and glossy with faint transverse striolae visible in some lights; pilosity of first gastral segment consisting of a few well-spaced erect and semi-erect setae and appressed or decumbent setae that are separated by more than their own length. **General characters.** Honey-coloured, gaster light brown to brown.

#### Measurements.

Worker (n = 7): CI 92–98; EI 25–31; EL 0.12–0.16; HL 0.42–0.64; HW 0.38–0.61; ML 0.56–0.78; MTL 0.32–0.44; PpH 0.07–0.10; PpL 0.23–0.33; SI 87–110; SL 0.42–0.54.

#### Comments.

This very small, shining ant is confined to the far north of Australia. In WA, specimens have been taken from the Cape Leveque turnoff, Great Northern Hwy, near Broome, N of Pardoo Hill and from the Nita Downs turnoff, Great Northern Hwy. Ants in the TERC Collection believed to be of the same species have been taken from locations in West Rankin Stn, Connell’s Lagoon and Alexandra Stn., all in the NT. *Melophorus gracilis* resembles a small, light brown *M. biroi* but differs in having erect setae present on the pronotum and first gastral tergite. The species can be differentiated from other, similar species by a combination of its small eye, visibly thin cuticle and lack of sculpture on the mesopleuron. In a five-gene tree (Figure 4), *M. gracilis* comes out as sister to *M. biroi*. This species nests directly into sandy soils and workers at one nest were seen carrying small carrion (dead workers of *Monomorium rothsteini*). In all likelihood they are general scavengers of plant and dead animal material.

#### Etymology.

Latin *gracilis* (‘slender’); adjective in the nominative singular.

**Figure 37.**
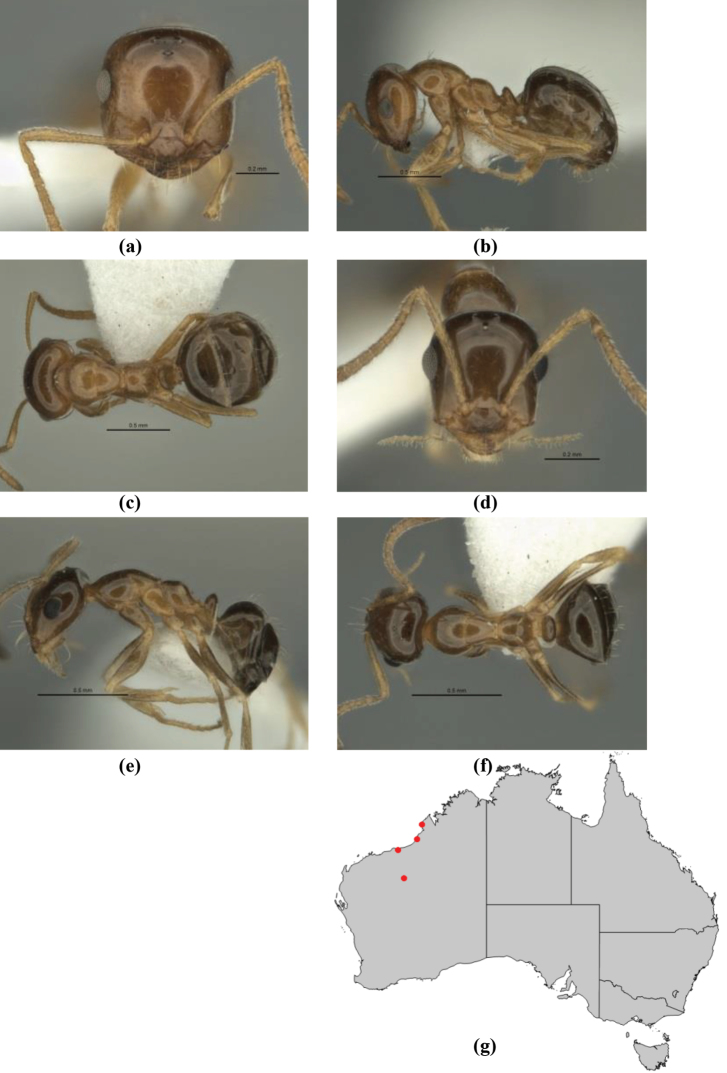
*Melophorus gracilis* sp. n.: major worker paratype (ANIC32-900209–bottom ant) frons (**a**), profile (**b**) and dorsum (**c**); minor worker holotype (ANIC32-900209–middle ant) frons (**d**), profile (**e**) and dorsum (**f**); distribution map for the species (**g**). Low resolution scale bars: 0.5 mm (**b, c**, **e, f**); 0.5 mm (**a, d**).

### Melophorus latinotus

Taxon classificationAnimaliaHymenopteraFormicidae

Heterick, Castalanelli & Shattuck sp. n.

http://zoobank.org/F7E1F489-0886-4D23-AB32-2871C55E5E51

#### Types.

Minor worker from Eneabba region 29°24'27"S, 115°06'49"E, Western Australia, 24-28 November 2006, A. Gove/N. McCoy, *Melaleuca leuropoma* sandplain slope: pitfall trap 2H [JDM32-002004] (WAM). Paratypes: 2 minor workers from Eneabba region 29°41'45"S, 115°11'18"E, Western Australia, 24-28 November 2006, A. Gove/N. McCoy, *Conospermum triplinerv*/ *Eremaea beaufortioides*, sandplain flat: pitfall trap 23N [JDM32-002003] (WAM); 2 minor workers with same collection data as preceding paratypes [ANIC] .

#### Other material examined.

**Western Australia**: 9 kn N Yanchep (Leicester, K./McDavitt, S.).

#### Diagnosis.

*Melophorus latinotus* can be placed in the *M. biroi* species-group on the basis of characters of the clypeus, propodeum, mandible and palps. The species is placed in the *M. biroi* species-complex on the basis of a further suite of characters (viz, metatibia of major worker with only one preapical spur [except rarely in the *mjobergi* clade]; clypeal psammophore placed anteriorly at or just above anterior margin of clypeus in the minor worker and often in the major worker; head dorsoventrally compressed to varying degrees in the minor worker of most species with the eyes placed high on the sides; compact legs, and small body size [excluding *mjobergi* clade] HW of smallest minor 0.36 mm, average HW of smallest minors 0.46 mm; HW of largest known major 1.29 mm, average HW of largest majors [where known] 1.05 mm). Uniform, minute, net-like microreticulation and a transverse row of short, stout unmodified setae placed across the centre of pronotum distinguish minor workers of this rare ant from similar forms in the *M. biroi* species-complex. The major worker is unknown.

#### Minor worker description.

**Head.** Head square; posterior margin of head planar or weakly convex; frons matt or with weak sheen, microreticulate or microreticulate-shagreenate; pilosity of frons consisting exclusively or almost exclusively of well-spaced, appressed setae only (small, erect setae, if present, usually confined to ocular triangle or posterior margin of head). Eye moderate (eye length 0.20–0.49 length of side of head capsule); in full-face view, eyes set above midpoint of head capsule; in profile, eye set around midline of head capsule; eyes elliptical or slightly reniform. In full-face view, frontal carinae distinctly concave; frontal lobes straight in front of antennal insertion. Anteromedial clypeal margin broadly and evenly convex; clypeal psammophore set below midpoint of clypeus; palp formula 6,4. Five mandibular teeth in minor worker; mandibles triangular, weakly incurved; third mandibular tooth distinctly shorter than apical tooth and teeth numbers two and four; masticatory margin of mandibles approximately vertical or weakly oblique. **Mesosoma.** Integument of pronotum, mesonotum and mesopleuron matt or with weak sheen and microreticulate throughout; anterior mesosoma in profile rounded anteriad, thereafter pronotum and whole of mesonotum flattened and on a higher plane than propodeum; appearance of erect pronotal setae short, (i.e., longest erect setae shorter than length of eye) and unmodified; in profile, metanotal groove a narrow but deep slit; propodeum matt or with a weak sheen and microreticulate; propodeum angulate, propodeal angle blunt; length ratio of propodeal dorsum to its declivity between 1:1 and 1:2; erect propodeal setae always absent; appressed propodeal setulae short, separated by more than own length and inconspicuous; propodeal spiracle situated on or beside declivitous face of propodeum, and longer (length ≥ 0.50 × height of propodeum). **Petiole.** In profile, petiolar node squamiform; in full-face view, shape of petiolar node uniformly rounded; node matt with indistinct microsculpture. **Gaster.** Gaster matt with faint, indistinct microsculpture; pilosity of first gastral tergite consisting of well-spaced, long, whitish, appressed setae with erect setae (present in at least some workers) confined to margin of the sclerite. **General characters.** Colour dull brown.

#### Measurements.

Worker (n = 2): CI 101–103; EI 26–26; EL 0.15–0.17; HL 0.54–0.59; HW 0.56–0.59; ML 0.75–0.78; MTL 0.41–0.45; PpH 0.09–0.08; PpL 0.34–0.35; SI 108–114; SL 0.60–0.68.

#### Comments.

There seems little doubt that this uncommon ant has a very localized distribution on the Swan Coastal Plain in WA from about Perth to south of Geraldton. Only the minor worker is known. Just two collections have been recorded; one from Eneabba and one from Yanchep National Park. The species resembles *M. biroi* but has a few, short, erect setae on the pronotum, and is very dull and matt in appearance with the cuticular pattern on the mesosoma a fine, reticulate mesh. No specimens were available for sequencing and there are no ecological data.

#### Etymology.

Latin *latus* (‘broad’) plus Latinized Greek *notus* (Greek *notos* ‘back’); adjective in the nominative singular.

**Figure 38.**
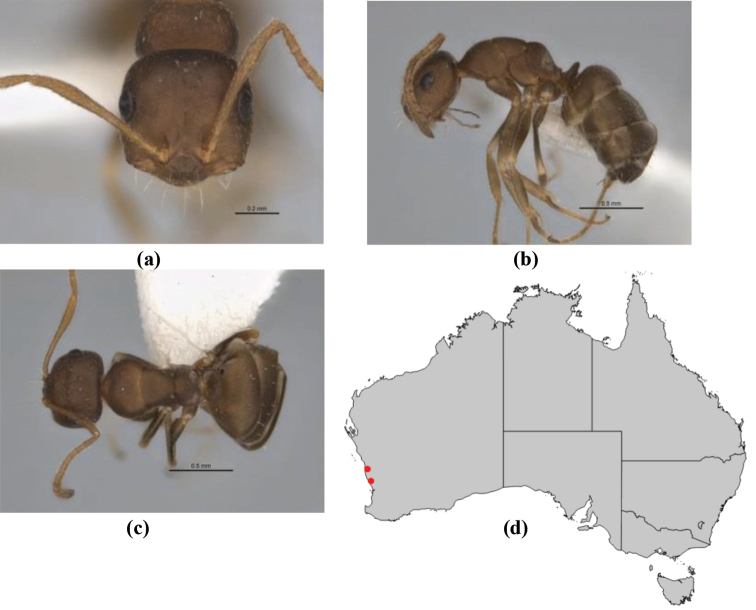
*Melophorus latinotus* sp. n.: minor worker holotype (JDM32-002004) frons (**a**), profile (**b**) and dorsum (**c**); distribution map for the species (**d**). Low resolution scale bars: 0.5 mm (**b, c**); 0.2 mm (**a**).

### Melophorus lissotriches

Taxon classificationAnimaliaHymenopteraFormicidae

Heterick, Castalanelli & Shattuck sp. n.

http://zoobank.org/71415F18-A8CC-4DB3-B486-FCF8016F7E9D

#### Types.

Holotype minor worker (bottom ant) from St George (‘Saint George’-*sic*), Queensland, 23 January 1966, B.B. Lowery, sav. Woodland, ANIC Ants Vial 20.214 [ANIC32-900148] (ANIC). Paratypes: Major and minor worker on same pin and with same details as holotype (ANIC); major and minor worker from 10 km E of Mt Ive HS, Gawler Ranges, South Australia, 2 October 1980, P.J.M. Greenslade, A2 SA (BMNH); 3 minor workers from 12 km E of Emu, Victoria Desert, South Australia, 5-10 October 1976, P.J.M. Greenslade, *Casuarina* (MCZ); 2 minor workers from Koonamore, South Australia, 24-27 February 1973, P.J.M. Greenslade, pitfall traps, 2) [ANIC32-900131] (MCZ); minor worker from Chandlers Breakaway, Western Australia, 16 September 1988, B. Heterick, soil, native vegetation, rural environment, 452, 8*Mel*BH33 (SAM); 2 major workers and a minor worker from Black Swan Mine 30°28'S, 121°43'E, Western Australia, 11 December 2003-5 January 2004, P. Langlands/J. Osborne; Site data C2:5 [JDM32-004807] (WAM).

#### Other material examined.

**New South Wales**: 40 km NNW Louth, Lake Mere (Greenslade, P.J.M.), Bogan River (Armstrong, J.), Callubri Station, 2 mi from homestead (Greaves, T.), CSIRO Lake Mere Field Station, near Louth (Bryannah, M.). **Northern Territory**: 25 km N Alice Springs (Shattuck, S.O.), Kunoth Paddock, near Alice Springs (Greenslade, P.J.M.), Manbulloo, SW Katherine (Greenslade, P.J.M.), Manbulloo, SW Katherine (Greenslade, P.J.M.), nr Limbunya turnoff (Heterick, B.E. [M226]), Tanami (Greenslade, P.J.M.). **Queensland**: ‘Gumbardo’ (Beutel, T.), ‘Merigol’ (Beutel, T.), 75 km E Cunnamulla (Greenslade, P.J.M.), St. George (Lowery, B.B.), St. George (Lowery, B.B.), St. George (Lowery, B.B.). **South Australia**: 10 km E Mt Ive Homestead, Gawler Ranges (Greenslade, P.J.M.), 50 km E Vokes Hill, Victoria Desert (Greenslade, P.J.M.), Observatory Hill, Victoria Desert (Greenslade, P.J.M.). **Western Australia**: Mulga, NE Goldfields (Pringle, H.J.R. [ANIC32-029567]).

#### Diagnosis.

*Melophorus lissotriches* can be placed in the *M. biroi* species-group on the basis of characters of the clypeus, propodeum, mandible and palps. The species is also placed in the *M. mjobergi* clade but differs from the most derived species in that the major and media workers do not have the deeply recessed area around the frontal carinae and medial sector of clypeus or the impressed setae-bearing sockets visible in the heads of major and media workers of *M. mjobergi*, *M. postlei* and *M. compactus*.Minor and major workers of *M. lissotriches* are clothed with fine, appressed silvery setae that form pubescence in the minor worker, at least, in conjunction with multiple scattered, modified erect setae (the modified setae varying from distally slightly flattened to clavate) on the head, mesosoma and gaster. This species can only be confused with its near relation, *M. graciliceps*, but differs in having, in full-face view, the eye of the minor worker moderately convex and bulging well beyond the outline of head capsule. In profile, the mesosoma of the *M. lissotriches* minor worker is sinuous, the mesonotum dipping towards its junction with the propodeum and forming a weak v-shaped notch. The frontal carinae of major worker is straight or weakly convex (concave in the *M. graciliceps* major worker), and the cuticle of the major worker head is matt or weakly shining and minutely pitted.

#### Minor worker description.

**Head.** Head rectangular; posterior margin of head strongly convex; frons shining with indistinct microsculpture that is most pronounced on lower surfaces; frons consisting of appressed pubescence, with many short, unmodified, erect setae. Eye moderate (eye length 0.20–0.49 length of side of head capsule); in full-face view, eyes set above midpoint of head capsule; in profile, eye set around midline of head capsule; eyes elliptical or slightly reniform. In full-face view, frontal carinae concave; frontal lobes straight in front of antennal insertion. Anteromedial clypeal margin broadly and evenly convex; clypeal psammophore set at or just above anterior clypeal margin; palp formula 6,4. Five mandibular teeth in minor worker; mandibles triangular, weakly incurved; third mandibular tooth distinctly shorter than apical tooth and teeth numbers two and four; masticatory margin of mandibles approximately vertical or weakly oblique. **Mesosoma.** Integument of pronotum, mesonotum and mesopleuron moderately shining and shagreenate throughout; anterior mesosoma in profile broadly convex; appearance of erect pronotal setae short and often expanded distally, at times clavate; in profile, metanotal groove shallow, broadly V or U-shaped; propodeum shining and shagreenate; propodeum angulate, propodeal angle blunt; length ratio of propodeal dorsum to its declivity between 1:1 and 1:2; erect propodeal setae present and sparse to moderate (1-12); appressed propodeal setulae long and closely aligned, creating pubescence; propodeal spiracle situated on or beside declivitous face of propodeum, and longer (length ≥ 0.50 × height of propodeum). **Petiole.** In profile, petiolar node rectangular, vertex blunt, directed posteriad; in full-face view, shape of petiolar node uniformly rounded; node shining and distinctly shagreenate-microreticulate. **Gaster.** Gaster weakly shining with indistinct shagreenation; pilosity of first gastral tergite consisting of thick, appressed setae that form pubescence, interspersed with numerous short, bristly, erect setae. **General characters.** Colour honey-brown.

#### Major worker description.

**Head.** Head quadrate (i.e., heart-shaped); posterior margin of head weakly concave; cuticle of frons matt or with weak sheen, indistinctly shagreenate; pilosity of frons a mixture of many short, erect, bristly setae interspersed with regularly spaced appressed setae. Eye small (eye length less than 0.2 × length of head capsule); in full-face view, eyes set above midpoint of head capsule; in profile, eye set anteriad of midline of head capsule; eyes elliptical. In full-face view, frontal carinae weakly convex; frontal lobes curved toward antennal insertion. Anterior clypeal margin broadly convex with anteromedial dimple; clypeal psammophore set at or above midpoint of clypeus; palp formula 6,4. Five mandibular teeth in major worker; mandibles triangular, weakly incurved; third mandibular tooth distinctly shorter than apical tooth and teeth numbers two and four; masticatory margin of mandibles approximately aligned vertically or weakly oblique. **Mesosoma.** Integument of pronotum, mesonotum and mesopleuron shining with indistinct microsculpture that is most pronounced on lower surfaces; anterior mesosoma in profile pronotum smoothly rounded anteriad and flattened posteriad, mesonotum narrowly convex; erect pronotal setae short and often expanded distally, at times clavate; in profile, metanotal groove shallow, broadly V- or U-shaped; propodeum shining and shagreenate; propodeum always smoothly rounded; propodeal dorsum and declivity confluent; erect propodeal setae present and abundant (at least a dozen); appressed propodeal setae long and closely aligned, creating pubescence; propodeal spiracle situated on or beside declivitous face of propodeum, and longer (length ≥ 0.50 × height of propodeum). In profile, petiolar node squamiform; in full-face view, shape of petiolar node uniformly rounded, or generally rounded with median indentation; node shining and faintly shagreenate-microreticulate. **Petiole.** In profile, petiolar node squamiform; in full-face view, petiolar node generally rounded with median indentation or hollow; appearance of node shining and smooth with vestigial microreticulation. **Gaster.** Gaster weakly shining with indistinct shagreenation; pilosity of first gastral tergite consisting of short, bristly, erect setae over well-spaced, short, appressed setae. **General characters.** Colour of foreparts orange, gaster tawny-brown with more orange towards the sclerite margins.

#### Measurements.

Worker (n = 6): CI 89–105; EI 16–29; EL 0.17–0.26; HL 0.68–1.51; HW 0.60–1.58; ML 1.01–1.84; MTL 0.92–1.11; PpH 0.10–0.14; PpL 0.44–0.78; SI 62–173; SL 0.98–1.04.

#### Comments.

*Melophorus lissotriches* closely resembles *M. graciliceps* but can be recognized by the different profile and more bulbous eyes (minor worker) and the matt or weakly shining head with its straight or weakly convex frontal carinae (major worker). *Melophorus lissotriches* occurs widely in Australia and occupies the same sort of arid and semi-arid habitat as *M. graciliceps*, but has a generally more northerly distribution and does not appear to occur in Victoria. Restricted sequencing (three genes) confirms its position in the *M. mjobergi* clade and the mtDNA sequence places it close to *M. graciliceps*.

This species has been taken on several different types of soils (notably, red soil and brown soil plain) and vegetation zones (box-pine scrub, savanna woodland and *Callitris* woodland are mentioned on labels). The species has also been collected from a paddock. The ant is active at very high temperatures: five workers were collected by B. B. Lowery at 3.30pm when the temperature was 104 degrees Fahrenheit (40 degrees Celsius). He remarked that these foragers moved very fast and resembled pale *Iridomyrmex*.

#### Etymology.

Greek *lissos* (‘smooth’, ‘polished’) plus pl. of *trichos* (‘hair’); noun in the nominative plural standing in apposition to the generic name.

**Figure 39.**
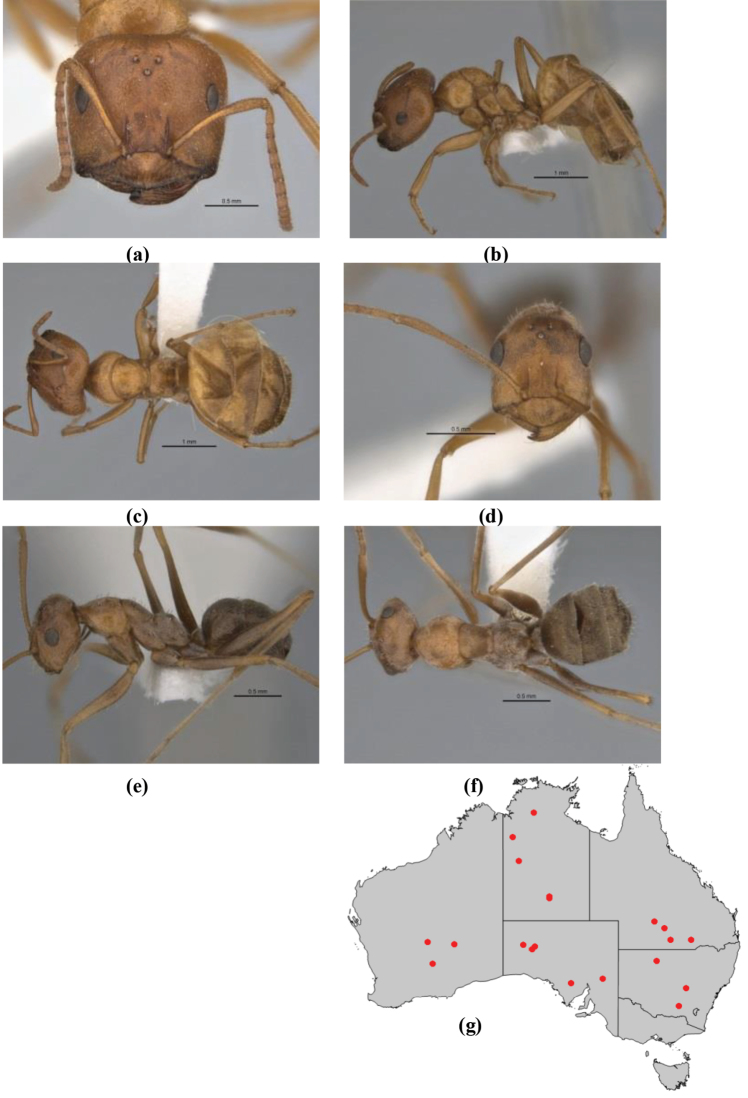
*Melophorus lissotriches* sp. n.: major worker paratype (ANIC32-900148–top ant) frons (**a**), profile (**b**) and dorsum (**c**); minor worker holotype (ANIC32-900148–bottom ant) frons (**d**), profile (**e**) and dorsum (**f**); distribution map for the species (**g**). Low resolution scale bars: 1 mm (**b, c**); 0.5 mm (**a, d–f**).

### Melophorus longiceps

Taxon classificationAnimaliaHymenopteraFormicidae

Heterick, Castalanelli & Shattuck sp. n.

http://zoobank.org/9F45E0BF-07F1-4804-B90D-1389936F295B

#### Types.

Holotype minor worker from CSIRO Lake Mere field stn near Louth, New South Wales, paddock B, January 1995, M. Bryannah, paddock D (EXC) Interc[ept], *Melophorus* sp. K [ANIC32-900104] (ANIC). Paratypes: 2 minor workers from CSIRO Lake Mere field stn near Louth, New South Wales, paddock B, January 1995, M. Bryannah, paddock E Runoff, ‘*Melophorus* sp. K’ [ANIC32-066665] (BMNH); major worker from CSIRO Lake Mere field stn near Louth, New South Wales, paddock B, January 1995, M. Bryannah, paddock G Runoff, ‘*Melophorus* sp. K’ [ANIC32-066665] (MCZ).

#### Diagnosis.

*Melophorus longiceps* can be placed in the *M. biroi* species-group on the basis of characters of the clypeus, propodeum, mandible and palps. The species is placed in the *M. biroi* species-complex on the basis of a further suite of characters (viz, metatibia of major worker with only one preapical spur [except rarely in the *mjobergi* clade]; clypeal psammophore placed anteriorly at or just above anterior margin of clypeus in the minor worker and often in the major worker; head dorsoventrally compressed to varying degrees in the minor worker of most species with the eyes placed high on the sides; compact legs, and small body size [excluding *mjobergi* clade] HW of smallest minor 0.36 mm, average HW of smallest minors 0.46 mm; HW of largest known major 1.29 mm, average HW of largest majors [where known] 1.05 mm). In profile, the head of the minor worker is distinctly dorsoventrally flattened and, in full-face view, extremely narrow (CI ≤ 75). This extreme narrowness is a unique character that defines this species. Some *M. biroi* have rather narrow heads, but in those cases the CI is > 85. The major worker is unknown.

#### Minor worker description.

**Head.** Head rectangular; posterior margin of head planar or weakly concave; frons matt or with weak sheen, shagreenate; frons consisting exclusively or almost exclusively of well-spaced, appressed setae only (small, erect setae, if present, usually confined to ocular triangle or posterior margin of head). Eye moderate (eye length 0.20–0.49 length of side of head capsule); in full-face view, eyes set above midpoint of head capsule; in profile, eye set anteriad of midline of head capsule; eyes elliptical or slightly reniform. In full-face view, frontal carinae distinctly concave; frontal lobes straight in front of antennal insertion. Anteromedial clypeal margin broadly and evenly convex; clypeal psammophore set at or just above anterior clypeal margin; palp formula 6,4. Five mandibular teeth in minor worker; mandibles triangular, weakly incurved; third mandibular tooth distinctly shorter than apical tooth and teeth numbers two and four; masticatory margin of mandibles approximately vertical or weakly oblique. **Mesosoma.** Integument of pronotum, mesonotum and mesopleuron with weak to moderate sheen, shagreenate on pronotum and dorsum of mesonotum, otherwise microreticulate; anterior mesosoma in profile weakly elevated anteriad, thereafter gently sinuate, pronotum and mesonotum on same plane; erect pronotal setae absent; in profile, metanotal groove shallow, indicated mainly by an angle; propodeum matt or with a weak sheen and microreticulate; propodeum angulate, propodeal angle blunt, or distinctly angulate, propodeal angle sharp; length ratio of propodeal dorsum to its declivity about 1:1; erect propodeal setae always absent; appressed propodeal setulae short, separated by more than own length and inconspicuous; propodeal spiracle situated on or beside declivitous face of propodeum, and shorter (length < 0.50 × height of propodeum). **Petiole.** In profile, petiolar node squamiform; in full-face view, shape of petiolar node uniformly rounded; node shining and distinctly microreticulate. **Gaster.** Gaster shining, shagreenate (‘LP record’ appearance); pilosity of first gastral tergite consisting of well-spaced short, inconspicuous, appressed setae only, erect setae always absent. **General characters.** Colour uniformly chocolate with some tan highlights.

#### Measurements.

Worker (n = 2): CI 76–81; EI 30–38; EL 0.15–0.16; HL 0.56–0.63; HW 0.43–0.51; ML 0.72–0.82; MTL 0.41–0.43; PpH 0.07–0.10; PpL 0.29–0.34; SI 111–121; SL 0.52–0.56.

#### Comments.

Currently, *Melophorus longiceps*, known only from the minor worker, has been recorded from the Lake Mere Field Station, near Louth, NSW. However, workers morphologically similar to this species were seen in the TERC Collection but time did not permit a detailed examination. This material came from WA (Ningbing Ra) and NT (Kalkarindji, Humbert River and Pigeon Hole Stn) and included a major worker. The different biogeography, however, precludes a too ready association of the two sets of taxa. The elongate, flattened head of this species separates it from the rest of the genus, but in other respects it is identical to *M. biroi* (in which the head capsule varies in length). A worker was collected from a paddock, but there are no other ecological data, and no specimens were available for sequencing.

#### Etymology.

Latin *longus* (‘long’) plus -*ceps* (‘-headed’ [from *caput*]); adjective in the nominative singular.

**Figure 40.**
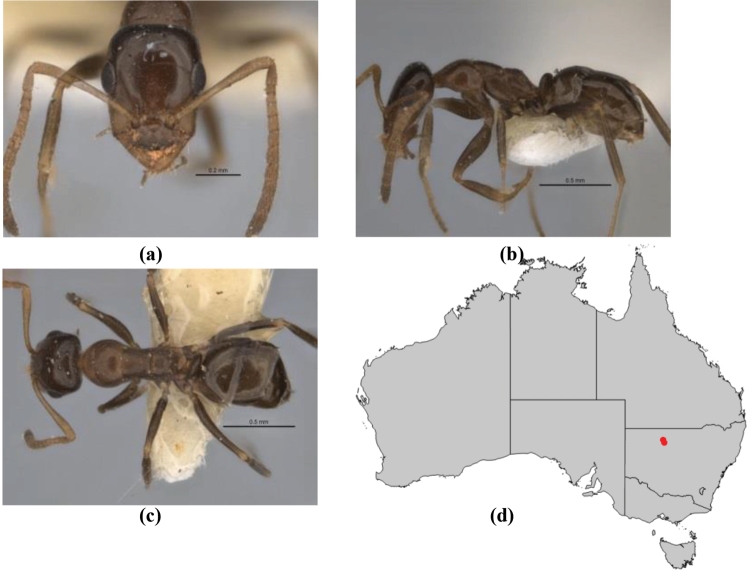
*Melophorus longiceps* sp. n.: minor worker holotype (ANIC32-900104) frons (**a**), profile (**b**) and dorsum (**c**); distribution map for the species (**d**). Low resolution scale bars: 0.5 mm (**b, c**); 0.2 mm (**a**).

### Melophorus macrops

Taxon classificationAnimaliaHymenopteraFormicidae

Heterick, Castalanelli & Shattuck sp. n.

http://zoobank.org/99133D0F-9A9B-490E-BFE6-32CB9F629325

#### Types.

Holotype minor worker from Lake Mere field stn, New South Wales (label now missing, possibly mislaid during photographing), ‘*Melophorus* sp. J’ [ANIC32-900105] (ANIC). Paratypes: 3 minor workers from Wilpena Pound, South Australia, 18 June 1972, B.B. Lowery (ANIC); minor worker from Cunnamulla, Queensland, 17 September 1974, P.J.M. Greenslade, (8), 16), [ANIC32-900101] (MCZ).

#### Diagnosis.

*Melophorus macrops* can be placed in the *M. biroi* species-group on the basis of characters of the clypeus, propodeum, mandible and palps. The species is placed in the *M. biroi* species-complex on the basis of a further suite of characters (viz, metatibia of major worker with only one preapical spur [except rarely in the *mjobergi* clade]; clypeal psammophore placed anteriorly at or just above anterior margin of clypeus in the minor worker and often in the major worker; head dorsoventrally compressed to varying degrees in the minor worker of most species with the eyes placed high on the sides; compact legs, and small body size [excluding *mjobergi* clade]; HW of smallest minor 0.36 mm, average HW of smallest minors 0.46 mm; HW of largest known major 1.29 mm, average HW of largest majors [where known] 1.05 mm). The minor worker of *M. macrops* is defined at a species level by its large eye (EI 40-41), the presence of at least several erect setae on the mesosoma including propodeal setae, the deeply impressed metanotal groove (when viewed in profile) and the rounded propodeum. The first gastral tergite has erect setae, including a line of marginal setae. The major worker is unknown.

#### Minor worker description.

**Head.** Head approximately oval with straight sides; posterior margin of head weakly convex; frons shining and smooth except for piliferous pits; pilosity of frons a mixture of short, erect and semi-erect setae interspersed with shorter decumbent setae and well-spaced, short, appressed setae. Eye large (eye length ≥ 0.50 × length of side of head capsule), or moderate (eye length 0.20–0.49 length of side of head capsule); in full-face view, eyes set at about midpoint of head capsule; in profile, eye set anteriad of midline of head capsule; eyes elliptical or slightly reniform. In full-face view, frontal carinae straight, divergent posteriad; frontal lobes curved toward antennal insertion. Anteromedial clypeal margin broadly and evenly convex and protrusive; clypeal psammophore set at or just above anterior clypeal margin; palp formula 6,4. Five mandibular teeth in minor worker; mandibles triangular, weakly incurved; third mandibular tooth distinctly shorter than apical tooth and teeth numbers two and four; masticatory margin of mandibles approximately vertical or weakly oblique. **Mesosoma.** Integument of pronotum, mesonotum and mesopleuron shining and smooth on dorsum, entire lower mesopleuron distinctly striolate-microreticulate; anterior mesosoma in profile weakly elevated anteriad, thereafter gently sinuate, pronotum and mesonotum on same plane; appearance of erect pronotal setae short, (i.e., longest erect setae shorter than length of eye) and unmodified; in profile, metanotal groove shallow, broadly V or U-shaped; propodeum shining and smooth on dorsum and declivitous face, metapleuron with striolate-microreticulate sculpture; propodeum smoothly rounded or with indistinct angle; propodeal dorsum and declivity confluent; erect propodeal setae present and sparse to moderate (1-12); appressed propodeal setulae long and separated by at least own length; propodeal spiracle situated on or beside declivitous face of propodeum, and shorter (length < 0.50 × height of propodeum). **Petiole.** In profile, petiolar node squamiform; in full-face view, shape of petiolar node uniformly rounded; node shining and smooth throughout. **Gaster.** Gaster smooth and glossy; pilosity of first gastral tergite consisting of well-spaced, erect and semi-erect setae interspersed with regularly placed appressed setae. **General characters.** Colour of foreparts uniformly tan (or head dark chocolate, mesosoma tan), gaster dark chocolate.

#### Measurements.

Worker (n = 2): CI 92–95; EI 40–41; EL 0.18–0.19; HL 0.48–0.51; HW 0.45–0.47; ML 0.61–0.68; MTL 0.32–0.33; PpH 0.08–0.08; PpL 0.24–0.25; SI 109–110; SL 0.49–0.51.

#### Comments.

*Melophorus macrops* is here described from minor workers from Lake Mere Field Station, NSW, Cunnamulla, QLD and Wilpena Pound, SA, respectively. The only other material seen is a pin of three workers (a fourth point on the pin held a minor worker of *Melophorus ludius*–now removed) from Wilpena Pound, SA. This pin was inadvertently included among specimens of *Melophorus sericothrix*, so was not included in data collection. In appearance this ant is morphologically similar to *M. biroi*, but has at least a dozen erect setae on the mesosoma, and the eye is rather large (EI 40-41). The ant can also be distinguished from the smaller-eyed *M. gracilis* by the scalloped mesopleuron. The Lake Mere specimen was collected from a paddock. Apart from this, nothing more is known about *M. macrops* and no specimens were available for sequencing.

#### Etymology.

Greek *makros* (‘long’) plus *ops* (‘eye’); adjective in the nominative singular.

**Figure 41.**
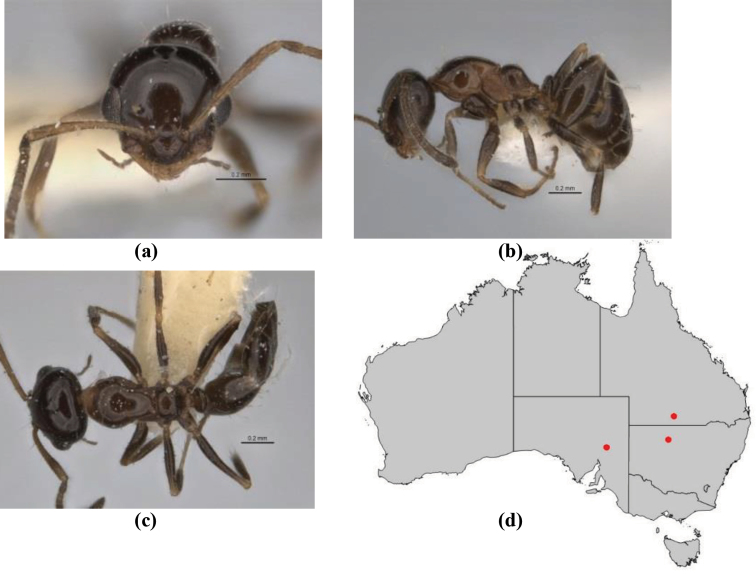
*Melophorus macrops* sp. n.: minor worker holotype (ANIC32-900105) frons (**a**), profile (**b**) and dorsum (**c**); distribution map for the species (**d**). Low resolution scale bars: 0.2 mm (**a–c**).

### Melophorus microreticulatus

Taxon classificationAnimaliaHymenopteraFormicidae

Heterick, Castalanelli & Shattuck sp. n.

http://zoobank.org/1B4715FA-B33D-4197-A484-93A6A232C6E1

#### Types.

Holotype minor worker from Emu Camp, Victoria Desert, 5 October 1976, P.J.M. Greenslade, (8), h1, [ANIC32-900172] (ANIC). Paratypes: major and media worker from Gawler Range, South Australia, 4 October 1972, P.J.M. Greenslade, (2) [ANIC32-900171] (ANIC); 3 minor workers from Fowlers Gap, New South Wales, 18 November 1979, P.J.M. Greenslade, (4) (ANIC); 2 minor workers from Gawler Ranges, South Australia, 4 October 1972, P.J.M. Greenslade, (2) A (BMNH); 2 minor workers from Brookfield Conservation Park 34°19'S, 139°29'E, South Australia, 29 October 1991, S. Shattuck, # 2525.7 [ANIC32-900098] (MCZ); 2 minor workers from Cunnamulla, Queensland, 17 September 1974, P.J.M. Greenslade, (8), gpt (QM); 2 minor workers from 5 km NE Koonamore HS, South Australia, 26 February 1973, P.J.M. Greenslade, (1) K1/lo(?) 13 (SAM); minor worker from 7 km NE Leinster, Western Australia, 16 September 1988, B. Heterick, soil, native vegetation, rural environment, 451, 8*Mel*Bh25 [JDM32-004550] (WAM).

#### Other material examined.

**New South Wales**: 40 km NNW Louth, Lake Mere (Greenslade, P.J.M.), 40 km NNW Louth, Lake Mere (Greenslade, P.J.M.), Bogan River (Armstrong, J. [ANIC32-900099]), CSIRO Lake Mere Field Station, near Louth (Bryannah, M.), Fowlers Gap (Greenslade, P.J.M.), Fowlers Gap (Greenslade, P.J.M.). **Northern Territory**: Kunoth Paddock, near Alice Springs (Greenslade, P.J.M.). **Queensland**: ‘Merigol’ (Beutel, T.), 10 km E Eulo (Greenslade, P.J.M.), Cunnamulla (Greenslade, P.J.M.), Eulo (Greenslade, P.J.M.). **South Australia**: 15 km NE Mt Bryan (Greenslade, P.J.M.), 1 km W Emu Camp, Victoria Desert (Greenslade, P.J.M.), 50 km E Emu Junction, Victoria Desert (Greenslade, P.J.M.), 50 km E Vokes Hill, Victoria Desert (Greenslade, P.J.M.), 7 km NW Morgan (Greenslade, P.J.M.), 8 km NW Morgan (Greenslade, P.J.M.), Cambrai (Greenslade, P.J.M.), Koonamore (Greenslade, P.J.M.), Koonamore (Greenslade, P.J.M.), Koonamore, 2 km NE homestead (Greenslade, P.J.M.), Koonamore (Greenslade, P.J.M.). **Western Australia**: 30 km SE Kambalda (Walliss, N. [JDM32-004551]).

#### Diagnosis.

*Melophorus microreticulatus* can be placed in the *M. biroi* species-group on the basis of characters of the clypeus, propodeum, mandible and palps. The species is placed in the *M. biroi* species-complex on the basis of a further suite of characters (viz, metatibia of major worker with only one preapical spur [except rarely in the *mjobergi* clade]; clypeal psammophore placed anteriorly at or just above anterior margin of clypeus in the minor worker and often in the major worker; head dorsoventrally compressed to varying degrees in the minor worker of most species with the eyes placed high on the sides; compact legs, and small body size [excluding *mjobergi* clade]; HW of smallest minor 0.36 mm, average HW of smallest minors 0.46 mm; HW of largest known major 1.29 mm, average HW of largest majors [where known] 1.05 mm). This species is characterised by having large eyes (EI 40 ≥). The minor worker is of small size (HW 0.40 mm ≥) and lacks erect setae on mesosoma and first gastral tergite. The minor worker can be distinguished from similar size *M. biroi*, the only species with which it may be confused, by possessing distinct microreticulate sculpture over the mesosoma (this sculpturing may be weaker on the pronotum in some specimens). The major worker has a posterior clypeal margin that is not arched or falling away between the antennal insertion and tentorial pit, and an anterior clypeal margin that is straight and not protuberant. This, and the microreticulate sculpture will serve to distinguish the *M. microreticulatus* major worker from the major worker of *M. biroi*.

#### Minor worker description.

**Head.** Head approximately oval with straight sides; posterior margin of head planar to strongly convex; frons shining with superficial shagreenation or microreticulation only, or matt or with weak sheen, microreticulate or microreticulate-shagreenate; frons consisting exclusively or almost exclusively of well-spaced, appressed setae only (small, erect setae, if present, usually confined to ocular triangle or posterior margin of head). Eye large (eye length ≥ 0.50 × length of side of head capsule); in full-face view, eyes set at about midpoint of head capsule; in profile, eye set anteriad of midline of head capsule; eyes elliptical or slightly reniform. In full-face view, frontal carinae straight or weakly convex; frontal lobes curved inward in front of antennal insertion. Anteromedial clypeal margin broadly and evenly convex and protrusive; clypeal psammophore set below midpoint of clypeus; palp formula 6,4. Five mandibular teeth in minor worker; mandibles triangular, weakly incurved; third mandibular tooth distinctly shorter than apical tooth and teeth numbers two and four; masticatory margin of mandibles approximately vertical or weakly oblique. **Mesosoma.** Integument of pronotum, mesonotum and mesopleuron matt or with weak sheen and microreticulate throughout, or pronotum smooth and shining, mesonotum shining and superficially microreticulate, mesopleuron densely microreticulate and may be almost matt; anterior mesosoma in profile smoothly rounded anteriad, thereafter pronotum and whole of mesonotum flattened and on same plane as propodeum; erect pronotal setae absent; in profile, metanotal groove shallow, broadly V or U-shaped; propodeum matt or with a weak sheen and microreticulate; propodeum angulate, propodeal angle blunt; length ratio of propodeal dorsum to its declivity between 3:2 and 4:3; erect propodeal setae always absent; appressed propodeal setulae long, each reaching setae behind and in front, but not forming pubescence; propodeal spiracle situated on or beside declivitous face of propodeum, and shorter (length < 0.50 × height of propodeum). **Petiole.** In profile, petiolar node squamiform; in full-face view, shape of petiolar node uniformly rounded; node shining and smooth throughout. **Gaster.** Gaster smooth and glossy; pilosity of first gastral tergite consisting of well-spaced short, inconspicuous, appressed setae only, erect setae always absent. **General characters.** Colour of head and mesosoma dark brown to blackish-brown, mesosoma variably brown-and-orange-tan.

#### Major worker description.

**Head.** Head square; posterior margin of head planar or weakly concave; cuticle of frons shining and smooth except for piliferous pits and a few striolae around antennal insertions and Frontal carinae; frons consisting exclusively or almost exclusively of well-spaced, appressed setae only (small, erect setae, if present, usually confined to ocular triangle or posterior margin of head). Eye moderate (eye length 0.20–0.49 length of head capsule); in full-face view, eyes set at about midpoint of head capsule; in profile, eye set anteriad of midline of head capsule; eyes elliptical. In full-face view, frontal carinae straight or weakly convex; frontal lobes curved inward in front of antennal insertion. Anterior clypeal margin broadly and evenly convex; clypeal psammophore set at or above midpoint of clypeus; palp formula 6,4. Five mandibular teeth in major worker; mandibles triangular, weakly incurved; third mandibular tooth distinctly shorter than apical tooth and teeth numbers two and four; masticatory margin of mandibles approximately aligned vertically or weakly oblique. **Mesosoma.** Integument of pronotum, mesonotum and mesopleuron matt or with weak sheen and microreticulate throughout; anterior mesosoma in profile pronotum smoothly rounded anteriad and flattened posteriad, mesonotum narrowly convex; erect pronotal setae short, (i.e., shorter than length of eye) and unmodified; in profile, metanotal groove shallow, broadly V- or U-shaped; propodeum shining, dorsum and declivitous face of propodeum mainly smooth, but with weak to strong vertical striolae arising from metapleuron; propodeum wedge-shaped, tapering dorsad; length ratio of propodeal dorsum to its declivity between 3:2 and 4:3; erect propodeal setae absent; appressed propodeal setae short, separated by more than own length and inconspicuous; propodeal spiracle situated on or beside declivitous face of propodeum, and shorter (length less than 0.50 × height of propodeum). **Petiole.** In profile, petiolar node squamiform; in full-face view, shape of petiolar node uniformly rounded; node shining and smooth with vestigial microreticulation anteriad. **Gaster.** Gaster smooth and glossy; pilosity of first gastral tergite consisting of well-spaced, erect and semi-erect setae interspersed with regularly spaced appressed setae. **General characters.** Colour concolorous light brown or orange tan.

#### Measurements.

Worker (n = 8): CI 95–111; EI 25–45; EL 0.18–0.19; HL 0.41–0.70; HW 0.40–0.78; ML 0.55–0.90; MTL 0.26–0.44; PpH 0.06–0.09; PpL 0.23–0.39; SI 65–88; SL 0.35–0.50.

#### Comments.

*Melophorus microreticulatus* is a reasonably common small *Melophorus* thus far recorded from arid or semi-arid parts of NSW, NT, QLD, SA and WA, but it undoubtedly also occurs in western Victoria (one record coming from Brookfield Conservation Park near the SA, Vic border). This species is distinctive through its large eye, netted microreticulate mesosoma, protuberant propodeum and small size. As mentioned above, it fits in a clade that includes several other small species that possess a scalloped or otherwise sculptured mesopleuron and distinctive propodeum. The smoother specimens suggest a likely close relationship with *M. biroi*.

A tiny minor worker (M317) from Canna in the mid north of WA was successfully sequenced for COI. This specimen and the voucher that accompanied it are atypical for *M. microreticulatus*, with smaller (but still large) eyes, a slightly longer antennal scape and a narrower head capsule, but share with that species the characteristic netted sculpture and narrow, porrect node. The specimens may represent a separate species near to *M. microreticulatus*, but equally could simply be nanitic workers from a new colony. On the COI tree M317 was sister to the clade that includes *M. gracilis*, *M. mjobergi*, and *M. postlei*. No other specimens were available for sequencing. Specimens collected at Lake Mere were taken from a paddock. The principal author of this work has also collected the species in tall eucalyptus woodland east of Southern Cross, WA. A worker was collected in mulga woodland at Merigol Station, QLD. Apart from these records, ecological data are absent.

#### Etymology.

Compound of Greek *mikros* (‘small’) plus Latin *reticulatus* (‘netted’); adjective in the nominative singular.

**Figure 42.**
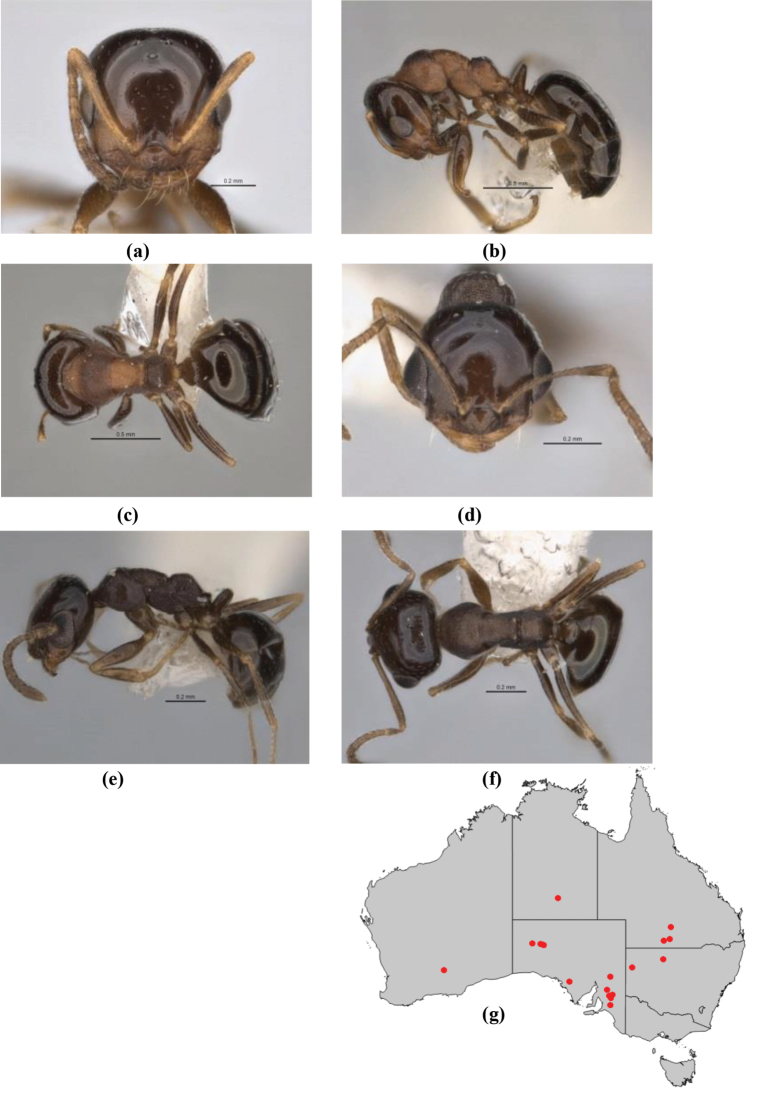
*Melophorus microreticulatus* sp. n.: major worker paratype (ANIC32-900171–bottom ant) frons (**a**), profile (**b**) and dorsum (**c**); minor worker holotype (ANIC32-900172) frons (**d**), profile (**e**) and dorsum (**f**); distribution map for the species (**g**). Low resolution scale bars: 0.5 mm (**b, c**); 0.2 mm (**a, d–f**).

### Melophorus minimus

Taxon classificationAnimaliaHymenopteraFormicidae

Heterick, Castalanelli & Shattuck sp. n.

http://zoobank.org/D717B6CC-1649-43A0-A425-735EF1394368

#### Types.

Holotype minor worker (bottom ant) from St George (‘Saint George’-*sic*), Queensland, 14 January 1965, B.B. Lowery, sav. Woodland, ANIC Ants Vial 20.215 [ANIC32-900133] (ANIC). Paratypes: 2 minor workers on same pin and with same details as holotype (ANIC); minor worker from 50 km E of Cunnamulla, Queensland, 17 September 1976, P.J.M. Greenslade, (9), 21 [ANIC32-900132] (BMNH); 3 minor workers on separate pin with same details as holotype (MCZ); minor worker from Milang Conservation Park, Koonamore, South Australia, 24 February 1973, P.J.M. Greenslade, (8), *M.* sp. 34) (SAM); major and 2 minor workers from Tropicana Minesite 29°15'40"S, 124°35'50"E, Western Australia, January 2009, J. Summerhayes, pitfall trap *Casuarina*, CA 1:5 [JDM32-005036] (WAM).

#### Other material examined.

**New South Wales**: Warialda (Oliver, T.). **Queensland**: ‘Merigol’ (Beutel, T.) (QM), Tindaree via Tara (House, A & Brown, S.) (QM).

#### Diagnosis.

*Melophorus minimus* can be placed in the *M. biroi* species-group on the basis of characters of the clypeus, propodeum, mandible and palps. The species is placed in the *M. biroi* species-complex on the basis of a further suite of characters (viz, metatibia of major worker with only one preapical spur [except rarely in the *mjobergi* clade]; clypeal psammophore placed anteriorly at or just above anterior margin of clypeus in the minor worker and often in the major worker; head dorsoventrally compressed to varying degrees in the minor worker of most species with the eyes placed high on the sides; compact legs, and small body size [excluding *mjobergi* clade]; HW of smallest minor 0.36 mm, average HW of smallest minors 0.46 mm; HW of largest known major 1.29 mm, average HW of largest majors [where known] 1.05 mm). *Melophorus minimus* is the tiniest *Melophorus* with a head width of 0.47 mm in one measured major worker. Apart from minute size, the minor worker of this species has a distinctive profile with a strongly truncate, cuboidal propodeum that descends into its declivitous face at an angle approaching ninety degrees. The minor worker eye is large (EI 40-41) and the antennal scape is shorter than in most *Melophorus* (SI as little as 94). The mesosoma and first gastral tergite are always glabrous. The size of the major worker alone is sufficient to identify it, but its large eye combined with its smooth, glabrous mesosoma also separate it from other small *Melophorus* major workers in the *M. biroi* species-complex.

#### Minor worker description.

**Head.** Head approximately oval with straight sides; posterior margin of head planar to strongly convex; frons shining with superficial shagreenation or microreticulation only; frons consisting exclusively or almost exclusively of well-spaced, appressed setae only (small, erect setae, if present, usually confined to ocular triangle or posterior margin of head). Eye moderate (eye length 0.20–0.49 length of side of head capsule); in full-face view, eyes set at about midpoint of head capsule; in profile, eye set anteriad of midline of head capsule; eyes elliptical or slightly reniform. In full-face view, frontal carinae concave; frontal lobes straight in front of antennal insertion. Anteromedial clypeal margin broadly and evenly convex; clypeal psammophore set at or just above anterior clypeal margin; palp formula 6,4. Five mandibular teeth in minor worker; mandibles triangular, weakly incurved; third mandibular tooth distinctly shorter than apical tooth and teeth numbers two and four; masticatory margin of mandibles approximately vertical or weakly oblique. **Mesosoma.** Integument of pronotum, mesonotum and mesopleuron shining and mainly smooth, vestigial shagreenation most noticeable on humeri and mesopleuron; anterior mesosoma in profile weakly elevated anteriad, thereafter gently sinuate, pronotum and mesonotum on same plane; erect pronotal setae absent; in profile, metanotal groove a weak or vestigial furrow; propodeum shining and shagreenate; propodeum angulate, propodeal angle blunt; length ratio of propodeal dorsum to its declivity about1:1; erect propodeal setae always absent; appressed propodeal setulae sparse or absent, if present then not regularly spaced; propodeal spiracle situated on or beside declivitous face of propodeum, and longer (length ≥ 0.50 × height of propodeum). **Petiole.** In profile, petiolar node squamiform; in full-face view, shape of petiolar node uniformly rounded; node shining and smooth with vestigial sculpture. **Gaster.** Gaster shining, shagreenate (‘LP record’ appearance); pilosity of first gastral tergite consisting of well-spaced short, inconspicuous, appressed setae only, erect setae always absent. **General characters.** Colour mainly concolorous brown; mesonotum may be orange tan.

#### Major worker description.

**Head.** Head square; posterior margin of head planar; cuticle of frons shining and smooth except for piliferous pits and a few striolae around antennal insertions and frontal carinae; frons consisting exclusively or almost exclusively of well-spaced, appressed setae only (small, erect setae, if present, usually confined to ocular triangle or posterior margin of head). Eye moderate (eye length 0.20–0.49 length of head capsule); in full-face view, eyes set at about midpoint of head capsule; in profile, eye set anteriad of midline of head capsule; eyes elliptical, or roughly ovoid, eye narrowed posteriad. In full-face view, frontal carinae straight, divergent posteriad; frontal lobes curved towards antennal insertion. Anterior clypeal margin broadly and evenly convex; clypeal psammophore set below midpoint of clypeus; palp formula 6,4. Five mandibular teeth in major worker; mandibles triangular, weakly incurved; third mandibular tooth distinctly shorter than apical tooth and teeth numbers two and four; masticatory margin of mandibles approximately aligned vertically or weakly oblique. **Mesosoma.** with weak to moderate sheen and superficial microreticulation; anterior mesosoma in profile broadly convex; erect pronotal setae absent; in profile, metanotal groove a narrow but deep slit; propodeum shining and superficially microreticulate; propodeum smoothly rounded; propodeal dorsum and declivity confluent; erect propodeal setae absent; appressed propodeal setae sparse or absent, if present then not regularly spaced; propodeal spiracle situated on or beside declivitous face of propodeum, and shorter (length less than 0.50 × height of propodeum). **Petiole.** In profile, petiolar node squamiform; in full-face view, shape of petiolar node tapered with blunt vertex; node shining and smooth with vestigial microreticulation. **Gaster.** Gaster shining, shagreenate (‘LP record’ appearance); pilosity of first gastral tergite consisting of short, well-spaced appressed setae only. **General characters.** Colour dark brown, lateral mesonotum lighter in colour.

#### Measurements.

Worker (n = 4): CI 88–96; EI 40–41; EL 0.14–0.17; HL 0.37–0.44; HW 0.33–0.47; ML 0.43–0.55; MTL 0.23–0.28; PpH 0.06–0.06; PpL 0.19–0.24; SI 94–104; SL 0.34–0.39.

#### Comments.

With a total length of only around one millimetre, the minor workers of this species are probably the smallest *Melophorus* and are likely ecological competitors with small *Monomorium* of the same dimensions. At present the species is known from a handful of records from arid and semi-arid NSW (TERC), QLD, SA and WA, but it has probably been overlooked in other mainland states because of its minute size. This species can be identified by a combination of its small size, short antennal scape, glossy appearance, anteriorly located clypeal psammophore, and square propodeum. No specimens were available for sequencing. Recorded habits are remnant brigalow, savanna woodland and mulga. Specimens from Merigol Station, QLD and Tindaree via Tara, QLD were pitfall-trapped but there are no other data.

#### Etymology.

Latin (dim.) *minimus* (‘least’); adjective in the nominative singular.

**Figure 43.**
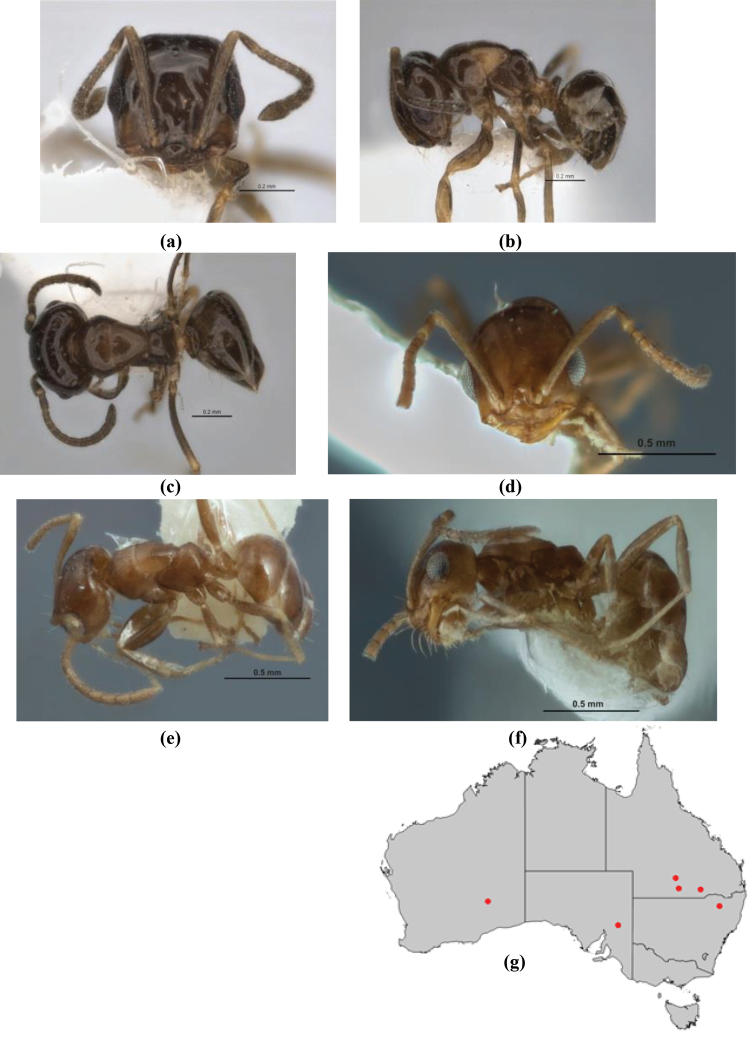
*Melophorus minimus* sp. n.: major worker paratype (JDM32- 005036–top ant) frons (**a**), profile (**b**) and dorsum (**c**); minor worker holotype (ANIC32-900133–bottom ant) frons (**d**), profile (**e**) and dorsum (**f**); distribution map for the species (**g**). Low resolution scale bars: 0.5 mm (**d–f**); 0.2 mm (**a–c**).

### Melophorus mjobergi

Taxon classificationAnimaliaHymenopteraFormicidae

Forel

Melophorus mjobergi Forel 1915: 88 (combination in M. (Erimelophorus) by Wheeler 1935: 71).

#### Types.

Syntype (likely) minor worker, Broome, Western Australia [ANIC] (examined: ANIC specimen The minor worker lacks Forel’s red ‘typus’ label, but possesses a handwritten label with ‘type’ on it and very probably belongs to the series of eight workers inspected by Forel when he described this species, despite the presence of a ‘co-type’ label.) Also examined: Images of syntype minor worker, Broome, Western Australia [MHNG] [NHRS] (AntWeb images of MHNG specimen CASENT0909819, images of NHRS specimen [image no. NHRS-HEVA 000003949]). The CASC ant is accompanied by a red ‘typus’ label, but the NHRS specimen is accompanied by a handwritten label only.

#### Other material examined.

**Northern Territory**: nr Limbunya turnoff (Heterick, B.E. [M225]). **Western Australia**: 14 km E Roebuck Plains RH (Heterick, B.E. [M191]), 30 km N Sandfire Hill (Heterick, B.E. [M183]), 4 km N Cape Leveque turnoff (Heterick, B.E. [M250]), 4.3 km N Stanley rest area (Heterick, B.E. [M255]), 47 km E Fitzroy Crossing (Heterick, B.E. [M238]), 5 km N Pardoo Hill (Heterick, B.E. [M264]), Argyle Diamond Mine via Kununurra (Postle, A. [JDM32-001567]), Argyle Diamond Mine via Kununurra (Postle, A. [JDM32-001566]), Argyle Diamonds via Kununurra (Postle, A.T. [JDM32-001561]), Argyle Diamonds via Kununurra (Postle, A.T. [JDM32-001563]), Dampier Downs turnoff (Heterick, B.E. [M252]), Erskine Ra (Heterick, B.E. [M192]), Ethel Creek (Singarayar, F.K. [JDM32-004667]), Lissadell Rd turnoff (Heterick, B.E. [M212]), Nita Downs turnoff (Heterick, B.E. [M260]), Pardoo Hill (Heterick, B.E. [M179]), Willie Creek turnoff (Heterick, B.E. [M277]).

#### Diagnosis.

*Melophorus mjobergi* can be placed in the *M. biroi* species-group on the basis of characters of the clypeus, propodeum, mandible and palps. The species is also placed in the *M. mjobergi* clade because of characters best seen in the major worker. These include (seen in full-face view) the deeply recessed area around the frontal carinae and medial sector of clypeus visible in the major and media workers, the psammophore generally placed on or just above anterior margin of clypeus, and the minor workers hairy with bristly, short erect setae. In *Melophorus mjobergi* the head of all workers is microreticulate and moderately shining to matt and the setae-bearing sockets on the head of the major worker are impressed so as to appear as small, oval depressions. In full-face view, the antennal carina of the major worker is limited to a flange around the antennal insertion, a weak ridge representing the posterior extension of carina is strongly concave posteriad. This will differentiate this ant from *M. compactus*. Also, unlike *M. compactus*, the eye of the minor worker is placed anteriorly on head capsule and is moderate in size (in profile, eye length ≈ 0.25× length of side of head capsule). *Melophorus mjobergi* is very similar to *M. postlei* but the appressed setae in all workers is loose and relatively thick and does not obscure the underlying cuticle, which is moderately shining in appearance.

#### Minor worker description.

**Head.** Head approximately oval with straight sides; posterior margin of head strongly convex; frons matt or with weak sheen, microreticulate or microreticulate-shagreenate; pilosity of frons a mixture of a few well-spaced, erect setae interspersed with appressed setae only, or consisting of appressed pubescence, with many short, unmodified, erect setae. Eye moderate (eye length 0.20–0.49 length of side of head capsule); in full-face view, eyes set above midpoint of head capsule; in profile, eye set around midline of head capsule; eyes elliptical or slightly reniform. In full-face view, frontal carinae straight, divergent posteriad; frontal lobes straight in front of antennal insertion. Anteromedial clypeal margin broadly and evenly convex; clypeal psammophore set at or just above anterior clypeal margin; palp formula 6,4. Five mandibular teeth in minor worker; mandibles triangular, weakly incurved; third mandibular tooth distinctly shorter than apical tooth and teeth numbers two and four; masticatory margin of mandibles approximately vertical or weakly oblique. **Mesosoma.** Integument of pronotum, mesonotum and mesopleuron moderately shining and shagreenate throughout; anterior mesosoma in profile smoothly rounded anteriad, thereafter pronotum and whole of mesonotum flattened and on same plane as propodeum; appearance of erect pronotal setae short, (i.e., longest erect setae shorter than length of eye) and unmodified; in profile, metanotal groove shallow, indicated mainly by an angle; propodeum shining and shagreenate; propodeum angulate, propodeal angle blunt; length ratio of propodeal dorsum to its declivity between 1:1 and 1:2; erect propodeal setae present and sparse to moderate (1-12); appressed propodeal setulae long and closely aligned, creating pubescence, or long, each reaching setae behind and in front, but not forming pubescence; propodeal spiracle situated on or beside declivitous face of propodeum, and longer (length ≥ 0.50 × height of propodeum). **Petiole.** In profile, petiolar node squamiform; in full-face view, shape of petiolar node uniformly rounded; node shining and distinctly shagreenate-microreticulate. **Gaster.** Gaster weakly shining with indistinct shagreenation; pilosity of first gastral tergite consisting of well-spaced, short, thick, erect setae over long, closely aligned, whitish, appressed setae. **General characters.** Colour concolorous light brown.

#### Major worker description.

**Head.** Head square; posterior margin of head planar or weakly concave; cuticle of frons matt or with weak sheen, with many fine, longitudinal striolae anteriad; pilosity of frons a mixture of many short, erect, bristly setae interspersed with regularly spaced appressed setae. Eye small (eye length < 0.20 × length of head capsule); in full-face view, eyes set above midpoint of head capsule; in profile, eye set anteriad of midline of head capsule; eyes elliptical. In full-face view, frontal carinae distinctly concave; frontal lobes curved toward antennal insertion. Anterior clypeal margin narrowly convex and protruding anteromedially, clypeal margin entire or weakly indented; clypeal psammophore set at or just above anterior clypeal margin; palp formula 6,4. Four mandibular teeth in major worker; mandibles triangular, weakly incurved; third mandibular tooth distinctly shorter than apical tooth and teeth number two, but longer and narrower than basal tooth; masticatory margin of mandibles approximately aligned vertically or weakly oblique. **Mesosoma.** Integument of pronotum, mesonotum and mesopleuron matt or with weak sheen and microreticulate throughout; anterior mesosoma in profile convex anteriad, straight thereafter; erect pronotal setae short, (i.e., shorter than length of eye) and unmodified; in profile, metanotal groove shallow, narrowly V-shaped; propodeum matt or with a weak sheen and microreticulate; propodeum smoothly rounded or with indistinct angle; propodeal dorsum and declivity confluent; erect propodeal setae present and abundant (at least a dozen); no appressed propodeal setae, but a few relatively long decumbent setae present, not creating pubescence; propodeal spiracle situated on or beside declivitous face of propodeum, and longer (length greater than 0.50 × height of propodeum). **Petiole.** In profile, petiolar node narrowly squamiform; in full-face view, shape of petiolar node tapered with blunt vertex; node shining and superficially microreticulate. **Gaster.** Gaster shining with indistinct shagreenation; pilosity of first gastral tergite consisting of a mixture of curved, erect and semi- erect setae and decumbent setae that are not sufficiently close to form pubescence. **General characters.** Colour uniformly light olive brown.

#### Measurements.

Worker (n = 8): CI 89–89; EI 29–30; EL 0.16–0.17; HL 0.63–0.65; HW 0.56–0.58; ML 0.86–0.88; MTL 0.57–0.60; PpH 0.08–0.10-; PpL 0.35–0.36; SI 136–137; SL 0.76–0.79.

#### Comments.

*Melophorus mjobergi* is difficult to distinguish from *M. postlei*, but is shiny rather than matt in appearance and the appressed setae on the mesopleuron and gaster are more widely separated and do not obscure the underlying cuticle. However, on the five-gene and COI gene trees the two taxa are distinctly separate although closely related. The known distribution of the species is QLD, SA (TERC – one locality) and WA. The principal author has recorded the ‘*pillipes*’ condition in worker material from Killarney Stn in NT (TERC), but this characteristic is otherwise confined to the members of the *fieldi* and *anderseni* complexes (to which this ant is unrelated) and this note requires further investigation.

*Melophorus mjobergi* is very common in the Pilbara and Kimberley regions of WA, where it was among the most abundant *Melophorus* taken by the principal author in roadside collections. Collections were made in a variety of habitats including sand plain, red clay soil, grassy woodland, tall grass over light soil, the crest of a small sand dune, a station rubbish dump with much woody litter and grasses, and on a grassed road verge near a petrol station. This considerably augments previous information on the species: as with so many other *Melophorus*, earlier collectors omitted to record any data for this small ant apart from the fact that several specimens were collected in pitfall traps and one worker from Avon Downs Stn, NT, was taken ‘on flowers’.

**Figure 44.**
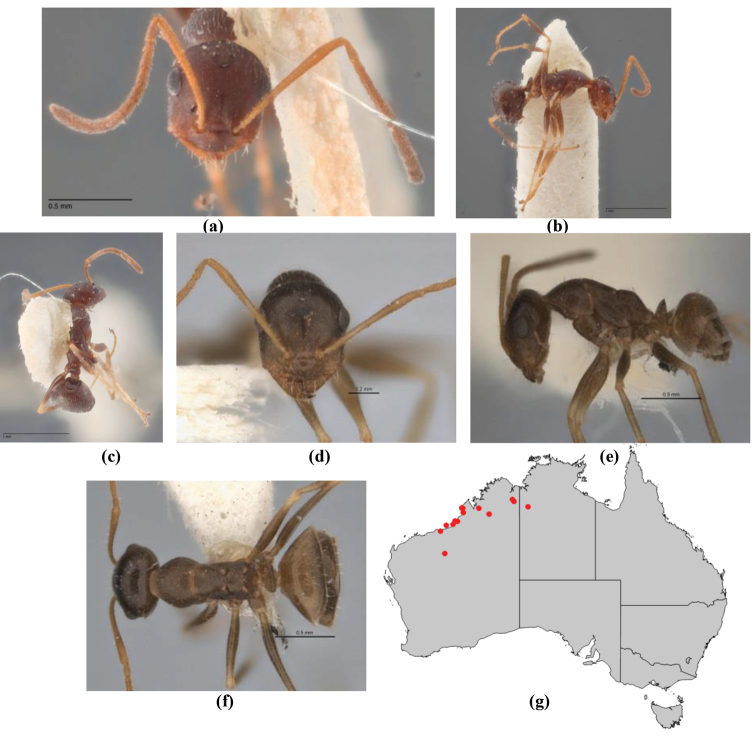
*Melophorus mjobergi* Forel: NHRS minor worker syntype (NHRS-HEVA 000003949) frons (**a**), profile (**b**) and dorsum (**c**); non–type minor worker (JDM32-004667–bottom ant) frons (**d**), profile (**e**) and dorsum (**f**); distribution map for the species (**g**). Low resolution scale bars: 1 mm (**b, c**); 0.5 mm (**e–f**); 0.2 mm (**a, d**).

### Melophorus postlei

Taxon classificationAnimaliaHymenopteraFormicidae

Heterick, Castalanelli & Shattuck sp. n.

http://zoobank.org/06F99F08-7308-4176-B80C-E66CABED61CF

#### Types.

Holotype minor worker from Kapalga, Northern Territory, 16 January 1983, P.J.M. Greenslade, Sai, Sa [ANIC32-900181] (ANIC). Paratypes: 3 major workers from Rimbija Is., Wessell Islands, 11.01S, 136.45 E, Northern Territory, 3-14 February 1977; T. A. Weir [ANIC32-900087] (ANIC); 2 minor workers from Kapalga (pencil), Alligator Rivers area 7-9 September 1983, P.J.M. Greenslade, 8i traps (BMNH); 2 minor workers from Manbulloo, SW Katherine, Northern Territory, 7-11 April 1978, P.J.M. Greenslade, 25 (MCZ).

#### Other material examined.

**Northern Territory**: 30 km SW Katherine (Greenslade, P.J.M.), Avon Downs (Colles, D.H.), Kapalga (Greenslade, P.J.M. [ANIC32-900181]), Kapalga (Greenslade, P.J.M.), Manbulloo Research Station (Gross, G.), Manbulloo, SW Katherine (Greenslade, P.J.M.), Manbulloo, SW Katherine (Greenslade, P.J.M. [ANIC32-900086]), nr Limbunya turnoff (Heterick, B.E. [M221]), **Western Australia**: Dunham River (rest area) (Heterick, B.E. [M216]), 100 km N Meekatharra (Heath, M & Turpin, J. [JDM 32-004896]).

#### Diagnosis.

*Melophorus postlei* can be placed in the *M. biroi* species-group on the basis of characters of the clypeus, propodeum, mandible and palps. The species is also placed in the *M. mjobergi* clade because of characters best seen in the major worker. These include (seen in full-face view) the deeply recessed area around the frontal carinae and medial sector of clypeus visible in the major and media workers, the psammophore generally placed on or just above anterior margin of clypeus, and the minor workers hairy with bristly, short erect setae. In *Melophorus postlei* the head of all workers is microreticulate and moderately shining to matt and the setae-bearing sockets on the head of the major worker are impressed so as to appear as small, oval depressions. In full-face view, the antennal carina of the major worker is limited to a flange around the antennal insertion, a weak ridge representing the posterior extension of carina is strongly concave posteriad. This will differentiate this ant from *M. compactus*. Also, unlike *M. compactus*, the eye of the minor worker is placed anteriorly on head capsule and is moderate in size (in profile, eye length ≈ 0.25× length of side of head capsule). *Melophorus postlei* can be distinguished from its near relative, *M. mjobergi*, in that the appressed setae in all workers is fine and forms pubescence that largely obscures the underlying cuticle, which is matt in appearance.

#### Minor worker description.

**Head.** Head square; posterior margin of head strongly convex; frons matt or with weak sheen, microreticulate or microreticulate-shagreenate; frons consisting of appressed pubescence, with many short, unmodified, erect setae. Eye moderate (eye length 0.20–0.49 length of side of head capsule); in full-face view, eyes set above midpoint of head capsule; in profile, eye set anteriad of midline of head capsule; eyes elliptical or slightly reniform. In full-face view, frontal carinae straight or weakly convex; frontal lobes straight in front of antennal insertion. Anteromedial clypeal margin broadly and evenly convex; clypeal psammophore set at or above midpoint of clypeus; palp formula 6,4. Five to six mandibular teeth in minor worker; mandibles triangular, weakly incurved; third mandibular tooth distinctly shorter than apical tooth and teeth numbers two and four; masticatory margin of mandibles approximately vertical or weakly oblique. **Mesosoma.** Integument of pronotum, mesonotum and mesopleuron matt or with weak sheen and microreticulate throughout; anterior mesosoma in profile smoothly rounded anteriad, thereafter pronotum and whole of mesonotum flattened and on same plane as propodeum; appearance of erect pronotal setae short, (i.e., longest erect setae shorter than length of eye) and unmodified; in profile, metanotal groove a weak or vestigial furrow; propodeum shining and microreticulate, or matt or with a weak sheen and microreticulate; propodeum smoothly rounded or with indistinct angle; propodeal dorsum and declivity confluent; erect propodeal setae present and sparse to moderate (1-12); appressed propodeal setulae long and closely aligned, creating pubescence; propodeal spiracle situated on or beside declivitous face of propodeum, and longer (length ≥ 0.50 × height of propodeum). **Petiole.** In profile, petiolar node squamiform; in full-face view, shape of petiolar node tapered with blunt vertex; node shining and distinctly microreticulate. **Gaster.** Gaster weakly shining with indistinct shagreenation; pilosity of first gastral tergite consisting of thick, appressed setae that form pubescence, interspersed with numerous short, bristly, erect setae. **General characters.** Colour brown to chocolate (gaster may be darker than foreparts).

#### Major worker description.

**Head.** Head quadrate (i.e., heart-shaped); posterior margin of head planar or weakly concave; cuticle of frons matt or with weak sheen, microreticulate; pilosity of frons a mixture of many short, erect, bristly setae interspersed with regularly spaced appressed setae. Eye moderate (eye length 0.20–0.49 length of head capsule); in full-face view, eyes set at about midpoint of head capsule; in profile, eye set anteriad of midline of head capsule; eyes elliptical. In full-face view, frontal carinae distinctly concave; frontal lobes curved toward antennal insertion. Anterior clypeal margin narrowly convex and protruding anteromedially, clypeal margin entire or weakly indented; clypeal psammophore set at or just above anterior clypeal margin; palp formula 6,4. Four mandibular teeth in major worker; mandibles triangular, weakly incurved; third mandibular tooth distinctly shorter than apical tooth and teeth numbers two and four; masticatory margin of mandibles approximately aligned vertically or weakly oblique. **Mesosoma.** Integument of pronotum, mesonotum and mesopleuron matt or with weak sheen and microreticulate throughout; anterior mesosoma in profile gently sinuous after initial steep pronotal incline; erect pronotal setae short, (i.e., shorter than length of eye) and unmodified; in profile, metanotal groove shallow, broadly V- or U-shaped; propodeum matt or with a weak sheen and microreticulate; propodeum smoothly rounded or with indistinct angle; propodeal dorsum and declivity confluent; erect propodeal setae present and abundant (at least a dozen); appressed propodeal setae long and closely aligned, creating pubescence; propodeal spiracle situated on or beside declivitous face of propodeum, and shorter (length less than 0.50 × height of propodeum). **Petiole.** In profile, petiolar node squamiform; in full-face view, shape of petiolar node tapered with blunt vertex; node shining and distinctly microreticulate. **Gaster.** Gaster weakly shining with indistinct shagreenation; pilosity of first gastral tergite consisting of a mixture of curved, erect and semi-erect setae and decumbent setae that form a variable pubescence. **General characters.** Colour uniformly reddish-brown.

#### Measurements.

Worker (n = 6): CI 87–114; EI 17–33; EL 0.15–0.23; HL 0.53–1.16; HW 0.46–1.33; ML 0.73–1.34; MTL 0.47–0.75; PpH 0.08–0.15; PpL 0.31–0.57; SI 61–144; SL 0.66–0.81.

#### Comments.

This small, neat *Melophorus* can be recognized by its thick pubescence, which obscures the underlying cuticle on the mesopleuron and forms a thatch on the first gastral tergite. Most Collections come from the NT, but the ant also occurs in the Kimberley and northern goldfields regions of WA and (in all probability) in far northern QLD. Sequencing data for two specimens confirm their sister relationship to *M. mjobergi*, which they very closely resemble. A mallee habitat ‘on sand’ is the only ecological note from labels for this species.

#### Etymology.

After Dr. Tony Postle; noun in the genitive case.

**Figure 45.**
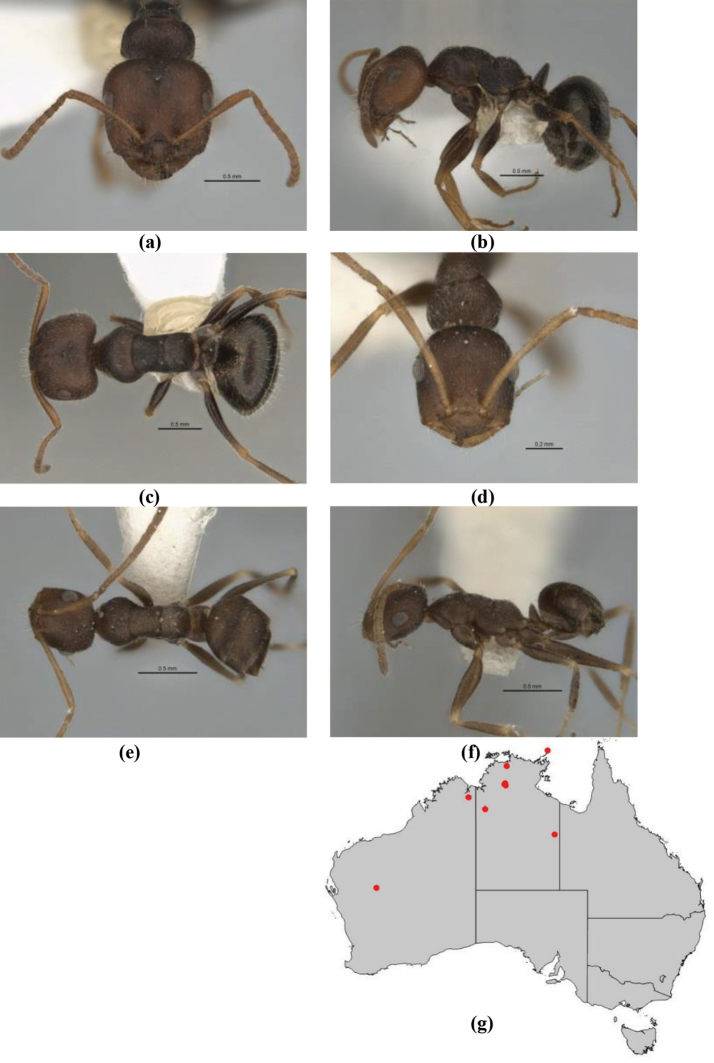
*Melophorus postlei* sp. n.: major worker paratype (ANIC32-900087–middle worker) frons (**a**), profile (**b**) and dorsum (**c**); minor worker holotype (ANIC32-900081) frons (**d**), profile (**e**) and dorsum (**f**); distribution map for the species (**g**). Low resolution scale bars: 0.5 mm (**a–c, e, f**); 0.2 mm (**d**).

### Melophorus propebiroi

Taxon classificationAnimaliaHymenopteraFormicidae

Heterick, Castalanelli & Shattuck sp. n.

http://zoobank.org/FB8F2745-60B4-4E99-96B1-EBF1EE3D8C15

#### Types.

Holotype minor worker from Perth, Western Australia, 14 October 1976, J. D. Majer, P. trap RH10 A1089 [JDM32-001947] (WAM). Paratypes: minor worker from Murdoch 32°03'59"S, 115°51'07"E, Western Australia, August, 2011, C. Smithyman, control (S2/T2): remnant bushland adjacent to South Street Railway Station, Curtin University JDM Collection donated 13 Jan 2015 (WAM); minor worker from Bunbury, Western Australia, 20 December 1969, B.B. Lowery [ANIC32-900112] (ANIC).

#### Diagnosis.

*Melophorus propebiroi* can be placed in the *M. biroi* species-group on the basis of characters of the clypeus, propodeum, mandible and palps. The species is placed in the *M. biroi* species-complex on the basis of a further suite of characters (viz, metatibia of major worker with only one preapical spur [except rarely in the *mjobergi* clade]; clypeal psammophore placed anteriorly at or just above anterior margin of clypeus in the minor worker and often in the major worker; head dorsoventrally compressed to varying degrees in the minor worker of most species with the eyes placed high on the sides; compact legs, and small body size [excluding *mjobergi* clade]; HW of smallest minor 0.36 mm, average HW of smallest minors 0.46 mm; HW of largest known major 1.29 mm, average HW of largest majors [where known] 1.05 mm). The minor worker of *M. propebiroi* closely resembles *M. biroi* but has relatively long, partially overlapping appressed setae that form a weak pubescence on the mesosoma and gaster and also numerous short, bristly, erect setae on the mesosoma and gaster. The pilosity pattern enables this species to be distinguished from all other taxa in its species-complex. Only the minor worker is known.

#### Minor worker description.

**Head.** Head square; posterior margin of head planar or weakly convex; frons matt or with weak sheen, microreticulate or microreticulate-shagreenate; pilosity of frons a mixture of a few well-spaced, erect setae interspersed with appressed setae only. Eye moderate (eye length 0.20–0.49 length of side of head capsule); in full-face view, eyes set above midpoint of head capsule; in profile, eye set anteriad of midline of head capsule; eyes elliptical or slightly reniform. In full-face view, frontal carinae straight, divergent posteriad; frontal lobes straight in front of antennal insertion. Anteromedial clypeal margin broadly and evenly convex; clypeal psammophore set at or just above anterior clypeal margin; palp formula 6,4. Five mandibular teeth in minor worker; mandibles triangular, weakly incurved; third mandibular tooth distinctly shorter than apical tooth and teeth numbers two and four; masticatory margin of mandibles approximately vertical or weakly oblique. **Mesosoma.** Integument of pronotum, mesonotum and mesopleuron with weak to moderate sheen, shagreenate on pronotum and dorsum of mesonotum, otherwise microreticulate; anterior mesosoma in profile smoothly rounded anteriad, thereafter pronotum and whole of mesonotum flattened and on same plane as propodeum; appearance of erect pronotal setae short, (i.e., longest erect setae shorter than length of eye) and unmodified; in profile, metanotal groove a narrow but deep slit; propodeum shining and densely microreticulate, with distinct striolae on metapleuron; propodeum angulate, propodeal angle blunt; length ratio of propodeal dorsum to its declivity greater than 1:2; erect propodeal setae always absent; appressed propodeal setulae long and closely aligned, creating pubescence; propodeal spiracle situated on or beside declivitous face of propodeum, and longer (length ≥ 0.50 × height of propodeum). **Petiole.** In profile, petiolar node squamiform; in full-face view, shape of petiolar node tapered with squared-off vertex; node shining and distinctly microreticulate. **Gaster.** Gaster shining with superficial microreticulation; pilosity of first gastral tergite consisting of well-spaced, short, thick, erect setae over long, closely aligned, whitish, appressed setae. **General characters.** Colour concolorous brown.

#### Measurements.

Worker (n = 2): CI 97–101; EI 24–25; EL 0.15–0.16; HL 0.64–0.67; HW 0.59–0.67; ML 0.82–0.91; MTL 0.44–0.51; PpH 0.10–0.10; PpL 0.37–0.41; SI 112–119; SL 0.70–0.76.

#### Comments.

As with *M. latinotus*, this species appears to be confined to woodland on the Swan Coastal Plain, WA, where it appears occasionally in collections. Apart from the material recorded here, this ant has also been taken in the Gingin area, near the Mitchell Freeway and at Bold Park. The major worker is unknown. *Melophorus propebiroi* looks very much like a hirsute *M. biroi*, and its weak pubescence, small eye and general pilosity enable it to be distinguished from other, similar species that occur with it in sympatry. No specimens were available for sequencing and nothing is known of its habits.

#### Etymology.

Latin *prope* (‘near’) plus *biroi*.

**Figure 46.**
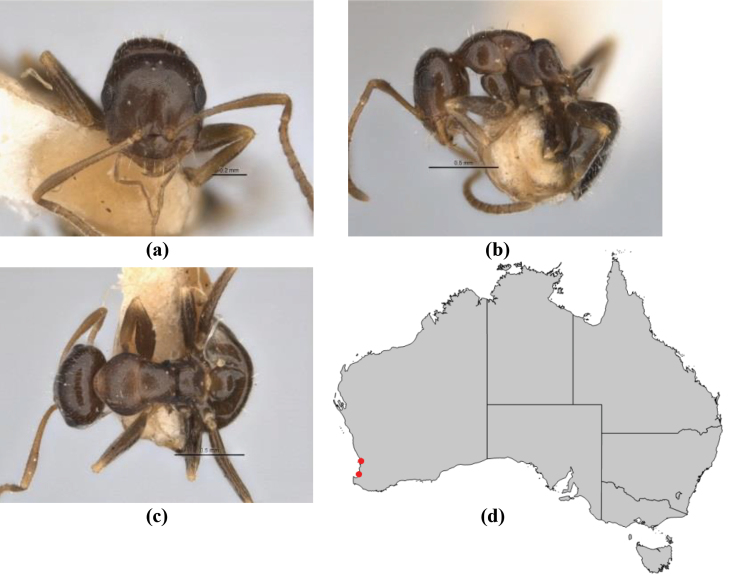
*Melophorus propebiroi* sp. n.: minor worker holotype (JDM32-001947) frons (**a**), profile (**b**) and dorsum (**c**); distribution map for the species (**d**). Low resolution scale bars: 0.5 mm (**b, c**); 0.2 mm (**a**).

### Melophorus turbineus

Taxon classificationAnimaliaHymenopteraFormicidae

Heterick, Castalanelli & Shattuck sp. n.

http://zoobank.org/04F327C1-FEFC-40C2-A5F6-29D387F1FC20

#### Types.

Holotype minor worker (bottom ant) from Gawler Ranges, South Australia, 4 October 1972, P.J.M. Greenslade, (5), 4/10 (5), *M.* sp. 8 (ANIC32- 066636) (ANIC). Paratypes: Minor worker on same pin and with same details as holotype (damaged after photograph taken) (ANIC); minor worker from Gawler Ranges, Western Australia, 4 October 1972, P.J.M. Greenslade, (5) [ANIC-900100] (MCZ).

#### Diagnosis.

*Melophorus turbineus* can be placed in the *M. biroi* species-group on the basis of characters of the clypeus, propodeum, mandible and palps. The species is placed in the *M. biroi* species-complex on the basis of a further suite of characters (viz, metatibia of major worker with only one preapical spur [except rarely in the *mjobergi* clade]; clypeal psammophore placed anteriorly at or just above anterior margin of clypeus in the minor worker and often in the major worker; head dorsoventrally compressed to varying degrees in the minor worker of most species with the eyes placed high on the sides; compact legs, and small body size [excluding *mjobergi* clade]; HW of smallest minor 0.36 mm, average HW of smallest minors 0.46 mm; HW of largest known major 1.29 mm, average HW of largest majors [where known] 1.05 mm). Next to *M. minimus*, *M. turbineus* is probably the smallest *Melophorus*. The minor worker has a distinctive propodeum that is produced vertically and is conical in profile. The propodeal spiracle is very large, being approximately 0.75× height of propodeum. The eye is also large (eye length approximately 0.40× length of side of head capsule). These characteristics make this species readily recognisable. The major worker is unknown.

#### Minor worker description.

**Head.** Head approximately oval with straight sides; posterior margin of head weakly convex, or planar or weakly convex; frons shining and smooth except for piliferous pits; frons consisting exclusively or almost exclusively of well-spaced, appressed setae only (small, erect setae, if present, usually confined to ocular triangle or posterior margin of head). Eye moderate (eye length 0.20–0.49 length of side of head capsule); in full-face view, eyes set at about midpoint of head capsule; in profile, eye set around midline of head capsule; roughly ovoid, eye narrowed posteriad. In full-face view, frontal carinae distinctly concave; frontal lobes curved inward in front of antennal insertion. Anteromedial clypeal margin straight and retroussé anteromedially; clypeal psammophore set at or just above anterior clypeal margin; palp formula 6,4. Five mandibular teeth in minor worker to seven; mandibles triangular, weakly incurved; third mandibular tooth distinctly shorter than apical tooth and teeth numbers two and four; masticatory margin of mandibles approximately vertical or weakly oblique. **Mesosoma.** Integument of pronotum, mesonotum and mesopleuron shining and smooth on dorsum, entire lower mesopleuron distinctly striolate-microreticulate; anterior mesosoma in profile pronotum smoothly rounded anteriad and flattened posteriad, mesonotum narrowly convex; erect pronotal setae absent; in profile, metanotal groove shallow, broadly V or U-shaped; propodeum shining, with multiple hair like striolae; propodeum sharply conical; length ratio of propodeal dorsum to its declivity not applicable, propodeal dorsum reduced to a narrow sliver; erect propodeal setae always absent; appressed propodeal setulae sparse or absent, if present then not regularly spaced; propodeal spiracle situated on or beside declivitous face of propodeum, and longer (length ≥ 0.50 × height of propodeum). **Petiole.** In profile, petiolar node squamiform; in full-face view, shape of petiolar node uniformly rounded; node shining and smooth throughout. **Gaster.** Gaster smooth and glossy; pilosity of first gastral tergite consisting of well-spaced short, inconspicuous, appressed setae only, erect setae always absent. **General characters.** Colour of head and gaster chocolate, mesosoma tan.

#### Measurements.

Worker (n = 2): CI 98–100; EI 33–35; EL 0.13–0.14; HL 0.37–0.43; HW 0.36–0.43; ML 0.53–0.54; MTL 0.24–0.28; PpH 0.06–0.07; PpL 0.18–0.21; SI 88–96; SL 0.35–0.38.

#### Comments.

Minor workers of this tiny species are recognizable because of their roundly conical propodeum when seen in profile, and their large propodeal spiracle. Like other members of their clade, they have a scalloped mesopleuron. No sequencing work has been done on the taxon. The major worker is unknown and the minor worker material is confined to two pins of ants collected in the Gawler Ranges, SA. There are no accompanying collection data (apart from the fact that the worker on one pin would have been pitfall-trapped by Greenslade) and nothing more is known of this ant.

#### Etymology.

Latin *turbineus* (‘conical’); adjective in nominative case.

**Figure 47.**
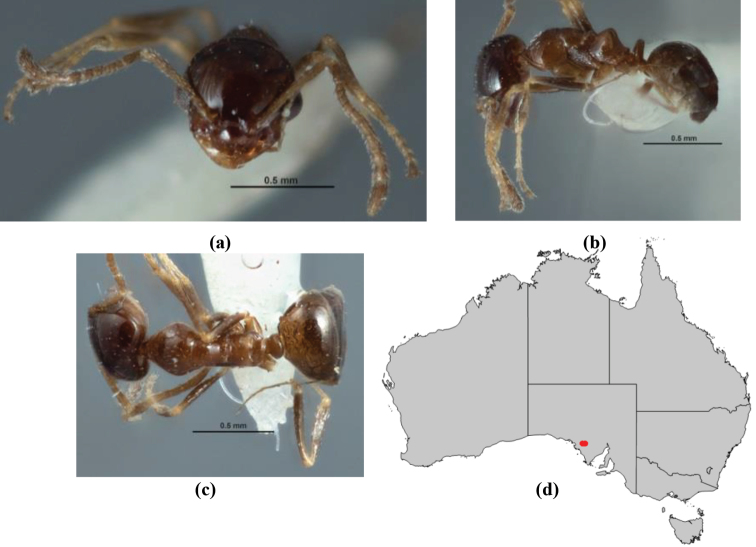
*Melophorus turbineus* sp. n.: minor worker holotype (ANIC32-066636–bottom ant) frons (**a**), profile (**b**) and dorsum (**c**); distribution map for the species (**d**).

### *Melophorus brevignathus* complex

This is a small group (three species) of slightly odd-looking *Melophorus* with large, blocky, square heads, narrow mandibles and, especially in the case of *M. brevignathus* and *M. quadratus*, small, anteriorly-positioned, flattened eyes and a strongly sinuate anterior clypeal margin. *Melophorus marmar* appears less closely related to the other pair of taxa but shares the diagnosis for this small complex. The media and minor worker of *M. marmar* have large, protrusive eyes and overall these subcastes strongly resemble smaller workers of *Melophorus diversus*. However, the conformation of the clypeus and the coarsely striate nature of the five-toothed mandible in *M. marmar* easily separate the two species. *Melophorus marmar* has been sequenced, and the molecular data suggest a possible relationship with the *M. wheeleri* complex.

### Melophorus brevignathus

Taxon classificationAnimaliaHymenopteraFormicidae

Heterick, Castalanelli & Shattuck sp. n.

http://zoobank.org/F5249470-AA26-44A5-BE05-0D4F737E8DFB

#### Types.

Holotype minor worker (bottom ant) from Saint George [*sic*], Queensland, 15 January 1965, B.B. Lowery, sav. Woodland, ANIC Ants Vial 20.216 [ANIC32-900055] (ANIC). Paratypes: 2 major workers on same pin and with same details as holotype (ANIC); 2 minor workers from Kunoth Paddock, Northern Territory on separate pin but with exactly the same details but lacking small label ‘10’ (BMNH); 2 media workers and a minor worker from Kunoth Paddock, near Alice Springs, Northern Territory, 26 October 1974, P.J.M. Greenslade, (12), 10 (MCZ); 2 major workers and a minor worker from 75 km W of Sandringham HS, Queensland, 6 June 1980, P.J.M. Greenslade, (5), 20) (QM).

#### Other material examined.

**Northern Territory**: Kunoth Paddock, near Alice Springs (Greenslade, P.J.M.). **Queensland**: St. George (Lowery, B.B.).

#### Diagnosis.

*Melophorus brevignathus* can be placed in the *M. biroi* species-group on the basis of characters of the clypeus, propodeum, mandible and palps. Furthermore, this species can also be placed in the *M. brevignathus* species-complex. This species-complex has the following diagnostic characters: in full-face view the head capsules of the major, media and minor workers are square with small, flattened eyes (except in the media and minor workers of *M. marmar*, which have a large, convex eye [but the eye is flattened in the major worker]); in profile, the eyes are placed anteriad of the midline of head capsule; the anterior margin of the clypeus is distinctly sinuate, projecting anteromedially as a bluntly triangular extension or flattened dimple in major and media workers; the five-toothed mandible of all workers is very narrow, parallel and coarsely striate throughout its length (broader and more finely striate in most members of the *M. fieldi* and the *M. biroi* species-complexes); and the maxillary palps in all workers is short, barely attaining neck sclerite at their greatest extension and often only reaching the midpoint of venter of head capsule when the head is moderately inclined. The eye of *Melophorus brevignathus* is placed low on head capsule, its bottom third intersected by an imaginary horizontal line separating head capsule (excluding mandibles) into equal upper and lower sectors, thus separating it from *M. quadratus*. The species can be distinguished from *M. marmar* by the following: in full-face view, the head is distinctly convex and the appressed setae on the first gastral tergite in all workers are shorter and separated from the preceding and succeeding rows by at least 2× their own length. The eyes of the minor worker are similar to those of major worker.

#### Minor worker description.

**Head.** Head square; posterior margin of head weakly concave; frons shining with superficial shagreenation or microreticulation only; frons consisting exclusively or almost exclusively of well-spaced, appressed setae only (small, erect setae, if present, usually confined to ocular triangle or posterior margin of head). Eye moderate (eye length 0.20–0.49 length of side of head capsule); in full-face view, eyes set above midpoint of head capsule; in profile, eye set anteriad of midline of head capsule; eyes elliptical or slightly reniform. In full-face view, frontal carinae straight, divergent posteriad; frontal lobes straight in front of antennal insertion. Anteromedial clypeal margin sinuate with anteromedial dimple; clypeal psammophore set below midpoint of clypeus; palp formula 6,4. Mandibular teeth in minor worker four to five; mandibles narrow, mandibular blade truncate, internal and external margins parallel or nearly so; third mandibular tooth distinctly shorter than apical tooth and tooth no. two, tooth no. four vestigial; masticatory margin of mandibles approximately vertical or weakly oblique. **Mesosoma.** Integument of pronotum, mesonotum and mesopleuron moderately shining and shagreenate throughout; anterior mesosoma in profile broadly convex; appearance of erect pronotal setae short, (i.e., longest erect setae shorter than length of eye) and unmodified, or erect pronotal setae absent; in profile, metanotal groove shallow, broadly V or U-shaped; propodeum shining and microreticulate; propodeum angulate, propodeal angle blunt; length ratio of propodeal dorsum to its declivity between 1:1 and 1:2; erect propodeal setae always absent; appressed propodeal setulae short, separated by more than own length and inconspicuous; propodeal spiracle situated on or beside declivitous face of propodeum, and shorter (length < 0.50 × height of propodeum). **Petiole.** In profile, petiolar node squamiform; in full-face view, shape of petiolar node tapered with blunt vertex; node shining and distinctly microreticulate. **Gaster.** Gaster shining with superficial microreticulation; pilosity of first gastral tergite consisting of well-spaced, erect and semi-erect setae interspersed with regularly placed appressed setae. **General characters.** Colour of foreparts brown to dark brown, gaster blackish-brown.

#### Major worker description.

**Head.** Head as for minor worker; posterior margin of head weakly concave; cuticle of frons shining with superficial shagreenation or microreticulation only; frons consisting exclusively or almost exclusively of well-spaced, appressed setae only (small, erect setae, if present, usually confined to ocular triangle or posterior margin of head). Eye moderate (eye length 0.20–0.49 length of head capsule); in full-face view, midpoint of head capsule; in profile, eye set anteriad of midline of head capsule; eyes elliptical. In full-face view, frontal carinae straight, divergent posteriad; frontal lobes straight in front of antennal insertion, or curved inward in front of antennal insertion. Anterior clypeal margin sinuate with anteromedial dimple; clypeal psammophore set below midpoint of clypeus; palp formula 6,4. Mandibular teeth in major worker 4-5; mandibles narrow, mandibular blade truncate, internal and external margins parallel or nearly so; third mandibular tooth distinctly shorter than apical tooth, but equivalent in length to remaining teeth; masticatory margin of mandibles approximately aligned vertically or weakly oblique. **Mesosoma.** Integument of pronotum, mesonotum and mesopleuron moderately shining and shagreenate throughout; anterior mesosoma in profile broadly convex; erect pronotal setae short, (i.e., shorter than length of eye) and unmodified; in profile, metanotal groove shallow, broadly V- or U-shaped; propodeum shining and finely striolate and microreticulate; propodeum angulate, propodeal angle blunt; length ratio of propodeal dorsum to its declivity between 1:1 and 1:2; erect propodeal setae absent; appressed propodeal setae short, separated by more than own length and inconspicuous; propodeal spiracle situated on or beside declivitous face of propodeum, and shorter (length less than 0.50 × height of propodeum). **Petiole.** In profile, petiolar node squamiform; in full-face view, shape of petiolar node uniformly rounded, or tapered with blunt vertex; node shining and distinctly microreticulate. **Gaster.** Gaster shining with superficial microreticulation; pilosity of first gastral tergite consisting of well-spaced, erect and semi-erect setae interspersed with regularly spaced appressed setae, or consisting of short, bristly, erect setae over well-spaced, short, appressed setae. **General characters.** Colour of foreparts reddish-brown or brown, gaster blackish-brown.

#### Measurements.

Worker (n = 6): CI 109–112; EI 16–23; EL 0.21–0.26; HL 0.84–1.44; HW 0.92–1.62; ML 1.00–1.56; MTL 0.67–1.02; PpH 0.12–0.18; PpL 0.41–0.63; SI 66–84; SL 0.77–1.06.

#### Comments.

*Melophorus brevignathus* is distinguished from *Melophorus quadratus* by the position of the eye towards the middle of the head capsule, and from *Melophorus marmar* by other details of the head capsule, the size of the eyes in the minor worker and the appearance of the setae on the first gastral tergite. This species has been collected in box pine scrub over red soil and in savanna woodland in QLD and it also occurs in the NT. The species has not been sequenced and no other details are known.

#### Etymology.

Latin *brevis* (‘short’) plus Neo-Latin *gnathus* (‘jaw’); adjective in the nominative singular.

**Figure 48.**
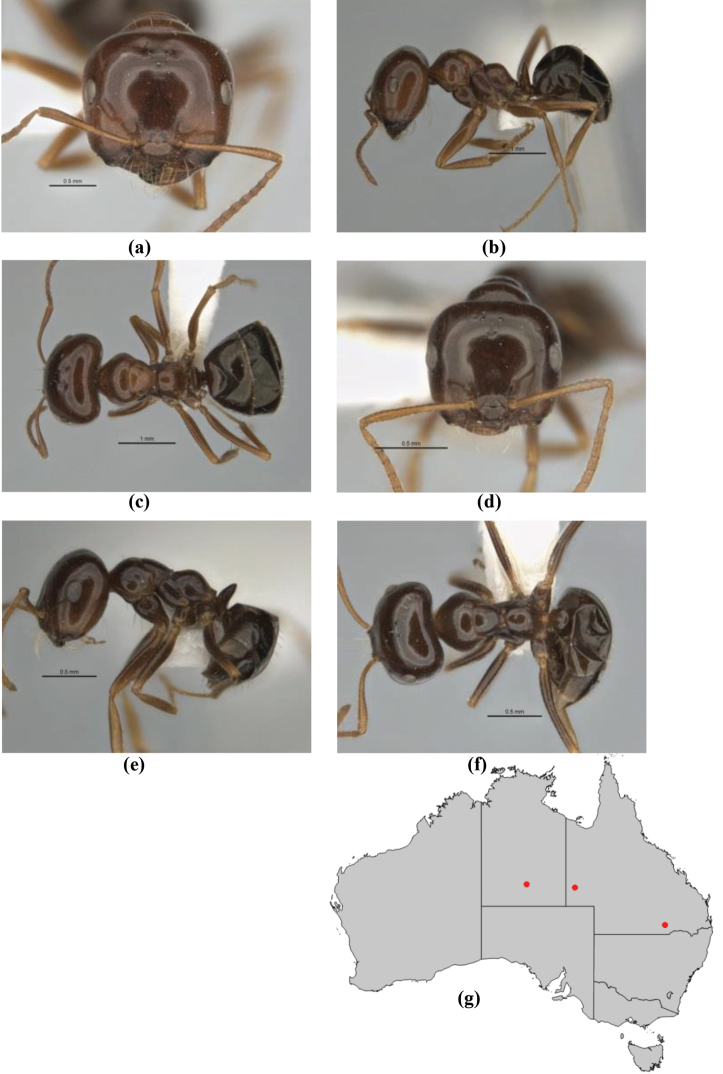
*Melophorus brevignathus* sp. n.: major worker paratype (ANIC32-900055–middle ant) frons (**a**), profile (**b**) and dorsum (**c**); minor worker holotype (ANIC32-900055–bottom ant) frons (**d**), profile (**e**) and dorsum (**f**); distribution map for the species (**g**). Low resolution scale bars: 1 mm (**b, c**); 0.5 mm (**a, d–f**).

### Melophorus marmar

Taxon classificationAnimaliaHymenopteraFormicidae

Heterick, Castalanelli & Shattuck sp. n.

http://zoobank.org/BDCFF8E7-A098-4F8E-96A6-E77F1B366267

#### Types.

Minor worker holotype (bottom ant) from 16 km E of Fred Spring, Curdimurka, South Australia, 2 October 1978, P.J.M. Greenslade, inside fence on veg., 1E [ANIC32-900054] (ANIC). Paratypes: major and minor worker on same pin and with same details as holotype (ANIC); major worker, media worker and minor worker from Davenport Springs, 40 km W of Maree, South Australia, 30 September 1978, P.J.M. Greenslade, near water, 38. 3 (BMNH); major worker, media worker and minor worker from c. 27 km S of William Creek, South Australia 29.05s × 136.31E, South Australia, 22 September 1972, J.E. Feehan, ANIC Ants Vial 16.59 (MCZ).

#### Other material examined.

**Western Australia**: Lake Austin (Heterick, B.E. [M287/M288/M289]), Lake Lefroy (Gordon, R.).

#### Diagnosis.

*Melophorus marmar* species can be placed in the *M. biroi* species-group on the basis of characters of the clypeus, propodeum, mandible and palps. Furthermore, this species can also be placed in the *M. brevignathus* species-complex. This species-complex has the following diagnostic characters: in full-face view the head capsules of the major, media and minor workers are square with small, flattened eyes (except in the media and minor workers of *M. marmar*, which have a large, convex eye [but the eye is flattened in the major worker]); in profile, the eyes are placed anteriad of the midline of head capsule; the anterior margin of the clypeus is distinctly sinuate, projecting anteromedially as a bluntly triangular extension or flattened dimple in major and media workers; the five-toothed mandible of all workers is very narrow, parallel and coarsely striate throughout its length (broader and more finely striate in most members of the *M. fieldi* and the *M. biroi* species-complexes); and the maxillary palps in all workers is short, barely attaining neck sclerite at their greatest extension and often only reaching the midpoint of venter of head capsule when the head is moderately inclined. The eye of *Melophorus marmar* is placed low on head capsule, its bottom third intersected by an imaginary horizontal line separating head capsule (excluding mandibles) into equal upper and lower sectors, thus separating it from *M. quadratus*. The species can be distinguished from *M. brevignathus* by the following: in full-face view, the head is straight or even weakly concave and the appressed setae on the first gastral tergite in all workers are longer and separated from the preceding and succeeding rows by ≥ 1× their own length. The eyes of the minor and media workers large and convex, those of the major worker flat.

#### Minor worker description.

**Head.** Head square; posterior margin of head planar to weakly convex; frons shining with superficial shagreenation or microreticulation only; pilosity of frons a mixture of a few well-spaced, erect setae interspersed with appressed setae only. Eye moderate (eye length 0.20–0.49 length of side of head capsule); in full-face view, eyes set above midpoint of head capsule; in profile, eye set anteriad of midline of head capsule; eyes elliptical or slightly reniform. In full-face view, frontal carinae straight or weakly convex; frontal lobes straight in front of antennal insertion. Anteromedial clypeal margin straight, or broadly and evenly convex with or without a vestigial anteromarginal dimple; clypeal psammophore set at or just above anterior clypeal margin; palp formula 6,4. Five to six mandibular teeth in minor worker; mandibles narrow, mandibular blade truncate, internal and external margins parallel or nearly so; third mandibular tooth slightly shorter than apical tooth, but equivalent in length to remaining teeth; masticatory margin of mandibles approximately vertical or weakly oblique. **Mesosoma.** Integument of pronotum, mesonotum and mesopleuron shining and mainly smooth, vestigial shagreenation most noticeable on humeri and mesopleuron; anterior mesosoma in profile smoothly rounded anteriad, mesonotum flattened and projecting marginally over pronotum and on same plane as propodeum; appearance of erect pronotal setae short, (i.e., longest erect setae shorter than length of eye) and unmodified, or erect pronotal setae absent; in profile, metanotal groove shallow, broadly V or U-shaped; propodeum shining and smooth or with superficial and almost invisible microsculpture; propodeum rounded or angulate, propodeal angle blunt; length ratio of propodeal dorsum to its declivity about 1:1; erect propodeal setae always absent; appressed propodeal setulae short, separated by more than own length and inconspicuous; propodeal spiracle situated on or beside declivitous face of propodeum, and shorter (length < 0.50 × height of propodeum). **Petiole.** In profile, petiolar node squamiform; in full-face view, shape of petiolar node tapered with blunt vertex; node shining and distinctly shagreenate-microreticulate. **Gaster.** Gaster shining with superficial microreticulation; pilosity of first gastral tergite consisting of well-spaced, erect and semi-erect setae interspersed with regularly placed appressed setae. **General characters.** Colour shining brown to black, antennae yellow to light brown, legs often dark medially and conspicuously paler near joints.

#### Major worker description.

**Head.** Head as for minor worker; posterior margin of head weakly concave; cuticle of frons shining and smooth except for piliferous pits; pilosity of frons a mixture of well-spaced, distinctly longer erect and semi-erect setae interspersed with shorter decumbent setae. Eye moderate (eye length 0.20–0.49 length of head capsule), or small, (eye length less than 0.2 × length of head capsule); in full-face view, eyes set at midpoint of head capsule; in profile, eye set anteriad of midline of head capsule; eyes elliptical. In full-face view, frontal carinae straight or weakly convex; frontal lobes straight in front of antennal insertion. Anterior clypeal margin weakly sinuate with anteromedial dimple; clypeal psammophore set below midpoint of clypeus; palp formula 6,4. Five mandibular teeth in major worker; mandibles narrow, strap-like, internal and external borders parallel or nearly so; third mandibular tooth distinctly shorter than apical tooth, but equivalent in length to remaining teeth; masticatory margin of mandibles approximately aligned vertically or weakly oblique. **Mesosoma.** Integument of pronotum, mesonotum and mesopleuron shining with very superficial microreticulation, entire lower mesopleuron distinctly shagreenate, or moderately shining and shagreenate throughout; anterior mesosoma in profile broadly convex; erect pronotal setae short, (i.e., shorter than length of eye) and unmodified; in profile, metanotal groove shallow, broadly V- or U-shaped; propodeum shining and shagreenate; propodeum angulate, propodeal angle blunt; length ratio of propodeal dorsum to its declivity between 1:1 and 1:2; erect propodeal setae present and abundant (at least a dozen), or present and sparse to moderate (1-12); appressed propodeal setae long, each reaching setae behind and in front, but not forming pubescence; propodeal spiracle situated on or beside declivitous face of propodeum, and shorter (length less than 0.50 × height of propodeum). **Petiole.** In profile, petiolar node squamiform; in full-face view, shape of petiolar node tapered with blunt vertex, or tapered with sharp vertex; node shining and distinctly microreticulate. **Gaster.** Gaster shining with superficial microreticulation; pilosity of first gastral tergite consisting of a mixture of curved, erect and semi-erect setae and decumbent setae that form a variable pubescence. **General characters.** Colour shining brown, legs paler distally.

#### Measurements.

Worker (n = 4): CI 105–112; EI 16–18; EL 0.21–0.25; HL 1.09–1.40; HW 1.15–1.57; ML 1.21–1.56; MTL 0.88–1.13; PpH 0.13–0.16; PpL 0.49–0.60; SI 69–76; SL 0.88–1.09.

#### Comments.

In WA, *Melophorus marmar* was seen by the principal author nesting in Lake Austin (see Figs 3(d), 4) and collecting seeds, leaves and possibly carrion from the lake surrounds. On Lake Austin, the ant builds a cup-shaped mound of soil around the nest hole which is five or more centimetres above the dry lake surface, possibly as insurance against waterlogging. Some nests seen were more than forty metres from the edge of the Lake. This species also occurs in SA, and may be the *Melophorus* investigated by Schultheiss and his co-workers (Schultheiss et al. 2012; Cheng et al. 2014), as part of a study on navigation in desert ants. Label data for three workers collected at Davenport Springs, 40 km W Marree indicate that they were collected ‘near water’.

#### Etymology.

Old Irish *marmar* (‘marble’): after the shining appearance of the ant; adjective in the nominative singular.

**Figure 49.**
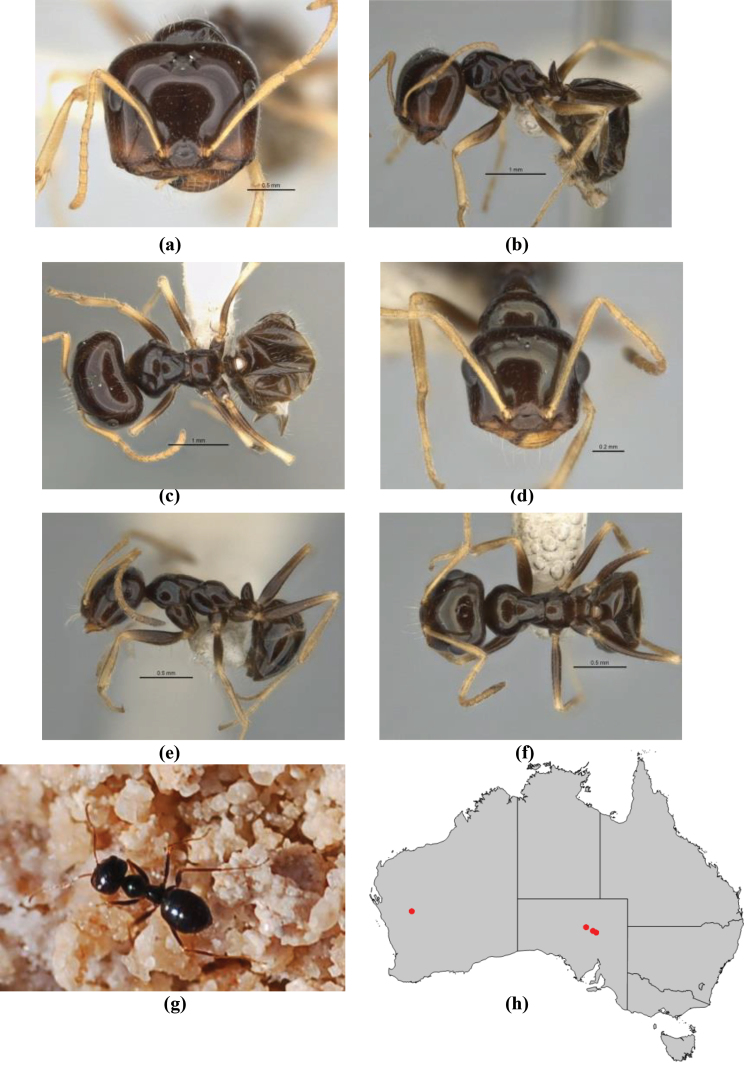
*Melophorus marmar* sp. n.: major worker paratype (ANIC32-900054–top ant) frons (**a**), profile (**b**) and dorsum (**c**); minor worker holotype (ANIC32-900054–bottom ant) frons (**d**), profile (**e**) and dorsum (**f**); *Melophorus marmar* forager on the dry surface of Lake Austin (**g**); distribution map for the species (**h**). Low resolution scale bars: 1 mm (**b, c**); 0.5 mm (**a, e, f**); 0.2 mm (d).

### Melophorus quadratus

Taxon classificationAnimaliaHymenopteraFormicidae

Heterick, Castalanelli & Shattuck sp. n.

http://zoobank.org/151E6B29-4CD2-4D08-AB58-B3CBC1070B49

#### Types.

Holotype minor worker (bottom ant) from c. 9 km E of Finke, Northern Territory, 30 September 1972, J.E. Feehan, ANIC Ants Vial 15.155 [ANIC32-900053]. Paratypes: Major worker on same pin and with same details as holotype (damaged) (ANIC); 3 minor workers from Birthday Hill, N. Tarcoola, 3 October 1976, P.J.M. Greenslade, (2) (ANIC); 2 major workers from 5 km E Kingoonya 30.55S, 135.23E, South Australia, 30 September 1981, D. Davidson/S. Morton, 59a (BMNH); 3 minor workers from Mt Davies turnoff, Victoria Desert, South Australia, 8 October 1976, P.J.M. Greenslade, (2) [ANIC32-900183] (MCZ). major worker and minor worker from Vokes Hill, Victoria Desert, South Australia, 8 October 1976, P.J.M Greenslade (6)a (SAM).

#### Other material examined.

**South Australia**: 3 km W Emu Camp, Victoria Desert (Greenslade, P.J.M.), Emu Camp, Victoria Desert (Greenslade, P.J.M.), Lake Meramangye, Victoria Desert (Greenslade, P.J.M.).

#### Diagnosis.

*Melophorus quadratus* can be placed in the *M. biroi* species-group on the basis of characters of the clypeus, propodeum, mandible and palps. Furthermore, this species can also be placed in the *M. brevignathus* species-complex. This species-complex has the following diagnostic characters: in full-face view the head capsules of the major, media and minor workers are square with small, flattened eyes (except in the media and minor workers of *M. marmar*, which have a large, convex eye [but the eye is flattened in the major worker]); in profile, the eyes are placed anteriad of the midline of head capsule; the anterior margin of the clypeus is distinctly sinuate, projecting anteromedially as a bluntly triangular extension or flattened dimple in major and media workers; the five-toothed mandible of all workers is very narrow, parallel and coarsely striate throughout its length (broader and more finely striate in most members of the *M. fieldi* and the *M. biroi* species-complexes); and the maxillary palps in all workers is short, barely attaining neck sclerite at their greatest extension and often only reaching the midpoint of venter of head capsule when the head is moderately inclined. Unlike *M. brevignathus* and *M. marmar*, *M. quadratus* has the eye, in full-face view, placed very high on capsule, and slightly above an imaginary horizontal line separating the head capsule (excluding mandibles) into equal upper and lower sectors; moreover the workers are glabrous (erect mesosomal setae are present in the other two species in the complex).

#### Minor worker description.

**Head.** Head square, or quadrate (i.e., heart-shaped); posterior margin of head planar or weakly concave, or strongly concave; frons matt or with weak sheen, microreticulate or microreticulate-shagreenate; frons consisting exclusively or almost exclusively of well-spaced, appressed setae only (small, erect setae, if present, usually confined to ocular triangle or posterior margin of head). Eye small (eye length less than 0.2 × length of side of head capsule); in full-face view, eyes set above midpoint of head capsule; in profile, eye set anteriad of midline of head capsule; eyes elliptical or slightly reniform. In full-face view, frontal carinae straight, divergent posteriad; frontal lobes straight in front of antennal insertion. Anteromedial clypeal margin broadly emarginate with projecting anteromedial dimple; clypeal psammophore set at or above midpoint of clypeus; palp formula 6,4. Five mandibular teeth in minor worker; mandibles narrow, mandibular blade truncate, internal and external margins parallel or nearly so; third mandibular tooth distinctly shorter than apical tooth and teeth numbers two and four; masticatory margin of mandibles approximately vertical or weakly oblique. **Mesosoma.** Integument of pronotum, mesonotum and mesopleuron with weak to moderate sheen and superficial microreticulation (more pronounced on mesopleuron); anterior mesosoma in profile broadly convex; erect pronotal setae absent; in profile, metanotal groove shallow, indicated mainly by an angle; propodeum shining and microreticulate; propodeum smoothly rounded or with indistinct angle; propodeal dorsum and declivity confluent; erect propodeal setae always absent; appressed propodeal setulae short, separated by more than own length and inconspicuous; propodeal spiracle situated at least twice its width from the declivitous face of propodeum, and shorter (length < 0.50 × height of propodeum). **Petiole.** In profile, petiolar node squamiform; in full-face view, shape of petiolar node tapered with blunt vertex; node shining and distinctly microreticulate. **Gaster.** Gaster shining, shagreenate (‘LP record’ appearance); pilosity of first gastral tergite consisting of well-spaced short, inconspicuous, appressed setae, erect setae (present in at least some workers) confined to margin of sclerite. **General characters.** Colour blackish-brown.

#### Major worker description.

**Head.** Head as for minor worker; posterior margin of head weakly concave; cuticle of frons shining with superficial shagreenation or microreticulation only; frons consisting exclusively or almost exclusively of well-spaced, appressed setae only (small, erect setae, if present, usually confined to ocular triangle or posterior margin of head). Eyes small, (eye length less than 0.2 × length of head capsule); in full-face view, midpoint of head capsule; in profile, eye set anteriad of midline of head capsule; eyes elliptical. In full-face view, frontal carinae straight, divergent posteriad; frontal lobes curved inward in front of antennal insertion. Anterior clypeal margin sinuate, weakly projecting anteromedially; clypeal psammophore set at or above midpoint of clypeus; palp formula 6,4. Five mandibular teeth in major worker; mandibles narrow, mandibular blade truncate, internal and external margins parallel or nearly so; third mandibular tooth distinctly shorter than apical tooth and teeth numbers two and four; masticatory margin of mandibles approximately aligned vertically or weakly oblique. **Mesosoma.** Integument of pronotum, mesonotum and mesopleuron with weak to moderate sheen, shagreenate on pronotum and dorsum of mesonotum, otherwise microreticulate; anterior mesosoma in profile broadly convex; erect pronotal setae absent; in profile, metanotal groove shallow, broadly V- or U-shaped; propodeum shining and microreticulate; propodeum always smoothly rounded; propodeal dorsum and declivity confluent; erect propodeal setae absent; appressed propodeal setae short, separated by more than own length and inconspicuous; propodeal spiracle situated at least twice its width from the declivitous face of propodeum, and shorter (length less than 0.50 × height of propodeum). **Petiole.** In profile, petiolar node squamiform; in full-face view, shape of petiolar node uniformly rounded; node shining and smooth with vestigial microreticulation anteriad. **Gaster.** Gaster shining, shagreenate (‘LP record’ appearance); pilosity of first gastral tergite consisting of well-spaced short, inconspicuous, appressed setae, erect setae (present in at least some workers) confined to margin of the sclerite. **General characters.** Colour blackish-brown.

#### Measurements.

Worker (n = 4): CI 105–112; EI 16–18; EL 0.21–0.25; HL 1.09–1.40; HW 1.15–1.57; ML 1.21–1.56; MTL 0.88–1.13; PpH 0.13–0.16; PpL 0.49–0.60; SI 69–76; SL 0.88–1.09.

#### Comments.

Workers of *Melophorus quadratus* are distinguished from those of the preceding two species by the high position of the eyes on the head capsule; workers are also glabrous. The species has been collected from remote localities in the Victoria Desert and from Tarcoola, SA, with one record from the Finke River, NT. Nothing more is known about this ant.

#### Etymology.

Latin *quadratus* (‘square’); adjective in the nominative singular.

**Figure 50.**
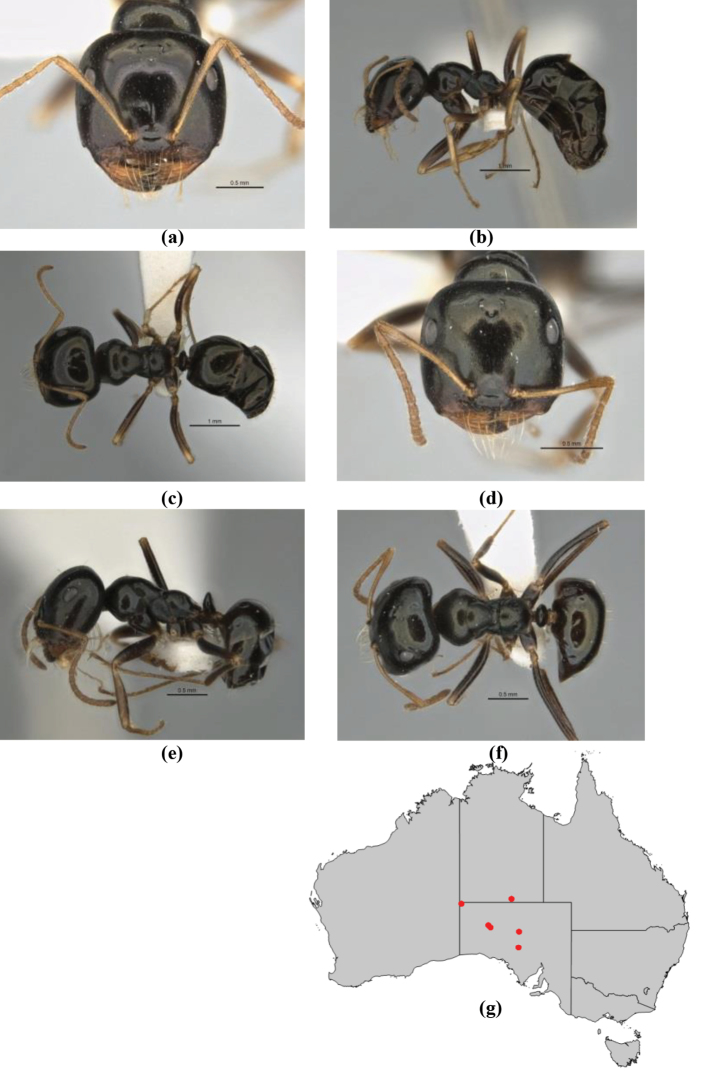
*Melophorus quadratus* sp. n.: major worker paratype (ANIC32-900053–top ant [damaged after photograph taken]) frons (**a**), profile (**b**) and dorsum (**c**); minor worker holotype (ANIC32-900053–bottom ant) frons (**d**), profile (**e**) and dorsum (**f**); distribution map for the species (**g**). Low resolution scale bars: 1 mm (**b, c**); 0.5 mm (**a, d–f**).

### *Melophorus fieldi* complex

This is far and away the most taxonomically testing of the *Melophorus* complexes with 23 species recognized here and others likely, and seems to represent an ongoing speciation event. Some of the most populous taxa (such as *M. hirsutipes*, *M. sulla* and *M. turneri*) show considerable morphological and genetic variation (and variation within one of these parameters is not always consonant with the variation seen in the other), and delimiting some species has been very difficult. *Melophorus turneri*, in fact, might be considered functionally as a ‘metaspecies’, with very unclear genetic boundaries between this ant and its presumed derivatives.

Phylogenetically speaking, the *M. fieldi* complex is the most recent of the *Melophorus* complexes tested, and has evidently undergone rapid speciation. Phylogenetic reconstruction of this complex is complicated by the lack of divergence found within the nuclear genes (see tables), incomplete lineage sorting, and mitochondrial homoplasy. The mitochondrial gene was useful in defining species boundaries apart from those for *M. sulla*, *M. turneri*, and *M. hirsutipes*, these species being polyphyletic on the COI tree. However, morphological evidence to support this result is lacking. When the third base of the COI gene was removed, the ESS scores and posterior probability values were increased, in the case of *M. sulla*, *M. turneri*, and *M. hirsutipes* helping consolidate these species into fewer clades, but the latter three species nonetheless still remained polyphyletic. However, a case for clearly defined polyphyly was not supported by the nuclear genes. For each of the abovementioned species there was either no genetic difference between groups based on the nuclear genes, or their mitochondrial clades were accompanied by a mixture of nuclear sequences. The value of using multiple genes rather than simple barcoding is informative here; if only the COI gene had been used to evaluate this material then the result would have necessitated splitting each of these three taxa into several additional species.

**Figure 51.**
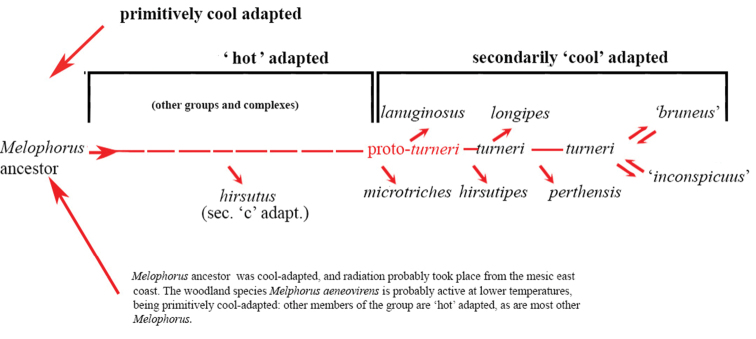
Speculative diagram depicting how cool–adapted foraging by *Melophorus* in the *M. fieldi* complex may have arisen.

There is some evidence that a clade of closely related ants in the *M. fieldi* complex has reverted to foraging in cooler weather. In May 2015, *Melophorus bruneus*, *M. hirsutipes*, *M. lanuginosus*, *M. longipes* and *M. turneri* were collected by the senior author in woodland north of Wiluna. The ants were active in relatively low temperatures (the shade temperature was in the high teens to low twenties Celsius). No other *Melophorus* were found to be active. This restriction of foraging activity to a clade of closely related species (see Figure 51, above) suggests a secondary reversion to generalised foraging behaviour within a genus characterised by thermophilic activity. Unsurprisingly, these species are also among the most derived *Melophorus*. On the other hand, species such as *Melophorus aeneovirens* that likely resemble closely the ancestral form and live in cooler, more mesic environments may be primitively cool-adapted. The figure above suggests a possible evolutionary path leading to this change of behaviour. Taxa represented are those known or likely to be primarily or secondarily adapted to foraging in cool conditions or in both cool and warm conditions. The evolutionary steps are suggested by the molecular data shown elsewhere in this paper. Among the specimens collected were some with apparent hybrid traits (e.g., ‘*bruneus*-*turneri*’), hence the suggestion of gene flow to and from a parental species (here postulated to be the widespread *M. turneri* and its ancestor).

### Melophorus ankylochaetes

Taxon classificationAnimaliaHymenopteraFormicidae

Heterick, Castalanelli & Shattuck sp. n.

http://zoobank.org/8B43EC5C-23EC-433D-A08B-BE9453F59184

#### Types.

Holotype minor worker from Kwelkan 31°08'36"S, 117°59'43"E, Western Australia, 12 March 2006, M. Russell, Hand collected, grid survey [JDM32-001990] (WAM). Paratypes: major, media and two minor workers from 17 km S of Rabbit Flat, Northern Territory, 19 May 1986, P.J.M. Greenslade, (8), 8 (ANIC); 3 major workers from Finley, New South Wales, 16 February 1934, W. B. White [ANIC32-900091] (BMNH); 3 minor workers from 21 km S of Yuendumu, Northern Territory, 24 May 1986, P.J.M. Greenslade, (5), (MCZ); major worker from 29 km NNW of Balfour Downs 22°32'39"S, 120°47'57"E, Western Australia, 14 May 2006-13 August 2006, CALM Pilbara Survey, Site BDRN07: Ethylene glycol pitfalls [JDM32-004761] (SAM); 3 minor workers from 12 km N of Billabong RH 26°44'S, 114°35'E, Western Australia, 11 December 2001, Heterick, B.E., Light scrub: red soil: am [JDM32-001993] (WAM).

#### Other material examined.

**Northern Territory**: Bessie Spring, 8 km ESE Cape Crawford (Feehan, J.E.), Jabiru (Greenslade, P.J.M.), Kunoth Paddock, near Alice Springs (Greenslade, P.J.M.), Kunoth Paddock, near Alice Springs (Greenslade, P.J.M.), Tennant Creek (Field, J.F.). **Queensland**: ‘Gumbardo’ (Beutel, T.), ‘Merigol’ (Beutel, T.), ‘Paingo’ turnoff (Monteith & Cook), 20 km S Winton (nr. Bladensburg Natl. Pk.) (Shattuck, S.O. [ANIC32-039878]), 26 mi NNW Mt. Isa (Dowse, J.E.), St. George (Lowery, B.B.). **Western Australia**: 1 km W Canna (Heterick, B.E. [M321]), 30 km SE Kambalda (Walliss, N. [JDM32-004715]), G. J. Rd, 108 km E Carnarvon (Heterick, B.E. [M304]).

#### Diagnosis.

*Melophorus ankylochaetes* can be placed in the *M. biroi* species-group on the basis of characters of the clypeus, propodeum, mandible and palps. The species is also placed in the *M. fieldi* species-complex because of the appearance of the anteriorly placed clypeal psammophore, the compact propodeum, the presence of more than one preapical spine on the metatibia, at least in the major worker, the long, even spindly legs, and the unmodified mandible in the major worker. Furthermore, this species clusters with several others in the *M. fieldi* complex which share the following characters: gaster with curved erect setae, semi-erect setae and a few decumbent setae only, genuine appressed setae lacking; the body generally strongly sculptured and hirsute and the antennal scapes and legs with whorls of many fine, straight setae. *Melophorus ankylochaetes* is distinguishable from *M. incisus* as the mesonotum and propodeum are not globose; the mesonotum being separated from the mesopleuron and propodeum by a weak groove or indentation. *Melophorus ankylochaetes* can also be distinguished from populations of *Melophorus hirsutipes* with the ‘*pillipes*’ condition because of the narrow, squamiform petiolar node of major and media workers (when viewed in profile) and the shiny appearance of the head (more matt and even rugulose in *M. hirsutipes*). The minor worker is not so distinctive, but can still be separated from the minor worker of *M. hirsutipes* (“*pillipes*”) by the more shining appearance of the head and anterior mesosoma and by its rounded propodeum.

#### Minor worker description.

**Head.** Head square; posterior margin of head weakly convex; frons striolate anteriad, smooth and shining posteriad; pilosity of frons a mixture of short, erect and semi-erect setae interspersed with shorter decumbent setae and well-spaced, short, appressed setae. Eye moderate (eye length 0.20–0.49 length of side of head capsule); in full-face view, eyes set at about midpoint of head capsule; in profile, eye set anteriad of midline of head capsule; eyes elliptical or slightly reniform. In full-face view, frontal carinae straight or weakly convex; frontal lobes straight in front of antennal insertion. Anteromedial clypeal margin convex, weakly acuminate anteromedially; clypeal psammophore set at or above midpoint of clypeus; palp formula 6,4. Five mandibular teeth in minor worker; mandibles triangular, weakly incurved; third mandibular tooth distinctly shorter than apical tooth and teeth numbers two and four; masticatory margin of mandibles approximately vertical or weakly oblique. **Mesosoma.** Integument of pronotum, mesonotum and mesopleuron shining and mainly smooth, vestigial shagreenation most noticeable on humeri and mesopleuron; anterior mesosoma in profile smoothly rounded anteriad, thereafter pronotum and whole of mesonotum flattened and on same plane as propodeum; appearance of erect pronotal setae long (i.e., longest erect setae longer than length of eye) and unmodified; in profile, metanotal groove a narrow but deep slit; propodeum shining, with multiple hair like striolae, or shining and uniformly striolate; propodeum smoothly rounded or with indistinct angle; propodeal dorsum and declivity confluent; erect propodeal setae present and abundant (greater than 12); appressed propodeal setulae long and separated by at least own length; propodeal spiracle situated on or beside declivitous face of propodeum, and shorter (length < 0.50 × height of propodeum). **Petiole.** In profile, petiolar node squamiform; in full-face view, shape of petiolar node uniformly rounded; node shining and smooth throughout. **Gaster.** Gaster smooth and glossy; pilosity of first gastral tergite consisting wholly or mainly of long, curved setae, appressed setae apparently absent. **General characters.** Colour mainly foreparts russet with gaster blackish-brown; more rarely concolorous blackish-brown.

#### Major worker description.

**Head.** Head horizontally rectangular, broader than wide; posterior margin of head weakly concave; cuticle of frons shining and smooth except for piliferous pits and a few striolae around antennal insertion sand frontal carinae; pilosity of frons a mixture of well-spaced, distinctly longer erect and semi-erect setae interspersed with shorter decumbent setae. Eye moderate (eye length 0.20–0.49 length of head capsule); in full-face view, eyes set at about midpoint of head capsule; in profile, eye set anteriad of midline of head capsule; eyes elliptical. In full-face view, frontal carinae straight, divergent posteriad; frontal lobes straight in front of antennal insertion. Anterior clypeal margin broadly convex with anteromedial dimple; clypeal psammophore set at or above midpoint of clypeus; palp formula 6,4. Five mandibular teeth in major worker; mandibles triangular, weakly incurved; third mandibular tooth distinctly shorter than apical tooth and teeth numbers two and four, or absent; masticatory margin of mandibles approximately aligned vertically or weakly oblique. **Mesosoma.** Integument of pronotum, mesonotum and mesopleuron shining with indistinct microsculpture that is most pronounced on lower surfaces; anterior mesosoma in profile pronotum smoothly rounded anteriad and flattened posteriad, mesonotum narrowly convex; erect pronotal setae long (i.e., longer than length of eye) and unmodified; in profile, metanotal groove deep, V-shaped; propodeum shining and finely striolate and microreticulate; propodeum smoothly rounded or with indistinct angle, or angulate, propodeal angle blunt; length ratio of propodeal dorsum to its declivity between 1:1 and 1:2, or not applicable, propodeal dorsum and declivity confluent; erect propodeal setae present and abundant (at least a dozen); appressed propodeal setae long, each reaching setae behind and in front, but not forming pubescence; propodeal spiracle situated on or beside declivitous face of propodeum, and shorter (length less than 0.50 × height of propodeum). **Petiole.** In profile, petiolar node squamiform; in full-face view, shape of petiolar node uniformly rounded, or tapered with blunt vertex; node shining and faintly shagreenate-microreticulate. **Gaster.** Gaster shining with superficial microreticulation; pilosity of first gastral tergite consisting mainly of well-spaced short, semi-erect and decumbent setae. **General characters.** Colour of foreparts dark orange tan, gaster blackish-brown.

#### Measurements.

Worker (n = 6): CI 101–126; EI 19–38; EL 0.20–0.33; HL 0.51–1.39; HW 0.52–1.76; ML 0.71-.1.71; MTL 0.43–1.12; PpH 0.07–0.20; PpL 0.27–0.65; SI 63–118; SL 0.61–1.12.

#### Comments.

Unlike some related taxa, *Melophorus ankylochaetes* is easy to characterize as a shining and very hairy *Melophorus*, with many fine, flexuous setae on the body, curved erect and suberect setae only on the first gastral tergite and whorls of ‘*pillipes*’-type setae on the tibiae. Populations of the ant without the ‘*pillipes*’ condition may occur (given its position within the *M. fieldi* complex), but the other characters mentioned should still enable specimens to be recognized. Genetic sequencing strongly supports this species as a sister to *M. incisus* (which has a similar vestiture on the gaster) within the *M. fieldi* complex, but *M. ankylochaetes* does not have such deep sulci between the mesosomal anatomical regions (e.g., especially the anepisternum and katepisternum). *Melophorus ankylochaetes* has a broad distribution in the northern states (NSW, NT, QLD and WA) and probably also occurs in northern SA, but is unlikely to be found in Victoria or Tasmania. Ecological data are lacking, but the ant has been found in box-pine scrub over red soil (QLD) and foraging on the ground in mallee (WA).

#### Etymology.

Compound of Greek *ankylos* (‘crooked’) plus Greek *khaitē* ‘long hair’ (here, ‘bristle’); noun in the nominative plural standing in apposition to the generic name.

**Figure 52.**
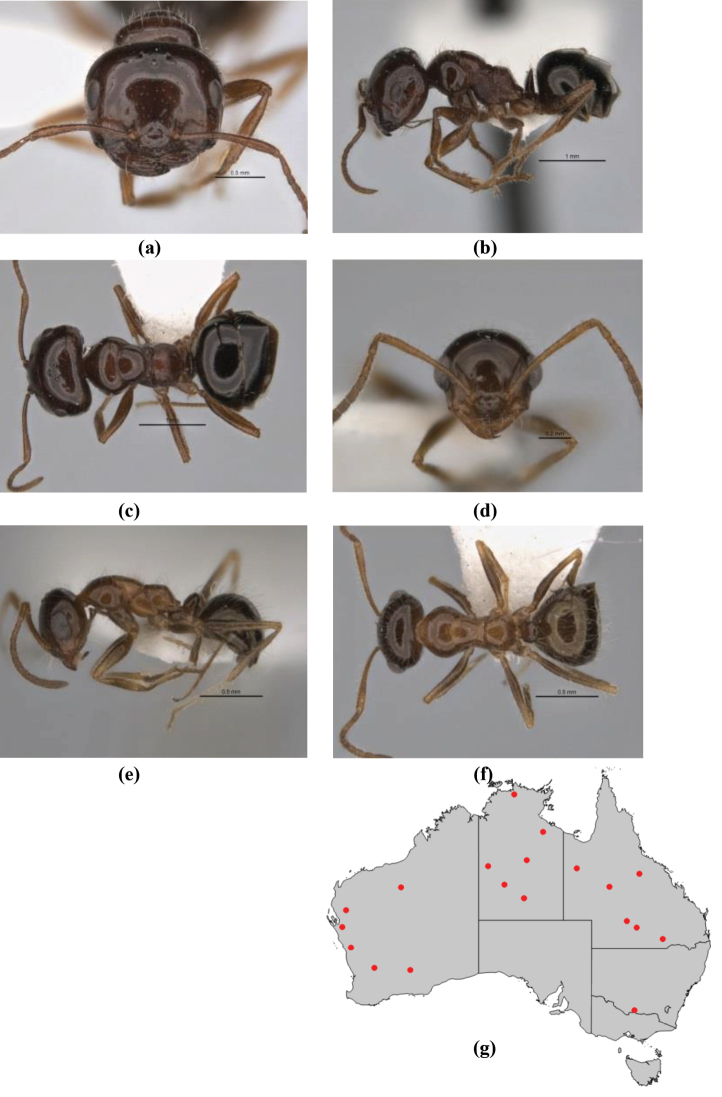
*Melophorus ankylochaetes* sp. n.: major worker paratype (JDM32-004761) frons (**a**), profile (**b**) and dorsum (**c**); minor worker holotype (JDM32-001990) frons (**d**), profile (**e**) and dorsum (**f**); distribution map for the species (**g**). Low resolution scale bars: 1 mm (**b, c**); 0.5 mm (**a, e, f**); 0.2 mm (**d**).

### Melophorus bruneus

Taxon classificationAnimaliaHymenopteraFormicidae

McAreavey

Melophorus (Melophorus) brunea McAreavey 1949: 20, figs 57–65.

#### Types.

Holotype minor worker carded with paratype major worker and queen on top card, and paratype major worker and paratype minor worker of bottom card, all on same pin, also paratype major, minor and media workers and queens on two other pins, Nyngan New South Wales [ANIC] (examined: ANIC specimens ANIC32-05344).

#### Other material examined.

**New South Wales**: 25 km ENE Fort Gray Homestead (Morton, S.R.), 40 mi N Warren (Lowery, B.B.), Bogan River (Armstrong, J.), Callubri Station, 2 mi from homestead (Greaves, T.), CSIRO Lake Mere Field Station, near Louth (Bryannah, M.), Fowlers Gap (Greenslade, P.J.M.), Fowlers Gap (Greenslade, P.J.M.), Kapunda, N Nyngan (Greenslade, P.J.M.), Sturt National Park (Greenslade, P.J.M.), Trundle (Lowery, B.B.). **Queensland**: ‘Gumbardo’ (Beutel, T.), ‘Merigol’ (Beutel, T.), 12 mi WSW Camel Creek Homestead, W Ingham (Dowse, J.E.), 22 mi E Condamine (Dowse, J.E.), 25 km SW Warwick (Greenslade, P.J.M.), 40 km E Cameron Corner (Greenslade, P.J.M.), 65 km E Birdsville (Forrest, J.), Eulo (Greenslade, P.J.M.), Illaweena St. Drewvale (QM Party), Redlands Hilliards Ck, nr South St (RHC1) (QM Party), St. George (Lowery, B.B.). **South Australia**: 15 km NE Umberatana, Flinders Ranges (Greenslade, P.J.M.), Eyre Hwy, 9.7 km NE Cootra (Heterick, B.E. [M324]), Lake Short Res. (Churchett, G. & R. [M87/M102]), Vokes Hill, Victoria Desert (Greenslade, P.J.M.). **Victoria**: 15 km WNW Yaapeet (Andersen, A.N.), Sea Lake (Goudie). **Western Australia**: 12 km S of Exmouth (Heterick, B.E. [JDM32-001781]), 64 km E of Southern Cross (Heterick, B.E. [JDM32-001784]), Sandstone Rd turnoff (Heterick, B.E. [M295]), Tropicana Minesite (Summerhayes, J. [JDM32-004538]).

#### Diagnosis.

*Melophorus bruneus* can be placed in the *M. biroi* species-group on the basis of characters of the clypeus, propodeum, mandible and palps. The species is also placed in the *M. fieldi* species-complex because of the appearance of the anteriorly placed clypeal psammophore, the compact propodeum, the presence of more than one preapical spine on the metatibia, at least in the major worker, the long, even spindly legs and the unmodified mandible in the major worker. *Melophorus bruneus* workers require careful checking to distinguish them from related species, particularly those of *M. turneri* and *M. fieldi*. Most *M. bruneus* workers can be distinguished from all taxa except *M. fieldi* by the following combination of characters: (1) eye relatively large (eye length 0.50× length of side of head capsule in minor worker, approximately 0.33× length of side of head capsule in major worker ≥), (2) in full-face view, periphery of upper frons surrounded to about the level of the eyes with short, bristly, erect setae that are often flattened distally; (3) minor worker small (HW ≈ 0.56-0.59 mm); (4) non-iridescent head of major worker relatively smooth and gleaming (e.g., Fig. 54(a)) and, (5) clypeal psammophore of fine setae placed at or about midpoint of clypeus. The minor worker of *Melophorus bruneus* is distinguished from that of *M. fieldi* by its brown or dark reddish-brown but not blackish-brown colour that lacks a dull, silky sheen, by its smoothly convex pronotum when seen in profile, by the longer, often flexuous erect setae on the mesosoma (length of longer setae ≥ greatest width of antennal scape) and, in relatively glabrous minor workers, the long and conspicuously pale appressed setae on the mesosoma. The major worker has short, sometimes modified setae that are far more numerous than those of the major worker of *M. fieldi* and these give a shaggy appearance to most majors; moreover, the appressed setae on gaster are longer than in *M. fieldi* and overlap. Some populations of *M. bruneus* have glabrous minor workers. These can be distinguished from related species (particularly *M. turneri* and *M. inconspicuus*) by their matt, microreticulate heads and the relatively long, pale, appressed setae on the gaster (similar species usually have erect setae on the mesosoma, they have smoother and more shining heads and they have short, appressed setae on the gaster).

#### Minor worker description.

**Head.** Head approximately oval with straight sides; posterior margin of head planar or weakly convex; frons matt or with weak sheen, microreticulate or microreticulate-shagreenate; pilosity of frons a mixture of a few well-spaced, erect setae interspersed with appressed setae only. Eye large (eye length ≥ 0.50 × length of side of head capsule); in full-face view, eyes set at about midpoint of head capsule; in profile, eye set around midline of head capsule; eyes elliptical or slightly reniform. In full-face view, frontal carinae straight or weakly convex; frontal lobes curved toward antennal insertion. Anteromedial clypeal margin broadly and evenly convex; clypeal psammophore set at or above midpoint of clypeus; palp formula 6,4. Five to six mandibular teeth in minor worker; mandibles narrow, strap-like, internal and external margins parallel or nearly so; third mandibular tooth distinctly shorter than apical tooth and teeth numbers two and four; masticatory margin of mandibles approximately vertical or weakly oblique. **Mesosoma.** Integument of pronotum, mesonotum and mesopleuron shining and microreticulate, microreticulation reduced on humeri, or with weak to moderate sheen and superficial microreticulation (more pronounced on mesopleuron); anterior mesosoma in profile broadly convex; appearance of erect pronotal setae short, (i.e., longest erect setae shorter than length of eye) and unmodified, or short and often expanded distally, at times clavate; in profile, metanotal groove shallow, broadly V or U-shaped; propodeum matt or with a weak sheen and microreticulate; propodeum angulate, propodeal angle blunt; length ratio of propodeal dorsum to its declivity between 1:1 and 1:2; erect propodeal setae present and abundant (greater than 12); appressed propodeal setulae long and closely aligned, creating pubescence; propodeal spiracle situated nearer to midpoint of propodeum than to its declivitous face, and shorter (length < than 0.50 × height of propodeum). **Petiole.** In profile, petiolar node squamiform; in full-face view, shape of petiolar node uniformly rounded; node shining and distinctly shagreenate-microreticulate. **Gaster.** Gaster weakly shining with indistinct shagreenation; pilosity of first gastral tergite consisting of thick, appressed setae that form pubescence, interspersed with numerous short, bristly, erect setae. **General characters.** Colour brown, gaster (and often head) darker than mesosoma.

#### Major worker description.

**Head.** Head square; posterior margin of head planar or weakly convex; cuticle of frons matt or with weak sheen, indistinctly shagreenate; pilosity of frons a mixture of a few well-spaced, erect setae interspersed with appressed setae only. Eye moderate (eye length 0.20–0.49 length of head capsule); in full-face view, eyes set at about midpoint of head capsule; in profile, eye set anteriad of midline of head capsule; eyes elliptical. In full-face view, frontal carinae straight, divergent posteriad; frontal lobes straight in front of antennal insertion. Anterior clypeal margin broadly convex with anteromedial dimple; clypeal psammophore set at or above midpoint of clypeus; palp formula 6,4. Five mandibular teeth in major worker; mandibles narrow, strap-like, internal and external borders parallel or nearly so; third mandibular tooth distinctly shorter than apical tooth and teeth numbers two and 4; masticatory margin of mandibles approximately aligned vertically or weakly oblique. **Mesosoma.** Integument of pronotum, mesonotum and mesopleuron matt with indistinct shagreenate sculpture throughout; anterior mesosoma in profile broadly convex; erect pronotal setae short, (i.e., shorter than length of eye) and unmodified, or short and often expanded distally, at times clavate; in profile, metanotal groove shallow, broadly V- or U-shaped; propodeum shining and shagreenate; propodeum angulate, propodeal angle blunt; length ratio of propodeal dorsum to its declivity between 1:1 and 1:2; erect propodeal setae present and abundant (at least a dozen); appressed propodeal setae long and separated by at least own length; propodeal spiracle situated at least twice its width from the declivitous face of propodeum, and shorter (length less than 0.50 × height of propodeum), or situated on or beside declivitous face of propodeum, and shorter (length less than 0.50 × height of propodeum). **Petiole.** In profile, petiolar node squamiform; in full-face view, shape of petiolar node tapered with blunt vertex; node shining and faintly shagreenate-microreticulate. **Gaster.** Gaster shining, shagreenate (‘LP record’ appearance); pilosity of first gastral tergite consisting of well-spaced, erect and semi-erect setae interspersed with regularly spaced appressed setae. **General characters.** Colour of head and foreparts brownish-orange to brown with darker brown to blackish gaster.

#### Measurements.

Worker (n = 8): CI 103–127; EI 24–41; EL 0.24–0.37; HL 0.57–1.23; HW 0.59–1.55; ML 0.78–1.55; MTL 0.48–0.94; PpH 0.08–0.14; PpL 0.31–0.64; SI 64–108; SL 0.64–0.99.

#### Comments.

Major workers of *Melophorus bruneus* can be mistaken for the large-eyed major workers of some *Melophorus turneri*, so careful attention needs to be given to the circlet of short, stout setae on the vertex of the head capsule and the numerous, short, curved, erect setae on the head and mesosoma in the former. Most minor workers are distinctive with their vestiture of long, flexuous setae, but some populations in the NT and SA lack such setae. In these cases the matt, microreticulate nature of the frons and long appressed setae on the mesosoma are diagnostic, except for separation from *M. fieldi*. Minor and major workers of *M. fieldi* and *M. bruneus* can be separated based on different degrees of pilosity. While material has been available for sequencing, obtaining molecular data has proven difficult. The position of *M. bruneus* in the three-gene tree (Suppl. material 1) suggests a close relationship with *M. fulvidus*. However, this is counterindicated by evidence of likely hybridization with *M. turneri* (well-separated from *M. bruneus* on the three-gene tree). The evolutionary position of *M. bruneus* within the *M. fieldi* complex must therefore be considered uncertain.

This species is widely distributed in all mainland Australian states, but is more common in drier, inland areas. Pitfall traps have accounted for most specimens, but some have been hand-collected. Label data variously record savanna woodland, *Callitris*, mallee, scribbly gum and heath and ‘pure *Eucalyptus dum* (*osa*)’ habitat and there are two records of red soil. One specimen was collected in a parking bay. In all likelihood this a generalist scavenger of plant and animal matter like many other members of the *M. fieldi* complex. Minor workers collected by the principal author north of Wiluna, WA, in relatively cool conditions (for a *Melophorus*) emerged timidly from a single, simple nest hole in red, lateritic soil with many surface ironstone pebbles. There is no other mention of the ecology of the ant from the labels, and neither is there any other information from McAreavey in his description of the taxon.

**Figure 53.**
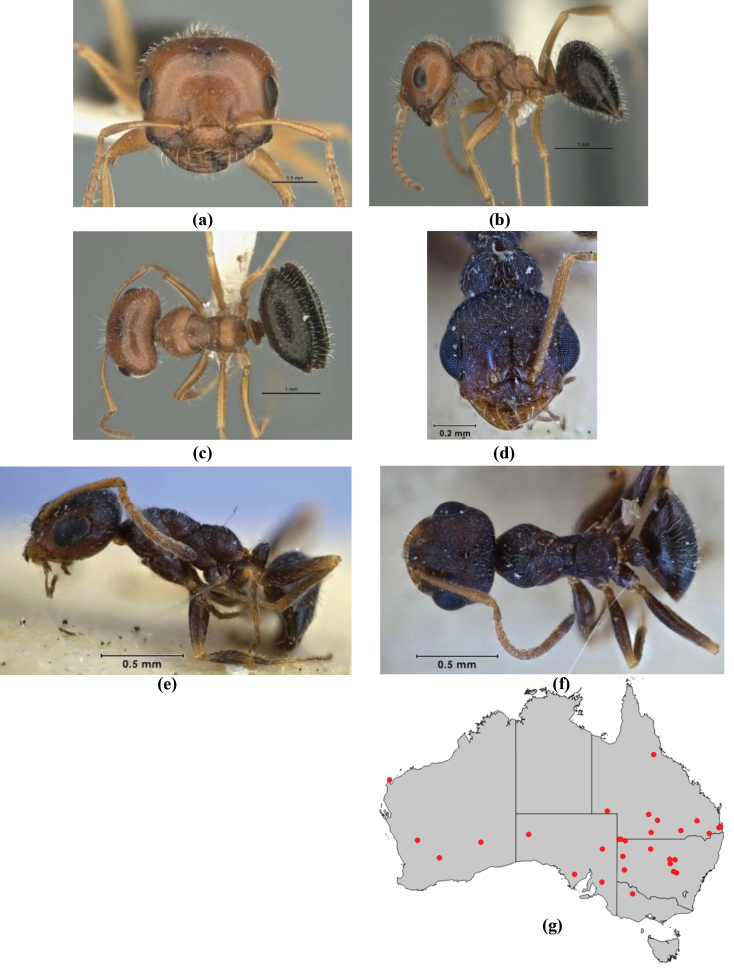
*Melophorus bruneus* McAreavey: non–type major worker (ANIC32-900218) frons (**a**), profile (**b**) and dorsum (**c**); minor worker paratype (ANIC32-053433–top card) frons (**d**), profile (**e**) and dorsum (**f**); distribution map for the species (**g**). Low resolution scale bars: 1 mm (**b, c**); 0.5 mm (**a**).

### Melophorus eumorphus

Taxon classificationAnimaliaHymenopteraFormicidae

Heterick, Castalanelli & Shattuck sp. n.

http://zoobank.org/C526D4D2-1073-46C8-B0C5-ECF509D85501

#### Types.

Holotype minor worker (bottom ant) from 53 km E Vokes Hill, Victoria Desert, South Australia, 9 October 1976, P.J.M. Greenslade [ANIC32-900173] (ANIC). Paratypes: 2 minor workers on same pin and with same details as holotype (ANIC); 2 minor workers from Cambrai, South Australia, 24-28 January 1972, P.J.M. Greenslade (ANIC); minor worker from Koonamore, South Australia, 25 February 1972, P.J.M. Greenslade, (5) (BMNH); 2 minor workers from Observatory Hill, Victoria Desert, South Australia, 7 October 1972, P.J.M. Greenslade, (4), 26) (SAM); major and media worker from intersection of Holland track and Norseman Road 32°24'51"S, 119°27'33"E, Western Australia, 2 January 2006, B.E. Heterick, sandplain/laterite heathland, foraging on ground [JDM32-001981] (WAM); minor worker from 64 km E of Southern Cross, Western Australia, 18 April, 1987, B. Heterick, soil, native vegetation, rural environment, 245, 8*Mel*BH26, [JDM32-001982] (WAM).

#### Other material examined.

**New South Wales**: CSIRO Lake Mere Field Station, near Louth (Bryannah, M.). **South Australia**: Cambrai (Greenslade, P.J.M.), Cambrai (Greenslade, P.J.M. [ANIC32-900102]), Cambrai (Greenslade, P.J.M.), Cambrai (Greenslade, P.J.M.), Koonamore (Greenslade, P.J.M.), Koonamore (Greenslade, P.J.M.). **Western Australia**: 1 km W Canna (Heterick, B.E. [M320]).

#### Diagnosis.

*Melophorus eumorphus* can be placed in the *M. biroi* species-group on the basis of characters of the clypeus, propodeum, mandible and palps. The species is also placed in the *M. fieldi* species-complex because of the appearance of the anteriorly placed clypeal psammophore, the compact propodeum, the presence of more than one preapical spine on the metatibia, at least in the major worker, the long, even spindly legs and the unmodified mandible in the major worker. The appressed setae on the gaster in all workers of *M. eumorphus* are very small and inconspicuous when the gaster is moderately distended, and are separated from one another by at least their own length, these appressed setae also being inconspicuous on the mesosoma and never long and silvery. The mesosoma is glabrous in the minor worker or has one or a few flexuous, erect setae. The node of the minor worker is often squamiform and most commonly the cuticle is shining or even glossy with vestigial or weak shagreenation. These features serve to differentiate *M. eumorphus* from most of the members of its complex. This ant may be distinguished from the few remaining similar species (notably *M. longipes*, *M. turneri* and *M. vitreus*) by the following characters: the minor worker is very small (HW < 0.55 mm), the frons of the head capsule is smooth and shining, the eye is large (eye length approximately 0.40 × length of head capsule), the mesopleuron has distinct, wrinkled or scalloped sculpture that may extend to the mesonotum (this is a particularly good feature to help distinguish this ant from the similar-sized *M. vitreus*) and the propodeum is a rounded cube. The major worker is also very small (HW ≤ 0.73 mm) with a large eye (eye length approximately 0.38 × length of head capsule) and the mesopleural sculpture as for the minor worker

#### Minor worker description.

**Head.** Head approximately oval with straight sides; posterior margin of head strongly convex; frons shining and smooth except for piliferous pits; frons consisting exclusively or almost exclusively of well-spaced, appressed setae only (small, erect setae, if present, usually confined to ocular triangle or posterior margin of head). Eye large (eye length ≥ 0.50 × length of side of head capsule), or moderate (eye length 0.20–0.49 length of side of head capsule); in full-face view, eyes set at about midpoint of head capsule; in profile, eye set around midline of head capsule; eyes elliptical or slightly reniform. In full-face view, frontal carinae distinctly concave; frontal lobes curved toward antennal insertion. In full-face view, anteromedial clypeal margin broadly convex with anteromedial dimple; clypeal psammophore set below midpoint of clypeus; palp formula 6,4. Five mandibular teeth in minor worker; mandibles triangular, weakly incurved; third mandibular tooth distinctly shorter than apical tooth and teeth numbers two and four; masticatory margin of mandibles approximately vertical or weakly oblique. **Mesosoma.** Integument of pronotum, mesonotum and mesopleuron shining and smooth on dorsum, entire lower mesopleuron distinctly striolate-microreticulate; anterior mesosoma in profile broadly convex; appearance of erect pronotal setae long (i.e., longest erect setae longer than length of eye) and unmodified, or erect pronotal setae absent; in profile, metanotal groove deep, ‘V’-shaped; propodeum shining, with multiple hair like striolae; propodeum smoothly rounded or with indistinct angle; propodeal dorsum and declivity confluent; erect propodeal setae always absent; appressed propodeal setulae long, each reaching setae behind and in front, but not forming pubescence; propodeal spiracle situated on or beside declivitous face of propodeum, and shorter (length < 0.50 × height of propodeum). **Petiole.** In profile, petiolar node subcuboidal, vertex bluntly rounded; in full-face view, shape of petiolar node square with rounded angles; node shining and smooth throughout. **Gaster.** Gaster smooth and glossy; pilosity of first gastral tergite consisting of well-spaced, erect and semi-erect setae interspersed with regularly placed appressed setae, or consisting of well-spaced short, inconspicuous, appressed setae only, erect setae always absent. **General characters.** Colour of head and gaster chocolate, mesosoma tan.

#### Major worker description.

**Head.** Head square; posterior margin of head planar or weakly convex; cuticle of frons shining and smooth except for piliferous pits; frons consisting exclusively or almost exclusively of well-spaced, appressed setae only (small, erect setae, if present, usually confined to ocular triangle or posterior margin of head). Eye moderate (eye length 0.20–0.49 length of head capsule); in full-face view, eyes set at about midpoint of head capsule; in profile, eye set anteriad of midline of head capsule; eyes elliptical. In full-face view, frontal carinae straight, divergent posteriad; frontal lobes straight in front of antennal insertion. Anterior clypeal margin broadly convex with anteromedial dimple; clypeal psammophore set at or above midpoint of clypeus; palp formula 6,4. Five mandibular teeth in major worker; mandibles triangular, weakly incurved; third mandibular tooth distinctly shorter than apical tooth and teeth numbers two and four; masticatory margin of mandibles approximately aligned vertically or weakly oblique. **Mesosoma.** Integument of pronotum, mesonotum and mesopleuron shining with very superficial microreticulation, entire lower mesopleuron distinctly shagreenate; anterior mesosoma in profile pronotum smoothly rounded anteriad and flattened posteriad, mesonotum narrowly convex; erect pronotal setae long (i.e., longer than length of eye) and unmodified; in profile, metanotal groove deep, V-shaped; propodeum shining, dorsum and declivitous face of propodeum mainly smooth, but with weak to strong vertical striolae arising from metapleuron; propodeum smoothly rounded or with indistinct angle; propodeal dorsum and declivity confluent; erect propodeal setae present and sparse to moderate (1-12); appressed propodeal setae long and separated by at least own length; propodeal spiracle situated on or beside declivitous face of propodeum, and shorter (length less than 0.50 × height of propodeum). **Petiole.** In profile, petiolar node squamiform; in full-face view, shape of petiolar node uniformly rounded; node shining and smooth throughout. **Gaster.** Gaster shining, shagreenate (‘LP record’ appearance); pilosity of first gastral tergite consisting of well-spaced, erect and semi-erect setae interspersed with regularly spaced appressed setae. **General characters.** Colour as for minor worker.

#### Measurements.

Worker (n = 6): CI 105–110; EI 26–33; EL 0.16–0.21; HL 0.46–0.73; HW 0.49–0.73; ML 0.64–0.91; MTL 0.38–0.56; PpH 0.07–0.11; PpL 0.23–0.33; SI 91–105; SL 0.51–0.73.

#### Comments.

*Melophorus eumorphus* is a small, shining species that appears to be widespread in arid and semi-arid habitats in temperate Australia (in NSW, SA and WA). The ant can be separated from similar species in its clade by a combination of its bimodal mesosoma when seen in profile, its scalloped mesopleuron, its total or almost total lack of erect setae on the mesosoma and its thick, knob-like petiolar node. Superficially, it is similar in appearance to several members of the *M. ludius* complex, but has the spindly legs, the posteriorly placed clypeal psammophore and the long maxillary palps that are typical of many of the members of the *M. fieldi* complex. One WA specimen has been genetically sequenced. On a five-gene tree this species is linked to *M. perthensis*; however a close evolutionary relationship between the pair does not have strong branch support. The ant is sister to *M. longipes* on a three-gene tree but, again, branch support is relatively weak. There are little ecological data on the species: the sequenced worker from Canna, WA, was collected in mallee woodland over red loam, while five samples were taken from dunes in Cambrai, SA and a single worker was taken from a paddock at the Lake Mere Field Station in NSW.

#### Etymology.

Greek *eu* (‘well’, ‘good’) plus Latinized Greek *morphus* (Greek *morphé* ‘shape’, ‘form’); adjective in the nominative singular.

**Figure 54.**
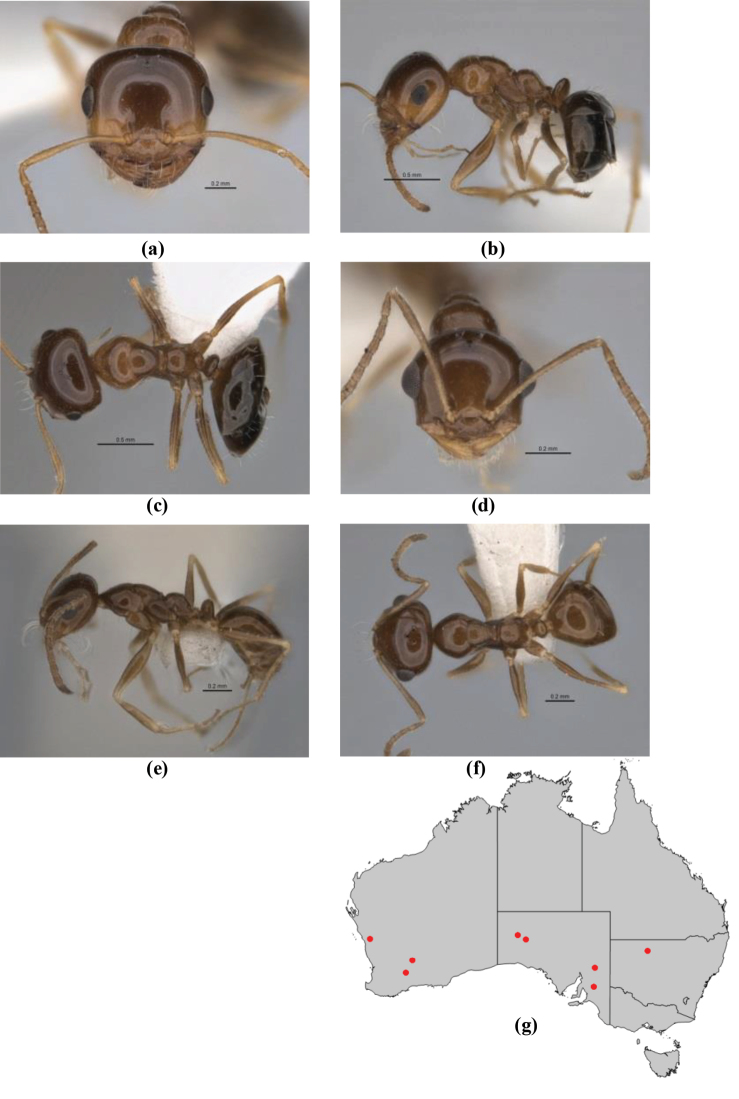
*Melophorus eumorphus* sp. n.: major worker paratype (JDM32-001981–top ant) frons (**a**), profile (**b**) and dorsum (**c**); minor worker holotype (ANI32-900173–bottom ant) frons (**d**), profile (**e**) and dorsum (**f**); distribution map for the species (**g**). Low resolution scale bars: 0.5 mm (**b, c**); 0.2 mm (**a, d–f**).

### Melophorus fieldi

Taxon classificationAnimaliaHymenopteraFormicidae

Forel

Melophorus fieldi Forel 1910: 62 (combination in M. (Erimelophorus) by Wheeler 1935: 71). Type. Holotype major worker, Tennants [*sic*] Creek, Northern Territory [MHNG] examined.

#### Other material examined.

**New South Wales**: Mundi Mundi, near Broken Hill (Valentine, J.). **Northern Territory**: 14 mi NNE Narwietooma Homestead (McInnes & Dowse), 17 km S Rabbit Flat (Greenslade, P.J.M.). **South Australia**: 53 km E Vokes Hill, Victoria Desert (Greenslade, P.J.M.), Blyth (Lowery, B.B.), Blyth, N Adelaide (Lowery, B.B.), Brookfield (Greenslade, P.J.M.), Cambrai (Greenslade, P.J.M. [ANIC32-900060]), Koonamore, 5 km NE homestead (Greenslade, P.J.M.). **Western Australia**: 1 km W Canna (Heterick, B.E. [M316]), 15.2 km S Koolyanobbing (Heterick, B.E. [M02/M31/M32]), Black Swan Mine (Langlands, P. & Osbourne, J. [JDM32-001489]), Mt Crawford (Heterick, B.E. [JDM32-004669]), Westonia (Harris, R., *et.al.* [JDM32-001490]), Woolgangie (Heterick, B.E. [JDM32-001488]).

#### Diagnosis.

*Melophorus fieldi* can be placed in the *M. biroi* species-group on the basis of characters of the clypeus, propodeum, mandible and palps. The species is also placed in the *M. fieldi* species-complex because of the appearance of the anteriorly placed clypeal psammophore, the compact propodeum, the presence of more than one preapical spine on the metatibia, at least in the major worker, the long, even spindly legs and the unmodified mandible in the major worker. *Melophorus fieldi* workers require careful checking to distinguish them from related species, particularly those of *M. turneri* and *M. bruneus*. Most *M. fieldi* workers can be distinguished from all taxa except *M. bruneus* by the following combination of characters: (1) eye relatively large (eye length 0.50× length of side of head capsule in minor worker, approximately 0.33× length of side of head capsule in major worker ≥), (2) in full-face view, periphery of upper frons surrounded to about the level of the eyes with short, bristly, erect setae that are often flattened distally; (3) minor worker small (HW ≈ 0.56-0.59 mm); (4) non-iridescent head of major worker relatively smooth and gleaming (e.g., Fig. 54(a)) and, (5) clypeal psammophore of fine setae placed at or about midpoint of clypeus. The minor worker of *M. fieldi* is a concolorous blackish-brown, finely shagreenate with a dull silky sheen, while, in profile, the pronotum rises gradually to its junction with the mesonotum without any obvious convexity and the mesonotum is slightly humped (see under the diagnosis for *M. bruneus* for differences between *M. fieldi* and that species in these particulars). The mesosoma has a few to many short, straight, bristly setae, these often expanded distally, the length of the longest setae ≤ greatest length of antennal scape, and the appressed setae are short and inconspicuous. The major and media worker and media workers are concolorous brown (the holotype is an orange-yellow with a brown gaster), with scattered short, sometimes modified setae and the appressed setae on the gaster are well-separated and do not overlap (unlike the case with *M. bruneus*).

#### Minor worker description.

**Head.** Head square; posterior margin of head weakly convex; frons matt or with weak sheen, shagreenate; pilosity of frons a mixture of a few well-spaced, erect setae interspersed with appressed setae only. Eye large (eye length ≥ 0.50 × length of side of head capsule); in full-face view, eyes set at about midpoint of head capsule; in profile, eye set around midline of head capsule; roughly ovoid, eye narrowed posteriad. In full-face view, frontal carinae straight, divergent posteriad; frontal lobes curved toward antennal insertion. Anteromedial clypeal margin broadly and evenly convex; clypeal psammophore set at or above midpoint of clypeus; palp formula 6,4. Mandibular teeth in minor worker four to five; mandibles narrow, strap-like, internal and external margins parallel or nearly so; third mandibular tooth distinctly shorter than apical tooth and teeth numbers two and four; masticatory margin of mandibles approximately vertical or weakly oblique. **Mesosoma.** Integument of pronotum, mesonotum and mesopleuron moderately shining and shagreenate throughout; anterior mesosoma in profile broadly convex; appearance of erect pronotal setae short, (i.e., longest erect setae shorter than length of eye) and unmodified; in profile, metanotal groove shallow, indicated mainly by an angle; propodeum shining and shagreenate; propodeum angulate, propodeal angle blunt; length ratio of propodeal dorsum to its declivity about1:1; erect propodeal setae present and sparse to moderate (1-12); appressed propodeal setulae short, separated by more than own length and inconspicuous; propodeal spiracle situated at least twice its width from the declivitous face of propodeum, and shorter (length < 0.50 × height of propodeum). **Petiole.** In profile, petiolar node squamiform; in full-face view, shape of petiolar node tapered with blunt vertex; node shining and distinctly shagreenate-microreticulate. **Gaster.** Gaster weakly shining with indistinct shagreenation, or shining, shagreenate (‘LP record’ appearance); pilosity of first gastral tergite consisting of well-spaced, erect and semi-erect setae interspersed with regularly placed appressed setae. **General characters.** Colour dark chocolate.

#### Major worker description.

**Head.** Head horizontally rectangular, broader than wide; posterior margin of head planar or weakly convex; cuticle of frons shining with superficial shagreenation or microreticulation only; pilosity of frons a mixture of a few well-spaced, erect setae interspersed with appressed setae only. Eye moderate (eye length 0.20–0.49 length of head capsule); in full-face view, eyes set at about midpoint of head capsule; in profile, eye set anteriad of midline of head capsule; roughly ovoid, eye narrowed posteriad. In full-face view, frontal carinae straight or weakly convex; frontal lobes curved inward in front of antennal insertion. Anterior clypeal margin broadly and evenly convex; clypeal psammophore set at or above midpoint of clypeus; palp formula 6,4. Five mandibular teeth in major worker; mandibles narrow, strap-like, internal and external borders parallel or nearly so; third mandibular tooth distinctly shorter than apical tooth and teeth numbers two and four; masticatory margin of mandibles approximately aligned vertically or weakly oblique. **Mesosoma.** Integument of pronotum, mesonotum and mesopleuron matt or with weak sheen and microreticulate throughout; anterior mesosoma in profile broadly convex; erect pronotal setae short, (i.e., shorter than length of eye) and unmodified; in profile, metanotal groove shallow, broadly V- or U-shaped; propodeum shining and shagreenate; propodeum smoothly rounded or with indistinct angle; propodeal dorsum and declivity confluent; erect propodeal setae present and abundant (at least a dozen); appressed propodeal setae short, separated by more than own length and inconspicuous; propodeal spiracle situated nearer to midpoint of propodeum than to its declivitous face, and shorter (length less than 0.50 × height of propodeum). **Petiole.** In profile, petiolar node squamiform; in full-face view, shape of petiolar node tapered with blunt vertex; node shining and faintly shagreenate-microreticulate. **Gaster.** Gaster shining, shagreenate (‘LP record’ appearance); pilosity of first gastral tergite consisting of well-spaced, short, thick, erect setae interspersed with minute, appressed setae. **General characters.** Colour brown, lower frons and appendages generally pale brownish-yellow (however, the holotype has bright orange-yellow fore-parts and brown gaster).

#### Measurements.

Worker (n = 6): CI 105–115; EI 26–42; EL 0.24–0.31; HL 0.54–1.03; HW 0.56–1.19; ML 0.79–1.32; MTL 0.56–0.84; PpH 0.09–0.13; PpL 0.33–0.56; SI 77–127; SL 0.71–0.92.

#### Comments.

What we here determine to be major workers of *Melophorus fieldi* are difficult to distinguish from the major workers of *Melophorus bruneus*, with which they share the same features (e.g., large eyes, short antennal scapes and circlet of short, stout setae on the vertex). The degree of pilosity, however, differs for the two taxa, with *M. bruneus* being more hirsute. The minor workers have a characteristic *facies*, with very large, bulging eyes, black, matt, highly microreticulate mesosoma with a silky sheen and a few scattered short, erect setae on the dorsum of the mesosoma. Specimens have been collected from NSW, NT, SA and WA.

Mention must be made here of the strikingly different colour of the holotype, which is only slightly paler than syntypes of *M. turneri aesopus*, collected from the same area at the same time as *M. fieldi* by Field. The holotype, a major worker, is bright orange-yellow with a brown gaster, compared with the uniformly chocolate colouration of normal *M. fieldi* major workers. Possibly, genuine *M. fieldi* represents a third species, unrepresented among material we have seen and are ascribing to that taxon; but against this is the fact that all other features of the non-types appear to conform to the description of the species (the ant is not identical with the often lighter-coloured *M. bruneus* because the erect setae are sparse on the mesosoma and the appressed setae on the gaster do not overlap). Other *Melophorus* species can also be very colour-variable. Forel mentions that the gaster on this specimen was distended, but no mention is made in the description of whether the ant was collected while foraging or from the nest. If collected from the nest, one would expect other workers to have also been collected. However, if collected from near a nest this ant may be a teneral (although the specimen looks sturdy enough). Having said all this, additional material of the light-coloured morph from the type locality, especially if minor workers are also collected, may require the taxonomy of *M. fieldi* to be revisited.

As with *Melophorus bruneus*, sequencing, even of fresh nest material, has produced poor results for this species. The ant clusters with *M. parvimolaris* on a three-gene tree, but this placement is undoubtedly factitious. Three workers were collected in mallee scrub at Blyth, near Adelaide, SA and a worker taken at Broken Hill was collected near a creek. In WA, specimens from the north-eastern wheatbelt have been collected in red loamy soil in mallee and in dry sclerophyll woodland. In all likelihood this species, like *M. bruneus*, is a generalist scavenger of animal and plant materials in semi-arid woodlands throughout its range.

**Figure 55.**
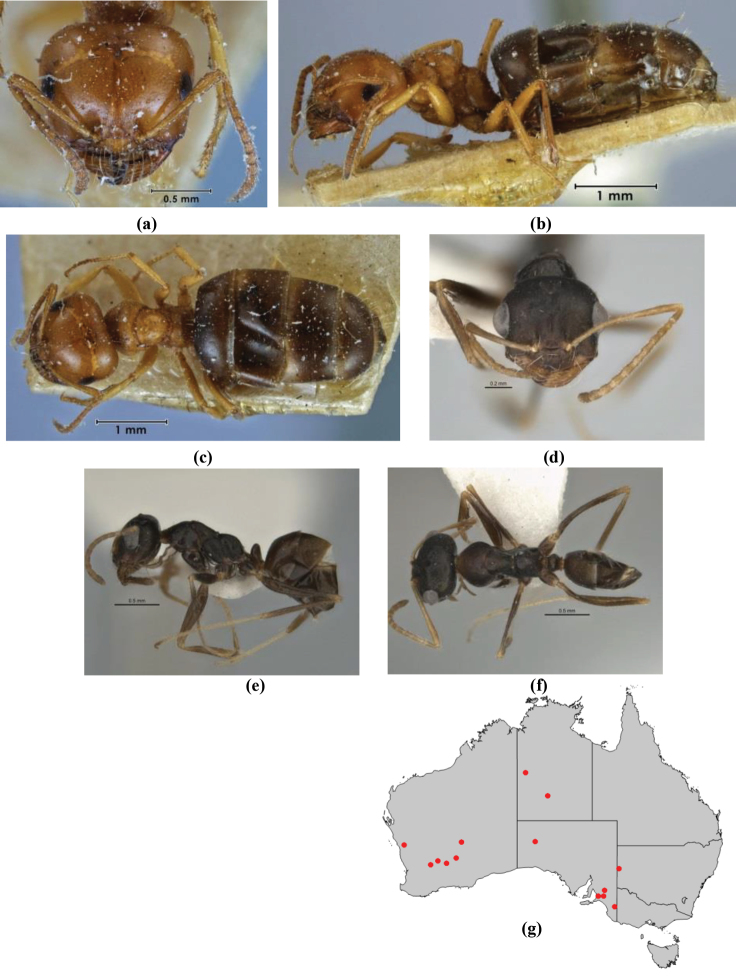
*Melophorus fieldi* Forel: MHNG major worker syntype frons (**a**), profile (**b**) and dorsum (**c**); non–type minor worker (JDM32-001489) frons (**d**), profile (**e**) and dorsum (**f**); distribution map for the species (**g**). Low resolution scale bars: 0.5 mm (**e, f**); 0.2 mm (**d**).

### Melophorus fulvidus

Taxon classificationAnimaliaHymenopteraFormicidae

Heterick, Castalanelli & Shattuck sp. n.

http://zoobank.org/59DA28A7-ECAF-4741-9978-8A5305C2492E

#### Types.

Holotype minor worker (top ant), from St George, Queensland, 14 January 1965 3.30 pm, B.B. Lowery, M., R154, red soil, box pine scrub, 104°, foraging, very fast, (other side of label) nest a small dome [ANIC32-900119] (ANIC). Paratypes: Major worker, 2 media workers and 2 minor workers on same pine and with same details as holotype (ANIC); 2 minor workers from Manbulloo, SW of Katherine, Northern Territory, 7-11 April 1978, P.J.M. Greenslade, BB, 17) [ANIC32-900118] (MCZ).

#### Other material examined.

**Queensland**: ‘Gumbardo’ (Beutel, T.), ‘Merigol’ (Beutel, T.), St. George (Lowery, B.B. [ANIC32-900119]). **Western Australia**: 3 km E Fitzroy Crossing (Heterick, B.E. [M197]), Ethel Creek (Varris, P.A. [JDM32-004579]).

#### Diagnosis.

*Melophorus fulvidus* can be placed in the *M. biroi* species-group on the basis of characters of the clypeus, propodeum, mandible and palps. This species is also placed in the *M. fieldi* species-complex because of the appearance of the anteriorly placed clypeal psammophore, the compact propodeum, the presence of more than one preapical spine on the metatibia, at least in the major worker, the long, even spindly legs and the unmodified mandible in the major worker. *Melophorus fulvidus* is a relatively distinctive species. The Major, media and minor workers are uniformly microreticulate and, in profile, the petiolar node of the minor and media worker is thick and tuberculate in shape, while that of the major worker is squamiform. The metatibial apical spur is absent in this species. The mesosoma of the minor and media workers is matt and glabrous, while that of the major worker has around 12 short, bristly, erect setae. These features, combined with the distinctive colour (minor and media workers are orange, the major worker orange with a brown gaster), will serve to identify the species.

#### Minor worker description.

**Head.** Head square; posterior margin of head planar or weakly convex; frons matt or with weak sheen, microreticulate or microreticulate-shagreenate; frons consisting exclusively or almost exclusively of well-spaced, appressed setae only (small, erect setae, if present, usually confined to ocular triangle or posterior margin of head). Eye moderate (eye length 0.20–0.49 length of side of head capsule); in full-face view, eyes set at about midpoint of head capsule; in profile, eye set anteriad of midline of head capsule; eyes elliptical or slightly reniform. In full-face view, frontal carinae distinctly concave; frontal lobes straight in front of antennal insertion. Anteromedial clypeal margin straight; clypeal psammophore set at or above midpoint of clypeus; palp formula 6,4. Five mandibular teeth in minor worker; mandibles triangular, weakly incurved; third mandibular tooth distinctly shorter than apical tooth and teeth numbers two and four; masticatory margin of mandibles approximately vertical or weakly oblique. **Mesosoma.** Integument of pronotum, mesonotum and mesopleuron matt or with weak sheen and microreticulate throughout; anterior mesosoma in profile broadly convex; appearance of erect pronotal setae long (i.e., longest erect setae longer than length of eye) and unmodified; in profile, metanotal groove shallow, indicated mainly by an angle; propodeum matt or with a weak sheen and microreticulate; propodeum angulate, propodeal angle blunt; length ratio of propodeal dorsum to its declivity about 4:3; erect propodeal setae always absent; appressed propodeal setulae sparse or absent, if present then not regularly spaced propodeal spiracle situated at least twice its width from the declivitous face of propodeum, and shorter (length < 0.50 × height of propodeum). **Petiole.** In profile, petiolar node cuboidal, rounded above; in full-face view, shape of petiolar node square with rounded angles; node matt and microreticulate. **Gaster.** Gaster matt with distinct microreticulate pattern; pilosity of first gastral tergite consisting of well-spaced short, inconspicuous, appressed setae only, erect setae always absent. **General characters.** Colour concolorous dusky light orange.

#### Major worker description.

**Head.** Head square; posterior margin of head planar or weakly convex; cuticle of frons matt or with weak sheen, microreticulate; frons consisting mainly of appressed and stout erect setae, the latter bristly in appearance and distinctly modified (flattened) distally. Eye moderate (eye length 0.20–0.49 length of head capsule); in full-face view, eyes set at about midpoint of head capsule; in profile, eye set anteriad of midline of head capsule; eyes elliptical. In full-face view, frontal carinae distinctly concave; frontal lobes straight in front of antennal insertion. Anterior clypeal margin broadly and evenly convex; clypeal psammophore set below midpoint of clypeus; palp formula 6,4. Five mandibular teeth in major worker, or 4-5; mandibles triangular, weakly incurved; third mandibular tooth distinctly shorter than apical tooth and teeth numbers two and four, or absent; masticatory margin of mandibles approximately aligned vertically or weakly oblique. **Mesosoma.** Integument of pronotum, mesonotum and mesopleuron matt or with weak sheen and microreticulate throughout; anterior mesosoma in profile broadly convex; erect pronotal setae short and often expanded distally, at times clavate; in profile, metanotal groove shallow, indicated mainly by an angle and metathoracic spiracles; propodeum matt or with a weak sheen and microreticulate; propodeum angulate, propodeal angle blunt; length ratio of propodeal dorsum to its declivity between 1:1 and 1:2; erect propodeal setae present and sparse to moderate (1-12); appressed propodeal setae sparse or absent, if present then not regularly spaced; propodeal spiracle situated on or beside declivitous face of propodeum, and shorter (length less than 0.50 × height of propodeum). **Petiole.** In profile, petiolar node squamiform; in full-face view, shape of petiolar node uniformly rounded; node matt, and microreticulate. **Gaster.** Gaster weakly shining with indistinct shagreenation; pilosity of first gastral tergite consisting of thick, often distally flattened, erect setae over well-spaced, short, appressed setae. **General characters.** Colour of foreparts orange, gaster light orange-brown.

#### Measurements.

Worker (n = 6): CI 98–113; EI 26–37; EL 0.17–0.24; HL 0.47–0.83; HW 0.46—0.94; ML 0.68–1.14; MTL 0.46–0.66; PpH 0.08–0.12; PpL 0.30–0.50; SI 81–132; SL 0.61–0.77.

#### Comments.

Rather surprisingly, this species in TERC is placed by Andersen in his ‘*fulvihirtus*’ group (as ‘species 21’), despite its morphological similarity to similarly-coloured *M. fieldi* complex members. Limited sequencing (just one specimen) clearly places it within the *M. fieldi* complex. However, its closest relative remains uncertain. The uniform microreticulation of the cuticle, the yellowish-orange colour and the tuberculate nature of the node in minor and media workers are sufficient to characterize the species. Despite its relative rarity in collections the species is widespread, with samples from NT, QLD, and WA. Specimens from Gumbardo and Merigol Stations (QM) were collected in mulga habitat. In general, this ant has a northern distribution, with material from St George, Qld, constituting the most southerly record. The St George specimens were collected in box-pine scrub over red soil. The collector added the comment that the ant was foraging in 104°F and was ‘very fast’.

#### Etymology.

Late-Latin *fulvidus* (‘dull brownish-yellow’); adjective in the nominative singular.

**Figure 56.**
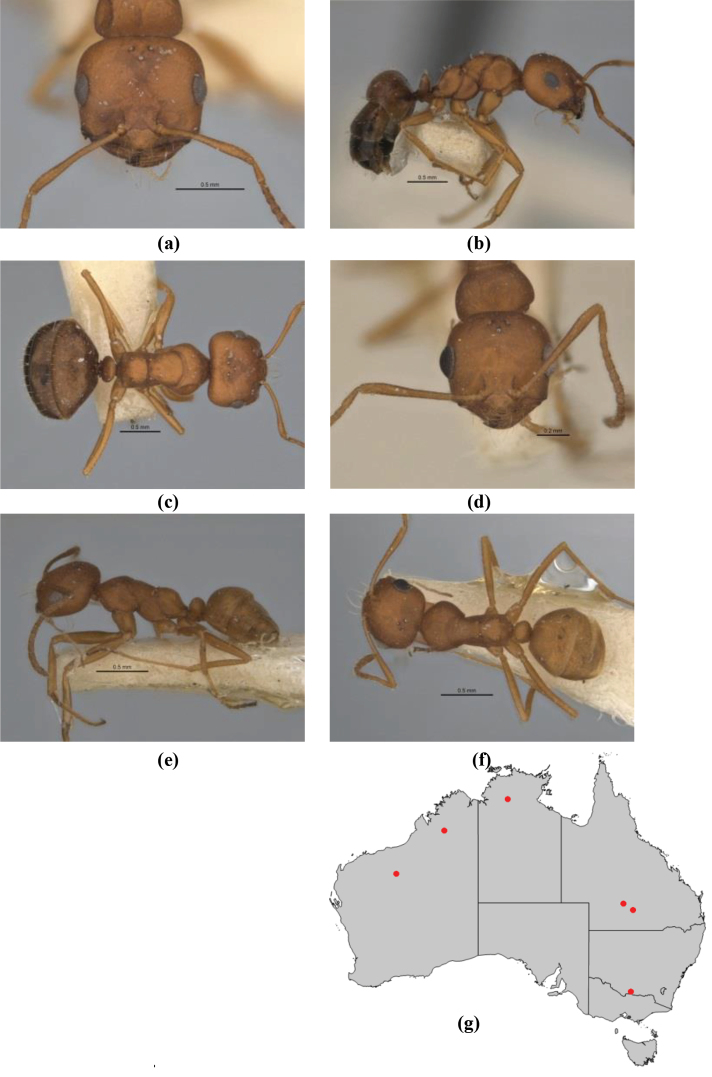
*Melophorus fulvidus* sp. n.: major worker paratype (ANIC32-900119–second ant from top) frons (**a**), profile (**b**) and dorsum (**c**); minor worker holotype (ANIC32-900119–top ant) frons (**d**), profile (**e**) and dorsum (**f**); distribution map for the species (**g**). Low resolution scale bars: 0.5 mm (**a–c, e, f**); 0.2 mm (**d**).

### Melophorus gilliatensis

Taxon classificationAnimaliaHymenopteraFormicidae

Heterick, Castalanelli & Shattuck sp. n.

http://zoobank.org/26EE221C-44FA-4E77-A037-6C3C1AD13900

#### Types.

Holotype minor worker (top ant) from 3 miles ENE of Gilliat, Queensland, 15 April 1962, J. E. Dowse, A.307 [ANIC32-066645] (ANIC). Paratypes: 2 major workers on same pin and with same details as the holotype (ANIC); minor worker and 2 major workers from 3 miles ENE of Gilliat, Queensland, 15 April 1962, J. E. Dowse, A.307 [ANIC32-900059] (MCZ); minor worker from Ernest Henry Mine 20°28.0'S, 140°43.1'E, M. Sanders, site 1 (grassland A), pitfall 52010, native *Atrebla* grassland, Queensland Museum Loan Date: Nov. 2012 No. Ent 12.47 (QM).

#### Diagnosis.

*Melophorus gilliatensis* can be placed in the *M. biroi* species-group on the basis of characters of the clypeus, propodeum, mandible and palps. The species is also placed in the *M. fieldi* species-complex because of the appearance of the anteriorly placed clypeal psammophore, the compact propodeum, the presence of more than one preapical spine on the metatibia, at least in the major worker, the long, even spindly legs and the unmodified mandible in the major worker. The clypeus is folded back abruptly slightly below its midpoint, so that the anterior portion projects as a protuberance at about 90° to the mandible, the clypeal psammophore being placed along the line of demarcation. The unusual form of the clypeus, suggestive of ants in the *M. wheeleri* complex, serves to identify the species, which is restricted to a small area of central QLD.

#### Minor worker description.

**Head.** Head square; posterior margin of head planar or weakly concave; frons shining with superficial shagreenation or microreticulation only; pilosity of frons a mixture of short, erect and semi-erect setae interspersed with shorter decumbent setae and well-spaced, short, appressed setae. Eye moderate (eye length 0.20–0.49 length of side of head capsule); in full-face view, eyes set above midpoint of head capsule; in profile, eye set anteriad of midline of head capsule; eyes elliptical or slightly reniform. In full-face view, frontal carinae straight or weakly convex; frontal lobes straight in front of antennal insertion. Anteromedial clypeal margin straight and retroussé anteromedially; clypeal psammophore set below midpoint of clypeus; palp formula 6,4. Five mandibular teeth in minor worker; mandibles triangular, weakly incurved; third mandibular tooth distinctly shorter than apical tooth and teeth numbers two and four; masticatory margin of mandibles approximately vertical or weakly oblique. **Mesosoma.** Integument of pronotum, mesonotum and mesopleuron shining and microreticulate, microreticulation reduced on humeri; anterior mesosoma in profile broadly convex; appearance of erect pronotal setae short, (i.e., longest erect setae shorter than length of eye) and unmodified; in profile, metanotal groove shallow, broadly V or U-shaped; propodeum shining and finely striolate and microreticulate; propodeum smoothly rounded or with indistinct angle, or angulate, propodeal angle blunt; length ratio of propodeal dorsum to its declivity between 1:1 and 1:2; erect propodeal setae present and sparse to moderate (1-12); appressed propodeal setulae long, each reaching setae behind and in front, but not forming pubescence; propodeal spiracle situated on or beside declivitous face of propodeum, and shorter (length < 0.50 × height of propodeum). **Petiole.** In profile, petiolar node squamiform; in full-face view, shape of petiolar node uniformly rounded; node shining and faintly striolate and microreticulate. **Gaster.** Gaster shining, shagreenate (‘LP record’ appearance); pilosity of first gastral tergite consisting of a mixture of curved, erect and semi-erect setae and decumbent and appressed setae that form a variable pubescence. **General characters.** Colour of foreparts brown, gaster dark brown.

#### Major worker description.

**Head.** Head square tending to trapezoid anteriad; posterior margin of head weakly concave; cuticle of frons shining with superficial shagreenation or microreticulation only; pilosity of frons a mixture of many long, curved, semi-erect setae over well-spaced short semi-erect, decumbent and appressed setae. Eye moderate (eye length 0.20–0.49 length of head capsule); in full-face view, eyes set above midpoint of head capsule; in profile, eye set anteriad of midline of head capsule; eyes elliptical. In full-face view, frontal carinae straight or weakly convex; frontal lobes straight in front of antennal insertion. Anterior clypeal margin broadly and evenly convex; clypeal psammophore set below midpoint of clypeus; palp formula 6,4. Five mandibular teeth in major worker; mandibles triangular, weakly incurved; third mandibular tooth distinctly shorter than apical tooth and teeth numbers two and four; masticatory margin of mandibles approximately aligned vertically or weakly oblique. **Mesosoma.** Integument of pronotum, mesonotum and mesopleuron shining with very superficial microreticulation, entire lower mesopleuron distinctly shagreenate; anterior mesosoma in profile pronotum smoothly rounded anteriad and flattened posteriad, mesonotum narrowly convex; erect pronotal setae long (i.e., longer than length of eye) and unmodified; in profile, metanotal groove shallow, indicated mainly by an angle and metathoracic spiracles; propodeum shining and finely striolate and microreticulate; propodeum smoothly rounded or with indistinct angle; propodeal dorsum and declivity confluent; erect propodeal setae present and abundant (at least a dozen); appressed propodeal setae long, each reaching setae behind and in front, but not forming pubescence; propodeal spiracle situated nearer to midpoint of propodeum than to its declivitous face, and shorter (length less than 0.50 × height of propodeum). **Petiole.** In profile, petiolar node squamiform; in full-face view, shape of petiolar node generally rounded with median indentation; node shining and faintly striolate and microreticulate. **Gaster.** Gaster shining, shagreenate (‘LP record’ appearance); pilosity of first gastral tergite consisting of a mixture of curved, erect and semi- erect setae and decumbent setae that form a variable pubescence. **General characters.** Colour of foreparts very variable, including orange, black, or orange with brownish-red head; gaster dark brown to black.

#### Measurements.

Worker (n = 4): CI 103–120; EI 18–32; EL 0.28–0.30; HL 0.83–1.38; HW 0.85=1.66; ML 1.20–1.66; MTL 0.77–1.05; PpH 0.11–0.20; PpL 0.50–0.81; SI 64–113; SL 0.97–1.07.

#### Comments.

Material belonging to this species has been collected in just two almost contiguous localities (Gilliat and Ernest Henry Mine) in central QLD. The minor worker from Ernest Henry Mine was collected in a pitfall trap in *Astrebla* grassland. Nothing more is known of this rare and localized ant.

#### Etymology.

Named for the type locality; third declension masculine suffix added to toponym to form an adjective.

**Figure 57.**
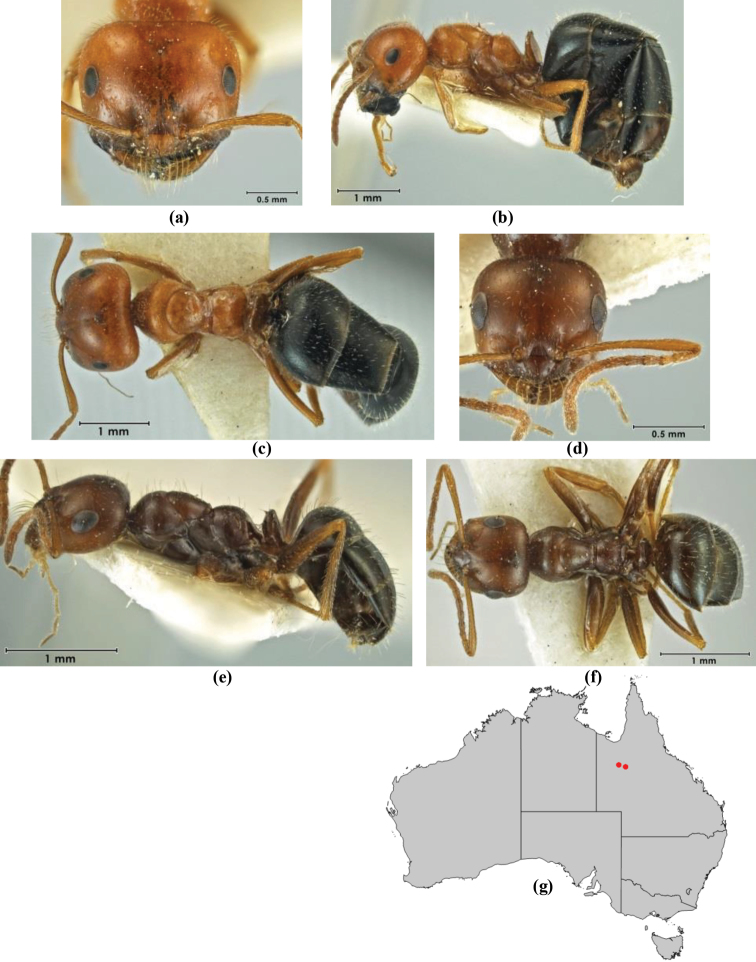
*Melophorus gilliatensis* sp. n.: major worker (ANIC32-066645–top ant) paratype frons (**a**), profile (**b**) and dorsum (**c**); minor worker holotype (ANIC32-066645–middle ant) frons (**d**), profile (**e**) and dorsum (**f**); distribution map for the species (**g**).

### Melophorus hirsutipes

Taxon classificationAnimaliaHymenopteraFormicidae

Heterick, Castalanelli & Shattuck sp. n.

http://zoobank.org/A67B0851-12FF-44BE-AFE1-FACBE20A8216

#### Types.

Holotype minor worker (bottom ant) from Penrose Park, Broken Hill, New South Wales, 10 April 1960, R.H. Mew, dry sclerophyll (typed and hand-written label, respectively) [ANIC32-900147] (ANIC). Paratypes: 2 major workers on same pin and with same details as minor worker (ANIC); 2 major workers and a minor worker from Muloorina HS 29°17'16"S, 137°55'22"E, Lake Eyre South, South Australia, 10 October 2005, S.O. Shattuck #4529, low mound with simple entrance (ANIC ANT VIAL 75-34) [ANIC32-029182] (BMNH); 2 major workers and a minor worker from Pennrose Park, Broken Hill, New South Wales, 10 April 1960, R.H. Mew, dry sclerophyll [ANIC32-900152] (MCZ); minor worker from billabong W Salty Bore, Craven Peak Station 23°01'22"S, 138°13'18"E, Queensland, 11–14 April 2007, C. Lemann, malaise trough, coolabahs and herbs beside billabong [ANIC32-035381] (QM); major worker and minor worker from Cambrai, South Australia, 29 February 1972, P.J.M. Greenslade [ANIC32-900155] (SAM).

#### Other material examined.

**New South Wales**: 20 mi N Tottenham (Lowery, B.B. [ANIC32-900153]), 20 mi SE Bourke (Greaves, T.), 40 km NNW Louth, Lake Mere (Greenslade, P.J.M. [ANIC32-900093]), Fowlers Gap (Greenslade, P.J.M. [ANIC32-900164]), Fowlers Gap (Greenslade, P.J.M. [ANIC32-900163]), Sturt National Park (Greenslade, P.J.M.). **Northern Territory**: 11 km N Tennant Creek (Davidson, D. & Morton, S.), 30 km SW Katherine (Greenslade, P.J.M. [ANIC32-900092]), about 5 km N Henbury Homestead (Feehan, J.E. [ANIC32-900150]), Elliott (Weatherill, L.), Kunoth Paddock, near Alice Springs (Greenslade, P.J.M.), Manbulloo, SW Katherine (Greenslade, P.J.M.), Tanami (Greenslade, P.J.M.), Tanami (Greenslade, P.J.M.). **Queensland**: ‘Gumbardo’ (T. Beutel), ‘Merigol’ (T. Beutel), ‘Merigol’ (T. Beutel), 14 mi W Cloncurry (Dowse, J.E.), Alton Downs old homestead, about 48 km SW Birdsville (Feehan, J.E. [ANIC32-900151]), Mt. Isa (Weatherill, L.), Nairana (E. M. Exley), Sandringham (Morton, S. [ANIC32-900162]), Sandringham Station, 55 km NW Bedourie (Morton, S.R. [ANIC32-900161]). **South Australia**: 10 km E Mt Ive Homestead, Gawler Ranges (Greenslade, P.J.M.), 10 km E Mt Ive Homestead, Gawler Ranges (Greenslade, P.J.M.), 2 km NE Kalamurina Homestead (Feehan, J.E. [ANIC32-900159]), 30 km S Granite Downs (Greenslade, P.J.M.), 3 km NE homestead, Koonamore (Greenslade, P.J.M.), 80 km E Emu, Victoria Desert (Greenslade, P.J.M. [ANIC32-900158]), Cambrai (Greenslade, P.J.M. [ANIC32-900157]), Eckerts Creek (Barmera Primary [ANIC32-046265]), Eyre Hwy, 9.7 km NE Cootra (B.E. Heterick [M74]), Koonamore (Greenslade, P.J.M.), Koonamore (Greenslade, P.J.M.), Koonamore (Greenslade, P.J.M.), Moolooloo, Flinders Ranges (Riddle, A.R.), Olympic Dam (Matthews, E.G. & Watts, C.), Oraparinna, Flinders Ranges (Greenslade, P.J.M. [ANIC32-900160]). **Victoria**: 15 km WNW Yaapeet (Andersen, A.N. [ANIC32-900167]), 15 km WNW Yaapeet (Andersen, A.N. [ANIC32-900168]). **Western Australia**: 1.5 km S Koolyanobbing (B.E. Heterick [M19/M51/M52]), 50 km NE Wiluna (F. Bokhari [M323]), 70 km N Halls Creek (B.E. Heterick [M210]), Cape Bernier (Andersen, A.N.), Coomallo Downs (B.E. Heterick [M01]), Durack River (J. B. Stuckey [M114]), Ethel Creek (Varris, P.A. [JDM32-004544]), G. J. Rd, 108 km E Carnarvon (B.E. Heterick [M311]), KW Road, Lancelin district (B.E. Heterick [M119]), Margaret River, Marillana Creek (Dunlop, N. [JDM32-004547]), Mulga Rock (S. Dunnart [M92]), Mulga, NE Goldfields (Pringle, H.J.R. [ANIC32-029568]), Pannawonica Hill (F. Bokhari [M153]), Rabbit Proof Fence (B.E. Heterick [M69/M70]), Wittenoom Gorge (Heterick, B.E. [JDM32-004543]), Yampi 2 Stn, Kimberley Area (Palmer, C. [JDM32-004546]).

#### Diagnosis.

*Melophorus hirsutipes* can be placed in the *M. biroi* species-group on the basis of characters of the clypeus, propodeum, mandible and palps. The species is also placed in the *M. fieldi* species-complex because of the appearance of the anteriorly placed clypeal psammophore, the compact propodeum, the presence of more than one preapical spine on the metatibia, at least in the major worker, the long, even spindly legs and the unmodified mandible in the major worker.

The species is highly variable in appearance: (1) *Melophorus hirsutipes* (‘‘*pillipes*’’). This form of the species clusters with several others in the *M. fieldi* complex which share the following characters: gaster with curved erect setae, semi-erect setae and a few decumbent setae only, genuine appressed setae lacking; the body generally strongly sculptured and hirsute and the antennal scapes and legs with whorls of many fine, straight setae. *Melophorus hirsutipes* is distinguishable from *M. incisus* in that the mesonotum and propodeum are not globose and the mesonotum is separated from the mesopleuron and propodeum by a weak groove or indentation. *Melophorus hirsutipes* can be distinguished from *Melophorus ankylochaetes* because of the thicker, petiolar node of major and media workers (when viewed in profile), which is more tubercular in appearance and often directed posteriad. The sculpture of the head varies from weakly shining to matt and rugulose. The minor worker has a sloping propodeum with curved, erect setae.

(2) The less hirsute form of *M. hirsutipes*, which lacks erect setae on the tibiae, can be distinguished from similar *M. fieldi* complex species by the following characters: the mesosoma has long, flexuous, curved erect setae (the pronotal setae and those on anterior mesosoma being curved posteriad, setae on posterior mesonotum being curved anteriad, while those on the propodeum are curved in both directions); rather long appressed setae are present on the mesonotum and gaster but these do not combine to form a silvery pubescence; in profile, the anterior two-thirds of clypeus is often straight or slightly concave, forming an oblique surface, the anteromedial clypeal dimple weak or vestigial and, in profile, the petiolar node in the minor worker is thick, to about 0.7× as wide as high, with its dorsum noticeably directed posteriad in many instances.

#### Minor worker description.

**Head.** Head square; posterior margin of head strongly convex or weakly convex; sculpture of frons very variable, from matt, uniformly striate or rugose (northern populations) down a cline to shining with superficial shagreenation or microreticulation only (many southern populations); pilosity of frons a mixture of short, erect and semi-erect setae interspersed with shorter decumbent setae and well-spaced, short, appressed setae. Eye moderate (eye length 0.20–0.49 length of side of head capsule); in full-face view, eyes set above midpoint of head capsule; in profile, eye set anteriad of midline of head capsule; eyes more-or-less circular, elliptical or slightly reniform. In full-face view, frontal carinae straight, divergent posteriad to distinctly concave; frontal lobes straight in front of antennal insertion. Anteromedial clypeal margin broadly and evenly convex with or without anteromedian dimple; clypeal psammophore set at or above midpoint of clypeus; palp formula 6,4. Five mandibular teeth in minor worker; mandibles triangular, weakly incurved; third mandibular tooth distinctly shorter than apical tooth and teeth numbers two and four; masticatory margin of mandibles approximately vertical or weakly oblique. **Mesosoma.** Integument of pronotum, mesonotum and mesopleuron varying in appearance from matt or weakly shining to strongly shining and mainly smooth (sculpture shagreenate or even striate-rugose on pronotum and dorsum of mesonotum in northern populations and mainly lacking in southern populations with vestigial shagreenation most noticeable on humeri and mesopleuron); anterior mesosoma in profile broadly convex; appearance of erect pronotal setae long (i.e., longest erect setae longer than length of eye) and unmodified; in profile, metanotal groove varying from a vestigial furrow to broadly V or U-shaped; propodeum shining, shagreenate or matt and rugose with multiple hair like striolae; propodeum angulate, propodeal angle blunt; length ratio of propodeal dorsum to its declivity about 4:3, or about 1:1 or smoothly rounded with propodeal dorsum and propodeal declivity confluent (northern populations); erect propodeal setae present and sparse to abundant (greater than 12); appressed propodeal setulae long and separated by at least own length, or sparse or absent, if present then not regularly spaced; propodeal spiracle situated at least twice its width from the declivitous face of propodeum, and shorter (length < 0.50 × height of propodeum), or situated on or beside declivitous face of propodeum, and shorter (length < 0.50 × height of propodeum). **Petiole.** In profile, petiolar node subcuboidal to rectangular, vertex blunt, directed posteriad; in full-face view, shape of petiolar node uniformly rounded; node weakly to moderately shining (northern) to shining and smooth with vestigial sculpture (southern). **Gaster.** Gaster shining with superficial microreticulation; pilosity of first gastral tergite consisting of a mixture of curved, erect and semi-erect setae and decumbent and appressed setae that form a variable pubescence, or wholly or mainly of long, curved setae, appressed setae apparently absent. **General characters.** Colour of foreparts orange-tan or light brown, gaster brown or chocolate.

#### Major worker description.

**Head.** Head horizontally rectangular, broader than wide; posterior margin of head planar or weakly convex (northern) or weakly concave (southern); cuticle of frons varying along a cline from matt or with a weak sheen, indistinct or coriaceous (northern) to shining with superficial shagreenation or microreticulation only (southern); pilosity of frons a mixture of well-spaced, distinctly longer erect and semi-erect setae interspersed with shorter decumbent or appressed setae. Eye moderate (eye length 0.20–0.49 length of head capsule); in full-face view, eyes set at (northern) or above (southern) midpoint of head capsule; in profile, eye set anteriad of midline of head capsule; eyes elliptical. In full-face view, frontal carinae straight, divergent posteriad, or distinctly concave; frontal lobes straight in front of antennal insertion. Anterior clypeal margin broadly and evenly convex, or broadly convex with anteromedial dimple; clypeal psammophore set at or above midpoint of clypeus; palp formula 6,4. Five mandibular teeth in major worker; mandibles triangular, weakly incurved; third mandibular tooth distinctly shorter than apical tooth and teeth numbers two and four; masticatory margin of mandibles approximately aligned vertically or weakly oblique. **Mesosoma.** Integument of pronotum, mesonotum and mesopleuron matt or weakly shining and coriaceous (northern) to moderately shining and shagreenate throughout (southern); anterior mesosoma in profile broadly convex; erect pronotal setae long (i.e., longer than length of eye) and unmodified; in profile, metanotal groove shallow (indicated mainly by an angle and the metathoracic spiracles) to broadly V- or U-shaped; propodeum weakly shining with pitted, rugose sculpture dorsally and striate rugosity laterally, or matt or with a weak sheen and indistinctly shagreenate (northern) or propodeum shining and finely striolate and microreticulate (southern); propodeum smoothly rounded or with indistinct angle (northern) or angulate, propodeal angle blunt; length ratio of propodeal dorsum to its declivity between 1:1 and 1:2 (southern); erect propodeal setae present and abundant (at least a dozen); appressed propodeal setae sparse or absent, if present then not regularly spaced (northern) but long, each seta reaching setae behind and in front, but not forming pubescence (southern); propodeal spiracle situated at least twice its width from the declivitous face of propodeum, and shorter (length less than 0.50 × height of propodeum), or situated on or beside declivitous face of propodeum, and shorter (length less than 0.50 × height of propodeum). **Petiole.** In profile, petiolar node narrowly conical, vertex blunt (northern) to squamiform (southern); in full-face view, shape of petiolar node square with rounded angles, uniformly rounded, or tapered with blunt vertex; node shining and faintly shagreenate-microreticulate. **Gaster.** Gaster shining, shagreenate (‘LP record’) to shining with superficial microreticulation; pilosity of first gastral tergite consisting of wholly or mainly of long, curved setae, appressed setae apparently absent (mainly northern) or a mixture of curved, erect and semi-erect setae and decumbent setae that form a variable pubescence (mainly southern). **General characters.** Colour of foreparts orange-tan, gaster brown to chocolate.

#### Measurements.

Worker (n = 6): CI 106–127; EI 17–32; EL 0.20–0.33; HL 0.72–1.54; HW 0.76–2.06; ML 1.03–2.06; MTL 0.66–1.38; PpH 0.10–0.26; PpL0.40–0.83; SI 67–114; SL 0.87–1.44.

#### Comments.

*Melophorus hirsutipes* is one of a cluster of closely related members of the *M. fieldi* complex that are not only abundant and widely distributed throughout the Australian mainland, but pose at times intractable taxonomic issues. This species, as presently constituted, reveals a bewildering degree of variability in its sculpture and appearance and is by far the most physically variable *Melophorus*. Specimens at morphological extremes would justifiably be placed in several different taxa were only a few specimens available for analysis. However, in actual fact, many samples of this ant are available. Connecting this bewildering variation are the long, flexuous, mesosomal pilosity, the lack of long, silvery appressed setae that form a thatch and the general appearance of the node. Populations with the ‘*pillipes*’ condition from the temperate zone are often extremely hairy, smooth and shining with long, tapering metatibiae. The mesosomal dorsum is undulant and most characteristically the clypeus is conspicuously flat, even depressed, with the clypeal psammophore placed high on that sclerite. At the other extreme, workers from the Kimberley region in WA are more compact and may present with a matt, distinctly corrugated appearance to the cuticle (a degree of sculpture that is unusual in *Melophorus*), a convex, parallel-ridged clypeus and more robust, curved mesosomal setae. Further south, the workers are areolate-rugose. In these latter two cases the petiolar node is broad and explanate (compared with the narrowly ellipsoid, often tilted node in the smoother worker). Workers of other populations still have a matt appearance, but the sculpture is reduced to an even shagreenation, and the shape of the node falls in between these two extremes. Many populations of appearance intermediate between the two extremes exist, but do not necessarily form a morphological cline: workers from the same locality (Merigol Station, QLD) belong to both the smooth, shining morphotype and the areolate-rugose morphotype. Other workers from southern locations do not exhibit the ‘*pillipes*’ condition and are generally less hirsute. The more markedly sculptured morphs were originally named ‘*hispidus*’, on the assumption they constituted a good species.

On a five-gene tree, this species is sister to *M. inconspicuus* and these two taxa in turn are most closely related to *M. microtriches*. This is a strongly attested result, especially given the firm branch support in the five-gene tree, the fact that multiple specimens of *M. hirsutipes* and *M. microtriches* were able to be successfully sequenced, and the convergent morphology of the three species.

*Melophorus hirsutipes* workers form a significant proportion of the catch in many drier areas of Australia, and the species is present in all mainland Australian states. Most material has been taken in pitfall traps. This species and *M. lanuginosus* seem to be particularly common in heathland, but *M. hirsutipes* has also been taken in mulga woodland, hummock grassland, and sclerophyll woodland. Red clay soil seems to be a preferred substrate. No data are available on the habits of the species, but the ant exhibits no particular morphological specializations compared with related species and is probably a generalized scavenger feeding on animal and plant material.

#### Etymology.

Latin *hirsutus* (‘shaggy’) plus *pes* (‘foot’); adjective in the nominative case.

**Figure 58.**
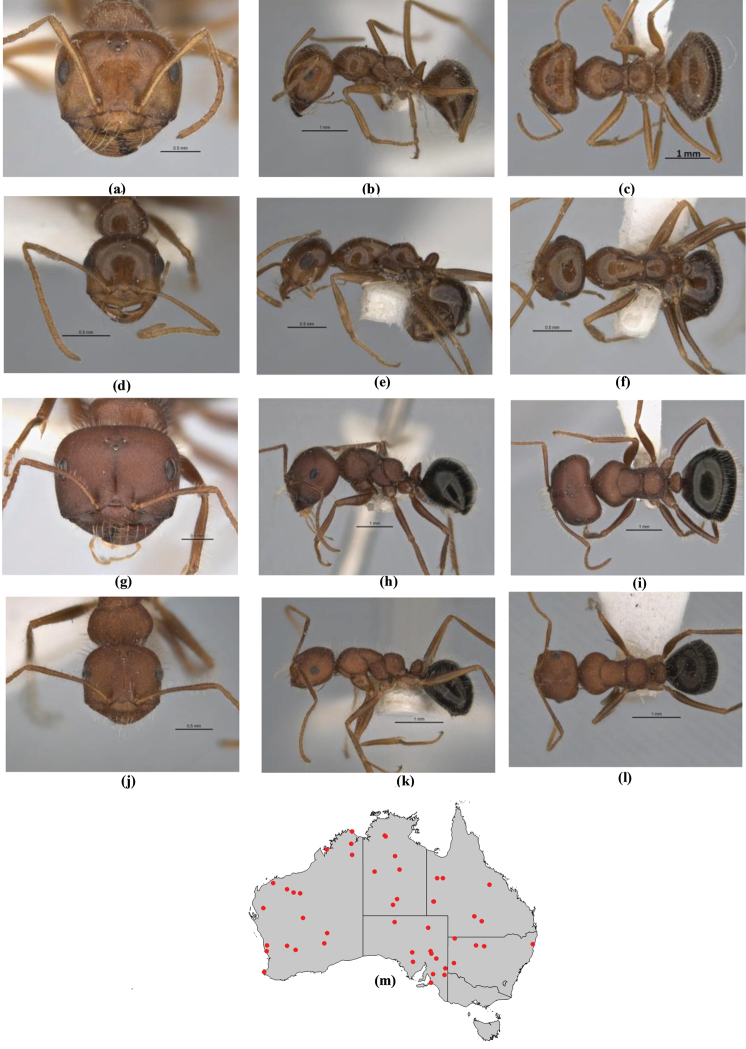
*Melophorus hirsutipes* sp. n.: major worker paratype (ANIC32-900147–top ant) frons (**a**), profile (**b**) and dorsum (**c**); minor worker holotype (ANIC32-066645–bottom ant) frons (**d**), profile (**e**) and dorsum (**f**); ‘*hispidus*’ form major worker (ANIC32-900093) frons (**g**), profile (**h**) and dorsum (**i**); ‘*hispidus*’ form minor worker (JDM32-004543) frons (**j**), profile (**k**) and dorsum (**l**) distribution map for the species (**m**). Low resolution scale bars: 1 mm (**b, h**, **j, k**, l); 0.5 mm (**a, d–g, j**).

### Melophorus incisus

Taxon classificationAnimaliaHymenopteraFormicidae

Heterick, Castalanelli & Shattuck sp. n.

http://zoobank.org/39F96085-AFAF-4295-93E0-1A5FB7562F69

#### Types.

Holotype minor worker (bottom ant) from 5 km SE of Anthony’s Lagoon 18.00S, 135.34E, Northern Territory, 14 October 1981, D. Davidson & S. Morton, 167b [ANIC32-900170] (ANIC). Paratypes: 2 minor workers on same pin and with same details as holotype (ANIC); major worker from Argyle Diamonds via Kununurra, July 1991, Site 23 *Melophorus* sp. 15, Postle, A.T. [JDM32-001996] (ANIC); minor and major worker from 5 km SE of Anthony’s Lagoon 18.00S, 135.34E, Northern Territory, 14 October 1981, D. Davidson & S. Morton, 167a [ANIC32-900170], V, K, 6 (BMNH); 2 media and a minor worker from 22 km SE of Katherine, Northern Territory, 8 April 1978, P.J.M. Greenslade, (7), 14 [ANIC32-900090] (MCZ); minor worker from Marandoo minesite 22°38'11"S, 118°07'31"E, Western Australia, 28 August-18 September 2009, J.L. Brown, pitfall trap RR1 0M, mine dump [JDM32-004528] (WAM).

#### Other material examined.

**Western Australia**: 30 km N Sandfire Hill (Heterick, B.E. [M184/M185/M186]), Pannawonica (Boisduval, M. [JDM32-004620]), Port Hedland (Weatherill, L.), Port Hedland (Heterick, B.E. [M270]), Sandford Rock (Yandle, S. [JDM32-001991]).

#### Diagnosis.

*Melophorus incisus* can be placed in the *M. biroi* species-group on the basis of characters of the clypeus, propodeum, mandible and palps. The species is also placed in the *M. fieldi* species-complex because of the appearance of the anteriorly placed clypeal psammophore, the compact propodeum, the presence of more than one preapical spine on the metatibia, at least in the major worker, the long, even spindly legs and the unmodified mandible in the major worker. Furthermore, this species clusters with several others in the *M. fieldi* complex which share the following characters: gaster with curved erect setae, semi-erect setae and a few decumbent setae only, genuine appressed setae lacking; the body generally strongly sculptured and hirsute and the antennal scapes and legs with whorls of many fine, straight setae. *Melophorus incisus* can then be distinguished from closely allied forms by its globose mesonotum and propodeum and the fact that the mesonotum, the mesopleuron and the propodeum are separated from each other by a deep sulcus.

#### Minor worker description.

**Head.** Head approximately oval with straight sides; posterior margin of head strongly convex; frons shining and smooth except for piliferous pits, or matt or with weak sheen, microreticulate or microreticulate-shagreenate; pilosity of frons a mixture of short, erect and semi-erect setae interspersed with shorter decumbent setae and well-spaced, short, appressed setae. Eye moderate (eye length 0.20–0.49 length of side of head capsule); in full-face view, eyes set above midpoint of head capsule; in profile, eye set anteriad of midline of head capsule; eyes elliptical or slightly reniform. In full-face view, frontal carinae straight, convergent posteriad; frontal lobes curved inward in front of antennal insertion. Anteromedial clypeal margin broadly convex with anteromedial dimple; clypeal psammophore set at or above midpoint of clypeus; palp formula 6,4. Five mandibular teeth in minor worker; mandibles triangular, weakly incurved; third mandibular tooth distinctly shorter than apical tooth and teeth numbers two and four; masticatory margin of mandibles approximately vertical or weakly oblique. **Mesosoma.** Integument of pronotum, mesonotum and mesopleuron matt or with weak sheen and microreticulate throughout, or pronotum smooth and shining, mesonotum shining and superficially microreticulate, mesopleuron densely microreticulate and may be almost matt; anterior mesosoma in profile broadly convex; appearance of erect pronotal setae long (i.e., longest erect setae longer than length of eye) and unmodified; in profile, metanotal groove a narrow but deep slit; propodeum shining and densely microreticulate, with distinct striolae on metapleuron; propodeum always smoothly rounded; length ratio of propodeal dorsum to its declivity not applicable, propodeal dorsum reduced to a narrow sliver; erect propodeal setae present and abundant (greater than 12); appressed propodeal setulae sparse or absent, if present then not regularly spaced propodeal spiracle situated at least twice its width from the declivitous face of propodeum, and shorter (length < 0.50 × height of propodeum). **Petiole.** In profile, petiolar node subcuboidal, vertex bluntly rounded; in full-face view, shape of petiolar node uniformly rounded; node shining and smooth throughout, or shining and striolate. **Gaster.** Gaster smooth and glossy; pilosity of first gastral tergite consisting wholly or mainly of long, curved setae, appressed setae apparently absent. **General characters.** Colour of foreparts reddish-brown to chocolate, gaster chocolate.

#### Major worker description.

**Head.** Head square; posterior margin of head strongly convex; cuticle of frons shining and smooth except for piliferous pits and a few striolae around antennal insertions and frontal carinae; pilosity of frons a mixture of well-spaced, distinctly longer erect and semi-erect setae interspersed with shorter decumbent setae. Eye moderate (eye length 0.20–0.49 length of head capsule); in full-face view, eyes set at midpoint of head capsule; in profile, eye set anteriad of midline of head capsule; eyes elliptical. In full-face view, frontal carinae concave; frontal lobes curved inward in front of antennal insertion. Anterior clypeal margin broadly convex with anteromedial dimple; clypeal psammophore set at or above midpoint of clypeus; palp formula 6,4. Five mandibular teeth in major worker; mandibles triangular, weakly incurved; third mandibular tooth distinctly shorter than apical tooth and teeth numbers two and four; masticatory margin of mandibles approximately aligned vertically or weakly oblique. **Mesosoma.** Integument of pronotum, mesonotum and mesopleuron shining with very superficial microreticulation, entire lower mesopleuron distinctly shagreenate; anterior mesosoma in profile broadly convex; erect pronotal setae long (i.e., longer than length of eye) and unmodified; in profile, metanotal groove deep, V-shaped; propodeum shining and microreticulate; propodeum always smoothly rounded; propodeal dorsum and declivity confluent; erect propodeal setae present and abundant (at least a dozen); appressed propodeal setae sparse or absent, if present then not regularly spaced; propodeal spiracle situated on or beside declivitous face of propodeum, and longer (length ≥ 0.50 × height of propodeum). In profile, petiolar node squamiform; in full-face view, shape of petiolar node tapered with squared-off vertex; node shining and smooth throughout. **Gaster.** Gaster shining with superficial microreticulation; pilosity of first gastral tergite consisting wholly or mainly of long, curved setae, appressed setae apparently absent. **General characters.** Colour of foreparts orange tan, gaster brown.

#### Measurements.

Worker (n = 6): CI 96–117; EI 19–31; EL 0.18–0.29; HL 0.60–1.27; HW 0.78–1.49; ML 0.84–1.55; MTL 0.48–0.93; PpH 0.13–0.20; PpL 0.36–0.59; SI 70–118; SL 0.68–1.05.

#### Comments.

A small, hairy species, *Melophorus incisus* is easily recognized by the deeply incised grooves on the mesosoma, and the rotund nature of the anterior thoracic segments and the propodeum. This ant resembles *M. ankylochaetes*, and genetic sequencing confirms a sister relationship between the two taxa. The species occupies a wide range in the NT and WA, but has not been recorded from any other state thus far. Foragers have been aspirated on the crest of a small dune 30 km from Sandfire Hill, WA and from among tussock grass in seaside dunes in the town of Port Hedland, WA, but other data are lacking.

#### Etymology.

Latin *incisus* (‘cut’ or ‘dissected’); adjective in the nominative case.

**Figure 59.**
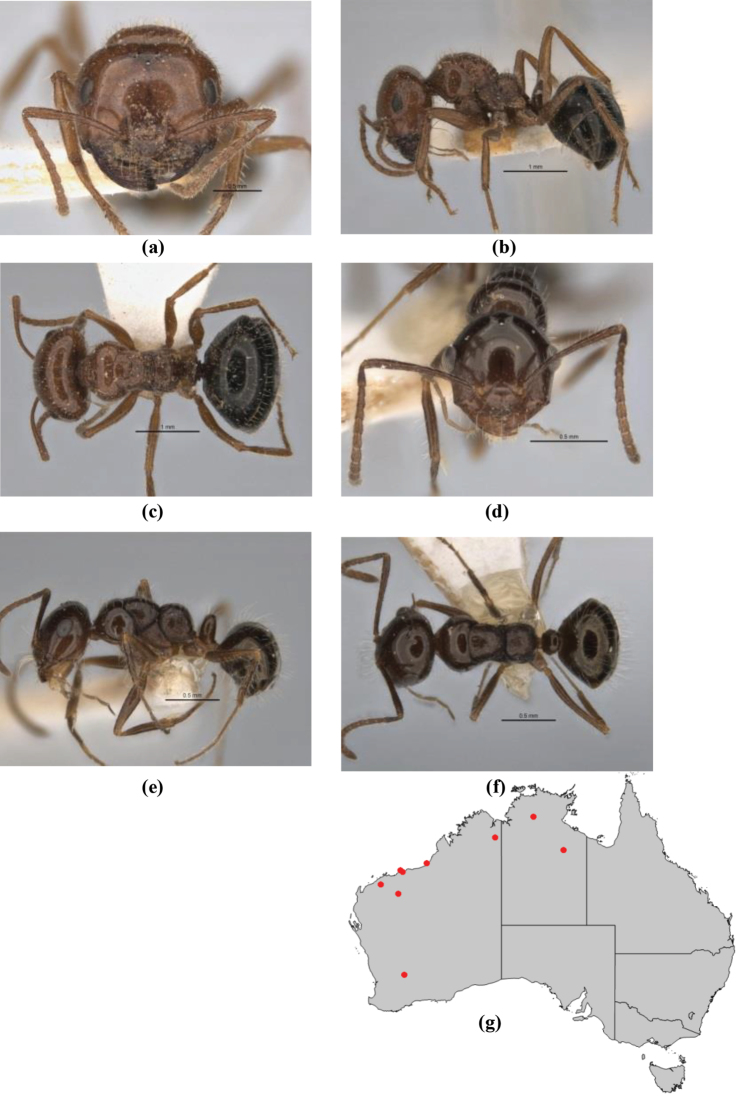
*Melophorus incisus* sp. n.: major worker paratype (JDM32-001996) frons (**a**), profile (**b**) and dorsum (**c**); minor worker holotype (ANIC32-900170–bottom ant) frons (**d**), profile (**e**) and dorsum (**f**); distribution map for the species (**g**). Low resolution scale bars: 1 mm (**b, c**); 0.5 mm (**a, d–f**).

### Melophorus inconspicuus

Taxon classificationAnimaliaHymenopteraFormicidae

Heterick, Castalanelli & Shattuck sp. n.

http://zoobank.org/3160ECE9-81F1-45CB-9F15-CD4EECD27273

#### Types.

Holotype minor worker (bottom ant) from Mt Gason, South Australia, January-February 1995 (collector not stated); 546, ANIC ANTS VIAL 75.63 [ANIC32-006700] (ANIC). Paratypes: Major and media worker on the same pin and with the details as the holotype (ANIC); 3 major workers from 10 km E of Mt Ive HS, Gawler Range, South Australia, 22 October 1980, P.J.M. Greenslade, B Se [ANIC32-066426] (BMNH); 2 major workers and minor worker from Banff, Coorong, South Australia, 13 October 1975, P.J.M. Greenslade, (3) [ANIC32-066405] (BMNH); 2 major workers and minor worker from Tomago 32°52'S, 151°45'E, New South Wales, 17 December 1992, G.P. Jackson, mined 1963 [ANIC32-015278] (MCZ); 1 major, 1 media and minor worker from Rocky River, Kangaroo Island, South Australia, [‘PT/’] 3 January 1977, P.J.M. Greenslade, (SAM); 2 media workers and minor worker from Whitfords Beach 31°47'S, 115°43'E, Western Australia, 2 July 2005, B.E. Heterick, car park verge beside dune, mid pm [JDM32-001790] (WAM); major worker and 2 media workers from Stirling Range Gold Holes, Western Australia, 28 October 1969, R.W.T, c 250m, dry sclero., R.W.Taylor Accession 69.580 [ANIC32-066398] (WAM).

#### Other material examined.

**New South Wales**: 40 km NNW Louth, Lake Mere (Greenslade, P.J.M.), 40 km W Broken Hill (Greenslade, P.J.M.), Fowlers Gap (Greenslade, P.J.M.), Fowlers Gap (Greenslade, P.J.M.), Mundi Mundi, near Broken Hill (Valentine, J.), near Deniliquin (Zakharov [ANIC32-013879]). **Northern Territory**: 105 km N Yuendumu (Greenslade, P.J.M.), 15 km SE Alice Springs (Davidson, D. & Morton, S.), 17 km NW Alexandria Downs (Davidson, D. & Morton, S.), 20 km S Alice Springs (Andersen, A.N.), 20 km S Alice Springs (Greenslade, P.J.M.), 20 km SE Alice Springs (Davidson, D. & Morton, S.), 20 km SE Alice Springs (Davidson, D. & Morton, S.), 25 km W Tennant Creek (Greenslade, P.J.M.), about 50 km E Finke (Feehan, J.E.), Kunoth Paddock, near Alice Springs (Greenslade, P.J.M.), Tanami (Greenslade, P.J.M.), Tanami (Greenslade, P.J.M.). **Queensland**: 20 mi S St George (Lowery, B.B.), Mt. Isa (Weatherill, L.), near Thallon (Lowery, B.B.). **South Australia**: 10 km E Mt Ive Homestead, Gawler Ranges (Greenslade, P.J.M.), 10 km E Mt Ive Homestead, Gawler Ranges (Greenslade, P.J.M.), 10 km SW Meningie (Greenslade, P.J.M.), 12 km W Emu, Victoria Desert (Greenslade, P.J.M.), 18 km NNE Meningie (Greenslade, P.J.M.), 20 km NE Macumba Station (Davidson, D. & Morton, S.), 30 km S Granite Downs (Greenslade, P.J.M.), 3 km NE homestead, Koonamore (Greenslade, P.J.M.), 5 km WNW Pitlochry Homestead, Coorong-Keith (Greenslade, P.J.M.), 85 km W Mabel Creek (Greenslade, P.J.M.), Banff, Coorong (Greenslade, P.J.M.), Bay of Shoals, Kangaroo Island (Greenslade, P.J.M.), Belair (Greenslade, P.J.M.), Bindyi, Koonamore (Greenslade, P.J.M.), BM 17, Coorong (Greenslade, P.J.M.), Cambrai (Greenslade, P.J.M.), Cambrai (Greenslade, P.J.M.), Cambrai (Greenslade, P.J.M.), Cambrai (Greenslade, P.J.M.), Cambrai (Greenslade, P.J.M.), Cambrai (Greenslade, P.J.M.), Cambrai (Greenslade, P.J.M.), Cockatoo, Coorong (Greenslade, P.J.M.), Cockatoo, Coorong (Greenslade, P.J.M.), Curdimurka (Feehan, J.E.), E Horse Rock, Port Lincoln, Eyre Peninsula (Greenslade, P.J.M.), Fairview Conservation Park, N Lucindale (Greenslade, P.J.M.), Fairview Conservation Park, N Lucindale (Greenslade, P.J.M.), Gawler Ranges (Greenslade, P.J.M.), Hanson Bay, Kangaroo Island (Greenslade, P.J.M.), head of Bight Cliff, 54 km WbyS Nullarbor (Taylor, R.W.), Innes National Park, Yorke Peninsula (Greenslade, P.J.M.), Koonamore (Greenslade, P.J.M.), Koonamore (Greenslade, P.J.M.), Moorowie Plain (Greenslade, P.J.M.), Oraparinna, Flinders Ranges (Greenslade, P.J.M.), Poochera (Taylor, R.W. & Bartell, R.J.), Remarkable Rocks, Kangaroo Island (Greenslade, P.J.M.), Salt Creek, 8 km S (Animal and Plant Control Commission), Salt Creek, Coorong (Greenslade, P.J.M.), Salt Lagoon, near Bay of Shoals, Kangaroo Island (Greenslade, P.J.M.), Sandy Creek, Kangaroo Island (Greenslade, P.J.M.), Victor Harbour (Greenslade, P.J.M.), West Bay, Kangaroo Island (Greenslade, P.J.M.), Yorke Peninsula (Greenslade, P.J.M.). **Victoria**: 15 km WNW Yaapeet (Andersen, A.N.), 15 km WNW Yaapeet (Andersen, A.N.), Little Desert (Andersen, A.N.). **Western Australia**: 1 km S Capricorn RH (Heterick, B.E. [M275]), 12 mi N Wittenoom (McInnes & Dowse), 36 mi NE Carnegie Homestead (McInnes, R. & Dowse, J.), 48 mi E Cosmo Newberry Mission (McInnes & Dowse), 48 mi E Cosmo Newberry Mission (McInnes & Dowse), Argyle Diamonds via Kununurra (Postle, A. [JDM32-004713]), Binningup turnoff (Heterick, B.E. [M123]), Bold Park (Achour, P. [JDM32-001802]), Boundary Island (Piek, J. [JDM32-001804]), Ethel Creek (Varris, P.A. [JDM32-004577]), Gibb R. (Mercer, R. [JDM32-001821]), Hope Valley (Heterick, B.E.), Israelite Bay (Greaves, T.), Martin (Heterick, B.E.), Milyeannup (Parkinson, W. [JDM32-001791]), Mt. Whaleback, Newman (Walker, K.J. [JDM32-001828]), Nerren Nerren Stn. (Harvey, M.S. & Waldock, J.M. [JDM32-004759]), Perth (Majer, J.D. [JDM32-001813]), Perth (Clark, J.).

#### Diagnosis.

*Melophorus inconpicuus* can be placed in the *M. biroi* species-group on the basis of characters of the clypeus, propodeum, mandible and palps. The species is also placed in the *M. fieldi* species-complex because of the appearance of the anteriorly placed clypeal psammophore, the compact propodeum, the presence of more than one preapical spine on the metatibia, at least in the major worker, the long, even spindly legs and the unmodified mandible in the major worker. *Melophours inconspicuus* belongs to a clade of very similar and highly derived *Melophorus* that are also common and widespread, and specimens have to be checked carefully using the following characters before they can be distinguished from *M. bruneus*, *M. fieldi*, *M. hirsutipes*, *M. longipes*, *M. lanuginosus* and *M. turneri*: the eye in minor workers is moderate in size (large in *M. bruneus* and *M. fieldi* minor wokers), in profile, the clypeus is evenly convex or weakly concave in its anterior third, the petiolar node is thin, much less than 0.7× as wide as high and either straight or weakly bent posteriad. The appressed setae on the mesosoma and gaster are moderate in length and interspersed with bristly erect setae (this is a good character by which to distinguish this ant from workers of *M. turneri*, in which the appressed setae are very short), the silvery pubescence is limited to small areas of the pronotum or entirely absent (forms a thick thatch in *M. lanuginosus*); the erect setae on mesosoma are generally few and bristly in appearance (often < diameter of eye, unlike the longer and more flexuous setae in *M. hirsutipes*) and the entire mesosoma may be glabrous. The cuticle is generally weakly shagreenate, except for the mesopleuron, and the foreparts are commonly moderately shining. the petiolar node in the minor worker essentially squamiform, that of major worker a true scale. The major worker can be distinguished from major workers of *M. bruneus* and *M. fieldi* by its larger size (HW > 1.70 mm versus HW < 1.60 mm) and its less finely sculptured and shinier appearance. The ‘*pillipes*’ condition is always absent.

#### Minor worker description.

**Head.** Head square; posterior margin of head planar or weakly convex; frons matt or with weak sheen, microreticulate or microreticulate-shagreenate; pilosity of frons a mixture of a few well-spaced, erect setae interspersed with appressed setae only, or consisting exclusively or almost exclusively of well-spaced, appressed setae only (small, erect setae, if present, usually confined to ocular triangle or posterior margin of head). Eye moderate (eye length 0.20–0.49 length of side of head capsule); in full-face view, eyes set above midpoint of head capsule; in profile, eye set anteriad of midline of head capsule; eyes elliptical or slightly reniform. In full-face view, frontal carinae straight or weakly convex; frontal lobes straight in front of antennal insertion. Anteromedial clypeal margin broadly emarginate with projecting anteromedial dimple, or straight; clypeal psammophore set at or above midpoint of clypeus; palp formula 6,4. Five mandibular teeth in minor worker; mandibles triangular, weakly incurved; third mandibular tooth distinctly shorter than apical tooth and teeth numbers two and four; masticatory margin of mandibles approximately vertical or weakly oblique. **Mesosoma.** Integument of pronotum, mesonotum and mesopleuron with weak to moderate sheen, shagreenate on pronotum and dorsum of mesonotum, otherwise microreticulate; anterior mesosoma in profile broadly convex; appearance of erect pronotal setae short, (i.e., longest erect setae shorter than length of eye) and unmodified, or erect pronotal setae absent; in profile, metanotal groove shallow, broadly V or U-shaped; propodeum matt or with a weak sheen and microreticulate; propodeum smoothly rounded or with indistinct angle; propodeal dorsum and declivity confluent; erect propodeal setae variable in number, may be absent; appressed propodeal setulae long, each reaching setae behind and in front, but not forming pubescence; propodeal spiracle situated on or beside declivitous face of propodeum, and longer (length ≥ 0.50 × height of propodeum). **Petiole.** In profile, petiolar node squamiform; in full-face view, shape of petiolar node uniformly rounded, or tapered with blunt vertex; node shining and distinctly microreticulate. **Gaster.** Gaster shining with superficial microreticulation; pilosity of first gastral tergite consisting of thick, appressed setae that form pubescence, interspersed with numerous short, bristly, erect setae. ***General characters***. Colour concolorous brown to blackish-brown.

#### Major worker description.

**Head.** Head horizontally rectangular, broader than wide; posterior margin of head planar or weakly concave; cuticle of frons shining with superficial shagreenation or microreticulation only, or matt or with weak sheen, microreticulate; pilosity of frons a mixture of a few well-spaced, erect setae interspersed with appressed setae only. Eye moderate (eye length 0.20–0.49 length of head capsule); in full-face view, eyes set above midpoint of head capsule; in profile, eye set anteriad of midline of head capsule; eyes elliptical. In full-face view, frontal carinae straight, divergent posteriad; frontal lobes straight in front of antennal insertion. Anterior clypeal margin broadly convex with anteromedial dimple; clypeal psammophore set at or above midpoint of clypeus; palp formula 6,4. Five mandibular teeth in major worker; mandibles triangular, weakly incurved; third mandibular tooth distinctly shorter than apical tooth and teeth numbers two and four; masticatory margin of mandibles approximately aligned vertically or weakly oblique. **Mesosoma.** Integument of pronotum, mesonotum and mesopleuron moderately shining and shagreenate throughout; anterior mesosoma in profile broadly convex; erect pronotal setae short, (i.e., shorter than length of eye) and unmodified; in profile, metanotal groove shallow, broadly V- or U-shaped; propodeum shining and finely striolate and microreticulate; propodeum angulate, propodeal angle blunt; length ratio of propodeal dorsum to its declivity between 1:1 and 1:2; erect propodeal setae present and abundant (at least a dozen); appressed propodeal setae long, each reaching setae behind and in front, but not forming pubescence; propodeal spiracle situated on or beside declivitous face of propodeum, and shorter (length less than 0.50 × height of propodeum). **Petiole.** In profile, petiolar node squamiform; in full-face view, shape of petiolar node uniformly rounded, or tapered with blunt vertex; node shining and faintly shagreenate-microreticulate. **Gaster.** Gaster shining, shagreenate (‘LP record’ appearance); pilosity of first gastral tergite consisting of short, bristly, erect setae over well-spaced, short, appressed setae. **General characters.** Colour of foreparts variably orange tan to chocolate, gaster chocolate.

#### Measurements.

Worker (n = 8): CI 96–125; EI 18–37; EL 0.21–0.31; HL 0.59–1.38; HW 0.57–1.72; ML 0.86–1.68; MTL 0.58–1.05; PpH 0.08–0.19; PpL 0.35–0.71; SI 67–135; SL 0.76–1.15.

#### Comments.

The name for this species seems apt, as this dull, nondescript member of the *M. fieldi* complex is better defined by what it isn’t than by unique diagnostic characters. In fact, the taxonomy of this ant is unsatisfactory, and more than one species may be lumped under the diagnostic features contained in the key and the description. In appearance, what is here called *inconspicuus* most closely resembles a blackish to medium brownish cross between hairier near relatives (*M. hirsutipes* and *M. lanuginosus*) and the glabrous or near glabrous *M. turneri*. Workers range from glabrous to ants with scattered, short pilosity. The appressed setae are longer than in *M. turneri* (separated from one another on the first gastral segment by less than their length or actually overlapping) and the head and mesosoma are matt or weakly shining with shagreenate sculpture, never strongly shining. The eye is smallish to moderate in size in all subcastes. Seen in full-face view, minor, media and major workers always have a few short, stout, unmodified setae on the vertex and these may encircle the vertex to about eye level as in *M. bruneus* and *M. fieldi*. However, the relatively smaller eye (EI 18-37 compared with an EI of 24-41 in *M. bruneus* and 26-42 in *M. fieldi*) in most specimens and the longer antennal scape will enable this ant to be distinguished from those taxa. The ‘*pillipes*’ condition has not been seen in this species, and it lacks the thatch of silvery appressed setae seen in *M. lanuginosus*. In a five-gene tree summary, *M. inconspicuus* is sister to *M. hirsutipes*, though this is based on just a single specimen of the typical form. However, there is good branch support for this placement.

This ant has a wide distribution throughout mainland Australia and does not seem to have any preferred habitat. Most samples have been collected in SA and southwestern WA. The vegetation zones from which the species has been recorded are very diverse: Banksia woodland, dry sclerophyll, *Casuarina*, mallee woodland, *Eucalyptus largiflorens*, heath, mallee, savanna woodland, box-pine scrub, and ‘arid woodland’. Cambrai (SA) foragers were collected in dunes, while other samples have been taken near rivers, from clay pans and from red sand. At Hilarys, WA, the principal author has collected the blackish south-western form of this species in white beach sand at a car park about 150 m from the ocean. This form has a small eye. Black workers with similar features (held in ANIC) have been recorded from the Stirling RA, WA. The black form with the small eyes has not been successfully sequenced. Despite its ubiquity, there are no data on the habits of this species (which, in any case, would not be identifiable in ecological papers) apart from the terse ‘ground forager’ found on a couple of labels, but it seems safe to assume it is a general scavenger of small carrion and vegetative matter.

#### Etymology.

Late Latin *inconspicuus* (‘not conspicuous’); adjective in the nominative case.

**Figure 60.**
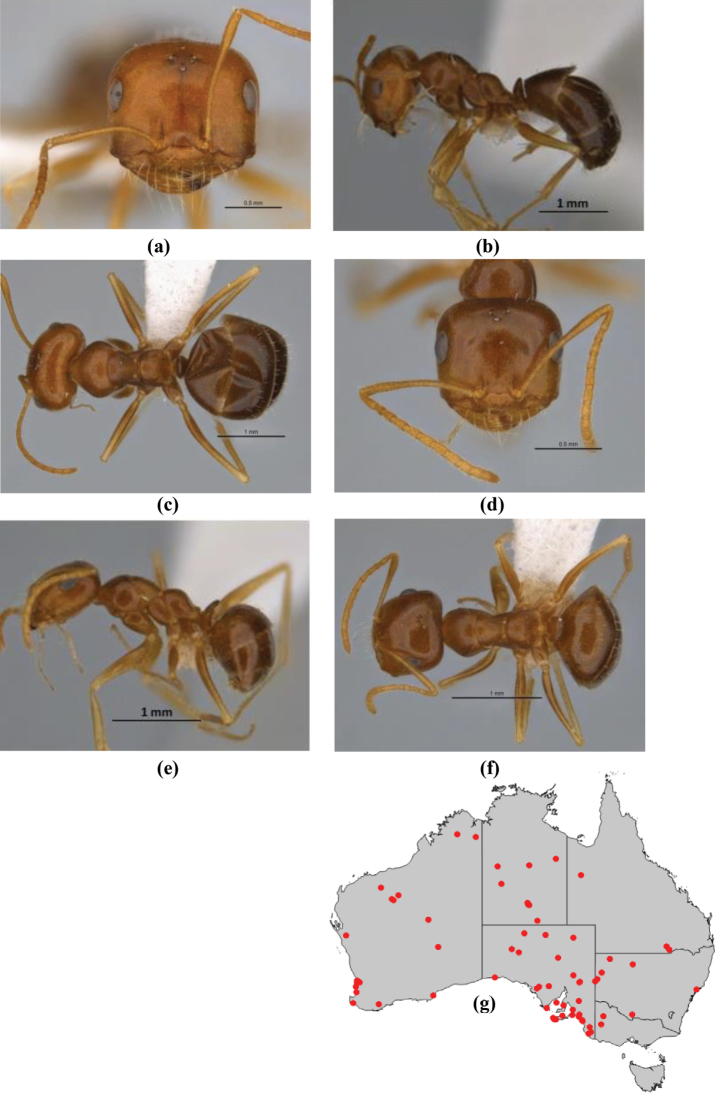
*Melophorus inconspicuus* sp. n.: major worker paratype (ANIC32-006700–top ant) frons (**a**), profile (**b**) and dorsum (**c**); minor worker holotype (ANIC32-006700–bottom ant) frons (**d**), profile (**e**) and dorsum (**f**); distribution map for the species (**g**). Low resolution scale bars: 1 mm (**c, f**); 0.5 mm (**a, d**).

### Melophorus isaiah

Taxon classificationAnimaliaHymenopteraFormicidae

Heterick, Castalanelli & Shattuck sp. n.

http://zoobank.org/345F11D2-3336-4D57-A75E-C9DA444CFE60

#### Types.

Holotype minor worker (bottom ant) from c. 9 km E of Finke, Northern Territory, 30 September 1972, J.E. Feehan, ANIC Ants Vial 15.156 [ANIC32-900184] (ANIC). Paratypes: major worker on same pin and with same details as holotype (ANIC); major worker and two minor workers from Bopeechee Siding, South Australia, 24 July 1956, RSM [*sic*], QHD3 A97, ‘*Melophorus fieldi* Forel Det. J. J. McAreavey 22 November 1957’ [although apparently belonging to the same complex, the two taxa differ in a number of subtle characters, including the head shape, mesosoma and pilosity-BEH] (BMNH); major worker and two minor workers from Bopeechee Siding, South Australia, 24 July 1956, RSM [*sic*], QHD3 A97 [ANIC32-900058] (MCZ); minor worker from Lake Eyre South, South Australia, 23 September 1980, H. Heatwole P.J.M. Greenslade, (12) (SAM).

#### Other material examined.

**Western Australia**: St. Ives GM via Kambalda (Curtin Students [JDM32-001803]).

#### Diagnosis.

*Melophorus isaiah* can be placed in the *M. biroi* species-group on the basis of characters of the clypeus, propodeum, mandible and palps. The species is also placed in the *M. fieldi* species-complex because of the appearance of the anteriorly placed clypeal psammophore, the compact propodeum, the presence of more than one preapical spine on the metatibia, at least in the major worker, the long, even spindly legs, and the unmodified mandible in the major worker. In full-face view, the eye of all workers is placed very high on the head, and slightly above an imaginary horizontal line separating the head (excluding the mandibles) into equal upper and lower sectors. The high placement of the eyes, especially in the minor worker where they occur at the junction of the vertex and the genae, sets this species apart from all other *Melophorus* within the *M. fieldi* species-complex.

#### Minor worker description.

**Head.** Head square; posterior margin of head planar or weakly convex; frons shining with superficial shagreenation or microreticulation only; frons consisting exclusively or almost exclusively of well-spaced, appressed setae only (small, erect setae, if present, usually confined to ocular triangle or posterior margin of head). Eye moderate (eye length 0.20–0.49 length of side of head capsule); in full-face view, eyes set above midpoint of head capsule; in profile, eye set anteriad of midline of head capsule; eyes elliptical or slightly reniform. In full-face view, frontal carinae concave; frontal lobes straight in front of antennal insertion. Anteromedial clypeal margin broadly and evenly convex; clypeal psammophore set at or above midpoint of clypeus; palp formula 6,4. Five mandibular teeth in minor worker; mandibles triangular, weakly incurved; third mandibular tooth distinctly shorter than apical tooth and teeth numbers two and four; masticatory margin of mandibles approximately vertical or weakly oblique. **Mesosoma.** Integument of pronotum, mesonotum and mesopleuron shining and mainly smooth, vestigial shagreenation most noticeable on humeri and mesopleuron; anterior mesosoma in profile convex anteriad, mesonotum often slightly overlapping pronotum, mesosoma planar or slightly sinuate posteriad; erect pronotal setae absent; in profile, metanotal groove shallow, indicated mainly by an angle; propodeum shining and microreticulate; propodeum always smoothly rounded; propodeal dorsum and declivity confluent; erect propodeal setae always absent; appressed propodeal setulae short, separated by more than own length and inconspicuous; propodeal spiracle situated at least twice its width from the declivitous face of propodeum, and shorter (length < 0.50 × height of propodeum), or situated on or beside declivitous face of propodeum, and shorter (length < 0.50 × height of propodeum). **Petiole.** In profile, petiolar node squamiform; in full-face view, shape of petiolar node uniformly rounded; node shining and faintly striolate and microreticulate. **Gaster.** Gaster shining, shagreenate (‘LP record’ appearance); pilosity of first gastral tergite consisting of well-spaced, erect and semi-erect setae interspersed with regularly placed appressed setae, or consisting of well-spaced short, inconspicuous, appressed setae, erect setae (present in at least some workers) confined to margin of sclerite. **General characters.** Colour brown (gaster usually darker) with legs that are pale yellowish and depigmented distally.

#### Major worker description.

**Head.** Head square; posterior margin of head planar or weakly concave; cuticle of frons shining with superficial shagreenation or microreticulation only; pilosity of frons a mixture of a few well-spaced, erect setae interspersed with appressed setae only, or consisting exclusively or almost exclusively of well-spaced, appressed setae only (small, erect setae, if present, usually confined to ocular triangle or posterior margin of head). Eye moderate (eye length 0.20–0.49 length of head capsule); in full-face view, eyes set above midpoint of head capsule; in profile, eye set anteriad of midline of head capsule; eyes elliptical. In full-face view, frontal carinae straight, divergent posteriad; frontal lobes straight in front of antennal insertion. Anterior clypeal margin broadly convex with anteromedial dimple; clypeal psammophore set at or above midpoint of clypeus; palp formula 6,4. Five mandibular teeth in major worker; mandibles triangular, weakly incurved; third mandibular tooth distinctly shorter than apical tooth and teeth numbers two and 4; masticatory margin of mandibles approximately aligned vertically or weakly oblique. **Mesosoma.** Integument of pronotum, mesonotum and mesopleuron shining with very superficial microreticulation, entire lower mesopleuron distinctly shagreenate; anterior mesosoma in profile convex anteriad, mesonotum overlapping pronotum, planar or slightly sinuate posteriad; erect pronotal setae short and unmodified, or weakly expanded distally, or erect pronotal setae absent; in profile, metanotal groove shallow, broadly V- or U-shaped; propodeum shining and microreticulate; propodeum angulate, propodeal angle blunt; length ratio of propodeal dorsum to its declivity between 1:1 and 1:2; erect propodeal setae present and sparse to moderate (1-12), or variable in number, may be absent; appressed propodeal setae short, separated by more than own length and inconspicuous; propodeal spiracle situated on or beside declivitous face of propodeum, and shorter (length less than 0.50 × height of propodeum). **Petiole.** In profile, petiolar node squamiform; in full-face view, shape of petiolar node tapered with blunt vertex; node shining and faintly shagreenate-microreticulate. **Gaster.** Gaster shining with superficial microreticulation, or shining, shagreenate (‘LP record’ appearance); pilosity of first gastral tergite consisting of well-spaced, erect and semi-erect setae interspersed with regularly spaced appressed setae. **General characters.** Colour of head orange tan or light brown, mesosoma brown, gaster dark brown, legs pale yellowish.

#### Measurements.

Worker (n = 4): CI 102–118; EI 19–31; EL 0.19–0.28; HL 0.60–1.26; HW 0.62–1.48; ML 0.86–1.50; MTL 0.61–1.05; PpH 0.08–0.15; PpL 0.36–0.63; SI 75–125; SL 0.77–1.11.

#### Comments.

The facetious scientific name proposed here reflects the relatively high placement of the eyes on the head capsule, which, together with its long palps, are sufficient to identify the ant. Specimens have been collected in remote locations in the NT, SA and WA, and this may account for the infrequency with which it appears in collections. No specimens were available for sequencing and nothing is known of its preferred habitat or its habits.

#### Etymology.

A pun on the high placement of the eyes on the head capsule; noun in the nominative singular used without declension.

**Figure 61.**
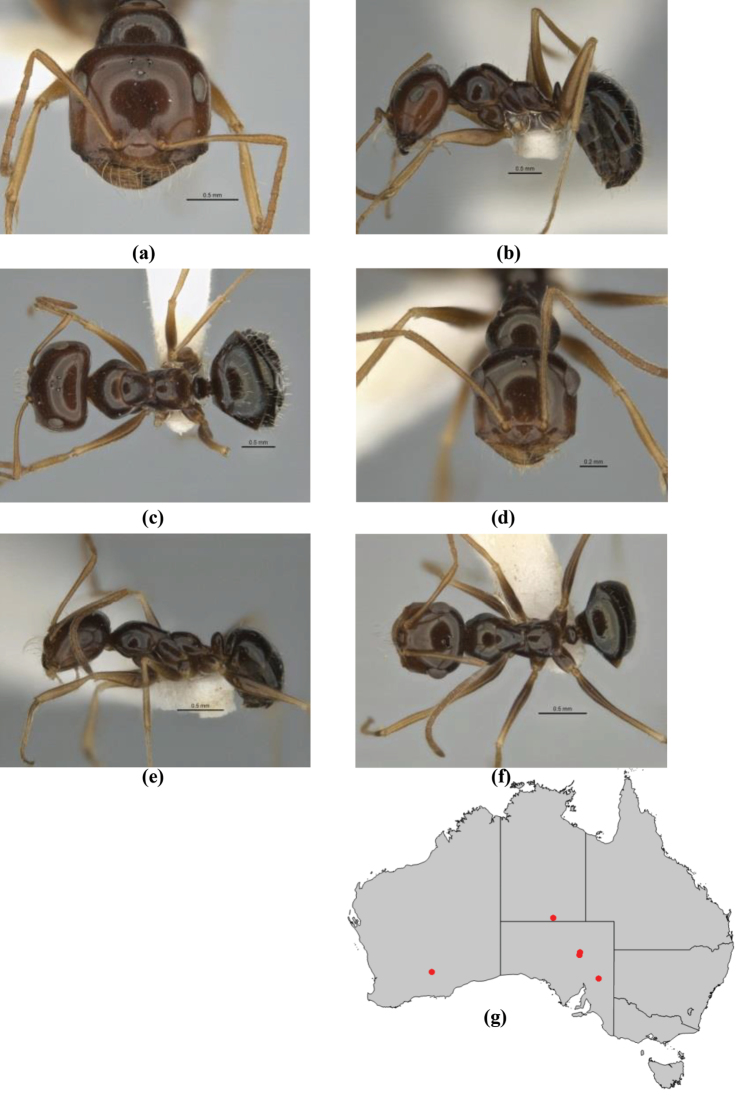
*Melophorus isaiah* sp. n.: major worker paratype (ANIC32-900184–top ant) frons (**a**), profile (**b**) and dorsum (**c**); minor worker holotype (ANIC32-900184–bottom ant) frons (**d**), profile (**e**) and dorsum (**f**); distribution map for the species (**g**). Low resolution scale bars: 0.5 mm (**a–c, e, f**); 0.2 mm (**d**).

### Melophorus lanuginosus

Taxon classificationAnimaliaHymenopteraFormicidae

Heterick, Castalanelli & Shattuck sp. n.

http://zoobank.org/7460E297-94EA-4D2A-BC51-37E201164643

#### Types.

Holotype minor worker (bottom ant) from Fowler’s Gap, New South Wales, 19 November 1979, (8) [ANIC32-900145] (ANIC). Paratypes: 2 major workers on same pin and with same details as holotype (ANIC); major and media worker from Kunoth Paddock, near Alice Springs, Northern Territory, 3-6 March 1975, P.J.M. Greenslade, Traps Hills, 5 (BMNH); major worker and media worker from Kunoth Paddock, near Alice Springs, Northern Territory, 26 October 1974, P.J.M. Greenslade, Traps Hills, 11 (MCZ); major worker and 2 minor workers from 17 km SE of Oodnadatta, South Australia, 24 September 1972, J.E. Feehan (SAM); major worker, media worker and 2 minor workers from Coorow 29°56'S, 116°03'E, Western Australia, 2 October 1999, B.E. Heterick, white sand [JDM32-001806] (WAM); major worker, 2 media workers and minor worker from Ethel Creek, Western Australia, 1993-4, P.A. Varris, nest 12, *Melophorus* sp. A [JDM32-001794] (WAM).

#### Other material examined.

**New South Wales**: Fowlers Gap (Greenslade, P.J.M.). **Northern Territory**: Kunoth Paddock, near Alice Springs (Greenslade, P.J.M.), Kunoth Paddock, near Alice Springs (Greenslade, P.J.M.). **South Australia**: Burnt Camp Dam, Calperum (Medlin, G. C.). **Western Australia**: 0.9 km along Tanami Rd (Heterick, B.E. [M206/M207/M208]), 18.5 km E Southern Cross (Heterick, B.E. [M132/M133/M134]), Dunham River (rest area) (Heterick, B.E. [M214/M215]), Marillana Ck. (Dunlop, N. [JDM32-001823]), Miling (Heterick, B.E. [M302/M303]), Panawonica [Pannawonica] (Brown, K. [JDM32-001827]), Pilbara Locality (collector unknown [JDM32-001812]).

#### Diagnosis.

*Melophorus lanuginosus* can be placed in the *M. biroi* species-group on the basis of characters of the clypeus, propodeum, mandible and palps. The species is also placed in the *M. fieldi* species-complex because of the appearance of the anteriorly placed clypeal psammophore, the compact propodeum, the presence of more than one preapical spine on the metatibia, at least in the major worker, the long, even spindly legs and the unmodified mandible in the major worker. In general, *Melophorus lanuginosus* is rendered distinctive by a combination of its long white setae, which form a thatch on the gaster and on the pronotum, the long erect setae on the mesosoma (longer setae > diameter of eye); cuticle with strong shagreenate sculpture, matt or with weak sheen and a relatively thick petiolar node in the minor worker (to about 0.6× as wide as high). Neither is the petiolar node a true scale in the major worker. In most specimens the anteromedial dimple is prominent and protrudes as a V-shaped lip. These features combined are found only in this species. In addition to these features, the “*pillipes*” form has whorls of fine, straight erect setae on the legs and antennal scape. A second variant found at a couple of sites in NW Australia has aberrant major and media workers. These possess a short, protrusive flange on the midline of the capsule between the frontal carinae, and frons adjacent to the frontal carinae is recessed as in the *M. mjobergi* clade major workers. Additionally in these subcastes, the torulus is bizarrely produced as a sleeve to the base of the antennal scape and its pedicel. Apart from these particular features, these two variant conform to the diagnosis of the typical *M. lanuginosus*.

#### Minor worker description.

**Head.** Head square; posterior margin of head planar or weakly convex; frons shining with superficial shagreenation or microreticulation only, or matt or with weak sheen, microreticulate or microreticulate-shagreenate; pilosity of frons a mixture of short, erect and semi-erect setae interspersed with shorter decumbent setae and well-spaced, short, appressed setae. Eye moderate (eye length 0.20–0.49 length of side of head capsule); in full-face view, eyes set above midpoint of head capsule, or set at about midpoint of head capsule; in profile, eye set anteriad of head capsule, or set around midline of head capsule; eyes elliptical or slightly reniform. In full-face view, frontal carinae distinctly concave; frontal lobes straight in front of antennal insertion. Anteromedial clypeal margin broadly convex with anteromedial dimple; clypeal psammophore set at or above midpoint of clypeus; palp formula 6,4. Five mandibular teeth in minor worker; mandibles triangular, weakly incurved; third mandibular tooth distinctly shorter than apical tooth and teeth numbers two and four; masticatory margin of mandibles approximately vertical or weakly oblique. **Mesosoma.** Integument of pronotum, mesonotum and mesopleuron with weak to moderate sheen and superficial microreticulation (more pronounced on mesopleuron); anterior mesosoma in profile broadly convex; appearance of erect pronotal setae long (i.e., longest erect setae longer than length of eye) and unmodified; in profile, metanotal groove shallow, indicated mainly by an angle; propodeum matt or with a weak sheen and indistinctly shagreenate; propodeum angulate, propodeal angle blunt; length ratio of propodeal dorsum to its declivity about 4:3, or about 1:1; erect propodeal setae present and abundant (greater than 12); appressed propodeal setulae long and closely aligned, creating pubescence, or long, each reaching setae behind and in front, but not forming pubescence; propodeal spiracle situated at least twice its width from the declivitous face of propodeum, and shorter (length < 0.50 × height of propodeum), or situated on or beside declivitous face of propodeum, and shorter (length < 0.50 × height of propodeum). **Petiole.** In profile, petiolar node squamiform, or subcuboidal, vertex bluntly rounded; in full-face view, shape of petiolar node uniformly rounded, or tapered with blunt vertex; node shining and smooth with vestigial sculpture, or matt and microreticulate. **Gaster.** Gaster weakly shining with indistinct shagreenation, or shining, shagreenate (‘LP record’ appearance); pilosity of first gastral tergite consisting of a mixture of curved, erect and semi-erect setae and decumbent and appressed setae that form a variable pubescence. **General characters.** Colour various shades of orange or brown, usually concolorous but gaster may be darker.

#### Major worker description.

**Head.** Head horizontally rectangular, broader than wide; posterior margin of head planar or weakly concave; cuticle of frons matt or with weak sheen, indistinctly shagreenate; pilosity of frons a mixture of well-spaced, distinctly longer erect and semi-erect setae interspersed with shorter decumbent setae. Eye moderate (eye length 0.20–0.49 length of head capsule); in full-face view, eyes set above midpoint of head capsule; in profile, eye set anteriad of midline of head capsule; eyes elliptical. In full-face view, frontal carinae straight or weakly convex; frontal lobes curved inward in front of antennal insertion. Anterior clypeal margin broadly convex with anteromedial dimple; clypeal psammophore set at or above midpoint of clypeus; palp formula 6,4. Five mandibular teeth in major worker; mandibles triangular, weakly incurved; third mandibular tooth distinctly shorter than apical tooth and teeth numbers two and four; masticatory margin of mandibles approximately aligned vertically or weakly oblique. **Mesosoma.** Integument of pronotum, mesonotum and mesopleuron moderately shining and shagreenate throughout; anterior mesosoma in profile broadly convex; erect pronotal setae short, (i.e., shorter than length of eye) and unmodified; in profile, metanotal groove shallow, broadly V- or U-shaped; propodeum matt or with weak sheen and microreticulate-striolate; propodeum angulate, propodeal angle blunt; length ratio of propodeal dorsum to its declivity between 1:1 and1:2; erect propodeal setae present and abundant (at least a dozen); appressed propodeal setae long and separated by at least own length; propodeal spiracle situated on or beside declivitous face of propodeum, and shorter (length less than 0.50 × height of propodeum). **Petiole.** In profile, petiolar node squamiform; in full-face view, shape of petiolar node uniformly rounded, or tapered with blunt vertex; node shining and faintly shagreenate-microreticulate. **Gaster.** Gaster weakly shining with indistinct shagreenation; pilosity of first gastral tergite consisting of thick, appressed setae that form pubescence, interspersed with numerous short, bristly, erect setae. **General characters.** Colour of foreparts various shades of light tan with brown gaster.

#### Measurements.

Worker (n = 6): CI 103–116; EI 21–31; EL 0.20–0.35; HL 0.61–1.45; HW 0.62–1.69; ML 0.92–1.22; MTL 0.67–1.22; PpH 0.10–0.19; PpL 0.37–0.65; SI 72–130; SL 0.81–1.22.

#### Comments.

In appearance this very hairy member of the *M. fieldi* complex most closely resembles *M. hirsutipes*, but the presence of long, silvery, overlapping appressed setae on the pronotum and first gastral segment and the narrower, more porrect petiolar node help to distinguish this ant from the former. The anteromedian clypeal prominence is strongly developed in many populations of this species. Despite its morphological similarity to *M. hirsutipes*, *M. lanuginosus* appears to be most closely related to *M. longipes*, and forms a sister relationship to that species on a five-gene tree – but see ‘Comments’ for the latter.

A small number of samples of workers of all subcastes from Packsaddle in the Pilbara, WA, reveal a strange development of the torulus. The torulus is merely produced as a short stalk in minor and media workers but in the major worker the lobes of the frontal carinae have become raised and invaginated to form ragged, projecting narrow sleeves to which the torulus is fused. The torulus further protrudes to surround the antennal condyle. This is analogous to the situation in *M. hirsutus*. The area around the frontal carinae is also recessed in the major worker. The end result is an ant of hybrid appearance that is not dissimilar in regard to its cephalic morphology to *M. mjobergi* or *M. postlei*. In addition, major and media workers, but not the minor workers, have a protruding flange that juts from the midline of the head within the frontal triangle (this area generally appears as a smooth, shining, unsculptured band in most *Melophorus*). The major also has a slightly more hunched appearance than the major workers of normal *M. lanuginosus*. In all other respects of their morphology, however, these workers resemble normal workers of *M. lanuginosus*. The reasons for this strange appearance are unknown and can only be guessed at. Hybridization with a species in the *M. mjobergi* complex seems unlikely because of their lack of relatedness to *M. lanuginosus*.

The species is well-distributed throughout drier inland habitats in NSW, NT, SA and WA, but does not appear to occur in QLD or Vic. In Southern Cross, WA, specimens have been taken in heathland on top of yellow sand, and from a lawn in the little WA wheatbelt town of Miling. Two workers were collected from a dune in Burnt Camp Dam, SA (SAM). No other meaningful data are available. In the field, this species appears similar to *M. turneri*, but is less shiny.

#### Etymology.

Latin *lanuginosus* (‘woolly’ or ‘downy’); adjective in the nominative singular.

**Figure 62.**
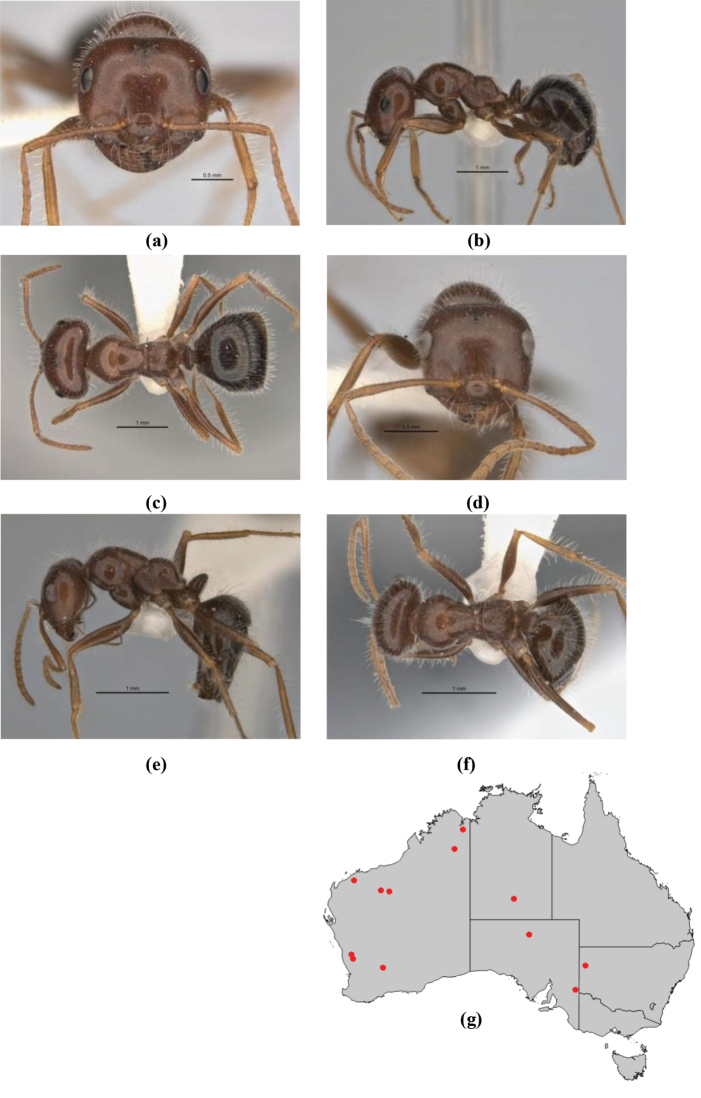
*Melophorus lanuginosus* sp. n.: major worker paratype (ANIC32-900145–top ant) frons (**a**), profile (**b**) and dorsum (**c**); minor worker holotype (ANIC32-900145–bottom ant) frons (**d**), profile (**e**) and dorsum (**f**); distribution map for the species (**g**). Low resolution scale bars: 1 mm (**b, c**, **e, f**); 0.5 mm (**a, d**).

### Melophorus longipes

Taxon classificationAnimaliaHymenopteraFormicidae

Heterick, Castalanelli & Shattuck sp. n.

http://zoobank.org/4411EB77-2D94-49D5-ADD4-0E001C26CB45

#### Types.

Holotype minor worker (bottom ant) From 10 km from Mt Ive Homestead, South Australia, 22 October 1980, P.J.M. Greenslade, B Se Gawler Ranges, South Australia, 16 (ANIC). Paratypes: 2 major workers on same pin and with same details as holotype (ANIC); 3 major workers from 85 km W Mabel Creek, South Australia, 9 October 1980, P.J.M. Greenslade, Sa EAD; (Top major worker photographed) [ANIC32-900193] (ANIC); major and 2 minor workers from 14 km S by W of Beltana, 30°56'S, 138°23'E South Australia, 14 September 1972, J.E. Feehan, ANIC Ants Vial 16.99 (MCZ); major and minor worker from Poochera 32°43'S, 134°50'E, Australia, 10-13.xi.1981, R.W. Taylor and R.J. Bartel, ‘Freightline site’ grassy areas not under trees, ANIC Ants Vial 1.167 (BMNH); media and minor worker from Vokes Hill, Victoria Desert, South Australia, 2 October 1976, P.J.M. Greenslade, (6) a, 8 (SAM); 2 majors and a minor worker from 19 miles SE by S of Karonie, 31°12'S, 122°42'E, Western Australia, 9 Nov. 1969, Key’s field notes. Trip 163. Stop 19392.9 (WAM); major and minor worker from Woolgangie, Western Australia, 18 September 1988, B. Heterick, soil, native veg., rural environ., 433, 8MelBH7C (WAM).

#### Other material examined.

**Northern Territory**: about 50 km E Finke (Feehan, J.E.). **South Australia**: 10 km E Mt Ive Homestead, Gawler Ranges (Greenslade, P.J.M.), 10 km E Mt Ive Homestead, Gawler Ranges (Greenslade, P.J.M. [ANIC32-900192]), 1 mi S Victory Downs Homestead (McInnes & Dowse), 20 mi W Mt Whinham (McInnes, R. & Dowse, J.), 25 mi NE Maralinga (McInnes & Dowse), 29 km ENE Yantanabie (Taylor, R.W., Bartell, R.J. & Lowery, B.B.), 34 mi NW Woomera (Greaves, T.), 3 km NE homestead, Koonamore (Greenslade, P.J.M.), 3 mi S Mt Morris, Musgrave Range (McInnes & Dowse), 5 mi NW Coffin Hill (McInnes & Dowse), Blyth, 4 mi W Clare (Lowery, B.B.), Cambrai (Greenslade, P.J.M.), Cambrai (Greenslade, P.J.M.), Cambrai (Greenslade, P.J.M.), Eyre Highway, 38 km W Kimba (Heterick, B.E.), Eyre Hwy, 9.7 km NE Cootra (Heterick, B.E. [M327]), Gawler Ranges (Greenslade, P.J.M.), Gawler Ranges (Greenslade, P.J.M.), Poochera (Taylor, R.W. & Bartell, R.J.), 50 km E Vokes Hill, Victoria Desert (Greenslade, P.J.M.). **Western Australia**: 1 km W Canna (Heterick, B.E. [M318]), 1.5 km S Koolyanobbing (Heterick, B.E. [M60]), 16 km S Mt Magnet (Heterick, B.E. [M291/M292]), 19 km N Hines Hill (Heterick, B.E. [M05/M22/M33/M34]), 19 mi SEbyS Karonie (Taylor, R.W.), 20 mi SW Mundiwindi (McInnes & Dowse), 23 mi SWbyS Coolgardie (Taylor, R.W.), 26 mi NWbyW Norseman (Taylor, R.W.), 6 mi W Karonie (Greaves, T.), 8 km N Bullfinch (Heterick, B.E. [M124]), 9 mi W Zanthus (Taylor, R.W.), Beebyn Stn. (Heterick, B.E. [JDM32-001783]), Coomallo Downs (Heterick, B.E. [M03/M42]), Dedari, 320 mi E Perth (Douglas, A.), Ethel Creek (Varris, P.A. [JDM32-001793]), Junana Rock, 9 km NW Mt. Ragged (Taylor, R.W.), Kadji Lake Rd. (Heterick, B.E. [JDM32-001795]), Kalgoolie (Greaves, T.), Mardathuna Rd turnoff (Heterick, B.E. [M23/M56]), Mulga, NE Goldfields (Pringle, H.J.R. [ANIC32-029564]), Port Hedland (Weatherill, L.).

#### Diagnosis.

*Melophorus longipes* can be placed in the *M. biroi* species-group on the basis of characters of the clypeus, propodeum, mandible and palps. The species is also placed in the *M. fieldi* species-complex because of the appearance of the anteriorly placed clypeal psammophore, the compact propodeum, the presence of more than one preapical spine on the metatibia, at least in the major worker, the long, even spindly legs and the unmodified mandible in the major worker. *Melophorus longipes* can be confused with several very similar and highly derived *Melophorus* that are also common and widespread, and specimens have to be checked carefully using the following characters before they can be distinguished from *M. eumorphus*, *M. turneri* and *M. vitreus*: *Melophorus longipes* can be distinguished from M. *eumorphus* by its larger size (minor worker HW 0.57 mm ≥; major worker HW > 1.50 mm); and less strongly sculptured mesopleuron. In turn, the *Melophorus longipes* minor workers lack the protuberant clypeus seen in *M. vitreus* minors, the clypeus being only weakly protuberant and usually with a distinct dimple at its anterior midpoint, are less gracile, have a squamiform petiolar node and do not exhibit the “*pillipes*” condition (which is general in *M. vitreus*). It is with many populations of *Melophorus turneri* that *Melophorus longipes* can be most easily confused. However, in *M. longipes* the metafemur of the minor worker is longer and attenuated towards its junction with the tibia (metafemur ≥ 0.90 × length of mesosoma) compared with the shorter, stouter metafemur in the *M. turneri* minor (metafemur ≤ 0.75 × length of mesosoma). In profile, the dorsum of the minor worker propodeum is smoothly curved on to its declivitous face in *M. longipes* whereas there is often a distinct angle in the case of *M. turneri*. Major workers are more difficult to differentiate. However, the major worker mesonotum in *M. longipes* is flat to weakly convex versus weakly to moderately convex in *M. turneri* major workers, the *M. longipes* major worker metafemur is increasingly depigmented towards its articulation with the tibia, and the tibia is depigmented yellowish-white. The appearance of the major worker metafemur in *M. turneri* workers seen is always uniform and similar in colour pattern to the tibia, both parts being ochraceous to yellowish.

#### Minor worker description.

**Head **. Head square; posterior margin of head planar or weakly convex; frons shining with superficial shagreenation or microreticulation only; pilosity of frons a mixture of a few well-spaced, erect setae interspersed with appressed setae only; Eye moderate (eye length 0.20–0.49 length of side of head capsule); in full-face view, eyes set at about midpoint of head capsule; in profile, eye set anteriad of midline of head capsule; eyes elliptical or slightly reniform. In full-face view, frontal carinae straight, divergent posteriad; frontal lobes straight in front of antennal insertion. Anteromedial clypeal margin broadly convex with anteromedial clypeal dimple; clypeal psammophore set at or above midpoint of clypeus; palp formula 6,4. Five mandibular teeth in major worker; mandibles triangular, weakly incurved; third mandibular tooth distinctly shorter than apical tooth and tooth numbers two and four; masticatory margin of mandibles vertical or weakly oblique. **Mesosoma.** Integument of pronotum, mesonotum and mesopleuron with weak to moderate sheen and superficial microreticulation (more pronounced on mesopleuron); anterior mesosoma broadly convex; appearance of erect pronotal setae short, (i.e., longest erect setae shorter than length of eye) and unmodified; in profile, metanotal groove shallow, broadly V or U-shaped; propodeum matt or with a weak sheen and microreticulate-striolate; propodeum smoothly rounded or with indistinct angle; propodeal dorsum and declivity confluent; erect propodeal setae few in number, may be absent; appressed propodeal setae short, separated by more than own length and inconspicuous; propodeal spiracle situated on or beside declivitous face of propodeum, and shorter (length < 0.50 × height of propodeum). **Petiole.** In profile, petiolar node squamiform to narrowly conical, vertex bluntly rounded; in full-face view, petiolar node tapered with blunt vertex; node shining and smooth throughout. **Gaster.** Gaster shining, shagreenate (‘LP record’ appearance); pilosity of first gastral tergite consisting of well-spaced erect and semi-erect setae interspersed with regularly placed appressed setae. **General characters.** Body tan with a brown gaster and the legs a light tan, becoming pale yellow distally.

#### Major worker description.

**Head **. Head square; posterior margin of head planar or weakly convex; frons shining with superficial shagreenation or microreticulation only; pilosity of frons a mixture of a few well-spaced, erect setae interspersed with appressed setae only; Eye moderate (eye length 0.20–0.49 length of side of head capsule); in full-face view, eyes set above midpoint of head capsule; in profile, eye set anteriad of midline of head capsule; eyes elliptical or slightly reniform. In full-face view, frontal carinae straight, divergent posteriad; frontal lobes straight in front of antennal insertion. Anteromedial clypeal margin broadly convex with anteromedial clypeal dimple; clypeal psammophore set at or above midpoint of clypeus; palp formula 6,4. Five mandibular teeth in major worker; mandibles triangular, weakly incurved; third mandibular tooth distinctly shorter than apical tooth and tooth numbers two and four; masticatory margin of mandibles vertical or weakly oblique. **Mesosoma.** Integument of pronotum, mesonotum and mesopleuron with weak to moderate sheen and superficial microreticulation (more pronounced on mesopleuron); anterior mesosoma broadly convex; appearance of erect pronotal setae short, (i.e., longest erect setae shorter than length of eye) and unmodified; in profile, metanotal groove shallow, broadly V or U-shaped; propodeum matt or with a weak sheen and microreticulate-striolate; propodeum smoothly rounded or with indistinct angle; propodeal dorsum and declivity confluent; erect propodeal setae present and abundant (greater than 12); appressed propodeal setae short, separated by more than own length and inconspicuous; propodeal spiracle situated on or beside declivitous face of propodeum, and shorter (length < 0.50 × height of propodeum). **Petiole.** In profile, petiolar node squamiform; in full-face view, node uniformly rounded or tapered with blunt vertex; node shining with vestigial sculpture. **Gaster.** Gaster shining, shagreenate (‘LP record’ appearance); pilosity of first gastral tergite consisting of well-spaced erect and semi-erect setae interspersed with regularly placed appressed setae). **General characters.** Body tan with a brown gaster and the legs an increasingly pale yellowish, becoming depigmented distally.

#### Measurements.

Worker (n = 10): CI 105–124; EI 18–33; EL 021–0.30; HL 0.61- 1.38; HW 0.64–1.70; ML 0.87–1.70; MTL 0.62–1.10; PpH 0.11–0.17; PpL 0.36–0.70; SI 69–118; SL 0.75–1.17.

#### Comments.

This species shares with *M. turneri* and *M. vitreus* a generally glabrous mesosoma with well-spaced, tiny appressed setae on the first gastral tergite. The ant can readily be separated from *M. vitreus* by its smaller eye and more squamiform node in the minor worker. In common with many *Melophorus*, major workers of this species are nondescript and easily mistaken for the majors of *M. turneri*, the easiest way to distinguish them being the light coloured, depigmented tibia and distal femora when compared with *M. turneri* major workers (which have tawny yellow or orange limbs). Distinguishing minor and media workers of *M. longipes* from *M. turneri* requires examination of the metatibia, which is measurably longer or at least narrower in individuals of the same size within the two species (total range 0.61-1.10 mm in *M. longipes* compared with 0.61-0.98 mm in *M. turneri*). In other respects, the two ants are virtually identical. *Melophorus longipes* records are restricted to SA and WA with just one NT record (50 km E Finke River). Despite its close physical similarity to *M. turneri*, the sister species of *M. longipes* is unclear, based on various gene trees. Populations of this species are closely related, and cluster tightly together on all individual gene trees. Also, unlike a number of other members of the *M. fieldi* complex, the ‘*pillipes*’ condition has not been seen in *M. longipes*.

Although both *M. longipes* and *M. turneri* are often sympatric, most records of *M. longipes* have come from inland locations well away from the coast. All vegetation records mention mallee, and this is therefore assumed to be the favoured habitat for this species. There are also several records of dunes and one of a salt lake and sand ridge. No data on the behaviour of the ant are available, but it is probably a generalist, given its distribution and morphology.

#### Etymology.

Latin *longus* (‘long’) plus *pes* (‘foot’); adjective in the nominative singular.

**Figure 63.**
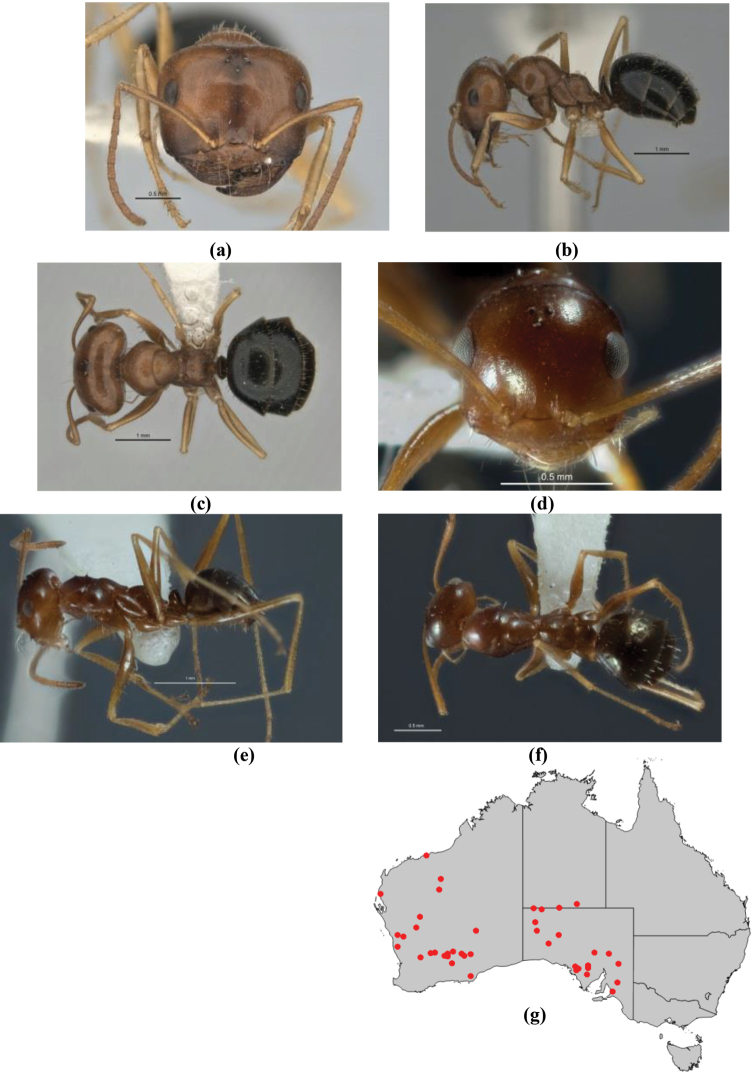
*Melophorus longipes* sp. n.: major worker paratype (ANIC32-900193–top ant) frons (**a**), profile (**b**) and dorsum (**c**); minor worker holotype ([ANIC] Mt Ive. Homestead, SA, 22 Oct. 1880, P.J.M. Greenslad**e–b**ottom ant) frons (**d**), profile (**e**) and dorsum (**f**); distribution map for the species (**g**). Low resolution scale bars: 1 mm (**b, c, e**); 0.5 mm (**a, d, f**).

### Melophorus major

Taxon classificationAnimaliaHymenopteraFormicidae

Forel Status novus

Melophorus fieldi var. major Forel 1915: 87.

#### Types.

Syntype major worker and minor workers: major and minor worker on separate pins, Kimberley district, Western Australia [MHNG]. Also examined: MHNG, MSNG, NHRS AntWeb images of specimens (MHNG specimens CASENT0909812, CASENT0909813, MSNG specimen CASENT0905126, NHRS specimen NHRS-HEVA00003949).

#### Other material examined.

**New South Wales**: Moree (I. Oliver). **Western Australia**: 11 km N Wiluna (Davidson, D. & Morton, S.), 11 km N Wiluna (Davidson, D. & Morton, S. [ANIC32-066606]), 11 km N Wiluna (Davidson, D. & Morton, S. [ANIC32-066608]).

#### Diagnosis.

*Melophorus major* can be placed in the *M. biroi* species-group on the basis of characters of the clypeus, propodeum, mandible and palps. The species is also placed in the *M. fieldi* species-complex because of the appearance of the anteriorly placed clypeal psammophore, the compact propodeum, the presence of more than one preapical spine on the metatibia, at least in the major worker, the long, even spindly legs, and the unmodified mandible in the major worker. The large-eyed *Melophorus major* closely resembles several related *Melophorus*, most obviously *M. bruneus*, *M. fieldi* and the *M. turneri candidus* form of *M. turneri*. The one distinctive feature that places at least the *M. major* minor worker apart from other *Melophorus* in its species-complex is the low placement of the clypeal psammophore: in profile, this structure is to be found just behind the anterior clypeal margin and it is also placed well below the midpoint of the clypeus in some major workers. At present the few known major workers cannot be separated with any confidence from related species or populations of species with an anteriorly situated psammophore, but the propodeum is narrow with an oblique dorsal face and the colour of major workers seen is shining brown with the gaster slightly darker, and this character may be helpful in some cases.

#### Minor worker description.

**Head.** Head square; posterior margin of head planar or weakly convex; frons shining with superficial shagreenation or microreticulation only; pilosity of frons a mixture of a few well-spaced, erect setae interspersed with appressed setae only. Eye large (eye length ≥ 0.50 × length of side of head capsule), or moderate (eye length 0.20–0.49 length of side of head capsule); in full-face view, eyes set at about midpoint of head capsule; in profile, eye set anteriad of midline of head capsule; eyes elliptical or slightly reniform. In full-face view, frontal carinae distinctly concave; frontal lobes straight in front of antennal insertion. Anteromedial clypeal margin broadly and evenly convex; clypeal psammophore set at or just above anterior clypeal margin; palp formula 6,4. Five mandibular teeth in minor worker; mandibles triangular, weakly incurved; third mandibular tooth distinctly shorter than apical tooth and teeth numbers two and four; masticatory margin of mandibles approximately vertical or weakly oblique. **Mesosoma.** Integument of pronotum, mesonotum and mesopleuron moderately shining and shagreenate throughout; anterior mesosoma in profile broadly convex; appearance of erect pronotal setae short, (i.e., longest erect setae shorter than length of eye) and unmodified; in profile, metanotal groove shallow, broadly V or U-shaped; propodeum shining and finely striolate and microreticulate; propodeum angulate, propodeal angle blunt; length ratio of propodeal dorsum to its declivity greater than 2:1; erect propodeal setae variable in number, may be absent; appressed propodeal setulae short, separated by more than own length and inconspicuous; propodeal spiracle situated on or beside declivitous face of propodeum, and longer (length ≥ 0.50 × height of propodeum), or situated on or beside declivitous face of propodeum, and shorter (length < 0.50 × height of propodeum). **Petiole.** In profile, petiolar node squamiform; in full-face view, shape of petiolar node uniformly rounded; node shining and distinctly shagreenate-microreticulate. **Gaster.** Gaster shining, shagreenate (‘LP record’ appearance); pilosity of first gastral tergite consisting of well-spaced, erect and semi-erect setae interspersed with regularly placed appressed setae. **General characters.** Colour of foreparts brown, gaster blackish-brown.

#### Major worker description.

**Head.** Head square; posterior margin of head weakly concave; cuticle of frons shining with superficial shagreenation or microreticulation only; pilosity of frons a mixture of a few well-spaced, erect setae interspersed with appressed setae only. Eye moderate (eye length 0.20–0.49 length of head capsule); in full-face view, eyes set at about midpoint of head capsule; in profile, eye set anteriad of midline of head capsule; eyes elliptical. In full-face view, frontal carinae straight, divergent posteriad; frontal lobes straight in front of antennal insertion. Anterior clypeal margin broadly and evenly convex; clypeal psammophore set at or just above anterior clypeal margin; palp formula 6,4. Five mandibular teeth in major worker; mandibles triangular, weakly incurved; third mandibular tooth distinctly shorter than apical tooth and teeth numbers two and four; masticatory margin of mandibles approximately aligned vertically or weakly oblique. **Mesosoma.** Integument of pronotum, mesonotum and mesopleuron moderately shining and shagreenate throughout; anterior mesosoma in profile broadly convex; erect pronotal setae short, (i.e., shorter than length of eye) and unmodified; in profile, metanotal groove shallow, indicated mainly by an angle and metathoracic spiracles; propodeum shining and shagreenate; propodeum angulate, propodeal angle blunt; length ratio of propodeal dorsum to its declivity between 1:1 and1:2, or greater than 1:2; erect propodeal setae present and abundant (at least a dozen); appressed propodeal setae long and separated by at least own length; propodeal spiracle situated on or beside declivitous face of propodeum, and longer (length ≥ 0.50 × height of propodeum). **Petiole.** In profile, petiolar node squamiform; in full-face view, shape of petiolar node uniformly rounded; node shining and faintly shagreenate-microreticulate. **Gaster.** Gaster shining with superficial microreticulation; pilosity of first gastral tergite consisting of well-spaced, erect and semi-erect setae interspersed with regularly spaced appressed setae. **General characters.** Colour of foreparts orange tan, gaster blackish-brown.

#### Measurements.

Worker (n = 2): CI 99–112; EI 23–37; EL 0.28–0.37; HL 0.76–1.45; HW 0.75–1.63; ML 1.07–1.71; MTL 0.69–1.08; PpH 0.12–0.16; PpL 0.43–0.71; SI 70–109; SL 0.82–1.14.

#### Comments.

Apart from the low placement of the clypeal psammophore, this medium-sized *Melophorus* is completely unremarkable and can easily be mistaken for *Melophorus inconspicuus* or a dark *M. hirsutipes*, to which it is likely closely related. Only a handful of pinned workers from Wiluna, WA, and one pin of two minor workers from Moree, NSW, were available for analysis. However, ants of similar appearance have regularly turned up in some collections made in the Pilbara region of WA, as part of a survey by large mining companies, and the species may not be uncommon. However, no specimens have been available for sequencing. Because of the difficulty in separating this species (especially major workers) from several morphologically very similar taxa, its range is uncertain but it is likely to occur sparsely in drier areas across the continent in suitable habitat. No ecological data are available.

The name ‘*major*’ is given new species level status in this revision. The syntypes for this species (two separate pins available for specimen analysis and three Automontage photographs on AntWeb) reveal a few small differences from the remaining material; namely, the eye in the non-type material (both major and minor workers) is larger and the erect pilosity is longer and a little more extensive. These differences may be explained by biogeography, the type specimens having been collected in the Kimberley (not shown on the map because of the vagueness of the collection locality), and the others much further south. The media worker available for inspection, however, has the same protuberant clypeus and anteriorly placed clypeal psammophore seen in the non-type minor workers. This feature is distinctive and is not seen in any other member of the *M. fieldi* complex. The major worker does not always share this feature and is difficult to separate from allied species. The dark colouration and short propodeum with an oblique dorsal face (compared with an often blocky, square propodeum in similar species) help in the identification of major workers, but minor workers are needed for identifications to be definitive.

**Figure 64.**
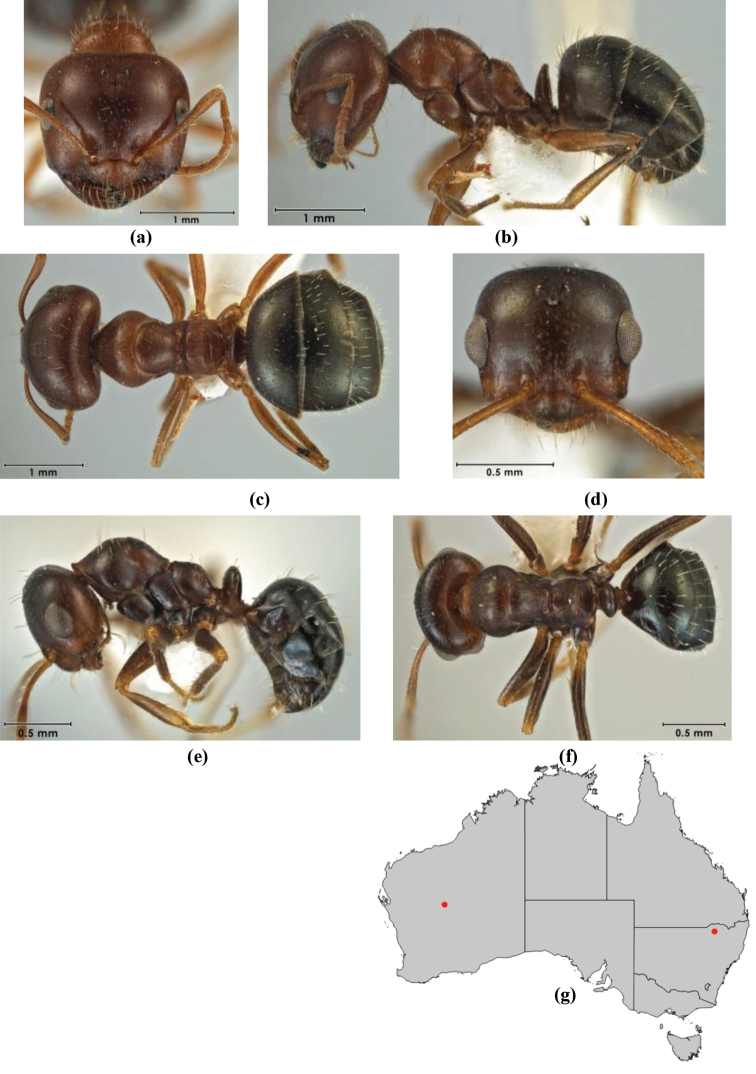
*Melophorus major* Forel: major worker (CASENT0905126) (MSNG) frons (**a**), profile (**b**) and dorsum (**c**); minor worker (NHRS-HEVA 000003949) (NHRS) frons (**d**), profile (**e**) and dorsum (**f**); distribution map for the species (**g**).

### Melophorus microtriches

Taxon classificationAnimaliaHymenopteraFormicidae

Heterick, Castalanelli & Shattuck sp. n.

http://zoobank.org/8C172FC6-1BA0-4CB4-AD70-501A758E283D

#### Types.

Holotype minor worker (bottom ant) from 50 km N of Tarcoola, South Australia, 3 October 1976, P.J.M. Greenslade, (3) [ANIC32-900121] (ANIC). Paratypes: 2 major workers on same pin and with same details as holotype (ANIC); media worker and major worker from 200 km NE of Alice Springs, Northern Territory, 29 May 1985 A.N. Andersen (ANIC); major worker from 76 km W of Mt Aloysius 26.06S, 127.51E, Western Australia, 16 November 1977, J.E. Feehan (ANIC); 3 minor workers from 17 km NW of Alexandria Downs 19.01S, 136.57E, Northern Territory, 13 October 1981, D. Davidson/S. Morton, 158a, lA(?), S (BMNH); major worker and 2 minor workers from 5 km SE of Oodnadatta 27.34S, 135.29E, South Australia, 4 October 1981, D. Davidson/S. Morton, 82A, A1 (MCZ); major worker and media worker from Millstream National Park 21°35'S, 117°36'E, Western Australia, 2 December 1985, P.S. Ward#, 300mm, 8069-5, ground foragers, riparian woodland (QM); major worker and 2 minor workers from 53 km E of Vokes Hill, Victoria Desert, South Australia, 9 October 1976, P.J.M. Greenslade, (6) (SAM).

#### Other material examined.

**New South Wales**: 40 km NNW Louth, Lake Mere (Greenslade, P.J.M.), 40 km NNW Louth, Lake Mere (Greenslade, P.J.M.), CSIRO Lake Mere Field Station, near Louth (Bryannah, M.), Fowlers Gap (Greenslade, P.J.M.), Fowlers Gap (Greenslade, P.J.M.), Fowlers Gap (Greenslade, P.J.M.). **Northern Territory**: 15 km SE Alice Springs (Davidson, D. & Morton, S.), 20 km S Alice Springs (Greenslade, P.J.M.), Kunoth Paddock, near Alice Springs (Greenslade, P.J.M.), Kunoth Paddock, near Alice Springs (Greenslade, P.J.M. [ANIC32-900120]), Kunoth Paddock, near Alice Springs (Greenslade, P.J.M.), NW Alice Springs (Greenslade, P.J.M.). **Queensland**: ‘Merigol’ (Beutel, T.), Eulo (Greenslade, P.J.M.), Kabali East, Cooloola (Greenslade, P.J.M.), Kabali East, Cooloola (Greenslade, P.J.M.), Kabali Fan, Cooloola (Greenslade, P.J.M.), Sandringham (Greenslade, P.J.M.), Sandringham (Greenslade, P.J.M.), Sandringham (Greenslade, P.J.M.), Sandringham Station, 55 km NW Bedourie (Morton, S.R.). **South Australia**: 15 km ENE Beltana, Flinders Ranges (Greenslade, P.J.M.), 30 km E Poeppel Corner, Simpson Desert (Greenslade, P.J.M.), 5 km NW Mt Davies turnoff, Victoria Desert (Greenslade, P.J.M.), 70 km E Emu, Victoria Desert (Greenslade, P.J.M.), 7 km NW Morgan (Greenslade, P.J.M.), 85 km W Mabel Creek (Greenslade, P.J.M.), Birdsville Track, 12 km SW Etadunna (Forrest, J.), Cambrai (Greenslade, P.J.M.), Cowarie, Birdsville Track (Greenslade, P.J.M.), Oraparinna, Flinders Ranges (Greenslade, P.J.M.), Oraparinna, Flinders Ranges (Greenslade, P.J.M.), Oraparinna, Flinders Ranges (Greenslade, P.J.M.), Oraparinna, Flinders Ranges (Greenslade, P.J.M.), Wilgena Hill, E. Tarcoola (Greenslade, P.J.M.). **Western Australia**: 0.5 km along Tanami Rd (Heterick, B.E. [M198/M199/M200/M201]), 30 km N Billabong RH (Heterick, B.E. [M11]), 40 km W Halls Creek (Heterick, B.E. [M231]), 5 km N Kumarina (Heterick, B.E. [M284/M285]), 58 km E Nanutarra (Ward, P.S.), 8 km N Auski RH (Heterick, B.E. [M274]), Dowerin (Heterick, B.E. [M67/M68]), Eyre Hwy, 20 km N Norseman (Heterick, B.E. [M335]), G[ascoyne]. J[unction]. Rd, 108 km E Carnarvon (Heterick, B.E. [M305]).

#### Diagnosis.

*Melophorus microtriches* can be placed in the *M. biroi* species-group on the basis of characters of the clypeus, propodeum, mandible and palps. The species is also placed in the *M. fieldi* species-complex because of the appearance of the anteriorly placed clypeal psammophore, the compact propodeum, the presence of more than one preapical spine on the metatibia, at least in the major worker, the long, even spindly legs, and the unmodified mandible in the major worker. *Melophorus microtriches* is a very matt species and has a distinctive, reticulate tibia and antennal scape. In addition, the erect setae on the metatibia are short and stout (length of longest setae < greatest width of tibia) and cannot be confused with ‘*pillipes*” condition setae which are long, straight and fine. In addition to the erect setae, this species possesses appressed metatibial setae that are thickly distributed and often form a distinct pubescence on the tibia. The gaster of the minor worker is also strongly pubescent. These characters combined make this species readily diagnosable.

#### Minor worker description.

**Head.** Head square; posterior margin of head planar or weakly convex; frons matt or with weak sheen, shagreenate; pilosity of frons a mixture of a few well-spaced, erect setae interspersed with appressed setae only. Eye moderate (eye length 0.20–0.49 length of side of head capsule); in full-face view, eyes set at about midpoint of head capsule; in profile eyes set anteriad of midline of head capsule; in profile, eyes elliptical or slightly reniform. In full-face view, frontal carinae straight or weakly convex; frontal lobes straight in front of antennal insertion. Anteromedial clypeal margin broadly convex with anteromedial dimple; clypeal psammophore set at or above midpoint of clypeus; palp formula 6,4. Five mandibular teeth in minor worker; mandibles triangular, weakly incurved; third mandibular tooth distinctly shorter than apical tooth and teeth numbers two and four; masticatory margin of mandibles approximately vertical or weakly oblique. **Mesosoma.** Integument of pronotum, mesonotum and mesopleuron matt with indistinct shagreenate sculpture throughout; anterior mesosoma in profile broadly convex; appearance of erect pronotal setae short, (i.e., longest erect setae shorter than length of eye) and unmodified; in profile, metanotal groove shallow, broadly V or U-shaped; propodeum matt or with a weak sheen and microreticulate; propodeum angulate, propodeal angle blunt; length ratio of propodeal dorsum to its declivity about 1:1; erect propodeal setae present and abundant (greater than 12); appressed propodeal setulae long and closely aligned, creating pubescence; propodeal spiracle situated at least twice its width from the declivitous face of propodeum, and shorter (length < 0.50 × height of propodeum), or situated on or beside declivitous face of propodeum, and shorter (length < 0.50 × height of propodeum). **Petiole.** In profile, petiolar node squamiform; in full-face view, shape of petiolar node tapered with blunt vertex; node shining and distinctly shagreenate-microreticulate. **Gaster.** Gaster weakly shining with indistinct shagreenation; pilosity of first gastral tergite consisting of thick, appressed setae that form pubescence, interspersed with numerous short, bristly, erect setae. **General characters.** Colour variegated orange tan and brown.

#### Major worker description.

**Head.** Head square, or rectangular; posterior margin of head planar or weakly convex; cuticle of frons matt or with weak sheen, indistinctly shagreenate; pilosity of frons a mixture of a few well-spaced, erect setae interspersed with appressed setae only. Eye moderate (eye length 0.20–0.49 length of head capsule); in full-face view, eyes set above midpoint of head capsule; in profile, eye set anteriad of midline of head capsule; eyes elliptical. In full-face view, frontal carinae straight or weakly convex; frontal lobes curved inward in front of antennal insertion. Anterior clypeal margin broadly convex with anteromedial dimple; clypeal psammophore set at or above midpoint of clypeus; palp formula 6,4. Five mandibular teeth in major worker; mandibles triangular, weakly incurved; third mandibular tooth distinctly shorter than apical tooth and teeth numbers two and 4; masticatory margin of mandibles approximately aligned vertically or weakly oblique. **Mesosoma.** Integument of pronotum, mesonotum and mesopleuron moderately shining and shagreenate throughout; anterior mesosoma in profile broadly convex; erect pronotal setae short, (i.e., shorter than length of eye) and unmodified; in profile, metanotal groove shallow, broadly V- or U-shaped; propodeum matt or with weak sheen and microreticulate-striolate; propodeum angulate, propodeal angle blunt; length ratio of propodeal dorsum to its declivity between 1:1 and1:2; erect propodeal setae present and abundant (at least a dozen); appressed propodeal setae long and separated by at least own length; propodeal spiracle situated on or beside declivitous face of propodeum, and shorter (length less than 0.50 × height of propodeum). **Petiole.** In profile, petiolar node squamiform; in full-face view, shape of petiolar node uniformly rounded, or tapered with blunt vertex; node shining and faintly shagreenate-microreticulate. **Gaster.** Gaster shining with superficial microreticulation; pilosity of first gastral tergite consisting of thick, appressed setae that form pubescence, interspersed with numerous short, bristly, erect setae. **General characters.** Colour variegated orange- or reddish-brown with brown gaster.

#### Measurements.

Worker (n = 8): CI 105–123; EI 23–31; EL 0.23–0.35; HL 0.69–1.28; HW 0.73–1.57; ML 1.06–1.72; MTL 0.65–1.00; PpH 0.12–0.17; PpL 0.43–0.72; SI 71–118; SL 0.86–1.11.

#### Comments.

*Melophorus microtriches* is one of the commoner members of the *M. fieldi* complex, and is found throughout mainland Australia. This ant is one of the few *Melophorus* that can be found in urbanized habitats, e.g., relictual woodland and rehabilitated road verges in the Perth metropolitan area. The species is readily recognizable by its vestiture of short, prickly setae on the body, limbs and antennal scapes, its finely sculptured antennal scape and legs and the long, overlapping, silvery appressed setae on the gaster. Typically, small minor workers are bicoloured with a dark pronotum and light-coloured mesonotum. The sequencing data are unequivocal: on a five-gene tree, *M. microtriches* is closely related to *M. hirsutipes*, which it strongly resembles.

Despite its ubiquity, there are not a lot of ecological data for the species. Substrates include gravel, red soil and grey sand. Known natural habitats include mulga, riparian woodland and ‘tall woodland’, but it is also found in disturbed habitats such as paddocks, the bushier street verges and occasionally parkland native gardens. There is no information on its habits, apart from the fact it is a ground forager.

#### Etymology.

Greek *mikros* (‘small’) plus pl. of *trichos* (‘hair’); noun in the nominative plural standing in apposition to the generic name.

**Figure 65.**
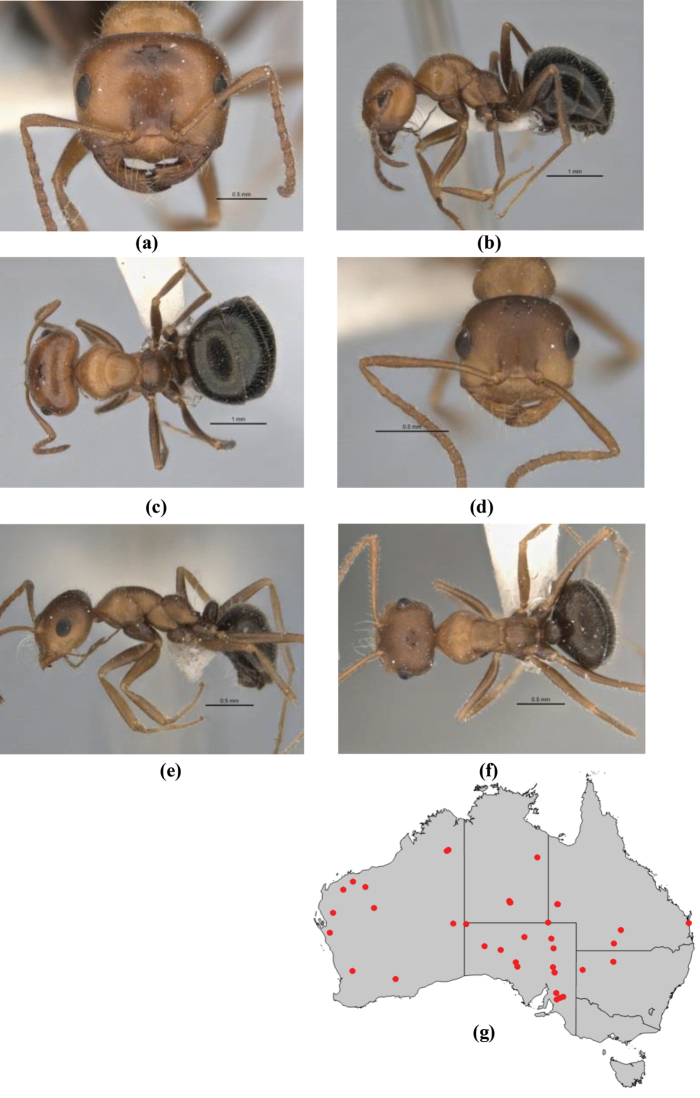
*Melophorus microtriches* sp. n.: major worker paratype (ANIC32-900121–middle ant) frons (**a**), profile (**b**) and dorsum (**c**); minor worker holotype (ANIC32-900121–bottom ant) frons (**d**), profile (**e**) and dorsum (**f**); distribution map for the species (**g**). Low resolution scale bars: 1 mm (**b, c**); 0.5 mm (**a, d–f**).

### Melophorus orthonotus

Taxon classificationAnimaliaHymenopteraFormicidae

Heterick, Castalanelli & Shattuck sp. n.

http://zoobank.org/C3D07714-436B-40A2-83D7-68E2087209E3

#### Types.

Holotype media worker from 85 km E Meentheena OC 21°17'44"S, 121°15'50"E, Western Australia, 29 July 2003-11 October 2004, CALM Pilbara Survey, Site NE13, Ethylene glycol pitfalls [JDM32-004590] (WAM). Paratype: Major(?) worker from 23 km NE of Warrawagine Hstd 20°41'54"S, 120°51'23"E, Western Australia, 22 May 2006-21 August 2006, CALM Pilbara Survey, Site: PHYEO1: Ethylene glycol pitfalls [JDM32-004589] (ANIC).

#### Diagnosis.

*Melophorus orthonotus* can be placed in the *M. biroi* species-group on the basis of characters of the clypeus, mandible and palps. The elongate propodeum, however, is unique in the species-group and resembles more closely the typical form of the propodeum in the *M. aeneovirens* species-group (see below). The species is also placed in the *M. fieldi* species-complex because of the appearance of the anteriorly placed clypeal psammophore, the compact propodeum, the presence of more than one preapical spine on the metatibia, at least in the major worker, the long, even spindly legs, and the unmodified mandible in the major worker. Uniquely among the *Melophorus* seen, where rare species are often only represented by minor workers, here it is the major and media worker of this species that are the sole representatives in the collections examined. Even so, the species cannot be confused with any other *Melophorus*. In profile, the major and media worker have a smooth, elongate propodeum, with the propodeal angle indicated only by a faint curve, the metanotal groove being a weak impression so that the mesonotal and propodeal outline is barely interrupted. The mesonotum and mesopleuron are not separated by any impression or suture, and the mesosternal outline is strongly convergent anteriad with the outline of mesonotum, similar to some ants in the *M. aeneovirens* species-group.

#### Media worker description.

**Head.** Head square; posterior margin of head weakly convex; frons shining and smooth except for piliferous pits; pilosity of frons a mixture of a few well-spaced, erect setae interspersed with appressed setae only. Eye large (eye length ≥ 0.50 × length of side of head capsule); in profile, eye set at about midpoint of head capsule; in profile, eye set anteriad of head capsule; eyes elliptical or slightly reniform. In full-face view, frontal carinae distinctly concave; frontal lobes straight in front of antennal insertion. Anteromedial clypeal margin broadly and evenly convex; clypeal psammophore set at or above midpoint of clypeus; palp formula 6,4. Five mandibular teeth in minor worker; mandibles triangular, weakly incurved; third mandibular tooth distinctly shorter than apical tooth and teeth numbers two and four; masticatory margin of mandibles approximately vertical or weakly oblique. **Mesosoma.** Integument of pronotum, mesonotum and mesopleuron shining and mainly smooth, vestigial shagreenation most noticeable on humeri and mesopleuron; anterior mesosoma in profile weakly elevated anteriad, thereafter gently sinuate, pronotum and mesonotum on same plane; appearance of erect pronotal setae short, (i.e., longest erect setae shorter than length of eye) and unmodified, or erect pronotal setae absent; in profile, metanotal groove shallow, broadly V or U-shaped; propodeum shining and smooth or with superficial and almost invisible microsculpture; propodeum always smoothly rounded; propodeal dorsum and declivity confluent; erect propodeal setae variable in number, may be absent; appressed propodeal setulae short, separated by more than own length and inconspicuous; propodeal spiracle situated on or beside declivitous face of propodeum, and shorter (length < 0.50 × height of propodeum). **Petiole.** In profile, petiolar node subcuboidal, vertex bluntly rounded; in full-face view, shape of petiolar node uniformly rounded; node shining and smooth throughout. **Gaster.** Gaster smooth and glossy; pilosity of first gastral tergite consisting of well-spaced, erect and semi-erect setae interspersed with regularly placed appressed setae. **General characters.** Colour of foreparts orange tan to reddish brown, gaster brown to blackish-brown.

#### Major worker description.

**Head.** Head square; posterior margin of head planar or weakly convex; cuticle of frons matt or with weak sheen, microreticulate; frons consisting exclusively or almost exclusively of well-spaced, appressed setae only (small, erect setae, if present, usually confined to ocular triangle or posterior margin of head). Eye moderate (eye length 0.20–0.49 length of head capsule); in full-face view, eyes set above midpoint of head capsule; in profile, eye set anteriad of midline of head capsule; eyes elliptical. In full-face view, frontal carinae straight, divergent posteriad; frontal lobes straight in front of antennal insertion. Anterior clypeal margin straight; clypeal psammophore set at or above midpoint of clypeus; palp formula 6,4. Five mandibular teeth in major worker; mandibles triangular, weakly incurved; third mandibular tooth distinctly shorter than apical tooth and teeth numbers two and four; masticatory margin of mandibles approximately aligned vertically or weakly oblique. **Mesosoma.** Integument of pronotum, mesonotum and mesopleuron moderately shining and shagreenate throughout; anterior mesosoma in profile broadly convex; erect pronotal setae short, (i.e., shorter than length of eye) and unmodified; in profile, metanotal groove shallow, indicated mainly by an angle and metathoracic spiracles; propodeum shining, with multiple hair like striolae; propodeum smoothly rounded or with indistinct angle; propodeal dorsum and declivity confluent; erect propodeal setae absent; appressed propodeal setae short, separated by more than own length and inconspicuous; propodeal spiracle situated on or beside declivitous face of propodeum, and longer (length ≥ 0.50 × height of propodeum). **Petiole.** In profile, petiolar node squamiform; in full-face view, shape of petiolar node generally rounded with median indentation; node shining and faintly shagreenate-microreticulate. **Gaster.** Gaster shining, shagreenate (‘LP record’ appearance); pilosity of first gastral tergite consisting of well-spaced short, inconspicuous, appressed setae, erect setae (present in at least some workers) confined to margin of the sclerite. **General characters.** Colour of head orange tan, mesosoma and legs orange, gaster blackish-brown.

#### Measurements.

Worker (n = 1): CI 104; EI 22; EL 0.25; HL 1.11; HW 1.16; ML 1.80; MTL 0.97; PpH 0.13; PpL 0.85; SI 111; SL 1.28.

#### Comments.

This species is described from two specimens taken in the Pilbara region, WA. As with *M. teretinotus*, in the *M. aeneovirens* species-group, this member of the *M. fieldi* group has a very weak to vestigial metanotal groove, which distinguishes it from other species in its complex. No samples were available for sequencing. The known specimens were collected in ethylene glycol pitfall traps, but no other information is available.

#### Etymology.

Compound of Greek: *othos* (‘straight’) plus *notus* (Neo-Latin ‘back’); adjective in the nominative case.

**Figure 66.**
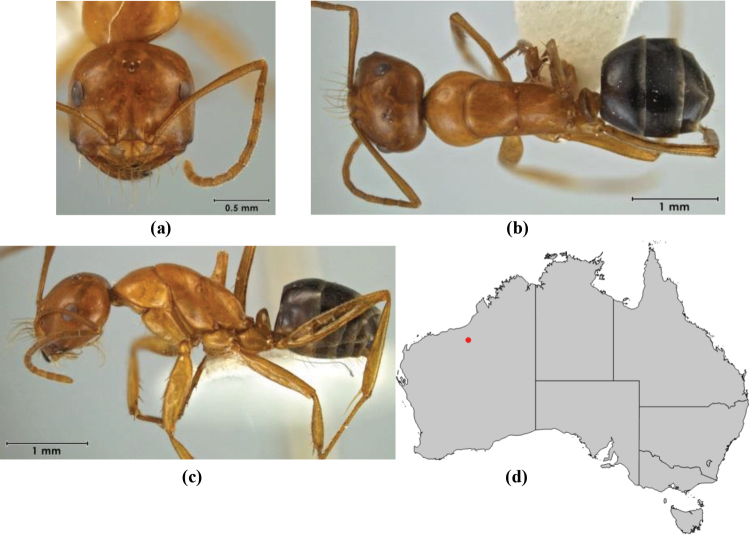
*Melophorus orthonotus* sp. n.: holotype media worker (JDM32-004590) frons (**a**), profile (**b**) and dorsum (**c**); distribution map for the species (**d**).

### Melophorus paramorphomenus

Taxon classificationAnimaliaHymenopteraFormicidae

Heterick, Castalanelli & Shattuck sp. n.

http://zoobank.org/3361590A-B0E0-4A69-82E2-E39655DA7FE0

#### Types.

Holotype minor worker (bottom ant) from Tropicana Minesite 29°15'40"S, 124°35'50"E, Western Australia, January 2009, J. Summerhayes, pitfall trap: *Casuarina* C1:4 [JDM32-004561] (WAM). Paratypes: 2 major workers on same pin with same details as holotype (WAM); 2 minor workers and queen from 12 km N of Billabong RH 26°44'S, 114°35'E, 11 December 2001, Heterick, B.E., Light scrub, red soil, am [JDM32-001988] (ANIC); minor worker from Barrow Island 20°49'43"S, 115°26'36"E, Western Australia, 17 May 2005, S. Callan, All 5 day pitfalls, R2 105 Pit 09 [JDM32-001989] (WAM).

#### Other material examined.

**Western Australia**: Barrow Island (Gunawardene, N. / Taylor, C. [M313]),

#### Diagnosis.

*Melophorus paramorphomenus* can be placed in the *M. biroi* species-group on the basis of characters of the clypeus, propodeum, mandible and palps. The species is also placed in the *M. fieldi* species-complex because of the appearance of the anteriorly placed clypeal psammophore, the compact propodeum, the presence of more than one preapical spine on the metatibia, at least in the major worker, the long, even spindly legs and the unmodified mandible in the major worker. *Melophorus paramorphomenus* cannot be confused with any other *Melophorus* except perhaps *M. cuneatus*, which, however, has the typical features of the *M. biroi* species-complex. The worker thorax possesses an apparent metanotum that is confluent with the mesonotum, and often extends over the propodeum. The metanotal suture is obsolete, its position indicated only by a superficial, transverse furrow (more pronounced in the major worker), the propodeum is reduced in size and wedge-shaped, with the narrow end of the wedge often under a fold of the metanotum and, finally, the metathoracic spiracle is lateral and situated within the metanotal sector

#### Minor worker description.

**Head.** Head square; posterior margin of head weakly convex; frons shining with superficial shagreenation or microreticulation only; frons consisting exclusively or almost exclusively of well-spaced, appressed setae only (small, erect setae, if present, usually confined to ocular triangle or posterior margin of head). Eye moderate (eye length 0.20–0.49 length of side of head capsule); in full-face view, eyes set at about midpoint of head capsule; in profile, set anteriad of head capsule; eyes elliptical or slightly reniform. In full-face view, frontal carinae straight or weakly convex; frontal lobes straight in front of antennal insertion. Anteromedial clypeal margin straight; clypeal psammophore set at or above midpoint of clypeus; palp formula 6,4. Five mandibular teeth in minor worker; mandibles triangular, weakly incurved; third mandibular tooth distinctly shorter than apical tooth and teeth numbers two and four; masticatory margin of mandibles approximately vertical or weakly oblique. **Mesosoma.** Integument of pronotum, mesonotum and mesopleuron shining and microreticulate, microreticulation reduced on humeri; anterior mesosoma in profile rounded anteriad, thereafter pronotum and whole of mesonotum flattened and on a higher plane than propodeum; appearance of erect pronotal setae short, (i.e., longest erect setae shorter than length of eye) and unmodified, or erect pronotal setae absent; in profile, metanotal groove absent; propodeum shining and finely striolate and microreticulate; propodeum wedge-shaped, tapering dorsad; length ratio of propodeal dorsum to its declivity not applicable, propodeal dorsum reduced to a narrow sliver; erect propodeal setae always absent; appressed propodeal setulae sparse or absent, if present then not regularly spaced; propodeal spiracle situated on or beside declivitous face of propodeum, and shorter (length < 0.50 × height of propodeum). **Petiole.** In profile, petiolar node squamiform; in full-face view, shape of petiolar node uniformly rounded; node shining and distinctly shagreenate-microreticulate. **Gaster.** Gaster weakly shining with indistinct shagreenation; pilosity of first gastral tergite consisting of well-spaced short, inconspicuous, appressed setae, erect setae (present in at least some workers) confined to margin of sclerite. **General characters.** Colour of foreparts dark ochre or orange tan, gaster brown.

#### Major worker description.

**Head.** Head square, or rectangular; posterior margin of head weakly convex; cuticle of frons shining and smooth except for piliferous pits; pilosity of frons a mixture of a few well-spaced, erect setae interspersed with appressed setae only. Eye moderate (eye length 0.20–0.49 length of head capsule); in full-face view, eyes set above midpoint of head capsule; in profile, eye set anteriad of midline of head capsule; eyes elliptical. In full-face view, frontal carinae straight or weakly convex; frontal lobes straight in front of antennal insertion. Anterior clypeal margin straight; clypeal psammophore set at or above midpoint of clypeus; palp formula 6,4. Four mandibular teeth in major worker-5; mandibles narrow, strap-like, internal and external borders parallel or nearly so; third mandibular tooth distinctly shorter than apical tooth and teeth numbers two and four; masticatory margin of mandibles approximately aligned vertically or weakly oblique. **Mesosoma.** Integument of pronotum, mesonotum and mesopleuron shining with indistinct microsculpture that is most pronounced on lower surfaces; anterior mesosoma in profile steeply rounded anteriad, thereafter pronotum and whole of mesonotum flattened and on a higher plane than propodeum; erect pronotal setae short, (i.e., shorter than length of eye) and unmodified; in profile, metanotal groove a weak furrow; propodeum shining, with multiple hair like striolae; propodeum wedge-shaped, tapering dorsad; length ratio of propodeal dorsum to its declivity not applicable, propodeal dorsum reduced to a narrow sliver; erect propodeal setae absent; appressed propodeal setae sparse or absent, if present then not regularly spaced; propodeal spiracle situated on or beside declivitous face of propodeum, and shorter (length less than 0.50 × height of propodeum). **Petiole.** In profile, petiolar node squamiform; in full-face view, shape of petiolar node uniformly rounded; node shining and smooth with vestigial microreticulation anteriad. **Gaster.** Gaster shining with superficial microreticulation; pilosity of first gastral tergite consisting of thick, often distally flattened, erect setae over well-spaced, short, appressed setae. **General characters.** Colour orange tan with brown gaster.

#### Measurements.

Worker (n = 4): CI 103–120; EI 20–29; EL 0.17–0.27; HL 0.58–1.12; HW0.60–1.35; ML 0.84–1.45; MTL 0.51–0.87; PpH 0.05–0.04; PpL 0.27–0.47; SI 75–121; SL 0.73–1.01.

#### Comments.

The peculiarly developed metanotum, under which the propodeum is squeezed, distinguishes this ant from all others. *Melophorus cuneatus*, in the *M. ludius* complex, bears a superficial resemblance, but here the propodeum projects to the dorsum of the trunk as a wedge: that ant also possesses the diagnostic characters of its species complex (posteriorly placed clypeal psammophore, etc.). Andersen (2007), who erroneously places this species (and *M. cuneatus*?) in the *M. mjobergi* complex (‘group’), comments on its resemblance to *Notoncus enormis*. The ant is known only from WA where it is found from about Kalbarri to Barrow Island. One inland sample has been collected at Tropicana Minesite, east of Laverton. Attempts to sequence pitfall trap material from Barrow Island have been unsuccessful, probably because of collection into diluted alcohol. The principal author of this revision has collected the species from a nest in red soil 12 km N of Billabong Station in the Midwest, but regrettably (as is the case with many other *Melophorus*) other data are lacking.

#### Etymology.

Greek *paramorphos* (‘change of physical shape’) plus Latinized Greek *menus* (Greek *menos* ‘spirit’ or ‘force’) referring to the odd appearance of the mesosoma; adjective in the nominative case.

**Figure 67.**
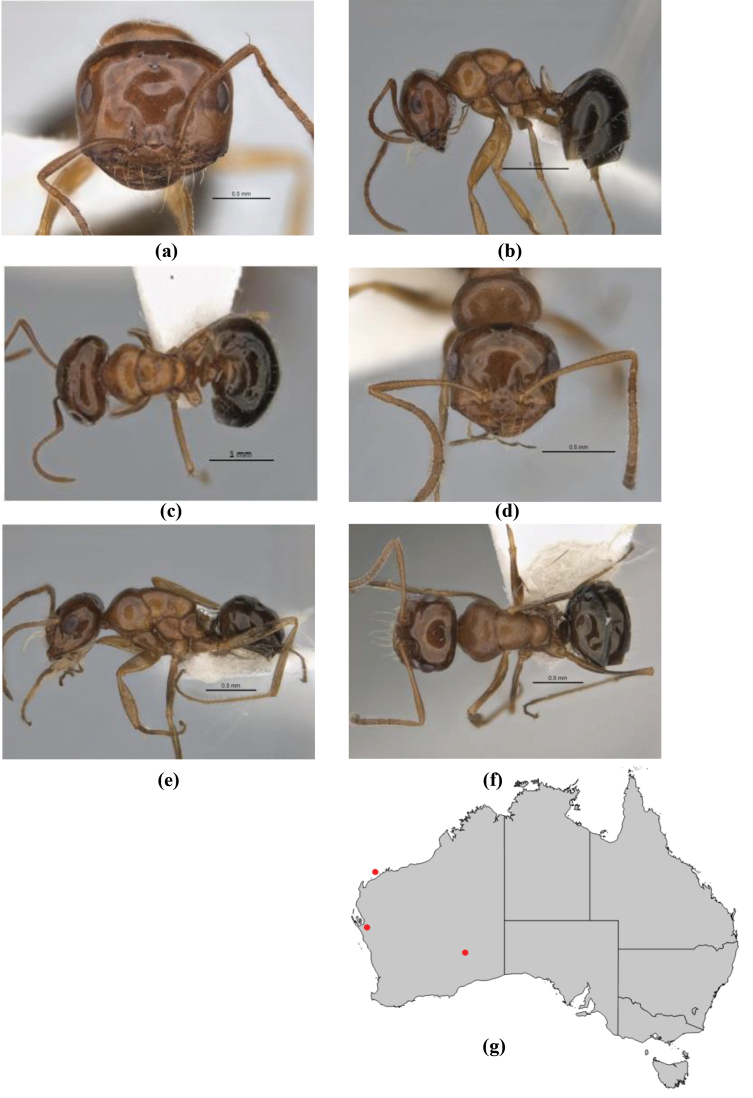
*Melophorus paramorphomenus* sp. n.: major worker paratype (JDM32-004561–top ant) frons (**a**), profile (**b**) and dorsum (**c**); minor worker holotype frons (**d**), profile (**e**) and dorsum (**f**); distribution map for the species (**g**). Low resolution scale bars: 1 mm (**b**); 0.5 mm (**a, d–f**).

### Melophorus perthensis

Taxon classificationAnimaliaHymenopteraFormicidae

Forel, stat n.

Melophorus turneri subsp. perthensis Wheeler 1934: 152.

#### Types.

Syntype major and minor workers: Rottnest Island and Kings Park, Western Australia [MCZ] (examined: images taken by S.O.Shattuck of MCZ specimens “cotype’ 23042, MCZ-ENT 00303602 [Kings Park] and “cotype’ 23042 [Rottnest Is.]. (Note. Series MCZ-ENT 00303602 includes a major worker of *M. chauliodon*.)

#### Other material examined.

**New South Wales**: Callubri Station (Greaves, T.), Mudgee (Lowery, B.B.), near Pooraka, 8mk E Florida, 10 km E Canbelego (Ward, P.S. [ANIC32-010587]), Pulletop Nature Reserve, near Griffith (Lowery, B.B.), Round Hill Reserve, 40 km NW Eubalong (Morton, S.R.). **Northern Territory**: 15 mi NW Mt Wedge Homestead (McInnes & Dowse), Johnstons Lagoon (Greaves, T.), Kunoth Paddock, near Alice Springs (Greenslade, P.J.M.), Kunoth Paddock, near Alice Springs (Greenslade, P.J.M.). **Queensland**: Mica Creek, Mount Isa (Burwell, C. J.), Illaweena St. Drewvale (QM Party), Tindaree, Hannaford Rd South via Tara (House, A./Brown, S.). **South Australia**: 10 km E Mt Ive Homestead, Gawler Ranges (Greenslade, P.J.M.), 10 km E Mt Ive Homestead, Gawler Ranges (Greenslade, P.J.M.), 10 km E Mt Ive Homestead, Gawler Ranges (Greenslade, P.J.M.), 10 km E Mt Ive Homestead, Gawler Ranges (Greenslade, P.J.M.), 16 mi SE Mt Wooltarlinna (McInnes & Dowse), 29 mi E Mt Davies, Tomkinson Range (McInnes, R. & Dowse, J.), 6 km W Wanilla, Eyre Peninsula (Greenslade, P.J.M.), Brookfield (Greenslade, P.J.M.), Calca (Lowery, B.B.), Gawler Ranges (Greenslade, P.J.M.), Streaky Bay (Lowery, B.B.). **Western Australia**: 109.5 km SE of Newman (van Leeuwen, S. & Bromilow, R.N. [JDM32-001836]), 15 mi ESE Leonora (McInnes & Dowse), 2 km N Capricorn RH (Heterick, B.E. [M175/M176/M177]), 23 mi S Youanmi (Douglas, A.M. & M.J.), 27 mi SEbyS Zanthus (Taylor, R.W.), 32 mi WNW Mt Samuel (McInnes, R. & Dowse, J.), 34 mi WSW Ravensthorpe (Greaves, T.), 40 mi W Esperance (Lowery, B.B.), 44 mi NE Carnegie Homestead (McInnes, R. & Dowse, J.), 48 mi N Balladonia Homestead (Taylor, R.W.), 4 mi S Pingrup (Greaves, T.), 5.5 km E of Merredin (Jacobs, M. [JDM32-001837]), 6 mi W Yellowdine (Greaves, T.), Alcoa via Jarrahdale (collector unknown [JDM32-001858]), Bartons Mill (Majer, J.D. [JDM32-001850]), Beechboro, Perth (Lowery, B.B.), Blue Hill Range (Bokhari, F. [M147]), Blue Hill Range (Bokhari, F. [M149]), Chandlers Breakaway (Heterick, B.E. [JDM32-001832]), Christmas Tree Well (Heterick, B.E. [M21/M57]), Coomallo Downs (Heterick, B.E. [M10/M40/M43/M44]), Darlington, E Perth (Lowery, B.B.), Del Park (Wallace, J. [JDM32-001846]), Dwellingup (Majer, J.D. [JDM32-001839]), Dwellingup (Majer, J.D. [JDM32-001838]), Dwellingup (Wallace, J. [JDM32-001842]), Harrismith (Heterick, B.E. [JDM32-001834]), Int. Holland Tr./Norseman Rd. (Heterick, B.E. [JDM32-001833]), Kings Park, Perth (collector unknown [ANIC32-006437]), Kings Park, Perth (Lowery, B.B.), KW Road, Lancelin district (Heterick, B.E. [M79/M120]), KW Road, Lancelin district (Heterick, B.E. [M76/M117]), Marillana Ck. (Dunlop, N. [JDM32-001861]), Mundaring Weir (collector unknown), Palmyra (Heterick, B.E. [JDM32-001831]), Perth (Majer, J.D. [JDM32-001848]), Rabbit Proof Fence Rd. (Heterick, B.E. [JDM32-004572]), Reabold H. (Majer, J.D. [JDM32-001845]), Reabold H. (Majer, J.D. [JDM32-001844]), Sappers Rd (Heterick, B.E. [M160]), Serpentine Falls, Serpentine National Park (Shattuck, S.O.), Tardun (Mercovich, C.), Yamarna Homestead (Richardson, S. [ANIC32-032237]), Yarra Road (Douglas, A.M. & M.J.).

#### Diagnosis.

*Melophorus perthensis* can be placed in the *M. biroi* species-group on the basis of characters of the clypeus, propodeum, mandible and palps. The species is also placed in the *M. fieldi* species-complex because of the appearance of the anteriorly placed clypeal psammophore, the compact propodeum, the presence of more than one preapical spine on the metatibia, at least in the major worker, the long, even spindly legs, and the unmodified mandible in the major worker. *Melophorus perthensis* is a relatively large (HW 0.72–1.92 mm), smooth and shiny species. The appressed setae on the gaster are very small and well-separated. This ant is most likely to be confused with some populations of *M. turneri*. However, when viewed in profile, the smoothly rounded pronotum and the mesonotum that is elongate and broadly convex and arches above the level of the pronotum in all workers make this ant distinctive in most cases. In a few eastern states populations of *M. turneri* the pronotum and mesonotum are strongly convex, but these workers do not possess the very truncate propodeum seen in *M. perthensis* that usually arises above the metanotal groove.

#### Minor worker description.

**Head.** Head square; posterior margin of head planar or weakly convex; frons shining with superficial shagreenation or microreticulation only; pilosity of frons a mixture of a few well-spaced, erect setae interspersed with appressed setae only, or consisting exclusively or almost exclusively of well-spaced, appressed setae only (small, erect setae, if present, usually confined to ocular triangle or posterior margin of head). Eye moderate (eye length 0.20–0.49 length of side of head capsule); in full-face view, eyes set above midpoint of head capsule; in profile, eye set anteriad of midline of head capsule; eyes elliptical or slightly reniform. In full-face view, frontal carinae distinctly concave; frontal lobes straight in front of antennal insertion. Anteromedial clypeal margin broadly convex with anteromedial dimple; clypeal psammophore set at or above midpoint of clypeus; palp formula 6,4. Five mandibular teeth in minor worker; mandibles triangular, weakly incurved; third mandibular tooth distinctly shorter than apical tooth and teeth numbers two and four; masticatory margin of mandibles approximately vertical or weakly oblique. **Mesosoma.** Integument of pronotum, mesonotum and mesopleuron with weak to moderate sheen and superficial microreticulation (more pronounced on mesopleuron); anterior mesosoma in profile broadly convex; appearance of erect pronotal setae short, (i.e., longest erect setae shorter than length of eye) and unmodified, or erect pronotal setae absent; in profile, metanotal groove deep, V-shaped; propodeum shining and finely striolate and microreticulate; propodeum always smoothly rounded; propodeal dorsum and declivity confluent; erect propodeal setae variable in number, may be absent; appressed propodeal setulae short, separated by more than own length and inconspicuous; propodeal spiracle situated on or beside declivitous face of propodeum, and shorter (length < 0.50 × height of propodeum). **Petiole.** In profile, petiolar node squamiform, or subcuboidal, vertex bluntly rounded; in full-face view, shape of petiolar node uniformly rounded; node shining and smooth throughout. **Gaster.** Gaster shining, shagreenate (‘LP record’ appearance); pilosity of first gastral tergite consisting of well-spaced, erect and semi-erect setae interspersed with regularly placed appressed setae. **General characters.** Colour of foreparts brown, gaster usually dark brown.

#### Major worker description.

**Head.** Head horizontally rectangular, broader than wide; posterior margin of head planar or weakly concave; cuticle of frons shining with superficial shagreenation or microreticulation only; pilosity of frons a mixture of a few well-spaced, erect setae interspersed with appressed setae only, or consisting exclusively or almost exclusively of well-spaced, appressed setae only (small, erect setae, if present, usually confined to ocular triangle or posterior margin of head). Eye moderate (eye length 0.20–0.49 length of head capsule); in full-face view, eyes set above midpoint of head capsule; in profile, eye set anteriad of midline of head capsule; eyes elliptical. In full-face view, frontal carinae concave; frontal lobes straight in front of antennal insertion. Anterior clypeal margin broadly convex with anteromedial dimple; clypeal psammophore set at or above midpoint of clypeus; palp formula 6,4. Five mandibular teeth in major worker; mandibles triangular, weakly incurved; third mandibular tooth distinctly shorter than apical tooth and teeth numbers two and four; masticatory margin of mandibles approximately aligned vertically or weakly oblique. **Mesosoma.** Integument of pronotum, mesonotum and mesopleuron shining with indistinct microsculpture that is most pronounced on lower surfaces; anterior mesosoma in profile broadly convex; erect pronotal setae long (i.e., longer than length of eye) and unmodified, or short, (i.e., shorter than length of eye) and unmodified; in profile, metanotal groove shallow, broadly V- or U-shaped; propodeum shining and finely striolate and microreticulate; propodeum smoothly rounded or with indistinct angle; propodeal dorsum and declivity confluent; erect propodeal setae present and sparse to moderate (1-12); propodeal spiracle situated on or beside declivitous face of propodeum, and shorter (length less than 0.50 × height of propodeum). **Petiole.** In profile, petiolar node squamiform; in full-face view, shape of petiolar node uniformly rounded, or generally rounded with median indentation, node shining and faintly shagreenate-microreticulate. **Gaster.** Gaster weakly shining with indistinct shagreenation; pilosity of first gastral tergite consisting of well-spaced, erect and semi-erect setae interspersed with regularly spaced appressed setae. **General characters.** Colour reddish-brown, gaster darker.

#### Measurements.

Worker (n = 8): CI 97–119; EI 17–32; EL 0.23–0.33; HL 0.74–1.61; HW 0.72–1.92; ML 1.06–2.02; MTL 0.69–1.36; PpH 0.11–0.19; PpL 0.44–0.74; SI 70–123; SL 0.89–1.35.

#### Comments.

This is a very common species and one of the few *Melophorus* for which there is detailed ecological information. *Melophorus perthensis* is characterized by the rounded mesonotum, giving this sclerite an arcuate appearance in profile. In some populations, particularly on the east coast, this trait is not so accentuated, and other characters are required to discriminate between the species and its relatives, particularly *M. turneri*. The truncated propodeum is commonly helpful for identifying minor workers, and *M. perthensis* is generally larger than *M. turneri* (ML 1.06–2.02 mm compared with 0.90–1.57 mm). This species has been recorded from all mainland Australian states except Victoria, but probably also occurs there. However, its occurrence in Tasmania is problematic. Genetically, the ant is distinct from M. *turneri*, and is raised to species in this revision. However, on individual gene trees, members of this taxon cluster close to some *M. turneri* populations.

As with *Melophorus sulla*, the type material reveals confusion, with more than one species included in the syntype series. A media or small major worker collected in Kings Park, Western Australia on 20 October 1931 by the Harvard Australian Expedition led by William Morton Wheeler and held at MCZ (‘co-type 23042’) is actually *Melophorus chauliodon* (sp. n. this paper). *Melophorus chauliodon* and *Melophorus perthensis* are by far the two most common *Melophorus* in the Perth metropolitan area, frequently occur together and bear a superficial resemblance to one another.

Majer et al. (2011) found *M. perthensis* was a key dispersal agent of seeds in a variety of woodland sites in the Darling Plateau and the Swan Coastal Plain between Perth in the north and Manjimup in the south (Two other non-*Melophorus* ants were also studied). Major findings connected with *M. perthensis* were as follows:

• this species was seasonally active between October and April, with peaks of activity in December and January. No activity was recorded between May and September,

• peak diurnal activity was in the early afternoon,

• fire caused a decline in activity in three out of four site studied, with 1.7 times as many individuals active before burns than after burns,

• this species prefers low ground cover and low understorey shade for its nest sites,

• the nests themselves comprise a vertical channel with a series of side galleries at regular intervals,

• mean number of workers per nest was 230.4,

• food items comprised seeds (29.1% of forage), miscellaneous plant fragments (25.3%) and invertebrates (54.6%),

• regression models relating ant foraging activity to myrmechore flowering revealed a four month lag time, and

• seedling emergence was much higher around nests of this species (and other ant nests) than elsewhere.

The researchers also found nests of this species in the sandplain were deeper than in lateritic soils, and colony sizes were much lower than for *Melophorus bagoti*. Label data reveals these ants can be found in mallee scrub and in red soil in other parts of Australia. This species is the commonest *Melophorus* found in the Perth metropolitan area, along with *M. chauliodon*. In the summer season, active nests can be seen in sandy soils in yards, gardens, beside footpaths, and, indeed, in any area where there is sufficient soil for ants to nest. Probably because of its thermophilic behavior, this is one native ant species that is not fazed by the presence of aggressive invasive exotics such as the Big-headed ant *Pheidole megacephala* (Fabricius) and the Argentine ant *Linepithema humile* (Jerdon).

**Figure 68.**
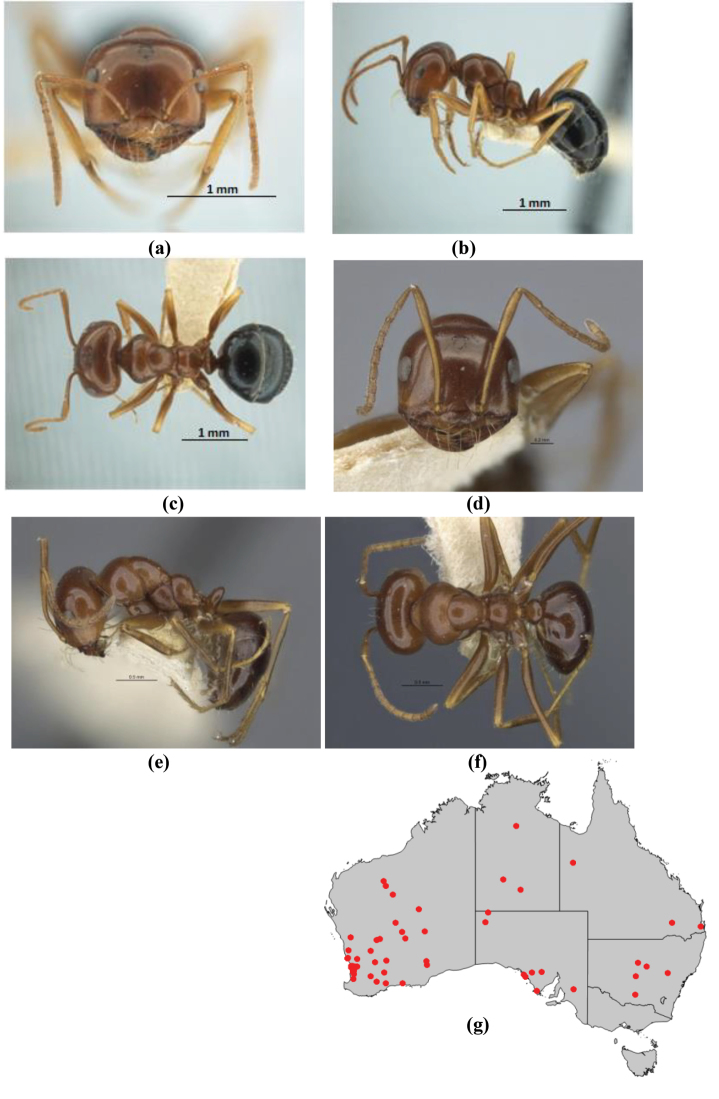
*Melophorus perthensis* Wheeler: MCZ major worker syntype (MCZC002302) frons (**a**), profile (**b**) and dorsum (**c**); MCZ minor worker syntype (MCZ-ENT00303602) frons (**d**), profile (**e**) and dorsum (**f**); distribution map for the species (**g**). Low resolution scale bars: 0.5 mm (**e, f**); 0.5 mm (**d**).

### Melophorus sericothrix

Taxon classificationAnimaliaHymenopteraFormicidae

Heterick, Castalanelli & Shattuck sp. n.

http://zoobank.org/F159A353-DAC6-4527-9DD2-43DD617C88D4

#### Types.

Holotype minor worker (bottom ant) from Brookfield Conservation Park 34°19'S, 139°29'E, South Australia, 29 October 1991, S. Shattuck #2506.3 [ANIC32-900174] (ANIC). Paratypes: 2 minor workers on same pin and with same details as holotype (ANIC); 3 major workers from Brookfield Conservation Park 34°19'S, 139°29'E, South Australia, 29 October 1991, S. Shattuck #2507.3 [ANIC32-900103] (ANIC); 2 minor workers from Worsley 32°23'S, 115°56'E, Western Australia, February 2014, G. Orabi, WP92, litter [JDM32-001557] (BMNH); 3 minor workers, a media worker and 3 major workers from 9 miles E of Newdegate, Western Australia, 4 October 1947, T. Greaves, ‘*Melophorus*’ (MCZ); 3 major workers, ‘Grs(?), Brookfield, 198h 5(?)’ [South Australia], 128 (SAM); minor and media worker from Eneabba-Leeman Road 29°52.26'S, 115°05.45'E, Western Australia, April 2004, R. Dunn, pitfall trap (L57), 1.5m shrubland, white sand on limestone, ‘ID: *Melophorus mjobergi* Forel det. by: B.E. Heterick [*sic*], Date: 14 Nov. 2004’, ‘Checked against syntype specimen’ [Note: this identification is now determined to be incorrect, despite the very similar appearance of the minor worker of these two species-BEH] [JDM32-001559] (WAM); 2 minor workers from 2 km S of Worsley 32°24'S, 115°56'E, Western Australia, 11 February 2004, G. Orabi, WP (West Pit) 92, hand-collected, litter [JDM32-001558] (WAM).

#### Other material examined.

**South Australia**: 14 km SW Taplin (Vertebrate Survey [M84/M107]), Oraparinna, Flinders Ranges (Greenslade, P.J.M.), Poochera (Taylor, R.W. & Bartell, R.J.). **Western Australia**: Boddington (Postle, A.C. [JDM32-001568]), Eneabba (Bisevac, L. & Heterick, B.E. [JDM32-001560]), Eneabba (Sartori, M. & Stone, R. [JDM32-001565]), Harrismith (Jacobs, M. [JDM32-001556]), Tammin (Greaves, T.).

#### Diagnosis.

*Melophorus sericothrix* can be placed in the *M. biroi* species-group on the basis of characters of the clypeus, propodeum, mandible and palps. The species is also placed in the *M. fieldi* species-complex because of the appearance of the anteriorly placed clypeal psammophore, the compact propodeum, the presence of more than one preapical spine on the metatibia, at least in the major worker, the long, even spindly legs, and the unmodified mandible in the major worker. *Melophorus sericothrix* possesses several characters which, taken, together, combine to define the species adequately: in profile, the head of the minor worker is weakly to moderately dorsoventrally compressed, also, when seen in profile, the eye is set above the midpoint of the gena, the clypeal psammophore of the major worker is set at around the midpoint of the clypeus to halfway between midpoint and anterior margin, and that of media and minor workers from below the midpoint to just above the anterior margin. The head, body and legs of the minor worker are strongly pubescent, all worker subcastes having many short, unmodified, prickly, erect setae on the head and body with a couple to a moderate number of such setae also distributed along the antennal scapes. However, these setae are mainly or wholly absent from the tibiae. The propodeum has an obliquely declivitous face. Small members of the *M. mjobergi* clade which have many unmodified erect setae may be confused with this species, but major workers of *M. compactus*, *mjobergi* and *M. postlei* have only one preapical metatibial spine, and the head of the major worker of *M. sericothrix* is very typically that of the *M. fieldi* complex members and not at all like the head of the major worker of M. *compactus*, *M. mjobergi* or *M. postlei*.

#### Minor worker description.

**Head.** Head rectangular; posterior margin of head planar or weakly convex; frons matt or with weak sheen, microreticulate or microreticulate-shagreenate; frons consisting of appressed pubescence, with many short, unmodified, erect setae. Eye moderate (eye length 0.20–0.49 length of side of head capsule); in full-face view, eyes set above midpoint of head capsule; in profile, eye set anteriad of midline of head capsule; eyes elliptical or slightly reniform. In full-face view, frontal carinae concave; frontal lobes straight in front of antennal insertion. Anteromedial clypeal margin broadly and evenly convex; clypeal psammophore set below midpoint of clypeus; palp formula 6,4. Five mandibular teeth in minor worker; mandibles triangular, weakly incurved; third mandibular tooth distinctly shorter than apical tooth and teeth numbers two and four; masticatory margin of mandibles approximately vertical or weakly oblique. **Mesosoma.** Integument of pronotum, mesonotum and mesopleuron matt with indistinct shagreenate sculpture throughout; anterior mesosoma in profile weakly elevated anteriad, thereafter gently sinuate, pronotum and mesonotum on same plane; appearance of erect pronotal setae short, (i.e., longest erect setae shorter than length of eye) and unmodified; in profile, metanotal groove a narrow but deep slit; propodeum matt or with a weak sheen and indistinctly shagreenate; propodeum smoothly rounded or with indistinct angle, or angulate, propodeal angle blunt; length ratio of propodeal dorsum to its declivity about 1:1, or not applicable, propodeal dorsum and declivity confluent; erect propodeal setae present and abundant (greater than 12); appressed propodeal setulae long and closely aligned, creating pubescence; propodeal spiracle situated on or beside declivitous face of propodeum, and shorter (length < 0.50 × height of propodeum). **Petiole.** In profile, petiolar node squamiform; in full-face view, shape of petiolar node uniformly rounded; node matt and microreticulate. **Gaster.** Gaster weakly shining with indistinct shagreenation; pilosity of first gastral tergite consisting of thick, appressed setae that form pubescence, interspersed with numerous short, bristly, erect setae. **General characters.** Colour concolorous smoky, dark greyish-brown to black or variegated blackish-brown and tan.

#### Major worker description.

**Head.** Head square; posterior margin of head planar or weakly concave; cuticle of frons striolate anteriad, smooth and shining posteriad; pilosity of frons a mixture of a few well-spaced, erect setae interspersed with appressed setae only. Eye moderate (eye length 0.20–0.49 length of head capsule); in full-face view, eyes set above midpoint of head capsule; in profile, eye set anteriad of midline of head capsule; eyes elliptical. In full-face view, frontal carinae concave; frontal lobes straight in front of antennal insertion. Anterior clypeal margin broadly and evenly convex; clypeal psammophore set below midpoint of clypeus; palp formula 6,4. Five mandibular teeth in major worker; mandibles triangular, weakly incurved; third mandibular tooth distinctly shorter than apical tooth and teeth numbers two and four; masticatory margin of mandibles approximately aligned vertically or weakly oblique. **Mesosoma.** Integument of pronotum, mesonotum and mesopleuron moderately shining and shagreenate throughout; anterior mesosoma in profile broadly convex; erect pronotal setae short, (i.e., shorter than length of eye) and unmodified; in profile, metanotal groove shallow, broadly V- or U-shaped; propodeum matt or with weak sheen and microreticulate-striolate; propodeum angulate, propodeal angle blunt; length ratio of propodeal dorsum to its declivity between 1:1 and1:2; erect propodeal setae present and abundant (at least a dozen); appressed propodeal setae long and closely aligned, creating pubescence; propodeal spiracle situated on or beside declivitous face of propodeum, and shorter (length less than 0.50 × height of propodeum). **Petiole.** In profile, petiolar node squamiform; in full-face view, shape of petiolar node uniformly rounded; node shining and faintly shagreenate-microreticulate. **Gaster.** Gaster weakly shining with indistinct shagreenation; pilosity of first gastral tergite consisting of a mixture of curved, erect and semi- erect setae and decumbent setae that form a variable pubescence. **General characters.** Colour of foreparts orange to dark tan, gaster dark brown.

#### Measurements.

Worker (n = 8): CI 87–120; EI 18–33; EL 0.17–0.26; HL 0.58–1.20; HW 0.50–1.44; ML 0.80–1.66; MTL 0.51–1.00; PpH 0.07–0.16; PpL 0.30–0.68; S 84–149I; SL 0.75–1.22.

#### Comments.

This hirsute member of the genus is identifiable by the combination of many mostly short, erect, unmodified setae on the body and antennal scapes but absent from the legs, the placement of the clypeal psammophore and the presence of more than one preapical spur on the metatibiae. Sequencing of old material has been unsuccessful, but the ant is here presumed to belong to the *M. fieldi* complex despite its superficial resemblance to several members of the *M. mjobergi* complex. Populations of the ant are known from southwestern Australia and southeastern SA, but it probably also occurs in suitable habitat in northern Victoria and southwestern NSW. One mention of ‘mallee woodland’ is the only ecological note, but specimens from southwestern Australia collected at Worsley and Boddington would have come from the tall sclerophyll woodland found in these localities. As with most other *Melophorus* the habits of this ant are unstudied.

#### Etymology.

Compound of Latin *sericus* (‘silky’) plus Greek *thrix* (‘hair’).

**Figure 69.**
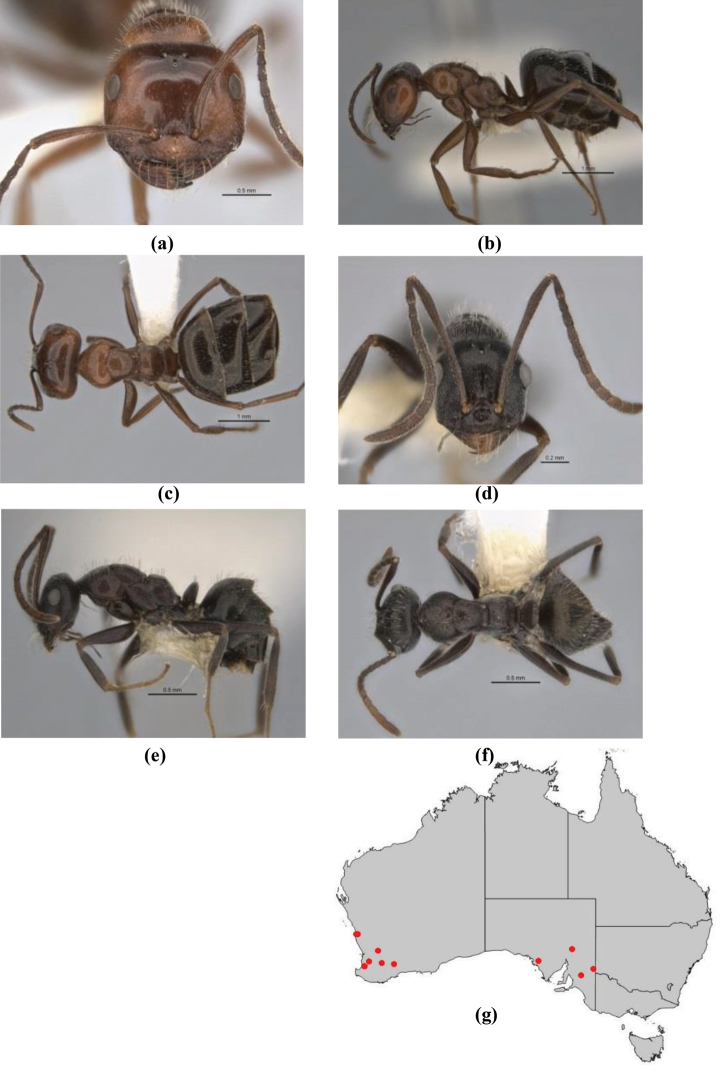
*Melophorus sericothrix* sp. n.: major worker paratype (ANIC32-900103–top ant) frons (**a**), profile (**b**) and dorsum (**c**); minor worker holotype (ANIC32-900174–bottom ant) frons (**d**), profile (**e**) and dorsum (**f**); distribution map for the species (**g**). Low resolution scale bars: 1 mm (**b, c**); 0.5 mm (**a, e, f**); 0.2 mm (**d**).

### Melophorus setosus

Taxon classificationAnimaliaHymenopteraFormicidae

Heterick, Castalanelli & Shattuck sp. n.

http://zoobank.org/B45186F6-A87F-4F20-9870-AE108EE8E81C

#### Types.

Holotype minor worker (middle ant) from Colyer Creek, 8 km N of Alice Springs 23°37'S, 133°52'E, Northern Territory, 25 January 1991, S. Shattuck #2180-7 [ANIC32-066657] (ANIC). Paratypes: Major worker and minor worker on same pin with same details as holotype (ANIC); 3 minor workers from 27 km SE of Katherine, Northern Territory, 8 April 1978, P.J.M. Greenslade, (5) (MCZ); minor worker from Ethel Creek, Western Australia, 1993-4, P.A. Varris, ‘*Melophorus* sp. O’, 96 [JDM32-004549] (WAM).

#### Other material examined.

**Queensland**: Mt Pollux, SW base (Wright, S.G.), Mt Pollux, SW base (Wright, S.G.).

#### Diagnosis.

*Melophorus setosus* can be placed in the *M. biroi* species-group on the basis of characters of the clypeus, propodeum, mandible and palps. The species is also placed in the *M. fieldi* species-complex because of the appearance of the anteriorly placed clypeal psammophore, the compact propodeum, the presence of more than one preapical spine on the metatibia, at least in the major worker, the long, even spindly legs, and the unmodified mandible in the major worker. The vestiture of short modified setae that clothe the head and body of this ant (but not the legs or antennal scape), and the distinctive frontal carinae that are straight or weakly convex and converge anteriad rather than diverging, as in most *Melophorus*, make this species unmistakeable.

#### Minor worker description.

**Head.** Head square; posterior margin of head weakly convex; frons matt or with weak sheen, microreticulate or microreticulate-shagreenate; frons consisting mainly of stout, appressed and erect setae, both sets of setae modified and thickened and occasionally clavate (featherlike) in appearance. Eye moderate (eye length 0.20–0.49 length of side of head capsule); in full-face view, eyes set above midpoint of head capsule; in profile, eye set anteriad of midline of head capsule; eyes elliptical or slightly reniform. In full-face view, frontal carinae straight, divergent posteriad; frontal lobes curved inward in front of antennal insertion. Anteromedial clypeal margin broadly and evenly convex and protrusive; clypeal psammophore set below midpoint of clypeus; palp formula 6,4. Five mandibular teeth in minor worker; mandibles triangular, weakly incurved; third mandibular tooth distinctly shorter than apical tooth and teeth numbers two and four; masticatory margin of mandibles approximately vertical or weakly oblique. **Mesosoma.** Integument of pronotum, mesonotum and mesopleuron with weak to moderate sheen, shagreenate on pronotum and dorsum of mesonotum, otherwise microreticulate; anterior mesosoma in profile smoothly rounded anteriad, thereafter pronotum and whole of mesonotum flattened and on same plane as propodeum; appearance of erect pronotal setae short and unmodified, or weakly expanded distally; in profile, metanotal groove shallow, broadly V or U-shaped; propodeum shining and uniformly striolate; propodeum angulate, propodeal angle blunt; length ratio of propodeal dorsum to its declivity about 3:2; erect propodeal setae present and abundant (greater than 12); appressed propodeal setulae long, each reaching setae behind and in front, but not forming pubescence; propodeal spiracle situated at least twice its width from the declivitous face of propodeum, and shorter (length < 0.50 × height of propodeum). **Petiole.** In profile, petiolar node squamiform; in full-face view, shape of petiolar node uniformly rounded; node shining and smooth with vestigial sculpture. **Gaster.** Gaster shining, shagreenate (‘LP record’ appearance); pilosity of first gastral tergite consisting of thick, often distally flattened, erect setae over well-spaced, short, appressed setae. **General characters.** Colour of foreparts orange tan to brick red (head may be darker), gaster black with or without violet iridescence.

#### Major worker description.

**Head.** Head horizontally rectangular, broader than wide; posterior margin of head weakly concave; cuticle of frons matt or with weak sheen, indistinctly shagreenate; frons consisting mainly of appressed and stout erect setae, the latter bristly in appearance and distinctly modified (flattened) distally. Eye moderate (eye length 0.20–0.49 length of head capsule); in full-face view, eyes set above midpoint of head capsule; in profile, eye set anteriad of midline of head capsule; eyes elliptical. In full-face view, frontal carinae straight or weakly convex; frontal lobes curved inward in front of antennal insertion. Anterior clypeal margin broadly and evenly convex; clypeal psammophore set at or above midpoint of clypeus; palp formula 6,4. Five mandibular teeth in major worker; mandibles triangular, weakly incurved; third mandibular tooth distinctly shorter than apical tooth and teeth numbers two and four; masticatory margin of mandibles approximately aligned vertically or weakly oblique. **Mesosoma.** Integument of pronotum, mesonotum and mesopleuron moderately shining and shagreenate throughout; anterior mesosoma in profile broadly convex; erect pronotal setae short and often expanded distally, at times clavate; in profile, metanotal groove deep, V-shaped; propodeum shining, with multiple hair-like striolae; propodeum smoothly rounded or with indistinct angle; propodeal dorsum and declivity confluent; erect propodeal setae present and sparse to moderate (1-12); appressed propodeal setae long and closely aligned, creating pubescence; propodeal spiracle situated at least twice its width from the declivitous face of propodeum, and shorter (length less than 0.50 × height of propodeum). **Petiole.** In profile, petiolar node squamiform; in full-face view, shape of petiolar node tapered with squared-off vertex; node shining and faintly shagreenate-microreticulate. **Gaster.** Gaster shining, shagreenate (‘LP record’ appearance); pilosity of first gastral tergite consisting of thick, often distally flattened, erect setae over well-spaced, short, appressed setae. **General characters.** Colour of foreparts orange tan, gaster black.

#### Measurements.

Worker (n = 4): CI 108–118; EI 22–28; EL 0.26–0.33; HL 0.86–1.27; HW 0.93–1.49; ML 1.20–1.52; MTL 0.78–0.93; PpH 0.17–0.17; PpL 0.48–0.61; SI 75–105; SL 0.98–1.11.

#### Comments.

The rather uncommon but widespread *Melophorus setosus* is well represented in the TERC Collection (as ‘Group H’) with specimens from northern NT, QLD and WA. Material in other collections has come from NT (ANIC), QLD (QM) and WA (WAM). This species has a distinctive *facies*, with straight rather than concave frontal carinae and stout, modified setae that are especially abundant in the minor worker. This combination enables the ant to be distinguished from all other *Melophorus*, although the bulbous clypeus associates it superficially with *M. caeruleoviolaceus*. This ant is possibly also more distantly related to *M. solitudinis*. Andersen (2007) notes the apparent association of this ant with *Monomorium rothsteini* (‘*rothsteini* group’ in Andersen) nests and queries whether this may be another nest raider. Queensland Museum specimens were hand collected as foragers in brigalow scrub. No other data are known for this species, and no samples have been available for sequencing.

#### Etymology.

Latin *setosus* (‘bristly’); adjective in the nominative singular.

**Figure 70.**
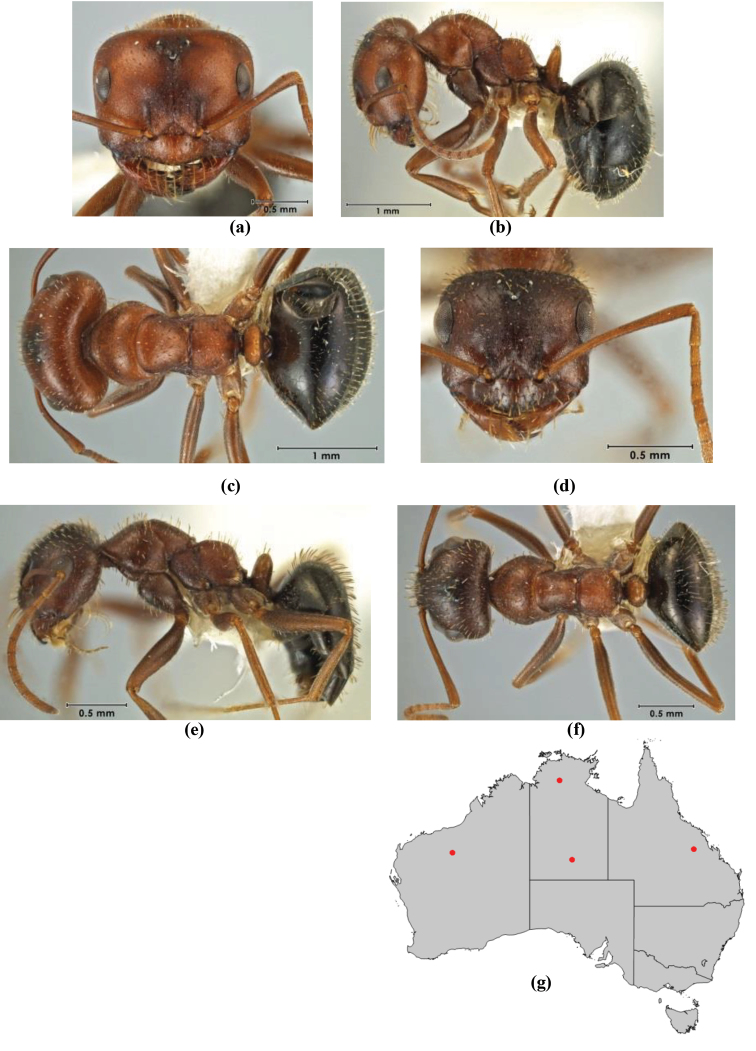
*Melophorus setosus* sp. n.: major worker paratype (ANIC32-066657–top ant) frons (**a**), profile (**b**) and dorsum (**c**); minor worker holotype (ANIC32-066657–middle ant) frons (**d**), profile (**e**) and dorsum (**f**); distribution map for the species (**g**).

### Melophorus solitudinis

Taxon classificationAnimaliaHymenopteraFormicidae

Heterick, Castalanelli & Shattuck sp. n.

http://zoobank.org/321DB31F-BFC7-4640-975C-E988F2DB06C3

#### Types.

Holotype minor worker from Tropicana Minesite 29°20'27"S, 124°29'08"E, Western Australia, January 2009, J. Summerhayes, pitfall; trap: mallee MA2:1 [JDM32-004513] (WAM). Paratypes: major and minor worker from 16 miles ENE of Cosmo Newbery [*sic*] Mission, Western Australia, 14 October 1960, McInnes & Dowse [ANIC32-066647] (ANIC).

#### Diagnosis.

*Melophorus solitudinis* can be placed in the *M. biroi* species-group on the basis of characters of the clypeus, propodeum, mandible and palps. The species is also placed in the *M. fieldi* species-complex because of the appearance of the anteriorly placed clypeal psammophore, the compact propodeum, the presence of more than one preapical spine on the metatibia, at least in the major worker, the long, even spindly legs, and the unmodified mandible in the major worker. In *Melophorus solitudinis* the body of both the major and the minor worker are clothed with modified, erect setae (distally thickened, clavate or spatulate) to various degrees in addition to unmodified setae. In full-face view, the heads of the major and minor worker are matt with microreticulate sculpture. These features separate this taxon from most others. The major worker of this species is more densely sculptured than are *M. bruneus* and *M. fieldi*, the *M. solitudinis* minor worker is much larger than the minor workers of those two species, and its weakly divergent frontal carinae distinguish this ant from *M. setosus*.

#### Minor worker description.

**Head.** Head square; posterior margin of head planar or weakly concave; frons matt or with weak sheen, microreticulate or microreticulate-shagreenate; pilosity of frons a mixture of short, erect and semi-erect setae interspersed with shorter decumbent setae and well-spaced, short, appressed setae. Eye moderate (eye length 0.20–0.49 length of side of head capsule); in full-face view, eyes set above midpoint of head capsule; in profile, eye set anteriad of midline of head capsule; eyes elliptical or slightly reniform. In full-face view, frontal carinae straight or weakly convex; frontal lobes straight in front of antennal insertion. Anteromedial clypeal margin broadly and evenly convex; clypeal psammophore set below midpoint of clypeus; palp formula 6,4. Five mandibular teeth in minor worker; third mandibular tooth distinctly shorter than apical tooth and teeth numbers two and four; masticatory margin of mandibles approximately vertical or weakly oblique. **Mesosoma.** Integument of pronotum, mesonotum and mesopleuron matt or with weak sheen and microreticulate throughout; anterior mesosoma in profile broadly convex; appearance of erect pronotal setae short and often expanded distally, at times clavate; in profile, metanotal groove shallow, broadly V or U-shaped; propodeum matt or with a weak sheen and microreticulate; propodeum angulate, propodeal angle blunt; length ratio of propodeal dorsum to its declivity about1:1; erect propodeal setae present and abundant (greater than 12); appressed propodeal setulae long and separated by at least own length; propodeal spiracle situated on or beside declivitous face of propodeum, and longer (length ≥ 0.50 × height of propodeum). **Petiole.** In profile, petiolar node squamiform; in full-face view, shape of petiolar node tapered with blunt vertex; node matt and microreticulate. **Gaster.** Gaster weakly shining and microreticulate; pilosity of first gastral tergite consisting of well-spaced, short, thick, erect setae over long, closely aligned, whitish, appressed setae. **General characters.** Colour of foreparts and legs orange-tan, gaster brown.

#### Major worker description.

**Head.** Head square; posterior margin of head planar or weakly concave; cuticle of frons matt or with weak sheen, microreticulate; frons consisting mainly of appressed and stout erect setae, the latter bristly in appearance and distinctly modified (flattened) distally. Eye moderate (eye length 0.20–0.49 length of head capsule); in full-face view, eyes set above midpoint of head capsule; in profile, eye set anteriad of midline of head capsule; eyes elliptical. In full-face view, frontal carinae straight, divergent posteriad; frontal lobes straight in front of antennal insertion. Anterior clypeal margin broadly and evenly convex; clypeal psammophore set at or above midpoint of clypeus; palp formula 6,4. Five mandibular teeth in major worker; third mandibular tooth distinctly shorter than apical tooth and teeth numbers two and four; masticatory margin of mandibles approximately aligned vertically or weakly oblique. **Mesosoma.** Integument of pronotum, mesonotum and mesopleuron matt or with weak sheen and microreticulate throughout; anterior mesosoma in profile broadly convex; erect pronotal setae short and often expanded distally, at times clavate; in profile, metanotal groove shallow, broadly V- or U-shaped; propodeum matt or with weak sheen and microreticulate-striolate; propodeum angulate, propodeal angle blunt; length ratio of propodeal dorsum to its declivity about 1:1; erect propodeal setae present and abundant (at least a dozen); appressed propodeal setae sparse or absent, if present then not regularly spaced; propodeal spiracle situated at least twice its width from the declivitous face of propodeum, and shorter (length less than 0.50 × height of propodeum). **Petiole.** In profile, petiolar node squamiform; in full-face view, shape of petiolar node tapered with blunt vertex; node shining and faintly shagreenate-microreticulate. **Gaster.** Gaster shining with superficial microreticulation; pilosity of first gastral tergite consisting of longish, closely aligned, appressed setae interspersed with short, bristly, erect setae (some distally flattened). **General characters.** Colour of foreparts and legs orange-tan, gaster brown.

#### Measurements.

Worker (n = 2): CI 109–117; EI 19–24; EL 0.26–0.34; HL 0.99–1.37; HW 1.08–1.59; ML 1.34–1.67; MTL 0.89–1.09; PpH 0.17–0.24; PpL 0.54–0.71; SI 78–99; SL 1.07–1.24.

#### Comments.

*Melophorus solitudinis* is a fairly squat, dull species that is characterized by a combination of erect setae on the tibiae, modified setae on the head and mesosoma and densely shagreenate sculpture of the cuticle. The appearance of the setae is not unlike that found in *M. setosus*, but the two species are unlikely to be closely related. The ant is known from just three workers from two sites in inland WA. Despite the paucity of material, the ant may not be rare: because of its remote habitat it is unlikely to be seen by the majority of collectors. Nothing is known of its habits.

#### Etymology.

Latin *solitudinis* (‘loneliness’): a reference to the ant’s remote habitat; adjective in the nominative case.

**Figure 71.**
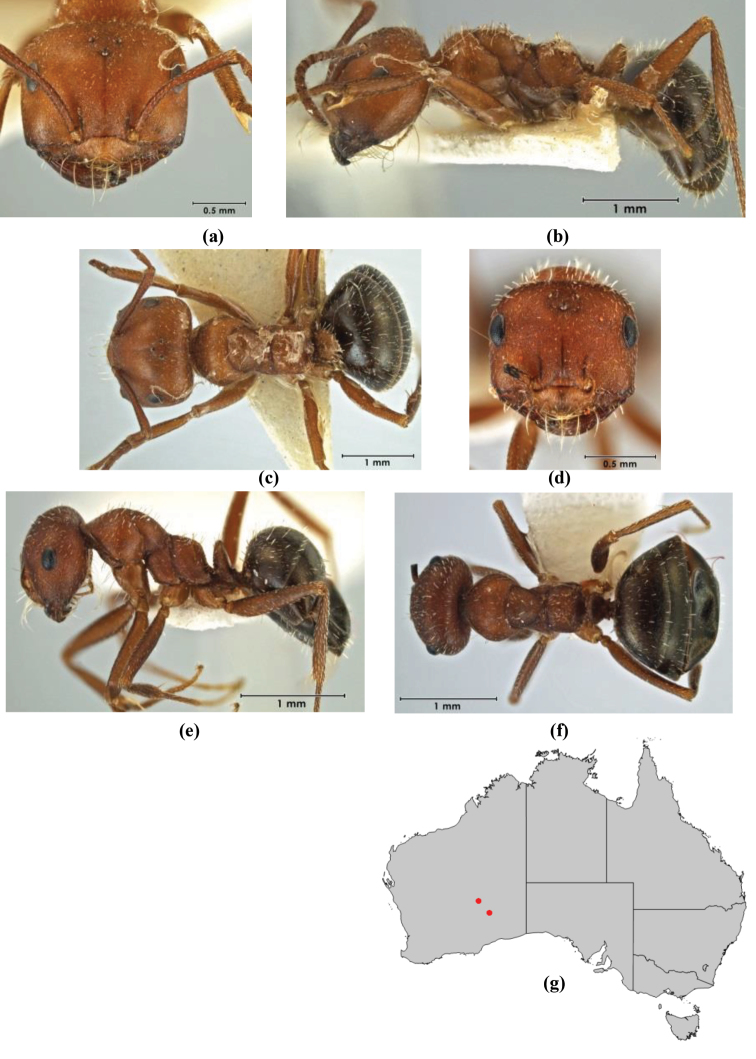
*Melophorus solitudinis* sp. n.: major worker paratype (ANIC32-066647–bottom ant) frons (**a**), profile (**b**) and dorsum (**c**); minor worker holotype (JDM32-004513) frons (**d**), profile (**e**) and dorsum (**f**); distribution map for the species (**g**).

### Melophorus sulla

Taxon classificationAnimaliaHymenopteraFormicidae

Forel, Status novus

Melophorus ludius subsp. sulla Forel 1910: 66.
Melophorus ludius r. sulla v. breviscapa Forel 1915: 89. Unavailable name (Taylor 1986)

#### Types.

Syntype major workers, Tennants [*sic*] Creek, Northern Territory [ANIC] [MHNG], the ANIC major worker here designated lectotype. The MHNG major worker (CASENT0909817) is here designated a paralectotype. (See also ‘Comments’ below for other possible paralectotypes.)

#### Other material examined.

**Northern Territory**: 10 mi W Narwietooma Homestead (McInnes & Dowse), 11 km N Tennant Creek (Davidson, D. & Morton, S.), 20 km S Alice Springs (Greenslade, P.J.M.), 25 km SSW The Granites, Tanami Desert (Morton, S. & Greenslade, P.J.M.), 5 km SE Anthony’s Lagoon (Davidson, D. & Morton, S.), 8 mi W Mulga Park Homestead (McInnes & Dowse), about 3 km W Alice Springs (Feehan, J.E. [ANIC32-900144]), about 5 km N Henbury Homestead (Feehan, J.E.), Kunoth Paddock, near Alice Springs (Greenslade, P.J.M.), Kunoth Paddock, near Alice Springs (Greenslade, P.J.M.), Kunoth Paddock, near Alice Springs (Greenslade, P.J.M.), Manbulloo Research Station (Gross, G.), Manbulloo, SW Katherine (Greenslade, P.J.M.), Manbulloo, SW Katherine (Greenslade, P.J.M.), Newcastle Waters (Chinnick, L.), Tanami (Greenslade, P.J.M.), Tanami (Greenslade, P.J.M.), Tanami (Greenslade, P.J.M.), Tanami (Greenslade, P.J.M.), Tanami (Greenslade, P.J.M.), Tanami (Greenslade, P.J.M.), Tanami (Greenslade, P.J.M.), Tanami (Greenslade, P.J.M.), Tanami Desert (Greenslade, P.J.M.), Tennant Creek. **Queensland**: ‘Gumbardo’ (Beutel, T.), 13 km from S-bend on Plum Pudding Track (Lemann, C. [ANIC32-035482]), 2 mi NE Quamby (Dowse, J.E.), Sandringham Station, 55 km NW Bedourie (Morton, S.R.). **South Australia**: 10 mi WNW Mt Wooltarlinna (McInnes & Dowse), 10 km E Mt Ive Homestead, Gawler Ranges (Greenslade, P.J.M.), 12 km W Emu, Victoria Desert (Greenslade, P.J.M.), 12 km W Emu, Victoria Desert (Greenslade, P.J.M.), 18 mi W Mt. Morris (McInnes & Dowse), 20 km NE Macumba Station (Davidson, D. & Morton, S.), 30 km E Poeppel Corner, Simpson Desert (Greenslade, P.J.M.), 31 mi SSW Observatory Hill (McInnes & Dowse), 37 mi W Victory Downs Homestead (McInnes & Dowse), 40 km WNW Emu, Victoria Desert (Greenslade, P.J.M.), 4 km W Purni Bore, SW Simpson Desert (Greenslade, P.J.M.), 50 km S Coober Pedy (Davidson, D. & Morton, S.), 53 km E Vokes Hill, Victoria Desert (Greenslade, P.J.M.), 53 km E Vokes Hill, Victoria Desert (Greenslade, P.J.M.), 5 km SE Oodnadatta (Davidson, D. & Morton, S.), 5 km SE Oodnadatta (Davidson, D. & Morton, S.), 85 km W Mabel Creek (Greenslade, P.J.M.), about 6.5 km N Leigh Creek (Feehan, J.E.), about 60 km N Oodnadatta (Feehan, J.E.), Cambrai (Greenslade, P.J.M.), Cambrai (Greenslade, P.J.M.), Cambrai (Greenslade, P.J.M.), Emu Camp, Victoria Desert (Greenslade, P.J.M.), Emu Camp, Victoria Desert (Greenslade, P.J.M.), Hookina Creek, Flinders Ranges (Greenslade, P.J.M.), Koonamore (Greenslade, P.J.M.), Koonamore, 2 km NE homestead (Greenslade, P.J.M.), Lake Meramangye, Victoria Desert (Greenslade, P.J.M.), Mt Gunson, SE Woomera (Greenslade, P.J.M.), Victoria Desert (Greenslade, P.J.M.). **Victoria**: 15 km WNW Yaapeet (Andersen, A.N.). **Western Australia**: 100 km EbyN Cosmo Newberry (Feehan, J.E.), 11 km N Leonora (Davidson, D. & Morton, S.), 13 mi SSE Roy Hill Homestead (McInnes & Dowse), 14 km E Roebuck Plains RH (Heterick, B.E. [M188]), 150 km SW Giles Meteorological Station (Heatwole, H.), 16 km S Mt Magnet (Heterick, B.E. [M290]), 16 km SbyW Onslow (Feehan, J.E.), 18.5 km E Southern Cross (Heterick, B.E. [M136]), 19 km N Hines Hill (Heterick, B.E. [M16]), 26 mi NWbyW Norseman (Taylor, R.W.), 28 mi SE Roebourne (McInnes & Dowse), 3 km E Fitzroy Crossing (Heterick, B.E. [M196]), 30 km S of ‘The Overlander’ (Heterick, B.E. [JDM32-001541]), 31 mi WNW Mt Davies, Tomkinson Range (McInnes & Dowse), 33 mi SE Giles (McInnes & Dowse), 37 mi SW Mundiwindi (McInnes & Dowse [ANIC32-900050]), 38 mi NE Giles (McInnes & Dowse), 40 mi NNE Giles (McInnes & Dowse), 43 mi WNW Wiluna (McInnes & Dowse), 47 km E Fitzroy Crossing (Heterick, B.E. [M236]), 5 km N Cunyu Station (Davidson, D. & Morton, S.), 60 km N Ajana (Upton, M.S.), Barrow Isle [Barrow I.] (Heatwole, H. [JDM32-001548]), Bungalbin Hill, E Goldfields (Humphries, W.F. [JDM32-001551]), Coomallo Downs (Heterick, B.E. [M41]), County Downs Hsd (south boundary) (Heterick, B.E. [M249]), Ellendale Stn turnoff (Heterick, B.E. [M193/M194/M195]), G. J. Rd, 90 km E Carnarvon (Heterick, B.E. [M312]), Gantheaume Point (Broome) (Heterick, B.E. [JDM32-001540]), Hines Hill (Heterick, B.E. [JDM32-001543]), Int. Holland Tr./Norseman Rd. (Heterick, B.E. [JDM32-001542]), Kanka water hole, near Warburton (Heatwole, H. & Greenslade, P.J.M.), Meekatharra (Mercovich, C.), Mulga, NE Goldfields (Pringle, H.J.R. [ANIC32-029630]), Mulga, NE Goldfields (Pringle, H.J.R. [ANIC32-029631]), Nita Downs turnoff (Heterick, B.E. [M261]), Northam (Majer, J.D. [JDM32-001545]), nr 300 km peg N Sandfire (Heterick, B.E. [M263]), Pardoo Stn turnoff (Heterick, B.E. [M267]), Paynes Find (Heterick, B.E. [JDM32-001539]), Pollock Hills ['Pollack hills'], 32 mi WNW Mt Webb (McInnes & Dowse), Rabbit Proof Fence Rd. (Heterick, B.E. [JDM32-004756]), Willare Bridge (Heterick, B.E. [M189]), Yamarna Homestead (Richardson, S. [ANIC32-032251]).

#### Diagnosis.

*Melophorus sulla* can be placed in the *M. biroi* species-group on the basis of characters of the clypeus, propodeum, mandible and palps. The species is also placed in the *M. fieldi* species-complex because of the appearance of the anteriorly placed clypeal psammophore, the compact propodeum, the presence of more than one preapical spine on the metatibia, at least in the major worker, and the long, even spindly legs. Most major workers of *M. sulla* have an unmodified mandible but a few majors from WA have a crushing mandible such as is seen in *M. wheeleri* species-complex major workers. (see below). *Melophorus sulla* (***sensu stricto***) usually has glabrous workers in all subcastes, although one or two flexuous, erect setae may be seen on the mesosoma. The appressed setae on the gaster of all workers are very small and inconspicuous when the gaster is moderately distended, such setae being separated from one another by at least their own length. These appressed setae are also inconspicuous on the mesosoma and never long and silvery. The node of the minor worker is often squamiform, and most commonly the cuticle is shining or even glossy with vestigial or weak shagreenation. The species is distinguished from similar species like *M. turneri* or *M. longipes* by its light yellow or ochre colour, some populations having a darker, brownish gaster along with the pale foreparts. One variant morph has a major worker with a modified, crushing mandible but the taxonomic significance of this modification is not known, especially since the minor workers of this morph are indistinguishable from other *M. sulla* minors. The “*pillipes*’’ condition has not been seen in this species.

#### Minor worker description.

**Head.** Head square; posterior margin of head strongly convex; frons shining with superficial shagreenation or microreticulation only; frons consisting exclusively or almost exclusively of well-spaced, appressed setae only (small, erect setae, if present, usually confined to ocular triangle or posterior margin of head). Eye large (eye length ≥ 0.50 × length of side of head full-face view, eyes set at about midpoint of head capsule; in profile, eye set around midline of head capsule; eyes elliptical or slightly reniform, or elongate. In full-face view, frontal carinae concave; frontal lobes straight in front of antennal insertion. Anteromedial clypeal margin broadly and evenly convex; clypeal psammophore set at or above midpoint of clypeus; palp formula 6,4. Five to six mandibular teeth in minor worker; mandibles triangular, weakly incurved, or narrow, strap-like, internal and external margins parallel or nearly so; third mandibular tooth distinctly shorter than apical tooth and teeth numbers two and four; masticatory margin of mandibles approximately vertical or weakly oblique. **Mesosoma.** Integument of pronotum, mesonotum and mesopleuron shining and mainly smooth, vestigial shagreenation most noticeable on humeri and mesopleuron; anterior mesosoma in profile broadly convex; erect pronotal setae absent; in profile, metanotal groove shallow, broadly V or U-shaped; propodeum shining and smooth or with superficial and almost invisible microsculpture; propodeum smoothly rounded or with indistinct angle; propodeal dorsum and declivity confluent; erect propodeal setae always absent; appressed propodeal setulae sparse or absent, if present then not regularly spaced; propodeal spiracle situated on or beside declivitous face of propodeum, and longer (length ≥ 0.50 × height of propodeum). **Petiole.** In profile, petiolar node narrowly conical, vertex blunt, directed posteriad; in full-face view, shape of petiolar node uniformly rounded; node shining and smooth throughout. **Gaster.** Gaster shining, shagreenate (‘LP record’ appearance); pilosity of first gastral tergite consisting of well-spaced short, inconspicuous, appressed setae, erect setae (present in at least some workers) confined to margin of sclerite. **General characters.** Colour pale yellow-orange (in full-face view, lower frons often visibly depigmented).

#### Major worker description.

**Head.** Head horizontally rectangular, broader than wide; posterior margin of head weakly concave; cuticle of frons shining with superficial shagreenation or microreticulation only; frons consisting exclusively or almost exclusively of well-spaced, appressed setae only (small, erect setae, if present, usually confined to ocular triangle or posterior margin of head). Eye moderate (eye length 0.20–0.49 length of head capsule); in full-face view, eyes set above midpoint of head capsule; in profile, eye set anteriad of midline of head capsule; eyes elliptical. In full-face view, frontal carinae straight, divergent posteriad; frontal lobes straight in front of antennal insertion. Anterior clypeal margin broadly concave; clypeal psammophore set at or above midpoint of clypeus; palp formula 6,4. Mandibular teeth in major worker always 4; mandibles strongly incurved, apical sector weakly carinate or incompletely carinate; third mandibular tooth distinctly shorter than apical tooth and teeth numbers two and four; masticatory margin of mandibles approximately aligned vertically or weakly oblique. **Mesosoma.** Integument of pronotum, mesonotum and mesopleuron moderately shining and shagreenate throughout; anterior mesosoma in profile broadly convex; erect pronotal setae absent; in profile, metanotal groove shallow, broadly V- or U-shaped; propodeum shining and shagreenate; propodeum smoothly rounded or with indistinct angle; propodeal dorsum and declivity confluent; erect propodeal setae absent; appressed propodeal setae short, separated by more than own length and inconspicuous; propodeal spiracle situated at least twice its width from the declivitous face of propodeum, and shorter (length less than 0.50 × height of propodeum). **Petiole.** In profile, petiolar node squamiform; in full-face view, shape of petiolar node tapered with sharp vertex; node shining and smooth with vestigial microreticulation anteriad. **Gaster.** Gaster weakly shining with indistinct shagreenation; pilosity of first gastral tergite consisting of thick, often distally flattened, erect setae over well-spaced, short, appressed setae. **General characters.** Colour yellow-orange (in full-face view, lower frons often visibly depigmented).

#### Measurements.

Worker (n = 8): CI 87–116; EI 17–33; EL 0.14–0.28; HL 0.50–1.46; HW 0.43–1.69; ML 0.69–1.79; MTL 0.49–1.24; PpH 0.06–0.17; PpL 0.30–0.72; SI 71–151; SL 0.65–1.20.

#### Comments.

The characteristically pallid *Melophorus sulla*, which occurs in all mainland Australian states, poses more taxonomic problems than any other *Melophorus* except, perhaps, *M. biroi*. Not the least of these problems is the fact that while the *facies* of most major workers are typical of the *M. fieldi* complex, a few workers from midwestern WA have the broad heads and crushing mandible of the *M. wheeleri* complex. More frequently, the clypeus, especially in major workers, exhibits considerable plasticity; in some cases (mainly in far northern and arid area populations) this sclerite is flattened with a posteriorly placed clypeal psammophore, in other cases it is more-or-less protuberant with the psammophore in the middle of the sclerite. Those workers with a very protuberant clypeus tend to be of deeper colour than the very pale, depigmented individuals with the flattened clypeus. The most common colour form is a concolorous light yellow-ochre with a two-toned head capsule (darker above and conspicuously paler around the lower genae and the clypeus). This is the form that was given the unavailable name *Melophorus ludius sulla breviscapa* by Forel. Other workers may be uniformly canary yellow or yellow with orange tinges on the head and a light brown gaster. The minor workers of all morphotypes are more uniform in appearance than their major workers and have long spindly appendages, similar to those of *M. hirsutipes* and *M. lanuginosus*. However, although closely related, *M. sulla* does not appear to be a sister group to these taxa.

The typology of the taxon is also illustrative of outdated taxonomic practice. Two specimens that appear to be part of the original type series examined by Forel are listed and noted at the head of this section. Both individuals are major workers, and possess a red ‘typus’ label. The ANIC specimen is here designated lectotype to fix the name for this problematic species; the other specimen, held by MHNG, becomes a paralectotype. Forel also examined an unspecified number of queens and males in the original type series. The latter, if they bear a red ‘typus’ label, also become paralectotypes in this revision. In addition to these specimens a pin of two major workers held by SAMA and a major worker held by MSNG are labeled ‘co-type.’ Since the collection data are identical, these specimens may have been regarded by Forel as the equivalent of paratypes, but they are not mentioned as such in his original description and it is not known if they were part of the original group of specimens that he saw. Since the information is equivocal, no paralectotype label has been attached to these specimens. However, should the designated lectotype and paralectotypes become lost or destroyed they could be reconsidered for paralectotype and thus for neotype status (ICZN, section 74F). Definitely removed from the type series are two sets of minor workers labeled as ‘co-types’, one of which is held by MCZ and the other (two workers) by SAM. Forel explicitly states he saw no minor workers of this species in the consignment. In fact, the SAMA workers are *M. wheeleri*!

As previously discussed, the genetics of this species are confusing. The mitochondrial gene suggests several populations; however the morphology and the nuclear genes don’t support this result. Future work is needed to examine the population structure of *M. sulla* in order to determine if multiple species exist. No samples of the specimens with a wheeleri-type major worker were sequenced (this morphotype appears to be rare) and, apart from the major worker mandible and broad head, these ants are indistinguishable from other *M. sulla*, and the minor workers cannot be distinguished in any way from those of other populations. There is the possibility that such major workers represent no more than a rare genetic trait. Hybridization with a member of the *M. wheeleri* complex is unlikely because of the genetic distance between the *M. fieldi* and the *M. wheeleri* complexes. The two forms of *M. sulla* are separated in the taxonomic key, but the unique status of the aberrant member of the nominal species is not established, and this aspect is flagged for further investigation.

This arid or semi-arid species is active in a variety of habitats but seems to prefer very sandy areas, hence the frequent mention of ‘dunes’ on label data. However, the ant also occurs in dry watercourses and clay soils. A variety of ecosystems support this ant, and named vegetation in label data includes gidgee, mallee, *Casuarina* and *Eucalyptus dumosa*. The manner of leaving the nest is quite intriguing. The ants are extremely timid: the principal author has watched a nest entrance of this species for many minutes. The ants seemed to be most active around the middle of the day and left the nest in pulses of several to many workers, which streamed in and out to forage or deposit sand grains at very high speed. In between these pulses of activity, there was no movement around the nest. Possibly this behavior may reduce predation, with the irregular activity and speed across the hot soil surface lessening the likelihood of a predator being able to pick off workers at regular intervals. Alternately, the behavior may be related to subtle changes in solar radiation, which affects thermoregulation in the ants.

**Figure 72.**
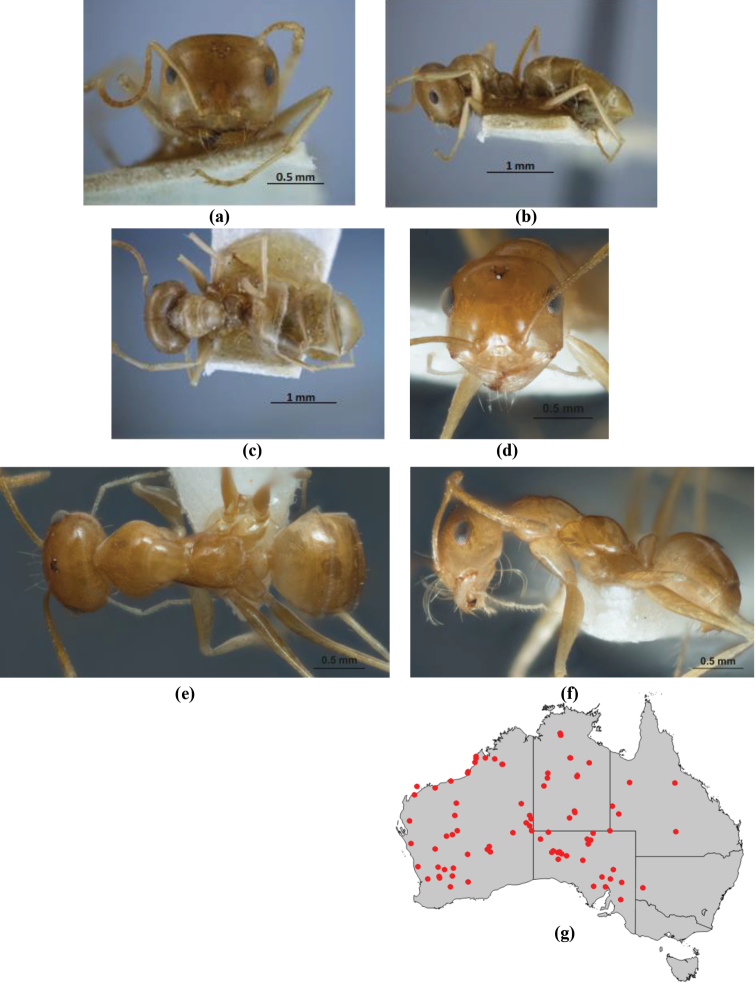
*Melophorus sulla* Forel: MCZ major worker syntype (CASENT090917) frons (**a**), profile (**b**) and dorsum (**c**); non-type minor worker (ANIC: 50 km S Coober Pedy, SA, 2 Oct. 1981, D. Davidson/S. Morton, 69, (bottom ant) frons (**d**), profile (**e**) and dorsum (**f**); distribution map for the species (**g**).

### Melophorus turneri

Taxon classificationAnimaliaHymenopteraFormicidae

Forel

Melophorus turneri Forel 1910: 63 (combination in M. (Erimelophorus) by Wheeler 1935: 71). See also Viehmeyer 1914: 43 (q.); Wheeler, G.C. & Wheeler, J. 1968: 206 (l.). Types. Syntype minor and major workers, Cape York, Queensland [ANIC, MHNG] (examined: ANIC specimen ANIC32-0534444, here designated lectotype, AntWeb images of MHNG specimens CASENT0909822, CASENT0909823 here designated paralectotypes
Melophorus turneri aesopus Forel
Melophorus turneri subsp. aesopus Forel 1910: 64. See also Clark, J. (1938). The Sir Joseph Banks Islands. Reports of the McCoy Society for Field Investigation and Research. Part 10. Formicidae (Hymenoptera). Proceedings of the Royal Society of Victoria. (n.s.) 50: 356-382. Types. Syntype major and minor workers: Tennants [*sic*] Creek, Northern Territory [ANIC, MHNG] (examined: ANIC specimen CASENT0172012, AntWeb images of MHNG specimens CASENT0909824 , CASENT0909825). **Syn. n.**
Melophorus turneri candidus Santschi
Melophorus turneri st. candida Santschi 1919: 328, fig. 2. Types. Syntype major and minor workers: Victoria [BMNH - Arnold Coll. B. M. 1934-354, NHMB] (examined: BMNH specimens, AntWeb images of NHMB specimen CASENT0172012). **Syn. n.**
Melophorus pillipes Santschi
Melophorus pillipes Santschi 1919: 329, fig. 2 (combination in M. (Erimelophorus) by Wheeler 1935: 71). Types. Syntypes of major and minor workers: Townsville, Queensland [NHMB] (examined: AntWeb images of NHMB specimens CASENT0912333, CASENT0912334). **Syn. n.**

#### Other material examined.

**Australian Capital Territory**: Black Mountain (Greaves, T.), Black Mountain (Greaves, T.), Black Mountain (general) (Billen, J. [ANIC32-013528]), Black Mountain, near CSIRO (Berg, R.Y.), Black Mountain, near Haydon Drive (Berg, R.Y.), Mt Ainslie (Berg, R.Y.), Mt Ainslie (Brooks, C.G.), Mt. Ainslie (Shattuck, S.O. [ANIC32-023694]), Mt. Pleasant (Lowery, B.B.). **New South Wales**: 10 km W Temora (Shattuck, S.O. [ANIC32-039535]), 3 mi WNW Volo Homestead, Lake Poopalloo (Greaves, T.), 9.5 km S Bungendore (Shattuck, S.O. [ANIC32-047378]), Fowlers Gap (Greenslade, P.J.M.), Mudgee (Lowery, B.B.), Mudgee, aerodrome (Lowery, B.B.), Mudgee, near aerodrome (Lowery, B.B.), Mungindi (Lowery, B.B.), near Queanbeyan, 1 mi W junction Captains Flat & Bungendore Roads (Berg, R.Y.), Newholme Road, near Armidale (Sakurai, Y.), Olive Downs Station, 55 km NW Tibooburra (Morton, S.R.), Rockley (Hamilton, J.T.), Sturt National Park (Greenslade, P.J.M.), Sturt National Park (Greenslade, P.J.M.), Sturt National Park (Greenslade, P.J.M.), Sturt National Park (Greenslade, P.J.M.), Tomago (Jackson, G.P. [ANIC32-015276]), Whiporie, 55 km S Casino (York, A.), Willandra National Park (Shattuck, S.O.). **Northern Territory**: 102 km N Yuendumu (Greenslade, P.J.M.), 105 km N Yuendumu (Greenslade, P.J.M.), 11 km N Tennant Creek (Davidson, D. & Morton, S.), 12 km SW Katherine (Greenslade, P.J.M.), 15 km SW Katherine (Greenslade, P.J.M.), 20 km SE Katherine (Greenslade, P.J.M.), 23 km SW Katherine (Greenslade, P.J.M.), 23 km SW Katherine (Greenslade, P.J.M.), 25 km W Tennant Creek (Greenslade, P.J.M.), 27 km SW Katherine (Greenslade, P.J.M.), 27 km SW Katherine (Greenslade, P.J.M.), 2 km W Katherine (Greenslade, P.J.M.), 30 km SW Katherine (Greenslade, P.J.M.), 40 km W Wave Hill (Davidson, D. & Morton, S.), 5 km S Jabiru, Alligator Rivers area (Greenslade, P.J.M.), about 56 km N Kulgera (Feehan, J.E.), about 5 km N Henbury Homestead (Feehan, J.E.), Jabiru (Greenslade, P.J.M.), Jabiru, Alligator Rivers area (Greenslade, P.J.M.), Kunoth Paddock, near Alice Springs (Greenslade, P.J.M.), Kunoth Paddock, near Alice Springs (Greenslade, P.J.M.), Kunoth Paddock, near Alice Springs (Greenslade, P.J.M.), Kunoth Paddock, near Alice Springs (Greenslade, P.J.M.), Kunoth Paddock, near Alice Springs (Greenslade, P.J.M.), Kunoth Paddock, near Alice Springs (Greenslade, P.J.M.), Kunoth Paddock, near Alice Springs (Greenslade, P.J.M.), Manbulloo, SW Katherine (Greenslade, P.J.M.), Manbulloo, SW Katherine (Greenslade, P.J.M.), Manbulloo, SW Katherine (Greenslade, P.J.M.), Tanami (Greenslade, P.J.M.), Tanami (Greenslade, P.J.M.), Tanami Desert (Greenslade, P.J.M.). **Queensland**: ‘Gumbardo’ (Beutel, T.), ‘Merigol’ (Beutel, T.), ‘Paingo’ turnoff (Monteath & Cook), 13 km E by S Weipa (Zborowski, P. & Shattuck, S.O. [ANIC32-043343]), 13 km from S-bend on Plum Pudding Track (Lemann, C. [ANIC32-035480]), 13 mi WNW Capella (Dowse, J.E.), 16 mi ESE Gilbert River crossing, E Croydon (Dowse, J.), 16 mi NNW Miles (Dowse, J.E.), 2 mi N Greenvale Homestead, W Ingham (Dowse, J.E.), 3.2 km NW homestead on Plum Pudding Track, Cravens Peak Station (Lemann, C. [ANIC32-036868]), 30 km SSE Heathlands (Shattuck, S.O. [ANIC32-039870]), 35 mi SSW Nebo (Dowse, J.E.), 38 mi N Goondiwindi (Dowse, J.E.), 40 km E Cameron Corner (Greenslade, P.J.M.), 40 km E Cameron Corner (Greenslade, P.J.M.), 45 km E Cunnamulla (Greenslade, P.J.M.), 4 km NE Batavia Downs (Zborowski, P. & Calder, A. [ANIC32-042927]), 50 km E Cunnamulla (Greenslade, P.J.M.), 65 km E Birdsville (Forrest, J.), 6 mi W Blackwater (Dowse, J.E.), 75 km E Cunnamulla (Greenslade, P.J.M.), 9.5 km E Mareeba (Shattuck, S.O. [ANIC32-030606]), Archer River, Cape York (Wild, A.L. [ANIC32-030284]), Blackwood NP (Monteath & Cook), Cunnamulla (Greenslade, P.J.M.), Doolandella, Cloverdale St. (Burwell C. J., Wright, S. G.), Eneby, 1.6 km ESE (Monteith, Cook), Gregory Dev. Rd, Sardine Ck (Monteath & Cook), Hann River (Zborowski, P. & Horak, M. [ANIC32-031063]), Horn Island, Torres Strait (Heatwole, H. & Cameron, E.), Illaweena St. Drewvale (QM Party), Koondooloo, Hannaford Rd North, via Tara (House & Brown), Lolworth NP (Wright, S.), Lords Table, W base (Burwell, C. J.), Mt Isa (Weatherill, J.), Musselbrook Camp (Naumann, I.D. [ANIC32-006731]), Mutyi, Cooloola (Greenslade, P.J.M.), Mutyi, Cooloola (Greenslade, P.J.M.), N. Stradbroke Is. Enterprise (QM Party), Red Falls, 7 km WSW (Monteath, G. B.), Redlands Hilliards Ck, nr South St (RHC1) (Stanisic, J.), Sandringham (Morton, S.), Sandringham (Morton, S.), Sandringham (Morton, S.), Sandringham (Greenslade, P.J.M.), Sandringham (Greenslade, P.J.M.), Sandringham (Morton, S.), Sandringham (Morton, S.), St. George (Lowery, B.B.), St. George (Lowery, B.B.), Sunnybank (Wheeler, W.M.), Tindaree, Hannaford Rd South via Tara (House, A./Brown, S.). **South Australia**: 13 km S Quorn, Flinders Ranges (Greenslade, P.J.M.), 15 km NE Mt Bryan (Greenslade, P.J.M.), 15 km WSW Blinman, Flinders Ranges (Greenslade, P.J.M.), 16 mi SE Woolcalla (Greaves, T.), 1 km E Curdimurka (Feehan, J.E.), 1 km NW Elatina Hut, Flinders Ranges (Greenslade, P.J.M.), 1 km W Emu Camp, Victoria Desert (Greenslade, P.J.M.), 50 km S Coober Pedy (Davidson, D. & Morton, S.), 53 km E Vokes Hill, Victoria Desert (Greenslade, P.J.M.), 5 km W Auburn (Greenslade, P.J.M.), 9 mi NW Port Kenny (Greaves, T.), about 18 km SbyE Poochera (Taylor, R.W. & Bartell, R.J.), Adelaide (Womersley, H.W.), Alligator Gorge, Flinders Ranges (Greenslade, P.J.M.), Belair (Greenslade, P.J.M.), Belair (Greenslade, P.J.M.), Belair (Greenslade, P.J.M.), Belair (Greenslade, P.J.M.), Belair (Greenslade, P.J.M.), Belair (Greenslade, P.J.M.), Belair (Greenslade, P.J.M.), Belair (Greenslade, P.J.M.), Bindyi, Koonamore (Greenslade, P.J.M.), Blyth, 4 mi W Clare (Lowery, B.B.), Blyth, 4 mi W Clare (Lowery, B.B.), Blyth, N Adelaide (Lowery, B.B.), Blyth, N Adelaide (Lowery, B.B.), Calca (Lowery, B.B.), Cambrai (Greenslade, P.J.M.), Cambrai (Greenslade, P.J.M.), Cambrai (Greenslade, P.J.M.), Cambrai (Greenslade, P.J.M.), Cambrai (Greenslade, P.J.M.), Carrickalinga, Normanville (Greenslade, P.J.M.), Carrieton (Greenslade, P.J.M.), Elatina Hut, Oriparinna, Flinders Ranges (Greenslade, P.J.M.), Elatina Hut, Oriparinna, Flinders Ranges (Greenslade, P.J.M.), Eyre Hwy, 9.7 km NE Cootra (Heterick, B.E. [M325/M326]), Gawler Ranges (Greenslade, P.J.M.), Glen Osmond (Greenslade, P.J.M.), Moockra Tower (Greenslade, P.J.M.), Napperby, Flinders Ranges (Greenslade, P.J.M.), Napperby, Flinders Ranges (Greenslade, P.J.M.), Napperby, Flinders Ranges (Greenslade, P.J.M.), Napperby, Flinders Ranges (Greenslade, P.J.M.), Oraparinna, Flinders Ranges (Greenslade, P.J.M.), Oraparinna, Flinders Ranges (Greenslade, P.J.M. [ANIC32-900123]), Oraparinna, Flinders Ranges (Greenslade, P.J.M.), Oraparinna, Flinders Ranges (Greenslade, P.J.M.), Oraparinna, Flinders Ranges (Greenslade, P.J.M.), Oraparinna, Flinders Ranges (Greenslade, P.J.M.), Oraparinna, Flinders Ranges (Greenslade, P.J.M.), Oraparinna, Flinders Ranges (Greenslade, P.J.M.), Poochera (Taylor, R.W. & Bartell, R.J.), Port Adelaide (Lowery, B.B.), Port Adelaide (Lowery, B.B.), Port Augusta (Lowery, B.B.), Sandy Creek, Kangaroo Island (Greenslade, P.J.M.), Scrubby Gully, Sevenhill (Lowery, B.B.), Sevenhill (Lowery, B.B.), Sevenhill (Lowery, B.B.), Sevenhill (Lowery, B.B.), Sevenhill (Lowery, B.B.), Sevenhill (Lowery, B.B.), Sevenhill (Lowery, B.B. [ANIC32-900124]), Spalding Cove, Port Lincoln (Greenslade, P.J.M.), Streaky Bay (Lowery, B.B.), Streaky Bay (McAreavey, J.), Streaky Bay (Lowery, B.B.), Wudinna townsite (Heterick, B.E. [M330]). **Victoria**: 15 km WNW Yaapeet (Andersen, A.N.), 15 km WNW Yaapeet (Andersen, A.N.), 15 km WNW Yaapeet (Andersen, A.N.), Bendigo (McAreavey, J.), Eltham (Lowery, B.B.), Hopetoun (Andersen, A.N.), Lerderderg Gorge (Lowery, B.B.), Mount Feathertop Alps (Lowery, B.B.), Mt Ben Cairn (Greaves, T.), Rotamah Island, Gippsland Lakes (Andersen, A.N.), Wangarratta (Bruce, W.A.), Wyperfeld National Park (Taylor, R.W.). **Western Australia**: ‘The Granites’ Mt Magnet (Heterick, B.E. [M172]), 1 km S Capricorn RH (Heterick, B.E. [M278/M279/M280]), 1.5 km S Koolyanobbing (Heterick, B.E. [M24/M50]), 47 km E Fitzroy Crossing (Heterick, B.E. [M237]), 8 km N Bullfinch (Heterick, B.E. [M125/M126/M127/M128/M129]), Coomallo Downs (Heterick, B.E. [M06/M29/M30]), Edagee Rd (Heterick, B.E. [M75/M118]), Eyre Hwy, 20 km N Norseman (Heterick, B.E. [M336]), G. J. Rd, 108 km E Carnarvon (Heterick, B.E. [M306]), Mardathuna Rd turnoff (Heterick, B.E. [M55]), Mettler Lake Rd (Heterick, B.E. [M164]), Pardoo Stn turnoff (Heterick, B.E. [M268]), Rabbit Proof Fence Rd (Heterick, B.E. [M58/M59]), Sandstone Rd turnoff (Heterick, B.E. [M293]).

#### Diagnosis.

*Melophorus turneri* can be placed in the *M. biroi* species-group on the basis of characters of the clypeus, propodeum, mandible and palps. The species is also placed in the *M. fieldi* species-complex because of the appearance of the anteriorly placed clypeal psammophore, the compact propodeum, the presence of more than one preapical spine on the metatibia, at least in the major worker, long, even spindly legs and an unmodified mandible in the major worker. *Melophorus turneri* has a very variable morphology, the populations overall resemble those of up to a dozen other species, and this taxon is almost impossible to define in a simple species diagnosis. However, in all populations the appressed setae on the gaster of all subcastes are very small and inconspicuous when the gaster is moderately distended, such setae being separated from one another by at least their own length (unlike *M. inconspicuus*, *M. hirsutipes*, etc.). For differences between *M. turneri* and *M. eumorphus* and *M. vitreus*, which share this type of pilosity, see the Diagnosis under those species. These appressed setae are also inconspicuous on the mesosoma and never long and silvery (as in *M. lanuginosus*). The node of the minor worker is often squamiform, and most commonly the cuticle is shining or even glossy with vestigial or weak shagreenation. *Melophorus longipes* is very similar to ‘typical’ *M. turneri* but the metafemur of the minor worker of *M. turneri* is shorter and stouter (0.75 × length of mesosoma ≤). In profile, the minor worker propodeum generally has a weak to strong angle between the dorsal and declivitous surfaces, also differentiating this ant from *M. longipes*. The major worker is more difficult to distinguish from that of *M. longipes*, but the *turneri* mesonotum is generally moderately convex in profile and the metafemur uniformly pale brown ochre to yellowish, the tibia being of the same colour as the femur. *Melophorus perthensis* can also be confused with *M. turneri*: see the Diagnosis under that species for the distinguishing characters between the two ants.

The “*pillipes*” condition occurs within this species (*Melophorus pillipes* Santschi being reduced to a junior synonym of *M. turneri* in this work). *Melophorus turneri* with the “*pillipes*” condition can be distinguished from *M. ankylochaetes*, *M. incisus* and *M. hirsutipes* by having true appressed setae on the gaster, and from two other ants with the “*pillipes*” condition (*M. vitreus* and *M. lanuginosus*) by the characters mentioned under the Diagnosis for those two species. Finally, *M. turneri* (“*pillipes*”) has a smooth mesopleuron, whereas those individuals of *M. hirsutipes* (“*pillipes*”) which have appressed setae on the gaster have a sculptured mesopleuron.

#### Minor worker description.

**Head.** Head square; posterior margin of head planar or weakly convex; frons shining with superficial shagreenation or microreticulation only; pilosity of frons a mixture of a few well-spaced, erect setae interspersed with appressed setae only. Eye moderate (eye length 0.20–0.49 length of side of head capsule); in full-face view, eyes set at about midpoint of head capsule; in profile, eye set around midline of head capsule; roughly ovoid, eye narrowed posteriad. In full-face view, frontal carinae straight or weakly convex; frontal lobes straight in front of antennal insertion. Anteromedial clypeal margin broadly and evenly convex, or broadly convex with anteromedial dimple; clypeal psammophore set at or above midpoint of clypeus; palp formula 6,4. Five mandibular teeth in minor worker; mandibles triangular, weakly incurved; third mandibular tooth distinctly shorter than apical tooth and teeth numbers two and four; masticatory margin of mandibles approximately vertical or weakly oblique. **Mesosoma.** Integument of pronotum, mesonotum and mesopleuron shining and microreticulate, microreticulation reduced on humeri; anterior mesosoma in profile broadly convex; appearance of erect pronotal setae long (i.e., longest erect setae longer than length of eye) and unmodified, or short, (i.e., longest erect setae shorter than length of eye) and unmodified, or erect pronotal setae absent; in profile, metanotal groove shallow, broadly V or U-shaped; propodeum shining and smooth or with superficial and almost invisible microsculpture; propodeum smoothly rounded or with indistinct angle, or angulate, propodeal angle blunt; length ratio of propodeal dorsum to its declivity about 1:1; erect propodeal setae variable in number, may be absent; appressed propodeal setulae short, separated by more than own length and inconspicuous; propodeal spiracle situated on or beside declivitous face of propodeum, and shorter (length < 0.50 × height of propodeum). **Petiole.** In profile, petiolar node squamiform; in full-face view, shape of petiolar node uniformly rounded; node shining and smooth throughout. **Gaster.** Gaster shining with superficial microreticulation; pilosity of first gastral tergite consisting of well-spaced, erect and semi-erect setae interspersed with regularly placed appressed setae. **General characters.** Colour of foreparts usually shades of red or orange with brown gaster, occasionally uniformly brown or dark brown.

#### Major worker description.

**Head.** Head horizontally rectangular, broader than wide; posterior margin of head planar or weakly concave; cuticle of frons shining and smooth except for piliferous pits, or shining with superficial shagreenation or microreticulation only; pilosity of frons a mixture of a few well-spaced, erect setae interspersed with appressed setae only. Eye moderate (eye length 0.20–0.49 length of head capsule); in full-face view, eyes set above midpoint of head capsule; in profile, eye set anteriad of midline of head capsule; roughly ovoid, eye narrowed posteriad. In full-face view, frontal carinae concave; frontal lobes straight in front of antennal insertion. Anterior clypeal margin broadly convex with anteromedial dimple; clypeal psammophore set at or above midpoint of clypeus; palp formula 6,4. Five mandibular teeth in major worker; mandibles triangular, weakly incurved; third mandibular tooth distinctly shorter than apical tooth and teeth numbers two and 4; masticatory margin of mandibles approximately aligned vertically or weakly oblique. **Mesosoma.** Integument of pronotum, mesonotum and mesopleuron shining with indistinct microsculpture that is most pronounced on lower surfaces; anterior mesosoma in profile broadly convex; erect pronotal setae short, (i.e., shorter than length of eye) and unmodified; in profile, metanotal groove shallow, broadly V- or U-shaped; propodeum shining and shagreenate; propodeum smoothly rounded or with indistinct angle, or angulate, propodeal angle blunt; length ratio of propodeal dorsum to its declivity between 1:1 and 1:2; erect propodeal setae present and abundant (at least a dozen), or present and sparse to moderate (1-12); propodeal spiracle situated on or beside declivitous face of propodeum, and shorter (length less than 0.50 × height of propodeum). **Petiole.** In profile, petiolar node squamiform; in full-face view, shape of petiolar node generally rounded with median indentation, or tapered with blunt vertex; node shining and faintly shagreenate-microreticulate. **Gaster.** Gaster shining with superficial microreticulation; pilosity of first gastral tergite consisting of well-spaced, erect and semi-erect setae interspersed with regularly spaced appressed setae. **General characters.** Colour of foreparts variably orange, tan or brown, gaster brown to blackish.

#### Measurements.

Worker (n = 8): CI 91–118; EI 22–39; EL 0.23–0.34; HL 0.63–1.31; HW 0.58–1.54; ML 0.90–1.57; MTL 0.61–0.98; PpH 0.11–0.19; PpL 0.37–0.65; SI 73–141; SL 0.81–1.13.

#### Comments.

In many ways, this is the archetypal *Melophorus* and the one most likely to be seen by members of the public in temperate Australia, especially in rural areas. The ant has a more widespread distribution throughout mainland Australia than any other member of the genus, and can be found from the tip of Cape York, in QLD, to the southernmost parts of Victoria. There are, however, no Tasmanian records. The unexceptional appearance of the ant can lead it be confused with several other members of the *fieldi* complex, from which it can be distinguished by the combination of the appearance of the appressed setae on the first gastral tergite (separated by more than their length), the moderate eye size in most populations, its relatively short, thick metatibia and the usually glabrous or relatively glabrous mesosoma. Populations that display the ‘*pillipes*’ condition – the taxon *pillipes* is in fact here relegated to junior synonym status under *turneri*–are among the most spectacular manifestations of the variation seen in *turneri*; workers being hairy and shiny, with a brush of fine, straight, erect setae that branch from their tibiae in whorls at a right angle or near, and with a strongly truncate propodeum.

We acknowledge, however, that what we are here calling ‘*turneri*’ may prove to be a complex of several species. Apart from wide morphological variability, this possibility is given greater weight by the polyphyletic nature of the genetic evidence. Discontinuous clusters of *turneri* taxa appear within the clades that comprise the *fieldi* complex. Nonetheless, no morphological feature distinguishes any one separated cluster from another. Of the named taxa that are subsumed under ‘*turneri*’ here, the taxon that is perhaps the best candidate for separate species-level status is *M. turneri candidus*. However, M346 (*M. turneri candidus*) groups strongly with M353 (*turneri*) based on the COI gene. This form is most often encountered in cooler, temperate areas of Australia such as the Adelaide hills and parts of Victoria. The workers of this morphotype are small, compact *turneri* with a large eye and often but not always a convex mesonotum (reminiscent of *M. perthensis*, but the propodeum is not protuberant and is the typical one found in *M. turneri*). These ants also have a shining appearance and are generally convergent upon *Melophorus bruneus* in their morphology. Some minor workers of this form are also strongly morphologically convergent on *M. major* but the psammophore is placed well above the anterior clypeal margin. Others are more gracile. Some workers are glabrous, while others have a couple to several long, erect setae on the mesosoma and sometimes the antennal scape. Overall, among the more conspicuous morphs there are many others which seem to intergrade with other morphotypes of *M. turneri*. Several samples of large-eyed *M. turneri* workers have been sequenced, these coming from populations in the Pilbara which lack the bulbous mesonotum. The results do not point to uniqueness. Since no common diagnostic character or characters can be derived for *M. turneri candidus*, this species becomes a junior synonym in this monograph.

In contrast to *M. turneri candidus*, *Melophorus turneri aesopus* Forel and *Melophorus turneri* Forel itself appear to belong to the same lineage of large-bodied, dark *turneri*, and do not differ in any particulars. The former name therefore becomes a junior synonym in this work. *Melophorus pillipes* includes some of the most spectacularly beautiful of the small *Melophorus*. Apart from the strange condition that leads to conspicuous hairiness, *Melophorus pillipes* includes populations that exhibit a very glassy cuticle that is aesthetically pleasing for the researcher. These ants tend to have a high node and a straight mesonotum and long, fine, flexuous setae on the head and body and whorls of long, fine, straight setae on the legs and antennal scape. However, they cannot be discretely categorized since length and abundance of pilosity, height of node and degree of sculpture vary from the extreme to workers that have a few straight, fine, *pillipes*-type setae on the legs and antennae and perhaps the head and mesosoma. These latter ants also have some degree of fine cuticular sculpture. The very spectacular *M. pillipes* that are mostly found in eastern Australia with a few pockets of occurrence in the top end have not been sequenced. However, the apparent spectrum of worker phenotypes from very smooth, shining and hairy to the normal appearance of *M. turneri* with a few *pillipes*-type setae on the legs (but short, as in *M. microtriches* rather than long as in the spectacular morphotype) makes phenotypic plasticity a more parsimonious explanation of the phenomenon than genetic uniqueness to our mind. We therefore provisionally synonymize *M. pillipes* under *M. turneri* as an interesting variation on a theme. However, we acknowledge an investigation of the relationship between ‘*pillipes*’ type ants and conventional *Melophorus turneri* workers would be a worthwhile specialized project to explore in greater depth.

We here designate the ANIC syntype major worker of *M. turneri* as the lectotype to fix the name for this variable taxon, the remaining workers held in MHNG becoming paralectotypes.

This very common ant has been taken from a great variety of habitats but mallee scrub and sandy soils predominate in the label data. However, *M. turneri* has even been taken amid mangroves (Port Adelaide, South Australia)! In Western Australia the species is one of a small number of native ants regularly collected in paddocks. The ant is therefore likely to be an adaptable generalist.

**Figure 73.** *Melophorus turneri* Forel: ANIC ‘*turneriaesopus*’ major worker syntype (CAS0172012) frons (**a**), profile (**b**) and dorsum (**c**); MCZ ‘*turneriaesopus*’ major worker syntype (MCZC00023031) frons (**d**), profile (**e**) and dorsum (**f**); BMNH ‘*turnericandida*’ major worker syntype (BMNH Arnold Coll. BM 1934-354) frons (**g**), profile (**h**) and dorsum (**i**); BMNH ‘*turnericandida*’ minor worker syntype (BMNH: same card and labels as for major worker) frons (**j**), profile (**k**) and dorsum (**l**); MCZ ‘*pillipes*’ major worker syntype (MCZ: Cotype 23032) frons (**m**), profile (**n**) and dorsum (**o**); MCZ ‘*pillipes*’ minor worker syntype (MCZ: Cotype 23032) frons (**p**), profile (**q**) and dorsum (**r**); ‘*turneri*’ non–type major worker (ANIC32-059628) frons (**s**), profile (**t**) and dorsum (**u**); ‘*turneri*’ non–type minor worker (ANIC32-900192) frons (**v**), profile (**w**) and dorsum (**x**); distribution map for the species for the species (**y**). Low resolution scale bars: 0.5 mm (**w, x**); 0.2 mm (**v**).

### Melophorus vitreus

Taxon classificationAnimaliaHymenopteraFormicidae

Heterick, Castalanelli & Shattuck sp. n.

http://zoobank.org/E99F05E9-403D-4459-9CA4-96ED64C5113D

#### Types.

Holotype minor worker (top ant) from Black Swan Mine 30°28'S, 121°43'E, Western Australia, 11 December 2003-5 January 2004, P. Langlands/J. Osborne, site data C4,8 [JDM32-004585] (WAM). Paratypes: 3 minor workers on same pin and with same details as holotype (WAM); 4 minor workers from Kunoth Paddock near Alice Springs, Northern Territory, 3-6 February 1975, P.J.M. Greenslade, Trap VM, 20) [ANIC32-900122] (ANIC); minor worker from 15 km SE of Alice Springs 23.51S, 133.58E, Northern Territory, 9 October 1991, D. Davidson/S. Morton, 120C (BMNH); 2 minor workers from 17 km S of Rabbit Flat, Northern Territory, 19 May 1986, P.J.M. Greenslade, (8) B, 11) (MCZ); minor worker from Mt Whaleback, Newman, Western Australia, July 1984, K. J. Walker [JDM32-004586] (QM).

#### Other material examined.

**Queensland**: ‘Gumbardo’ (Beutel, T.), ‘Merigol’ (Beutel, T.). **Western Australia**: 1 km W Canna (Heterick, B.E. [M322]).

#### Diagnosis.

*Melophorus vitreus* can be placed in the *M. biroi* species-group on the basis of characters of the clypeus, propodeum, mandible and palps. The species is also placed in the *M. fieldi* species-complex because of the appearance of the anteriorly placed clypeal psammophore, the compact propodeum, the presence of more than one preapical spine on the metatibia, at least in the major worker, the long, even spindly legs and the unmodified mandible in the major worker. In profile, the minor worker of *M. vitreus* is gracile, with a short, thick petiolar node terminating in a smoothly rounded vertex, the clypeus is rounded and somewhat protuberant the eye very large (eye length ≥ 0.50 × length of side of head capsule) and the cuticle is extremely glossy with the mesopleuron completely smooth or with only a very superficial microreticulate pattern. The combination of the very large eye, the protuberant clypeus, the smooth mesopleuron and the general glossiness will serve to identify the ant. Most specimens exhibit the “*pillipes*’ condition. The major worker is unknown.

#### Minor worker description.

**Head.** Head square; posterior margin of head weakly convex; frons shining and smooth except for piliferous pits; pilosity of frons a mixture of a few well-spaced, erect setae interspersed with appressed setae only. Eye large (eye length ≥ 0.50 × length of side of head capsule); in full-face view, eye set at about midpoint of head capsule; in profile, eye set anteriad of head capsule; eyes elliptical or slightly reniform. In full-face view, frontal carinae distinctly concave; frontal lobes straight in front of antennal insertion. Anteromedial clypeal margin broadly and evenly convex; clypeal psammophore set at or above midpoint of clypeus; palp formula 6,4. Five mandibular teeth in minor worker; mandibles triangular, weakly incurved; third mandibular tooth distinctly shorter than apical tooth and teeth numbers two and four; masticatory margin of mandibles approximately vertical or weakly oblique. **Mesosoma.** Integument of pronotum, mesonotum and mesopleuron shining and mainly smooth, vestigial shagreenation most noticeable on humeri and mesopleuron; anterior mesosoma in profile weakly elevated anteriad, thereafter gently sinuate, pronotum and mesonotum on same plane; appearance of erect pronotal setae short, (i.e., longest erect setae shorter than length of eye) and unmodified, or erect pronotal setae absent; in profile, metanotal groove shallow, broadly V or U-shaped; propodeum shining and smooth or with superficial and almost invisible microsculpture; propodeum always smoothly rounded; propodeal dorsum and declivity confluent; erect propodeal setae variable in number, may be absent; appressed propodeal setulae short, separated by more than own length and inconspicuous; propodeal spiracle situated on or beside declivitous face of propodeum, and shorter (length < 0.50 × height of propodeum). **Petiole.** In profile, petiolar node subcuboidal, vertex bluntly rounded; in full-face view, shape of petiolar node uniformly rounded; node shining and smooth throughout. **Gaster.** Gaster smooth and glossy; pilosity of first gastral tergite consisting of well-spaced, erect and semi-erect setae interspersed with regularly placed appressed setae. **General characters.** Colour of foreparts orange tan to reddish brown, gaster brown to blackish-brown.

#### Measurements.

Worker (n = 6): CI 99–106; EI 36–41; EL 0.23–0.25; HL 0.56–0.66; HW 0.56–0.70; ML 0.81–0.96; MTL 0.45–0.52; PpH 0.08–0.107; PpL 0.30–0.36; SI 96–105; SL 0.59–0.67.

#### Comments.

This small ant resembles a smooth, glossy, gracile *M. turneri*, but can be distinguished from that species by features of the clypeus, the eye and the node and by its glossy, gracile mesosoma. Most specimens seen exhibit the ‘*pillipes*’ condition, but a few workers lack these fine setae. The species is definitely known from NT and WA but, based on its habitat preference, it may also be looked for in SA. Sequencing data places it near *M. incisus* and *M. ankylochaetes*. Limited ecological data indicate it has been collected in mallee and also on lateritic sandplain with an overlay of proteaceous heathland and small trees.

#### Etymology.

Latin *vitreus* (‘of glass’ or ‘resembling glass’); adjective in the nominative singular.

**Figure 74.**
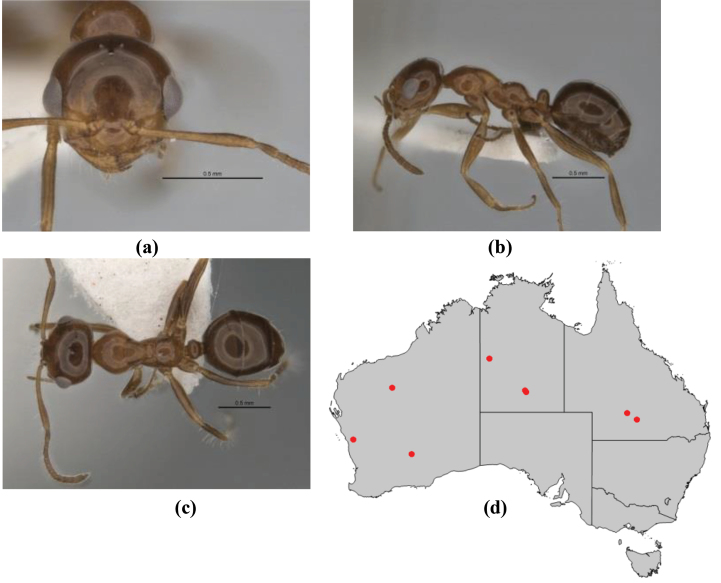
*Melophorus vitreus* sp. n.: holotype minor worker (JDM32-004585) frons (**a**), profile (**b**) and dorsum (**c**); distribution map for the species (**d**). Low resolution scale bars: 0.5 mm (**a–c**).

### *Melophorus oblongiceps* complex

The *Melophorus oblongiceps* complex is monotypic with one constituent species. All subcastes have a very similar head capsule and have short maxillary palps. All specimens have been collected in the vicinity of Lake Eyre, which suggests the species may be closely adapted to its lacustrine environment.

### Melophorus oblongiceps

Taxon classificationAnimaliaHymenopteraFormicidae

Heterick, Castalanelli & Shattuck sp. n.

http://zoobank.org/2ABCECE5-E372-4A5A-B595-CA9A05FD5EDC

#### Types.

Holotype minor worker (bottom ant) from c. 7 km SE of William Creek 28.57S× 136.24E, South Australia, 22 September 1972, J. E. Feehan, ANIC Ants Vial 16.64 [ANIC32-900057] (ANIC). Paratypes: major worker and media worker on same pin and with same details as holotype (ANIC); 2 major workers and a minor worker from Sulphur Peninsula, Lake Eyre North, South Australia, 21 August 1967, G.P. Cross & F.J. Mitchell, nests in damp margin of Lake Eyre, S.A. Mus. specs. [ANIC32-900056] (BMNH); major worker, media worker and minor worker from Sulphur Peninsula on separate pin with same details as the immediately preceding, but without ANIC number (SAM)minor worker and 4 major workers from Curdimurka, South Australia, 1 April 1972, B.B. Lowery, in soft, damp mud margin of Lake Eyre (MCZ).

#### Other material examined.

**South Australia**: Curdimurka (Lowery, B.B.), Lake Harris (Hudson, P. [M340]).

#### Diagnosis.

*Melophorus oblongiceps* can be placed in the *M. biroi* species-group on the basis of the characters of the clypeus, propodeum, mandible and palps. However, it occupies a separate, monotypic species-complex. *Melophorus oblongiceps* can be determined by the following characters: major and minor workers with a combination of long mandibles (i.e., the apical tooth of the retracted mandible reaches to at least the tentorial pit on the opposite side of the head capsule), similar appearance of the head capsule in major and minor workers and short maxillary palps (segments four to six combined barely longer than segment three), in profile, the entire palp reaching only to the middle of the venter of the head capsule in the minor worker when the head is moderately inclined. The head of the major worker is without a short, massive, elbowed mandible directed posteriad. All workers possess five mandibular teeth; the median sector of clypeus is uniformly convex and moderately protuberant with a small, blunt angle at the midpoint of the anterior clypeal margin.

#### Minor worker description.

**Head.** Head square; posterior margin of head planar or weakly convex; frons shining with superficial shagreenation or microreticulation only; pilosity of frons a mixture of short, erect and semi-erect setae interspersed with shorter decumbent setae and well-spaced, short, appressed setae. Eye moderate (eye length 0.20–0.49 length of side of head capsule); in full-face view, eyes set above midpoint of head capsule; in profile, eye set anteriad of midline of head capsule; eyes elliptical or slightly reniform. In full-face view, frontal carinae distinctly concave; frontal lobes straight in front of antennal insertion. Anteromedial clypeal margin broadly and evenly convex; clypeal psammophore set at or just above anterior clypeal margin; palp formula 6,4. Five mandibular teeth in minor worker; mandibles triangular, weakly incurved; third mandibular tooth distinctly shorter than apical tooth and teeth numbers two and four; masticatory margin of mandibles approximately vertical or weakly oblique. **Mesosoma**. Integument of pronotum, mesonotum and mesopleuron moderately shining and shagreenate throughout; anterior mesosoma in profile broadly convex; appearance of erect pronotal setae short, (i.e., longest erect setae shorter than length of eye) and unmodified, or erect pronotal setae absent; in profile, metanotal groove shallow, broadly V or U-shaped; propodeum shining and microreticulate; propodeum always smoothly rounded; propodeal dorsum and declivity confluent; erect propodeal setae always absent; appressed propodeal setulae short, separated by more than own length and inconspicuous; propodeal spiracle situated on or beside declivitous face of propodeum, and shorter (length < 0.50 × height of propodeum). **Petiole.** In profile, petiolar node squamiform; in full-face view, shape of petiolar node uniformly rounded; node shining and smooth with vestigial sculpture. **Gaster.** Gaster shining with superficial microreticulation; pilosity of first gastral tergite consisting of a mixture of curved, erect and semi-erect setae and decumbent and appressed setae that form a variable pubescence. **General characters.** Colour russet.

#### Major worker description.

**Head.** Head horizontally rectangular, broader than wide; posterior margin of head weakly concave; cuticle of frons shining with superficial shagreenation or microreticulation only; pilosity of frons a mixture of well-spaced, distinctly longer erect and semi-erect setae interspersed with shorter decumbent setae. Eye moderate (eye length 0.20–0.49 length of head capsule); in full-face view, eyes set above midpoint of head capsule; in profile, eye set anteriad of midline of head capsule; eyes elliptical. In full-face view, frontal carinae distinctly concave; frontal lobes straight in front of antennal insertion. Anterior clypeal margin broadly and evenly convex; clypeal psammophore set at or just above anterior clypeal margin; palp formula 6,4. Five mandibular teeth in major worker; mandibles triangular, weakly incurved; third mandibular tooth distinctly shorter than apical tooth and teeth numbers two and four; masticatory margin of mandibles approximately aligned vertically or weakly oblique. **Mesosoma.** Integument of pronotum, mesonotum and mesopleuron shining with very superficial microreticulation, entire lower mesopleuron distinctly shagreenate; anterior mesosoma in profile broadly convex; erect pronotal setae short, (i.e., shorter than length of eye) and unmodified; in profile, metanotal groove shallow, broadly V- or U-shaped; propodeum shining and microreticulate; propodeum smoothly rounded or with indistinct angle; propodeal dorsum and declivity confluent; erect propodeal setae present and abundant (at least a dozen); appressed propodeal setae long, each reaching setae behind and in front, but not forming pubescence; propodeal spiracle situated on or beside declivitous face of propodeum, and shorter (length less than 0.50 × height of propodeum). **Petiole.** In profile, petiolar node squamiform; in full-face view, shape of petiolar node tapered with blunt vertex, or tapered with sharp vertex; node shining and distinctly microreticulate. **Gaster.** Gaster shining with superficial microreticulation; pilosity of first gastral tergite consisting of a mixture of curved, erect and semi- erect setae and decumbent setae that form a variable pubescence. **General characters.** Colour russet.

#### Measurements.

Worker (n = 2): CI 104–114; EI 18–26; EL 0.23–0.35; HL 0.86–1.74; HW 0.89–1.98; ML 1.26–2.22; MTL 1.01–1.48; PpH 0.13–0.21; PpL 0.49–0.86; SI 71–121; SL 1.08–1.42.

#### Comments.

*Melophorus oblongiceps* appears to be restricted to the damp terrestrial environment around Lake Eyre, SA. This species resembles some members of the *fieldi* and *wheeleri* complexes, but can be distinguished by the similar appearance of the different subcastes of workers, the short palps and the non-massive five-toothed mandibles. The habits of the ant have not been documented but it may have some specializations, since the only ecological notes mentioned on labels indicate it nests and forages in the damp margins at the edge of Lake Eyre. Sequencing of one specimen has been largely unsuccessful, but sufficient to indicate the species may belong somewhere in the vicinity of the *M. wheeleri* complex.

#### Etymology.

Latin *oblongus* (‘longer than broad’) plus -*ceps* (‘-headed’ [from *caput*]); adjective in the nominative singular.

**Figure 75.**
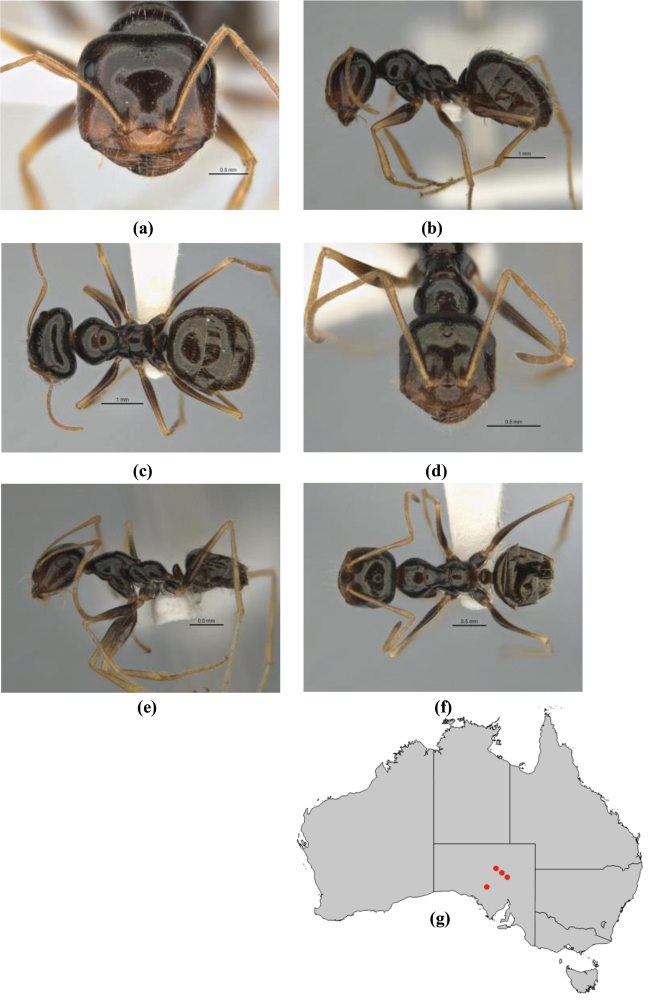
*Melophorus oblongiceps* sp. n.: major worker paratype (ANIC32-900057–top ant) frons (**a**), profile (**b**) and dorsum (**c**); minor worker holotype (ANIC32-900057–bottom ant) frons (**d**), profile (**e**) and dorsum (**f**); distribution map for the species (**g**). Low resolution scale bars: 1 mm (**b, c**); 0.5 mm (**a, d–f**).

### *Melophorus wheeleri* complex

Major workers of this complex are often spectacular ants with very broad heads and massive, crushing mandibles for milling seeds. Minor workers are similar to those of the *M. fieldi* complex, but the usually finely striate mandibles often have more than five teeth. Within the broader *wheeleri* complex there are recognisable smaller clades usually involving a pair of closely related species that agree in both their morphology and their molecular characteristics; e.g., *M. laticeps* and *M. pelorocephalus*, and *M. parvimolaris* and *M. xouthos*. Possibly differentiation of these sibling species arose through fragmentation of their physical environment within recent geological time.

### Melophorus brevipalpus

Taxon classificationAnimaliaHymenopteraFormicidae

Heterick, Castalanelli & Shattuck sp. n.

http://zoobank.org/10E61CC6-FD84-451A-AD03-66F50D51AF8B

#### Types.

Holotype minor worker (bottom ant) from Emu Camp, Victoria Desert, South Australia, 5 October 1976, P.J.M. Greenslade, (6) [ANIC32-066590] (ANIC), Paratypes: major worker on same pin and with same details as holotype (ANIC); minor and major worker from 50 km S of Coober Pedy 29.27S, 134.51E, South Australia, 2 October 1981, D. Davidson/S. Morton, 67a (ANIC); media and minor worker from Observatory Hill, Victoria Desert, South Australia, 7 October 1976, P.J.M. Greenslade, (5) (BMNH); 2 media workers from 10 km E of Mt Ive HS, Gawler Ranges, South Australia, 21-22 October 1980, P.J.M. Greenslade, 18) (MCZ); 2 major workers and a media worker from 10 km E of Mt Ive HS, Gawler Ranges, South Australia, 21–22 October 1980, P.J.M. Greenslade, B Se (SAM).

#### Other material examined.

**South Australia**: 10 km E Mt Ive Homestead, Gawler Ranges (Greenslade, P.J.M.), 85 km W Mabel Creek (Greenslade, P.J.M.), 85 km W Mabel Creek (Greenslade, P.J.M.), Koonamore, Milang Conservation Park (Greenslade, P.J.M.).

#### Diagnosis.

*Melophorus brevipalpus* can be placed in the *M. biroi* species-group on the basis of characters of the clypeus, propodeum, mandible and palps. The species is also placed in the *M. wheeleri* species-complex because it agrees with the following apomorphies possessed by the complex: the minor worker often has more than five teeth, the largest major worker has a short, massive, elbowed mandible directed posteriad; in profile, the maxillary palps are short in the major and generally short in minor workers (in the minor worker, usually only attaining the neck sclerite at their maximum extent when the head is moderately inclined) and, in full-face view, the anterior margin of the clypeus in the large major worker is usually planar or weakly concave (variable in other subcastes but planar or narrowly protuberant anterior clypeal margins predominate). The reduced palps in *M. brevipalpus* (PF of 4,3; 3,3 and possibly 2,3 [undissected major worker]) only parallel the reduced palps in the unrelated and morphologically very different *M. potteri* species-group. These palps are extremely short and when they are directed posteriad do not reach the hypostomal border. This feature alone is sufficient to identify the species within the *M. wheeleri* complex.

#### Minor worker description.

**Head.** Head square; posterior margin of head planar or weakly convex; frons shining with superficial shagreenation or microreticulation only; frons consisting exclusively or almost exclusively of well-spaced, appressed setae only (small, erect setae, if present, usually confined to ocular triangle or posterior margin of head). Eye moderate (eye length 0.20–0.49 length of side of head capsule); in full-face view, eyes set above midpoint of head capsule; in profile, eye set anteriad of midline of head capsule; eyes elliptical or slightly reniform. In full-face view, frontal carinae concave; frontal lobes straight in front of antennal insertion. Anteromedial clypeal margin straight; clypeal psammophore set at or above midpoint of clypeus; palp formula variably reduced (4,3 or 3,3). Five to six mandibular teeth in minor worker; mandibles triangular, weakly incurved; third mandibular tooth distinctly shorter than apical tooth, but equivalent in length to remaining teeth; masticatory margin of mandibles approximately vertical or weakly oblique. **Mesosoma.** Integument of pronotum, mesonotum and mesopleuron shining and microreticulate, microreticulation reduced on humeri; anterior mesosoma in profile broadly convex; erect pronotal setae absent; in profile, metanotal groove shallow, broadly V or U-shaped; propodeum shining and microreticulate; propodeum angulate, propodeal angle blunt; length ratio of propodeal dorsum to its declivity about 1:1; erect propodeal setae always absent; appressed propodeal setulae short, separated by more than own length and inconspicuous; propodeal spiracle situated on or beside declivitous face of propodeum, and shorter (length < 0.50 × height of propodeum). **Petiole.** In profile, petiolar node squamiform; in full-face view, shape of petiolar node uniformly rounded; node shining and smooth with vestigial sculpture. **Gaster.** Gaster shining, shagreenate (‘LP record’ appearance); pilosity of first gastral tergite consisting of well-spaced short, inconspicuous, appressed setae, erect setae (present in at least some workers) confined to margin of sclerite. **General characters.** Colour of foreparts orange tan to brown, gaster blackish-brown to black.

#### Major worker description.

**Head.** Head horizontally rectangular, broader than wide; posterior margin of head planar or weakly concave; cuticle of frons shining and smooth except for piliferous pits; frons consisting exclusively or almost exclusively of well-spaced, appressed setae only (small, erect setae, if present, usually confined to ocular triangle or posterior margin of head). Eye small (eye length less than 0.2 × length of head capsule); in full-face view, eyes set above midpoint of head capsule; in profile, eye set anteriad of midline of head capsule; eyes elliptical. In full-face view, frontal carinae straight, divergent posteriad; frontal lobes curved inward in front of antennal insertion, or curved toward antennal insertion. Anterior clypeal margin broadly emarginate; clypeal psammophore set at or above midpoint of clypeus; palp formula variably reduced (4,3 or 3,3 or even 2,3).Five or six mandibular teeth in major worker; mandibles strongly incurved, apical sector weakly carinate or incompletely carinate; third mandibular tooth distinctly shorter than apical tooth, but equivalent in length to remaining teeth; masticatory margin of mandibles approximately aligned vertically or weakly oblique. **Mesosoma.** Integument of pronotum, mesonotum and mesopleuron shining with very superficial microreticulation, entire lower mesopleuron distinctly shagreenate; anterior mesosoma in profile broadly convex; erect pronotal setae short, (i.e., shorter than length of eye) and unmodified, or erect pronotal setae absent; in profile, metanotal groove shallow, indicated mainly by an angle and metathoracic spiracles; propodeum shining and microreticulate; propodeum angulate, propodeal angle blunt; length ratio of propodeal dorsum to its declivity between 1:1 and 1:2; erect propodeal setae variable in number, may be absent; appressed propodeal setae short, separated by more than own length and inconspicuous; propodeal spiracle situated on or beside declivitous face of propodeum, and shorter (length less than 0.50 × height of propodeum). **Petiole.** In profile, petiolar node squamiform; in full-face view, shape of petiolar node tapered with blunt vertex, or tapered with squared-off vertex; node shining and smooth with vestigial microreticulation anteriad. **Gaster.** Gaster shining, shagreenate (‘LP record’ appearance); pilosity of first gastral tergite consisting of well-spaced, erect and semi-erect setae interspersed with regularly spaced appressed setae. **General characters.** Colour of foreparts orange tan (head of deeper hue), gaster dark brown.

#### Measurements.

Worker (n = 6): CI 112–118; EI 20–29; EL 0.20–0.26; HL 0.61–1.12; HW 0.68–1.31; ML 0.85–1.29; MTL 0.53–0.83; PpH 0.11–0.13; PpL 0.35–0.51; SI 68–92; SL 0.63–0.90.

#### Comments.

Because of its very short palps, which have an abbreviated number of segments, and its morphological resemblance to *M. wheeleri*, *Melophorus brevipalpus* cannot be mistaken for any other *Melophorus*. While a reduced PF is also found in several other species, including two in the *M. potteri* species-group, these latter taxa have a distinctive *habitus*. *Melophorus brevipalpus* has thus far only been recorded at a few localities in SA. No specimens were available for sequencing. One specimen was collected in a pitfall trap, but there are no other data. Nonetheless, given its apparent affinities with the *M. wheeleri* complex, we tentatively associate this species with a granivorous lifestyle.

#### Etymology.

Latin *brevis* (‘short’) plus *palpus* (‘stroking’/‘caress’; applied to the palps of an arthropod); noun in the nominative singular standing in apposition to the generic name.

**Figure 76.**
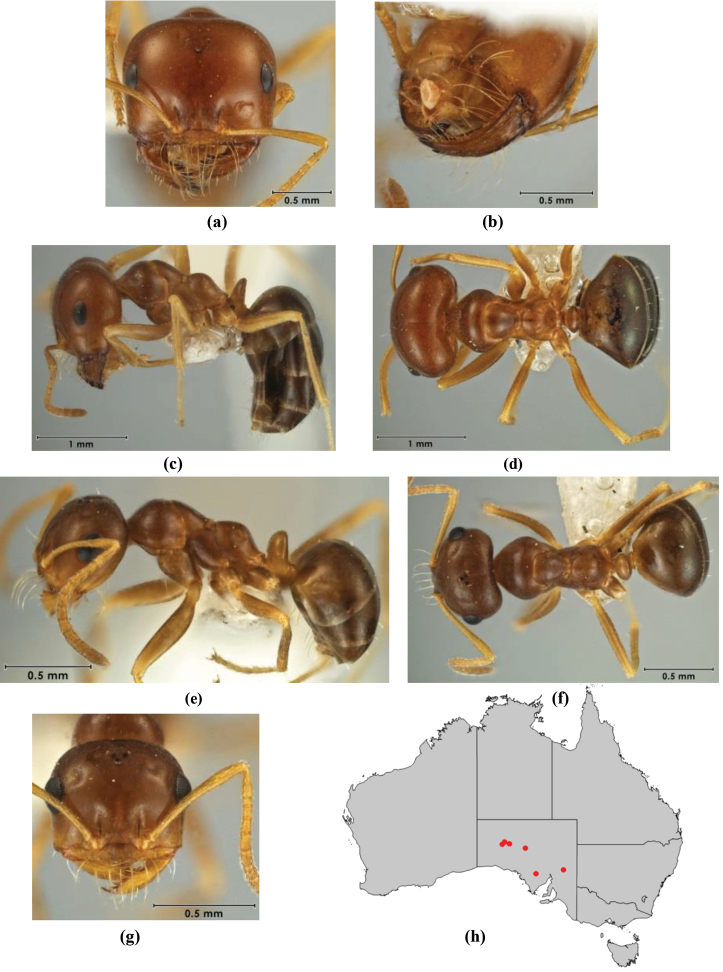
*Melophorus brevipalpus* sp. n.: major worker paratype (ANIC32-066590–top ant) frons (**a**), underside of head showing palps (**b**); profile (**c**) and dorsum (**d**); minor worker holotype (ANIC32-066590–bottom ant) frons (**e**), profile (**f**) and dorsum (**g**); distribution map for the species (**h**).

### Melophorus caeruleoviolaceus

Taxon classificationAnimaliaHymenopteraFormicidae

Heterick, Castalanelli & Shattuck sp. n.

http://zoobank.org/A819AB8E-CAB1-4DAA-8A58-54F700A0715D

#### Types.

Holotype minor worker (middle ant) from 53 km E of Vokes Hill, Victoria Desert, South Australia, 9 October 1976, P.J.M. Greenslade, (11) [ANIC32-900183] (ANIC). Paratypes: media worker and minor worker on same pin and with same details as holotype (ANIC); 4 minor workers from Emily Gap, Northern territory, 18 May 1978, Zhakharov, nest N65 [ANIC32-0133892] (BMNH); 2 major workers from 16 km W of Mt Aloysius 26.015S, 128.26'E, Western Australia, 16 November 1977, J. E. Feehan, associated collector T. A. Weir [ANIC32-900182] (MCZ); 3 minor workers from Wilgena Hill E. Tarcoola, South Australia, 2 October 1976, P.J.M. Greenslade, (3)a. (SAM); minor worker from 146.8 km SSE of Newman 24°35'21"S, 120°15'40"E, Western Australia, April 1997, S. van Leeuwen & R. N. Bromilow, Perm. Invert. pitfall trap B1. Mulga woodland [JDM32-004943] (WAM).

#### Other material examined.

**New South Wales**: Acacia Vale, near Broken Hill (Greenslade, P.J.M. [ANIC32-900052]). **Northern Territory**: Emily Gap (Zakharov), **Queensland**: Ernest Henry Mine (Sanders, M.). **South Australia**: 80 km W Emu, Victoria Desert (Greenslade, P.J.M.), 85 km W Mabel Creek (Greenslade, P.J.M.), 8 mi E Wilgena (Greaves, T. [ANIC32-900051]), Mt Davies turnoff, Victoria Desert (Greenslade, P.J.M.), **Western Australia**: Pannawonica Hill (Bokhari, F. [M152]).

#### Diagnosis.

*Melophorus caeruleoviolaceus* can be placed in the *M. biroi* species-group on the basis of characters of the clypeus, propodeum, mandible and palps. The species is also placed in the *M. wheeleri* species-complex because it agrees with the following apomorphies possessed by the complex: the minor worker often has more than five teeth, the largest major worker has a short, massive, elbowed mandible directed posteriad; in profile, the maxillary palps are short in the major and generally short in minor workers (in the minor worker, usually only attaining the neck sclerite at their maximum extent when the head is moderately inclined) and, in full-face view, the anterior margin of the clypeus in the large major worker is usually planar or weakly concave (variable in other subcastes but planar or narrowly protuberant anterior clypeal margins predominate). Within its complex, *M. caeruleoviolaceus* is characterised as a strongly bicoloured, relatively smooth species with black, brown or variegated brown head, orange mesosoma and black gaster, the gaster and often the head (in darker morphs) with distinct bluish to violet iridescence. This colour pattern is found in all subcastes but the colour pattern and bluish or violet iridescence is not shared by any other member of the *M. wheeleri* species-complex.

#### Minor worker description.

**Head.** Head square; posterior margin of head weakly convex; frons shining with superficial shagreenation or microreticulation only; pilosity of frons a mixture of a few well-spaced, erect setae interspersed with appressed setae only. Eye moderate (eye length 0.20–0.49 length of side of head capsule); in full-face view, eyes set above midpoint of head capsule; in profile, eye set anteriad of midline of head capsule; eyes elliptical or slightly reniform. In full-face view, frontal carinae straight, divergent posteriad; frontal lobes straight in front of antennal insertion. Anteromedial clypeal margin broadly and evenly convex; clypeal psammophore set at or above midpoint of clypeus; palp formula 6,4. Five to six mandibular teeth in minor worker; mandibles triangular, weakly incurved; third mandibular tooth distinctly shorter than apical tooth and teeth numbers two and four; masticatory margin of mandibles approximately vertical or weakly oblique. **Mesosoma.** Integument of pronotum, mesonotum and mesopleuron moderately shining and shagreenate throughout; anterior mesosoma in profile broadly convex; appearance of erect pronotal setae short, (i.e., longest erect setae shorter than length of eye) and unmodified; in profile, metanotal groove shallow, broadly V or U-shaped; propodeum shining and finely striolate and microreticulate; propodeum smoothly rounded or with indistinct angle, or angulate, propodeal angle blunt; length ratio of propodeal dorsum to its declivity about 4:3; erect propodeal setae present and abundant (greater than 12); appressed propodeal setulae short, separated by more than own length and inconspicuous; propodeal spiracle situated on or beside declivitous face of propodeum, and shorter (length < 0.50 × height of propodeum). **Petiole.** In profile, petiolar node, squamiform or subcuboidal, vertex bluntly rounded; in full-face view, shape of petiolar node uniformly rounded; node shining and smooth with vestigial sculpture. **Gaster.** Gaster shining, shagreenate (‘LP record’ appearance); pilosity of first gastral tergite consisting of a mixture of curved, erect and semi-erect setae and decumbent and appressed setae that form a variable pubescence. **General characters.** Colour of most of head, and coxae blackish-brown, mesosoma orange tan, gaster black; gaster (and often, head) with violet iridescence.

#### Major worker description.

**Head.** Head horizontally rectangular, broader than wide; posterior margin of head planar or weakly concave; cuticle of frons matt or with weak sheen, indistinctly shagreenate; pilosity of frons a mixture of many short, erect, bristly setae interspersed with regularly spaced appressed setae. Eye moderate (eye length 0.20–0.49 length of head capsule); in full-face view, eyes set above midpoint of head capsule; in profile, eye set anteriad of midline of head capsule; roughly ovoid, eye narrowed posteriad. In full-face view, frontal carinae straight, divergent posteriad; frontal lobes curved inward in front of antennal insertion. Anterior clypeal margin straight; clypeal psammophore set at or above midpoint of clypeus; palp formula 6,4. Four distinct mandibular teeth in major worker; mandibles weakly incurved; third mandibular tooth distinctly shorter than apical tooth and teeth numbers two and four; masticatory margin of mandibles approximately aligned vertically or weakly oblique. **Mesosoma.** Integument of pronotum, mesonotum and mesopleuron moderately shining and shagreenate throughout; anterior mesosoma in profile broadly convex; erect pronotal setae short, (i.e., shorter than length of eye) and unmodified; in profile, metanotal groove shallow, broadly V- or U-shaped; propodeum shining, with multiple hair like striolae; propodeum angulate, propodeal angle blunt; length ratio of propodeal dorsum to its declivity between 1:1 and 1:2; erect propodeal setae present and abundant (at least a dozen); appressed propodeal setae short, separated by more than own length and inconspicuous; propodeal spiracle situated at least twice its width from the declivitous face of propodeum, and shorter (length less than 0.50 × height of propodeum). **Petiole.** In profile, petiolar node narrowly conical, vertex blunt; in full-face view, shape of petiolar node uniformly rounded; node shining and faintly shagreenate-microreticulate. **Gaster.** Gaster weakly shining with indistinct shagreenation; pilosity of first gastral tergite consisting of well-spaced, short, thick, erect setae interspersed with minute, appressed setae. **General characters.** Colour of head, mesosoma and coxae variegated blackish-brown and orange tan, gaster black; gaster (and, often, head) with violet iridescence.

#### Measurements.

Worker (n = 6): CI 108–125; EI 21–31; EL 0.22–0.30; HL 0.68–1.15; HW 0.73–1.44; ML 0.97–1.54; MTL 0.59–0.86; PpH 0.11–0.18; PpL 0.40–0.63; SI 66–97; SL 0.71–0.95.

#### Comments.

Although the overall appearance of this species is typical of many of the *M. biroi* species-group, the violet iridescence on the gaster, and, in some instances, bluish iridescence on the head and body of the ant, make even old museum specimens strikingly attractive and serve to distinguish this ant from other *Melophorus*. The taxon is morphologically similar to *M. setosus*, and the minor worker shares the same protuberant clypeus, square vertex and porrect, rounded node. *Melophorus setosus*, however, is visibly covered in short, markedly modified erect setae, while the setae in *M. caeruleoviolaceus* are only slightly spatulate at their ends. Moreover, the major worker of *M. setosus* has the characteristic mandible of the *M. fieldi* complex, unlike the crushing *wheeleri*-type mandible found in major workers of *M. caeruleoviolaceus*. Sequencing has been carried out on one worker collected in hummock grassland near Pannawonica, WA. On a five-gene tree the WA specimen is placed near to *M. parvimolaris*, but is sister to *M. chauliodon* on a three-gene tree, and this is likely to be the more accurate placement. Although the species is found in remote desert and semi-arid areas in (at the least) NSW, NT, SA and WA, ecological data are wanting.

#### Etymology.

Latin *caeruleus* (‘sky-blue’) plus *violaceus* (‘violet’); referring to the attractively bicoloured appearance of this species; adjective in the nominative singular.

**Figure 77.**
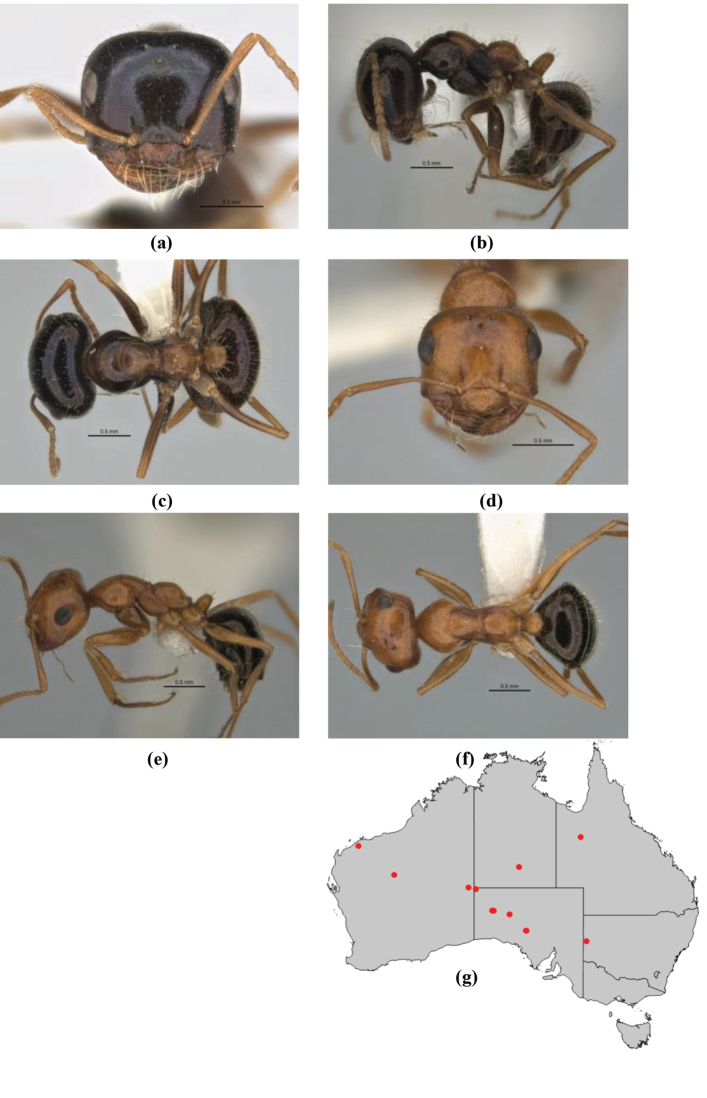
*Melophorus caeruleoviolaceus* sp. n.: major worker paratype (ANIC32-900182–bottom ant) frons (**a**), profile (**b**) and dorsum (**c**); minor worker holotype (ANIC32-900183–middle ant) frons (**d**), profile (**e**) and dorsum (**f**); distribution map for the species (**g**). Low resolution scale bars: 0.5 mm (**a–f**).

### Melophorus cerasinoniger

Taxon classificationAnimaliaHymenopteraFormicidae

Heterick, Castalanelli & Shattuck sp. n.

http://zoobank.org/D6B613D6-90E6-49EA-98AB-EED71BF29911

#### Types.

Holotype minor worker (fifth ant from top) from Tailem Bend, South Australia, 26 January 1969, B.B. Lowery, limestone mallee [ANIC32-900136] (ANIC). Paratypes: 4 major workers and 2 minor workers on same pin and with same details as holotype (ANIC); 2 minor workers, minor worker and 2 major workers from Blyth, South Australia, 28 November 1957, B. Lowery, ‘*M. laticeps* Wh.’ [*sic*] (ANIC); 3 major workers from Big Desert, Victoria, 10 December 1979, A. N. Andersen, ‘*Melophorus*’ (BMNH); 2 major workers from 7 km NW of Morgan, 16 December 1976, P.J.M. Greenslade, (6) (MCZ); media worker from Mundoora National Park, South Australia, 16 January 1975, P.J.M. Greenslade (SAM).

#### Other material examined.

**New South Wales**: Bogan River (Armstrong, J.), Callubri Station (Armstrong, J.). **South Australia**: Cambrai (Greenslade, P.J.M.), Cambrai (Greenslade, P.J.M.), Cambrai (Greenslade, P.J.M.), Cambrai (Greenslade, P.J.M.), Murray Bridge (Lea), Scorpion Springs CP, at northern entrance (Gross, G. F. [M85]), Waikerie (Greenslade, P.J.M.).

#### Diagnosis.

*Melophorus cerasinoniger* can be placed in the *M. biroi* species-group on the basis of characters of the clypeus, propodeum, mandible and palps. The species is also placed in the *M. wheeleri* species-complex because it agrees with the following apomorphies possessed by the complex: the minor worker often has more than five teeth, the largest major worker has a short, massive, elbowed mandible directed posteriad; in profile, the maxillary palps are short in the major and generally short in minor workers (in the minor worker, usually only attaining the neck sclerite at their maximum extent when the head is moderately inclined) and, in full-face view, the anterior margin of the clypeus in the large major worker is usually planar or weakly concave (variable in other subcastes but planar or narrowly protuberant anterior clypeal margins predominate). All workers of *M. cerasinoger* have a narrowly convex mesonotum, the pronotum being steeply (i.e., 60° >) inclined and flattened dorsally. Minor workers have a maximum of five mandibular teeth and in the major worker all teeth are on the same plane. This combination of characters, and in particular the conformation of the pronotum and mesonotum serves to distinguish the species.

#### Minor worker description.

**Head.** Head square; posterior margin of head weakly concave; frons shining and smooth except for piliferous pits; frons consisting exclusively or almost exclusively of well-spaced, appressed setae only (small, erect setae, if present, usually confined to ocular triangle or posterior margin of head). Eye moderate (eye length 0.20–0.49 length of side of head capsule); in full-face view, eyes set above midpoint of head capsule; in profile, eye set anteriad of midline of head capsule; eyes elliptical or slightly reniform. Frontal carinae straight or weakly convex in full-face view, or straight, divergent posteriad; frontal lobes straight in front of antennal insertion. Anteromedial clypeal margin broadly and evenly convex; clypeal psammophore set at or above midpoint of clypeus; palp formula 6,4. Five mandibular teeth in minor worker; mandibles triangular, weakly incurved; third mandibular tooth distinctly shorter than apical tooth and teeth numbers two and four; masticatory margin of mandibles approximately vertical or weakly oblique. **Mesosoma.** Integument of pronotum, mesonotum and mesopleuron shining and mainly smooth, vestigial shagreenation most noticeable on humeri and mesopleuron; anterior mesosoma in profile pronotum smoothly rounded anteriad and flattened posteriad, mesonotum narrowly convex; appearance of erect pronotal setae short and unmodified, or weakly expanded distally, or erect pronotal setae absent; in profile, metanotal groove deep, ‘V’-shaped; propodeum shining and microreticulate; propodeum always smoothly rounded; propodeal dorsum and declivity confluent; erect propodeal setae always absent; appressed propodeal setulae short, separated by more than own length and inconspicuous; propodeal spiracle situated on or beside declivitous face of propodeum, and shorter (length < 0.50 × height of propodeum). **Petiole.** In profile, petiolar node squamiform; in full-face view, shape of petiolar node uniformly rounded; node shining and smooth with vestigial sculpture. **Gaster.** Gaster shining, shagreenate (‘LP record’ appearance); pilosity of first gastral tergite consisting of well-spaced, erect and semi-erect setae interspersed with regularly placed appressed setae. **General characters.** Colour of foreparts bright brownish-orange, gaster black or blackish-brown.

#### Major worker description.

**Head.** Head horizontally rectangular, broader than wide; posterior margin of head weakly concave; cuticle of frons shining and smooth except for piliferous pits; frons consisting exclusively or almost exclusively of well-spaced, appressed setae only (small, erect setae, if present, usually confined to ocular triangle or posterior margin of head). Eyes small, (eye length less than 0.2 × length of head capsule); in full-face view, eyes set above midpoint of head capsule; in profile, eye set anteriad of midline of head capsule; eyes elliptical. In full-face view, frontal carinae straight, divergent posteriad; frontal lobes curved toward antennal insertion. Anterior clypeal margin straight, or broadly convex with anteromedial dimple; clypeal psammophore set at or above midpoint of clypeus; palp formula 6,4. Mandibular teeth in major worker always 4; mandibles strongly incurved, apical sector weakly carinate or incompletely carinate; third mandibular tooth distinctly shorter than apical tooth and teeth numbers two and four; masticatory margin of mandibles approximately aligned vertically or weakly oblique. **Mesosoma.** Integument of pronotum, mesonotum and mesopleuron shining and mainly smooth, vestigial shagreenation most noticeable on humeri and mesopleuron; anterior mesosoma in profile pronotum smoothly rounded anteriad and flattened posteriad, mesonotum narrowly convex; erect pronotal setae short, (i.e., shorter than length of eye) and unmodified; in profile, metanotal groove deep, v-shaped; propodeum shining and shagreenate; propodeum always smoothly rounded; propodeal dorsum and declivity confluent; erect propodeal setae variable in number, may be absent; appressed propodeal setae short and closely aligned in sparse patches; propodeal spiracle situated on or beside declivitous face of propodeum, and shorter (length less than 0.50 × height of propodeum). **Petiole.** In profile, petiolar node squamiform; in full-face view, shape of petiolar node uniformly rounded; node shining and smooth with vestigial microreticulation anteriad. **Gaster.** Gaster shining, shagreenate (‘LP record’ appearance); pilosity of first gastral tergite consisting of well-spaced, erect and semi-erect setae interspersed with regularly spaced appressed setae. **General characters.** Colour of foreparts bright brownish-orange, gaster black or blackish-brown.

#### Measurements.

Worker (n = 6): CI 116–124; EI 13–18; EL 0.23–0.32; HL 1.09–1.95; HW 1.26–2.42; ML 1.37–2.00; MTL 0.91–1.29; PpH 0.15–0.21; PpL 0.54–0.74; SI 55–82; SL 1.03–1.33.

#### Comments.

*Melophorus cerasinoniger* is an aesthetically appealing species because of its bright, usually cherry-red foreparts and black gaster (thus the name chosen here for the ant). Along with the colour, the steeply convex pronotum and flattened posterior pronotum and anterior mesonotum serve to separate this taxon from related or similar species. No specimens of this exclusively eastern Australian ant, found in NSW, SA and Vic, were available for sequencing. Interestingly, all collections of *M. cerasinoniger* have been taken from the Murray-Darling drainage system, and thus the ecological requirements of the species may be associated with features of the habitat to be found along that system of rivers. Be that as it may, the only ecological records indicate the species has been collected from dunes at Cambrai and in limestone mallee in Tailem Bend, both in SA.

#### Etymology.

Latin *cerasin* (‘of cherry’ [here, colour]) plus *niger* (‘black’); adjective in the nominative singular.

**Figure 78.**
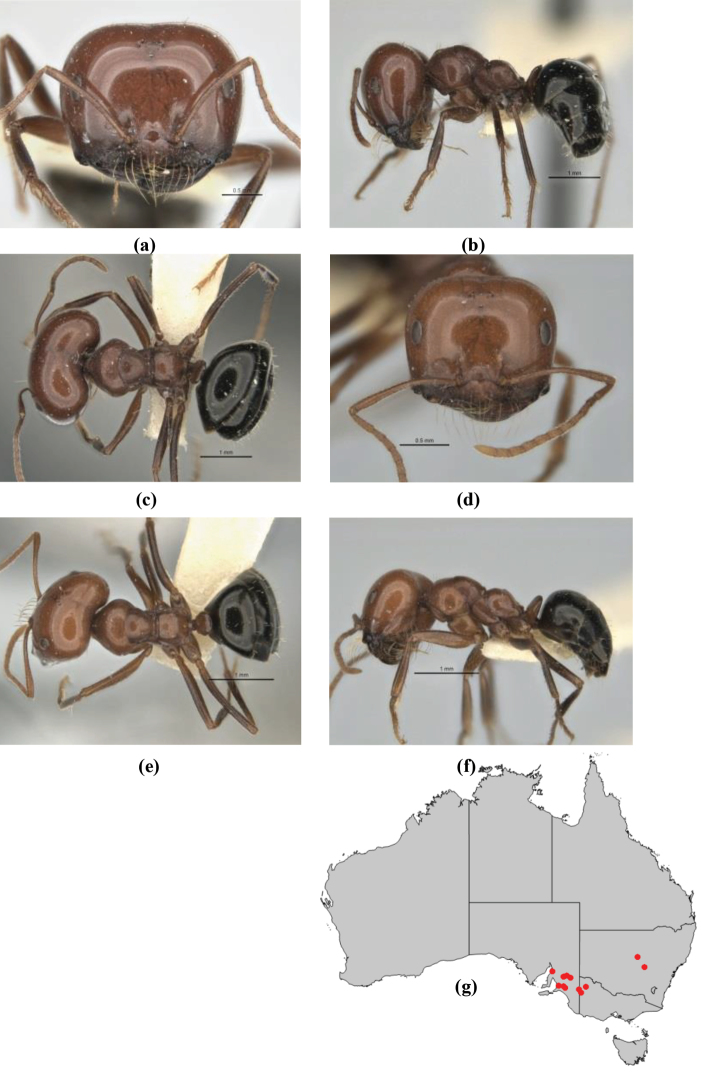
*Melophorus cerasinoniger* sp. n.: major worker paratype (ANIC32-900136–top ant) frons (**a**), profile (**b**) and dorsum (**c**); minor worker holotype (ANIC32-900136–fifth ant from top) frons (**d**), profile (**e**) and dorsum (**f**); distribution map for the species (**g**). Low resolution scale bars: 1 mm (**b, c**, **e, f**); 0.5 mm (**a, d**).

### Melophorus chauliodon

Taxon classificationAnimaliaHymenopteraFormicidae

Heterick, Castalanelli & Shattuck sp. n.

http://zoobank.org/49B91F9C-9B74-493B-B358-537FA9C1183B

#### Types.

Holotype minor worker (middle ant) from Sandringham Station 25.03S, 139.03E, 55 km NW of Bedourie, Queensland, 21 February 1980, S. R. Morton, S2, 4 [ANIC32-900189] (ANIC). Paratypes: minor worker and major worker on same pin with same details as holotype (ANIC); 3 minor workers from 16 km W of Mt Aloysius 26.01S, 128.26E, Western Australia, 16 November 1977, J. E. Feehan, associated collector, T. A. Weir [ANIC32-900067] (ANIC); major worker from 80 km E of Emu, Victoria Desert, South Australia, 8 October 1976, P.J.M. Greenslade, (3) (BMNH); 3 media workers from Sturt National Park, New South Wales, 24 November 1979, P. J. M. Greenslade, (4), 37 (MCZ); major worker and media worker from 20 km W of Cameron Corner, South Australia, 25 November 1979, P.J.M. Greenslade, (1) (SAM); major and minor worker and male from Lexia 31°36'48"S, 115°56'49"E, Western Australia, 9 October 2005, B.E. Heterick, *Banksia* open woodland, *Adenanthos* understorey, white quartz sand [JDM32-001877] (WAM); 2 media workers and minor worker from Hope Valley 32°11'53"S, 115°50'14"E, Western Australia, 21 October 2007, B.E. Heterick, *Banksia*/*Allocasuarina* sandplain, grey over yellow sand [JDM32-001878] (WAM).

#### Other material examined.

**New South Wales**: Fowlers Gap (Greenslade, P.J.M.). **Northern Territory**: New Crown Homestead (Feehan, J.E.), Tanami (Greenslade, P.J.M.). **Queensland**: 40 km E Cameron Corner (Greenslade, P.J.M.), Bors Creek, E Eulo (Greenslade, P.J.M.), Eulo (Greenslade, P.J.M.), Sandringham (Greenslade, P.J.M.), Sandringham (Greenslade, P.J.M.), Sandringham (Greenslade, P.J.M.), Sandringham (Greenslade, P.J.M.), Sandringham (Greenslade, P.J.M.), Sandringham (Greenslade, P.J.M.), Sandringham (Greenslade, P.J.M.), Sandringham (Greenslade, P.J.M.), Sandringham Station, 55 km NW Bedourie (Morton, S.R.). **South Australia**: 10 mi W Childara Rock (Greenslade, P.J.M.), 12 km W Emu, Victoria Desert (Greenslade, P.J.M.), 14 km SbyW Beltana (Feehan, J.E.), 20 km NE Macumba Station (Davidson, D. & Morton, S.), 2 km NE Kalamurina Homestead (Feehan, J.E.), 30 km S Granite Downs (Greenslade, P.J.M.), 50 km S Coober Pedy (Davidson, D. & Morton, S.), 53 km E Vokes Hill, Victoria Desert (Greenslade, P.J.M.), 5 km S Anna Creek (Greenslade, P.J.M.), 5 km S Kallakoopah, 135 km ENE Macumba Homestead, Simpson Desert (Greenslade, P.J.M.), 85 km W Mabel Creek (Greenslade, P.J.M.), about 6.5 km N Leigh Creek (Feehan, J.E.), Birdsville Track, 12 km SW Etadunna (Forrest, J.), Brookfield Conservation Park (Shattuck, S.O.), Emu Camp, Victoria Desert (Greenslade, P.J.M.), Emu Camp, Victoria Desert (Greenslade, P.J.M.), Emu Camp, Victoria Desert (Greenslade, P.J.M.), Koonamore (Greenslade, P.J.M.), Lake Meramangye, Victoria Desert (Greenslade, P.J.M.), Mt Davies turnoff, Victoria Desert (Greenslade, P.J.M.), Mt Gunson, SE Woomera (Greenslade, P.J.M.), Nillinghoo, Koonamore (Greenslade, P.J.M.), Olympic Dam Site (Mathews, E. G., Watts, C.). **Western Australia**: 14 mi W Mt Webb (McInnes & Dowse), 2 mi W Koonalda Station (Greaves, T. [ANIC32-900068]), Binningup turnoff (Heterick, B.E. [M77/M122]), Bungulla (Greaves, T.), East Fremantle (Heterick, B.E. [JDM32-001879]), G[ascoyne]. J[unction]. Rd, 108 km E Carnarvon (Heterick, B.E. [M308]), Geraldton (Weatherill, L.), Hollywood (Greaves, T.), Kings Park (Lowery, B.B.), Mardathuna Rd turnoff (Heterick, B.E. [M26/M45]), Melville (Heterick, B.E. [M341]), Melville (Heterick, B.E.), Yoothapina (Hides, D. [JDM32-004851]).

#### Diagnosis.

*Melophorus chauliodon* can be placed in the *M. biroi* species-group on the basis of characters of the clypeus, propodeum, mandible and palps. The species is also placed in the *M. wheeleri* species-complex because it agrees with the following apomorphies possessed by the complex: the minor worker often has more than five teeth, the largest major worker has a short, massive, elbowed mandible directed posteriad; in profile, the maxillary palps are short in the major and generally short in minor workers (in the minor worker, usually only attaining the neck sclerite at their maximum extent when the head is moderately inclined) and, in full-face view, the anterior margin of the clypeus in the large major worker is usually planar or weakly concave (variable in other subcastes but planar or narrowly protuberant anterior clypeal margins predominate). *Melophorus chauliodon* major and media workers are readily identifiable by virtue of their having a variably offset tusk-like basal mandibular tooth. This feature is not found in any other *Melophorus*. The minor worker is less distinctive, but is glabrous, its eye is an elongated ellipse (length of eye ≥ 0.3 × length of side of head) and in full-face view it has a square head shape, and this combination of features sufficiently characterise it when compared with other members of the species-complex.

#### Minor worker description.

**Head.** Head square; posterior margin of head planar or weakly convex; frons shining with superficial shagreenation or microreticulation only, or matt or with weak sheen, microreticulate or microreticulate-shagreenate; frons consisting exclusively or almost exclusively of well-spaced, appressed setae only (small, erect setae, if present, usually confined to ocular triangle or posterior margin of head). Eye moderate (eye length 0.20–0.49 length of side of head capsule); in full-face view, eyes set at about midpoint of head capsule; in profile, eye set anteriad of midline of head capsule; eyes elliptical or slightly reniform, or elongate. In full-face view, frontal carinae straight or weakly convex; frontal lobes straight in front of antennal insertion. Anteromedial clypeal margin straight and retroussé anteromedially or weakly convex; clypeal psammophore set at or above midpoint of clypeus; palp formula 6,4. Five to seven mandibular teeth in minor worker; mandibles triangular, weakly incurved; third mandibular tooth distinctly shorter than apical tooth and teeth numbers two and four; masticatory margin of mandibles approximately vertical or weakly oblique. **Mesosoma.** Integument of pronotum, mesonotum and mesopleuron shining and mainly smooth, vestigial shagreenation most noticeable on humeri and mesopleuron; anterior mesosoma in profile broadly convex; erect pronotal setae absent; in profile, metanotal groove shallow, indicated mainly by an angle; propodeum shining and shagreenate; propodeum angulate, propodeal angle blunt; length ratio of propodeal dorsum to its declivity about1:1; erect propodeal setae always absent; appressed propodeal setulae short, separated by more than own length and inconspicuous; propodeal spiracle situated on or beside declivitous face of propodeum, and shorter (length < 0.50 × height of propodeum). **Petiole.** In profile, petiolar node squamiform; in full-face view, shape of petiolar node uniformly rounded; node shining and smooth throughout. **Gaster.** Gaster shining, shagreenate (‘LP record’ appearance); pilosity of first gastral tergite consisting of well-spaced short, inconspicuous, appressed setae only, erect setae always absent. **General characters.** Colour variable, ranging from foreparts orange or orange tan with dark brown gaster to concolorous brownish-black.

#### Major worker description.

**Head.** Head horizontally rectangular, broader than wide; posterior margin of head weakly concave; cuticle of frons striolate anteriad, smooth and shining posteriad; pilosity of frons a mixture of a few well-spaced, erect setae interspersed with appressed setae only. Eye small (eye length less than 0.2 × length of head capsule); in full-face view, eyes set above midpoint of head capsule, or set at about midpoint of head capsule; in profile, eye set anteriad of midline of head capsule; eyes elliptical. In full-face view, frontal carinae concave; frontal lobes straight in front of antennal insertion. Anterior clypeal margin broadly emarginate, or weakly crenulate at midpoint; clypeal psammophore set at or above midpoint of clypeus; palp formula 6,4. Five mandibular teeth in major worker; mandibles strongly incurved, apical sector uniformly carinate and forming a bifurcate, horizontal ledge that terminates in the basal and pre-basal teeth; third mandibular tooth distinctly shorter than apical tooth and teeth numbers two and four; masticatory margin of mandibles approximately aligned vertically or weakly oblique. **Mesosoma.** Integument of pronotum, mesonotum and mesopleuron moderately shining and shagreenate throughout; anterior mesosoma in profile broadly convex; erect pronotal setae short, (i.e., shorter than length of eye) and unmodified; in profile, metanotal groove shallow, broadly V- or U-shaped; propodeum shining, with multiple hair like striolae; propodeum smoothly rounded or with indistinct angle; propodeal dorsum and declivity confluent; erect propodeal setae present and sparse to moderate (1-12); appressed propodeal setae short, separated by more than own length and inconspicuous; propodeal spiracle situated on or beside declivitous face of propodeum, and shorter (length less than 0.50 × height of propodeum). **Petiole.** In profile, petiolar node squamiform; in full-face view, shape of petiolar node tapered with sharp vertex, or tapered with squared-off vertex; node shining and faintly shagreenate-microreticulate. **Gaster.** Gaster shining, shagreenate (‘LP record’ appearance); pilosity of first gastral tergite consisting of short, bristly, erect setae over well-spaced, short, appressed setae. **General characters.** Colour of foreparts brownish-orange to orange-brown, gaster dark brown to blackish-brown.

#### Measurements.

Worker (n = 8): CI 114–125; EI 14–32; EL 0.21–0.38; HL 0.57–2.22; HW 0.64–2.77; ML 0.85–2.34; MTL 0.58–1.49; PpH 0.10–0.26; PpL 0.34–0.90; SI 54–101; SL 0.65–1.51.

#### Comments.

*Melophorus chauliodon* is a morphologically and genetically compact species. The major and media workers are easily identified by the large and often tusk-like basal tooth that is variably situated along the basal margin. In many WA populations this tooth is highly-developed and, at the rear of the gape, virtually occludes the gap between the mandible and the anterior margin of the clypeus. In eastern populations the tooth is situated more anteriorly along the apical margin of the mandible and is less strongly developed. Minor workers lack this anatomical feature but can be distinguished from similar species by a combination of their glabrous mesosoma, the straight anterior margin of the clypeus, the appearance of the head capsule and the elongate eye. The five-gene tree suggests that this species falls within the *M. fieldi* complex; however the three-gene tree indicates otherwise, and places this species within the *M. wheeleri* species-complex, and this is likely to be the more correct placement in view of the morphology.

*Melophorus chauliodon* is found in all mainland states except the ACT and Vic. The species is also absent from Tasmania. Existing label data for dry, pinned material is scanty, but a specimen from SA was taken in cane grass. Sequenced material has been collected from red clay soil, mulga woodland and from suburban lawns. This species is particularly common in and around the Perth metropolitan area, and can occur in highly built up locations. The phylogenetic position the species occupies and the nature of the mandible suggests it is largely or wholly granivorous.

#### Etymology.

Greek *chaulios* (‘outstanding’/’impressive’) plus *odon* (‘tooth’); noun in the nominative singular standing in apposition to the generic name

**Figure 79.**
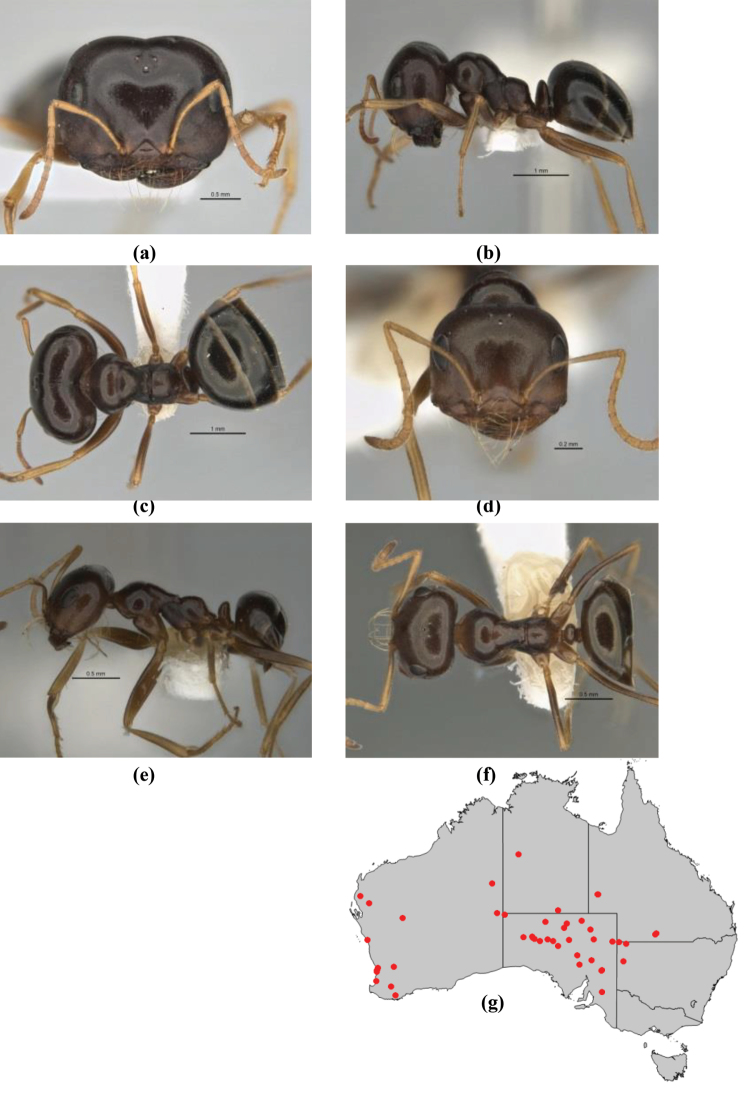
*Melophorus chauliodon* sp. n.: major worker paratype (ANIC32-900189–bottom ant) frons (**a**), profile (**b**) and dorsum (**c**); minor worker holotype (ANIC32-900189–middle ant) frons (**d**), profile (**e**) and dorsum (**f**); distribution map for the species (**g**). Low resolution scale bars: 1 mm (**b, c**); 0.5 mm (**a, e, f**); 0.2 (**d**).

### Melophorus diversus

Taxon classificationAnimaliaHymenopteraFormicidae

Heterick, Castalanelli & Shattuck sp. n.

http://zoobank.org/C06EE24B-D2D1-40E3-BFB2-60C276B93DA5

#### Types.

Holotype minor worker (bottom ant) from SW of Sandringham, Queensland, 7-13 August 1980, P.J.M. Greenslade, SRM Traps, (18), 30) [ANIC32-900186] (ANIC). Paratypes: minor worker on same pin and with same details as holotype (ANIC); media and 2 minor workers from Sandringham Station 25[*sic*-should be ‘24’-BEH].03S, 139.03E, 55 km NW of Bedourie, Queensland, 16 January 1980, S. R. Morton, p4 (BMNH); major worker and two media workers from Sturt National Park, New South Wales, 23 November 1979, P.J.M. Greenslade, (7) (MCZ); major worker from Sturt National Park, New South Wales, 22 November 1979, P.J.M. Greenslade, (4) [ANIC32-900062] (MCZ); media and 2 minor workers from Sandringham Station 24.03S, 139.03E, 55 km NW of Bedourie, Queensland, 16 January 1979, S. R. Morton, 12 [duplicate labels], ex C. (QM); 2 minor workers and a media worker from Kunoth Paddock, near Alice Springs, Northern Territory, 26 October 1974, P.J.M. Greenslade, (9), 3a) (SAM).

#### Other material examined.

**New South Wales**: 40 km NNW Louth, Lake Mere (Greenslade, P.J.M.), Callubri Station (Greaves, T.), Conservation Park, Fowlers Gap (Greenslade, P.J.M. [ANIC32-900061]), CSIRO Lake Mere Field Station, near Louth (Bryannah, M.), Fowlers Gap Station, 110 km N Broken Hill (Morton, S.), Fowlers Gap Station, 110 km N Broken Hill (Morton, S.), Fowlers Gap Station, 110 km N Broken Hill (Morton, S.), Olive Downs Station, 55 km NW Tibooburra (Morton, S.R.), Olive Downs Station, 55 km NW Tibooburra (Morton, S.R.), Olive Downs Station, 55 km NW Tibooburra (Morton, S.R.), Sturt National Park (Greenslade, P.J.M.), Sturt National Park (Greenslade, P.J.M.). **Queensland**: Sandringham (Greenslade, P.J.M.), Sandringham (Morton, S.), Sandringham (Morton, S.), Sandringham Station, 55 km NW Bedourie (Morton, S.R.), Thilla, 1:25000 Barrolka (Forrest, J.). **South Australia**: 2.8 km NNW Four Hills Trig. Peake Stn (Stony Desert Survey UB02 [M98/M99]), Innamincka, 3.8 km NNW Patchawarra (Stony Desert Surv. PC002 [M112]).

#### Diagnosis.

*Melophorus diversus* can be placed in the *M. biroi* species-group on the basis of characters of the clypeus, propodeum, mandible and palps. The species is also placed in the *M. wheeleri* species-complex because it agrees with the following apomorphies possessed by the complex: the minor worker often has more than five teeth, the largest major worker has a short, massive, elbowed mandible directed posteriad; in profile, the maxillary palps are short in the major and generally short in minor workers (in the minor worker, usually only attaining the neck sclerite at their maximum extent when the head is moderately inclined) and, in full-face view, the anterior margin of the clypeus in the large major worker is usually planar or weakly concave (variable in other subcastes but planar or narrowly protuberant anterior clypeal margins predominate). *Melophorus diversus* shares only with *M. wheeleri* the following combination of apomorphies: the anterior sector of the clypeus of minor worker is strongly folded back towards the mandible, and the clypeal psammophore is placed on a distinct ledge that may be carinate, the minor worker mandible has 5-9 teeth and denticles, the head, mesosoma and gaster of all workers have short, inconspicuous appressed setae that are usually separated by more than their own length (if more elongate, as in some small minor workers, then the ant is glossy and weakly sculptured) and the media and major workers are quite large *Melophorus* (HW of the large major worker ≥ 2.60 mm). Most workers of *Melophorus diversus* can be distinguished from *M. wheeleri* workers in having the head of the minor worker smooth with just vestigial sculpture (the head often glossy), erect non-marginal setae always present on first gastral tergite with one or two small erect setae also present on the pronotum of some individuals, the eye of the minor worker large (0.30× length of side of head capsule) and the major worker usually with many fine, erect setae on the mesosoma (though these may be more sparse and bristly in a few individuals). (In *Melophorus wheeleri* the head of minor worker always has some sculpture and is matt to moderately shining in appearance, erect setae are almost always lacking on the pronotum and the first gastral tergite, and the eye of the minor worker is usually less than 0.30× length of side of head capsule. The major worker is similar to the major worker of *M. diversus* but either has a glabrous mesosoma or possesses short, bristly setae on the mesosoma and is commonly matt in appearance [WA, NT] but may be glossy [eastern states]). However, the distinctions between these two species are not always observed and a few individuals, possibly hybrids, may be unable to be placed in either taxon with confidence.

#### Minor worker description.

**Head.** Head square, or quadrate (i.e., heart-shaped); posterior margin of head planar to strongly convex; frons shining with superficial shagreenation or microreticulation only, or matt or with weak sheen, shagreenate; frons consisting exclusively or almost exclusively of well-spaced, appressed setae only (small, erect setae, if present, usually confined to ocular triangle or posterior margin of head). Eye moderate (eye length 0.20–0.49 length of side of head capsule); in full-face view, eyes set above midpoint of head capsule; in profile, eye set anteriad of midline of head capsule, or set around midline of head capsule; eyes elliptical or slightly reniform, or elongate. In full-face view, frontal carinae straight or weakly convex; frontal lobes straight in front of antennal insertion. Anteromedial clypeal margin straight; clypeal psammophore set at or just above anterior clypeal margin; palp formula 6,4. Five to nine mandibular teeth in minor worker; mandibles triangular, weakly incurved; third mandibular tooth distinctly shorter than apical tooth, but equivalent in length to remaining teeth. **Mesosoma.** Integument of pronotum, mesonotum and mesopleuron moderately shining and shagreenate throughout; anterior mesosoma in profile broadly convex; erect pronotal setae absent; in profile, metanotal groove shallow, broadly V or U-shaped; propodeum shining and shagreenate; propodeum angulate, propodeal angle blunt; length ratio of propodeal dorsum to its declivity between 1:1 and 1:2; erect propodeal setae always absent; appressed propodeal setulae short, separated by more than own length and inconspicuous; propodeal spiracle situated on or beside declivitous face of propodeum, and shorter (length < 0.50 × height of propodeum). **Petiole.** In profile, petiolar node squamiform; in full-face view, shape of petiolar node uniformly rounded; node shining and smooth with vestigial sculpture. **Gaster.** Gaster shining, shagreenate (‘LP record’ appearance); pilosity of first gastral tergite consisting of well-spaced, erect and semi-erect setae interspersed with regularly placed appressed setae, or consisting of well-spaced short, inconspicuous, appressed setae, erect setae (present in at least some workers) confined to margin of sclerite. **General characters.** Colour light to dark brown, legs may be paler distally.

#### Major worker description.

**Head.** Head quadrate (i.e., heart-shaped); posterior margin of head weakly concave; cuticle of frons shining and smooth except for piliferous pits; frons consisting exclusively or almost exclusively of well-spaced, appressed setae only (small, erect setae, if present, usually confined to ocular triangle or posterior margin of head). Eyes small (eye length less than 0.2 × length of head capsule); in full-face view, eyes set above midpoint of head capsule; in profile, eye set anteriad of midline of head capsule; eyes elliptical. Frontal carinae concave in full-face view, or straight, divergent posteriad; frontal lobes curved inward in front of antennal insertion. Anterior clypeal margin broadly emarginate; clypeal psammophore set at or above midpoint of clypeus; palp formula 6,4. Mandibular teeth in major worker always 4; mandibles strongly incurved, apical sector weakly carinate or incompletely carinate; third mandibular tooth absent; masticatory margin of mandibles medially indented. **Mesosoma.** Integument of pronotum, mesonotum and mesopleuron moderately shining and shagreenate throughout; anterior mesosoma in profile broadly convex; erect pronotal setae short, (i.e., shorter than length of eye) and unmodified; in profile, metanotal groove shallow, broadly V- or U-shaped; propodeum shining and finely striolate and microreticulate; propodeum angulate, propodeal angle blunt; length ratio of propodeal dorsum to its declivity greater than 1:2; erect propodeal setae present and abundant (at least a dozen), or present and sparse to moderate (1-12); appressed propodeal setae short, separated by more than own length and inconspicuous; propodeal spiracle situated on or beside declivitous face of propodeum, and shorter (length less than 0.50 × height of propodeum). **Petiole.** In profile, petiolar node squamiform; in full-face view, shape of petiolar node tapered with median indentation; node shining and faintly shagreenate-microreticulate. **Gaster.** Gaster shining, shagreenate (‘LP record’ appearance); pilosity of first gastral tergite consisting of well-spaced, erect and semi-erect setae interspersed with regularly spaced appressed setae. **Colour.** Colour of head brownish-crimson to cherry red; trunk russet, legs light brown to ochre, gaster chocolate.

#### Measurements.

Worker (n = 6): CI 99–124; EI 14–38; EL 0.21–0.42; HL 0.56–2.48; HW 0.55–3.08; ML 0.74–2.31; MTL 0.45–1.39; PpH 0.09–0.26; PpL 0.31–0.93; S 45–103I; SL 0.57–1.37.

#### Comments.

As characterized here, *Melophorus diversus* is integrated in a clinal pattern with eastern Australian populations of *M. wheeleri*, and some specimens are not amenable to being allocated to either taxon with any confidence. In all likelihood, however, two separate taxa can be recognized here, albeit there appears to be limited gene flow, either simple hybridization or perhaps introgression, between the two species. The taxonomic key acknowledges the difficulty in determining the status of the workers of a few populations where gene flow with the parent species may be occurring. Apart from *M. wheeleri*, the ant can be distinguished from others in its complex by its glossy, weakly sculptured cuticle, the folded-back clypeus and the sparse nature of the appressed setae on the body and gaster, which are generally separated from one another by more than their own length. Sequencing of old material from SAMA has been unsuccessful, probably due to the denaturing of the preservative and the degradation of the DNA over time.

Most collections have made in NSW, with a few samples taken from southern QLD and one from Kunoth Paddock, near Alice Springs, in the NT. Regrettably, collection details are entirely lacking for this species.

#### Etymology.

Latin *diversus* (‘different’); adjective in the nominative singular.

**Figure 80.**
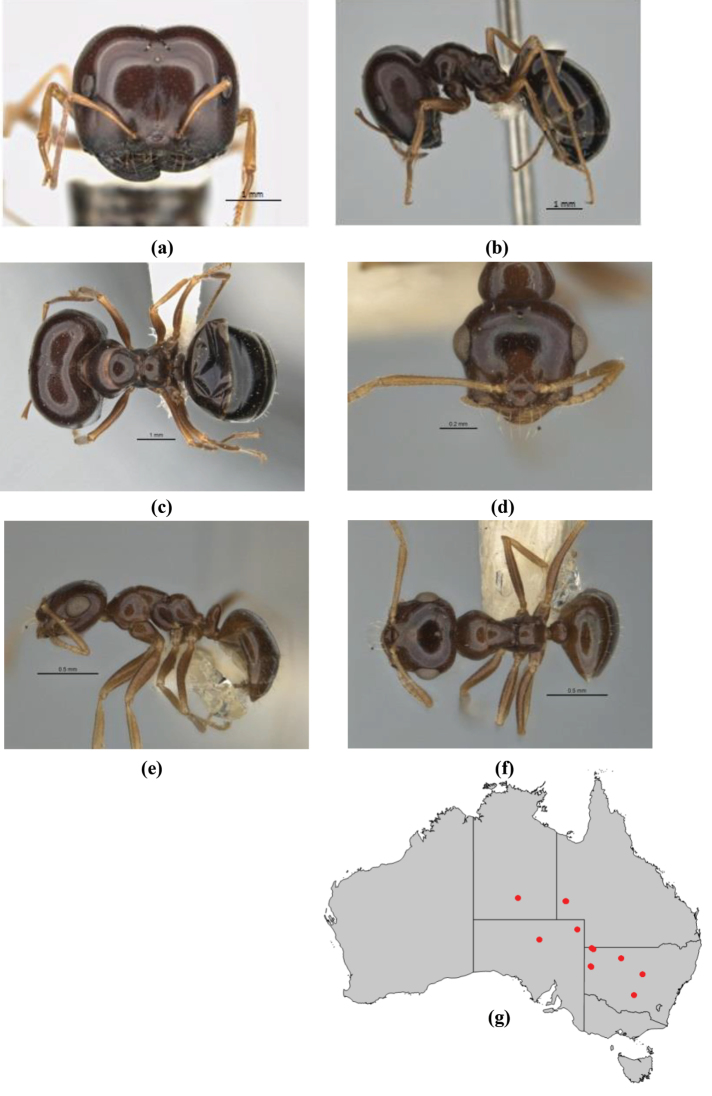
*Melophorus diversus* sp. n.: major worker paratype (ANIC32-900062) frons (**a**), profile (**b**) and dorsum (**c**); minor worker holotype (ANIC32-900186–bottom ant) frons (**d**), profile (**e**) and dorsum (**f**); distribution map for the species (**g**). Low resolution scale bars: 1 mm (**c**); 0.5 mm (**e, f**); 0,2 (**d**).

### Melophorus hexidens

Taxon classificationAnimaliaHymenopteraFormicidae

Heterick, Castalanelli & Shattuck sp. n.

http://zoobank.org/A9171A1C-28C9-4083-A09E-E160FF8AF276

#### Types.

Holotype minor worker (bottom ant) from Fowlers Gap, New South Wales, 18 November 1979, P.J.M. Greenslade, (9) [ANIC32-066603] (ANIC). Paratype: minor worker on same pin and with same details as holotype (ANIC); 3 minor workers from Lake Mere, 40 km NNW of Louth, New South Wales, 7 June 1986, P.J.M. Greenslade, (3), LM (ANIC); 3 minor workers from Cobbadah, 30°08'S, 150°46'E, NSW, March 2009, I. Oliver, Pitfall MEROO, 94T, ‘*Melophorus wheeleri*’ (MCZ); 3 minor workers from Cobbadah, 30°08'S, 150°46'E, NSW, March 2009, I. Oliver, Pitfall MEROO, 96, ‘*Melophorus wheeleri*’ (BMNH).

#### Diagnosis.

*Melophorus hexidens* can be placed in the *M. biroi* species-group on the basis of characters of the clypeus, propodeum, mandible and palps. The species is also placed in the *M. wheeleri* species-complex because it agrees with the following apomorphies possessed by the complex: the minor worker often has more than five teeth, the largest major worker has a short, massive, elbowed mandible directed posteriad; in profile, the maxillary palps are short in the major and generally short in minor workers (in the minor worker, usually only attaining the neck sclerite at their maximum extent when the head is moderately inclined) and, in full-face view, the anterior margin of the clypeus in the large major worker is usually planar or weakly concave (variable in other subcastes but planar or narrowly protuberant anterior clypeal margins predominate). *Melophorus hexidens* resembles common members of the *M. fieldi* species-complex in that it has an evenly protuberant clypeus and a psammophore that is set at the midpoint of the clypeus. However, it differs from such species in having six mandibular teeth and short maxillary palps that barely reach the neck sclerite. These features serve to distinguish *M. hexidens* from all other *Melophorus* of similar appearance. The major worker is unknown.

#### Minor worker description.

**Head.** Head square; posterior margin of head planar or weakly convex; frons matt or with weak sheen, microreticulate or microreticulate-shagreenate; frons consisting exclusively or almost exclusively of well-spaced, appressed setae only (small, erect setae, if present, usually confined to ocular triangle or posterior margin of head). Eye moderate (eye length 0.20–0.49 length of side of head capsule); in full-face view, eyes set above midpoint of head capsule; in profile, eye set anteriad of midline of head capsule; eyes elliptical or slightly reniform. In full-face view, frontal carinae distinctly concave; frontal lobes curved toward antennal insertion. Anteromedial clypeal margin broadly and evenly convex; clypeal psammophore set at or above midpoint of clypeus; palp formula 6,4. Mandibular teeth in minor worker six; mandibles triangular, weakly incurved; third mandibular tooth distinctly shorter than apical tooth, but equivalent in length to remaining teeth; masticatory margin of mandibles approximately vertical or weakly oblique. **Mesosoma.** Integument of pronotum, mesonotum and mesopleuron moderately shining and shagreenate throughout; anterior mesosoma in profile broadly convex; appearance of erect pronotal setae short, (i.e., longest erect setae shorter than length of eye) and unmodified, or erect pronotal setae absent; in profile, metanotal groove shallow, broadly V or U-shaped; propodeum shining and microreticulate; propodeum always smoothly rounded; propodeal dorsum and declivity confluent; erect propodeal setae always absent; appressed propodeal setulae sparse or absent, if present then not regularly spaced; propodeal spiracle situated at least twice its width from the declivitous face of propodeum, and shorter (length < 0.50 × height of propodeum). **Petiole.** In profile, petiolar node squamiform; in full-face view, shape of petiolar node uniformly rounded; node shining and smooth throughout. **Gaster.** Gaster shining, shagreenate (‘LP record’ appearance); pilosity of first gastral tergite consisting of well-spaced, erect and semi-erect setae interspersed with regularly placed appressed setae. **General characters.** Colour of foreparts orange tan, gaster brown.

#### Measurements.

Worker (n = 2): CI 106-108; EI 23-26; EL 0.24-0.25; HL 0.88-0.98; HW0.93-1.06; ML 1.21-1.29; MTL 0.75-0.81; PpH 0.13-0.16; PpL 0.50-0.54; SI 90-96; SL 0.89-0.96

#### Comments.

This species is known from four pins of minor workers collected at Fowler’s Gap, Lake Mere (presumably the Research Station near the lake) and Cobbadah, respectively, all sites being in NSW. These workers are very similar in general morphology to *M. turneri*, but differ in the number of mandibular teeth (six rather than five) and their short palps. They also resemble the (probably closely related) *M. purpureus*, but differ in the appearance of the clypeus and in the biogeography. The Cobbadah specimens were collected in pitfall traps. Nothing more is known of the species.

#### Etymology.

Compound of ancient Greek *hex* (‘six’) and Latin *dens* (‘tooth’); adjective in the nominative singular.

**Figure 81.**
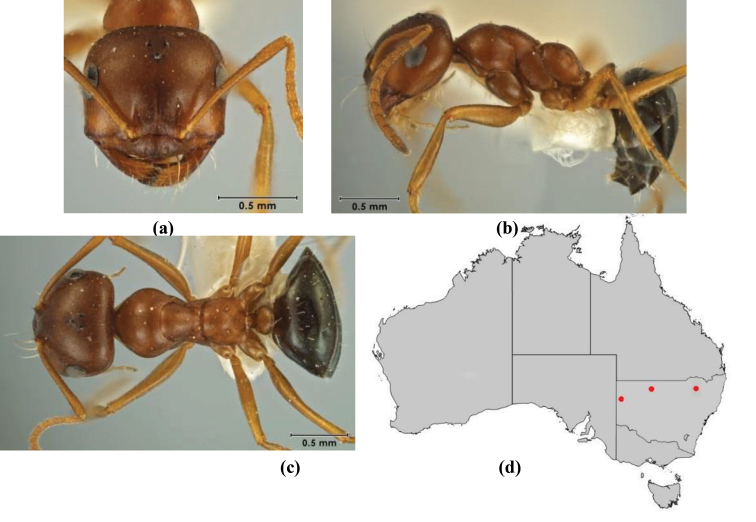
*Melophorus hexidens* sp. n.: holotype minor worker (ANIC32-066603–bottom ant) frons (**a**), profile (**b**) and dorsum (**c**); distribution map for the species (**d**).

### Melophorus laticeps

Taxon classificationAnimaliaHymenopteraFormicidae

Wheeler

Melophorus laticeps Wheeler 1915: 813, pl. 66, fig. 2 (combination in M. (Erimelophorus) by Wheeler 1935: 71. Type. Holotype queen: between Todmorden and Wantapella, South Australia [SAM] (examined: SAM specimen).

#### Other material examined.

**New South Wales**: 3 mi W Pindara Homestead (Greaves, T.), 40 km NNW Louth, Lake Mere (Greenslade, P.J.M.), 40 km NNW Louth, Lake Mere (Greenslade, P.J.M.), 40 km NNW Louth, Lake Mere (Greenslade, P.J.M.), 40 km W Broken Hill (Greenslade, P.J.M.), 40 km W Hillston (Morton, S.), 5 km W Broken Hill (Greenslade, P.J.M.), 5 mi WSW Topar Hotel, E Broken Hill (Greaves, T.), 6 mi W Tickalara Homestead (Greaves, T.), 7 mi SW Packsaddle Tank (Greaves, T.), about 37 km S Broken Hill (Kohout, R.J.), Acacia Vale, near Broken Hill (Greenslade, P.J.M.), Broken Hill (Lowery, B.B.), CSIRO Lake Mere Field Station, near Louth (Bryannah, M.), Duffy Lane, Broken Hill (Smith, T.), Finley (White, W.B.), Fowlers Gap (Greenslade, P.J.M.), Fowlers Gap (Greenslade, P.J.M.), Fowlers Gap (Greenslade, P.J.M.), Fowlers Gap (Greenslade, P.J.M.), Fowlers Gap (Greenslade, P.J.M.), Fowlers Gap (Greenslade, P.J.M.), Fowlers Gap (Ellis, M.), Fowlers Gap (Greenslade, P.J.M.), Fowlers Gap (Greenslade, P.J.M.), Fowlers Gap Station, 110 km N Broken Hill (Morton, S.), Fowlers Gap, near Sandstone Tank (Ward, P.S. [ANIC32-010588]), Hay, Kavs Station, Broken Hill (Lowery, B.B.), Menindee (Lowery, B.B.), Mundi Mundi, near Broken Hill (Valentine, J.), Netallie Hill, 10 mi W Wilcannia (Greaves, T.), Sturt National Park (Greenslade, P.J.M.), Tibooburra (L.C.), Tibooburra Aerodrome (Greaves, T.), Tilpa (Lowery, B.B.). **Northern Territory**: 20 km SE Alice Springs (Davidson, D. & Morton, S.), 5 mi N Alice Springs (McInnes & Dowse), 5 mi N Alice Springs (McInnes & Dowse), Alice Springs (Harrington, S.A. [ANIC32-000711]), Kunoth Paddock, near Alice Springs (Greenslade, P.J.M.), Kunoth Paddock, near Alice Springs (Greenslade, P.J.M.), Simpson Gap (Feehan, J.E.). **Queensland**: ‘Gumbardo’ (Beutel, T.), ‘Merigol’ (Beutel, T.), 75 km W Sandringham Homestead (Greenslade, P.J.M.), 8 mi NNE Nockatunga (Greaves, T.), Mareeba (Hill, H.E.), Mareeba (Lowery, B.B.), Marino (Wetherly, A.), Miles (Lowery, B.B.), Millungera Station (Greaves, T.), Nockatunga Station (Greaves, T.), Sandringham (Greenslade, P.J.M.), Sandringham (Greenslade, P.J.M.), Sandringham (Greenslade, P.J.M.), Sandringham (Greenslade, P.J.M.), Sandringham (Greenslade, P.J.M.), Sandringham (Greenslade, P.J.M.), Sandringham (Greenslade, P.J.M.), Sandringham (Greenslade, P.J.M.), Sandringham (Greenslade, P.J.M.), Sandringham (Greenslade, P.J.M.), Sandringham (Greenslade, P.J.M.), Sandringham Station, 55 km NW Bedourie (Morton, S.R.), Sandringham Station, 55 km NW Bedourie (Morton, S.R.), Sandringham Station, 55 km NW Bedourie (Morton, S.R.), Sandringham Station, 55 km NW Bedourie (Morton, S.R.), Sandringham Station, 55 km NW Bedourie (Morton, S.R.). **South Australia**: 10 km NW Kimba (Greenslade, P.J.M.), 10 km SW Morgan (Halliday, R.B.), 11 mi NE Elliston (Greaves, T.), 12 mi E Penong (Greaves, T.), 20 km S Welbourne Hill (Greenslade, P.J.M.), 30 km E Poeppel Corner, Simpson Desert (Greenslade, P.J.M. [ANIC32-900187]), 35 mi SSE Emu (Forde, N.), 38 mi W Nullarbor Station (Greaves, T.), 39 mi SSE Ooldea (McInnes & Dowse), 50 km E Purni Bore, 150 km NE Macumba Homestead, Simpson Desert (Greenslade, P.J.M.), 5 km SE Oodnadatta (Davidson, D. & Morton, S. [ANIC32-900065]), 7 km NW Morgan (Greenslade, P.J.M.), 7 km NW Morgan (Greenslade, P.J.M.), 7 km NW Morgan (Greenslade, P.J.M.), 7 km NW Morgan (Greenslade, P.J.M.), 7 km NW Morgan (Greenslade, P.J.M.), about 25 km W Marree (Feehan, J.E.), about 6.5 km N Leigh Creek (Feehan, J.E.), Birdsville Track, 12 km SW Etadunna (Forrest, J.), Birthday Hill, N Tarcoola (Greenslade, P.J.M.), Cambrai (Greenslade, P.J.M.), Cambrai (Greenslade, P.J.M.), Cambrai (Greenslade, P.J.M.), Cambrai (Greenslade, P.J.M.), Ceduna (Lowery, B.B.), Chowilla (Greenslade, P.J.M.), Chowilla (Wood, T.G.), Davenport Ra (Gee, P. & I. [M86/M101]), head of Bight Cliff, 54 km WbyS Nullarbor (Taylor, R.W.), Moorowie Plain (Greenslade, P.J.M.), Mt Gason, about 41 km SSW Clifton Hills Homestead (Feehan, J.E.), near Macumba Rail Station (Bunic, I.), Nillinghoo, Koonamore (Greenslade, P.J.M. [ANIC32-900188]), Poochera (Taylor, R.W. & Bartell, R.J.), Poochera (Billen, J. [ANIC32-013311]), Streaky Bay (Lowery, B.B.), Streaky Bay (McAreavey, J.), Yudnamutana, NE Flinders Range (Lowery, B.B. [ANIC32-900066]). **Victoria**: Pink Lake (Graham, N.). **Western Australia**: 1 km S Capricorn RH (Heterick, B.E. [M282]), 5 km N Cunyu Station (Davidson, D. & Morton, S.), 5 km N Cunyu Station (Davidson, D. & Morton, S.), 9 mi E Cardawan Homestead, SSW Mundiwindi (McInnes & Dowse), Ethel Creek (Varris, P.A. [JDM32-004806]).

#### Diagnosis.

*Melophorus laticeps* can be placed in the *M. biroi* species-group on the basis of characters of the clypeus, propodeum, mandible and palps. The species is also placed in the *M. wheeleri* species-complex because it agrees with the following apomorphies possessed by the complex: the minor worker often has more than five teeth, the largest major worker has a short, massive, elbowed mandible directed posteriad; in profile, the maxillary palps are short in the major and generally short in minor workers (in the minor worker, usually only attaining the neck sclerite at their maximum extent when the head is moderately inclined) and, in full-face view, the anterior margin of the clypeus in the large major worker is usually planar or weakly concave (variable in other subcastes but planar or narrowly protuberant anterior clypeal margins predominate). In full-face view, the broad head capsule of the minor worker of *M. laticeps* is expanded towards the mandibular insertions, giving it a slight to strongly accentuated trapezoidal shape, and the basal margin of the major worker mandible is a carinate ledge set at 90° to the rest of the mandible throughout its length, with the (slightly) offset basal tooth horizontal and the sub-basal tooth with a horizontal and a vertical plane. A mandibular carina is present to varying degrees in media and minor workers. All workers have a glabrous mesosoma. These characters will distinguish *M. laticeps* from all other *Melophorus* except for its close relation, *M. pelorocephalus*. The heads of minor and media workers of *M. pelorocephalus*, however, are even more exaggeratedly trapezoidal and the upper head of this species is distinctly darker in colour than the lower head and the mesosoma (all are uniformly coloured in *M. laticeps*).

#### Minor worker description.

**Head.** Head square or rectangular, tending to trapezoid; posterior margin of head planar or weakly convex; frons shining with superficial shagreenation or microreticulation only; frons consisting exclusively or almost exclusively of well-spaced, appressed setae only (small, erect setae, if present, usually confined to ocular triangle or posterior margin of head). Eye moderate (eye length 0.20–0.49 length of side of head capsule); in full-face view, eyes set above midpoint of head capsule; in profile, eye set anteriad of midline of head capsule; eyes elliptical or slightly reniform. In full-face view, frontal carinae distinctly concave; frontal lobes straight in front of antennal insertion. Anteromedial clypeal margin straight; clypeal psammophore set at or above midpoint of clypeus; palp formula 6,4. Five to six mandibular teeth in minor worker; mandibles triangular, weakly incurved; third mandibular tooth distinctly shorter than apical tooth and teeth numbers two and four; masticatory margin of mandibles approximately vertical or weakly oblique. **Mesosoma.** Integument of pronotum, mesonotum and mesopleuron moderately shining and shagreenate throughout; anterior mesosoma in profile convex anteriad, mesonotum often slightly overlapping pronotum, mesosoma planar or slightly sinuate posteriad; erect pronotal setae absent; in profile, metanotal groove shallow, broadly V or U-shaped; propodeum shining and shagreenate; propodeum smoothly rounded or with indistinct angle; propodeal dorsum and declivity confluent; erect propodeal setae always absent; appressed propodeal setulae short, separated by more than own length and inconspicuous; propodeal spiracle situated nearer to midpoint of propodeum than to its declivitous face, and shorter (length < than 0.50 × height of propodeum). **Petiole.** In profile, petiolar node narrowly conical, vertex blunt, directed posteriad; in full-face view, shape of petiolar node tapered with blunt vertex; node shining and smooth with vestigial sculpture. **Gaster.** Gaster shining, shagreenate (‘LP record’ appearance); pilosity of first gastral tergite consisting of well-spaced short, inconspicuous, appressed setae, erect setae (present in at least some workers) confined to margin of sclerite. **General characters.** Colour of foreparts tan or reddish (legs may be darker) gaster dark brown to black.

#### Major worker description.

**Head.** Head horizontally rectangular, broader than wide; posterior margin of head weakly concave; cuticle of frons shining and smooth except for piliferous pits; pilosity of frons a mixture of a few well-spaced, erect setae interspersed with appressed setae only. Eye small (eye length less than 0.2 × length of head capsule); in full-face view, eyes set above midpoint of head capsule; in profile, eye set anteriad of midline of head capsule; eyes elliptical. In full-face view, frontal carinae distinctly concave; frontal lobes curved toward antennal insertion. Anterior clypeal margin straight; clypeal psammophore set at or above midpoint of clypeus; palp formula 6,4. Mandibular teeth in major worker always 4; mandibles strongly incurved, apical sector uniformly carinate and forming a bifurcate, horizontal ledge that terminates in the basal and pre-basal teeth; third mandibular tooth distinctly shorter than apical tooth, but equivalent in length to remaining teeth; masticatory margin of mandibles approximately aligned vertically or weakly oblique. **Mesosoma.** Integument of pronotum, mesonotum and mesopleuron shining and mainly smooth, vestigial shagreenation most noticeable on humeri and mesopleuron; anterior mesosoma in profile broadly convex; erect pronotal setae long (i.e., longer than length of eye) and unmodified, or erect pronotal setae absent; in profile, metanotal groove shallow, indicated mainly by an angle and metathoracic spiracles; propodeum always smoothly rounded; propodeal dorsum and declivity confluent; erect propodeal setae absent; appressed propodeal setae short, separated by more than own length and inconspicuous; propodeal spiracle situated nearer to midpoint of propodeum than to its declivitous face, and shorter (length less than 0.50 × height of propodeum), or situated at least twice its width from the declivitous face of propodeum, and shorter (length less than 0.50 × height of propodeum). **Petiole.** In profile, petiolar node squamiform; in full-face view, shape of petiolar node uniformly rounded, or tapered with blunt vertex; node shining and smooth throughout. **Gaster.** Gaster shining, shagreenate (‘LP record’ appearance); pilosity of first gastral tergite consisting of well-spaced, erect and semi-erect setae interspersed with regularly spaced appressed setae, or consisting of well-spaced short, inconspicuous, appressed setae, erect setae (present in at least some workers) confined to margin of the sclerite. **General characters.** Colour of foreparts reddish (legs light reddish-brown to brown), gaster black.

#### Measurements.

Worker (n = 8): CI 128–134; EI 11–19; EL 0.22–0.37; HL 0.89–2.56; HW 1.14–3.43; ML 1.49–2.64; MTL 1.13–1.95; PpH 0.16–0.27; PpL 0.57–1.08; SI 49–99; SL 1.13–1.69.

#### Comments.

This common species is found in all mainland states except the ACT, but is absent from wetter areas. *Melophorus laticeps* has a massive, carinate mandible that distinguishes it from all other *Melophorus* except for its close relative, *M. pelorocephalus*. Seen in full-face view, the latter has a trapezoidal head shape compared with a square shape in *M. laticeps*. Only one WA specimen of *M. laticeps* has been sequenced, but this is sufficient to reveal a sister relationship with *M. pelorocephalus*.

The species was described by Wheeler from a queen. This specimen, although damaged, exhibits the characteristic mandible that enables it to be associated easily with the worker (the head capsule is not splayed outwards near the mandibular insertions as would almost certainly be the case in the [as yet unknown] queen of *M. pelorocephalus*, the only alternative assignment). The ant has been taken in mulga and arid woodland; but the only edaphic note is that a specimen from near the Capricorn Roadhouse in WA was collected from red soil. The appearance of the mandible suggests a granivorous diet, but there are no data at present to confirm this.

**Figure 82.**
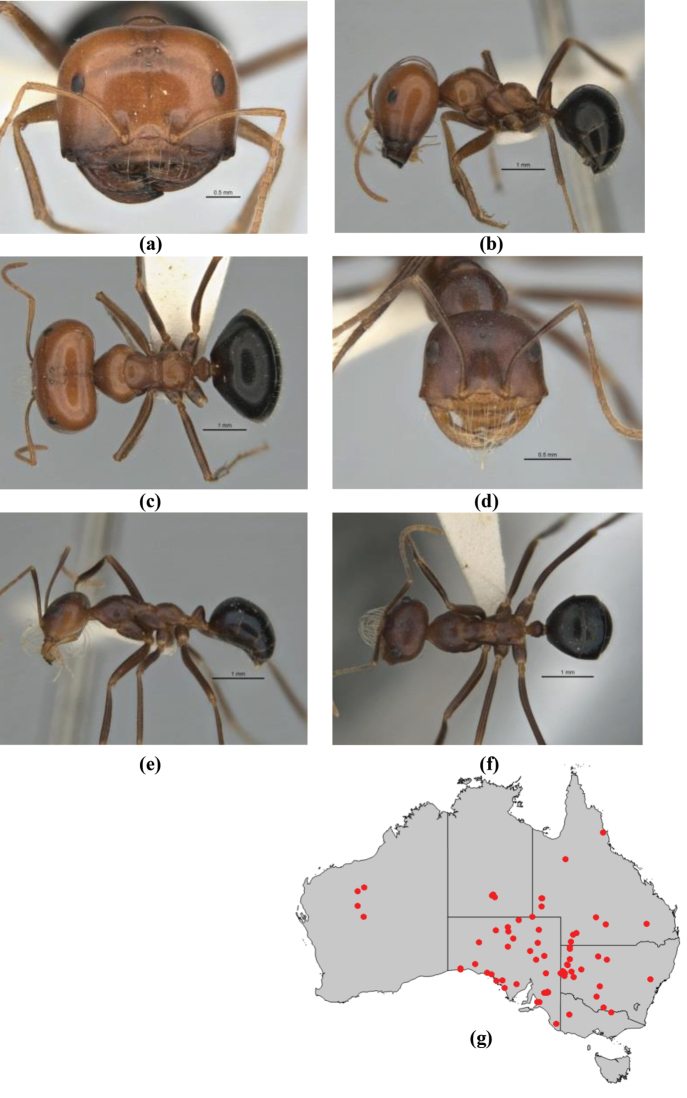
*Melophorus laticeps* Wheeler: no**n–t**ype major worker (ANIC32-900188) frons (**a**), profile (**b**) and dorsum (**c**); non–type minor worker (ANIC32-900187) frons (**d**), profile (**e**) and dorsum (**f**); distribution map for the species (**g**). Low resolution scale bars: 1 mm (**b, c**, **e, f**); 0.5 mm (**a, d**).

### Melophorus parvimolaris

Taxon classificationAnimaliaHymenopteraFormicidae

Heterick, Castalanelli & Shattuck sp. n.

http://zoobank.org/776666DC-49DF-48AD-B1D4-2FD508529701

#### Types.

Holotype minor worker (top ant) from Mt Bruce 23°35'42"S, 118°11'43"E, Western Australia, September 1991, S. van Leeuwen, Fire/Mulga research 3-1a [JDM32-004558] (WAM). Paratypes: Minor worker on same pin and with same details as holotype (WAM); major worker from Tanami, Northern territory, 17–21 April 1986, P.J.M. Greenslade, 11) [ANIC32-900134] (ANIC); 3 minor workers from 11 km N of Tennant Creek 19°32'S, 134°13'E, Northern Territory, 11 October 1981, D. Davidson/ S. Morton, 149C (MCZ).

#### Other material examined.

**Northern Territory**: Tanami (Greenslade, P.J.M.). **Western Australia**: 2 km SW Marda Pool (CALM Pilbara Survey [JDM32-004557]), 3.9 km N Kumarina (Heterick, B.E. [M173/M174]), 5 km N Kumarina (Heterick, B.E. [M283]), Yoothepina [Yoothapina] (Hides, D. [JDM32-001822]).

#### Diagnosis.

*Melophorus parvimolaris* can be placed in the *M. biroi* species-group on the basis of characters of the clypeus, propodeum, mandible and palps. The species is also placed in the *M. wheeleri* species-complex because it agrees with the following apomorphies possessed by the complex: the minor worker often has more than five teeth, the largest major worker has a short, massive, elbowed mandible directed posteriad; in profile, the maxillary palps are short in the major and generally short in minor workers (in the minor worker, usually only attaining the neck sclerite at their maximum extent when the head is moderately inclined) and, in full-face view, the anterior margin of the clypeus in the large major worker is usually planar or weakly concave (variable in other subcastes but planar or narrowly protuberant anterior clypeal margins predominate). The clypeus of *M. parvimolaris* is distinctly folded back towards the mandible and the clypeal psammophore is placed on a ledge in the minor worker, the head, mesosoma and gaster have relatively long, whitish, appressed setae that overlap and form a weak pubescence on the gaster and the ant has a distinct microreticulate or shagreenate sculpture and is matt or has a weak sheen. The minor worker has five mandibular teeth and the HW of the major worker is quite small (HW ≈ 1.45 mm). These characters serve to differentiate *M. parvimolaris* from all other *Melophorus* except *M. xouthos*. However, *M. parvimolaris* is uniformly brown, whereas *M. xouthos* is tawny orange-and-black.

#### Minor worker description.

**Head.** Head square or rectangular, tending to trapezoid; posterior margin of head weakly convex; frons matt or with weak sheen, shagreenate; frons consisting exclusively or almost exclusively of well-spaced, appressed setae only (small, erect setae, if present, usually confined to ocular triangle or posterior margin of head). Eye moderate (eye length 0.20–0.49 length of side of head capsule); in full-face view, eyes set above midpoint of head capsule; in profile, eye set anteriad of midline of head capsule; roughly ovoid, eye narrowed posteriad. In full-face view, frontal carinae distinctly concave; frontal lobes straight in front of antennal insertion. Anteromedial clypeal margin straight; clypeal psammophore set at or just above anterior clypeal margin; palp formula 6,4. Five mandibular teeth in minor worker; mandibles triangular, weakly incurved; third mandibular tooth distinctly shorter than apical tooth and teeth numbers two and four; masticatory margin of mandibles approximately vertical or weakly oblique. **Mesosoma.** Integument of pronotum, mesonotum and mesopleuron with weak to moderate sheen, shagreenate on pronotum and dorsum of mesonotum, otherwise microreticulate; anterior mesosoma in profile weakly elevated anteriad, thereafter gently sinuate, pronotum and mesonotum on same plane; erect pronotal setae absent; in profile, metanotal groove shallow, indicated mainly by an angle; propodeum matt or with a weak sheen and microreticulate; propodeum smoothly rounded or with indistinct angle; propodeal dorsum and declivity confluent; erect propodeal setae always absent; appressed propodeal setulae long, each reaching setae behind and in front, but not forming pubescence; propodeal spiracle situated on or beside declivitous face of propodeum, and shorter (length < 0.50 × height of propodeum). **Petiole.** In profile, petiolar node squamiform; in full-face view, shape of petiolar node tapered with blunt vertex; node shining and distinctly microreticulate, or matt and microreticulate. **Gaster.** Gaster weakly shining with indistinct shagreenation; pilosity of first gastral tergite consisting of well-spaced, long, whitish, appressed setae only, erect setae always absent. **General characters.** Colour concolorous chocolate.

#### Major worker description.

**Head.** Head horizontally rectangular, broader than wide; posterior margin of head planar or weakly concave; cuticle of frons matt or with weak sheen, indistinctly shagreenate; frons consisting mainly of appressed and stout erect setae, the latter bristly in appearance and distinctly modified (flattened) distally. Eye moderate (eye length 0.20–0.49 length of head capsule); in full-face view, eyes set above midpoint of head capsule; in profile, eye set anteriad of midline of head capsule; eyes elliptical. In full-face view, frontal carinae straight, divergent posteriad; frontal lobes straight in front of antennal insertion. Anterior clypeal margin broadly emarginate; clypeal psammophore set at or just above anterior clypeal margin; palp formula 6,4. Four mandibular teeth in major worker-5; mandibles strongly incurved, apical sector weakly carinate or incompletely carinate; third mandibular tooth distinctly shorter than apical tooth, but equivalent in length to remaining teeth; masticatory margin of mandibles approximately aligned vertically or weakly oblique. **Mesosoma.** Integument of pronotum, mesonotum and mesopleuron matt or with weak sheen and microreticulate throughout; anterior mesosoma in profile broadly convex; erect pronotal setae absent; in profile, metanotal groove shallow, indicated mainly by an angle and metathoracic spiracles; propodeum matt or with a weak sheen and microreticulate; propodeum angulate, propodeal angle blunt; length ratio of propodeal dorsum to its declivity between 1:1 and 1:2; erect propodeal setae variable in number, may be absent; appressed propodeal setae long and closely aligned, creating pubescence; propodeal spiracle situated on or beside declivitous face of propodeum, and shorter (length less than 0.50 × height of propodeum). **Petiole.** In profile, petiolar node squamiform; in full-face view, shape of petiolar node tapered with blunt vertex; node matt, shagreenate. **Gaster.** Gaster weakly shining with indistinct shagreenation; pilosity of first gastral tergite consisting of thick, often distally flattened, erect setae over well-spaced, short, appressed setae. **General characters.** Colour concolorous blackish-brown.

#### Measurements.

Worker (n = 6): CI 109–123; EI 17–30; EL 0.20–0.27; HL 0.61–1.23; HW 0.67–1.52; ML 0.87–1.38; MTL 0.55–0.91; PpH 0.09–0.16; PpL 0.32–0.57; SI 59–101; SL 0.68–0.89.

#### Comments.

This small, dull brown member of the *M. laticeps* species-group can be recognized by features of the clypeus, the presence of long, whitish, appressed setae on the body and gaster, and its mostly matt, shagreenate or microreticulate cuticle. *Melophorus xouthos* shares these features, but is bicoloured tawny orange-and-black and has legs with a bluish, iridescent sheen. Thus far *M. parvimolaris* has only been collected in arid and semi-arid regions in the NT and WA, but it probably also occurs in SA in areas of suitable habitat. Sequencing data on a five-gene tree suggests a sister relationship with *M. caeruleoviolaceus*, but this cannot be not assumed (see comments under the latter species). The ant is likely to be wholly or largely granivorous: on two separate occasions just north of Kumarina, WA, the principal author saw minor workers collecting small, wind-blown seeds.

#### Etymology.

Latin *parvus* (‘small’) plus *molaris* (‘adapted for grinding’, ‘molar tooth’); adjective in the nominative singular.

**Figure 83.**
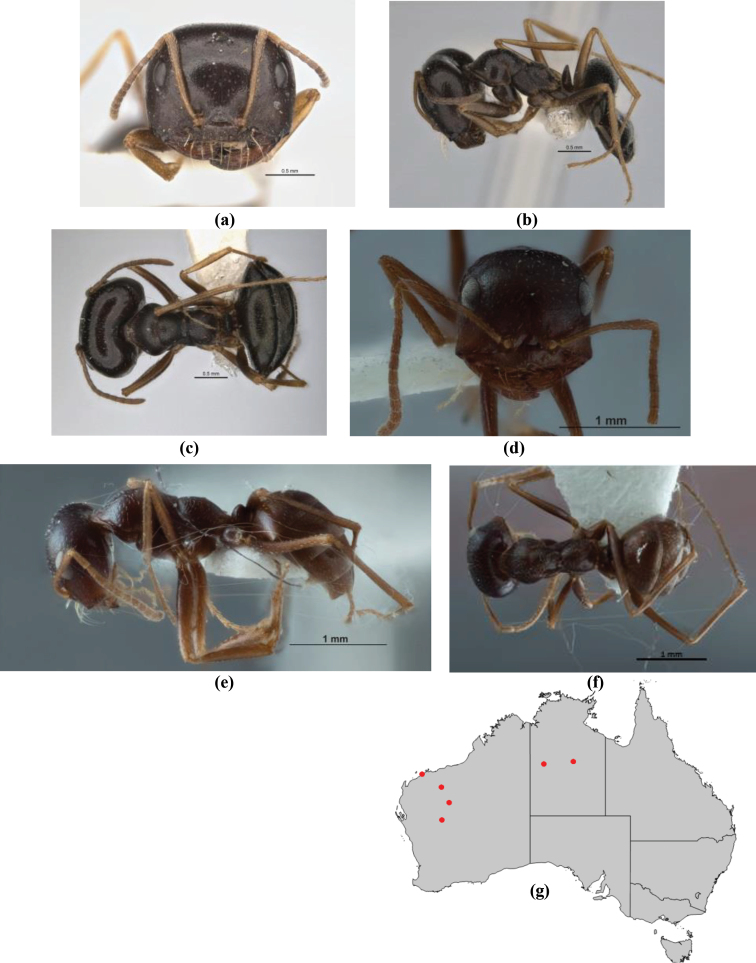
*Melophorus parvimolaris* sp. n.: major worker paratype (ANIC32-900134) frons (**a**), profile (**b**) and dorsum (**c**); minor worker holotype (JDM32-004558–top ant) frons (**d**), profile (**e**) and dorsum (**f**); distribution map for the species (**g**). Low resolution scale bars: 1 mm (**a–c**).

### Melophorus pelorocephalus

Taxon classificationAnimaliaHymenopteraFormicidae

Heterick, Castalanelli & Shattuck sp. n.

http://zoobank.org/5805B1E4-DD93-45DA-B7EB-2E3661EDF70F

#### Types.

Holotype minor worker (top ant) from 142.1 km SSE of Newman 24°31'53"S, 120°17'24"E, Western Australia, October 1996, S. van Leeuwen & R. N. Bromilow, Temp. invert. Pitfall trap, B3: Sand dune [JDM32-004714] (WAM). Paratypes: 2 minor workers on same pin with same details as holotype (WAM); minor and media worker from 142 km SSE of Newman 24°31'53"S, 120°17'24"E, Western Australia, October 1996, S. van Leeuwen & R. N. Bromilow, Temp. invert. Pitfall trap, B3: Sand dune [JDM32-004852] (ANIC); 2 major and a media worker from Tropicana Minesite 29°15'42"S, 124°30'47"E, Western Australia, January 2009, J. Summerhayes, pifall trap: marble dune, MD2:1 [JDM32-004898] (WAM).

#### Other material examined.

**South Australia**: Three Forges Waterhole (Gee, P. & L. (122) [M81/M104]). **Western Australia**: Little Sandy Desert (Guthrie, N. A. [ANIC32-900214]), Little Sandy Desert (Guthrie, N. A. [ANIC32-900215]).

#### Diagnosis.

*Melophorus pelorocephalus* can be placed in the *M. biroi* species-group on the basis of characters of the clypeus, propodeum, mandible and palps. The species is also placed in the *M. wheeleri* species-complex because it agrees with the following apomorphies possessed by the complex: the minor worker often has more than five teeth, the largest major worker has a short, massive, elbowed mandible directed posteriad; in profile, the maxillary palps are short in the major and generally short in minor workers (in the minor worker, usually only attaining the neck sclerite at their maximum extent when the head is moderately inclined) and, in full-face view, the anterior margin of the clypeus in the large major worker is usually planar or weakly concave (variable in other subcastes but planar or narrowly protuberant anterior clypeal margins predominate). In full-face view, the broad head capsule of the minor worker of *M. pelorocephalus* is strongly expanded towards the mandibular insertions, giving it a strongly accentuated trapezoidal shape, and the basal margin of the major worker mandible is a carinate ledge set at 90° to the rest of the mandible throughout its length, with the (slightly) offset basal tooth horizontal and the sub-basal tooth with a horizontal and a vertical plane. A mandibular carina is present to varying degrees in media and minor workers. All workers have a glabrous mesosoma. These characters will distinguish *M. pelorocephalus* from all other *Melophorus* except for its close relation, *M. laticeps*. The heads of minor, media and small major workers of *M. pelorocephalus*, however, are even more exaggeratedly trapezoidal than are the heads of *M. laticeps* workers and the upper head of this species is distinctly darker in colour than the lower head and the mesosoma (all are uniformly coloured in *M. laticeps*). *Melophorus pelorocephalus* is believed to have a large major worker, but only minor, media and small major workers have been seen in the collections examined.

#### Minor worker description.

**Head.** Head square or rectangular, tending to trapezoid; posterior margin of head strongly convex; frons matt or with weak sheen, shagreenate; pilosity of frons a mixture of a few well-spaced, erect setae interspersed with appressed setae only. Eye moderate (eye length 0.20–0.49 length of side of head capsule); in full-face view, eyes set above midpoint of head capsule; in profile, eye set anteriad of midline of head capsule; roughly ovoid, eye narrowed posteriad. In full-face view, frontal carinae straight or weakly convex; frontal lobes curved toward antennal insertion. Anteromedial clypeal margin straight; clypeal psammophore set at or above midpoint of clypeus; palp formula 6,4. Five mandibular teeth in minor worker; mandibles triangular, weakly incurved; third mandibular tooth distinctly shorter than apical tooth and teeth numbers two and four; masticatory margin of mandibles approximately vertical or weakly oblique. **Mesosoma.** Integument of pronotum, mesonotum and mesopleuron moderately shining and shagreenate throughout; anterior mesosoma in profile broadly convex; erect pronotal setae absent; in profile, metanotal groove shallow, broadly V or U-shaped; propodeum matt or with weak sheen and finely striolate; propodeum smoothly rounded or with indistinct angle; propodeal dorsum and declivity confluent; erect propodeal setae always absent; appressed propodeal setulae short, separated by more than own length and inconspicuous; propodeal spiracle situated nearer to midpoint of propodeum than to its declivitous face, and shorter (length < than 0.50 × height of propodeum). **Petiole.** In profile, petiolar node narrowly conical, vertex blunt, directed posteriad; in full-face view, shape of petiolar node tapered with blunt vertex; node shining and smooth with vestigial sculpture. **Gaster.** Gaster weakly shining with indistinct shagreenation; pilosity of first gastral tergite consisting of well-spaced short, inconspicuous, appressed setae, erect setae (present in at least some workers) confined to margin of sclerite. **General characters.** Colour of foreparts tan, head darker, legs and gaster dark chocolate.

#### Major worker description.

**Head.** Head horizontally rectangular, broader than wide; posterior margin of head weakly convex; cuticle of frons shining with superficial shagreenation and microreticulation only; consisting exclusively or almost exclusively of well-spaced, appressed setae only; Eye small (eye length less than 0.2 × length of head capsule); in full-face view, eyes set above midpoint of head capsule; in profile, eye set anteriad of midline of head capsule; eyes elliptical. In full-face view, frontal carinae straight or weakly convex; frontal lobes straight in front of antennal insertion. Anterior clypeal margin straight; clypeal psammophore set at or above midpoint of clypeus; palp formula 6,4. Mandibular teeth in major worker 4; mandibles strongly incurved, apical sector uniformly carinate and forming a bifurcate, horizontal ledge that terminates in the basal and pre-basal teeth; third mandibular tooth distinctly shorter than apical tooth, but equivalent in length to remaining teeth; masticatory margin of mandibles approximately aligned vertically or weakly oblique. **Mesosoma.** Integument of pronotum, mesonotum and mesopleuron moderately shining and shagreenate throughout; anterior mesosoma in profile broadly convex; erect pronotal setae absent; in profile, metanotal groove a weak furrow; integument of propodeum shining and finely striolate and microreticulate; propodeum with indistinct angle; length ratio of propodeal dorsum to its declivity not applicable, propodeal dorsum and declivity confluent; erect propodeal setae absent; appressed propodeal setae short, separated by more than own length and inconspicuous; propodeal spiracle situated at least twice its length from the declivitous face of the propodeum, and shorter (length less than 0.50 × height of propodeum). **Petiole.** In profile, petiolar node narrowly conical, vertex sharply defined; in full-face view, shape of petiolar node tapered with blunt vertex; node shining and smooth throughout; in full-face view, shape of petiolar node uniformly rounded; node shining and faintly striolate and microreticulate **Gaster.** Gaster shining, shagreenate (‘LP record’ appearance); pilosity of first gastral tergite consisting of well-spaced, short, inconspicuous, appressed setae only, erect setae absent. **General characters.** Colour reddish-brown, gaster black.

#### Measurements.

Worker (n = 4): CI 121–133; EI 19–26; EL 0.24–0.28; HL 0.76–1.13; HW 0.92–1.49; ML 1.16–1.76; MTL 0.90–1.38; PpH 0.12–0.18; PpL 0.44–0.64; SI 88–101; SL 0.93–1.32.

#### Comments.

The morphology and genetic sequencing data confirm a sister relationship between this species and *Melophorus laticeps*, both ants being separated from other *M. wheeleri* complex members by a combination of the nature of the mandible in the major worker and the size of the workers. In full-face view, the anterior angles of the head capsule in *M. pelorocephalus* are more protruding than in its sister taxon, allowing for a very wide mandibular gape (bizarrely so in the minor worker, hence the name adopted here). The very gracile appearance of this ant and that of *M. laticeps* suggest these ants move very swiftly over the ground surface when they are active. No information on the habits of the species is available, but specimens have been collected on red sand covered by spinifex and low shrubs in the Little Sandy Desert, WA. Elsewhere, this ant has been collected in the Pilbara region, WA and at Three Forges Waterhole in SA (SAM).

#### Etymology.

Greek *peloros* (‘monstrous’) plus Latinized Greek *kephalos* (‘head’); noun in the nominative singular standing in apposition to the generic name.

**Figure 84.**
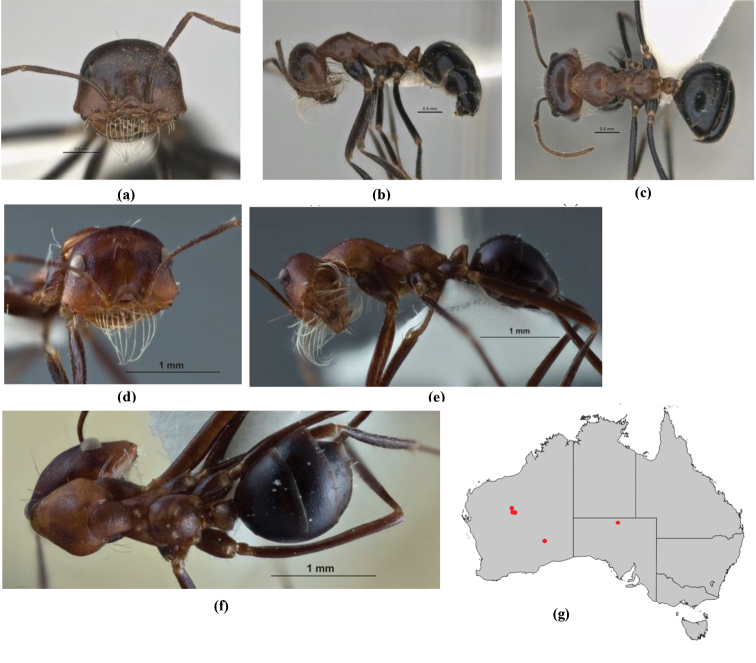
*Melophorus pelorocephalus* sp. n.: major worker paratype (JDM32-004898–bottom ant) frons (**a**), profile (**b**) and dorsum (**c**); minor worker holotype (JDM32-004714–top ant) frons (**d**), profile (**e**) and dorsum (**f**); distribution map for the species (**g**). Low resolution scale bars: 1 mm (**a–c**).

### Melophorus prominens

Taxon classificationAnimaliaHymenopteraFormicidae

Heterick, Castalanelli & Shattuck sp. n.

http://zoobank.org/7DAF10D4-A7BC-429A-9C3C-BB081F86C4D5

#### Types.

Holotype minor worker (bottom ant) Coolawanyah Station, Western Australia, 1953, K.C. Buller, ANIC ANTS VIAL 27.89 [ANIC32-900142] (ANIC). Paratypes: 2 major workers on the same pin and with the same details as the holotype (ANIC); 2 major workers and media worker from 28 miles SE of Roebourne, Western Australia, 21 April 1963, McInnes & Dowse, Central & NW Aust. 1963, Series A276 (ANIC); 2 major workers and minor worker from 18 miles ESE of Roebourne, Western Australia, 20 April 1963, McInnes & Dowse, 29170, Central & N.W. Aust. 1963, Series A273 (BMNH); 2 major workers from 15 miles S of Roebourne, Western Australia, 24 June 1967, G. Campbell, on grass flats carrying grass seeds (MCZ); 2 minor workers from 6 km N of Yalgoo, 19 March 1987, B. Heterick, 168, 8*Mel*BH28 [JDM32-001868] (WAM).

#### Diagnosis.

*Melophorus prominens* can be placed in the *M. biroi* species-group on the basis of characters of the clypeus, propodeum, mandible and palps. The species is also placed in the *M. wheeleri* species-complex because it agrees with the following apomorphies possessed by the complex: the minor worker often has more than five teeth, the largest major worker has a short, massive, elbowed mandible directed posteriad; in profile, the maxillary palps are short in the major and generally short in minor workers (in the minor worker, usually only attaining the neck sclerite at their maximum extent when the head is moderately inclined) and, in full-face view, the anterior margin of the clypeus in the large major worker is usually planar or weakly concave (variable in other subcastes but planar or narrowly protuberant anterior clypeal margins predominate). The broadly angulate projection of the clypeus in major and media workers automatically identifies them as belonging to this species. The minor worker is less distinctive, but is large (HW nearly 1 mm), has a convex, protrusive clypeus with a stout psammophore near the anterior margin, a minutely striate mandible and six or more teeth on the masticatory margin. These features separate it from numerous, smaller minor workers in the same species-complex. The *M. prominens* minor worker is most easily confused with the minor worker of *M. wheeleri* but is quite hairy and has erect setae on the mesosoma, including more than a dozen on the propodeum.

#### Minor worker description.

**Head.** Head rectangular; posterior margin of head weakly concave; frons matt or with weak sheen, shagreenate; pilosity of frons a mixture of a few well-spaced, erect setae interspersed with appressed setae only. Eye moderate (eye length 0.20–0.49 length of side of head capsule); in full-face view, eyes set above midpoint of head capsule; in profile, eye set anteriad of midline of head capsule; eyes elliptical or slightly reniform. In full-face view, frontal carinae straight or weakly convex; frontal lobes curved inward in front of antennal insertion. Anteromedial clypeal margin broadly and evenly convex and protrusive; clypeal psammophore set at or just above anterior clypeal margin; palp formula 3,4. Mandibular teeth in minor worker six to nine; mandibles triangular, weakly incurved; third mandibular tooth may be separated from tooth no. 3 and tooth no. 4 by one or more intercalary teeth, size appears to vary; masticatory margin of mandibles approximately vertical or weakly oblique. **Mesosoma. **Integument of pronotum, mesonotum and mesopleuron with weak to moderate sheen, shagreenate on pronotum and dorsum of mesonotum, otherwise microreticulate; anterior mesosoma in profile weakly elevated anteriad, thereafter gently sinuate, pronotum and mesonotum on same plane; appearance of erect pronotal setae long (i.e., longest erect setae longer than length of eye) and unmodified; in profile, metanotal groove shallow, broadly V or U-shaped; propodeum shining and microreticulate; propodeum smoothly rounded or with indistinct angle; propodeal dorsum and declivity confluent; erect propodeal setae present and abundant (greater than 12); appressed propodeal setulae long and separated by at least own length; propodeal spiracle situated on or beside declivitous face of propodeum, and shorter (length < 0.50 × height of propodeum). **Petiole.** In profile, petiolar node squamiform; in full-face view, shape of petiolar node uniformly rounded, or tapered with blunt vertex; node shining and distinctly microreticulate. **Gaster.** Gaster shining, shagreenate (‘LP record’ appearance); pilosity of first gastral tergite consisting of well-spaced, erect and semi-erect setae interspersed with regularly placed appressed setae. **General characters.** Colour concolorous chocolate.

#### Major worker description.

**Head.** Head quadrate (i.e., heart-shaped); posterior margin of head weakly concave; cuticle of frons shining with superficial shagreenation or microreticulation only; pilosity of frons a mixture of a few well-spaced, erect setae interspersed with appressed setae only. Eye small (eye length less than 0.2 × length of head capsule); in full-face view, eyes set above midpoint of head capsule; in profile, eye set anteriad of midline of head capsule; eyes elliptical. In full-face view, frontal carinae straight, divergent posteriad; frontal lobes curved inward in front of antennal insertion. Anterior clypeal margin convex, acuminate anteromedially, with anteromedial projection protruding at angle of 90° to clypeus; clypeal psammophore set at or just above anterior clypeal margin; palp formula 6,4. Mandibular teeth in major worker effectively 3, basal tooth reduced to angle only; mandibles strongly incurved, apical sector weakly carinate or incompletely carinate; third mandibular tooth larger than tooth no.2 (basal tooth represented by an angle or small denticle); masticatory margin of mandibles approximately aligned vertically or weakly oblique. **Mesosoma.** Integument of pronotum, mesonotum and mesopleuron shining with very superficial microreticulation, entire lower mesopleuron distinctly shagreenate; anterior mesosoma in profile broadly convex; erect pronotal setae long (i.e., longer than length of eye) and unmodified; in profile, metanotal groove shallow, broadly V- or U-shaped; propodeum shining and microreticulate; propodeum angulate, propodeal angle blunt; length ratio of propodeal dorsum to its declivity about 4:3; erect propodeal setae present and abundant (at least a dozen); appressed propodeal setae long, each reaching setae behind and in front, but not forming pubescence; propodeal spiracle situated at least twice its width from the declivitous face of propodeum, and shorter (length less than 0.50 × height of propodeum). **Petiole.** In profile, petiolar node squamiform; in full-face view, shape of petiolar node tapered with median indentation; node shining and faintly shagreenate-microreticulate. **Gaster.** Gaster shining, shagreenate (‘LP record’ appearance); pilosity of first gastral tergite consisting of well-spaced, erect and semi-erect setae interspersed with regularly spaced appressed setae. **General characters.** Colour concolorous dark chocolate.

#### Measurements.

Worker (n = 4): CI 108–125; EI 13–25; EL 0.24–0.47; HL 0.91–2.81; HW 0.98–3.52; ML 1.22–2.70; MTL 0.90–2.04; PpH 0.13–0.38; PpL 0.46–1.11; SI 50–91; SL 0.89–1.76.

#### Comments.

The *Melophorus prominens* major worker is rendered distinctive by an anteromedian clypeal prominence that is directed outwards at a 90° angle to the clypeus. The minor worker lacks this feature and is more difficult to identify; however, it has a convex anterior clypeal margin and is relatively hairy, enabling it to be distinguished from similar minor workers in the *M. wheeleri* cluster. All definitive collections of this ant have been taken in the mid-west or Pilbara regions of WA, but a pin of material taken from 30 km S of Strathburn Qld (TERC) may also belong to this species. No specimens of this ant have been sequenced and label records provide no additional data, but the species is likely to be granivorous, based on its undoubted affinities with *M. wheeleri*.

#### Etymology.

Latin *prominens* (‘projection’); participle in the nominative singular.

**Figure 85.**
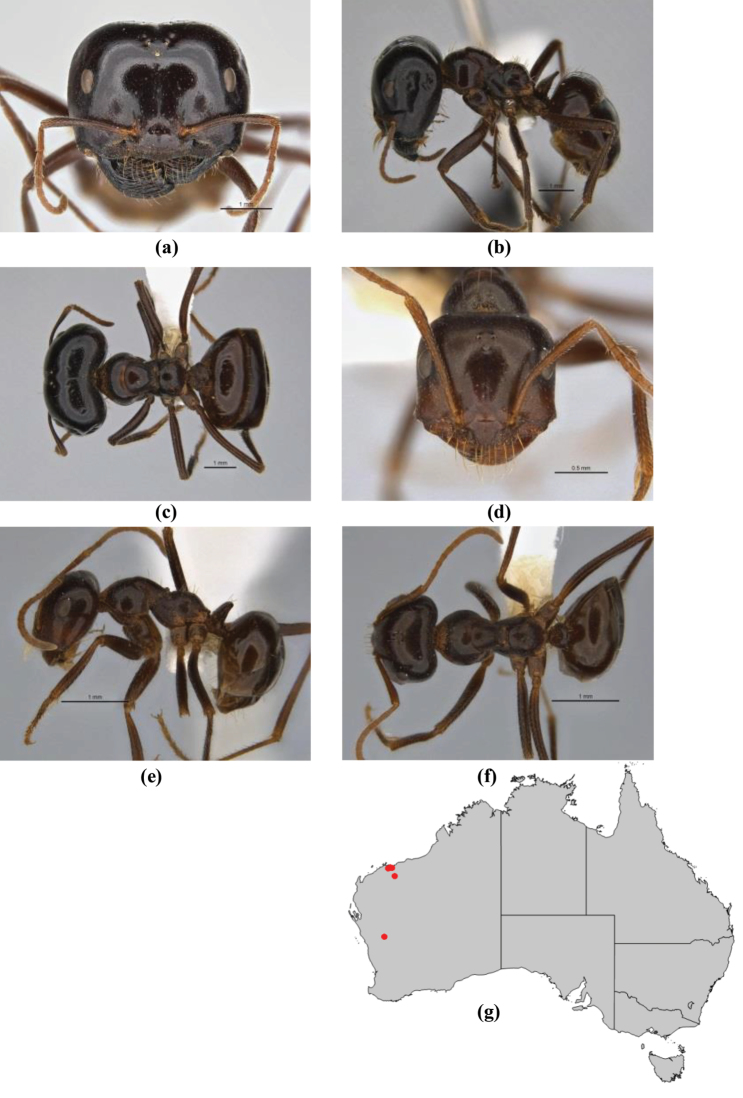
*Melophorus prominens* sp. n.: major worker paratype (ANIC32-900142–top ant) frons (**a**), profile (**b**) and dorsum (**c**); minor worker holotype (ANIC32-900142–bottom ant) frons (**d**), profile (**e**) and dorsum (**f**); distribution map for the species (**g**). Low resolution scale bars: 1 mm (**a–c, e, f**); 0.5 mm (**d**).

### Melophorus purpureus

Taxon classificationAnimaliaHymenopteraFormicidae

Heterick, Castalanelli & Shattuck sp. n.

http://zoobank.org/7CBC0ADE-FBF6-4152-B8DE-EB9195C01244

#### Types.

Holotype minor worker (middle ant) from Kadji Lake Rd, 29°07'S, 116°09'E, Western Australia, 28 January 1999, Heterick, B.E. Nest series 5 [JDM32-001885] (WAM). Paratypes: major and minor worker on same pin and with same details as holotype (WAM); 2 majors and a minor worker from Kadji Lake Rd, 29°07'S, 116°09'E, Western Australia, 28 January 1999, Heterick, B.E. Nest series 4 [ANIC32-001884] (ANIC); major and 2 minor workers from Kadji Lake Rd, 29°07'S, 116°09'E, Western Australia, 28 January 1999, Heterick, B.E. Nest series 3 [ANIC32-001883] (WAM).

#### Other material examined.

**Western Australia**: Mettler Lake Rd (Heterick, B.E. [M163]), Sappers Rd (Heterick, B.E. [M156/M157/M158/M159/M161/M162]).

#### Diagnosis.

*Melophorus purpureus* can be placed in the *M. biroi* species-group on the basis of characters of the clypeus, propodeum, mandible and palps. The species is also placed in the *M. wheeleri* species-complex because it agrees with the following apomorphies possessed by the complex: the minor worker often has more than five teeth, the largest major worker has a short, massive, elbowed mandible directed posteriad; in profile, the maxillary palps are short in the major and generally short in minor workers (in the minor worker, usually only attaining the neck sclerite at their maximum extent when the head is moderately inclined) and, in full-face view, the anterior margin of the clypeus in the large major worker is usually planar or weakly concave (variable in other subcastes but planar or narrowly protuberant anterior clypeal margins predominate). The combination of an evenly convex head capsule (when seen in full-face view), a clypeus that is not folded back, the absence of short, inconspicuous setae on the gaster and an evenly convex pronotum plus mesonotum when seen in profile serve to separate *M. purpureus* from most other members of the *M. wheeleri* complex. The minor worker bears a passing resemblance to that of *M. chauliodon* but has a spheroidal, not elongate, eye and the minor and media workers often possess two or more bristly setae on the mesosoma (mainly on the pronotum), whereas the *Melophorus chauliodon* minor and media workers are invariably glabrous. The major and media workers of *M. purpureus* are readily separable from their counterparts in *M. chauliodon* in not having an offset tusk-like tooth on the mandible. *Melophorus purpureus* also resembles *M. hexidens*, but that species seems to be endemic to NSW, and the clypeus is dfferent.

#### Minor worker description.

**Head.** Head square; posterior margin of head planar or weakly convex; frons shining with superficial shagreenation or microreticulation only; pilosity of frons a mixture of a few well-spaced, erect setae interspersed with appressed setae only. Eye moderate (eye length 0.20–0.49 length of side of head capsule); in full-face view, eyes set above midpoint of head capsule; in profile, eye set anteriad of midline of head capsule; roughly ovoid, eye narrowed posteriad. In full-face view, frontal carinae distinctly concave; frontal lobes straight in front of antennal insertion. Anteromedial clypeal margin broadly emarginate with projecting anteromedial dimple; clypeal psammophore set at or above midpoint of clypeus; palp formula 6,4. Five to six mandibular teeth in minor worker; mandibles triangular, weakly incurved; third mandibular tooth distinctly shorter than apical tooth and teeth numbers two and four; masticatory margin of mandibles approximately vertical or weakly oblique. **Mesosoma.** Integument of pronotum, mesonotum and mesopleuron shining and microreticulate, microreticulation reduced on humeri; anterior mesosoma in profile convex anteriad, mesonotum often slightly overlapping pronotum, mesosoma planar or slightly sinuate posteriad; appearance of erect pronotal setae short and spinous; in profile, metanotal groove shallow, broadly V or U-shaped; propodeum shining and microreticulate; propodeum smoothly rounded or with indistinct angle; propodeal dorsum and declivity confluent; erect propodeal setae always absent; appressed propodeal setulae short, separated by more than own length and inconspicuous; propodeal spiracle situated on or beside declivitous face of propodeum, and shorter (length < 0.50 × height of propodeum). **Petiole.** In profile, petiolar node squamiform; in full-face view, shape of petiolar node tapered with blunt vertex, or tapered with squared-off vertex; node shining and distinctly shagreenate-microreticulate. **Gaster.** Gaster shining, shagreenate (‘LP record’ appearance); pilosity of first gastral tergite consisting of well-spaced, erect and semi-erect setae interspersed with regularly placed appressed setae. **General characters.** Colour brownish-black, legs brown, tending yellowish distally.

#### Major worker description.

**Head.** Head horizontally rectangular, broader than wide; posterior margin of head weakly concave; cuticle of frons shining with superficial shagreenation or microreticulation only; pilosity of frons a mixture of a few well-spaced, erect setae interspersed with appressed setae only. Eye small (eye length less than 0.2 × length of head capsule); in full-face view, eyes set above midpoint of head capsule, or set at about midpoint of head capsule; in profile, eye set anteriad of midline of head capsule; eyes elliptical, or roughly ovoid, eye narrowed posteriad. In full-face view, frontal carinae straight or weakly convex, or straight, divergent posteriad; frontal lobes straight in front of antennal insertion. Anterior clypeal margin broadly concave, or broadly emarginate; clypeal psammophore set at or above midpoint of clypeus; palp formula 6,4. Five mandibular teeth in major worker; mandibles strongly incurved, apical sector weakly carinate or incompletely carinate; third mandibular tooth distinctly shorter than apical tooth, but equivalent in length to remaining teeth; masticatory margin of mandibles approximately aligned vertically or weakly oblique. **Mesosoma.** Integument of pronotum, mesonotum and mesopleuron shining with indistinct microsculpture that is most pronounced on lower surfaces; anterior mesosoma in profile gently sinuous after initial steep pronotal incline; erect pronotal setae short, (i.e., shorter than length of eye) and unmodified; in profile, metanotal groove shallow, broadly V- or U-shaped; propodeum shining and shagreenate; propodeum angulate, propodeal angle blunt; length ratio of propodeal dorsum to its declivity between 1:1 and 1:2; erect propodeal setae variable in number, may be absent; appressed propodeal setae short, separated by more than own length and inconspicuous; propodeal spiracle situated at least twice its width from the declivitous face of propodeum, and shorter (length less than 0.50 × height of propodeum), or situated on or beside declivitous face of propodeum, and shorter (length less than 0.50 × height of propodeum). **Petiole.** In profile, petiolar node squamiform; in full-face view, shape of petiolar node uniformly rounded, or tapered with blunt vertex, or tapered with squared-off vertex; node shining and faintly shagreenate-microreticulate. **Gaster.** Gaster shining, shagreenate (‘LP record’ appearance); pilosity of first gastral tergite consisting of well-spaced, erect and semi-erect setae interspersed with regularly spaced appressed setae. **General characters.** Colour body brownish-black, sometimes with dark reddish tints, legs variably brown and yellowish-brown.

#### Measurements.

Worker (n = 4): CI 113–126; EI 14–22; EL 0.21–0.38; HL 0.84–2.10; HW 0.95–2.65; ML 1.15–2.12; MTL 0.85–1.39; PpH 0.11–0.23; PpL 0.45–0.86; SI 51–100; SL 0.95–1.34.

#### Comments.

This West Australian species needs additional material to be successfully delimited: currently, the populations included under this name show a lot of variation. Populations in the mid-west are black, with sparse erect setae in all workers, but material from the deep southwest and south coast tends to reddish and minor workers may be glabrous. Sequenced material from Sappers Road near Lancelin, WA reveals a sister or near sister relationship with *M. wheeleri*, which it closely resembles, in a five-gene tree, and it is sister to *M. wheeleri* and *M. marmar* in a three-gene tree. The species, as currently constituted, can be separated from very similar ants by a combination of the conformation of the mandible (especially in the major worker), the shape of the eye and the appearance of the clypeus. In the field, minor workers, especially those of the paler southern form, may be mistaken for *M. turneri*. The principal author of this work has collected the dark form of the ant near salt lakes (Kadji Lake) and the southern form in light soil in heathland (Sappers Rd). Based on its morphology and genetic affinities, this species may be granivorous.

#### Etymology.

Latin *purpureus* (‘purple’); adjective in the nominative singular.

**Figure 86.**
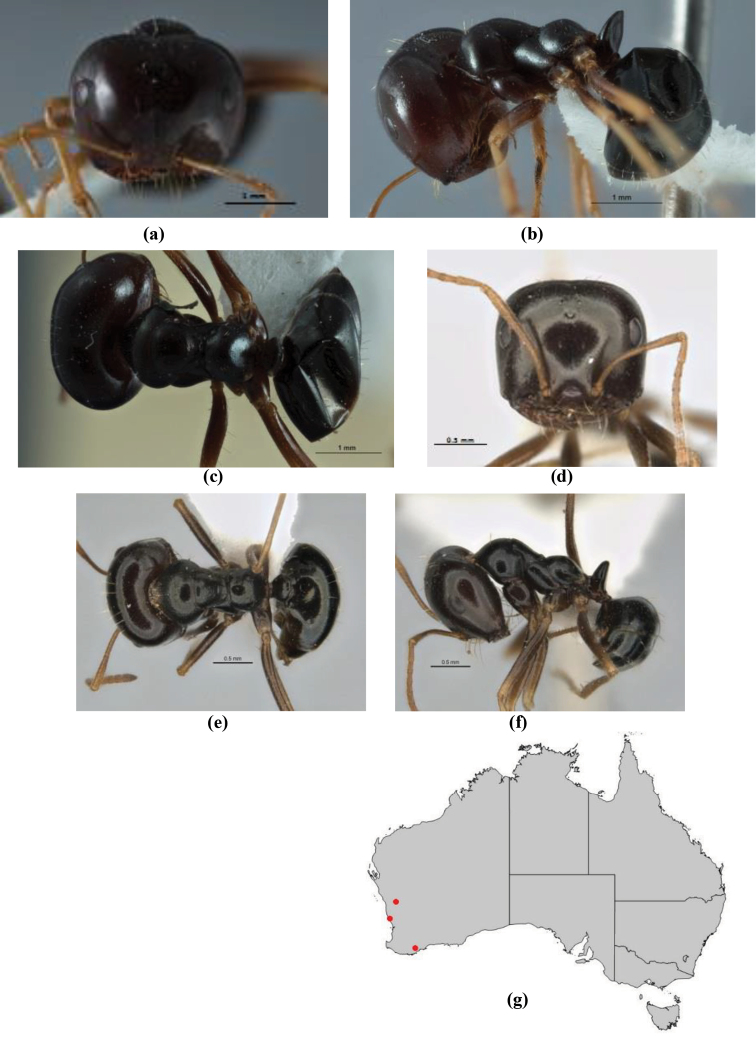
*Melophorus purpureus* sp. n.: major worker paratype (JDM32-001885–top ant) frons (**a**), profile (**b**) and dorsum (**c**); minor worker holotype (JDM32-001885–middle ant) frons (**d**), profile (**e**) and dorsum (**f**); distribution map for the species (**g**). Low resolution scale bars: 1 mm (**a–c**); 0.5 mm (**d–f**).

### Melophorus wheeleri

Taxon classificationAnimaliaHymenopteraFormicidae

Forel

Melophorus wheeleri Forel 1910: 60 (combination in M. (Erimelophorus) by Wheeler 1935: 71). Viehmeyer 1914: 43 (q.). See also: Forel 1915: 88. Types. Syntype (probable) major and minor workers Tennants [*sic*] Creek, Northern Territory [ANIC, BMNH, MHNG] (examined: ANIC specimens ANIC32-053440, AntWeb images of BMNH specimen BMNH(E)1016285, CASENT0903262 and MHNG specimens CASENT0909826, CASENT0909827). Also seen: three pins (1 × dealated queen, male and badly damaged minor (gaster, node and legs only), 1 × winged queen and two media workers and 1 × major, media and minor worker), Tennant Creek, Northern Territory (SAM). All three pins carry co-type labels and locality labels that appear to be written by another hand, and the syntype status of these nine specimens is thus brought into question.
Melophorus omniparens Forel
Melophorus omniparens Forel 1915: 85 (combination in M. (Erimelophorus) by Wheeler 1935: 71). Types. Syntype media and minor worker, Alice River, Queensland [MHNG] (examined: MHNG specimens). **Syn. n.**

#### Other material examined.

**Northern Territory**: 102 km N Yuendumu (Greenslade, P.J.M.), 11 km N Tennant Creek (Davidson, D. & Morton, S.), 11 mi SE Papunya Native Settlement (McInnes & Dowse), 16 mi N Mt Wedge Homestead (McInnes & Dowse), 17 km S Rabbit Flat (Greenslade, P.J.M.), 17 km S Rabbit Flat (Greenslade, P.J.M.), 20 km S Alice Springs (Greenslade, P.J.M.), 25 km N Alice Springs (Shattuck, S.O.), 35 mi E Sandy Blight Junction (McInnes & Dowse), 36 mi NW Tanami (McInnes & Dowse), 39 km NE Andado Homestead (Feehan, J.E.), 40 km W Wave Hill (Davidson, D. & Morton, S.), 44 km W Cloncurry (Shattuck, S.O.), 48 mi W Papunya Native Settlement (McInnes & Dowse), 50 km N Old Andado Homestead (Feehan, J.E.), 50 km WNW Hermansburg (Shattuck, S.O.), 5 km NE Barrow Creek (Davidson, D. & Morton, S.), 6 km W Bunda Stn turnoff (Heterick, B.E. [M228]), about 56 km N Kulgera (Feehan, J.E.), about 9 km E Finke (Feehan, J.E. [ANIC32-900185]), Atartinga, NW Alice Springs (Greenslade, P.J.M.), Johnstons Lagoon (Greaves, T. [ANIC32-900063]), Kunoth Paddock, near Alice Springs (Greenslade, P.J.M.), Kunoth Paddock, near Alice Springs (Greenslade, P.J.M.), Kunoth Paddock, near Alice Springs (Greenslade, P.J.M.), Kunoth Paddock, near Alice Springs (Greenslade, P.J.M.), Kunoth Paddock, near Alice Springs (Greenslade, P.J.M. [ANIC32-900064]), Kunoth Paddock, near Alice Springs (Greenslade, P.J.M.), Kunoth Paddock, near Alice Springs (Greenslade, P.J.M.), Kunoth Paddock, near Alice Springs (Greenslade, P.J.M.), Mulga (Greaves, T.), Simpson Gap (Feehan, J.E.), Tanami Desert (Greenslade, P.J.M.), Tanami Desert (Greenslade, P.J.M.), Tanami Desert (Greenslade, P.J.M.), Tennant Creek (Forel), Tennant Creek (Field, J.F.), Tennant Creek (Froggett, W.W. [ANIC32-053443]). **Queensland**: ‘Gumbardo’ (Beutel, T.), ‘Merigol’ (Beutel, T.), 11 mi E Cloncurry (Dowse, J.E.), 40 km E Cameron Corner (Greenslade, P.J.M.), 50 mi N Goondiwindi (Dowse, J.E.), 5 mi W Lotus Vale Homestead, N Normanton (Dowse, J.E.), Blair Athol Mine (Houston, W. [ANIC32-040323]), Camooweal Caves, 14 km SSE Camooweal (Shattuck, S.O. [ANIC32-039871]), Mareeba (Hill, H.E.), Tindaree, Hannaford Rd South via Tara (House, A./Brown, S.). **South Australia**: 1:2500 Gason Ya 636549 (Greenslade, P.J.M.), 15 km ENE Beltana (Greenslade, P.J.M.), 15 km NE Bryan (Greenslade, P.J.M.), 22 km NW Kimba (Greenslade, P.J.M.), 30 km S Granite Downs (Greenslade, P.J.M.), 5 mi ENE Watson (McInnes & Dowse), 7 km NW Morgan (Greenslade, P.J.M.), 7 km NW Morgan (Greenslade, P.J.M.), 7 km NW Morgan (Greenslade, P.J.M.), 7 km NW Morgan (Greenslade, P.J.M.), 8 mi ENE Ernabella Mission (McInnes & Dowse), 30 mi WSW Ernabella Mission (McInnes & Dowse), 90 km N Granite Downs (Greenslade, P.J.M.), about 25 km W Marree (Feehan, J.E.), about 33 km SE Oodnadatta (Feehan, J.E.), Buckleboo Homestead, N Eyre Peninsula (Greenslade, P.J.M.), Cooper Creek, 13 km NEbyN Etadunna Homestead (Feehan, J.E.), Emu Camp, Victoria Desert (Greenslade, P.J.M.), Erly Wanyawanya Roadhouse, Musgrove Range (McInnes & Dowse), Innamincka (Forrest, J.), Koonamore (Greenslade, P.J.M.), Koonamore (Greenslade, P.J.M.), Mundoora National Park (Greenslade, P.J.M.), Oraparinna, Flinders Ranges (Greenslade, P.J.M.), Woomera (Greenslade, P.J.M.). **Western Australia**: 0.9 km along Tanami Rd (Heterick, B.E. [M204/M205]), 10 km E Nicholson Rd turnoff (Heterick, B.E. [M229]), 11 mi E Mt. Aloysius (McInnes & Dowse), 15 mi NW Mt Squires, Barrow Range (McInnes, R. & Dowse, J.), 16 mi NNE Hawks Nest (McInnes & Dowse), 18 mi NW Mt Davies, Tomkinson Range (McInnes, R. & Dowse, J.), 18 mi WSW Roy Hill Homestead (McInnes & Dowse), 2.4 km along Tanami Rd (Heterick, B.E. [M209]), 20 mi NE Wongawol Homestead (McInnes & Dowse), 20 mi NW Leonora (McInnes & Dowse), 21 mi NW Wittenoon (McInnes & Dowse), 22 mi W Carnegie Homestead (McInnes & Dowse), 26 mi E Giles (McInnes & Dowse), 34 km NNW of Cowra LC (CALM Pilbara Survey [JDM32-001887]), 27 mi WNW Coolawanyah Homestead, NW Wittenoom (McInnes & Dowse), 28 km ESE Warburton (Feehan, J.E.), 28 km WbyS Docker River (Northern Territory) (Feehan, J.E.), 37 mi SW Mundiwindi (McInnes & Dowse), 41 mi NNE Giles (McInnes & Dowse), 43 mi ENE Meekatharra (McInnes & Dowse), 43 mi ENE Peak Hill (McInnes & Dowse), 43 mi SSE Wittenoon (McInnes & Dowse), 43 mi WNW Wiluna (McInnes & Dowse), 4 mi E Blackstone Camp, Blackstone Range (McInnes & Dowse), 52 mi W Roy Hill Homestead (McInnes & Dowse), 60 km W Halls Creek (Heterick, B.E. [M234]), 6 mi SW Roy Hill Homestead (McInnes & Dowse), 7 km S Cue (Heterick, B.E. [M286]), 74 km E Halls Creek (Heterick, B.E. [M230]), 8 km N Auski RH (Heterick, B.E. [M272/M273]), 8 km N Auski RH (Heterick, B.E. [M271]), 9 mi E Cardawan Homestead, SSW Mundiwindi (McInnes & Dowse), Argyle Diamonds via Kununurra (Postle, A.T. [JDM32-001872]), Beegull Waterhole, 132 km EbyN Cosmo Newberry (Feehan, J.E.), Broome (Baesjou, A.E.), Elvire Stn, Halls Creek (Gosnell, W. C.), Ethel Creek (Varris, P.A. [JDM32-001870]), G[ascoyne]. J[unction]. Rd, 108 km E Carnarvon (Heterick, B.E. [M307/M309]), Hines Hill (Heterick, B.E. [JDM32-001871]), Jigalong Stn. (Graham, R. [JDM32-001869]), Meekatharra (Lowery, B.B.), Port Hedland (Heterick, B.E. [M269]), South Hedland (Heterick, B.E. [JDM32-001867]), Tardun (Mercovich, C.), Tunnel Creek National Park (Ward, P.S.), Warburton (Philpott, C.), Without Locality (Humphreys, W.F., et.al. [JDM32-001873]), Yoothepina [Yoothapina] (Hides, D. [JDM32-001874]).

#### Diagnosis.

*Melophorus wheeleri* can be placed in the *M. biroi* species-group on the basis of characters of the clypeus, propodeum, mandible and palps. The species is also placed in the *M. wheeleri* species-complex because it agrees with the following apomorphies possessed by the complex: the minor worker often has more than five teeth, the largest major worker has a short, massive, elbowed mandible directed posteriad; in profile, the maxillary palps are short in the major and generally short in minor workers (in the minor worker, usually only attaining the neck sclerite at their maximum extent when the head is moderately inclined) and, in full-face view, the anterior margin of the clypeus in the large major worker is usually planar or weakly concave (variable in other subcastes but planar or narrowly protuberant anterior clypeal margins predominate). *Melophorus wheeleri* shares only with *M. diversus* the following combination of apomorphies: the anterior sector of the clypeus of minor worker is strongly folded back towards the mandible, and the clypeal psammophore is placed on a distinct ledge that may be carinate, the minor worker mandible has 5-9 teeth and denticles, the head, mesosoma and gaster of all workers have short, inconspicuous appressed setae that are usually separated by more than their own length (if more elongate, as in some small minor workers, then the ant is glossy and weakly sculptured) and the media and major workers are quite large *Melophorus* (HW of the large major worker ≥ 2.60 mm). Most workers of *Melophorus wheeleri* can be distinguished from *M. diversus* workers in having the head of minor worker always with some sculpture, the head being matt to moderately shining in appearance, erect setae are almost always lacking on the pronotum and the first gastral tergite, and the eye of the minor worker is usually less than 0.30× length of side of head capsule. The major worker is similar to the major worker of *M. diversus* but either has a glabrous mesosoma or possesses short, bristly setae on the mesosoma and is commonly matt in appearance (WA, NT) but may be glossy (eastern states). (In *M. diversus* the head of the minor worker is smooth with just vestigial sculpture [the head often glossy], erect non-marginal setae are always present on the first gastral tergite with one or two small erect setae also present on the pronotum of some individuals, the eye of the minor worker is large [0.30× length of side of head capsule] and the major worker usually has many fine, erect setae on the mesosoma [though these may be more sparse and bristly in a few individuals]). However, the distinctions between these two species are not always observed and a few individuals, possibly hybrids, may be unable to be placed in either taxon with confidence.

#### Minor worker description.

**Head.** Head square; posterior margin of head planar or weakly concave; frons matt or with weak sheen, microreticulate or microreticulate-shagreenate; frons consisting exclusively or almost exclusively of well-spaced, appressed setae only (small, erect setae, if present, usually confined to ocular triangle or posterior margin of head). Eye moderate (eye length 0.20–0.49 length of side of head capsule); in full-face view, eyes set above midpoint of head capsule; in profile, eye set anteriad of midline of head capsule; eyes elliptical or slightly reniform, or elongate. In full-face view, frontal carinae straight, divergent posteriad; frontal lobes straight in front of antennal insertion. Anteromedial clypeal margin straight, or broadly and evenly convex; clypeal psammophore set below midpoint of clypeus, or set at or just above anterior clypeal margin; palp formula 6,4. Five mandibular teeth in minor worker to nine; mandibles triangular, weakly incurved; third mandibular tooth may be separated from tooth no. 3 and tooth no. 4 by one or more intercalary teeth, size appears to vary; masticatory margin of mandibles approximately vertical or weakly oblique. **Mesosoma.** Integument of pronotum, mesonotum and mesopleuron moderately shining and shagreenate throughout; anterior mesosoma in profile broadly convex; erect pronotal setae absent; in profile, metanotal groove shallow, broadly V or U-shaped; propodeum shining and finely striolate and microreticulate; propodeum smoothly rounded or with indistinct angle, or angulate, propodeal angle blunt; length ratio of propodeal dorsum to its declivity about 1:1, or not applicable, propodeal dorsum and declivity confluent; erect propodeal setae always absent; appressed propodeal setulae short, separated by more than own length and inconspicuous; propodeal spiracle situated at least twice its width from the declivitous face of propodeum, and shorter (length < 0.50 × height of propodeum), or situated on or beside declivitous face of propodeum, and shorter (length < 0.50 × height of propodeum). **Petiole.** In profile, petiolar node squamiform; in full-face view, shape of petiolar node uniformly rounded; node shining and smooth with vestigial sculpture, or shining and distinctly shagreenate-microreticulate. **Gaster.** Gaster shining, shagreenate (‘LP record’ appearance); pilosity of first gastral tergite consisting of well-spaced short, inconspicuous, appressed setae, erect setae (present in at least some workers) confined to margin of sclerite. **General characters.** Colour reddish-brown with black gaster to concolorous blackish-brown.

#### Major worker description.

**Head.** Head quadrate (i.e., heart-shaped); posterior margin of head strongly concave; cuticle of frons shining and smooth except for piliferous pits, or shining with superficial shagreenation or microreticulation only, or matt or with weak sheen, microreticulate; frons consisting exclusively or almost exclusively of well-spaced, appressed setae only (small, erect setae, if present, usually confined to ocular triangle or posterior margin of head). Eye small (eye length less than 0.2 × length of head capsule); in full-face view, eyes set at about midpoint of head capsule; in profile, eye set anteriad of midline of head capsule; eyes elliptical. In full-face view, frontal carinae straight, divergent posteriad; frontal lobes curved toward antennal insertion. Anterior clypeal margin broadly emarginate; clypeal psammophore set at or above midpoint of clypeus; palp formula 6,4. Four mandibular teeth in major worker-5; mandibles strongly incurved, apical sector weakly carinate or incompletely carinate; third mandibular tooth distinctly shorter than apical tooth and teeth numbers two and four; masticatory margin of mandibles medially indented. **Mesosoma.** Integument of pronotum, mesonotum and mesopleuron shining with indistinct microsculpture that is most pronounced on lower surfaces; anterior mesosoma in profile broadly convex; erect pronotal setae short and unmodified, or weakly expanded distally; in profile, metanotal groove shallow, broadly V- or U-shaped; propodeum shining, with multiple hair like striolae; propodeum angulate, propodeal angle blunt; length ratio of propodeal dorsum to its declivity between1:1 and 1:2; erect propodeal setae variable in number, may be absent; appressed propodeal setae short, separated by more than own length and inconspicuous; propodeal spiracle situated on or beside declivitous face of propodeum, and shorter (length less than 0.50 × height of propodeum). **Petiole.** In profile, petiolar node squamiform; in full-face view, shape of petiolar node uniformly rounded, or generally rounded with median indentation, or tapered with squared-off vertex; node shining and faintly shagreenate-microreticulate. **Gaster.** Gaster shining, shagreenate (‘LP record’ appearance); pilosity of first gastral tergite consisting of well-spaced, erect and semi-erect setae interspersed with regularly spaced appressed setae. **General characters.** Colour of foreparts variably dark crimson or blackish-red (western form) to orange tan or vermillion (eastern form); gaster always dark brown to blackish-brown.

#### Measurements.

Worker (n = 8): CI 106-125; EI 11-31; EL 0.28-0.40; HL 0.69-2.85; HW 0.74-3.57; ML 0.87-2.54; MTL 0.55-1.49; PpH 0.09-0.30; PpL 0.35-1.11; SI 42-83; SL 0.61-1.51

#### Comments.

This extremely common *Melophorus* of arid and semi-arid areas can often be identified from its large nests, which typically are littered with small pebbles and plant remnants such as seed husks and stems (Plate). The species is ostensibly granivorous (so, Andersen, 2007), and workers are often seen transporting small seeds back to their nests. The ant can be identified and separated from near relatives by the glabrous first gastral tergite and smaller eye in the minor worker (less than 0.30× length of side of head capsule), features of the clypeus and body sculpture and the massive mandible of the major worker. Specimens from the ANIC Collection come from NT, QLD, SA and WA but this taxon seems to be replaced by *M. diversus* in NSW. However, as discussed above, differentiation between *M. diversus* and *M. wheeleri* is very difficult in some cases. All *M. wheeleri* sequenced belong to the more distinctive western form with its dark or even blackish-crimson colouring, and these specimens form a uniform cluster in the five-gene tree as a sister taxon to several other *M. wheeleri* complex species. In the three-gene tree, this species is sister to *M. marmar*. Unfortunately, the eastern form, which has a bright red or orange head in the major worker, was not represented in the sequencing.

*Melophorus omniparens* Forel, the type specimens of which come from Queensland, represents the glossy light-coloured form of the taxon. The two media workers syntypes seen have a sharply-defined edge to the anterior clypeal margin, which contrasts with the blunter, more carinate margin seen in the syntype workers of *Melophorus wheeleri*. Those latter workers, from Tennant Creek, NT, represent the darker, more matt western form of the ant. In the absence of evidence to the contrary (which may or may not come from sequencing of the eastern morphotype), these differences are here regarded as part of the intraspecific variation seen in the species, since the conformation of the clypeus is very variable across the broad range of the ant. *Melophorus omniparens* therefore becomes a junior synonym in this work.

Despite their ubiquity, label data for these ants are scanty, but they are able to tolerate disturbance provided their food source is nearby: ants were collected from a golf course at Meekatharra, WA. As mentioned above, small seeds seem to form their main diet: workers have been recorded carrying seeds of *Lepidium phlebopetalum* (Brassicaciae) at Jigalong Station, WA (Heterick 2009).

**Figure 87.**
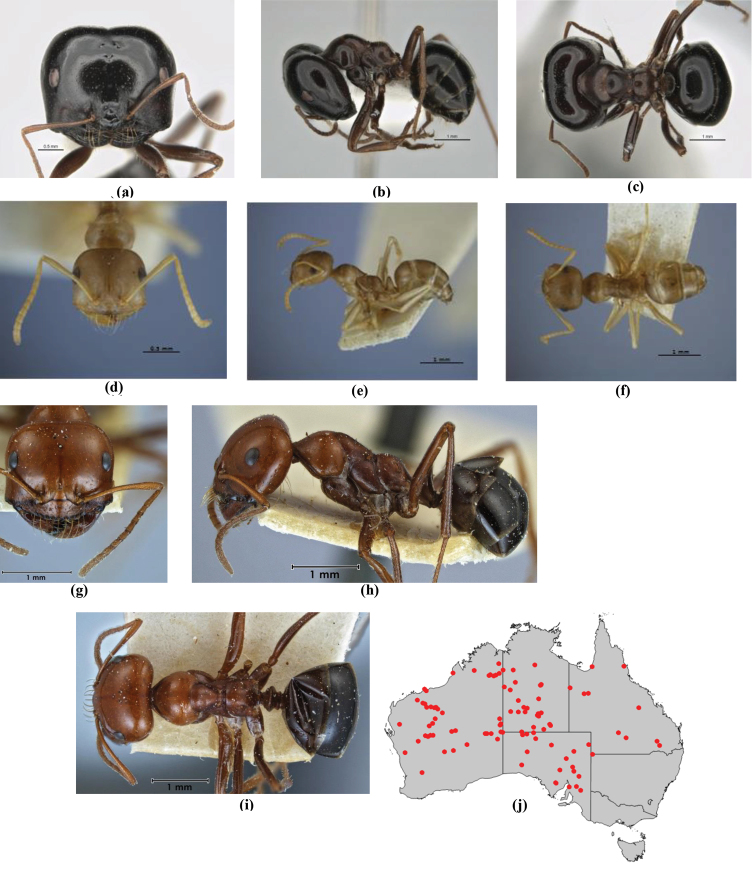
*Melophorus wheeleri* Forel: ‘*wheeleri*’ non–type major worker (JDM32-9001887) frons (**a**), profile (**b**) and dorsum (**c**); ‘*wheeleri*’ minor worker (MCZ: blanched syntype wrongly ascribed to *M. sulla* and part of that type series) (MCZC: Cotype 23030 [one of a pin of three ants]) frons (**d**), profile (**e**) and dorsum (**f**); MHNG ‘*omniparens*’ major worker syntype frons (**g**), profile (**h**) and dorsum (**i**); distribution map for the species (**j**). Low resolution scale bars: 1 mm (**b, c**); 0.5 mm (**a**).

### Melophorus xouthos

Taxon classificationAnimaliaHymenopteraFormicidae

Heterick, Castalanelli & Shattuck sp. n.

http://zoobank.org/7E10A14B-7534-471D-8479-9286082508A5

#### Types.

Holotype media worker from Little Sandy Desert 25°16'07"S, 120°27'33"E, Western Australia, 11-12 August 2012, N. A. Guthrie, dune crest: scattered shrubs and spinifex over red sand. Site 12.2, *Melophorus* revision sequenced specimen M138 [ANIC32-900211] (WAM). Paratypes: minor worker from 142.1 km SSE of Newman 24°31'53"S, 120°17'24"E, Western Australia, October 1996, S. van Leeuwen & R. N. Bromilow, Temp. invert. Pitfall trap, B3: Sand dune [JDM32-004852] (ANIC).

#### Other material examined.

**South Australia**: 26 km SSE Illintjitja (collector?? [SAMA database 32-005279]). **Western Australia**: 142.1 km SSE Newman (van Leeuwen, S. & Bromilow, R.N. [ANIC32-0900217]), Little Sandy Desert (Guthrie, N. A. [ANIC32-900211]), Little Sandy Desert (Guthrie, N. A. [ANIC32-900216]).

#### Diagnosis.

*Melophorus xouthos* can be placed in the *M. biroi* species-group on the basis of characters of the clypeus, propodeum, mandible and palps. The species is also placed in the *M. wheeleri* species-complex because it agrees with the following apomorphies possessed by the complex: the minor worker often has more than five teeth, the largest major worker has a short, massive, elbowed mandible directed posteriad; in profile, the maxillary palps are short in the major and generally short in minor workers (in the minor worker, usually only attaining the neck sclerite at their maximum extent when the head is moderately inclined) and, in full-face view, the anterior margin of the clypeus in the large major worker is usually planar or weakly concave (variable in other subcastes but planar or narrowly protuberant anterior clypeal margins predominate). The clypeus of *M. xouthos* is distinctly folded back towards the mandible and the clypeal psammophore is placed on a ledge in the minor worker, the head, mesosoma and gaster have relatively long, whitish, appressed setae that overlap and form a weak pubescence on the gaster and the ant has a distinct microreticulate or shagreenate sculpture and is matt or has a weak sheen. The minor worker has five mandibular teeth. These characters serve to differentiate *M. xouthos* from all other *Melophorus* except *M. parvimolaris*. However, *M. xouthos* is tawny orange-and-black whereas *M. parvimolaris* is uniformly brown. The major worker is unknown. Odd, wingless, worker-like *Melophorus* males are tentatively placed in this species.

#### Minor worker description.

**Head.** Head square tending to trapezoid; posterior margin of head planar or weakly convex; frons matt or with weak sheen, shagreenate; frons consisting exclusively or almost exclusively of well-spaced, appressed setae only (small, erect setae, if present, usually confined to ocular triangle or posterior margin of head). Eye moderate (eye length 0.20–0.49 length of side of head capsule); in full-face view, eyes set above midpoint of head capsule; in profile, eye set anteriad of midline of head capsule; eyes elliptical or slightly reniform. In full-face view, frontal carinae straight, divergent posteriad; frontal lobes curved towards antennal insertion. Anteromedial clypeal margin straight; clypeal psammophore set at or above midpoint of clypeus; palp formula 6,4. Five mandibular teeth in minor worker; narrow, strap-like, internal and external margins parallel or nearly so; third mandibular tooth only slightly smaller than tooth no. 4 but much smaller than tooth no. 2 in two specimens seen; masticatory margin of mandibles strongly oblique. **Mesosoma.** Integument of pronotum, mesonotum and mesopleuron matt or with a weak sheen and shagreenate throughout; anterior mesosoma in profile weakly elevated anteriad, thereafter gently sinuate, pronotum and mesonotum on same plane; erect pronotal setae absent; in profile, metanotal groove a weak or vestigial furrow; propodeum matt or with a weak sheen and indistinctly shagreenate; propodeum smoothly rounded or with indistinct angle; length ratio of propodeal dorsum to its declivity not applicable, propodeal dorsum and declivity confluent; erect propodeal setae always absent; appressed propodeal setulae long, each reaching setae behind and in front, but not forming pubescence; propodeal spiracle situated on or beside declivitous face of propodeum, and shorter (length < 0.50 × height of propodeum). **Petiole.** In profile, petiolar node narrowly rectangular, vertex blunt, directed posteriad; in full-face view, node tapered with blunt vertex; node matt with indistinct microsculpture. **Gaster.** Gaster weakly shining, shagreenate (‘LP record’ appearance); pilosity of first gastral tergite consisting of well-spaced, long, whitish, appressed setae with occasional erect non-marginal setae present in at least some workers, as well as a few marginal setae on the first gastral sclerite. **General characters. **Colour a dingy orange with some brownish infuscation on head and anterior mesosoma, legs black with bluish iridescence, gaster chocolate.

#### Measurements.

Worker (n =) : CI; EI; EL; HL; HW; ML; MTL; PpH; PpL; SI; SL.

#### Comments.

Only a handful of minor workers are known for this small *M. wheeleri* complex species, which appears to be confined to remote inland areas of SA and WA but may also occur in the southern NT. The long, whitish setae on the gaster and the appearance of the clypeus help separate this ant from all except one other species in the *laticeps* cluster, and the attractively mottled appearance distinguishes this ant from the very similar *M. parvimolaris*. While the five-gene tree places it as the sister to *M. fulvidus*, and the three-gene tree places the species as sister to *M. perthensis* (both of these ants being in the *M. fieldi* complex), both groupings are only weakly supported. Specimens taken from the Little Sandy Desert, WA, were collected on a dune crest over red sand with a scattered shrub and spinifex vegetative layer. However, the habits of the species are unknown.

#### Etymology.

Greek *xouthos* (‘yellowish-brown’); adjective in the nominative singular.

**Figure 88.**
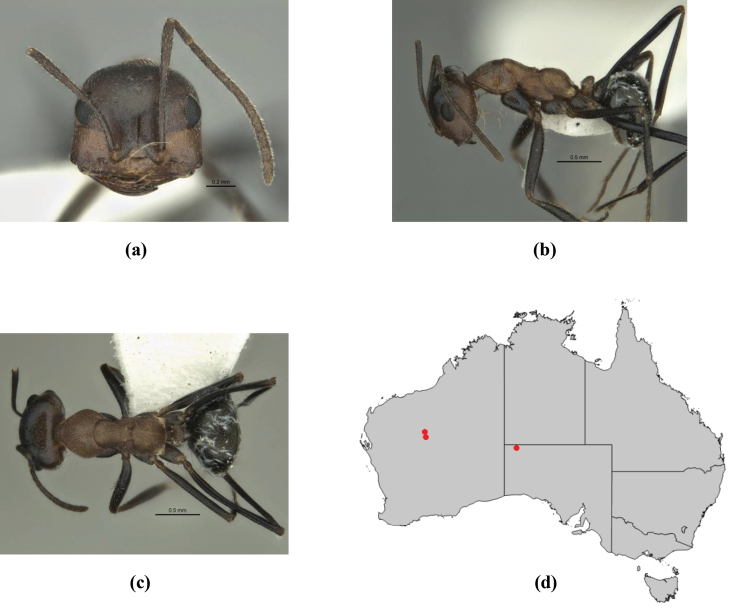
*Melophorus xouthos* sp. n.: holotype minor worker (ANIC32-900211) frons (**a**), profile (**b**) and dorsum (**c**); distribution map for the species (**d**). Low resolution scale bars: 0.5 mm (**b, c**); 0.2 mm (**a**).

### ‘Species K’

(specimens held in the TERC Collection, Darwin, and not available for loan). Specimens seen have prominent mandibles with many pointed teeth. However, their **facies** enabled the ants to be placed comfortably in the *M. wheeleri* species-complex. Subcaste was not recorded during a packed visit, but the ants were likely to have been minor workers (minor workers are the subcaste specified in Andersen’s (2007) key). Material in TERC has been collected from Kalkarindji NP (NT) and Purnululu NP (WA), respectively. However, the description (one major worker) below may relate to the major worker of this species. The description was originally attached to *M. griseus*, but the major worker of that species is unknown, and the nature of the mandibles suggests this ant is actually Species ‘K’. Unfortunately, the description was derived a number of years ago and the circumstances under which it was generated and the whereabouts of the specimen itself are unknown.

**Major worker description. Head.** Head square; posterior margin of head strongly concave; cuticle of frons shining and smooth except for piliferous pits; pilosity of frons a mixture of a few well-spaced, erect setae interspersed with appressed setae only. Eyes set at about midpoint of head capsule. Frontal lobes curved inward in front of antennal insertion. Anterior clypeal margin broadly concave; clypeal psammophore set at or just above anterior clypeal margin. Mandibular teeth in major worker more than 12; mandibles triangular, weakly incurved; masticatory margin of mandibles approximately aligned vertically or weakly oblique. **Mesosoma.** Integument of pronotum, mesonotum and mesopleuron shining and mainly smooth, vestigial shagreenation most noticeable on humeri and mesopleuron; anterior mesosoma in profile convex anteriad, mesonotum overlapping pronotum, planar or slightly sinuate posteriad; erect pronotal setae short, (i.e., shorter than length of eye) and unmodified; propodeum angulate, propodeal angle blunt; propodeal spiracle situated nearer to midpoint of propodeum than to its declivitous face, and longer (length ≥ 0.50 × height of propodeum). **Petiole.** In profile, petiolar node squamiform; in full-face view, shape of petiolar node tapered with sharp vertex; node shining and faintly striolate and microreticulate. **Gaster. **Gaster shining with superficial microreticulation; pilosity of first gastral tergite consisting of well-spaced, erect and semi-erect setae interspersed with regularly spaced appressed setae. **General characters.** Colour blackish-red.

**Measurements.** Worker (n = 1): CI 93–102; HFL 2.17–2.46; HL 0.64–0.7; HW 0.63–0.66; SI 90–96; SL 0.58–0.61.’

As a specimen or specimens of this interesting species was/were not able to be arranged on loan from TERC, a formal description description cannot be included in this monograph.

### *Melophorus fulvihirtus* species-group

This small group of rather stocky, prickly-looking *Melophorus* consists of two localized species from temperate mainland Australia. Like *M. anderseni*, *Melophorus fulvihirtus* is a raider of meat ant nests (in this case those of the common meat ant, *M. purpureus*), but it is not known whether the other constituent species has similar habits. No specimens of this species-group were successfully sequenced, although efforts were made to obtain a gene sequence from *Melophorus barbellulatus*.

### Melophorus barbellulatus

Taxon classificationAnimaliaHymenopteraFormicidae

Heterick, Castalanelli & Shattuck sp. n.

http://zoobank.org/566F2346-48EE-4520-AFA9-8DA7EB24EA87

#### Types.

Holotype media worker (middle ant on top rectangle) from Tardun, Western Australia, 24 May 1963, C. Mercer, *Melophorus* [ANIC32-900080]. Paratypes: 2 major workers, alate queen and 2 males on two rectangles on same pin with same details as holotype (ANIC); minor worker from Boddington, Western Australia, 1983, A.C. Postle, Worsley FAFS III WOR 103, ‘Name: *Melophorus fulvihirtus*, ID: Heterick, B.E., Date: 6 December 2001’ [*sic*] [Note: this ant is closely related to *M. fulvihirtus* but is not that species-BEH] [JDM32-001976] (WAM).

#### Other material examined.

**South Australia**: 14 km Keilira Stn (Hirst, D. [M110]).

#### Diagnosis.

*Melophorus barbellulatus* is one of only two members of the *M. fulvihirtus* species-group, both identified by having the head and mesosoma extensively covered with short, stout, peg-like bristles and, in outline, the pronotum and mesonotum flattened. The metatibial apical spur is stout but very short. This species is distinguished from its sister species by having the short peg-like setae much reduced on the antennal scape and absent from the legs, and the cuticle is finely shagreenate and weakly shining (matt and coriaceous in *M. fulvihirtus*). The posterior posterior margin of head of major and minor workers is planar, and the mandible of the minor worker is coarsely striate.

#### Minor worker description.

**Head.** Head square; posterior margin of head planar or weakly concave; frons matt or with weak sheen, microreticulate or microreticulate-shagreenate; frons consisting of very short, erect, spinous setae with closely aligned, very minute, appressed setae that create a faint silvery sheen. Eye moderate (eye length 0.20–0.49 length of side of head capsule); in full-face view, eye set at about midpoint of head capsule; in profile, eye set around midline of head capsule; eyes elliptical or slightly reniform. In full-face view, frontal carinae concave; frontal lobes curved inward in front of antennal insertion. Anteromedial clypeal margin broadly and evenly convex; clypeal psammophore set at or just above anterior clypeal margin; palp formula 6,4. Five to six mandibular teeth in minor worker; mandibles triangular, weakly incurved; third mandibular tooth distinctly shorter than apical tooth, but equivalent in length to remaining teeth; masticatory margin of mandibles approximately vertical or weakly oblique. **Mesosoma.** Integument of pronotum, mesonotum and mesopleuron matt or with weak sheen and microreticulate throughout; anterior mesosoma in profile smoothly rounded anteriad, thereafter pronotum and whole of mesonotum flattened and on same plane as propodeum; erect pronotal setae absent; in profile, metanotal groove a weak or vestigial furrow; propodeum matt or with a weak sheen and indistinctly shagreenate; propodeum angulate, propodeal angle blunt; length ratio of propodeal dorsum to its declivity about 1:1; erect propodeal setae present and abundant (greater than 12); appressed propodeal setulae minute and closely aligned, creating a silvery sheen; propodeal spiracle situated on or beside declivitous face of propodeum, and shorter (length < 0.50 × height of propodeum). **Petiole.** In profile, petiolar node squamiform; in full-face view, shape of petiolar node uniformly rounded; node matt with indistinct microsculpture. **Gaster.** Gaster weakly shining with indistinct shagreenation; pilosity of first gastral tergite consisting of thick, appressed setae that form pubescence, interspersed with numerous short, bristly, erect setae. **General characters.** Colour tan with areas of brown infuscation.

#### Major worker description.

**Head.** Head square; posterior margin of head planar or weakly concave; cuticle of frons matt or with weak sheen, microreticulate; frons consisting of very short, erect, spinous setae with closely aligned, very minute, appressed setae creating a faint silvery sheen. Eye moderate (eye length 0.20–0.49 length of head capsule); in full-face view, midpoint of head capsule; in profile, eye set anteriad of midline of head capsule; eyes elliptical. In full-face view, frontal carinae concave; frontal lobes curved toward antennal insertion. Anterior clypeal margin convex, acuminate anteromedially, margin entire; clypeal psammophore set at or just above anterior clypeal margin; palp formula 6,4. Five mandibular teeth in major worker; mandibles triangular, weakly incurved; third mandibular tooth distinctly shorter than apical tooth and teeth numbers two and four; masticatory margin of mandibles approximately aligned vertically or weakly oblique. **Mesosoma.** Integument of pronotum, mesonotum and mesopleuron matt or weakly shining and coriaceous; anterior mesosoma in profile steeply rounded anteriad, thereafter pronotum and whole of mesonotum flattened and on a higher plane than propodeum; erect pronotal setae absent; in profile, metanotal groove shallow, indicated mainly by an angle and metathoracic spiracles; propodeum matt or with a weak sheen and microreticulate; propodeum distinctly angulate, propodeal angle sharp; length ratio of propodeal dorsum to its declivity greater than 1:2; erect propodeal setae present and abundant (at least a dozen); appressed propodeal setae minute and closely aligned, creating a silvery sheen; propodeal spiracle situated on or beside declivitous face of propodeum, and shorter (length less than 0.50 × height of propodeum). **Petiole.** In profile, petiolar node squamiform; in full-face view, shape of petiolar node generally rounded with median indentation; node matt, and microreticulate. **Gaster.** Gaster weakly shining with indistinct shagreenation; pilosity of first gastral tergite consisting of well-spaced, short, thick, erect setae interspersed with minute, appressed setae. **General characters.** Colour of foreparts orange, gaster brown.

#### Measurements.

Worker (n = 3): CI 99–114; EI 20–28; EL 0.18–0.27; HL 0.62–1.31; HW 0.62–1.31; ML 0.79–1.48; MTL 0.45–0.76; PpH 0.10–0.13; PpL 0.36–0.69; SI 71–109; SL 0.68–0.93.

#### Comments.

*Melophorus barbellulatus* has been found in South Australia (SAMA, TERC), Victoria (TERC) and WA (WAM, TERC) but is nowhere common. The nature of the trunk and the short, peg-like setae align it with *M. fulvihirtus*, but sequencing of one specimen was unsuccessful, for reasons that are uncertain. The ant is rendered distinctive by reason of details of the sculpture, mandible and vertex of the head (see species-group key). No ecological or other data are available from labels, and it is unknown whether this species is an ant raider like its relative.

#### Etymology.

Latin dim. of *barbella* (‘short, stiff hair’) plus -*atus* (‘of the nature of’) referring to the tiny, short, stiff setae on the body; noun in the nominative singular standing in apposition to the generic name.

**Figure 89.**
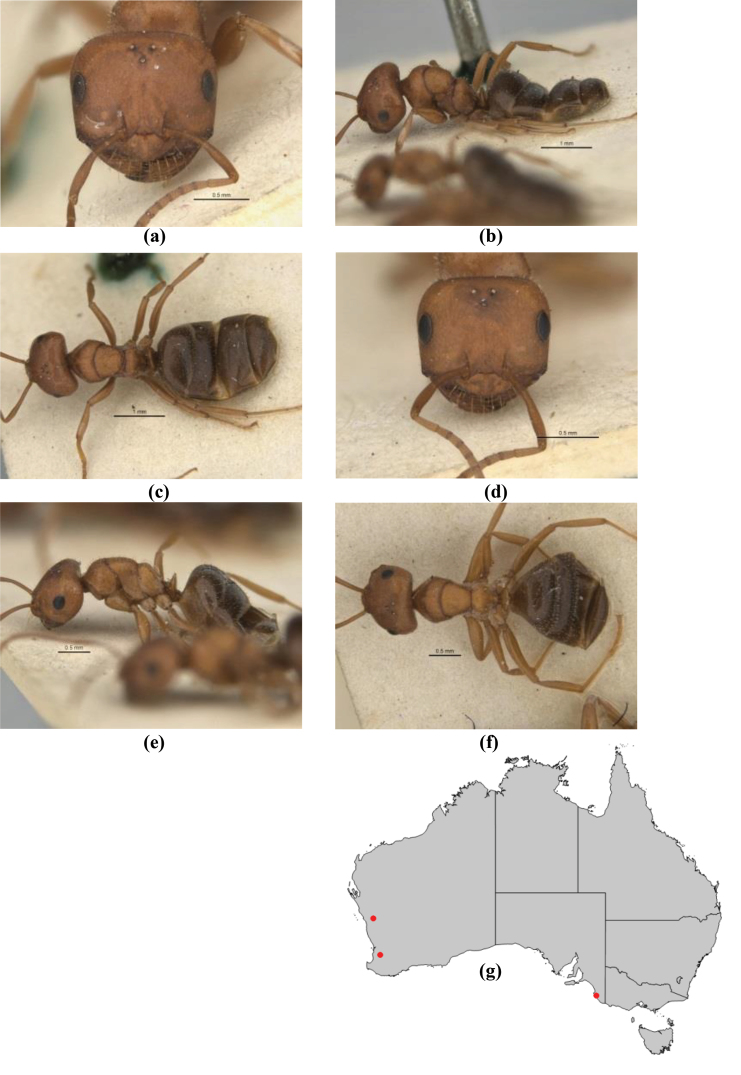
*Melophorus barbellulatus* sp. n.: major worker paratype (ANIC32-900080–ant nearest pin on top rectangle) frons (**a**), profile (**b**) and dorsum (**c**); minor worker holotype (ANIC32-900080–middle ant on top rectangle) frons (**d**), profile (**e**) and dorsum (**f**); distribution map for the species (**g**). Low resolution scale bars: 1 mm (**b, c**); 0.5 mm (**a, d–f**).

### Melophorus fulvihirtus

Taxon classificationAnimaliaHymenopteraFormicidae

Clark

Melophorus fulvihirtus Clark 1941: 88, pl. 13, figs 19-21.
Melophorus fulvohirtus Clark 1941 (invalid (unavailable name): misspelling in literature; so Bolton 1995, et al.) Types. Syntype major and minor workers, the minor worker headless: Patho, Victoria [MV] (examined: MV specimens T-11549, T-11550, T-11551).

#### Other material examined.

**New South Wales**: Campbelltown (Lowery, B.B. [ANIC32-900076]), Gabondery Mountains, 12 mi NW Trundle (Lowery, B.B.), Marayong, Sydney (Lowery, B.B.). **South Australia**: Teatree Gully (Halliday, R.B. [ANIC32-900178]). **Victoria**: Patho (Potter, H.A. [ANIC32-053435]), Patho (Potter, H.A.), Patho (Potter, H.A.), Patho (Potter, H.A.).

#### Diagnosis.

*Melophorus fulvihirtus is* one of only two members of the *M. fulvihirtus* species-group identified by having the head and mesosoma extensively covered with short, stout, peg-like bristles and, in outline, pronotum and mesonotum flattened. The metatibial apical spur is stout but very short. In *M. fulvihirtus* the posterior margin of the head of both major and minor workers is broadly concave, the appearance of the cuticle is matt and coriaceous, and the mandible of the minor worker is very finely striate, the individual striae being invisible under low power magnification

#### Minor worker description.

**Head.** Head quadrate (i.e., heart-shaped); posterior margin of head weakly concave; frons coriaceous; frons consisting of very short, erect, spinous setae with closely aligned, very minute, appressed setae that create a faint silvery sheen. Eye moderate (eye length 0.20–0.49 length of side of head capsule); in full-face view, eyes set above midpoint of head capsule; in profile, eye set anteriad of midline of head capsule; eyes elliptical or slightly reniform. In full-face view, frontal carinae straight or weakly convex; frontal lobes straight in front of antennal insertion. Anteromedial clypeal margin broadly and evenly convex; clypeal psammophore set at or just above anterior clypeal margin; palp formula 6,4. Five to six mandibular teeth in minor worker; mandibles triangular, weakly incurved; third mandibular tooth distinctly shorter than apical tooth and teeth numbers two and four; masticatory margin of mandibles approximately vertical or weakly oblique. **Mesosoma.** Integument of pronotum, mesonotum and mesopleuron matt and coriaceous; anterior mesosoma in profile smoothly rounded anteriad, thereafter pronotum and whole of mesonotum flattened and on same plane as propodeum; appearance of erect pronotal setae short and spinous; in profile, metanotal groove a weak or vestigial furrow; propodeum matt or with a weak sheen and microreticulate; propodeum distinctly angulate, propodeal angle sharp; length ratio of propodeal dorsum to its declivity greater than 1:2; erect propodeal setae present and abundant (greater than 12); appressed propodeal setulae minute and closely aligned, creating a silvery sheen; propodeal spiracle situated on or beside declivitous face of propodeum, and shorter (length < 0.50 × height of propodeum). **Petiole.** In profile, petiolar node squamiform; in full-face view, shape of petiolar node square with rounded angles; node matt and coriaceous, the peg-like setae with cuticular bases that protrude, creating a crenulate outline. **Gaster.** Gaster weakly shining and microreticulate; pilosity of first gastral tergite consisting of short, spinous, erect setae interspersed with massed, extremely minute, appressed setae that are visible only in certain lights. **General characters.** Colour tan with areas of brown infuscation.

#### Major worker description.

**Head.** Head square; posterior margin of head planar or weakly concave; cuticle of frons matt or with weak sheen, microreticulate; frons consisting of very short, erect, spinous setae with closely aligned, very minute, appressed setae creating a faint silvery sheen. Eye moderate (eye length 0.20–0.49 length of head capsule); in full-face view, midpoint of head capsule; in profile, eye set anteriad of midline of head capsule; eyes elliptical. In full-face view, frontal carinae straight, divergent posteriad; frontal lobes straight in front of antennal insertion. Anterior clypeal margin broadly and evenly convex; clypeal psammophore set below midpoint of clypeus; palp formula 6,4. Five mandibular teeth in major worker; mandibles triangular, weakly incurved; third mandibular tooth distinctly shorter than apical tooth and teeth numbers two and four; masticatory margin of mandibles approximately aligned vertically or weakly oblique. **Mesosoma.** Integument of pronotum, mesonotum and mesopleuron matt or weakly shining and coriaceous; anterior mesosoma in profile steeply rounded anteriad, thereafter pronotum and whole of mesonotum flattened and on a higher plane than propodeum; erect pronotal setae short and often expanded distally, at times clavate; in profile, metanotal groove shallow, broadly V- or U-shaped; propodeum shining and microreticulate, or matt or with a weak sheen and microreticulate; propodeum angulate, propodeal angle blunt; length ratio of propodeal dorsum to its declivity greater than 1:2; erect propodeal setae present and abundant (at least a dozen); appressed propodeal setae short, separated by more than own length and inconspicuous; propodeal spiracle situated on or beside declivitous face of propodeum, and shorter (length less than 0.50 × height of propodeum). **Petiole.** In profile, petiolar node squamiform; in full-face view, shape of petiolar node uniformly rounded, or square with rounded angles; node shining and faintly shagreenate-microreticulate, or matt, and microreticulate. **Gaster.** Gaster weakly shining with indistinct shagreenation; pilosity of first gastral tergite consisting of well-spaced, short, thick, erect setae interspersed with minute, appressed setae. **General characters.** Colour of foreparts orange, gaster brown.

#### Measurements.

Worker (n = 6): CI 116–130; EI 17–23; EL 0.24–0.40; HL 0.90–1.85; HW 1.05–2.40; ML 1.45–2.31; MTL 0.80–1.18; PpH 0.16–0.20; PpL 0.66–1.07; SI 57–126; SL 1.33–1.37.

#### Comments.

The appearance of *M. fulvihirtus* is rendered unmistakable because of a combination of short, stout, peg-like setae, deeply incised head capsule, broadly flattened femora and coriaceous cuticle. No specimens of this singular ant were available for sequencing, and, its apparently close ties with *M. barbellulatus* excepted, its relationship with other members of the genus is unknown. This species seems to be confined to southeastern Australia, where it has been collected in New South Wales, South Australia and Victoria. Apart from the account of the raiding habits of the ant in the paper by John Clark (1941) who described the species, label data from NSW (Gabondery Mts, 12 mi NW Trundle; Campbelltown, Sydney) also includes reference to the specimens being collected from *Iridomyrmex purpureus* mounds. Oddly enough, Clark does not mention what the raiding minor workers of this species were carrying, so the reader is left to guess whether their booty was eggs, larvae, pupae or provisions-a strange omission! Also rather odd is the fact that no material held in ANIC or SAMA seems to have been collected after 1974, and most specimens were taken much earlier, some from areas that are now highly urbanized. No material at all is held by QM, TERC or WAM. This leads us to wonder if populations of the ant have declined in recent decades. Certainly, populations of the putative host have not! Apart from its raiding behavior, label data are lacking for this species.

**Figure 90.**
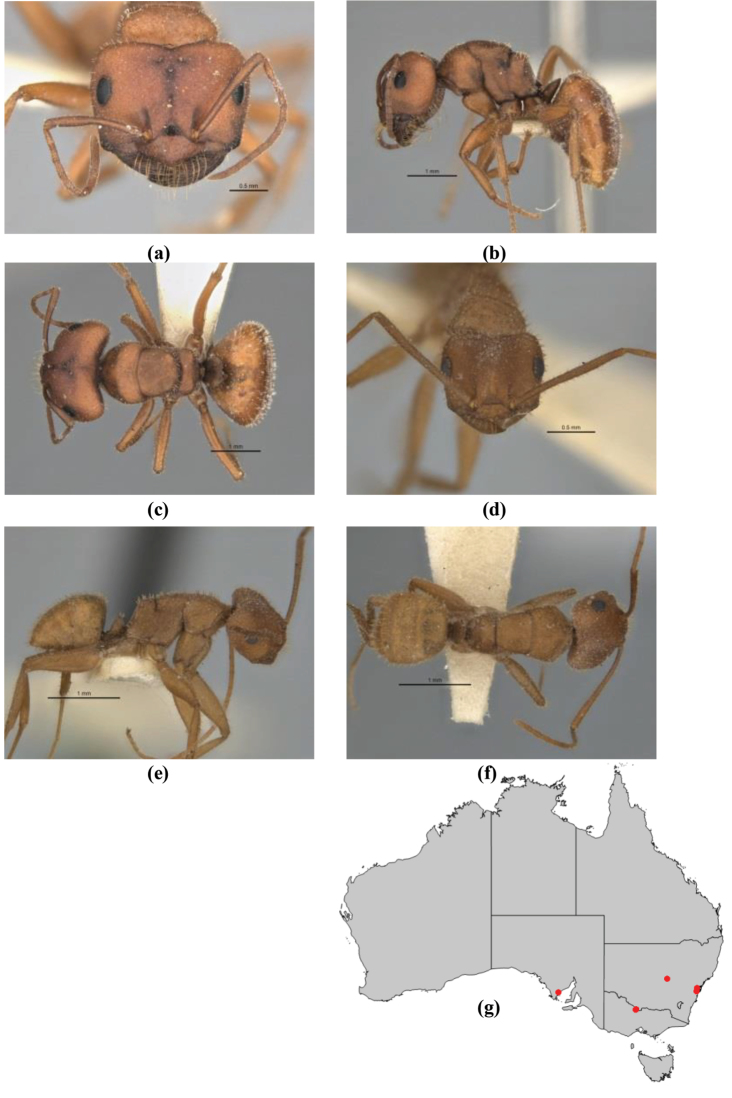
*Melophorus fulvihirtus* Clark: non–type major worker (ANIC32-900178) frons (**a**), profile (**b**) and dorsum (**c**); non–type minor worker (ANIC32-900076) frons (**d**), profile (**e**) and dorsum (**f**); distribution map for the species (**g**). Low resolution scale bars: 1 mm (**b, c**, **e, f**); 0.5 mm (**a, d**).

### *Melophorus ludius* species-group

#### *Melophorus hirsutus* complex

The sole member of this complex is a morphologically bizarre, cool-temperate species that has some anatomical links with the *M. ludius* group. Given its distribution and known habits, the species appears not to be thermophilic.

##### Melophorus hirsutus

Taxon classificationAnimaliaHymenopteraFormicidae

Forel

Melophorus hirsutus Forel 1902: 488 (combination in M. (Trichomelophorus) by Wheeler 1935: 71). Types. Syntype major and minor worker: MacKey, Queensland [MHNG] (examined: MHNG specimens).

###### Other material examined.

**Australian Capital Territory**: ANU (CFS) (Narendra, A. [M167]), Black Mountain (Taylor, R.W. [ANIC32-900043]), Black Mountain Reserve (Devonshire, J. [ANIC32-045115]), Black Mt., Site 1 (Barnett, N.J. [ANIC32-029779]), Mt. Ainslie (Shattuck, S.O. [ANIC32-047357]). **New South Wales**: 10 mi N Berowra (Lowery, B.B. [ANIC32-900035]), 4 km NE Mt. Wog Wog, 17 km SE Bombala (Margules, C.R. [ANIC32-900045]), 8 mi W Somerton (Greaves, T. [ANIC32-900038]), Armidale (Lowery, B.B. [ANIC32-900046]), Belanglo State Forest (Gush, T. [ANIC32-900034]), Bilpin, Blue Mountains (Lowery, B.B. [ANIC32-900032]), Burns Bay on Lane Cove River, Sydney (Lowery, B.B. [ANIC32-900044]), Eulah Creek, Narrabri (Room, P.M. [ANIC32-900040]), Katoomba (Wheeler, W.M. [ANIC32-900033]), Ourimbah State Forest (Lowery, B.B. [ANIC32-900039]), Uralla (Wheeler, W.M. [ANIC32-900049]), Whiporie, 55 km S Casino (York, A. [ANIC32-900041]). **Queensland**: Buhot Ck, Burbank (QM Party), Cairns (general) (Greaves, T. [ANIC32-045325]), Camp Mountain, Brisbane (Lowery, B.B. [ANIC32-900037]), Camp Mountain, Brisbane (Lowery, B.B. [ANIC32-900048]), Kirrama (Crowley, G. [ANIC32-900047]), Lords Table, SE base (Burwell), Peawaddy Gorge Lkt, 1 km S (Eddie, C. et al.), Tindaree, Hannaford Rd South via Tara (House, A./Brown, S.). **Victoria**: Ferntree Gully (Greaves, T. [ANIC32-900031]), Greensborough (McAreavey, J. [ANIC32-900036]), Lerderderg Gorge (Lowery, B.B. [ANIC32-900042]).

###### Diagnosis.

*Melophorus hirsutus* is distinguished from all other *Melophorus* by having the pronotum surrounded by a translucent lamina, the metanotal groove obsolete in the minor worker and just a weak furrow in major worke, the torulus pedunculate around the antennal insertions, and the cuticle of mesosoma with dense striolate-microreticulate sculpture. The ant is monotypic for its species-complex, but is tentatively placed in the *M. ludius* species-group on the basis of molecular evidence. The species is restricted to the eastern Australian seaboard.

###### Minor worker description.

**Head.** Head approximately oval with straight sides; posterior margin of head weakly convex; frons shining and smooth except for piliferous pits and a few striolae around antennal insertions and frontal carinae; pilosity of frons a mixture of a few well-spaced, erect setae interspersed with appressed setae only. Eye small (eye length less than 0.2 × length of side of head capsule); in full-face view, eyes set at about midpoint of head capsule; in profile, eye set around midline of head capsule; roughly ovoid, eye narrowed posteriad. In full-face view, frontal carinae absent or weakly produced in front of antennal insertions; frontal lobes pedunculate. Anteromedial clypeal margin broadly and evenly convex; clypeal psammophore set at or just above anterior clypeal margin; palp formula 6,4. Five to six mandibular teeth in minor worker; mandibles triangular, weakly incurved; third mandibular tooth distinctly shorter than apical tooth and teeth numbers two and four; masticatory margin of mandibles approximately vertical or weakly oblique. **Mesosoma.** Integument of pronotum, mesonotum and mesopleuron shining and striolate-micropunctate, with some shallow, oval depressions; anterior mesosoma in profile smoothly rounded anteriad, thereafter pronotum and whole of mesonotum flattened and on same plane as propodeum; appearance of erect pronotal setae long (i.e., longest erect setae longer than length of eye) and unmodified; in profile, metanotal groove vestigial but indicated by metathoracic spiracles; propodeum shining and finely striolate and microreticulate; propodeum planar dorsally with abrupt declivity; length ratio of propodeal dorsum to its declivity about 1:1; erect propodeal setae present and abundant (greater than 12); appressed propodeal setulae sparse or absent, if present then not regularly spaced; propodeal spiracle situated on or beside declivitous face of propodeum, and shorter (length < 0.50 × height of propodeum). **Petiole.** In profile, petiolar node squamiform; in full-face view, shape of petiolar node uniformly rounded; node shining and smooth throughout. **Gaster.** Gaster smooth and glossy; pilosity of first gastral tergite consisting of well-spaced, erect and semi-erect setae interspersed with regularly placed appressed setae. ***General Characters*.** Colour dark reddish brown, appendages tan.

###### Major worker description.

**Head.** Head square; posterior margin of head planar or weakly concave; cuticle of frons shining and finely striolate and microreticulate; pilosity of frons a mixture of a few well-spaced, erect setae interspersed with appressed setae only. Eye small (eye length less than 0.2 × length of head capsule); in full-face view, eyes set at about midpoint of head capsule; in profile, eye set anteriad of midline of head capsule; eyes elliptical. In full-face view, frontal carinae absent or weakly produced posteriad of antennal insertions; frontal lobes pedunculate. Anterior clypeal margin broadly and evenly convex; clypeal psammophore set at or just above anterior clypeal margin; palp formula 6,4. Five mandibular teeth in major worker; mandibles triangular, weakly incurved; third mandibular tooth distinctly shorter than apical tooth and teeth numbers two and four; masticatory margin of mandibles approximately aligned vertically or weakly oblique. **Mesosoma.** Integument of pronotum, mesonotum and mesopleuron shining and striolate-micropunctate, with some shallow, oval depressions; anterior mesosoma in profile smoothly rounded anteriad, thereafter pronotum and whole of mesonotum flattened and on same plane as propodeum; erect pronotal setae long (i.e., longer than length of eye) and unmodified; in profile, metanotal groove shallow, indicated mainly by an angle and metathoracic spiracles; propodeum shining and finely striolate and microreticulate; propodeum planar dorsally with abrupt declivity; length ratio of propodeal dorsum to its declivity between 1:1 and1:2; erect propodeal setae present and abundant (at least a dozen); appressed propodeal setae sparse or absent, if present then not regularly spaced propodeal spiracle situated on or beside declivitous face of propodeum, and shorter (length less than 0.50 × height of propodeum). **Petiole.** In profile, petiolar node squamiform; in full-face view, shape of petiolar node generally rounded with median indentation, node shining and smooth throughout. **Gaster.** Gaster smooth and glossy; pilosity of first gastral tergite consisting of a mixture of curved, erect and semi- erect setae and decumbent setae that form a variable pubescence. **General characters.** Colour of head crimson, mesosoma dark reddish-brown, gaster brown, appendages dark tan.

###### Measurements.

Worker (n = 6): CI 106–119; EI 12–17; EL 0.15–0.19; HL 0.82–1.26; HW 0.87–1.51; ML 1.18–1.75; MTL 0.75–1.07; PpH 0.13–0.18; PpL 0.62–0.73; SI 91–124; SL 1.37–1.08.

###### Comments.

Along with *M. majeri* this peculiar species is the most morphologically aberrant *Melophorus*, with its barrel-like mesosoma (not unlike that of some diapriid wasps), a variably pedunculated torulus otherwise found only in some *M. mjobergi* complex species and in one population of *M. lanuginosus*, and the margins of the pronotal humeri that are flattened to form a translucent flange. The range of this species is also unusual, as its main distribution is in the mountains and hills of the Great Dividing Range south of about Brisbane. North of Brisbane, although there are a few scattered records along the Great Dividing Range as far north as Cairns. States in which this ant occurs include ACT, NSW, QLD and Vic. There are no records for the NT, SA, Tas or WA.

The syntype major and minor workers reveal a rather conservative appearance compared with some populations: the torulus is only weakly pedunculate, the metathoracic spiracles are narrowly protuberant but not stalk-like and the compound eyes are of normal appearance. The two MHNG specimens appearing on separate points on the same pin are accompanied by one of Forel’s red ‘typus’ labels and almost certainly represent the two workers to which he refers in his description. The supposed syntype specimen Automontaged by AntWeb as CASENT0903268 (BMNH) should therefore be removed from the type series, despite similar collection details.

The morphology of the ant is very variable, the conformation of the eye being particularly noticeable and this may be of taxonomic significance: in some specimens the eye is quite conical and bizarrely projecting, while in others it is merely slightly bulbous. Intermediate states have been seen. Identifying and finding a pattern in this morphological variability would make for a worthwhile student project. Unfortunately, sequenceable material has been too limited in this project to be of use in identifying such variation, with only one specimen providing a signal for the five genes used in this project. Although tentatively placed within its own complex because of its odd morphology, mtDNA situates this species more broadly within the *M. ludius* species-group. However, this species shares with the *M. biroi* species-group the extremely flattened petiolar node in the major worker. Specimens have been collected in a variety of woodland habitats, including remnant brigalow, eucalypt forest and riparian forest.

**Figure 91.**
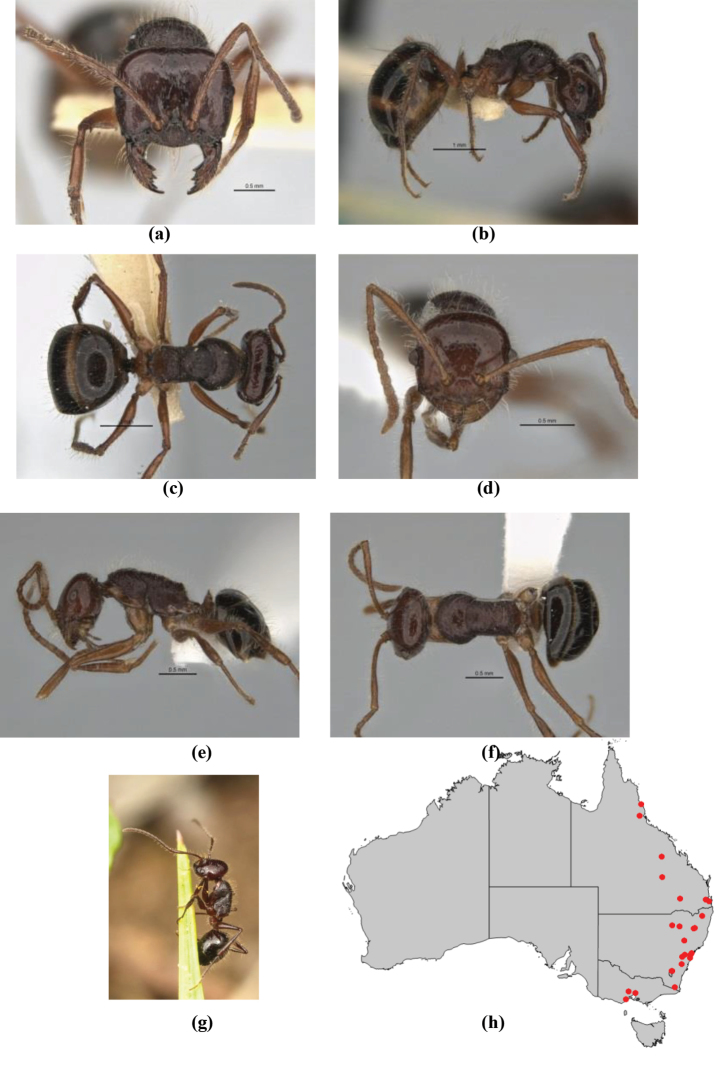
*Melophorus hirsutus* Forel: major worker (ANIC32-900039) frons (**a**), profile (**b**) and dorsum (**c**); minor worker (ANIC32-900043) frons (**d**), profile (**e**) and dorsum (**f**); *M. hirsutus* forager on grass stem (photo credit: A. J. Narendra) (**g**); distribution map for the species (**h**). Low resolution scale bars: 1 mm (**b, c**); 0.5 mm (**a, d–f**).

#### *Melophorus ludius* complex

This small complex of *Melophorus* cannot be distinguished morphologically from members of the *M. biroi* complex.

#### Melophorus ludius

Taxon classificationAnimaliaHymenopteraFormicidae

Forel

Melophorus ludius Forel 1902: 484. Types. Syntype major and minor workers (two pins): MacKey, Queensland [MHNG] (examined: MHNG specimens). Note: An Automontaged major and minor worker (2 × four views, AntWeb, CASENT0903266 and CASENT0903267, respectively) lack Forel’s hand-written labels and red ‘typus’ label, and may in fact not be syntypes. The blue ‘syntype’ label should therefore be treated with suspicion, although syntype status cannot be automatically excluded (see ICZN, section 73.2). Both specimens are the property of the BMNH.

##### Other material examined.

**Australian Capital Territory**: Capital Hill, Canberra (Lowery, B.B.). **New South Wales**: 40 km NNW Louth, Lake Mere (Greenslade, P.J.M.), CSIRO Lake Mere Field Station, near Louth (Bryannah, M.), CSIRO Lake Mere Field Station, near Louth (Bryannah, M.), Eastwood State Forest, near Armidale (Sakurai, Y.), Putty, 50 mi N Windsor (Lowery, B.B.), St. Ives (Lowery, B.B.), Tomago (Jackson, G.P. [ANIC32-015294]), Whiporie, 55 km S Casino (York, A.). **Queensland**: 14 km WbyN Hope Vale Mission (Cardale, J.C.), 8 km W Warwick (Greenslade, P.J.M.), Clermont, 4 km N (Burwell), Crows Nest NP, Perseverance Section (Burwell, C.), Karawatha forest site (QM Party), Kirrama (Greenslade, P.J.M.), Mount Coot-tha, Brisbane (Lowery, B.B. [ANIC32-900107]), Mutyi, Cooloola (Greenslade, P.J.M.), N. Stradbroke Is. Enterprise (QM Party), Noosa River, Cooloola (Greenslade, P.J.M.), Proserpine, Thompson Creek (Burwell, C.), Ransome Reserve (QM Party), State Forest 958 Bauple (House, A.P.N. & Vanderwoude, C.), Toomba (Monteath, G., Cook, D.). **South Australia**: 6 km W Wanilla, Eyre Peninsula (Greenslade, P.J.M.), Belair (Greenslade, P.J.M.), Belair (Greenslade, P.J.M.), Calca (Lowery, B.B.), Fairview Conservation Park, N Lucindale (Greenslade, P.J.M.), Kia-Ora, NE Burra (Greenslade, P.J.M.), Koonamore (Greenslade, P.J.M.), Para Wirra (Greenslade, P.J.M.), Salt Creek, Coorong (Greenslade, P.J.M.), Sandy Creek, Kangaroo Island (Greenslade, P.J.M.), Sevenhill (Lowery, B.B.), Sevenhill (Lowery, B.B.), Sevenhill (Lowery, B.B.), Sevenhill (Lowery, B.B.), Sevenhill (Lowery, B.B.), Spring Gully, Sevenhill (Lowery, B.B.), Wilpena Pound (Lowery, B.B.), Wudinna townsite (Heterick, B.E. [M332]). **Victoria**: 15 km WNW Yaapeet (Andersen, A.N.), 15 km WNW Yaapeet (Andersen, A.N.), 15 km WNW Yaapeet (Andersen, A.N.), 15 km WNW Yaapeet (Andersen, A.N.), 15 km WNW Yaapeet (Andersen, A.N.), Wyperfeld National Park (Andersen, A.N.). **Western Australia**: 1.5 km S Koolyanobbing (Heterick, B.E. [M04/M27/M28]), 30 km E Kununurra (Lowery, B.B. [ANIC32-050220]), 30 km S of ‘The Overlander’ (Heterick, B.E. [JDM32-001890]), 40 km S Dongara (Lowery, B.B.), 42 mi N Mundiwindi (McInnes & Dowse), 64 km E of Southern Cross (Heterick, B.E. [JDM32-001891]), 80 km, West Talbot Road, Beverley (Douglas, A.M. & M.J.), Alcoa (Wallace, J. [JDM32-001912]), Bentley (Majer, J.D.), Bentley (Majer, J.D. [JDM32-001919]), Bunbury (Lowery, B.B.), Coalmine Beach, Nornalup National Park, Walpole (Lawrence, J. & N.), Coolbellup (Heterick, B.E. [JDM32-001888]), Dwellingup (Majer, J.D. [JDM32-001893]), Ethel Creek (Varris, P.A. [JDM32-001946]), Eyre Hwy, 20 km N Norseman (Heterick, B.E. [M337]), Gold Holes, Stirling Range (Taylor, R.W. [ANIC32-900106]), Hope Valley (Heterick, B.), Int. Holland Tr./Norseman Rd. (Heterick, B.E. [JDM32-001967]), Junana Rock, 9 km NW Mt. Ragged (Taylor, R.W.), Karragullen (Majer, J.D. [JDM32-001920]), Kings Park, Perth (Lowery, B.B.), Kings Park, Perth (Lowery, B.B.), Kojonup (Majer, J.D. [JDM32-001904]), Manjimup (Majer, J.D. [JDM32-001901]), Milyeannup (Parkinson, W. [M62/M63]), Mulga, NE Goldfields (Pringle, H.J.R. [ANIC32-029572]), nr. Stirling Dam (Heterick, B.E. [JDM32-001889]), Perry Lakes (Majer, J.D. [JDM32-001911]), Reabold H. (Majer, J.D. [JDM32-001908]), Serpentine Falls, Serpentine National Park (Shattuck, S.O.), Without Locality (collector unknown [JDM32-001921]).

##### Diagnosis.

*Melophorus ludius* is placed in the *M. ludius* species-group on the basis of molecular data. However, in morphological appearance this taxon shares major diagnostic characters with the *M. biroi* complex (viz, metatibia of major worker with only one preapical spur; clypeal psammophore placed anteriorly at or just above anterior margin of clypeus in the minor worker and often in the major worker; legs compact, and small body size [HW of smallest minor < 0.40 mm, HW of largest major < 1.10 mm]). Like the other three members of its species-group, *M. ludius* is characterised by being weakly sculptured overall, with the cuticle of the mesosoma visibly thin, the mesonotum being translucent to varying degrees and the mesopleuron either smooth or with vestigial sculpture only. *Melophorus ludius* is distinguished from *M. pusillus* by the smaller eye (EI in *M. ludius* 19-30 compared with 31-40 in *M. pusillus* and from *M. translucens* by the shorter propodeum and propodeal spiracle (both rather elongate in *M. translucens*), and, as regards the major worker, the less bulbous and rather more opaque mesonotum.

##### Minor worker description.

**Head.** Head rectangular; posterior margin of head weakly convex; frons shining and smooth except for piliferous pits; frons consisting exclusively or almost exclusively of well-spaced, appressed setae only (small, erect setae, if present, usually confined to ocular triangle or posterior margin of head). Eye moderate (eye length 0.20–0.49 length of side of head capsule); in full-face view, eyes set at about midpoint of head capsule; in profile, eye set around midline of head capsule; eyes elliptical or slightly reniform. In full-face view, frontal carinae distinctly concave; frontal lobes curved toward antennal insertion. Anteromedial clypeal margin straight; clypeal psammophore set at or just above anterior clypeal margin; palp formula 6,4. Five mandibular teeth in minor worker; mandibles triangular, weakly incurved; third mandibular tooth distinctly shorter than apical tooth and teeth numbers two and four; masticatory margin of mandibles approximately vertical or weakly oblique. **Mesosoma.** Integument of pronotum, mesonotum and mesopleuron shining and mainly smooth, vestigial shagreenation most noticeable on humeri and mesopleuron; anterior mesosoma in profile smoothly rounded anteriad, thereafter pronotum and whole of mesonotum flattened and on same plane as propodeum; erect pronotal setae absent in western populations but one or two erect setae may be present in eastern populations; in profile, metanotal groove shallow, indicated mainly by an angle; propodeum shining and smooth or with superficial and almost invisible microsculpture; propodeum angulate, propodeal angle blunt; length ratio of propodeal dorsum to its declivity between 1:1 and1:2; erect propodeal setae always absent; appressed propodeal setulae sparse or absent, if present then not regularly spaced; propodeal spiracle situated on or beside declivitous face of propodeum, and longer (length ≥ 0.50 × height of propodeum). **Petiole.** In profile, petiolar node squamiform; in full-face view, shape of petiolar node uniformly rounded; node shining and smooth with vestigial sculpture. **Gaster.** Gaster shining with superficial microreticulation; pilosity of first gastral tergite consisting of well-spaced short, inconspicuous, appressed setae only in western populations, erect setae always absent; minor workers in eastern populations may possess one or two erect gastral setae on first gastral tergite. **General characters.** Colour light brown or yellow tan, concolorous or bicoloured (gaster always darker in bicoloured workers).

##### Major worker description.

**Head.** Head square; posterior margin of head planar or weakly concave; cuticle of frons shining and smooth except for piliferous pits; frons consisting exclusively or almost exclusively of well-spaced, appressed setae only (small, erect setae, if present, usually confined to ocular triangle or posterior margin of head). Eye moderate (eye length 0.20–0.49 length of head capsule); in profile, eye set at about midpoint of head capsule; in full-face view, eye set around midline of head capsule; eyes elliptical. In full-face view, frontal carinae straight, divergent posteriad; frontal lobes straight in front of antennal insertion. Anterior clypeal margin straight; clypeal psammophore set below midpoint of clypeus; palp formula 6,4. Five mandibular teeth in major worker; mandibles triangular, weakly incurved; third mandibular tooth distinctly shorter than apical tooth and teeth numbers two and four; masticatory margin of mandibles approximately aligned vertically or weakly oblique. **Mesosoma.** Integument of pronotum, mesonotum and mesopleuron shining with indistinct microsculpture that is most pronounced on lower surfaces; anterior mesosoma in profile pronotum smoothly rounded anteriad and flattened posteriad, mesonotum narrowly convex; erect pronotal setae long (i.e., longer than length of eye) and unmodified; in profile, metanotal groove shallow, broadly V- or U-shaped; propodeum shining and finely striolate and microreticulate; propodeum always smoothly rounded; propodeal dorsum and declivity confluent; erect propodeal setae variable in number, may be absent; appressed propodeal setae sparse or absent, if present then not regularly spaced; propodeal spiracle situated on or beside declivitous face of propodeum, and shorter (length less than 0.50 × height of propodeum). **Petiole.** In profile, petiolar node squamiform; in full-face view, shape of petiolar node uniformly rounded, or tapered with sharp vertex; node shining and faintly striolate and microreticulate. **Gaster.** Gaster shining with superficial microreticulation; pilosity of first gastral tergite consisting of well-spaced, erect and semi-erect setae interspersed with regularly spaced appressed setae. **General characters.** Colour of foreparts shades of yellow, orange or light tan, gaster brown.

##### Measurements.

Worker (n = 8): CI 90–116; EI 19–30; EL 0.12–0.20; HL 0.44–0.91; HW 0.39–1.05; ML 0.55–1.18; MTL 0.30–0.66; PpH 0.05–0.11; PpL 0.24–0.50; SI 83–120; SL 0.47–0.88.

##### Comments.

*Melophorus ludius*, a minute species, is common throughout a wide range of habitats throughout all mainland Australian states, but the majority of records have come from the southern states, generally in wetter areas. However, there is no doubt this tiny ant is one of the more adaptable members of its genus. While varying in colour from pale yellow to brown (concolorous or bicoloured), the species can be distinguished by the shiny, barely sculptured cuticle, rounded head and moderate-sized eye in the minor worker and additional features of the clypeus in the major worker. Major workers have fine, often flexuous setae on the pronotum, and this feature, the narrower width of the clypeus and the more sloping propodeum distinguish them from *M. biroi* workers of similar appearance. The *M. biroi* workers of all sub-castes also tend to be more matt and shagreenate in appearance than *M. ludius* workers. Based on the appearance of the type specimens and other material from QLD, minor workers of *Melophorus ludius* sensu stricto from the east coast are larger, always bicoloured with yellow foreparts and brown gaster and have a longer scape than have their counterparts in WA. They also tend to be hairier, and such differences may be taxonomically significant. However, no material from eastern Australian localities was available for genetic research.

Despite its apparently unremarkable morphology, *M. ludius* is distantly removed from *M. biroi* and its relatives on all molecular trees and appears to have some connection with the very aberrant *M. potteri*. A three-gene tree (not the one shown under ‘Suppl. material 1’) places it as the sister species to *M. pusillus*, which it very closely resembles, the chief difference being the large eye of the latter. Just the one sample of the western form of *M. ludius* has been available for sequencing, but this has been sufficient to indicate the distant relationship to *M. biroi* despite its convergent morphology.

Ecological data, while sparse as usual given the number of pinned specimens, indicates the many habitats from which this ant has been collected throughout Australia. One label note records nesting in sand, and the associated vegetation mentioned on labels includes dry sclerophyll, mallee (burned in a couple of instances), *Eucalyptus maculata* open forest, *Eucalyptus largiflorens* woodland, sandstone scrub, paddock, *Casuarina* woodland, and mulga bushland. Sequenced material has also been collected from red loam soil and from a parking bay. Queensland Museum material includes ants taken from a nest under a rock. As is the case with *M. dicyrtos*, QM workers include a couple of samples taken from faeces-baited pitfall traps. The species is common in some urban situations in the Perth metropolitan area, where tiny workers can be seen foraging on suburban lawns. Because of their minute size, depigmented appearance and the rapidity of their movement over the ground, the principal author finds they can be difficult to distinguish from acarine Prostigmata (Teneriffiidae?) of the same size that move in similar fashion over the ground in the same locations. Given its ubiquity, this species is likely to be generalist in its habits.

**Figure 92.**
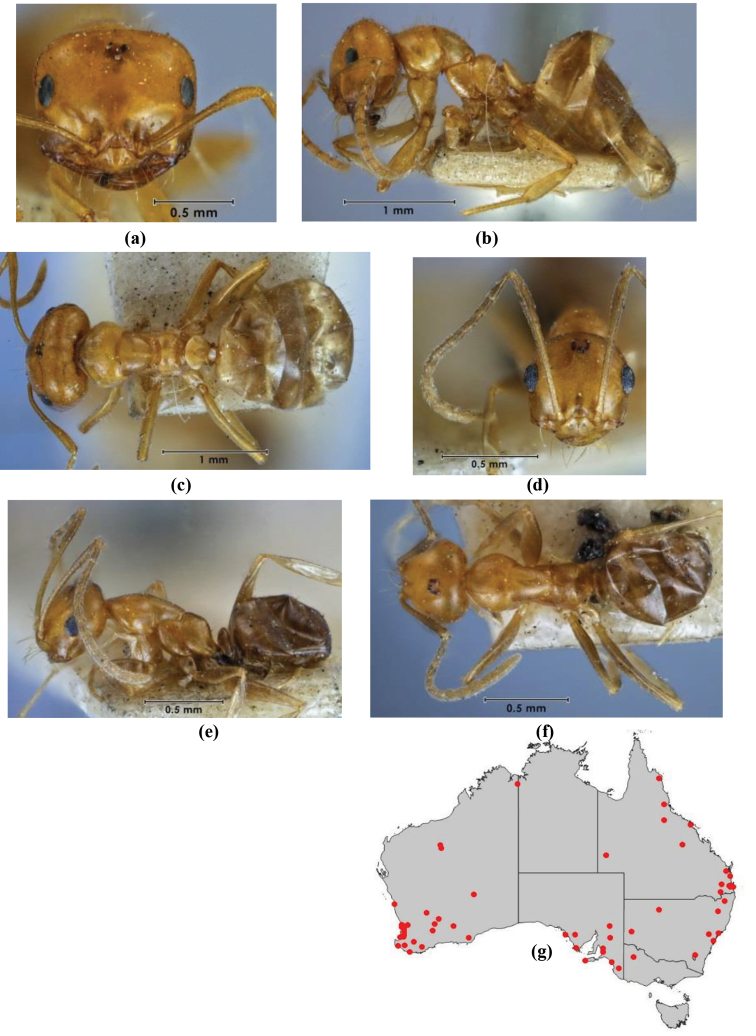
*Melophorus ludius* Forel: MHNG major worker syntype frons (**a**), profile (**b**) and dorsum (**c**); MHNG minor worker syntype (on same pin as major worker) frons (**d**), profile (**e**) and dorsum (**f**); distribution map for the species (**g**).

#### Melophorus pusillus

Taxon classificationAnimaliaHymenopteraFormicidae

Heterick, Castalanelli & Shattuck sp. n.

http://zoobank.org/3F0547F1-26C8-41A5-BF47-079D80A098C8

##### Types.

Holotype minor worker (bottom ant) from Tropicana Minesite 29°10'11"S, 124°33'38"E, Western Australia, January 2009, J. Summerhayes, pitfall trap *Casuarina*, CA 2:3 [JDM32-004668] (WAM). Paratypes: major worker on same pin and with same details as holotype (WAM); 2 minor workers from Kunoth Paddock, near Alice Springs, Northern Territory, 25 October 1980, P.J.M. Greenslade, SA EAD (ANIC); 2 minor workers from Gawler Ranges, South Australia, 1 October 1972, P.J.M. Greenslade, (9) (BMNH); 2 minor workers from 30 km S of Granite Downs, South Australia, 23 September 1980 (I), P.J.M. Greenslade, 9-3C (MCZ); 2 minor workers from 3.2 km NW of homestead on Plum Pudding track, Cravens Peak Station 23°19'00"S, 138°33'47", Queensland, 21-24 April 2007, C. Lemann, Malaise trap, gidgee flat, ANIC ANTS VIAL 76.104 [ANIC32-036867] (QM); 3 minor workers from Mt Gunson, South Australia, 1985, T. J. Case, 541 (SAM).

##### Other material examined.

**Queensland**: ‘Merigol’ (Beutel, T.), Bendidee Nat. Pk (Haines, J.). **South Australia**: 10 km E Mt Ive Homestead, Gawler Ranges (Greenslade, P.J.M.), Cambrai (Greenslade, P.J.M.), Cambrai (Greenslade, P.J.M.), Observatory Hill, Victoria Desert (Greenslade, P.J.M.). **Western Australia**: Black Swan Mine (Langlands, P./Osborne, J. [JDM32-004897]), Little Sandy Desert (Guthrie, N. A. [M145]).

##### Diagnosis.

*Melophorus pusillus* is placed in the *M. ludius* species-group on the basis of molecular data. However, in morphological appearance this taxon shares major diagnostic characters with the *M. biroi* complex (viz, metatibia of major worker with only one preapical spur; clypeal psammophore placed anteriorly at or just above anterior margin of clypeus in the minor worker and often in the major worker; legs compact, and small body size [HW of smallest minor < 0.40 mm, HW of largest major < 1.10 mm]). Like the other three members of its species-group, *M. pusillus* is characterised by being weakly sculptured overall, with the cuticle of the mesosoma visibly thin, the mesonotum being translucent to varying degrees and the mesopleuron either smooth or with vestigial sculpture only. *Melophorus pusillus* is distinguished from *M. ludius* by the larger eye (EI in 31-40 in *M. pusillus* compared with EI of 19-30 in *M. ludius*) and from *M. translucens* by the less elongate propodeum and shorter propodeal spiracle.

##### Minor worker description.

**Head.** Head approximately oval with straight sides; posterior margin of head strongly convex; frons shining with superficial shagreenation or microreticulation only; frons consisting exclusively or almost exclusively of well-spaced, appressed setae only (small, erect setae, if present, usually confined to ocular triangle or posterior margin of head). Eye large (eye length ≥ 0.50 × length of side of head capsule); in profile, eye set at about midpoint of head capsule; in profile, eye set around midline of head capsule; roughly ovoid, eye narrowed posteriad. In full-face view, frontal carinae distinctly concave; frontal lobes curved toward antennal insertion. Anteromedial clypeal margin straight; clypeal psammophore set at or above midpoint of clypeus; palp formula 6,4. Five mandibular teeth in minor worker; mandibles triangular, weakly incurved; third mandibular tooth distinctly shorter than apical tooth and teeth numbers two and four; masticatory margin of mandibles approximately vertical or weakly oblique. **Mesosoma.** Integument of pronotum, mesonotum and mesopleuron shining and mainly smooth, vestigial shagreenation most noticeable on humeri and mesopleuron; anterior mesosoma in profile weakly elevated anteriad, thereafter gently sinuate, pronotum and mesonotum on same plane; erect pronotal setae absent; in profile, metanotal groove a weak or vestigial furrow; propodeum shining and shagreenate; propodeum angulate, propodeal angle blunt; length ratio of propodeal dorsum to its declivity about1:1; erect propodeal setae always absent; appressed propodeal setulae short, separated by more than own length and inconspicuous; propodeal spiracle situated on or beside declivitous face of propodeum, and longer (length ≥ 0.50 × height of propodeum). **Petiole.** In profile, petiolar node squamiform; in full-face view, shape of petiolar node uniformly rounded; node shining and distinctly shagreenate-microreticulate. **Gaster.** Gaster shining with superficial microreticulation; pilosity of first gastral tergite consisting of well-spaced short, inconspicuous, appressed setae only, erect setae always absent. **General characters.** Colour pale yellow-orange to bright brown.

##### Major worker description.

**Head.** Head square; posterior margin of head planar or weakly convex; cuticle of frons shining and smooth except for piliferous pits; frons consisting exclusively or almost exclusively of well-spaced, appressed setae only (small, erect setae, if present, usually confined to ocular triangle or posterior margin of head). Eye moderate (eye length 0.20–0.49 length of head capsule); in full-face view, eyes set at about midpoint of head capsule; in profile, eye set anteriad of midline of head capsule; eyes elliptical, or roughly ovoid, eye narrowed posteriad. In full-face view, frontal carinae straight or weakly convex; frontal lobes straight in front of antennal insertion. Anterior clypeal margin broadly and evenly convex; clypeal psammophore set at or above midpoint of clypeus; palp formula 6,4. Five mandibular teeth in major worker; mandibles triangular, weakly incurved; third mandibular tooth distinctly shorter than apical tooth and teeth numbers two and four; masticatory margin of mandibles approximately aligned vertically or weakly oblique. **Mesosoma.** Integument of pronotum, mesonotum and mesopleuron moderately shining and shagreenate throughout; anterior mesosoma in profile broadly convex; erect pronotal setae absent; in profile, metanotal groove shallow, broadly V- or U-shaped; propodeum shining and finely striolate and microreticulate; propodeum smoothly rounded or with indistinct angle; propodeal dorsum and declivity confluent; erect propodeal setae absent; appressed propodeal setae sparse or absent, if present then not regularly spaced; propodeal spiracle situated on or beside declivitous face of propodeum, and shorter (length less than 0.50 × height of propodeum). **Petiole.** In profile, petiolar node squamiform; in full-face view, shape of petiolar node tapered with sharp vertex; node shining and smooth with vestigial microreticulation anteriad. **Gaster.** Gaster shining, shagreenate (‘LP record’ appearance); pilosity of first gastral tergite consisting of well-spaced, erect and semi-erect setae interspersed with regularly spaced appressed setae, or consisting of well-spaced short, inconspicuous, appressed setae, erect setae (present in at least some workers) confined to margin of the sclerite. **General characters.** Colour yellow ochre with brownish-yellow gaster to pale brown.

##### Measurements.

Worker (n = 6): CI 90–106; EI 31–40; EL 0.15–0.21; HL 0.41–0.64; HW 0.36–0.68; ML 0.52–0.86; MTL 0.30–0.52; PpH 0.05–0.08; PpL 0.22–0.37; SI 99–127; SL 0.46–0.67.

##### Comments.

*Melophorus pusillus* resembles nothing so much as a slightly elongate *M. ludius*, and is easily confused with that ant. The larger eye (EI 31-40 compared with EI 19-30) and the very thin cuticle on the mesosoma are the best way to distinguish this ant from *M. ludius*. The species is widely distributed in Australia, mainly in arid inland environments, and has been recorded from all mainland states except the ACT. Sequencing of one specimen makes it sister to *M. potteri* in both a five-gene and a three-gene tree. However, *M. potteri* has many synapomorphies (e.g., mandible, palp formula, appearance of the propodeal spiracle, etc.) not shared by *M. pusillus*, and the association of the two taxa (both on very long branches) may be due to long-branch attraction. Workers collected in Cambrai, SA were taken in dunes and two workers were collected in a malaise trough at Craven Peak in QLD, but no other ecological data are available.

##### Etymology.

Latin *pusillus* (‘puny’); adjective in the nominative singular.

**Figure 93.**
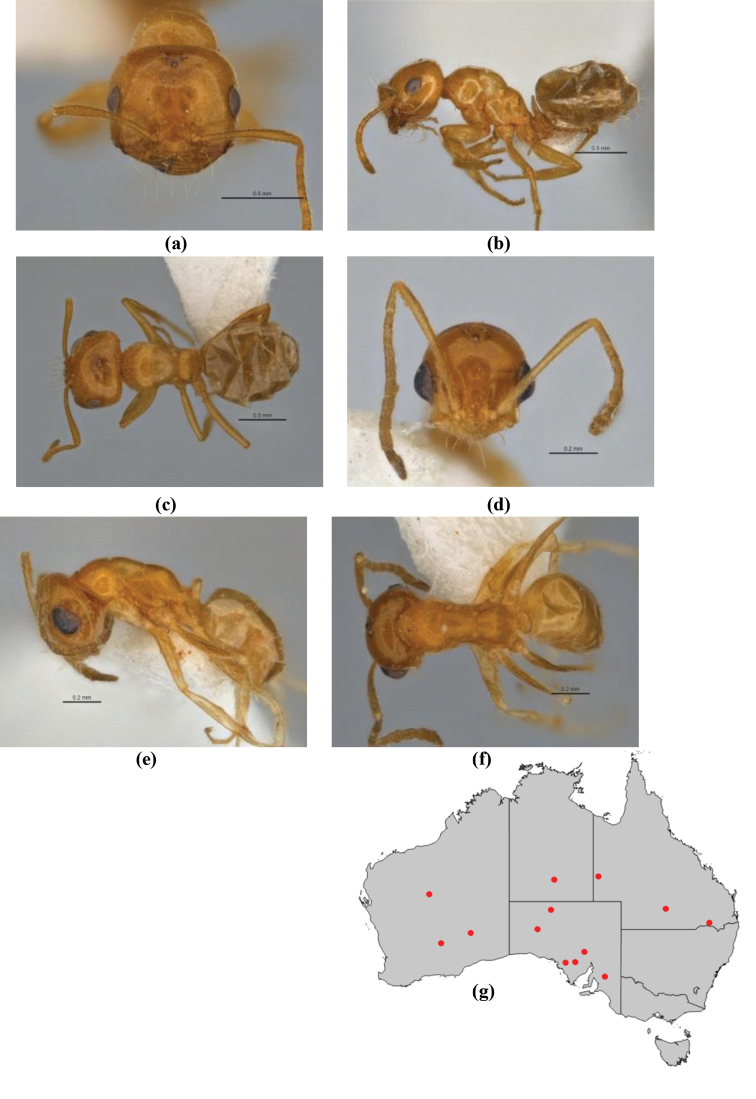
*Melophorus pusillus* sp. n.: major worker paratype (JDM32-004668–top ant) frons (**a**), profile (**b**) and dorsum (**c**); minor worker holotype (JDM32-004668–bottom ant) frons (**d**), profile (**e**) and dorsum (**f**); distribution map for the species (**g**). Low resolution scale bars: 0.5 mm (**a–c**); 0.2 mm (**d–f**).

#### Melophorus translucens

Taxon classificationAnimaliaHymenopteraFormicidae

Heterick, Castalanelli & Shattuck sp. n.

http://zoobank.org/ACFD054F-0159-48DD-82E1-F326D5216201

##### Types.

Holotype minor worker (bottom ant) from Swanbourne, Western Australia, 30 September 1987, B. Heterick, soil, native vegetation, urban dune, 295, 8*Mel*BH21 [JDM32-001966]. Paratypes: Major worker on same pin and with same details as holotype (WAM); minor worker and two major workers from Bold Park 31°56'S, 115°46'E, Western Australia, 28 March 2002, P. Achour, pitfall trap, coastal woodland [JDM32-001968] (WAM); minor worker from near Perth, Western Australia, 1978, M. Rossbach, A1387C83 [JDM32-001973] (ANIC); minor worker from near Perth, Western Australia, 1978, M. Rossbach, A1387C83 [JDM32-001973] (MCZ).

##### Other material examined.

**Western Australia**: Christmas Tree Well (Heterick, B.E. [M17/M53/M54]), Guilderton (Heterick, B.E. [JDM32-001970]).

##### Diagnosis.

*Melophorus translucens* is placed in the *M. ludius* species-group on the basis of molecular data. However, in morphological appearance this taxon shares major diagnostic characters with the *M. biroi* complex (viz, metatibia of major worker with only one preapical spur; clypeal psammophore placed anteriorly at or just above anterior margin of clypeus in the minor worker and often in the major worker; legs compact, and small body size [HW of smallest minor < 0.40 mm, HW of largest major < 1.10 mm]). Like the other three members of its species-group, *M. translucens* is characterised by being weakly sculptured overall, with the cuticle of the mesosoma visibly thin, the mesonotum being translucent to varying degrees and the mesopleuron either smooth or with vestigial sculpture only. In *Melophorus ltranslucens* the mesonotum is conspicuously translucent and its pale appearance often contrasts with the much more intense colour of the rest of the mesosoma (although some minor workers can be generally pale). *Melophorus translucens* is distinguished from both *M. ludius* and *M. pusillus* by its more elongate propodeum and propodeal spiracle.

##### Minor worker description.

**Head.** Head rectangular; posterior margin of head planar or weakly concave; frons shining with superficial shagreenation or microreticulation only; frons consisting exclusively or almost exclusively of well-spaced, appressed setae only (small, erect setae, if present, usually confined to ocular triangle or posterior margin of head). Eye moderate (eye length 0.20–0.49 length of side of head capsule); in full-face view, eyes set above midpoint of head capsule, or set at about midpoint of head capsule; in profile, eye set anteriad of head capsule; eyes elliptical or slightly reniform. In full-face view, frontal carinae concave; frontal lobes straight in front of antennal insertion. Anteromedial clypeal margin broadly and evenly convex; clypeal psammophore set at or just above anterior clypeal margin; palp formula 6,4. Five mandibular teeth in minor worker; mandibles triangular, weakly incurved; third mandibular tooth distinctly shorter than apical tooth and teeth numbers two and four; masticatory margin of mandibles approximately vertical or weakly oblique. **Mesosoma.** Integument of pronotum, mesonotum and mesopleuron shining and mainly smooth, vestigial shagreenation most noticeable on humeri and mesopleuron; anterior mesosoma in profile pronotum smoothly rounded anteriad and flattened posteriad, mesonotum narrowly convex, or smoothly rounded anteriad, thereafter pronotum and whole of mesonotum flattened and on same plane as propodeum; appearance of erect pronotal setae short, (i.e., longest erect setae shorter than length of eye) and unmodified, or erect pronotal setae absent; in profile, metanotal groove shallow, indicated mainly by an angle; propodeum shining and shagreenate; propodeum smoothly rounded or with indistinct angle; propodeal dorsum and declivity confluent; erect propodeal setae always absent; appressed propodeal setulae sparse or absent, if present then not regularly spaced; propodeal spiracle situated on or beside declivitous face of propodeum, and longer (length ≥ 0.50 × height of propodeum). **Petiole.** In profile, petiolar node squamiform; in full-face view, shape of petiolar node uniformly rounded; node shining and smooth with vestigial sculpture. **Gaster.** Gaster shining, shagreenate (‘LP record’ appearance); pilosity of first gastral tergite consisting of well-spaced, erect and semi-erect setae interspersed with regularly placed appressed setae, or consisting of well-spaced short, inconspicuous, appressed setae only, erect setae always absent. **General characters.** Colour of head, pronotum, propodeum orange or orange tan to brown, mesonotum translucent cream-yellow to yellow, gaster dark brown, appendages variably yellowish to brownish.

##### Major worker description.

**Head.** Head square; posterior margin of head planar or weakly concave; cuticle of frons shining with superficial shagreenation or microreticulation only; frons consisting exclusively or almost exclusively of well-spaced, appressed setae only (small, erect setae, if present, usually confined to ocular triangle or posterior margin of head). Eye moderate (eye length 0.20–0.49 length of head capsule); in full-face view, eyes set above midpoint of head capsule; in profile, eye set anteriad of midline of head capsule; eyes elliptical. In full-face view, frontal carinae concave; frontal lobes straight in front of antennal insertion. Anterior clypeal margin straight; clypeal psammophore set at or just above anterior clypeal margin; palp formula 6,4. Five mandibular teeth in major worker; mandibles triangular, weakly incurved; third mandibular tooth distinctly shorter than apical tooth and teeth numbers two and four; masticatory margin of mandibles approximately aligned vertically or weakly oblique. **Mesosoma.** Integument of pronotum, mesonotum and mesopleuron shining with indistinct microsculpture that is most pronounced on lower surfaces; anterior mesosoma in profile pronotum smoothly rounded anteriad and flattened posteriad, mesonotum narrowly convex; erect pronotal setae short, (i.e., shorter than length of eye) and unmodified; in profile, metanotal groove shallow, indicated mainly by an angle and metathoracic spiracles; propodeum shining and microreticulate; propodeum always smoothly rounded; propodeal dorsum and declivity confluent; erect propodeal setae absent; appressed propodeal setae sparse or absent, if present then not regularly spaced; propodeal spiracle situated on or beside declivitous face of propodeum, and longer (length ≥ 0.50 × height of propodeum). **Petiole.** In profile, petiolar node squamiform; in full-face view, shape of petiolar node tapered with squared-off vertex; node shining and smooth with vestigial microreticulation anteriad. **Gaster.** Gaster shining, shagreenate (‘LP record’ appearance); pilosity of first gastral tergite consisting of thick, appressed setae that form pubescence, interspersed with numerous short, bristly, erect setae. **General characters.** Colour of foreparts (excluding mesonotum) orange to orange tan, mesonotum yellow, gaster brown to dark brown.

##### Measurements.

Worker (n = 4): CI 69–111; EI 20–36; EL 0.12–0.15; HL 0.46–0.69; HW 0.32–0.77; ML 0.59–0.90; MTL 0.34–0.48; PpH 0.05–0.07; PpL 0.27–0.43; SI 74–149; SL 0.47–0.57.

##### Comments.

This species appears to be confined to white sand, with all bar one of the populations sampled occurring on the Swan Coastal Plain, WA (one collection was taken at Christmas Tree Well in the Darling Range). All of these samples have been collected in the vicinity of Perth but the ant probably has a broader distribution in suitable habitats. A nest examined by the principal author in white sand-dunes near Swanbourne Beach, Perth, WA was situated among low, coastal shrubs. The depigmented, almost transparent mesosoma (quite bulbous in the major worker) and obliquely elongate propodeal spiracle serve to distinguish it from most other ants, although pale forms of the tiny minor worker can be difficult to differentiate from those of *M. pusillus*. In such cases the smaller size of the eye in *M. translucens* is a useful diagnostic character. Minor workers can be colour variable, ranging from a depigmented yellow-and-white to dark brown-and-white, the depigmented mesonotum contrasting spectacularly with the rest of the mesosoma in the latter.

Given its generally unremarkable appearance, this ant appears to be surprisingly primitive. In both the bar-coding COI and the five-gene tree the species strongly assorts with *hirsutus* and, more broadly, forms with that latter species a sister-group relationship with *potteri* and *pusillus*, but falls within these latter on the three-gene tree. However, the species is retained in the *ludius* complex for now, based on its morphology, though it may eventually require separate complex or even species-group status. The species seems to have evolved earlier than the *M. biroi* species-group, based on branch length, and its current localized distribution may indicate a relictual status.

Apart from its being apparently restricted to white, sandy soils, nothing more is known about this ant.

##### Etymology.

Latin *trans* (‘across’, ‘through’) plus *lucens* (‘shining’); adjective in the nominative singular.

**Figure 94.**
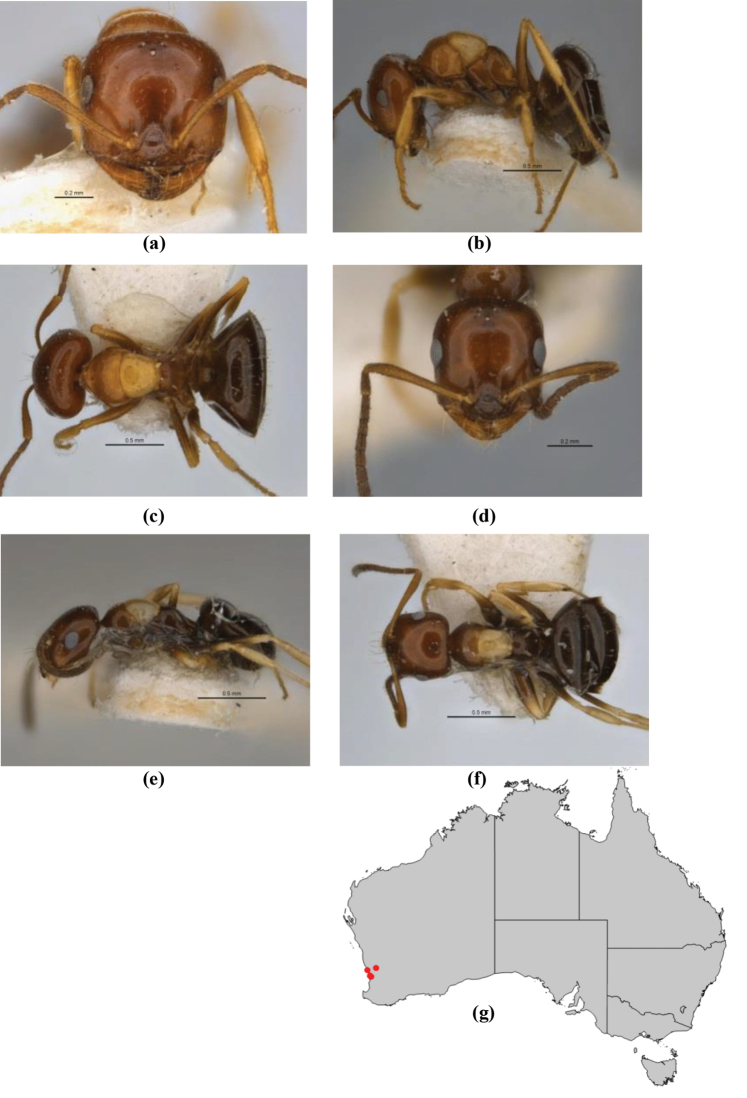
*Melophorus translucens* sp. n.: major worker paratype (JDM32-001966–top ant) frons (**a**), profile (**b**) and dorsum (**c**); minor worker holotype (JDM32-001966–bottom ant) frons (**d**), profile (**e**) and dorsum (**f**); distribution map for the species (**g**). Low resolution scale bars: 0.5 mm (**b, c**, **e, f**); 0.2 mm (**a, d**).

### *Melophorus majeri* species-group

The *Melophorus majeri* species-group consists of one very aberrant small species in which the horse-headed minor worker differs in spectacular fashion from any other *Melophorus* with its propodeal spines, its highly carinate mesosoma and its enormously elongate antennal scape that resembles the antenna of some pselaphid beetles. The major worker, however, is much less striking, although it retains propodeal denticles. The modifications may be life-style related, but, unfortunately, almost nothing is known of this very rare ant apart from the fact that it appears to be restricted to a few white sand-dune habitats in southwest WA. No specimens of this species-group were available for sequencing.

### Melophorus majeri

Taxon classificationAnimaliaHymenopteraFormicidae

Agosti

Melophorus majeri Agosti 1997: 166, figs 6–9. Types. Holotype minor and paratype major workers Hassel Road, Jerramungup, Western Australia [AMNH, ANIC, CASC] (examined: ANIC holotype (second ant on pin of three workers) and paratypes CASENT0172019, CASENT017020, AntWeb images of AMNH (CASENT0104674) and CASC (CASENT0173922, CASENT0173922) specimens). The AntWeb Automontage image for the holotype erroneously depicts a paratype major worker from the same pin as the holotype. specimens , Note. Syntype label also erroneously displayed for CASENT0104674 (paratype).

#### Other material examined.

**Western Australia**: Eneabba-Leeman Rd. (Dunn, R. [JDM32-001553]), Mettler Lake Rd. (15 km SW of Wellstead) (Heterick, B.E. [JDM32-001552]).

#### Diagnosis.

The armed propodeum in all worker sub-castes, the flattened and laterally carinate pronotum and mesonotum in the minor worker, the raised antennal lobes, and the absence of a metatibial apical spur distinguish *Melophorus majeri* from all other *Melophorus* and cause it to be placed in its own, unique species-group.

#### Minor worker description.

**Head.** Head elongate, truncate posteriad; posterior margin of head planar to strongly convex; frons matt or with weak sheen, microreticulate or microreticulate-shagreenate; frons consisting exclusively or almost exclusively of well-spaced, appressed setae only (small, erect setae, if present, usually confined to ocular triangle or posterior margin of head). Eye small (eye length less than 0.2 × length of side of head capsule); in full-face view, eyes set above midpoint of head capsule; in profile, eye set around midline of head capsule; eyes elliptical or slightly reniform. In full-face view, frontal carinae straight or weakly convex; frontal lobes straight, elevated. Anteromedial clypeal margin narrowly convex and protruding, clypeal margin entire or very weakly indented; clypeal psammophore set at or just above anterior clypeal margin; palp formula 6,4. Five to six mandibular teeth in minor worker; mandibles triangular, weakly incurved; third mandibular tooth distinctly shorter than apical tooth and teeth numbers two and four; masticatory margin of mandibles approximately vertical or weakly oblique. **Mesosoma.** Integument of pronotum, mesonotum and mesopleuron matt or with weak sheen and microreticulate throughout; anterior mesosoma in profile gently undulate, dorsal and lateral surfaces separated by carinae; erect pronotal setae absent; in profile, metanotal groove a weak or vestigial furrow; propodeum matt or with a weak sheen and microreticulate; propodeum distinctly angulate, propodeal angles produced as short, erect denticles; length ratio of propodeal dorsum to its declivity about 3:2; erect propodeal setae always absent; appressed propodeal setulae short, separated by more than own length and inconspicuous; propodeal spiracle situated at least twice its width from the declivitous face of propodeum, and longer (length ≥ 0.50 × height of propodeum). **Petiole.** In profile, petiolar node trapezoidal with sharply defined flange posteriad; in full-face view, shape of petiolar node tapered with squared-off vertex; node matt and microreticulate. **Gaster.** Gaster matt with distinct microreticulate pattern; pilosity of first gastral tergite consisting of well-spaced short, inconspicuous, appressed setae only, erect setae always absent. **General characters.** Colour blackish-brown.

#### Major worker description.

***Head.*** Head square; posterior margin of head planar or weakly concave; cuticle of frons matt or with weak sheen, microreticulate; frons consisting exclusively or almost exclusively of well-spaced, appressed setae only (small, erect setae, if present, usually confined to ocular triangle or posterior margin of head). Eye small (eye length less than 0.2 × length of head capsule); in full-face view, eyes set above midpoint of head capsule; in profile, eye set anteriad of midline of head capsule; eyes elliptical. In full-face view, frontal carinae straight or weakly convex; frontal lobes straight, elevated. Anterior clypeal margin convex, acuminate anteromedially, margin entire; clypeal psammophore set at or just above anterior clypeal margin; palp formula 6,4. Five mandibular teeth in major worker; mandibles triangular, weakly incurved; third mandibular tooth distinctly shorter than apical tooth and teeth numbers two and four; masticatory margin of mandibles approximately aligned vertically or weakly oblique. **Mesosoma.** Integument of pronotum, mesonotum and mesopleuron matt or with weak sheen and microreticulate throughout; anterior mesosoma in profile gently sinuous after initial steep pronotal incline; erect pronotal setae short, (i.e., shorter than length of eye) and unmodified; in profile, metanotal groove a narrow but deep slit; propodeum matt or with a weak sheen and microreticulate; propodeum distinctly angulate, propodeal angles produced as short denticles; length ratio of propodeal dorsum to its declivity between 1:1 and 1:2; erect propodeal setae absent; appressed propodeal setae short, separated by more than own length and inconspicuous; propodeal spiracle situated nearer to midpoint of propodeum than to its declivitous face, and shorter (length less than 0.50 × height of propodeum). **Petiole.** In profile, petiolar node squamiform; in full-face view, shape of petiolar node generally rounded with median indentation; node matt, and microreticulate. **Gaster.** Gaster matt with a distinct microreticulate pattern; pilosity of first gastral tergite consisting of well-spaced, erect and semi-erect setae interspersed with regularly spaced appressed setae. **General characters.** Colour generally dark brown, head brown.

#### Measurements.

Worker (n = 2): CI 70-102; EI 21-30; EL 0.14-0.19; HL 0.65-0.89; HW 0.46-0.91; ML 0.92-0.24; MTL 0.44-0.64; PpH 0.09-0.14; PpL 0.32-0.47; SI 96-223; SL 0.88-1.01

#### Comments.

This enigmatic species can be distinguished from all other *Melophorus* by reason of its armed propodeum in all worker subcastes. Other apomorphies include the absent metatibial apical spur (shared only with the unrelated *M. anderseni* group and *M. fulvidus*) and the peculiarly flattened and carinate mesosoma in the minor worker, hence our tentative placement of *M. majeri* in its own species-group. The affinities of this ant are unknown, as no specimens were available for sequencing, but the appearance of the major worker suggests it may be close to the *M. biroi* species-group, if not a very aberrant member of that group. The only known specimens (one nest series and a couple of strays) have come from white sand in southwestern WA. The minor worker, in particular, has more than a suggestion of being a mimic of a pselaphid beetle inquiline, with an extraordinary antennal scape that becomes incrassate distally and a flattened mesosoma. Could this be a kleptoparasite or subterranean nest raider of other ants? However that may be, minor workers are also active on the soil surface and have been collected as foragers. The nest series was associated with a piece of wood (Agosti 1997).

**Figure 95.**
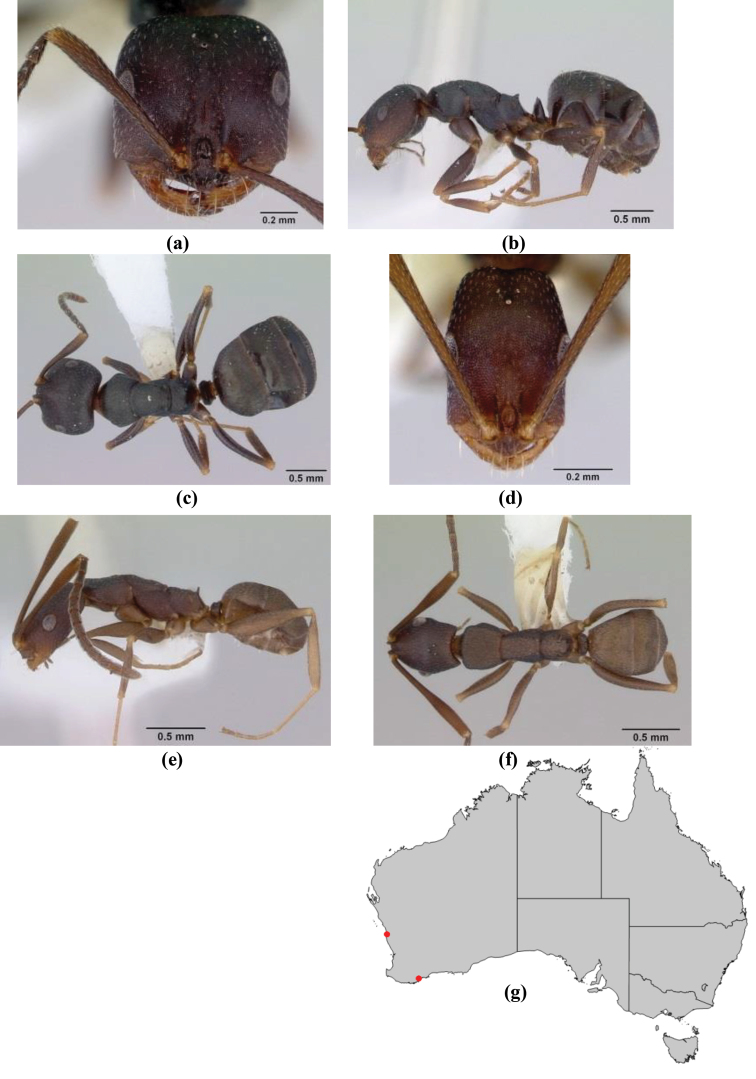
*Melophorus majeri* Agosti: ANIC major worker paratype (CAS0172019) frons (**a**), profile (**b**) and dorsum (**c**); ANIC minor worker holotype (CAS0172020–second ant on pin) frons (**d**), profile (**e**) and dorsum (**f**); distribution map for the species (**g**).

### *Melophorus potteri* species-group

This small group of three species includes at least one termite raider. Whereas other specialized carnivorous groups within the genus appear to be related to the *M. fieldi* species-group, if not actually atypical members of that group, the *M. potteri* species-group aligns more closely with *M. aeneovirens* in appearance. The mandibles are highly modified in two of the species, and this may relate to their specialized lifestyle.

### Melophorus macroschismus

Taxon classificationAnimaliaHymenopteraFormicidae

Heterick, Castalanelli & Shattuck sp. n.

http://zoobank.org/36E58E70-2BB9-41F4-9DE4-395BE9584F50

#### Types.

Holotype minor worker from near Warrachuppin 31°02'33"S, 118°46'23"E, Western Australia, April 2007, R. Harris, ant nest sample, salt affected remnant, T1Q5 [JDM32-001578] (WAM). Paratypes: 2 minor workers from 6 miles W of Yellowdine, Western Australia, 2 October 1947, T. Greaves, 7739, only specimens [ANIC32 900028] (ANIC); 2 minor workers from Queen Victoria Spring Nature Reserve 30°22'S, 123°37'E, Western Australia, December 1989, CALM, Pitfall trap, sandplain F2 [JDM32-001537] (WAM).

#### Diagnosis.

*Melophorus macroschismus* is one of three members of the *M. potteri* species-group. Like the other two species, *M. macrochismus* has a large, oblique propodeal spiracle situated well before the declivitous face of propodeum, the spiracle bisecting much of the propodeum. This species is distinguished from the remaining members of the group by a combination of its palp formula (PF 6,4), its matt general appearance, its oval head capsule, the broadly convex but not protrusive anterior clypeal margin, and the conventional appearance of the mandible (five distinct teeth) as opposed to the aberrant dentition of the other two species in the group.

#### Minor worker description.

**Head.** Head approximately oval with straight sides; posterior margin of head planar or weakly convex; frons matt or with weak sheen, microreticulate or microreticulate-shagreenate; frons consisting exclusively or almost exclusively of well-spaced, appressed setae only (small, erect setae, if present, usually confined to ocular triangle or posterior margin of head). Eye moderate (eye length 0.20–0.49 length of side of head capsule); in full-face view, eyes set above midpoint of head capsule; in profile, eye set anteriad of midline of head capsule; eyes elliptical or slightly reniform, or roughly ovoid, eye narrowed posteriad. In full-face view, frontal carinae straight or weakly convex; frontal lobes straight in front of antennal insertion. Anteromedial clypeal margin broadly and evenly convex; clypeal psammophore set at or just above anterior clypeal margin; palp formula 6,4. Five mandibular teeth in minor worker; mandibles triangular, weakly incurved; third mandibular tooth distinctly shorter than apical tooth, but equivalent in length to remaining teeth; masticatory margin of mandibles approximately vertical or weakly oblique. **Mesosoma.** Integument of pronotum, mesonotum and mesopleuron matt or with weak sheen and microreticulate throughout; anterior mesosoma in profile smoothly rounded anteriad, thereafter pronotum and whole of mesonotum flattened and on same plane as propodeum; erect pronotal setae absent; in profile, metanotal groove shallow, broadly V or U-shaped; propodeum matt or with a weak sheen and microreticulate; propodeum angulate, propodeal angle blunt; length ratio of propodeal dorsum to its declivity about 4:3; erect propodeal setae always absent; appressed propodeal setulae short, separated by more than own length and inconspicuous; propodeal spiracle situated nearer to midpoint of propodeum than to its declivitous face, and longer (length ≥ 0.50 × height of propodeum). **Petiole.** In profile, petiolar node squamiform; in full-face view, shape of petiolar node uniformly rounded; node shining and distinctly shagreenate-microreticulate. **Gaster.** Gaster weakly shining with indistinct shagreenation; pilosity of first gastral tergite consisting of well-spaced short, inconspicuous, appressed setae only, erect setae always absent. **General characters.** Colour of foreparts light tan or orange-brown, gaster brown.

#### Measurements.

Worker (n = 6): CI 92–95; EI 28–30; EL 0.21–0.22; HL 0.75–0.83; HW 0.68–0.79; ML 1.06–1.18; MTL 0.57–0.63; PpH 0.12–0.14; PpL 0.45–0.49; SI 91–95; SL 0.65–0.72.

#### Comments.

Apart from the diagnostic appearance of the propodeal spiracle, the morphology of the minor worker is unremarkable compared with other *Melophorus*, and the habits of this species may be generalized. The major worker is unknown. No sequencing has been carried out on *M. macroschismus*, which is represented by a few minor workers only from localities in the eastern wheatbelt and eastern goldfields, Western Australia and from Wilgena Stn, SA (TERC). The only available ecological data comes from a label for a specimen collected near Warrachupin, WA, which was taken from a nest in salt infected remnant bushland.

#### Etymology.

Latinized Greek *macros* (Greek *makros* ‘long’) plus *schismus* (Greek *schism* ‘cleft’); adjective in the nominative singular.

**Figure 96.**
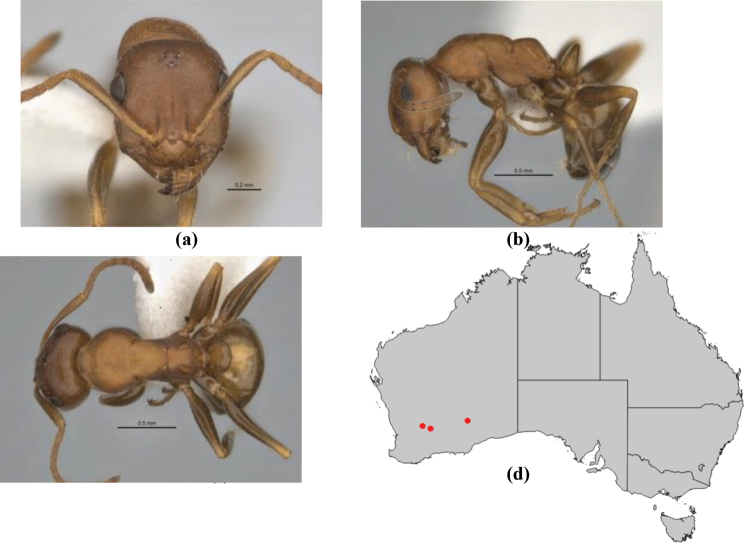
*Melophorus macroschismus* sp. n.: minor worker holotype (JDM32-001578) frons (**a**), profile (**b**) and dorsum (**c**); distribution map for the species (**d**). Low resolution scale bars: 0.5 mm (**b, c**); 0.2 mm (**a**).

### Melophorus pelecygnathus

Taxon classificationAnimaliaHymenopteraFormicidae

Heterick, Castalanelli & Shattuck sp. n.

http://zoobank.org/86CC5032-2E36-4F85-B0FB-F7151E39FC3C

#### Types.

Holotype minor worker (top ant) from 97.6 km SE of Newman 23°52'46"S, 120°30'12"E, Western Australia, April, 1997, S. van Leeuwen & R. N. Bromilow, Perm. invert. pitfall trap S5, Sandstone breakaway [JDM32-001575] (WAM). Paratypes: minor worker on same pin and with same details as holotype (WAM); major and minor from 142 km SSE of Newman 24°31'46"S, 120°17'45"E, Western Australia, August 1997, S. van Leeuwen & R. N. Bromilow, Perm. invert., pitfall trap B4, Spinifex sandplain [JDM32-001576] (ANIC); minor worker from Gawler Ranges, South Australia, 5 October 1972, P.J.M. Greenslade, (3), ‘*Melophorus* sp. 1 loan ANIC 1991’ [ANIC32 900030] (MCZ); major and media worker from Kwelkan 31°08'36"S, 117°59'43"E, Western Australia, 12 March 2006, M. Russell, Hand-collected, grid survey [JDM32-001574] (WAM);.

#### Other material examined.

**Queensland**: ‘Gumbardo’ (Beutel, T.), ‘Merigol’ (Beutel, T.). **South Australia**: Mt Victor (Greenslade, P.J.M. [ANIC32-900029]).

#### Diagnosis.

*Melophorus pelecygnathus* is one of three members of the *M. potteri* species-group. These three species have a large, oblique propodeal spiracle situated well before declivitous face of propodeum, the spiracle bisecting much of the propodeum. This species is distinguished from the remaining members of the group by the combination of its reduced palp formula (PF 3,4) and its large, securiform mandible whose masticatory margin is virtually edentate with only minute (often worn), evenly sized teeth except for the long apical tooth.

#### Minor worker description.

**Head.** Head rectangular; posterior margin of head planar to strongly convex; frons shining with superficial shagreenation or microreticulation only; frons consisting exclusively or almost exclusively of well-spaced, appressed setae only (small, erect setae, if present, usually confined to ocular triangle or posterior margin of head). Eye moderate (eye length 0.20–0.49 length of side of head capsule); in full-face view, eyes set above midpoint of head capsule; in profile, eye set anteriad of midline of head capsule; eye roughly ovoid, narrowed posteriad. In full-face view, frontal carinae straight, convergent posteriad; frontal lobes curved inward in front of antennal insertion. Anteromedial clypeal margin straight; clypeal psammophore set at or just above anterior clypeal margin; palp formula 3,4. Mandibular teeth in minor worker absent, mandible edentate or with small crenulations only; mandibles securiform; third mandibular tooth absent; masticatory margin of mandibles approximately vertical or weakly oblique. **Mesosoma.** Integument of pronotum, mesonotum and mesopleuron with weak to moderate sheen, shagreenate on pronotum and dorsum of mesonotum, otherwise microreticulate; anterior mesosoma in profile pronotum smoothly rounded anteriad and flattened posteriad, mesonotum narrowly convex; erect pronotal setae absent; in profile, metanotal groove shallow, broadly V or U-shaped; propodeum matt or with a weak sheen and microreticulate; propodeum smoothly rounded or with indistinct angle, or angulate, propodeal angle blunt; length ratio of propodeal dorsum to its declivity about 4:3; erect propodeal setae always absent; appressed propodeal setulae short, separated by more than own length and inconspicuous; propodeal spiracle situated nearer to midpoint of propodeum than to its declivitous face, and longer (length ≥ 0.50 × height of propodeum). **Petiole.** In profile, petiolar node narrowly conical, vertex blunt, directed posteriad, or subcuboidal, vertex bluntly rounded; in full-face view, shape of petiolar node uniformly rounded; node shining and distinctly shagreenate-microreticulate, or matt with indistinct microsculpture. **Gaster.** Gaster shining with superficial microreticulation, or shining, shagreenate (‘LP record’ appearance); pilosity of first gastral tergite consisting of well-spaced short, inconspicuous, appressed setae only, erect setae always absent. **General characters.** Colour bicoloured with reddish to chocolate head, orange- or reddish-brown mesosoma and dark brown gaster.

#### Major worker description.

**Head.** Head square; posterior margin of head planar or weakly concave; cuticle of frons shining and smooth except for piliferous pits; frons consisting exclusively or almost exclusively of well-spaced, appressed setae only (small, erect setae, if present, usually confined to ocular triangle or posterior margin of head). Eye small (eye length less than 0.2 × length of head capsule); in full-face view, eyes set above midpoint of head capsule; in profile, eye set anteriad of midline of head capsule; eye roughly ovoid, narrowed posteriad. Frontal carinae straight, convergent posteriad; frontal lobes curved inward in front of antennal insertion. Anterior clypeal margin straight; clypeal psammophore set at or just above anterior clypeal margin; palp formula 3,4. Mandibular teeth in major worker absent, mandible edentate or with small crenulations only; mandibles securiform; third mandibular tooth absent; masticatory margin of mandibles approximately aligned vertically or weakly oblique. **Mesosoma.** Integument of pronotum, mesonotum and mesopleuron with weak to moderate sheen, shagreenate on pronotum and dorsum of mesonotum, otherwise microreticulate; anterior mesosoma in profile pronotum smoothly rounded anteriad and flattened posteriad, mesonotum narrowly convex, or gently sinuous after initial steep pronotal incline; erect pronotal setae absent; in profile, metanotal groove shallow, broadly V- or U-shaped; propodeum shining and microreticulate; propodeum always smoothly rounded; propodeal dorsum and declivity confluent; erect propodeal setae absent; appressed propodeal setae short, separated by more than own length and inconspicuous; propodeal spiracle situated nearer to midpoint of propodeum than to its declivitous face, and longer (length ≥ 0.50 × height of propodeum), or situated nearer to midpoint of propodeum than to its declivitous face, and shorter (length less than 0.50 × height of propodeum). **Petiole.** In profile, petiolar node subcuboidal, vertex bluntly rounded; in full-face view, shape of petiolar node square with rounded angles; node shining and faintly shagreenate-microreticulate. **Gaster.** Gaster shining, shagreenate (‘LP record’ appearance); pilosity of first gastral tergite consisting of well-spaced short, inconspicuous, appressed setae only, erect setae always absent. Colour of foreparts orange to crimson, gaster chocolate.

#### Measurements.

Worker (n = 4): CI 103–104; EI 22–24; EL 0.22–0.31; HL 0.89–1.35; HW 0.92–1.40; ML 1.18–1.75; MTL 0.66–0.97; PpH 0.18–0.28; PpL 0.53–0.81; SI 73–74; SL 0.68–1.03.

#### Comments.

*Melophorus pelecygnathus* is a spectacular species with large, securiform (hatchet-shaped) mandibles in both the minor and major workers. The ant cannot be mistaken for any other *Melophorus*. The species has been recorded from at least NSW (TERC), QLD (QM), (TERC), SA and WA, and features in Fig. 15f in Greenslade 1979. The nature of the mandibles indicates that this ant is likely specialized for hunting particular prey, whether this is termites (as assumed by Andersen, 2007) or some other arthropod. Unfortunately, available labels do not provide any information on the ant apart from the necessary basics of place, date and collector.

#### Etymology.

Compound of Latinized Greek *pelecys* (Greek *pelekys* ‘hatchet’) plus Neo-Latin *gnathus* (‘jaw’); noun in the nominative singular standing in apposition to the generic name.

**Figure 97.**
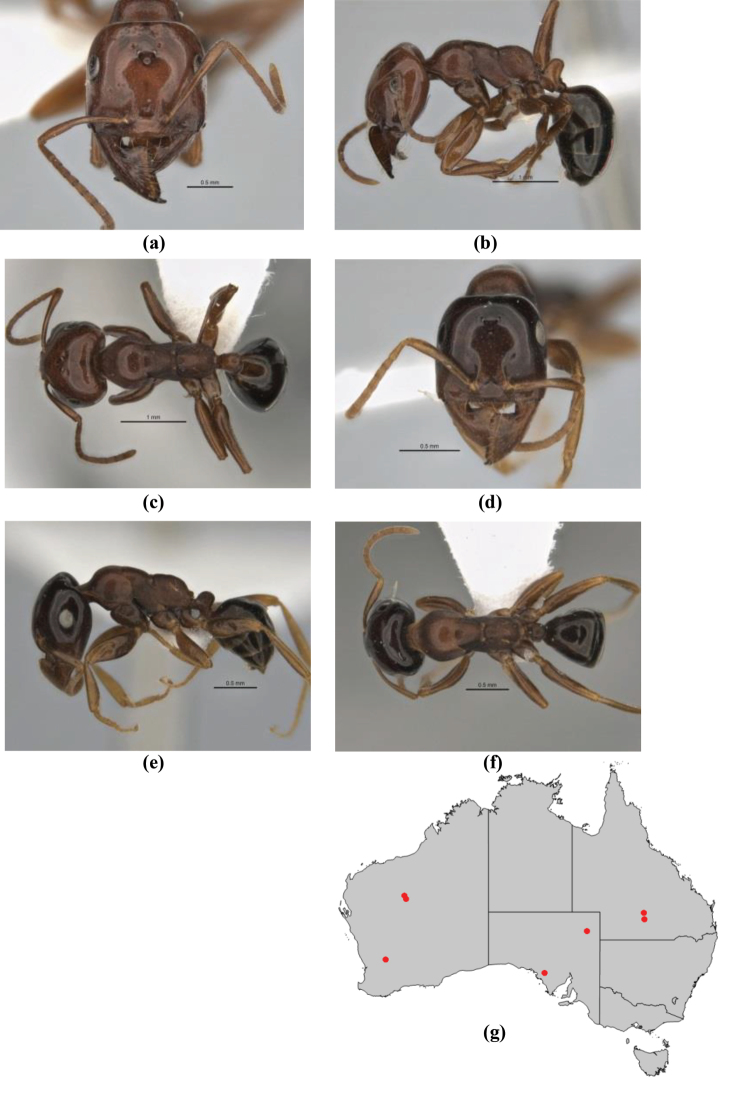
*Melophorus pelecygnathus* sp. n.: major worker paratype (JDM32-001574–bottom ant) frons (**a**), profile (**b**) and dorsum (**c**); minor worker holotype (JDM32-001575–top ant) frons (**d**), profile (**e**) and dorsum (**f**); distribution map for the species (**g**). Low resolution scale bars: 1 mm (**b, c**); 0.5 mm (**a, d–f**).

### Melophorus potteri

Taxon classificationAnimaliaHymenopteraFormicidae

McAreavey

Melophorus potteri McAreavey 1947: 25, fig. 1. Types. Syntype worker and queen: Patho, Victoria [MV] (examined: MV specimens T-11552, T-21872).

#### Other material examined.

**New South Wales**: 40 mi N Warren (Lowery, B.B.). **Northern Territory**: 15 km SE Alice Springs (Davidson, D. & Morton, S.), Lasseter Cave (Heatwole), Tanami (Greenslade, P.J.M.). **Queensland**: ‘Gumbardo’ (Beutel, T.), ‘Merigol’ (Beutel, T.), Sandringham (Greenslade, P.J.M.), St. George (Lowery, B.B.), Thallon (Lowery, B.B.). **Victoria**: Patho (Potter, H.A. [ANIC32-053439]), Patho (Potter, H.), Patho (Potter, H.A.), Patho (Potter, H.). **Western Australia**: 26 mi SSE Karonie (Taylor, R.W.), Pindar (Mercovich, C.). **Western Australia**: 12 km W of West Arthur (Jacobs, M. [JDM32-001571]), 18.5 km E Southern Cross (Heterick, B.E. [M135]), 19 km N Hines Hill (Heterick, B.E. [M07/M37/M38]), 74 km EbyN Cosmo Newberry (Feehan, J.E.), Eneabba (Bisevac, L. & Heterick, B.E. [JDM32-001572]), Ethel Creek (Varris, P.A. [JDM32-001573]), Rabbit Proof Fence Rd. (Heterick, B.E. [JDM32-004664]), Ravensthorpe (Lowery, B.B.).

#### Diagnosis.

*Melophorus potteri* is one of three members of the *M. potteri* species-group. Like the other two species, *M. potteri* has a large, oblique propodeal spiracle situated well before the declivitous face of propodeum, the spiracle bisecting much of the propodeum. This species is distinguished from the remaining members of the group by a combination of its glossy general appearance, its reduced palp formula (PF 3,4), its square or rectangular head capsule, the square protrusion of the anterior clypeal margin and the distally expanded basal margin of the mandible.

#### Minor worker description.

**Head.** Head rectangular; posterior margin of head planar to strongly convex; frons shining and smooth except for piliferous pits; frons consisting exclusively or almost exclusively of well-spaced, appressed setae only (small, erect setae, if present, usually confined to ocular triangle or posterior margin of head). Eye moderate (eye length 0.20–0.49 length of side of head capsule); in full-face view, eyes set at about midpoint of head capsule; in profile, eye set anteriad of midline of head capsule; roughly ovoid, eye narrowed posteriad. In full-face view, frontal carinae straight, divergent posteriad; frontal lobes straight in front of antennal insertion. Anteromedial clypeal margin narrowly protrusive anteromedially, the protrusion with a square border; clypeal psammophore set at or just above anterior clypeal margin; palp formula 3,4. Mandibular teeth in minor worker consisting of five teeth on the masticatory margin, with additional small denticles on the basal margin; mandibles securiform; third mandibular tooth distinctly shorter than apical tooth, but equivalent in length to remaining teeth; masticatory margin of mandibles approximately vertical or weakly oblique. **Mesosoma.** Integument of pronotum, mesonotum and mesopleuron with weak to moderate sheen, shagreenate on pronotum and dorsum of mesonotum, otherwise microreticulate; anterior mesosoma in profile broadly convex; appearance of erect pronotal setae long (i.e., longest erect setae longer than length of eye) and unmodified; in profile, metanotal groove shallow, indicated mainly by an angle; propodeum matt or with a weak sheen and microreticulate; propodeum angulate, propodeal angle blunt; erect propodeal setae always absent; appressed propodeal setulae short, separated by more than own length and inconspicuous; propodeal spiracle situated nearer to midpoint of propodeum than to its declivitous face, and longer (length ≥ 0.50 × height of propodeum). **Petiole.** In profile, petiolar node squamiform; in full-face view, shape of petiolar node rectangular and variably concave medially; node shining and distinctly shagreenate-microreticulate. **Gaster.** Gaster shining, shagreenate (‘LP record’ appearance); pilosity of first gastral tergite consisting of well-spaced short, inconspicuous, appressed setae only, erect setae always absent. ***General characters***. Colour of foreparts orange to dark tan, legs yellowish, gaster chocolate.

#### Major worker description.

**Head.** Head square; posterior margin of head planar or weakly concave; cuticle of frons shining and smooth except for piliferous pits; frons consisting exclusively or almost exclusively of well-spaced, appressed setae only (small, erect setae, if present, usually confined to ocular triangle or posterior margin of head). Eye moderate (eye length 0.20–0.49 length of head capsule); in full-face view, eyes set above midpoint of head capsule; in profile, eye set anteriad of midline of head capsule; eyes elliptical. In full-face view, frontal carinae straight, divergent posteriad; frontal lobes straight in front of antennal insertion. Anterior clypeal margin narrowly protrusive anteromedially, the protrusion with a square border; clypeal psammophore set at or just above anterior clypeal margin; palp formula 3,4. Mandibular teeth in major worker consisting of five teeth on the masticatory margin with additional small denticles on the basal margin; mandibles securiform; third mandibular tooth distinctly shorter than apical tooth, but equivalent in length to remaining teeth; masticatory margin of mandibles approximately aligned vertically or weakly oblique. **Mesosoma.** Integument of pronotum, mesonotum and mesopleuron with weak to moderate sheen, shagreenate on pronotum and dorsum of mesonotum, otherwise microreticulate; anterior mesosoma in profile broadly convex; erect pronotal setae absent; in profile, metanotal groove shallow, indicated mainly by an angle and metathoracic spiracles; propodeum shining and microreticulate; propodeum angulate, propodeal angle blunt; length ratio of propodeal dorsum to its declivity between 1:1 and 1:2; erect propodeal setae absent; appressed propodeal setae short, separated by more than own length and in conspicuous; propodeal spiracle situated nearer to midpoint of propodeum than to its declivitous face, and longer (length ≥ 0.50 × height of propodeum). **Petiole.** In profile, petiolar node squamiform; in full-face view, shape of petiolar node rectangular and variably concave medially; node shining and faintly shagreenate-microreticulate. ***Gaster***. Gaster shining, shagreenate (‘LP record’ appearance); pilosity of first gastral tergite consisting of well-spaced short, inconspicuous, appressed setae only, erect setae always absent. ***General characters***. Colour of foreparts orange to dark tan, legs yellowish, gaster brown.

#### Measurements.

Worker (n = 6): CI 83–100; EI 19–25; EL 0.22–0.25; HL 1.05–1.29; HW 0.87–1.30; ML 1.26–1.59; MTL 0.64–0.89; PpH 0.18–0.20; PpL 0.59–0.64; SI 80–82; SL 0.72–1.04.

#### Comments.

As with the closely related *M. pelecygnathus*, *Melophorus potteri* is a highly distinctive species that cannot be mistaken for any other *Melophorus*. The mandibles are securiform with the basal mandibular margin expanded, and the PF is 3,4. In addition, the ant shares the apomorphies of its group including the large, centrally placed and diagonal propodeal spiracle. The species, although not common, is widely distributed throughout Australia and has been recorded from all mainland states except the ACT and SA. In all likelihood, however, it also occurs in SA, at least. Several samples of the taxon have been available for sequencing, and on the five- and three-gene trees it appears as a sister to *Melophorus pusillus*. However, the two species have a very different appearance, and the placement of *M. potteri* as a sister to the *M. aeneovirens* complex on the highly conserved Wg tree seems more intuitively satisfying in view of the morphology.

*Melophorus potteri* is an obligate termitophage (McAreavey 1947) and is unusual among ants with this lifeway in that the raiding is done on an individual basis. According to McAreavey’s source (Herbert Potter) the individual raiding ants break open the termite mounds and then back out, dragging the adult termite workers with them. The raids take place in temperatures of ‘90°F’ (around 32 degrees Celsius). Although not mentioned by McAreavey, it is tempting to think that the shape of each mandible (which resembles a blunt pair of forceps) may assist the ant’s grip on the soft termite body. Ecological notes suggest this species does not favour any particular habitat – it can be found in the far north and the far south of the continent and in deserts and in wet coastal areas and everywhere in between – and its occurrence may be determined more by the distribution of its favoured prey than climatic factors. Mallee woodland appears to be a common habitat, and savanna woodland and box pine scrub are also mentioned. In several instances, the ant was collected over red soil. Two workers were ‘swept’ (presumably by a butterfly net) at Karonie in the Western Australian goldfields, but the short, squat limbs of the ant do not appear suited to climbing, so the sweep of the net was probably over quite low vegetation in this case.

**Figure 98.**
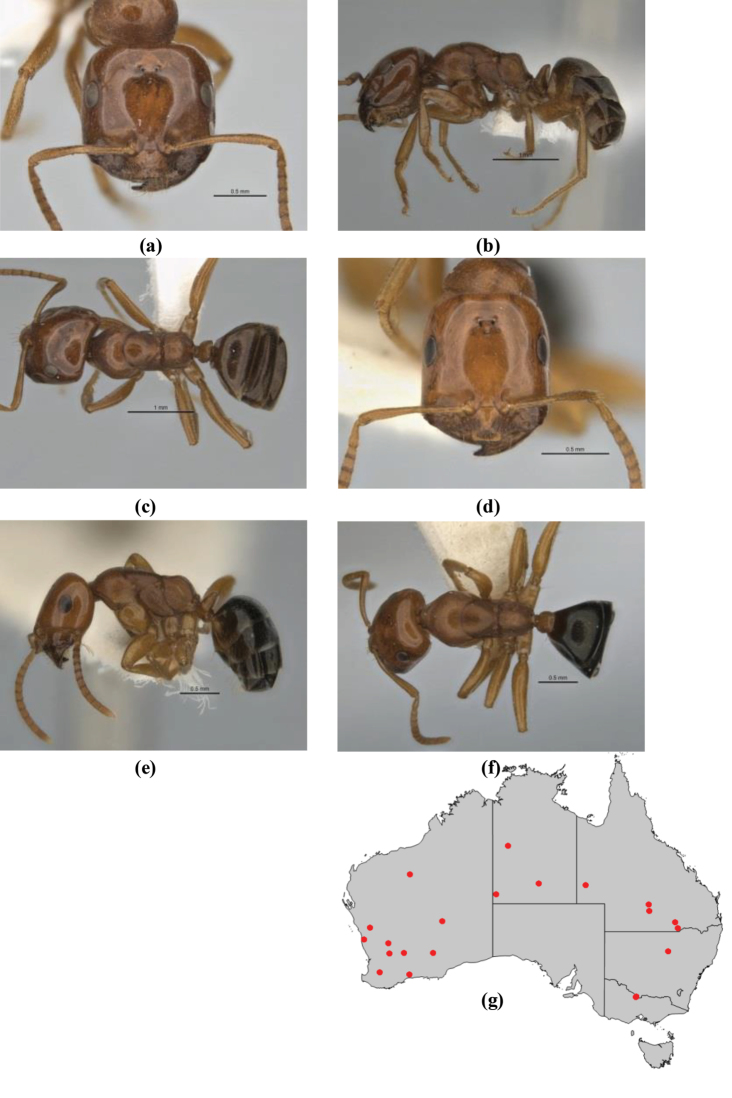
*Melophorus potteri* McAreavey: non–type major worker (ANIC32-900079) frons (**a**), profile (**b**) and dorsum (**c**); non–type minor worker (JDM32-001572) frons (**d**), profile (**e**) and dorsum (**f**); distribution map for the species (**g**). Low resolution scale bars: 1 mm (**b, c**); 0.5 mm (**a, d–f**).

### Species removed from *Melophorus*

**Figure 99.**
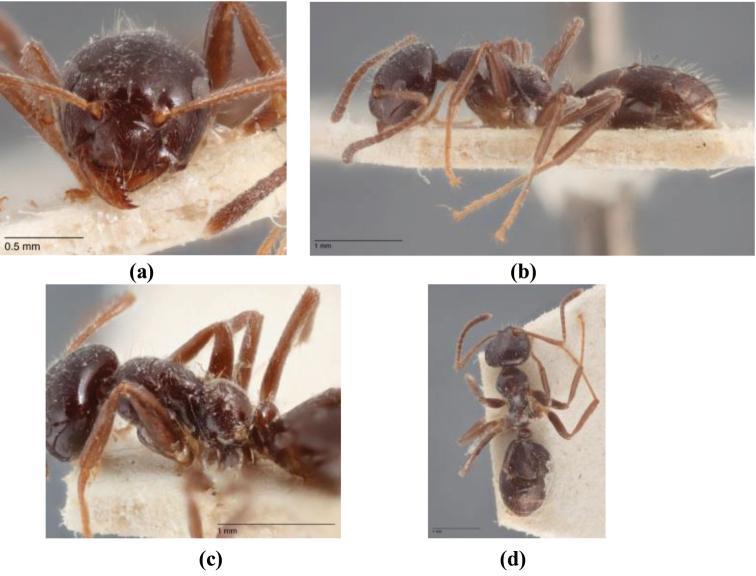
taxon *scipio* here transferred from *Melophorus* to *Prolasius*: sp. n.: SMNH minor worker holotype frons (**a**), profile (**b**), profile (enlargement) and dorsum (**d**). Low resolution scale bars: 1 mm (**b, c, d**); 0.5 mm (**a**).

***Melophorus scipio*** Forel 1915: 86 (combination in *Prolasius* by Wheeler 1935: 71; in *Melophorus* by Taylor & Brown 1985: 124) (Fig. 99a–d).

**Holotype.** Automontage (six views), W, Mt Bellenden Ker, Queensland (SMNH).

**Comments.** The whereabouts of the sole known specimen were unknown for many years, but investigation has recovered the holotype in SMNH, where it was in a different location to the type material. The Automontage photographs reveal a somewhat puzzling mosaic of characters that do not allow the single worker to be easily placed in either *Melophorus* or *Prolasius*. If the taxonomic key to ant genera in Shattuck (1999) is followed, the holotype is also excluded from any other Australian formicine genus. The appearance of the propodeal spiracle, which is circular, is not replicated in any known *Melophorus* and the ant lacks J-shaped setae on the underside of the head capsule (pers. comm. H. Vårdal) and the characteristic psammophore. The type locality–Mt Bellenden Ker, in tropical northern QLD–is also an unlikely site for members of this heat-loving genus. Mt Bellenden Ker receives the highest rainfall of any meteorological station in Australia (http://www.bom.gov.au/announcements/media_releases/ho/010105.shtml). Against easy placement in *Prolasius* is the rounded anterior margin of the clypeus, the extreme hairiness of the scape and the lack of an ocellar triangle (pers. comm. H. Vårdal) usually seen in this genus. However, the ant satisfies the present taxonomic diagnosis for *Prolasius*, though not *Melophorus*, so we here provisionally return *M. scipio* in the genus *Prolasius*. This agrees with Wheeler’s (1934) decision.

## Supplementary Material

XML Treatment for Melophorus

XML Treatment for Melophorus aeneovirens

XML Treatment for Melophorus attenuipes

XML Treatment for Melophorus canus

XML Treatment for Melophorus castaneus

XML Treatment for Melophorus clypeatus

XML Treatment for Melophorus curtus

XML Treatment for Melophorus fulgidus

XML Treatment for Melophorus gibbosus

XML Treatment for Melophorus griseus

XML Treatment for Melophorus kuklos

XML Treatment for Melophorus mullewaensis

XML Treatment for Melophorus platyceps

XML Treatment for Melophorus praesens

XML Treatment for Melophorus rufoniger

XML Treatment for Melophorus sulconotus

XML Treatment for Melophorus tenuis

XML Treatment for Melophorus teretinotus

XML Treatment for Melophorus bagoti

XML Treatment for Melophorus gracilipes

XML Treatment for Melophorus nemophilus

XML Treatment for Melophorus anderseni

XML Treatment for Melophorus andersenioides

XML Treatment for Melophorus chrysus

XML Treatment for Melophorus subulipalpus

XML Treatment for Melophorus argus

XML Treatment for Melophorus biroi

XML Treatment for Melophorus castanopus

XML Treatment for Melophorus compactus

XML Treatment for Melophorus cuneatus

XML Treatment for Melophorus dicyrtos

XML Treatment for Melophorus graciliceps

XML Treatment for Melophorus gracilis

XML Treatment for Melophorus latinotus

XML Treatment for Melophorus lissotriches

XML Treatment for Melophorus longiceps

XML Treatment for Melophorus macrops

XML Treatment for Melophorus microreticulatus

XML Treatment for Melophorus minimus

XML Treatment for Melophorus mjobergi

XML Treatment for Melophorus postlei

XML Treatment for Melophorus propebiroi

XML Treatment for Melophorus turbineus

XML Treatment for Melophorus brevignathus

XML Treatment for Melophorus marmar

XML Treatment for Melophorus quadratus

XML Treatment for Melophorus ankylochaetes

XML Treatment for Melophorus bruneus

XML Treatment for Melophorus eumorphus

XML Treatment for Melophorus fieldi

XML Treatment for Melophorus fulvidus

XML Treatment for Melophorus gilliatensis

XML Treatment for Melophorus hirsutipes

XML Treatment for Melophorus incisus

XML Treatment for Melophorus inconspicuus

XML Treatment for Melophorus isaiah

XML Treatment for Melophorus lanuginosus

XML Treatment for Melophorus longipes

XML Treatment for Melophorus major

XML Treatment for Melophorus microtriches

XML Treatment for Melophorus orthonotus

XML Treatment for Melophorus paramorphomenus

XML Treatment for Melophorus perthensis

XML Treatment for Melophorus sericothrix

XML Treatment for Melophorus setosus

XML Treatment for Melophorus solitudinis

XML Treatment for Melophorus sulla

XML Treatment for Melophorus turneri

XML Treatment for Melophorus vitreus

XML Treatment for Melophorus oblongiceps

XML Treatment for Melophorus brevipalpus

XML Treatment for Melophorus caeruleoviolaceus

XML Treatment for Melophorus cerasinoniger

XML Treatment for Melophorus chauliodon

XML Treatment for Melophorus diversus

XML Treatment for Melophorus hexidens

XML Treatment for Melophorus laticeps

XML Treatment for Melophorus parvimolaris

XML Treatment for Melophorus pelorocephalus

XML Treatment for Melophorus prominens

XML Treatment for Melophorus purpureus

XML Treatment for Melophorus wheeleri

XML Treatment for Melophorus xouthos

XML Treatment for Melophorus barbellulatus

XML Treatment for Melophorus fulvihirtus

XML Treatment for Melophorus hirsutus

XML Treatment for Melophorus ludius

XML Treatment for Melophorus pusillus

XML Treatment for Melophorus translucens

XML Treatment for Melophorus majeri

XML Treatment for Melophorus macroschismus

XML Treatment for Melophorus pelecygnathus

XML Treatment for Melophorus potteri

## Supplementary materials

Supplementary material 1 Phylogenetic species tree for three genes Link: https://doi.org/10.3897/zookeys.700.11784.suppl1 Data type: TIF file Explanation note: Phylogenetic species tree for three genes (COI, WG, LR). Outgroups included *Notoncus gilberti*, *Nylanderia braueri*, *Leptanilla* sp., *Pyramica pulchella*, and *Aenictus* sp. This dataset is made available under the Open Database License (http://opendatacommons.org/licenses/odbl/1.0/). The Open Database License (ODbL) is a license agreement intended to allow users to freely share, modify, and use this Dataset while maintaining this same freedom for others, provided that the original source and author(s) are credited.Brian E. Heterick, Mark Castalanelli, Steve O. Shattuck
