# Corrigendum: Revision of the ant genus *Melophorus* (Hymenoptera, Formicidae). ZooKeys 700: 1–420 (2017). https://doi.org/10.3897/zookeys.700.11784

**DOI:** 10.3897/zookeys.829.33316

**Published:** 2019-03-11

**Authors:** Brian E. Heterick, Mark Castalanelli, Steve O. Shattuck

**Affiliations:** 1 Curtin University of Technology, GPO Box U1987, Perth WA 6845, Australia Curtin University Perth Australia; 2 Western Australian Museum, Locked Bag 49, Welshpool WA 6986, Australia Western Australian Museum Welshpool DC Australia; 3 EcoDiagnostics Pty Ltd, 46–48 Banksia Rd, Welshpool WA 6106, Australia EcoDiagnostics Pty Ltd Welshpool DC Australia; 4 C/o CSIRO Entomology, P.O. Box 1700, Canberra, ACT 2601, Australia CSIRO Canberra Australia

## Abstract

Nil (Corrigendum)

*Melophorusbruneus* was described by McAreavey in 1949. The itemization of the type material for this species on page 235 under ‘Descriptions’ was as follows:

‘**Types**

Holotype minor worker carded with paratype major worker and queen on top card, and paratype major worker and paratype minor worker of bottom card, all on same pin, also paratype major, minor and media workers and queens on two other pins, Nyngan New South Wales [ANIC] (examined: ANIC specimens ANIC32-05344).’

This itemization is incorrect. For some unaccountable reason males on both the holotype pin and one of the two paratype pins were apparently mistaken for minor workers. There is also a small typographical error (‘of bottom card’ should read ‘on bottom card’). The correct itemization should be:

‘**Types**

Holotype minor worker carded with paratype major worker and queen on top card, and paratype major worker and paratype male on bottom card, all on same pin, also paratype major and minor workers, queens and males on a second pin, and paratype major and media workers on a third pin, Nyngan New South Wales [ANIC] (examined: ANIC specimens ANIC32-05344).’

