# Supplementary figures and images for: Revision of the ant genus Melophorus (Hymenoptera, Formicidae)
Source: Zookeys. 2017 Sep 20;(700):1–420. doi: 10.3897/zookeys.700.11784 (PMC5711039; doi:10.3897/zookeys.700.11784)

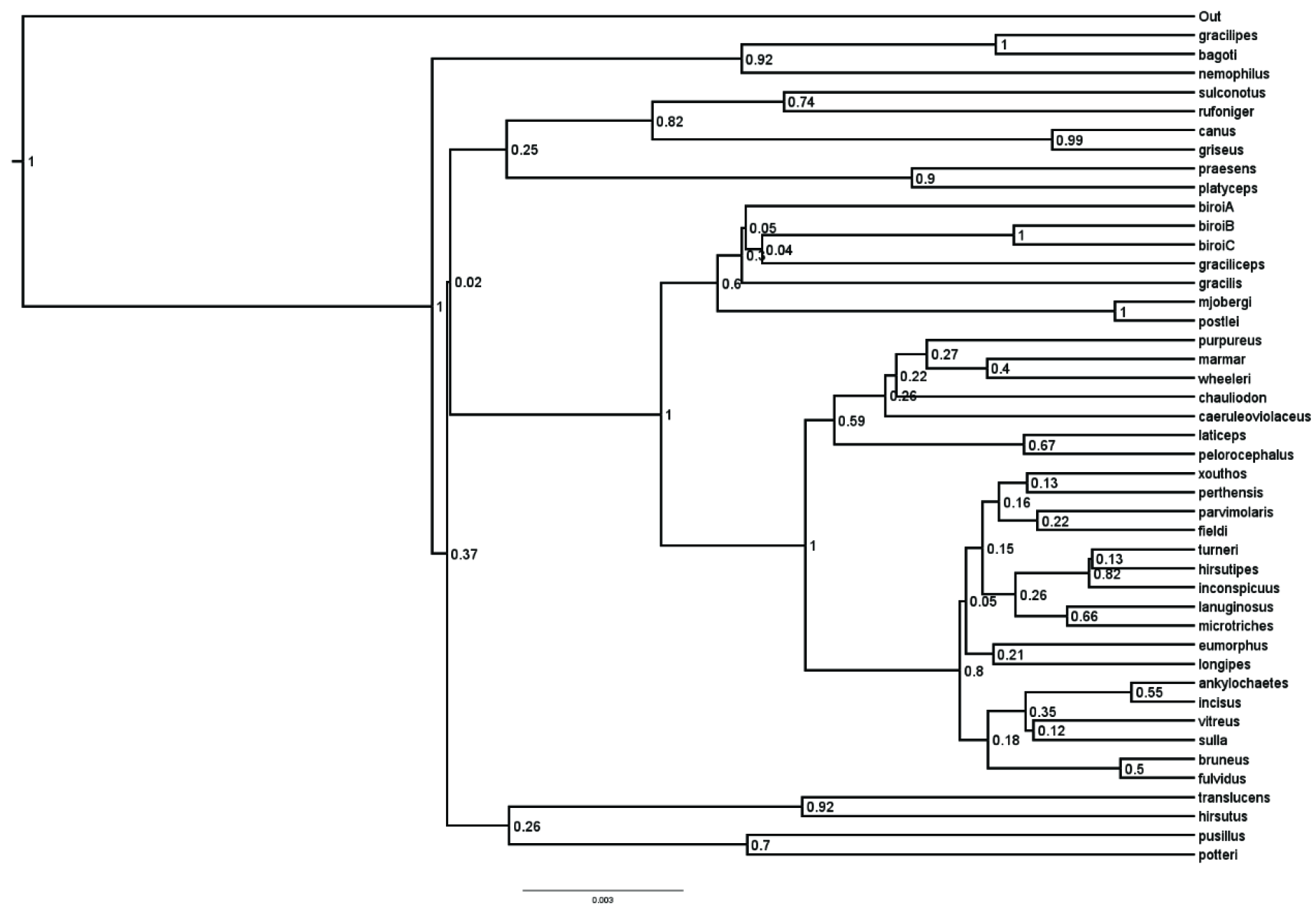

Supplement: Supplementary material 1 — Phylogenetic species tree for three genes [file zookeys-700-001-s001.tif]
